# Versatile Pyridinium Trifluoroborate Platform for Facile Preparation of ^18^F‑Labeled PET Tracers in Water

**DOI:** 10.1021/acscentsci.6c00164

**Published:** 2026-05-06

**Authors:** Wei Li, Yi Li, Tiyi Lyu, Yu Chen, Jingjing Ma, Yi Fang, Li Zhang

**Affiliations:** † The Fourth Affiliated Hospital of Soochow University & Key Laboratory of Organic Synthesis of Jiangsu Province, College of Chemistry, Chemical Engineering and Materials Science, 12582 Soochow University, Suzhou 215123, P. R. China; ‡ NHC Key Laboratory of Nuclear Medicine, Jiangsu Key Laboratory of Molecular Nuclear Medicine, 384648 Jiangsu Institute of Nuclear Medicine, Wuxi 214063, P. R. China

## Abstract

[^18^F]-Organotrifluoroborate salts are important radioprosthetic groups for positron emission tomography (PET) imaging. However, their application is hindered by hydrolytic instability *in vivo*. Several water-tolerant trifluoroborate scaffolds have been developed, but their multistep syntheses restrict structural diversity. Consequently, access to water-stable organotrifluoroborates with broad structural and physicochemical variation remains a challenge. Herein, a library of water-stable trifluoroborate reagents for PET tracer preparation has been developed. The reagents feature one-step synthesis, structural diversity, and high stability under aqueous conditions. The hydrolysis-resistant feature of the reagents enables the preparation of PET tracer precursors in water. Facile ^18^F–^19^F isotope exchange reactions facilitate the preparation of diverse PET tracers with pyridinium [^18^F]­BF_3_ prosthetic groups.

## Graphical abstract

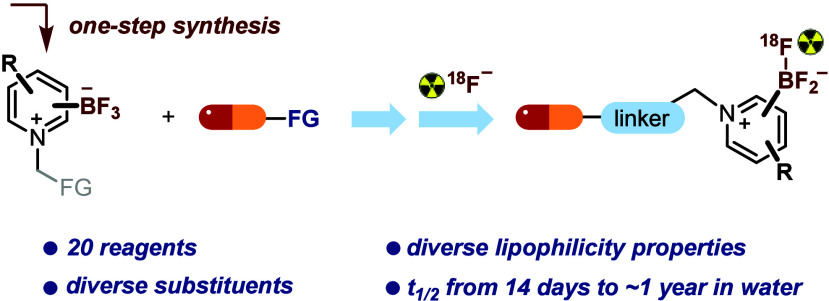

## Introduction

Positron emission tomography (PET), which accurately reveals the *in vivo* distribution of drug molecules, has found broad application in cancer diagnosis.^1^ The technique utilizes PET tracers containing an isotope that emits positrons.^2,3^ Fluorine-18 (^18^F) is a radionuclide whose nuclear decay characteristics are ideally suited for applications in PET imaging.^4–7^ However, one difficulty faced in the synthesis of ^18^F-containing PET tracers is the short half-life of the ^18^F isotope (110 min) as well as the aqueous conditions associated with the [^18^F]­fluoride source. An ideal PET tracer synthesis method requires a highly efficient process to incorporate ^18^F^–^ into functionalized molecules in the presence of water.^8^ Organotrifluoroborate (R-BF_3_^–^) salts have shown promising results in PET tracer synthesis due to the rapid ^18^F–^19^F isotope exchange process in aqueous conditions.^9–17^ However, traditional trifluoroborates are prone to rapid hydrolysis, leading to the decomposition of PET tracers under biological conditions.^18–23^ The development of ammonium-, phosphonium-, and NHC-based trifluoroborate salts^11–17^ has significantly improved the water stability of trifluoroborate structures and enabled their successful application in PET tracer preparation. However, the reagents for conjugation usually require multistep synthesis and may complicate further structural modifications. Herein, we report a new class of water-stable pyridinium trifluoroborate (PyBF_3_) reagents that could introduce trifluoroborate prosthetic groups with diverse physicochemical features. The resulting conjugates bear hydrolysis-resistant BF_3_ units, making them well-suited as precursors for PET tracer synthesis. Facile ^18^F–^19^F isotope exchange (IEX) with aqueous [^18^F]­fluoride serves as a straightforward and practical late-stage radiofluorination, enabling a modular and efficient synthesis of ^18^F-labeled PET tracers.

Previous mechanistic studies of the hydrolysis process of organotrifluoroborates have shown that aryltrifluoroborates with electron-withdrawing groups exhibit greater resistance to hydrolysis compared to those with electron-donating groups.^21–23^ However, even for the *p*-nitrophenyl BF_3_^–^ salt, the half-life is only 42 min in PBS buffer,^21^ which is insufficient for PET imaging (Scheme 1a). Mechanistically, introducing a cationic charge near the BF_3_ group could significantly enhance water stability.^9,10^ As a breakthrough, the Perrin group demonstrated that trifluoroborate reagents bearing an ammoniomethyl group (AMBF_3_) exhibit enhanced water stability (*t*_1/2_ ≈ 15 days in PBS buffer) compared to conventional trifluoroborate salts, making them well-suited for PET imaging (Scheme 1b).^9,11^ The Gabbaï group and the Li group have developed the phosphonium- and NHC-based trifluoroborate conjugates, which also show high stability under aqueous conditions (Scheme 1b).^10,14–17^ The Liu group has developed boronophenylalanine-based trifluoroborates (BBPA), which could be used not only for PET imaging but also for boron neutron capture therapy (BNCT).^24^ Despite these advances, most existing reagents require multistep preparation, and the diversity of structures as well as the physicochemical properties of PET tracers remain highly desirable. Herein, we report a library of pyridinium trifluoroborate reagents with versatile structures, which enable efficient conjugation with a variety of drug derivatives (Scheme 1c). These *N*-alkyl PyBF_3_ reagents are readily accessible via a one-step synthesis and exhibit excellent stability (*t*_1/2_ from 14 days to ∼1 year) in water. Various reagents with distinct substituents and physicochemical properties have been prepared, significantly expanding the reagent library for the introduction of the BF_3_ prosthetic group. Preliminary ^18^F-labeling studies show that PyBF_3_ conjugates undergo efficient ^18^F–^19^F isotope exchange with [^18^F]-fluoride in water, demonstrating the potential of PyBF_3_ reagents to broaden the chemical space for [^18^F]-PET tracer preparation.

**Scheme 1:**
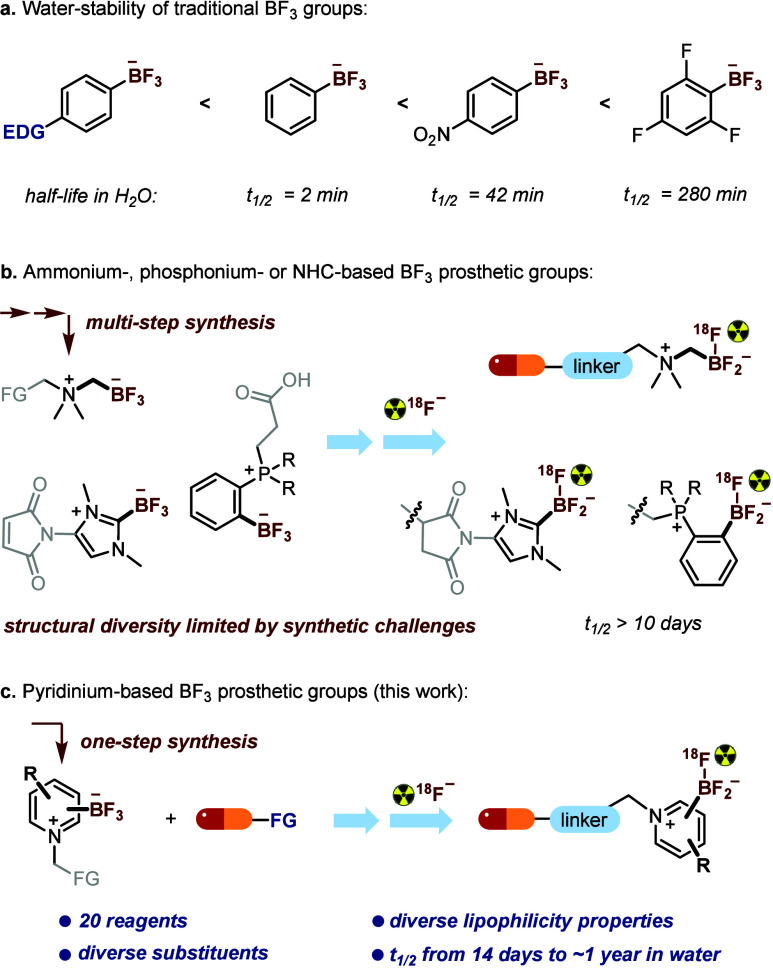
Representative Trifluoroborate Prosthetic Groups for PET Imaging

## Results and Discussion

Our group previously reported a library of water-stable zwitterionic pyridinium trifluoroborate reagents.^25^ The reagents exhibit high water-stability and can be used for Suzuki-Miyaura coupling in the presence of water. Inspired by previous BF_3_ reagents that can be used for conjugation reactions in water,^26^ we envisioned that N-substituted pyridinium BF_3_ reagents could serve as a new library of PET prosthetic groups. The pyridinium N–H moiety^25^ provides a convenient handle for one-step substitution, enabling the rapid synthesis of conjugation-ready reagents bearing substituted alkyl groups, such as alkynyl, azido, and heteroatom-containing substituents.

Experimentally, when pyridinium trifluoroborate was treated with K_2_CO_3_ and alkyl bromides in MeCN, the *N*-alkylpyridinium trifluoroborate reagents were obtained in high yields (Figure 1). Pyridinium trifluoroborate with boron substituents at C2, C3, and C4 positions were successfully prepared (**1a**, **1b**, **1c**). Quinolinium-3-yl trifluoroborate was also prepared (**1e**). The PyBF_3_ reagents with different substituents were also synthesized, including alkyl (**1d**, **1i**), alkoxyl (**1f**), and halogens (**1g**, **1h**, **1k**, **1m**). The zwitterionic reagents can be purified by column chromatography or recrystallization, providing a practical purification method. A gram scale reaction has been performed, and the yield remains similar compared to smaller-scale reactions. The X-ray structures of reagents **1a**–**1c** demonstrate the substituted nitrogen ring and the trifluoroborate structure unambiguously (Figure 1, see Supporting Information for details). In general, based on the substitution reaction, our PyBF_3_ reagent library for cross-coupling reactions was successfully transformed to a reagent library for the introduction of BF_3_ prosthetic groups.

**Figure 1:**
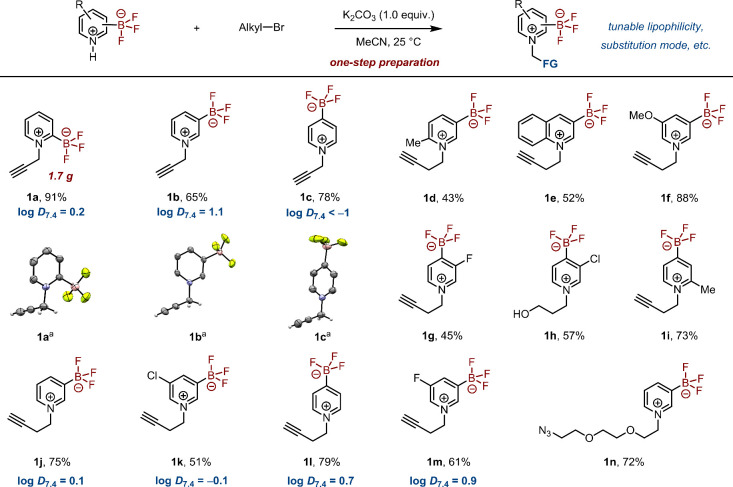
Preparation of *N*-alkylpyridinium trifluoroborate reagents. ^a^The X-ray crystal structures of **1a**–**1c** are shown with 50% probability ellipsoids. The hydrogen atoms on the pyridine ring are omitted for clarity.

Of particular interest, the reagents display distinct and diverse physicochemical properties. Lipophilicity (log *D*) measurements of reagents **1a**–**1c** reveal significant differences in their lipophilic character. For example, the log *D* value of C3-substituted reagent is 1.1, indicating higher lipophilicity, while C4-substituted reagent is highly hydrophilic, with a log *D* value below −1 (Figure 1, see Supporting Information for details). These pronounced differences in lipophilicity highlight the broad chemical and physical diversity within the reagent library.

A stability test of the zwitterionic pyridinium trifluoroborate bearing boron substitution at different positions was also carried out in aqueous conditions (Figure 2a). For example, according to ^19^F NMR monitoring, the half-life of reagent **1b** is more than 30 days in PBS buffer. Neither defluorination nor deboronation is favorable for the decomposition pathways of PyBF_3_ reagents.^21–23^ The Coulombic interaction in **1** inhibits the release of fluoride from the reagents and enhances the stability of the PyBF_3_ reagent, which is consistent with the water- and air-stable properties. It has been reported that the water stability of BF_3_ reagents correlates with their electronic structure.^23^ The pyridinium-2-yl group is significantly more electron-withdrawing than the corresponding 3-yl and 4-yl analogues, which is consistent with the enhanced water stability observed for **1a**. ^19^F NMR monitoring of the solvolysis reaction of reagent **1m** in PBS buffer revealed no detectable mono- or difluorinated species, indicating the transient nature of these intermediates (Figure 2b).^27^ A Lewis acid-promoted B–F bond cleavage reaction^8^ was applied as model reaction for DFT calculation, which revealed the high-energy nature of partially defluorinated intermediates, consistent with the experimental observations (Figure 2c). The HF and fluoride sequestration processes were investigated experimentally by previous mechanistic study.^22^

**Figure 2:**
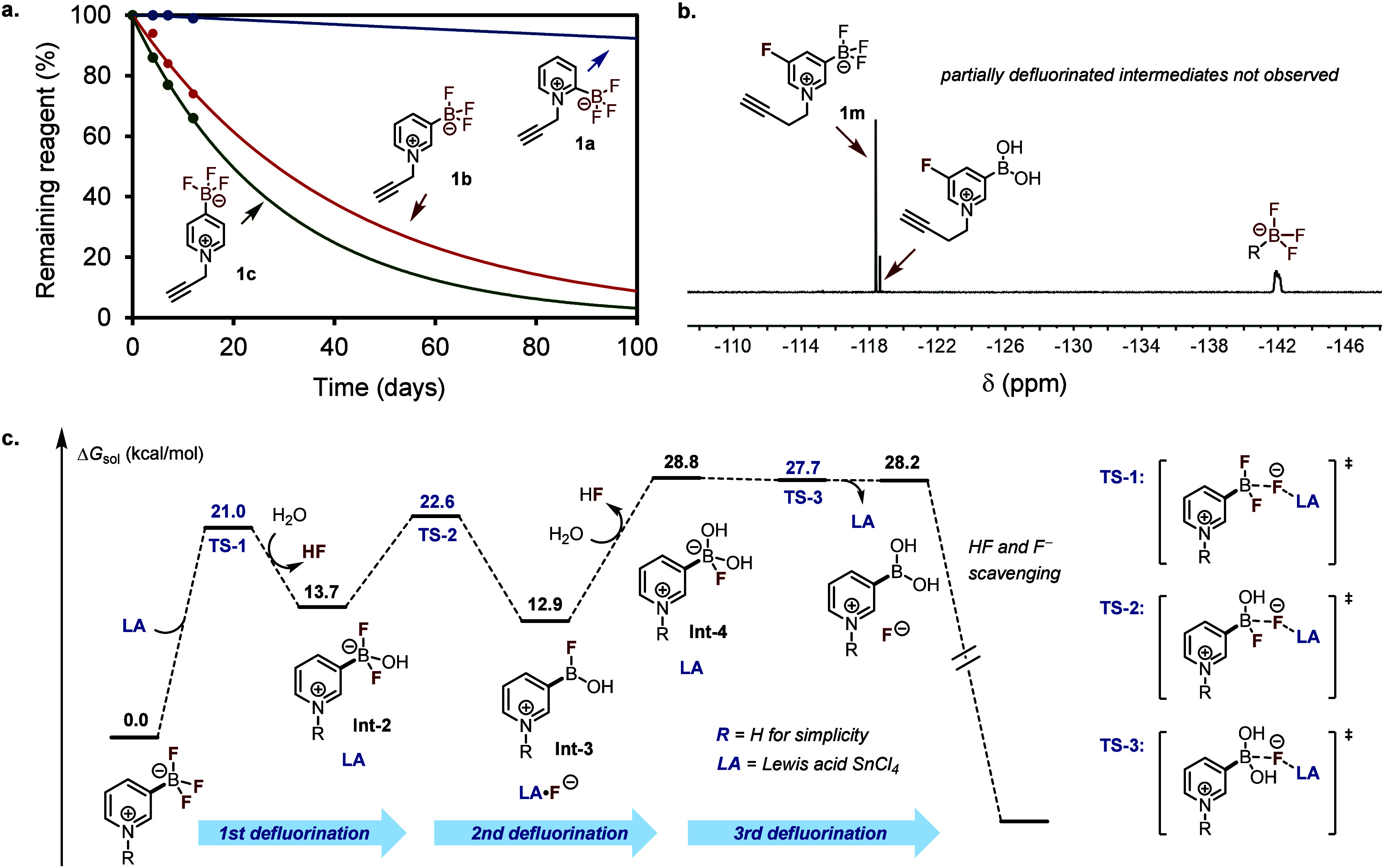
Decomposition of PyBF_3_ reagents in PBS buffer. **a.** The concentration of remaining **1a**–**1c** was determined by ^19^F NMR. **b.** ^19^F NMR analysis of the solvolysis reaction of reagent **1m** in PBS buffer. **c.** Potential surface of the defluorination processes, calculated at PBE0-D3­(BJ)/def2-TZVPP//PBE0-D3­(BJ)/def2-SVP level.

The click reaction provides a modular, water-compatible, and clean reaction for conjugation.^28–31^ The chemistry has also been widely applied in radio medicine precursor preparation.^32–34^ Copper­(I)-catalyzed reaction between terminal alkynes and azides (CuAAC) selectively yields triazole products and is suitable to produce PyBF_3_ conjugates (Figure 3).^35,36^ The click reaction was performed using various PyBF_3_ reagents with alkynyl side chains and benzyl azide, affording the corresponding products in high yields (**3a**-**9a**, **11a**-**13a**). Conversely, reagents bearing an azide group could be reacted with alkynyl-functionalized substrates as an alternative approach (**10a**). Conjugation reactions beyond CuAAC, such as esterification, also proceeded smoothly (**14a**). The results demonstrate the versatility of the PyBF_3_ reagents for the BF_3_-based conjugate preparation.

**Figure 3:**
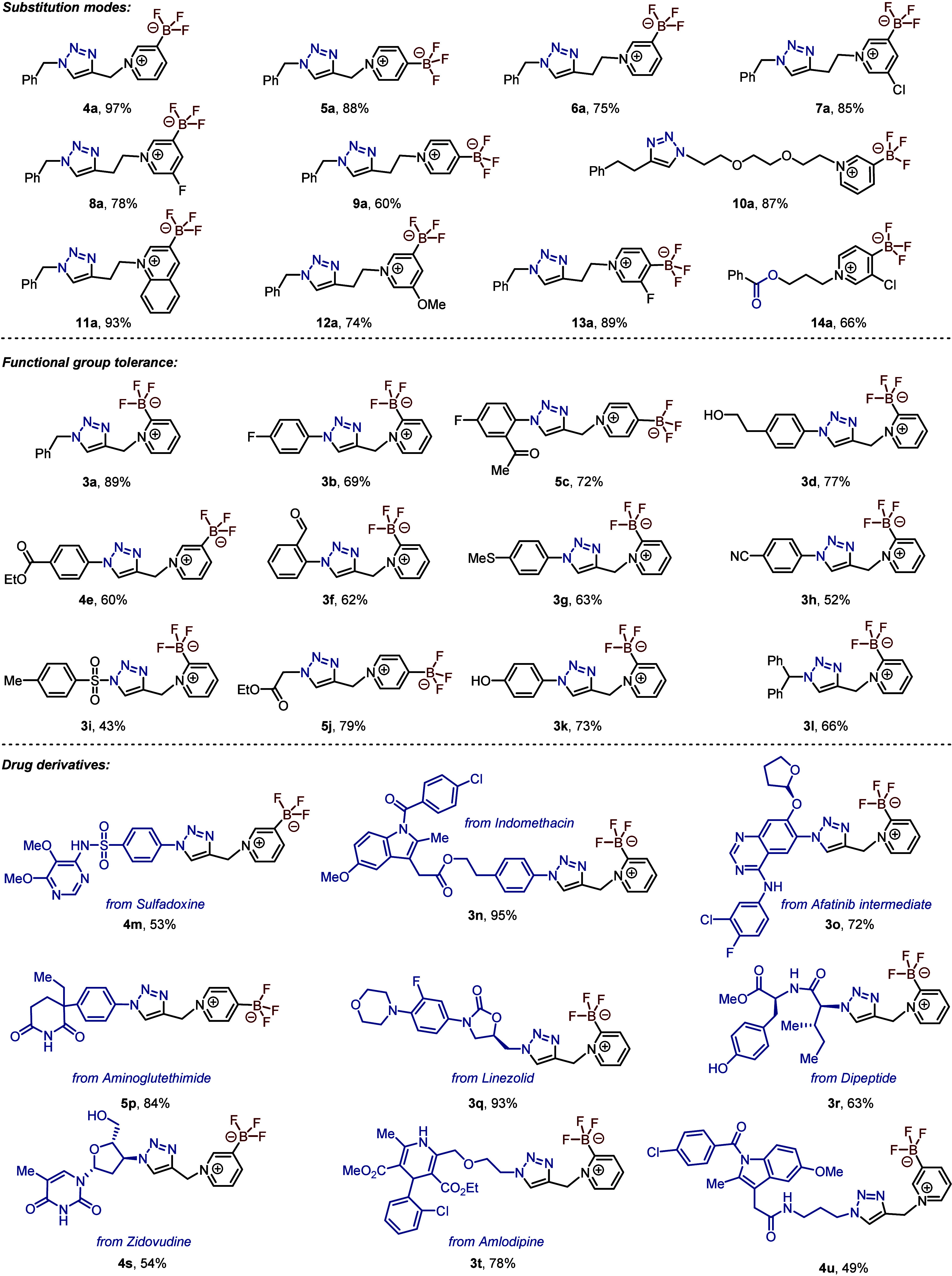
Substrate scope. ^a^Reaction condition: PyBF_3_ (0.3 mmol, 1 equiv), R-N_3_ (0.45 mmol, 1.5 equiv), CuSO_4_·5H_2_O (20 mol %), sodium ascorbate (40 mol %), ^*t*^BuOH:H_2_O (1:1, 0.2 M), 25 °C, 12 h. Yields of isolated products are reported.

A functional group tolerance investigation shows that halogens, alcohols, amines, aldehydes, ketones, esters, amides, and nitriles are all tolerated in the conjugation reactions (**3b**, **5c**, **3d**, **4e**, **3f**, **3h**, **3k**, and **3l**). Under the aqueous CuAAC reaction conditions, the click reactions between the PyBF_3_ reagents and the drug azide derivatives also work well to produce the drug-linker-PyBF_3_ conjugates (Figure 3). Derivatives of drug molecules containing azide groups can be easily prepared from corresponding amine drugs or drug intermediates via azide transfer reactions.^37,38^ Therefore, various drug molecules, such as sulfadoxine, indomethacin, aminoglutethimide, linezolid, zidovudine, amlodipine with a PyBF_3_ prosthetic group, have been prepared (**4m, 3n, 3o, 5p, 3q, 3r, 4s, 3t, 4u**), further demonstrating the utility of the reagents for late-stage modification of drug molecules. All the PyBF_3_ substituents remained intact under aqueous reaction conditions, further demonstrating the water stability of the pyridinium trifluoroborate scaffold.

The pyridinium trifluoroborate auxiliary represents a relatively bulky structural modification compared with direct ^18^F incorporation strategies. While minimal structural perturbation is desirable in certain tracer development paradigms, auxiliary-based labeling approaches provide complementary advantages, particularly in terms of operational simplicity, modularity, and rapid radiolabeling efficiency. We also envision this platform as a complementary strategy, particularly well-suited for rapid tracer development and applications involving medium-to-large bioactive molecules, where the relative steric impact is less pronounced.

Pyridinium trifluoroborate conjugates undergo a rapid ^18^F–^19^F isotope exchange process under acidic conditions to produce ^18^F PET tracers (Figure 4a). One of the advantages of the BF_3_ isotope exchange reaction is its aqueous reaction media, avoiding the tedious procedures to dry the [^18^F]-fluoride to ensure nucleophilicity. The IEX reactions were performed on click products, each bearing a BF_3_ moiety at different positions, using a [^18^F]­fluoride solution at 90 °C in DMF. Among these, click products **4a**–**12a**, **15a** were proved to be effective PET tracer precursors under mild conditions. For instance, treatment of benzyl–PyBF_3_ conjugate **5a** with a 10–12 mCi [^18^F]-fluoride solution afforded **[** ^**18**^ **F]­5a** with a radiochemical conversion (RCC) of 36 ± 2% within 10 min. These results demonstrate the versatility of the PyBF_3_ reagent library as a robust platform for PET tracer synthesis. Mild labeling conditions are necessary for late-stage radiofluorination due to the presence of diverse functional groups in drug molecules (Figure 4a). The click product derived from **1a** exhibited negligible RCC under mild conditions, likely due to the stronger B–F bond character arising from enhanced ion-pair stabilization, which may result in a higher activation barrier for ^18^F–^19^F exchange (see Supporting Information for details).

**Figure 4:**
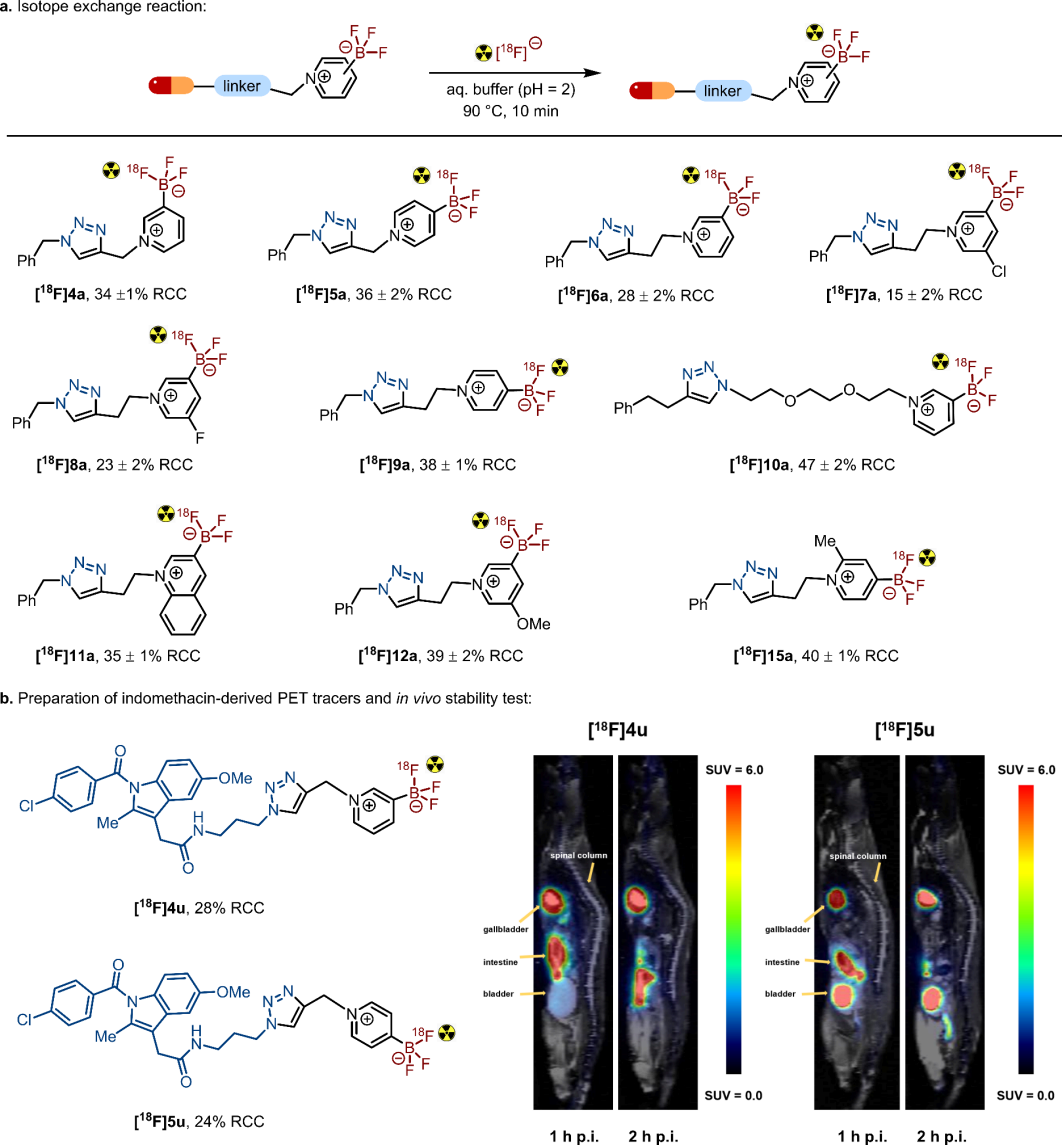
^18^F–^19^F isotope exchange reaction and *in vivo* stability test. **a.** Reaction condition: substrate (300 nmol) in DMF (20 μL), [^18^F]­fluoride (10–12 μCi/μL) in aqueous pyridazine-HCl buffer (40 μL, pH = 2.0), 90 °C for 10 min. RCC = radio-chemical conversion. **b.** Indomethacin-based PET tracers were purified by C18 light cartridge, and ∼7.4 MBq of [^18^F]**4u** or [^18^F]**5u** was used for microPET/MR imaging.

The click products from PyBF_3_ reagents **1b**–**1n** were considered as appropriate PET tracer precursors for ^18^F-labeling. For example, indomethacin-derived pyridinium-3-yl trifluoroborate (**4u**) and pyridinium-4-yl trifluoroborate (**5u**) were successfully converted to [^18^F]-labeled tracers, **[** ^**18**^ **F]­4u** and **[** ^**18**^ **F]­5u**, demonstrating the high functional group tolerance for the ^18^F–^19^F IEX strategy enabled by PyBF_3_ prosthetic groups (Figure 4b, left). We also evaluated the *in vivo* stability of probes **[** ^**18**^ **F]­4u** and **[** ^**18**^ **F]­5u** in a murine model (Figure 4b, right). PET/MR scans acquired at 1 and 2 h postinjection revealed clear accumulation of the conjugates in the gallbladder and their excretory pathways. Notably, no bone uptake was observed even at 2 h postinjection, indicating high *in vivo* stability of the tracers.

## Conclusion

In conclusion, a library of various water-stable PyBF_3_ reagents for PET tracer preparation has been developed. The conjugation reactions of the water-stable PyBF_3_ reagents with drug azide derivatives enable a modular strategy to prepare PET tracer precursors. The PyBF_3_ conjugates also show high efficiency for ^18^F–^19^F isotope exchange reactions. We believe that the facile preparation and the versatility of the reagents render pyridinium trifluoroborate a general and versatile platform for PET tracer synthesis. Further investigation into the radiofluorination reaction of the PyBF_3_ conjugates from various targeted drugs is in progress in our laboratory to make use of the water-stable PET tracer scaffold.

## Supplementary Material





## Notes

Detailed experimental procedures and spectroscopic characterization (PDF)
X-ray data (ZIP)
