# Supplementary material for: Versatile Pyridinium Trifluoroborate Platform for Facile Preparation of 18F‑Labeled PET Tracers in Water
Source: ACS Cent Sci. 2026 May 6;12(6):824–30. doi: 10.1021/acscentsci.6c00164 (PMC13306595; doi:10.1021/acscentsci.6c00164)
Supplement: Supplementary file 1 [file oc6c00164_si_001.pdf]

SUPPORTING INFORMATION

Versatile Pyridinium Trifluoroborate Platform for Facile Preparation of ^{18}F-Labeled PET Tracers in Water

Wei Li[1#], Yi Li[1#], Tiyi Lyu[1], Yu Chen[1], Jingjing Ma[1*], Yi Fang[2*], Li Zhang[1*]

[1] The Fourth Affiliated Hospital of Soochow University & Key Laboratory of Organic Synthesis of Jiangsu Province, College of Chemistry, Chemical Engineering and Materials Science, Soochow University, 215123 Suzhou (P. R. China)

[2] NHC Key Laboratory of Nuclear Medicine, Jiangsu Key Laboratory of Molecular Nuclear Medicine, Jiangsu Institute of Nuclear Medicine, 214063 Wuxi (P. R. China)

[#]These two authors contributed equally to this work

*E-mail: li.zhang@suda.edu.cn (L.Z); fangyi@jsinm.org (Y.F); jingjingmajj@suda.edu.cn (J.M)

TABLE OF CONTENTS

TABLE OF CONTENTS 2

MATERIALS AND METHODS 15

EXPERIMENTAL DATA 16

Preparation of *N*-alkyl $PyBF_3$ reagents for click reactions 16

(1-(Prop-2-yn-1-yl)pyridin-1-ium-2-yl)trifluoroborate (**1a**) 16
(1-(Prop-2-yn-1-yl)pyridin-1-ium-3-yl)trifluoroborate (**1b**) 17
(1-(Prop-2-yn-1-yl)pyridin-1-ium-4-yl)trifluoroborate (**1c**) 17
(1-(But-3-yn-1-yl)-6-methylpyridin-1-ium-3-yl)trifluoroborate (**1d**) 18
(1-(But-3-yn-1-yl)quinolin-1-ium-3-yl)trifluoroborate (**1e**) 19
(1-(But-3-yn-1-yl)-5-methoxypyridin-1-ium-3-yl)trifluoroborate (**1f**) 20
(1-(But-3-yn-1-yl)-3-fluoropyridin-1-ium-4-yl)trifluoroborate (**1g**) 21
(3-Chloro-1-(3-hydroxypropyl)pyridin-1-ium-4-yl)trifluoroborate (**1h**) 22
(1-(But-3-yn-1-yl)-2-methylpyridin-1-ium-4-yl)trifluoroborate (**1i**) 22
(1-(But-3-yn-1-yl)pyridin-1-ium-3-yl)trifluoroborate (**1j**) 23
(1-(But-3-yn-1-yl)-5-chloropyridin-1-ium-3-yl)trifluoroborate (**1k**) 24
(1-(But-3-yn-1-yl)pyridin-1-ium-4-yl)trifluoroborate (**1l**) 25
(1-(But-3-yn-1-yl)-5-fluoropyridin-1-ium-3-yl)trifluoroborate (**1m**) 26
(1-(2-(2-(2-Azidoethoxy)ethoxy)ethyl)pyridin-1-ium-3-yl)trifluoroborate (**1n**) 26
(6-Methyl-1-(prop-2-yn-1-yl)pyridin-1-ium-2-yl)trifluoroborate (**1o**) 27
(4-Fluoro-1-(prop-2-yn-1-yl)pyridin-1-ium-2-yl)trifluoroborate (**1p**) 28
(4-(*Tert*-butyl)-1-(prop-2-yn-1-yl)pyridin-1-ium-2-yl)trifluoroborate (**1q**) 29
(4-Methyl-1-(prop-2-yn-1-yl)pyridin-1-ium-2-yl)trifluoroborate (**1r**) 29
(4-Chloro-1-(prop-2-yn-1-yl)pyridin-1-ium-2-yl)trifluoroborate (**1s**) 30
(4-Methoxy-1-(prop-2-yn-1-yl)pyridin-1-ium-2-yl)trifluoroborate (**1t**) 31

Preparation of PyH-BF_3 reagents 32

(Pyridin-1-ium-3-yl)trifluoroborate (**1b-S**) 32
(Pyridin-1-ium-4-yl)trifluoroborate (**1c-S**) 33
(6-Methylpyridin-1-ium-3-yl)trifluoroborate (**1d-S**) 33
(Quinolin-1-ium-3-yl)trifluoroborate (**1e-S**) 34
(5-Methoxypyridin-1-ium-3-yl)trifluoroborate (**1f-S**) 35
(3-Fluoropyridin-1-ium-4-yl)trifluoroborate (**1g-S**) 35
(3-Chloropyridin-1-ium-4-yl)trifluoroborate (**1h-S**) 36
(2-Methylpyridin-1-ium-4-yl)trifluoroborate (**1i-S**) 37
(5-Chloropyridin-1-ium-3-yl)trifluoroborate (**1k-S**) 37

(5-Fluoropyridin-1-ium-3-yl)trifluoroborate (**1m-S**) 38
(4-Fluoropyridin-1-ium-2-yl)trifluoroborate (**1p-S**) 39

Stability tests 40

Lipophilicity (*D*) measurements 48

X-Ray crystallographic data 50

(1-(Prop-2-yn-1-yl)pyridin-1-ium-2-yl)trifluoroborate (CCDC 2428065) 50
Table S1. Crystallographic data 51
(1-(Prop-2-yn-1-yl)pyridin-1-ium-3-yl)trifluoroborate (CCDC 2457625) 52
Table S2. Crystallographic data 53
(1-(Prop-2-yn-1-yl)pyridin-1-ium-4-yl)trifluoroborate (CCDC 2456372) 54
Table S3. Crystallographic data 55
(4-Methoxy-1-(prop-2-yn-1-yl)pyridin-1-ium-2-yl)trifluoroborate (CCDC 2440358) 56
Table S4. Crystallographic data 57
(4-Chloro-1-(prop-2-yn-1-yl)pyridin-1-ium-2-yl)trifluoroborate (CCDC 2440357) 58
Table S5. Crystallographic data 59

General procedure for the click reaction 60

Preparation of conjugates 60

(1-((1-Benzyl-1*H*-1,2,3-triazol-4-yl)methyl)pyridin-1-ium-2-yl)trifluoroborate (**3a**) 60
(1-((1-Benzyl-1*H*-1,2,3-triazol-4-yl)methyl)pyridin-1-ium-3-yl)trifluoroborate (**4a**) 61
(1-((1-Benzyl-1*H*-1,2,3-triazol-4-yl)methyl)pyridin-1-ium-4-yl)trifluoroborate (**5a**) 61
(1-(2-(1-Benzyl-1*H*-1,2,3-triazol-4-yl)ethyl)pyridin-1-ium-3-yl)trifluoroborate (**6a**) 62
(1-(2-(1-Benzyl-1*H*-1,2,3-triazol-4-yl)ethyl)-5-chloropyridin-1-ium-3-yl)trifluoroborate (**7a**) 63
(1-(2-(1-Benzyl-1*H*-1,2,3-triazol-4-yl)ethyl)-5-fluoropyridin-1-ium-3-yl)trifluoroborate (**8a**) 64
(1-(2-(1-Benzyl-1*H*-1,2,3-triazol-4-yl)ethyl)pyridin-1-ium-4-yl)trifluoroborate (**9a**) 65
(1-(2-(2-(2-(4-Phenethyl-1*H*-1,2,3-triazol-1-yl)ethoxy)ethoxy)ethyl)pyridin-1-ium-3-yl)trifluoroborate (**10a**) 65
(1-(2-(1-Benzyl-1*H*-1,2,3-triazol-4-yl)ethyl)quinolin-1-ium-3-yl)trifluoroborate (**11a**) 66
(1-(2-(1-Benzyl-1*H*-1,2,3-triazol-4-yl)ethyl)-5-methoxypyridin-1-ium-3-yl)trifluoroborate (**12a**) 67
(1-(2-(1-Benzyl-1*H*-1,2,3-triazol-4-yl)ethyl)-3-fluoropyridin-1-ium-4-yl)trifluoroborate (**13a**) 68
(1-(3-(Benzoyloxy)propyl)-3-chloropyridin-1-ium-4-yl)trifluoroborate (**14a**) 69
(1-(2-(1-Benzyl-1*H*-1,2,3-triazol-4-yl)ethyl)-2-methylpyridin-1-ium-4-yl)trifluoroborate (**15a**) 69
(1-(2-(1-Benzyl-1*H*-1,2,3-triazol-4-yl)ethyl)-6-methylpyridin-1-ium-3-yl)trifluoroborate (**16a**) 70
(1-((1-(4-Fluorophenyl)-1*H*-1,2,3-triazol-4-yl)methyl)pyridin-1-ium-2-yl)trifluoroborate (**3b**) 71
(1-((1-(2-Acetyl-4-fluorophenyl)-1*H*-1,2,3-triazol-4-yl)methyl)pyridin-1-ium-4-yl)trifluoroborate (**5c**) 72
(1-((1-(4-(2-Hydroxyethyl)phenyl)-1*H*-1,2,3-triazol-4-yl)methyl)pyridin-1-ium-2-yl)trifluoroborate (**3d**) 73
(1-((1-(4-(Ethoxycarbonyl)phenyl)-1*H*-1,2,3-triazol-4-yl)methyl)pyridin-1-ium-3-yl)trifluoroborate (**4e**) 73

(1-((1-(2-Formylphenyl)-1*H*-1,2,3-triazol-4-yl)methyl)pyridin-1-ium-2-yl)trifluoroborate (**3f**) 74
(1-((1-(4-(Methylthio)phenyl)-1*H*-1,2,3-triazol-4-yl)methyl)pyridin-1-ium-2-yl)trifluoroborate (**3g**) 75
(1-((1-(4-Cyanophenyl)-1*H*-1,2,3-triazol-4-yl)methyl)pyridin-1-ium-2-yl)trifluoroborate (**3h**) 76
(1-((1-Tosyl-1*H*-1,2,3-triazol-4-yl)methyl)pyridin-1-ium-2-yl)trifluoroborate (**3i**) 76
(1-((1-(2-Ethoxy-2-oxoethyl)-1*H*-1,2,3-triazol-4-yl)methyl)pyridin-1-ium-4-yl)trifluoroborate (**5j**) 77
(1-((1-(4-Hydroxyphenyl)-1*H*-1,2,3-triazol-4-yl)methyl)pyridin-1-ium-2-yl)trifluoroborate (**3k**) 78
(1-((1-Benzhydryl-1*H*-1,2,3-triazol-4-yl)methyl)pyridin-1-ium-2-yl)trifluoroborate (**3l**) 79
Sulfadoxine derivative (**4m**) 80
Indomethacin derivative (**3n**) 80
Afatinib intermediate derivative (**3o**) 81
Aminoglutethimide derivative (**5p**) 82
Linezolid derivative (**3q**) 83
Dipeptide derivative (**3r**) 84
Zidovudine derivative (**4s**) 85
Amlodipine derivative (**3t**) 86
Indomethacin derivative (**4u**) 87
Indomethacin derivative (**5u**) 88

Preparation of starting materials 89

1-(2-Azido-5-fluorophenyl)ethanone (**2c**) 89
Indomethacin azide derivative (**2n**) 89
Dipeptide azide derivative (**2r**) 90
Indomethacin azide derivative (**2u**) 91

^{18}F-^{19}F isotope exchange of of the conjugates 93

General Information 93
[^{18}F](1-((1-Benzyl-1*H*-1,2,3-triazol-4-yl)methyl)pyridin-1-ium-3-yl)trifluoroborate (**[^{18}F]4a**) 93
[^{18}F](1-((1-Benzyl-1*H*-1,2,3-triazol-4-yl)methyl)pyridin-1-ium-4-yl)trifluoroborate (**[^{18}F]5a**) 96
[^{18}F](1-(2-(1-Benzyl-1*H*-1,2,3-triazol-4-yl)ethyl)pyridin-1-ium-3-yl)trifluoroborate (**[^{18}F]6a**) 98
[^{18}F](1-(2-(1-Benzyl-1*H*-1,2,3-triazol-4-yl)ethyl)-5-chloropyridin-1-ium-3-yl)trifluoroborate (**[^{18}F]7a**) 100
[^{18}F](1-(2-(1-Benzyl-1*H*-1,2,3-triazol-4-yl)ethyl)-5-fluoropyridin-1-ium-3-yl)trifluoroborate (**[^{18}F]8a**) 102
[^{18}F] (1-(2-(1-Benzyl-1*H*-1,2,3-triazol-4-yl)ethyl)pyridin-1-ium-4-yl)trifluoroborate (**[^{18}F]9a**) 104
[^{18}F](1-(2-(2-(2-(4-Phenethyl-1*H*-1,2,3-triazol-1-yl)ethoxy)ethoxy)ethyl)pyridin-1-ium-3-yl)trifluoroborate (**[^{18}F]10a**) 106
[^{18}F](1-(2-(1-Benzyl-1*H*-1,2,3-triazol-4-yl)ethyl)quinolin-1-ium-3-yl)trifluoroborate (**[^{18}F]11a**) 108
[^{18}F](1-(2-(1-Benzyl-1*H*-1,2,3-triazol-4-yl)ethyl)-5-methoxypyridin-1-ium-3-yl)trifluoroborate (**[^{18}F]12a**) 110
[^{18}F](1-(2-(1-Benzyl-1*H*-1,2,3-triazol-4-yl)ethyl)-2-methylpyridin-1-ium-4-yl)trifluoroborate (**[^{18}F]15a**) 112

Failed substrate 114
[18F]Indomethacin derivative (**[18F]4u**) 116
[18F]Indomethacin derivative (**[18F]5u**) 119
In vivo microPET imaging studies 121
MECHANISTIC STUDIES 122
DFT calculation 122
Methods 122
Results and discussion 122
Calculated coordinates 123
SPECTROSCOPIC DATA 130
^{1}H NMR of **3a** 130
^{13}C NMR of **3a** 131
^{19}F NMR of **3a** 132
^{11}B NMR of **3a** 133
^{1}H NMR of **4a** 134
^{13}C NMR of **4a** 135
^{19}F NMR of **4a** 136
^{11}B NMR of **4a** 137
^{1}H NMR of **5a** 138
^{13}C NMR of **5a** 139
^{19}F NMR of **5a** 140
^{11}B NMR of **5a** 141
^{1}H NMR of **6a** 142
^{13}C NMR of **6a** 143
^{19}F NMR of **6a** 144
^{11}B NMR of **6a** 145
^{1}H NMR of **7a** 146
^{13}C NMR of **7a** 147
^{19}F NMR of **7a** 148

^{11}B NMR of **7a** 149

^{1}H NMR of **8a** 150

^{13}C NMR of **8a** 151

^{19}F NMR of **8a** 152

^{11}B NMR of **8a** 153

^{1}H NMR of **9a** 154

^{13}C NMR of **9a** 155

^{19}F NMR of **9a** 156

^{11}B NMR of **9a** 157

^{1}H NMR of **10a** 158

^{13}C NMR of **10a** 159

^{19}F NMR of **10a** 160

^{11}B NMR of **10a** 161

^{1}H NMR of **11a** 162

^{13}C NMR of **11a** 163

^{19}F NMR of **11a** 164

^{11}B NMR of **11a** 165

^{1}H NMR of **12a** 166

^{13}C NMR of **12a** 167

^{19}F NMR of **12a** 168

^{11}B NMR of **12a** 169

^{1}H NMR of **13a** 170

^{13}C NMR of **13a** 171

^{19}F NMR of **13a** 172

^{11}B NMR of **13a** 173

^{1}H NMR of **14a** 174

^{13}C NMR of **14a** 175

^{19}F NMR of **14a** 176

^{11}B NMR of **14a** 177

^{1}H NMR of **15a** 178

^{13}C NMR of **15a** 179

^{19}F NMR of **15a** 180

^{11}B NMR of **15a** 181

^{1}H NMR of **16a** 182

^{13}C NMR of **16a** 183

^{19}F NMR of **16a** 184

^{11}B NMR of **16a** 185

^{1}H NMR of **3b** 186

^{13}C NMR of **3b** 187

^{19}F NMR of **3b** 188

^{11}B NMR of **3b** 189

^{1}H NMR of **5c** 190

^{13}C NMR of **5c** 191

^{19}F NMR of **5c** 192

^{11}B NMR of **5c** 193

^{1}H NMR of **3d** 194

^{13}C NMR of **3d** 195

^{19}F NMR of **3d** 196

^{11}B NMR of **3d** 197

^{1}H NMR of **4e** 198

^{13}C NMR of **4e** 199

^{19}F NMR of **4e** 200

^{11}B NMR of **4e** 201

^{1}H NMR of **3f** 202

^{13}C NMR of **3f** 203

^{19}F NMR of **3f** 204

^{11}B NMR of **3f** 205

^{1}H NMR of **3g** 206

^{13}C NMR of **3g** 207

^{19}F NMR of **3g** 208

^{11}B NMR of **3g** 209

^{1}H NMR of **3h** 210

^{13}C NMR of **3h** 211

^{19}F NMR of **3h** 212

^{11}B NMR of **3h** 213

^{1}H NMR of **3i** 214

^{13}C NMR of **3i** 215

^{19}F NMR of **3i**........ 216

^{11}B NMR of **3i** 217

^{1}H NMR of **5j** 218

^{13}C NMR of **5j** 219

^{19}F NMR of **5j**........ 220

^{11}B NMR of **5j** 221

^{1}H NMR of **3k** 222

^{13}C NMR of **3k** 223

^{19}F NMR of **3k**........ 224

^{11}B NMR of **3k** 225

^{1}H NMR of **3l** 226

^{13}C NMR of **3l** 227

^{19}F NMR of **3l**........ 228

^{11}B NMR of **3l** 229

^{1}H NMR of **4m** 230

^{13}C NMR of **4m**........ 231

^{19}F NMR of **4m** 232

^{11}B NMR of **4m** 233

^{1}H NMR of **3n** 234

^{13}C NMR of **3n** 235

^{19}F NMR of **3n** 236

^{11}B NMR of **3n** 237

^{1}H NMR of **3o** 238

^{13}C NMR of **3o** 239

^{19}F NMR of **3o** 240

^{11}B NMR of **3o** 241

^{1}H NMR of **5p** 242

^{13}C NMR of **5p** 243

^{19}F NMR of **5p** 244

^{11}B NMR of **5p** 245

^{1}H NMR of **3q** 246

^{13}C NMR of **3q** 247

^{19}F NMR of **3q** 248

^{11}B NMR of **3q** 249

^{1}H NMR of **3r** 250

^{13}C NMR of **3r** 251

^{19}F NMR of **3r** 252

^{11}B NMR of **3r** 253

^{1}H NMR of **4s** 254

^{13}C NMR of **4s** 255

^{19}F NMR of **4s** 256

^{11}B NMR of **4s** 257

^{1}H NMR of **3t** 258

^{13}C NMR of **3t** 259

^{19}F NMR of **3t** 260

^{11}B NMR of **3t** 261

^{1}H NMR of **4u** 262

^{13}C NMR of **4u** 263

^{19}F NMR of **4u** 264

^{11}B NMR of **4u** 265

^{1}H NMR of **5u** 266

^{13}C NMR of **5u** 267

^{19}F NMR of **5u** 268

^{11}B NMR of **5u** 269

^{1}H NMR of **2c** 270

^{13}C NMR of **2c** 271

^{19}F NMR of **2c** 272

^{1}H NMR of **2n** 273

^{13}C NMR of **2n** 274

^{1}H NMR of **2r** 275

^{13}C NMR of **2r** 276

^{1}H NMR of **2u** 277

^{13}C NMR of **2u** 278

^{1}H NMR of **1a** 279

^{19}F NMR of **1a** 280

^{11}B NMR of **1a** 281

^{13}C NMR of **1a** 282

^{1}H NMR of **1b** 283

^{19}F NMR of **1b** 284

^{11}B NMR of **1b** 285

^{13}C NMR of **1b** 286

^{1}H NMR of **1c** 287

^{19}F NMR of **1c** 288

^{11}B NMR of **1c** 289

^{13}C NMR of **1c** 290

^{1}H NMR of **1d** 291

^{19}F NMR of **1d** 292

^{11}B NMR of **1d** 293

^{13}C NMR of **1d** 294

^{1}H NMR of **1e** 295

^{19}F NMR of **1e** 296

^{11}B NMR of **1e** 297

^{13}C NMR of **1e** 298

^{1}H NMR of **1f** 299

^{19}F NMR of **1f** 300

^{11}B NMR of **1f** 301

^{13}C NMR of **1f** 302

^{1}H NMR of **1g** 303

^{19}F NMR of **1g** 304

^{11}B NMR of **1g** 305

^{13}C NMR of **1g** 306

^{1}H NMR of **1h** 307

^{19}F NMR of **1h** 308

^{11}B NMR of **1h** 309

^{13}C NMR of **1h** 310

^{1}H NMR of **1i** 311

^{19}F NMR of **1i** 312

^{11}B NMR of **1i** 313

^{13}C NMR of **1i** 314

^{1}H NMR of **1j** 315

^{19}F NMR of **1j** 316

^{11}B NMR of **1j** 317

^{13}C NMR of **1j** 318

^{1}H NMR of **1k** 319

^{19}F NMR of **1k** 320

^{11}B NMR of **1k** 321

^{13}C NMR of **1k** 322

^{1}H NMR of **1l** 323
^{19}F NMR of **1l** 324
^{11}B NMR of **1l** 325
^{13}C NMR of **1l** 326
^{1}H NMR of **1m** 327
^{19}F NMR of **1m** 328
^{11}B NMR of **1m** 329
^{13}C NMR of **1m** 330
^{1}H NMR of **1n** 331
^{19}F NMR of **1n** 332
^{11}B NMR of **1n** 333
^{13}C NMR of **1n** 334
^{1}H NMR of **1o** 335
^{19}F NMR of **1o** 336
^{11}B NMR of **1o** 337
^{13}C NMR of **1o** 338
^{1}H NMR of **1p** 339
^{19}F NMR of **1p** 340
^{11}B NMR of **1p** 341
^{13}C NMR of **1p** 342
^{1}H NMR of **1q** 343
^{19}F NMR of **1q** 344
^{11}B NMR of **1q** 345
^{13}C NMR of **1q** 346
^{1}H NMR of **1r** 347
^{19}F NMR of **1r** 348
^{11}B NMR of **1r** 349
^{13}C NMR of **1r** 350
^{1}H NMR of **1s** 351

^{19}F NMR of **1s** 352

^{11}B NMR of **1s** 353

^{13}C NMR of **1s** 354

^{1}H NMR of **1t** 355

^{19}F NMR of **1t** 356

^{11}B NMR of **1t** 357

^{13}C NMR of **1t** 358

^{1}H NMR of **1b-S** 359

^{19}F NMR of **1b-S** 360

^{11}B NMR of **1b-S** 361

^{13}C NMR of **1b-S** 362

^{1}H NMR of **1c-S** 363

^{19}F NMR of **1c-S** 364

^{11}B NMR of **1c-S** 365

^{13}C NMR of **1c-S** 366

^{1}H NMR of **1d-S** 367

^{19}F NMR of **1d-S** 368

^{11}B NMR of **1d-S** 369

^{13}C NMR of **1d-S** 370

^{1}H NMR of **1e-S** 371

^{19}F NMR of **1e-S** 372

^{11}B NMR of **1e-S** 373

^{13}C NMR of **1e-S** 374

^{1}H NMR of **1f-S** 375

^{19}F NMR of **1f-S** 376

^{11}B NMR of **1f-S** 377

^{13}C NMR of **1f-S** 378

^{1}H NMR of **1g-S** 379

^{19}F NMR of **1g-S** 380

^{11}B NMR of **1g-S**.....381

^{13}C NMR of **1g-S**.....382

^{1}H NMR of **1h-S**.....383

^{19}F NMR of **1h-S**.....384

^{11}B NMR of **1h-S**.....385

^{13}C NMR of **1h-S**.....386

^{1}H NMR of **1i-S**.....387

^{19}F NMR of **1i-S**.....388

^{11}B NMR of **1i-S**.....389

^{13}C NMR of **1i-S**.....390

^{1}H NMR of **1k-S**.....391

^{19}F NMR of **1k-S**.....392

^{11}B NMR of **1k-S**.....393

^{13}C NMR of **1k-S**.....394

^{1}H NMR of **1m-S**.....395

^{19}F NMR of **1m-S**.....396

^{11}B NMR of **1m-S**.....397

^{13}C NMR of **1m-S**.....398

^{1}H NMR of **1p-S**.....399

^{19}F NMR of **1p-S**.....400

^{11}B NMR of **1p-S**.....401

^{13}C NMR of **1p-S**.....402

REFERENCES.....403

MATERIALS AND METHODS

All reactions were carried out under ambient atmosphere unless otherwise stated and monitored by thin-layer chromatography (TLC). High-resolution mass spectra were obtained using *micrOTOF-Q III* from *Bruker, and GCT Primier* from *Waters*. Concentration under reduced pressure was performed by rotary evaporation at 25–40 °C at an appropriate pressure. Purified compounds were further dried under vacuum (10^{-6} – 10^{-3} bar). Yields refer to purified and spectroscopically pure compounds, unless otherwise stated.

Solvents

Anhydrous solvents in a sealed bottle were purchased from Inno-chem. All deuterated solvents were purchased from Aladdin and *J&K*.

Chromatography

Thin layer chromatography (TLC) was performed using Qingdaohaiyang TLC plates pre-coated with 250 μm thickness silica gel 60 F_{254} plates and visualized by fluorescence quenching under UV light. Flash column chromatography was performed using silica gel (40–63 μm particle size) purchased from Synthware.

Spectroscopy and Instruments

NMR spectra were recorded on a Bruker Avance III HD 400 spectrometer operating at 400 MHz, 376 MHz, 128 MHz, and 101 MHz, for ^{1}H, ^{19}F, ^{11}B, and ^{13}C acquisitions, or an Agilent DD2 600 MHz NMR Spectrometer operating at 600 MHz, and 151 MHz for ^{1}H, and ^{13}C acquisitions; Chemical shifts are reported in ppm with the solvent residual peak as the internal standard. For ^{1}H NMR: $CDCl_3$, δ 7.26, CD_3CN, δ 1.94, DMSO-d_6, δ 2.50. For ^{13}C NMR: $CDCl_3$, δ 77.16, CD_3CN, δ 1.32, DMSO-d_6, δ 39.52[1]. ^{19}F NMR spectra were referenced using a unified chemical shift scale based on the ^{1}H resonance of tetramethylsilane (1% (v/v) solution in the respective solvent). Data is reported as follows: s = singlet, d = doublet, t = triplet, q = quartet, m = multiplet, br = broad; coupling constants in Hz; integration.

Starting materials

All substrates were used as received from commercial suppliers, unless otherwise stated. Chemicals were purchased from *Inno-Chem, J&K, Leyan, Sigma-Aldrich*, *TCI*, *Alfa Aesar*. The azide substrates **2d**, **2e**, **2f**, **2g**, **2h**, **2k**, **2l**, **2m**, **2o**, **2p**, **2t** were prepared according to modified procedures of reported literatures[2,3]. The spectroscopic data of these compounds are consistent with those reported in the literature[4-12]. The (pyridin-1-ium-2-yl)trifluoroborate salts were prepared with our previous procedure[13].

Note: Although we do not have trouble with azide compounds, they are potentially explosive.

EXPERIMENTAL DATA

Preparation of *N*-alkyl $PyBF_3$ reagents for click reactions

(1-(Prop-2-yn-1-yl)pyridin-1-ium-2-yl)trifluoroborate (1a)

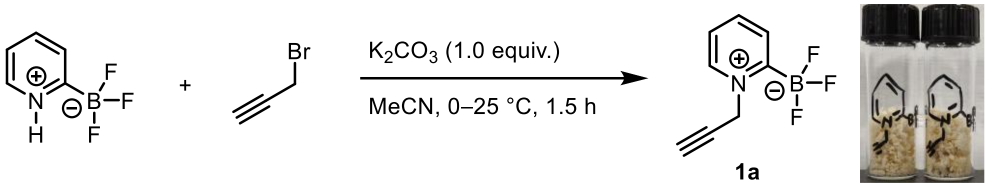

Gram-scale reaction: Under an ambient atmosphere, (pyridin-1-ium-2-yl)trifluoroborate (1.47 g, 10.0 mmol, 1.00 equiv.), K_2CO_3 (1.38 g, 10.0 mmol, 1.00 equiv.), and MeCN (50 mL, *c* = 0.2 M) were added to a 100-mL vial, followed by 3-bromoprop-1-yne (2.38 g, 20.0 mmol, 2.00 equiv.) dropwise using syringe at 0 °C. After stirring for 30 min, the mixture was allowed to stir at room temperature and monitored by TLC every 10 minutes until the formation of side products was observed. Then the mixture was added TFA (2.28 g, 20.0 mmol, 2.00 equiv.) to quench the reaction. After solvation, the solvent was removed under reduced pressure. The residue was purified by chromatography on silica gel, eluting with dichloromethane/methanol (from 100:1 to 50:1, v:v) to afford 1.68 g (91% yield) of the title compound as a pale yellow solid.

Melting Point = 105-106 °C.

$\mathbf{R}_f$ = 0.80 (dichloromethane/methanol, 10:1, v/v (UV))

NMR Spectroscopy:

^{1}**H NMR** (400 MHz, CD_3CN, 25 °C, δ): 8.81 (d, *J* = 6.3 Hz, 1H), 8.26 (t, *J* = 7.7 Hz, 1H), 8.11 (d, *J* = 7.8 Hz, 1H), 7.80 (t, *J* = 6.8 Hz, 1H), 5.51 (d, *J* = 2.6 Hz, 2H), 3.04 (t, *J* = 2.6 Hz, 1H).

^{13}**C {^{1}H} NMR** (101 MHz, CD_3CN, 25 °C, δ): 144.31(2C), 132.61 (q, *J* = 2.9 Hz), 126.88, 79.03, 76.99, 49.24 (q, *J* = 3.9 Hz). The carbon directly attached to the boron atom was not detected due to quadrupolar broadening.

^{19}**F {^{1}H} NMR** (376 MHz, CD_3CN, 25 °C, δ): –143.23 (q, *J* = 40.3 Hz).

^{11}**B NMR** (128 MHz, CD_3CN, 25 °C, δ): 0.59 (q, *J* = 40.3 Hz).

HRMS ESIpos (m/z) calc'd for $C_8H_7NBNaF_3^+$ $[M+Na]^+$, 208.0516; found, 208.0513. Deviation: –1.4 ppm.

(1-(Prop-2-yn-1-yl)pyridin-1-ium-3-yl)trifluoroborate (1b)

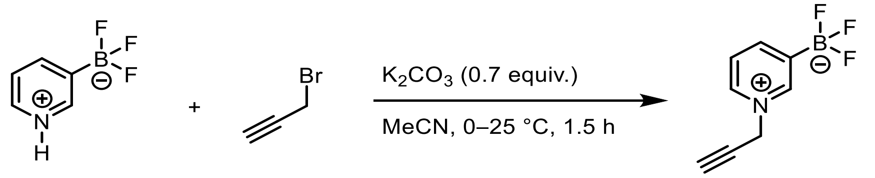

1b

Under an ambient atmosphere, (pyridin-1-ium-3-yl)trifluoroborate (441 mg, 3.00 mmol, 1.00 equiv.), K_2CO_3 (290 mg, 2.10 mmol, 0.700 equiv.), and MeCN (15 mL, *c* = 0.2 M) were added to a 20-mL vial, followed by 3-bromoprop-1-yne (714 mg, 6.00 mmol, 2.00 equiv.) dropwise using syringe at 0 °C. After stirring for 30 min, the mixture was allowed to stir at room temperature and monitored by TLC every 10 minutes until the formation of side products was observed. Then the mixture was added TFA (684 mg, 6.00 mmol, 2.00 equiv.) to quench the reaction. After solvation, the solvent was removed under reduced pressure. Subsequently, 10 mL of diethyl ether was added, the mixture was subjected to sonication, yielding a viscous solid material. The reaction mixture was subjected to ether decantation, followed by the addition of methanol 2 mL and subsequent sonication, which to afford 359 mg (65% yield) of the title compound as a pale yellow solid.

Melting Point = 143-144 °C.

R*f* = 0.50 (dichloromethane/methanol, 10:1, v/v (UV))

NMR Spectroscopy:

^{1}H NMR (400 MHz, CD_3CN, 25 °C, δ): 8.68 (s, 1H), 8.60 (dt, *J* = 6.2, 1.6 Hz, 1H), 8.55 (d, *J* = 7.5 Hz, 1H), 7.88 (t, *J* = 6.8 Hz, 1H), 5.33 (d, *J* = 2.6 Hz, 2H), 3.19 (t, *J* = 2.6 Hz, 1H).

^{13}C {^{1}H} NMR (101 MHz, CD_3CN, 25 °C, δ): 150.39, 146.22, 141.71, 127.95, 80.33, 75.28, 50.60. The carbon directly attached to the boron atom was not detected due to quadrupolar broadening.

^{19}F {^{1}H} NMR (376 MHz, CD_3CN, 25 °C, δ): –144.24 (q, *J* = 44.7 Hz).

^{11}B NMR (128 MHz, CD_3CN, 25 °C, δ): 1.79 (q, *J* = 44.7 Hz).

HRMS ESIpos (m/z) calc'd for $C_8H_7NBNaF_3^+$ $[M+Na]^+$, 208.0516; found, 208.0520. Deviation: +1.9 ppm.

(1-(Prop-2-yn-1-yl)pyridin-1-ium-4-yl)trifluoroborate (1c)

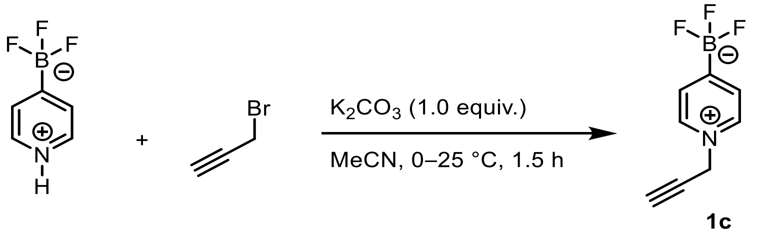

1c

Under an ambient atmosphere, (pyridin-1-ium-4-yl)trifluoroborate (73.5 mg, 0.500 mmol, 1.00 equiv.), K_2CO_3 (69 mg, 0.500 mmol, 1.00 equiv.), and MeCN (2.5 mL, *c* = 0.2 M) were added to a 4-mL vial, followed by 3-bromoprop-1-yne (119 mg, 1.00 mmol, 2.00 equiv.) dropwise using syringe at 0 °C. After stirring for 30 min, the mixture was allowed to stir at room temperature and monitored by TLC every 10 minutes until the formation of side products was observed. Then the mixture was added TFA (114 mg, 1.00 mmol, 2.00 equiv.) to quench the reaction. After solvation, the solvent was removed under reduced pressure. The residue was purified by chromatography on silica gel, eluting with dichloromethane/methanol (from 100:1 to 100:1, v:v) to afford 72.3 mg (78% yield) of the title compound as a pale yellow solid. The spectroscopic data are consistent with those previously reported[14].

Melting Point = 103-104 °C.

R*f* = 0.50 (dichloromethane/methanol, 10:1, v/v (UV))

NMR Spectroscopy:

^{1}H NMR (400 MHz, CD_3CN, 25 °C, δ): 8.54 (d, *J* = 6.1 Hz, 2H), 8.06 (d, *J* = 5.9 Hz, 2H), 5.30 (d, *J* = 2.6 Hz, 2H), 3.19 (t, *J* = 2.6 Hz, 1H).

^{13}C {^{1}H} NMR (101 MHz, CD_3CN, 25 °C, δ): 141.69, 131.52 (q, *J* = 1.8 Hz), 80.41, 75.14, 50.20. The carbon directly attached to the boron atom was not detected due to quadrupolar broadening.

^{19}F {^{1}H} NMR (376 MHz, CD_3CN, 25 °C, δ): –145.90 (q, *J* = 45.1 Hz).

^{11}B NMR (128 MHz, CD_3CN, 25 °C, δ): 1.63 (q, *J* = 45.1 Hz).

HRMS ESIpos (m/z) calc'd for $C_8H_7NBNaF_3^+$ $[M+Na]^+$, 208.0516; found, 208.0519. Deviation: +1.4 ppm.

Discussion: The previous method for the preparation of compound **1c** relied on the reaction of a pyridyl trifluoroborate potassium salt. However, such heteroaryl trifluoroborate potassium salts lack a well-developed reagent library. We believe that the high stability of zwitterionic N–H heteroaryl trifluoroborate reagents enables the derivatization to a variety of N-alkyl pyridinium trifluoroborate reagents, thereby leading to the successful establishment of a prosthetic group library.

(1-(But-3-yn-1-yl)-6-methylpyridin-1-ium-3-yl)trifluoroborate (1d)

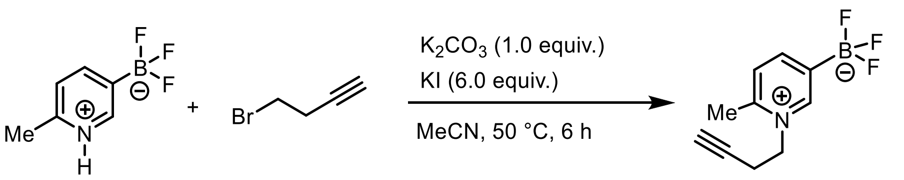

1d

Under an ambient atmosphere, (6-methylpyridin-1-ium-3-yl)trifluoroborate (322 mg, 2.00 mmol, 1.00 equiv.), K_2CO_3 (276 mg, 2.00 mmol, 1.00 equiv.), KI (1.99 g, 12.0 mmol, 6.00 equiv.), and MeCN (10.0 mL, *c* = 0.2 M) were added to a 20-mL vial, followed by 4-bromobut-1-yne (532 mg, 4.00 mmol, 2.00 equiv.). After solvation,

the reaction mixture was heated at 50 °C for 6 h. After solvation, the solvent was removed under reduced pressure. The residue was purified by chromatography on silica gel, eluting with dichloromethane/methanol (from 100:1 to 50:1, v:v) to afford 185 mg (43% yield) of the title compound as a white solid.

Melting Point = 154-155 °C.

R*f* = 0.60 (dichloromethane/methanol, 10:1, v/v (UV))

NMR Spectroscopy:

^{1}**H NMR** (400 MHz, DMSO-*d*$_6$, 25 °C, δ): 8.61 (s, 1H), 8.26 (d, *J* = 7.7 Hz, 1H), 7.80 (d, *J* = 7.7 Hz, 1H), 4.68 (t, *J* = 6.7 Hz, 2H), 3.32 (s, 1H), 2.87 (td, *J* = 6.6, 2.7 Hz, 2H), 2.78 (s, 3H).

^{13}**C {^{1}H} NMR** (101 MHz, DMSO-*d*$_6$, 25 °C, δ): 151.69, 148.38, 146.85, 128.30, 79.33, 74.70, 53.95, 19.29, 19.21. The carbon directly attached to the boron atom was not detected due to quadrupolar broadening.

^{19}**F {^{1}H} NMR** (376 MHz, DMSO-*d*$_6$, 25 °C, δ): –140.30 (brs).

^{11}**B NMR** (128 MHz, DMSO-*d*$_6$, 25 °C, δ): 1.99 (brs).

HRMS ESIpos (m/z) calc'd for $C_{10}H_{11}NBNaF_3^+$ [M+Na]$^+$, 236.0829; found, 236.0829. Deviation: 0.0 ppm.

(1-(But-3-yn-1-yl)quinolin-1-ium-3-yl)trifluoroborate (1e)

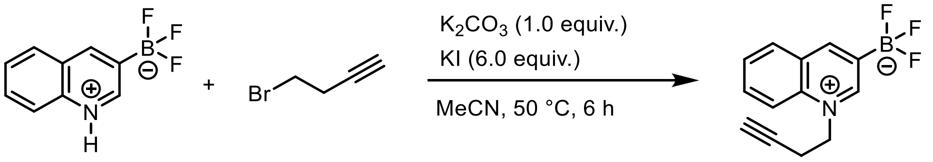

1e

Under an ambient atmosphere, (quinolin-1-ium-3-yl)trifluoroborate (197 mg, 1.00 mmol, 1.00 equiv.), K_2CO_3 (138 mg, 1.00 mmol, 1.00 equiv.), KI (996 mg, 6.00 mmol, 6.00 equiv.), and MeCN (5.0 mL, *c* = 0.2 M) were added to a 20-mL vial, followed by 4-bromobut-1-yne (266 mg, 2.00 mmol, 2.00 equiv.). After solvation, the reaction mixture was heated at 50 °C for 6 h. After solvation, the solvent was removed under reduced pressure. The residue was purified by chromatography on silica gel, eluting with dichloromethane/methanol (from 100:1 to 50:1, v:v) to afford 130 mg (52% yield) of the title compound as a white solid.

Melting Point = 244-245 °C.

R*f* = 0.60 (dichloromethane/methanol, 10:1, v/v (UV))

NMR Spectroscopy:

^{1}**H NMR** (400 MHz, DMSO-*d*$_6$, 25 °C, δ): 9.24 (s, 1H), 9.08 (s, 1H), 8.49 (d, *J* = 9.0 Hz, 1H), 8.42 (dd, *J* = 8.3, 1.6 Hz, 1H), 8.16 – 8.07 (m, 1H), 7.92 (t, *J* = 7.2 Hz, 1H), 5.23 (t, *J* = 6.3 Hz, 2H), 3.08 – 2.86 (m,

3H).

^{13}C {^{1}H} NMR (101 MHz, DMSO-d_6, 25 °C, δ): 152.56, 149.76 (q, J = 1.8 Hz), 136.18, 133.96, 130.26, 129.56, 128.91, 118.27, 79.31, 75.35, 54.23, 19.29. The carbon directly attached to the boron atom was not detected due to quadrupolar broadening.

^{19}F {^{1}H} NMR (376 MHz, CD_3CN, 25 °C, δ): –143.57 (q, J = 45.3 Hz).

^{11}B NMR (128 MHz, CD_3CN, 25 °C, δ): 2.06 (q, J = 45.3 Hz).

HRMS ESIpos (m/z) calc'd for $C_{13}H_{11}NBNaF_3^+$ [M+Na]$^+$, 272.0829; found, 272.0827. Deviation: –0.7 ppm.

(1-(But-3-yn-1-yl)-5-methoxypyridin-1-ium-3-yl)trifluoroborate (1f)

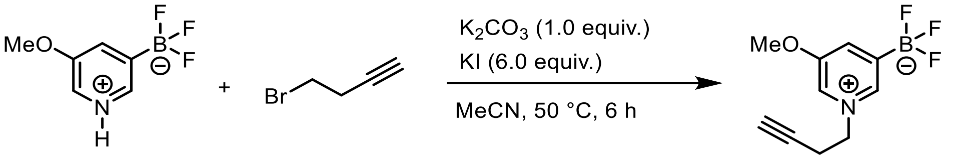

1f

Under an ambient atmosphere, (5-methoxypyridin-1-ium-3-yl)trifluoroborate (177 mg, 1.00 mmol, 1.00 equiv.), K_2CO_3 (138 mg, 1.00 mmol, 1.00 equiv.), KI (996 mg, 6.00 mmol, 6.00 equiv.), and MeCN (5.0 mL, c = 0.2 M) were added to a 20-mL vial, followed by 4-bromobut-1-yne (266 mg, 2.00 mmol, 2.00 equiv.). After solvation, the reaction mixture was heated at 50 °C for 6 h. After that, the solvent was removed by rotary evaporation. The residue was dissolved in ca. 1 mL MeOH, and precipitated with ca. 20 mL Et_2O. The suspension was decanted, and the solid was dried *in vacuo* to afford 202 mg (88% yield) of the title compound as a white solid.

Melting Point = 198-199 °C.

R_f = 0.60 (dichloromethane/methanol, 10:1, v/v (UV))

NMR Spectroscopy:

^{1}H NMR (400 MHz, CD_3CN, 25 °C, δ): 8.25 (s, 1H), 8.13 (dd, J = 2.7, 1.5 Hz, 1H), 8.03 (s, 1H), 4.53 (t, J = 6.6 Hz, 2H), 3.96 (s, 3H), 2.89 (td, J = 6.6, 2.7 Hz, 2H), 2.38 (t, J = 2.7 Hz, 1H).

^{13}C {^{1}H} NMR (101 MHz, DMSO-d_6, 25 °C, δ): 157.06, 139.10, 132.25, 129.43, 79.40, 74.77, 58.07, 56.73, 20.46. The carbon directly attached to the boron atom was not detected due to quadrupolar broadening.

^{19}F {^{1}H} NMR (376 MHz, CD_3CN, 25 °C, δ): –144.13 (q, J = 44.8 Hz).

^{11}B NMR (128 MHz, CD_3CN, 25 °C, δ): 1.77 (q, J = 44.8 Hz).

HRMS ESIpos (m/z) calc'd for $C_{10}H_{11}NBNaOF_3^+$ [M+Na]$^+$, 252.0778; found, 252.0779. Deviation: +0.4 ppm.

(1-(But-3-yn-1-yl)-3-fluoropyridin-1-ium-4-yl)trifluoroborate (1g)

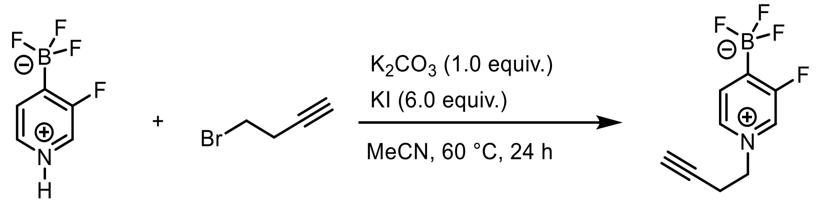

1g

Under an ambient atmosphere, (3-fluoropyridin-1-ium-4-yl)trifluoroborate (330 mg, 2.00 mmol, 1.00 equiv.), K_2CO_3 (276 mg, 2.00 mmol, 1.00 equiv.), KI (1.99 g, 12.0 mmol, 6.00 equiv.), and MeCN (10.0 mL, *c* = 0.2 M) were added to a 20-mL vial, followed by 4-bromobut-1-yne (532 mg, 4.00 mmol, 2.00 equiv.). After solvation, the reaction mixture was heated at 60 °C for 24 h. After that, the solvent was removed under reduced pressure. The residue was purified by chromatography on silica gel, eluting with dichloromethane/methanol (from 100:1 to 50:1, v:v) to afford 195 mg (45% yield) of the title compound as a pale yellow solid.

Melting Point = 210-211 °C.

R*f* = 0.60 (dichloromethane/methanol, 10:1, v/v (UV))

NMR Spectroscopy:

^{1}H NMR (400 MHz, CD_3CN, 25 °C, δ): 8.44 (s, 1H), 8.36 (d, *J* = 5.7 Hz, 1H), 8.04 (s, 1H), 4.56 (t, *J* = 6.5 Hz, 2H), 2.87 (td, *J* = 6.4, 2.5 Hz, 2H), 2.39 (t, *J* = 2.7 Hz, 1H).

^{13}C {^{1}H} NMR (101 MHz, DMSO-*d*6, 25 °C, δ): 162.73 (d, *J* = 246.7 Hz), 139.62 (d, *J* = 3.3 Hz), 132.06 (d, *J* = 43.6 Hz), 131.97 (dq, *J* = 13.5, 2.0 Hz), 79.12, 74.88, 58.24, 20.31. The carbon directly attached to the boron atom was not detected due to quadrupolar broadening.

^{19}F {^{1}H} NMR (376 MHz, CD_3CN, 25 °C, δ): –113.27 (1F), –142.73 (q, *J* = 39.2 Hz, 3F).

^{11}B NMR (128 MHz, DMSO-*d*6, 25 °C, δ): 1.26 (brs).

HRMS ESIpos (m/z) calc'd for $C_9H_8NBNaF_4^+$ $[M+Na]^+$, 240.0578; found, 240.0578. Deviation: 0.0 ppm.

(3-Chloro-1-(3-hydroxypropyl)pyridin-1-ium-4-yl)trifluoroborate (1h)

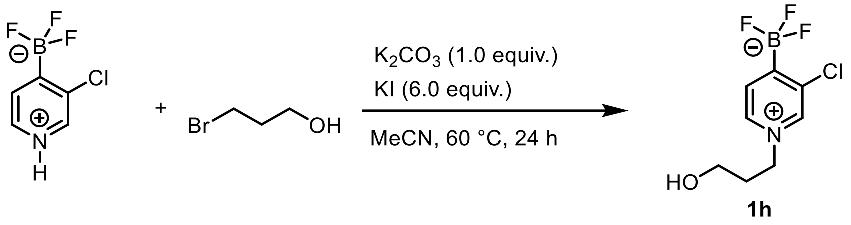

Under an ambient atmosphere, (3-chloropyridin-1-ium-4-yl)trifluoroborate (181 mg, 1.00 mmol, 1.00 equiv.), K_2CO_3 (138 mg, 1.00 mmol, 1.00 equiv.), KI (996 mg, 6.00 mmol, 6.00 equiv.), and MeCN (10.0 mL, *c* = 0.2 M) were added to a 20-mL vial, followed by 3-bromopropanol (278 mg, 2.00 mmol, 2.00 equiv.). After solvation, the reaction mixture was heated at 60 °C for 24 h. After that, the solvent was removed under reduced pressure. The residue was purified by chromatography on silica gel, eluting with dichloromethane/methanol (from 100:1 to 50:1, v:v) to afford 136.2 mg (57% yield) of the title compound as a pale yellow solid.

Melting Point = 128-129 °C.

R_f = 0.60 (dichloromethane/methanol, 3:1, v/v (UV))

NMR Spectroscopy:

^{1}H NMR (400 MHz, CD_3CN, 25 °C, δ): 8.58 (s, 1H), 8.40 (d, *J* = 5.9 Hz, 1H), 8.01 (d, *J* = 5.6 Hz, 1H), 4.53 (t, *J* = 7.0 Hz, 2H), 3.52 (t, *J* = 5.8 Hz, 2H), 2.89 (s, 1H), 2.10 (p, *J* = 6.8 Hz, 2H).

^{13}C {^{1}H} NMR (101 MHz, CD_3CN, 25 °C, δ): 142.56, 141.14, 139.75, 132.58 (q, *J* = 2.9 Hz), 59.27, 58.40, 33.73. The carbon directly attached to the boron atom was not detected due to quadrupolar broadening.

^{19}F {^{1}H} NMR (376 MHz, CD_3CN, 25 °C, δ): –144.17 (q, *J* = 42.6 Hz).

^{11}B NMR (128 MHz, CD_3CN, 25 °C, δ): 1.26 (q, *J* = 42.6 Hz).

HRMS ESIpos (m/z) calc'd for $C_8H_{10}NClBNaOF_3^+$ $[M+Na]^+$, 262.0388; found, 262.0383. Deviation: –1.9 ppm.

(1-(But-3-yn-1-yl)-2-methylpyridin-1-ium-4-yl)trifluoroborate (1i)

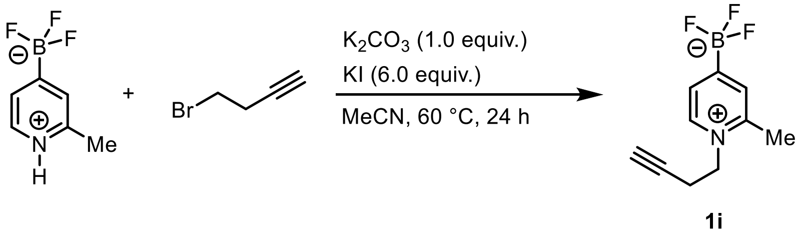

Under an ambient atmosphere, (2-methylpyridin-1-ium-4-yl)trifluoroborate (294 mg, 2.00 mmol, 1.00 equiv.),

K_2CO_3 (276 mg, 2.00 mmol, 1.00 equiv.), KI (1.99 g, 12.0 mmol, 6.00 equiv.), and MeCN (10.0 mL, *c* = 0.2 M) were added to a 20-mL vial, followed by 4-bromobut-1-yne (532 mg, 4.00 mmol, 2.00 equiv.). After solvation, the reaction mixture was heated at 60 °C for 24 h. After that, the solvent was removed by rotary evaporation. The residue was dissolved in ca. 2 mL MeOH, and precipitated with ca. 40 mL Et_2O. The suspension was decanted, and the solid was dried *in vacuo* to afford 155 mg (73% yield) of the title compound as a white solid.

Melting Point = 217-218 °C.

R_f = 0.70 (dichloromethane/methanol, 10:1, v/v (UV))

NMR Spectroscopy:

^{1}H NMR (400 MHz, CD_3CN, 25 °C, δ): 8.41 (d, *J* = 6.1 Hz, 1H), 7.86 (s, 1H), 7.80 (d, *J* = 6.1 Hz, 1H), 4.55 (t, *J* = 6.6 Hz, 2H), 2.84 (td, *J* = 6.6, 2.7 Hz, 2H), 2.75 (s, 3H), 2.37 (t, *J* = 2.7 Hz, 1H).

^{13}C {^{1}H} NMR (101 MHz, DMSO-d_6, 25 °C, δ): 151.73, 142.72, 131.90 (q, *J* = 2.0 Hz), 127.42 (q, *J* = 1.9 Hz), 79.38, 74.77, 53.90, 19.20, 19.08. The carbon directly attached to the boron atom was not detected due to quadrupolar broadening.

^{19}F {^{1}H} NMR (376 MHz, CD_3CN, 25 °C, δ): –145.82 (q, *J* = 45.9 Hz).

^{11}B NMR (128 MHz, CD_3CN, 25 °C, δ): 1.69 (q, *J* = 45.9 Hz).

HRMS ESIpos (m/z) calc'd for $C_{10}H_{11}NBNaF_3^+$ [M+Na]$^+$, 236.0829; found, 236.0828. Deviation: –0.4 ppm.

(1-(But-3-yn-1-yl)pyridin-1-ium-3-yl)trifluoroborate (1j)

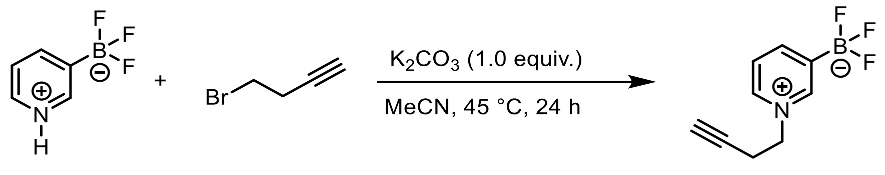

Under an ambient atmosphere, (pyridin-1-ium-3-yl)trifluoroborate (294 mg, 2.00 mmol, 1.00 equiv.), K_2CO_3 (276 mg, 2.00 mmol, 1.00 equiv.), and MeCN (10.0 mL, *c* = 0.2 M) were added to a 20-mL vial, followed by 4-bromobut-1-yne (532 mg, 4.00 mmol, 2.00 equiv.). After solvation, the reaction mixture was heated at 45 °C for 24 h. After that, the solvent was removed under reduced pressure. The residue was purified by chromatography on silica gel, eluting with dichloromethane/methanol (from 100:1 to 50:1, v:v) to afford 297 mg (75% yield) of the title compound as a pale yellow solid.

Melting Point = 137-138 °C.

R*f* = 0.50 (dichloromethane/methanol, 10:1, v/v (UV))

NMR Spectroscopy:

^{1}H NMR (400 MHz, CD_3CN, 25 °C, δ): 8.60 (s, 1H), 8.50 (d, *J* = 7.5 Hz, 1H), 8.45 (dt, *J* = 6.2, 1.6 Hz, 1H), 7.82 (t, *J* = 6.8 Hz, 1H), 4.56 (t, *J* = 6.5 Hz, 2H), 2.88 (td, *J* = 6.5, 2.7 Hz, 2H), 2.36 (t, *J* = 2.7 Hz, 1H).

^{13}C {^{1}H} NMR (101 MHz, CD_3CN, 25 °C, δ): 149.85, 146.83, 142.40, 127.57, 79.46, 73.98, 59.80, 21.62.

The carbon directly attached to the boron atom was not detected due to quadrupolar broadening.

^{19}F {^{1}H} NMR (376 MHz, CD_3CN, 25 °C, δ): –144.27 (q, *J* = 44.7 Hz).

^{11}B NMR (128 MHz, CD_3CN, 25 °C, δ): 1.82 (q, *J* = 44.7 Hz).

HRMS ESIpos (m/z) calc'd for $C_9H_9NBNaF_3^+$ [M+Na]$^+$, 222.0672; found, 222.0668. Deviation: –1.8 ppm.

(1-(But-3-yn-1-yl)-5-chloropyridin-1-ium-3-yl)trifluoroborate (1k)

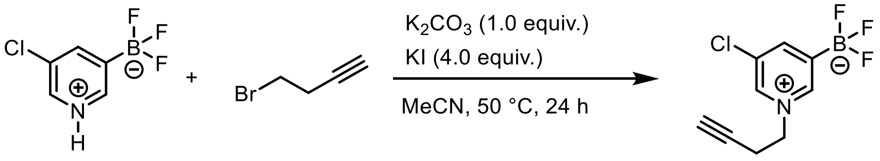

1k

Under an ambient atmosphere, (5-chloropyridin-1-ium-3-yl)trifluoroborate (36.2 mg, 0.200 mmol, 1.00 equiv.), K_2CO_3 (27.6 mg, 0.200 mmol, 1.00 equiv.), KI (220 mg, 0.800 mmol, 4.00 equiv.), and MeCN (1.00 mL, *c* = 0.2 M) were added to a 4-mL vial, followed by 4-bromobut-1-yne (53.2 mg, 0.400 mmol, 2.00 equiv.). After solvation, the reaction mixture was heated at 50 °C for 24 h. After that, the solvent was removed under reduced pressure. The residue was purified by chromatography on silica gel, eluting with dichloromethane/methanol (from 100:1 to 50:1, v:v) to afford 23.7 mg (51% yield) of the title compound as a pale yellow solid.

Melting Point = 202-203 °C.

R*f* = 0.60 (dichloromethane/methanol, 10:1, v/v (UV))

NMR Spectroscopy:

^{1}H NMR (400 MHz, CD_3CN, 25 °C, δ): 8.60 (s, 1H), 8.50 (d, *J* = 7.5 Hz, 1H), 8.45 (dt, *J* = 6.2, 1.6 Hz, 1H), 7.82 (t, *J* = 6.8 Hz, 1H), 4.56 (t, *J* = 6.5 Hz, 2H), 2.88 (td, *J* = 6.5, 2.7 Hz, 2H), 2.36 (t, *J* = 2.7 Hz, 1H).

^{13}C {^{1}H} NMR (101 MHz, DMSO-d_6, 25 °C, δ): 147.29, 145.21, 141.18, 132.86, 79.17, 75.00, 58.20, 20.30.The carbon directly attached to the boron atom was not detected due to quadrupolar broadening.

^{19}F {^{1}H} NMR (376 MHz, CD_3CN, 25 °C, δ): –144.22 (q, *J* = 43.2 Hz).

^{11}B NMR (128 MHz, CD_3CN, 25 °C, δ): 1.52 (q, *J* = 43.2 Hz).

HRMS ESIpos (m/z) calc'd for $C_9H_8NClBNaF_3^+$ [M+Na]$^+$, 256.0283; found, 256.0274. Deviation: –3.5 ppm.

(1-(But-3-yn-1-yl)pyridin-1-ium-4-yl)trifluoroborate (1l)

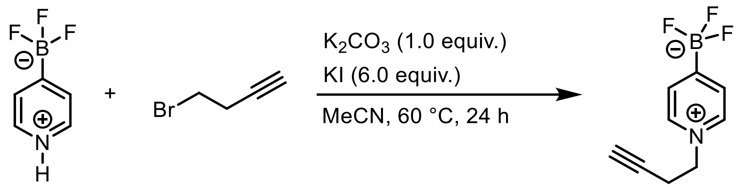

1l

Under an ambient atmosphere, (pyridin-1-ium-4-yl)trifluoroborate (294 mg, 2.00 mmol, 1.00 equiv.), K_2CO_3 (276 mg, 2.00 mmol, 1.00 equiv.), KI (2.00 g, 12.0 mmol, 6.00 equiv.), and MeCN (10.0 mL, *c* = 0.2 M) were added to a 20-mL vial, followed by 4-bromobut-1-yne (532 mg, 4.00 mmol, 2.00 equiv.). After solvation, the reaction mixture was heated at 60 °C for 24 h. After that, the solvent was removed under reduced pressure. The residue was purified by chromatography on silica gel, eluting with dichloromethane/methanol (from 100:1 to 50:1, v:v) to afford 315 mg (79% yield) of the title compound as a white solid.

Melting Point = 254-255 °C.

R_f = 0.65 (dichloromethane/methanol, 10:1, v/v (UV))

NMR Spectroscopy:

^{1}H NMR (400 MHz, CD_3CN, 25 °C, δ): 8.42 (d, *J* = 6.0 Hz, 2H), 8.01 (d, *J* = 5.9 Hz, 2H), 4.52 (t, *J* = 6.5 Hz, 2H), 2.85 (td, *J* = 6.5, 2.7 Hz, 2H), 2.36 (t, *J* = 2.7 Hz, 1H).

^{13}C {^{1}H} NMR (101 MHz, CD_3CN, 25 °C, δ): 142.36, 131.25 (d, *J* = 1.7 Hz), 79.35, 74.10, 59.43, 21.59.

The carbon directly attached to the boron atom was not detected due to quadrupolar broadening.

^{19}F {^{1}H} NMR (376 MHz, CD_3CN, 25 °C, δ): –145.94 (q, *J* = 44.7 Hz).

^{11}B NMR (128 MHz, CD_3CN, 25 °C, δ): 1.66 (q, *J* = 44.7 Hz).

HRMS ESIpos (m/z) calc'd for $C_9H_9NBNaF_3^+$ [M+Na]$^+$, 222.0672; found, 222.0671. Deviation: –0.5 ppm.

(1-(But-3-yn-1-yl)-5-fluoropyridin-1-ium-3-yl)trifluoroborate (1m)

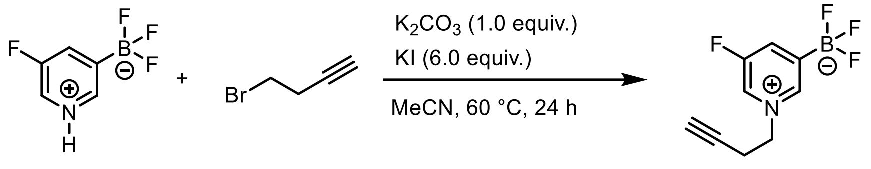

1m

Under an ambient atmosphere, (5-fluoropyridin-1-ium-3-yl)trifluoroborate (660 mg, 4.00 mmol, 1.00 equiv.), K_2CO_3 (552 mg, 4.00 mmol, 1.00 equiv.), KI (3.98 g, 24.0 mmol, 6.00 equiv.), and MeCN (20.0 mL, *c* = 0.2 M) were added to a 50-mL vial, followed by 4-bromobut-1-yne (1.06 g, 8.00 mmol, 2.00 equiv.). After solvation, the reaction mixture was heated at 60 °C for 24 h. After that, the solvent was removed under reduced pressure. The residue was purified by chromatography on silica gel, eluting with dichloromethane/methanol (from 100:1 to 50:1, v:v) to afford 529 mg (61% yield) of the title compound as a pale yellow solid.

Melting Point = 120-121 °C.

R_f = 0.60 (dichloromethane/methanol, 10:1, v/v (UV))

NMR Spectroscopy:

^{1}H NMR (400 MHz, DMSO-d_6, 25 °C, δ): 9.16 (ddd, *J* = 4.0, 2.6, 1.3 Hz, 1H), 8.74 (s, 1H), 8.29 (dd, *J* = 7.6, 2.6 Hz, 1H), 4.72 (t, *J* = 6.6 Hz, 2H), 3.05 (t, *J* = 2.6 Hz, 1H), 2.95 (td, *J* = 6.7, 2.7 Hz, 2H).

^{13}C {^{1}H} NMR (101 MHz, DMSO-d_6, 25 °C, δ): 159.44 (d, *J* = 252.7 Hz), 143.48, 134.67 (d, *J* = 13.9 Hz), 131.76 (d, *J* = 38.3 Hz), 79.13, 74.96, 58.33, 20.33. The carbon directly attached to the boron atom was not detected due to quadrupolar broadening.

^{19}F {^{1}H} NMR (376 MHz, CD_3CN, 25 °C, δ): –120.66 (1F), –144.22 (q, *J* = 43.1 Hz, 3F).

^{11}B NMR (128 MHz, CD_3CN, 25 °C, δ): 1.54 (q, *J* = 43.1 Hz).

HRMS ESIpos (m/z) calc'd for $C_9H_8NBNaF_4^+$ $[M+Na]^+$, 240.0578; found, 240.0582. Deviation: +1.7 ppm.

(1-(2-(2-(2-Azidoethoxy)ethoxy)ethyl)pyridin-1-ium-3-yl)trifluoroborate (1n)

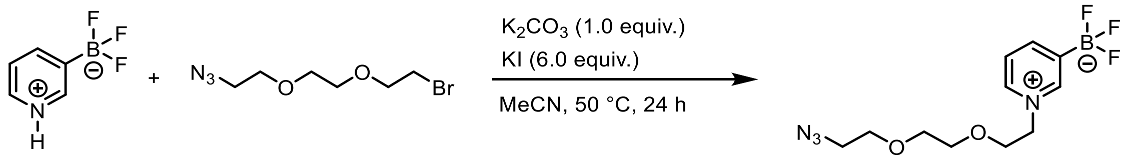

1n

Under an ambient atmosphere, (pyridin-1-ium-3-yl)trifluoroborate (147 mg, 1.00 mmol, 1.00 equiv.), K_2CO_3 (138 mg, 1.00 mmol, 1.00 equiv.), KI (1.65 g, 6.00 mmol, 6.00 equiv.), and MeCN (5.00 mL, *c* = 0.2 M) were added to a 20-mL vial, followed by 1-azido-2-(2-(2-bromoethoxy)ethoxy)ethane (357 mg, 1.50 mmol,

1.50 equiv.). After solvation, the reaction mixture was heated at 50 °C for 24 h. After that, the solvent was removed under reduced pressure. The residue was purified by chromatography on silica gel, eluting with dichloromethane/methanol (from 100:1 to 50:1, v:v) to afford 220 mg (72% yield) of the title compound as a pale yellow oil.

R*f* = 0.50 (dichloromethane/methanol, 10:1, v/v (UV))

NMR Spectroscopy:

^{1}H NMR (400 MHz, CD_3CN, 25 °C, δ): 8.56 (s, 1H), 8.50 – 8.43 (m, 2H), 7.80 (t, *J* = 6.8 Hz, 1H), 4.59 (t, *J* = 4.8 Hz, 2H), 3.90 (t, *J* = 4.9 Hz, 2H), 3.64 – 3.44 (m, 6H), 3.31 (t, *J* = 5.0 Hz, 2H).

^{13}C {^{1}H} NMR (101 MHz, CD_3CN, 25 °C, δ): 149.46, 147.11, 142.82, 127.33, 71.32, 70.86, 70.56, 70.04, 61.62, 51.36. The carbon directly attached to the boron atom was not detected due to quadrupolar broadening.

^{19}F {^{1}H} NMR (376 MHz, CD_3CN, 25 °C, δ): –143.67 (q, *J* = 43.7 Hz).

^{11}B NMR (128 MHz, CD_3CN, 25 °C, δ): 1.92 (q, *J* = 43.7 Hz).

HRMS ESIpos (m/z) calc'd for $C_{11}H_{16}BNaN_4O_2F_3^+$ [M+Na]$^+$, 327.1211; found, 327.1206. Deviation: –1.5 ppm.

(6-Methyl-1-(prop-2-yn-1-yl)pyridin-1-ium-2-yl)trifluoroborate (1o)

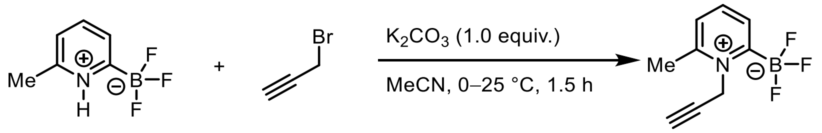

Under an ambient atmosphere, (6-methylpyridin-1-ium-2-yl)trifluoroborate (80.5 mg, 0.500 mmol, 1.00 equiv.), K_2CO_3 (69.0 mg, 0.500 mmol, 1.00 equiv.), and MeCN (2.5 mL, *c* = 0.2 M) were added to a 4-mL vial, followed by 3-bromoprop-1-yne (119 mg, 1.00 mmol, 2.00 equiv.) dropwise using syringe at 0 °C. After stirring for 30 min, the mixture was allowed to stir at room temperature and monitored by TLC every 10 minutes until the formation of side products was observed. Then the mixture was added TFA (114 mg, 1.00 mmol, 2.00 equiv.) to quench the reaction. After solvation, the solvent was removed under reduced pressure. The residue was purified by chromatography on silica gel, eluting with dichloromethane/methanol (from 100:1 to 50:1, v:v) to afford 93.3 mg (94% yield) of the title compound as a colorless solid.

Melting Point = 138-139 °C.

R*f* = 0.80 (dichloromethane/methanol, 10:1, v/v (UV))

NMR Spectroscopy:

^{1}H NMR (400 MHz, CD_3CN, 25 °C, δ): 8.13 (t, *J* = 7.8 Hz, 1H), 7.95 (d, *J* = 6.9 Hz, 1H), 7.64 (d, *J* = 7.7 Hz, 1H), 5.45 (d, *J* = 2.8 Hz, 2H), 2.89 (s, 3H), 2.87 (t, *J* = 2.5 Hz, 1H).

^{13}C {^{1}H} NMR (101 MHz, CD_3CN, 25 °C, δ): 155.99, 144.28, 130.53 (q, J = 3.0 Hz), 128.94, 77.17, 77.08, 46.17 (q, J = 4.0 Hz), 21.04. The carbon directly attached to the boron atom was not detected due to quadrupolar broadening.

^{19}F {^{1}H} NMR (376 MHz, CD_3CN, 25 °C, δ): –141.89 – –142.31 (m).

^{11}B NMR (128 MHz, CD_3CN, 25 °C, δ): 0.74 (q, J = 40.9 Hz).

HRMS ESIpos (m/z) calc'd for $C_9H_9NBNaF_3^+$ [M+Na]$^+$, 222.0672; found, 222.0673. Deviation: +0.5 ppm.

(4-Fluoro-1-(prop-2-yn-1-yl)pyridin-1-ium-2-yl)trifluoroborate (1p)

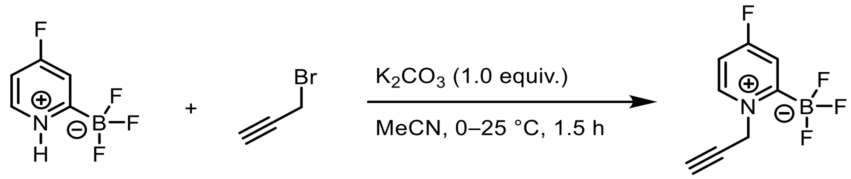

1p

Under an ambient atmosphere, (4-fluoropyridin-1-ium-2-yl)trifluoroborate (82.5 mg, 0.500 mmol, 1.00 equiv.), K_2CO_3 (69.0 mg, 0.500 mmol, 1.00 equiv.), and MeCN (2.5 mL, c = 0.2 M) were added to a 4-mL vial, followed by 3-bromoprop-1-yne (119 mg, 1.00 mmol, 2.00 equiv.) dropwise using syringe at 0 °C. After stirring for 30 min, the mixture was allowed to stir at room temperature and monitored by TLC every 10 minutes until the formation of side products was observed. Then the mixture was added TFA (114 mg, 1.00 mmol, 2.00 equiv.) to quench the reaction. After solvation, the solvent was removed under reduced pressure. The residue was purified by chromatography on silica gel, eluting with dichloromethane/methanol (from 100:1 to 50:1, v:v) to afford 92.4 mg (91% yield) of the title compound as a pale yellow solid.

Melting Point = 94-95 °C.

R_f = 0.80 (dichloromethane/methanol, 10:1, v/v (UV))

NMR Spectroscopy:

^{1}H NMR (400 MHz, CD_3CN, 25 °C, δ): 8.90 (dd, J = 7.1, 5.0 Hz, 1H), 7.83 – 7.77 (m, 1H), 7.57 (td, J = 7.0, 3.3 Hz, 1H), 5.47 (d, J = 2.6 Hz, 2H), 3.07 (t, J = 2.6 Hz, 1H).

^{13}C {^{1}H} NMR (101 MHz, CD_3CN, 25 °C, δ): 172.37 (d, J = 278.6 Hz), 149.03 (d, J = 12.1 Hz), 119.65 (dq, J = 18.5, 2.9 Hz), 115.69 (d, J = 23.3 Hz), 79.45, 76.74, 48.90 (q, J = 4.0 Hz). The carbon directly attached to the boron atom was not detected due to quadrupolar broadening.

^{19}F {^{1}H} NMR (376 MHz, CD_3CN, 25 °C, δ): –88.69 (1F), –143.97 (q, J = 39.0 Hz, 3F).

^{11}B NMR (128 MHz, CD_3CN, 25 °C, δ): 0.30 (q, J = 39.0 Hz).

HRMS ESIpos (m/z) calc'd for $C_8H_6NBNaF_4^+$ [M+Na]$^+$, 226.0422; found, 226.0426. Deviation: +1.8 ppm.

(4-(*Tert*-butyl)-1-(prop-2-yn-1-yl)pyridin-1-ium-2-yl)trifluoroborate (1q)

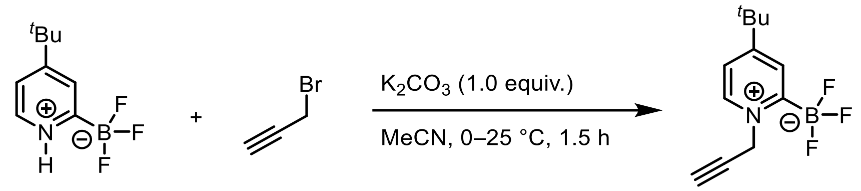

1q

Under an ambient atmosphere, (4-(*tert*-butyl)pyridin-1-ium-2-yl)trifluoroborate (101.5 mg, 0.500 mmol, 1.00 equiv.), K_2CO_3 (69.0 mg, 0.500 mmol, 1.00 equiv.), and MeCN (2.5 mL, *c* = 0.2 M) were added to a 4-mL vial, followed by 3-bromoprop-1-yne (119 mg, 1.00 mmol, 2.00 equiv.) dropwise using syringe at 0 °C. After stirring for 30 min, the mixture was allowed to stir at room temperature and monitored by TLC every 10 minutes until the formation of side products was observed. Then the mixture was added TFA (114 mg, 1.00 mmol, 2.00 equiv.) to quench the reaction. After solvation, the solvent was removed under reduced pressure. The residue was purified by chromatography on silica gel, eluting with dichloromethane/methanol (from 100:1 to 50:1, v:v) to afford 110.9 mg (92% yield) of the title compound as a colorless solid.

Melting Point = 136-137 °C.

R_f = 0.70 (dichloromethane/methanol, 10:1, v/v (UV))

NMR Spectroscopy:

^{1}H NMR (400 MHz, CD_3CN, 25 °C, δ): 8.68 (d, *J* = 6.7 Hz, 1H), 8.06 (s, 1H), 7.81 (dd, *J* = 6.7, 2.5 Hz, 1H), 5.45 (d, *J* = 2.6 Hz, 2H), 3.01 (t, *J* = 2.6 Hz, 1H), 1.36 (s, 9H).

^{13}C {^{1}H} NMR (101 MHz, CD_3CN, 25 °C, δ): 169.21, 143.67, 128.88 (q, *J* = 2.8 Hz), 124.18, 78.57, 77.20, 48.33 (q, *J* = 3.9 Hz), 36.62, 30.06. The carbon directly attached to the boron atom was not detected due to quadrupolar broadening.

^{19}F {^{1}H} NMR (376 MHz, CD_3CN, 25 °C, δ): –142.85 (q, *J* = 40.9 Hz).

^{11}B NMR (128 MHz, CD_3CN, 25 °C, δ): 0.66 (q, *J* = 40.9 Hz).

HRMS ESIpos (m/z) calc'd for $C_{12}H_{15}NBNaF_3^+$ $[M+Na]^+$, 264.1142; found, 264.1141. Deviation: –0.4 ppm.

(4-Methyl-1-(prop-2-yn-1-yl)pyridin-1-ium-2-yl)trifluoroborate (1r)

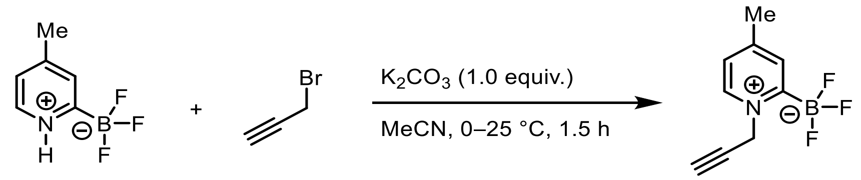

1r

Under an ambient atmosphere, (4-methylpyridin-1-ium-2-yl)trifluoroborate (80.5 mg, 0.500 mmol, 1.00 equiv.), K_2CO_3 (69.0 mg, 0.500 mmol, 1.00 equiv.), and MeCN (2.5 mL, *c* = 0.2 M) were added to a 4-mL vial, followed by 3-bromoprop-1-yne (119 mg, 1.00 mmol, 2.00 equiv.) dropwise using syringe at 0 °C. After stirring for 30 min, the mixture was allowed to stir at room temperature and monitored by TLC every 10 minutes until the formation of side products was observed. Then the mixture was added TFA (114 mg, 1.00 mmol, 2.00 equiv.) to quench the reaction. After solvation, the solvent was removed under reduced pressure. The residue was purified by chromatography on silica gel, eluting with dichloromethane/methanol (from 100:1 to 50:1, v:v) to afford 89.9 mg (90% yield) of the title compound as a colorless solid.

Melting Point = 132-133 °C.

R_f = 0.70 (dichloromethane/methanol, 10:1, v/v (UV))

NMR Spectroscopy:

^{1}H NMR (400 MHz, CD_3CN, 25 °C, δ): 8.62 (d, *J* = 6.5 Hz, 1H), 7.89 (s, 1H), 7.60 (dd, *J* = 6.6, 2.3 Hz, 1H), 5.43 (d, *J* = 2.6 Hz, 2H), 3.01 (t, *J* = 2.6 Hz, 1H), 2.53 (s, 3H).

^{13}C {^{1}H} NMR (101 MHz, CD_3CN, 25 °C, δ): 157.92, 143.34, 132.82 (q, *J* = 2.9 Hz), 127.34, 78.54, 77.14, 48.28 (q, *J* = 3.9 Hz), 21.79. The carbon directly attached to the boron atom was not detected due to quadrupolar broadening.

^{19}F {^{1}H} NMR (376 MHz, CD_3CN, 25 °C, δ): –142.84 (dd, *J* = 40.9 Hz).

^{11}B NMR (128 MHz, CD_3CN, 25 °C, δ): 0.60 (q, *J* = 40.9 Hz).

HRMS ESIpos (m/z) calc'd for $C_9H_9NBNaF_3^+$ $[M+Na]^+$, 222.0672; found, 222.0673. Deviation: +0.5 ppm.

(4-Chloro-1-(prop-2-yn-1-yl)pyridin-1-ium-2-yl)trifluoroborate (1s)

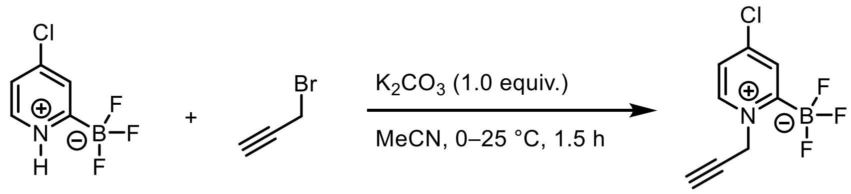

Under an ambient atmosphere, (4-chloropyridin-1-ium-2-yl)trifluoroborate (90.5 mg, 0.500 mmol, 1.00 equiv.), K_2CO_3 (69.0 mg, 0.500 mmol, 1.00 equiv.), and MeCN (2.5 mL, *c* = 0.2 M) were added to a 4-mL vial, followed by 3-bromoprop-1-yne (119 mg, 1.00 mmol, 2.00 equiv.) dropwise using syringe at 0 °C. After stirring for 30 min, the mixture was allowed to stir at room temperature and monitored by TLC every 10 minutes until the formation of side products was observed. Then the mixture was added TFA (114 mg, 1.00 mmol, 2.00 equiv.) to quench the reaction. After solvation, the solvent was removed under reduced pressure. The residue was purified by chromatography on silica gel, eluting with dichloromethane/methanol (from 100:1 to 50:1, v:v) to afford 77.4 mg (71% yield) of the title compound as a white solid.

Melting Point = 153-154 °C.

R*f* = 0.80 (dichloromethane/methanol, 10:1, v/v (UV))

NMR Spectroscopy:

^{1}H NMR (400 MHz, CD_3CN, 25 °C, δ): 8.77 (d, *J* = 6.8 Hz, 1H), 8.07 (s, 1H), 7.83 (dd, *J* = 6.8, 2.6 Hz, 1H), 5.47 (d, *J* = 2.6 Hz, 2H), 3.07 (t, *J* = 2.6 Hz, 1H).

^{13}C {^{1}H} NMR (101 MHz, CD_3CN, 25 °C, δ): 153.05, 145.63, 132.51 (q, *J* = 3.0 Hz), 127.31, 79.46, 76.47, 49.00 (q, *J* = 4.3 Hz). The carbon directly attached to the boron atom was not detected due to quadrupolar broadening.

^{19}F {^{1}H} NMR (376 MHz, CD_3CN, 25 °C, δ): –143.53 (q, *J* = 39.2 Hz).

^{11}B NMR (128 MHz, CD_3CN, 25 °C, δ): 0.31 (q, *J* = 39.2 Hz).

HRMS ESIpos (m/z) calc'd for $C_8H_6NBNaClF_3^+$ [M+Na]$^+$, 242.0126; found, 242.0127. Deviation: +0.4 ppm.

(4-Methoxy-1-(prop-2-yn-1-yl)pyridin-1-ium-2-yl)trifluoroborate (1t)

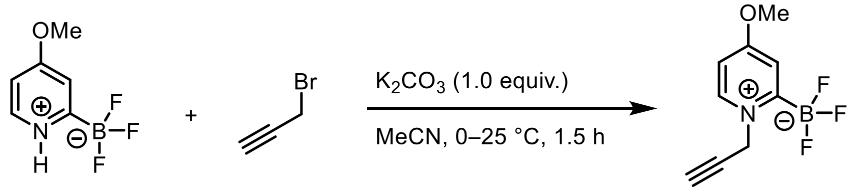

Under an ambient atmosphere, (4-methoxypyridin-1-ium-2-yl)trifluoroborate (88.5 mg, 0.500 mmol, 1.00 equiv.), K_2CO_3 (69.0 mg, 0.500 mmol, 1.00 equiv.), and MeCN (2.5 mL, *c* = 0.2 M) were added to a 4-mL vial, followed by 3-bromoprop-1-yne (119 mg, 1.00 mmol, 2.00 equiv.) dropwise using syringe at 0 °C. After stirring for 30 min, the mixture was allowed to stir at room temperature and monitored by TLC every 10 minutes until the formation of side products was observed. Then the mixture was added TFA (114 mg, 1.00 mmol, 2.00 equiv.) to quench the reaction. After solvation, the solvent was removed under reduced pressure. The residue was purified by chromatography on silica gel, eluting with dichloromethane/methanol (from 100:1 to 50:1, v:v) to afford 92.0 mg (86% yield) of the title compound as a white solid.

Melting Point = 140-141 °C.

R*f* = 0.80 (dichloromethane/methanol, 10:1, v/v (UV))

NMR Spectroscopy:

^{1}H NMR (400 MHz, CD_3CN, 25 °C, δ): 8.55 (d, *J* = 7.3 Hz, 1H), 7.43 (s, 1H), 7.20 (dd, *J* = 7.3, 3.2 Hz, 1H), 5.32 (d, *J* = 2.6 Hz, 2H), 4.03 (s, 3H), 2.97 (t, *J* = 2.6 Hz, 1H).

^{13}C {^{1}H} NMR (101 MHz, CD_3CN, 25 °C, δ): 170.70, 145.85, 115.73 (q, *J* = 3.0 Hz), 113.42, 78.07, 77.51, 57.95, 47.36 (q, *J* = 4.0 Hz). The carbon directly attached to the boron atom was not detected due to quadrupolar broadening.

^{19}F {^{1}H} NMR (376 MHz, CD_3CN, 25 °C, δ): –143.44 (q, *J* = 40.7 Hz).

^{11}B NMR (128 MHz, CD_3CN, 25 °C, δ): 0.52 (q, *J* = 40.7 Hz).

HRMS ESIpos (m/z) calc'd for $C_9H_9NBNaOF_3^+$ $[M+Na]^+$, 238.0622; found, 238.0624. Deviation: +0.8 ppm.

Preparation of PyH-BF_3 reagents

(Pyridin-1-ium-3-yl)trifluoroborate (1b-S)

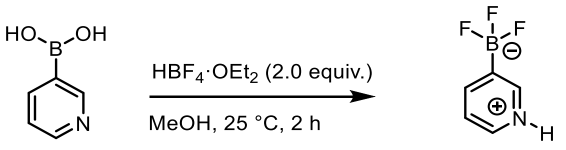

1b-S

Under an ambient atmosphere, pyridin-3-ylboronic acid (615 mg, 5.00 mmol, 1.00 equiv.), and MeOH (25 mL, *c* = 0.2 M) were added to a 100-mL round-bottom flask, followed by $HBF_4{\cdot}OEt_2$ (1.37 mL, 1.62 g, 10.0 mmol, 2.00 equiv.). After solvation, the solvent was removed by rotary evaporation. The residue was purified by chromatography on silica gel, eluting with dichloromethane/methanol (from 100:1 to 50:1, v:v) to afford 673 mg (91% yield) of the title compound as a white solid. The spectroscopic data are consistent with those recently reported[15].

Melting Point = 129-130 °C.

R_f = 0.20 (dichloromethane/methanol, 10:1, v/v (UV))

NMR Spectroscopy:

^{1}H NMR (400 MHz, CD_3CN, 25 °C, δ): 12.88 (brs, 1H), 8.58 (d, *J* = 7.6 Hz, 1H), 8.54 (s, 1H), 8.43 (d, *J* = 5.8 Hz, 1H), 7.83 (t, *J* = 6.7 Hz, 1H).

^{13}C {^{1}H} NMR (101 MHz, CD_3CN, 25 °C, δ): 151.20, 143.33, 139.12, 126.98. The carbon directly attached to the boron atom was not detected due to quadrupolar broadening.

^{19}F {^{1}H} NMR (376 MHz, CD_3CN, 25 °C, δ): –144.12 (q, *J* = 45.3 Hz).

^{11}B NMR (128 MHz, CD_3CN, 25 °C, δ): 1.95 (q, *J* = 45.3 Hz).

HRMS ESIpos (m/z) calc'd for $C_5H_5NBNaF_3^+$ $[M+Na]^+$, 170.0359; found, 170.0357. Deviation: –1.2 ppm.

Discussion: During the preparation of this manuscript, another method for the synthesis of compound **1b-S** was reported. However, this protocol requires the direct use of excess hazardous **HF** to achieve efficient conversion. In contrast, our protocol only requires a stoichiometric amount of $HBF_4 \cdot OEt_2$, providing a milder

and greener alternative.

(Pyridin-1-ium-4-yl)trifluoroborate (1c-S)

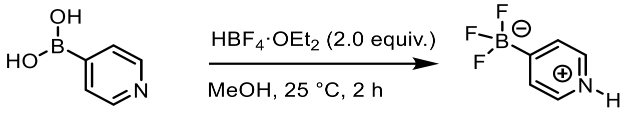

1c-S

Under an ambient atmosphere, pyridin-4-ylboronic acid (123 mg, 1.00 mmol, 1.00 equiv.), and MeOH (5 mL, *c* = 0.2 M) were added to a 20-mL vial, followed by $HBF_4{\cdot}OEt_2$ (274 µL, 324 mg, 2.00 mmol, 2.00 equiv.). After solvation, the solvent was removed by rotary evaporation. The residue was purified by chromatography on silica gel, eluting with dichloromethane/methanol (from 100:1 to 50:1, v:v) to afford 90.1 mg (70% yield) of the title compound as a white solid.

Melting Point = 279-280 °C.

$\mathbf{R}_f$ = 0.20 (dichloromethane/methanol, 10:1, v/v (UV))

NMR Spectroscopy:

^{1}H NMR (400 MHz, CD_3CN, 25 °C, δ): 12.59 (t, *J* = 67.1 Hz, 1H), 8.39 (t, *J* = 6.3 Hz, 2H), 8.03 (s, 2H).

^{13}C {^{1}H} NMR (101 MHz, CD_3CN, 25 °C, δ): 139.06 (2C), 130.59 (2C). The carbon directly attached to the boron atom was not detected due to quadrupolar broadening.

^{19}F {^{1}H} NMR (376 MHz, CD_3CN, 25 °C, δ): –146.00 (q, *J* = 45.4 Hz).

^{11}B NMR (128 MHz, CD_3CN, 25 °C, δ): 1.71 (q, *J* = 45.4 Hz).

HRMS ESIpos (m/z) calc'd for $C_5H_5NBNaF_3^+$ $[M+Na]^+$, 170.0359; found, 170.0355. Deviation: –2.4 ppm.

(6-Methylpyridin-1-ium-3-yl)trifluoroborate (1d-S)

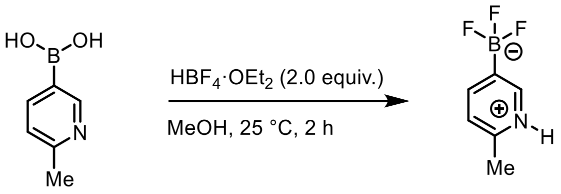

1d-S

Under an ambient atmosphere, (6-methylpyridin-3-yl)boronic acid (1.37 g, 10.0 mmol, 1.00 equiv.), and MeOH (50 mL, *c* = 0.2 M) were added to a 100-mL round-bottom flask, followed by $HBF_4{\cdot}OEt_2$ (2.74 mL, 3.24 g, 20.0 mmol, 2.00 equiv.). After solvation, the solvent was removed by rotary evaporation. The residue was dissolved in ca. 5 mL MeOH, and precipitated with ca. 95 mL Et_2O. The suspension was decanted, and the solid was dried *in vacuo* to afford 769 mg (48% yield) of the title compound as a white solid.

Melting Point = 172-173 °C.

R*f* = 0.50 (dichloromethane/methanol, 10:1, v/v (UV))

NMR Spectroscopy:

^{1}H NMR (400 MHz, CD_3CN, 25 °C, δ): 12.60 (t, *J* = 64.7 Hz, 1H), 8.42 (d, *J* = 7.9 Hz, 1H), 8.35 (d, *J* = 6.4 Hz, 1H), 7.63 (d, *J* = 7.8 Hz, 1H), 2.64 (s, 3H).

^{13}C {^{1}H} NMR (101 MHz, CD_3CN, 25 °C, δ): 151.23, 151.08, 142.42, 127.50, 19.57. The carbon directly attached to the boron atom was not detected due to quadrupolar broadening.

^{19}F {^{1}H} NMR (376 MHz, CD_3CN, 25 °C, δ): –143.69 (q, *J* = 45.8 Hz).

^{11}B NMR (128 MHz, CD_3CN, 25 °C, δ): 2.04 (q, *J* = 45.8 Hz).

HRMS ESIpos (m/z) calc'd for $C_6H_7NBNaF_3^+$ [M+Na]$^+$, 184.0516; found, 184.0516. Deviation: 0.0 ppm.

(Quinolin-1-ium-3-yl)trifluoroborate (1e-S)

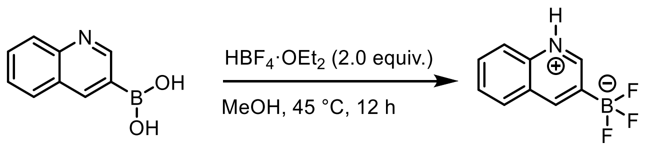

1e-S

Under an ambient atmosphere, quinolin-3-ylboronic acid (1.73 g, 10.0 mmol, 1.00 equiv.), and MeOH (50 mL, *c* = 0.2 M) were added to a 100-mL round-bottom flask, followed by $HBF_4·OEt_2$ (2.74 mL, 3.24 g, 20.0 mmol, 2.00 equiv.). After solvation, the solvent was removed by rotary evaporation. The residue was dissolved in ca. 5 mL MeOH, and precipitated with ca. 95 mL Et_2O. The suspension was decanted, and the solid was dried *in vacuo* to afford 1.78 g (90% yield) of the title compound as a white solid.

Melting Point = 233-234 °C.

R*f* = 0.40 (dichloromethane/methanol, 10:1, v/v (UV))

NMR Spectroscopy:

^{1}H NMR (400 MHz, CD_3CN, 25 °C, δ): 13.37 (t, *J* = 61.8 Hz, 1H), 9.10 (s, 1H), 8.93 (d, *J* = 7.2 Hz, 1H), 8.21 (d, *J* = 8.4 Hz, 1H), 8.11 (d, *J* = 8.6 Hz, 1H), 8.04 – 7.98 (m, 1H), 7.88 – 7.83 (m, 1H).

^{13}C {^{1}H} NMR (101 MHz, DMSO-*d*$_6$, 25 °C, δ): 148.87 (q, *J* = 2.2 Hz), 147.24, 136.27, 133.22, 128.89 (q, *J* = 3.4 Hz), 128.50, 120.14. The carbon directly attached to the boron atom was not detected due to quadrupolar broadening.

^{19}F {^{1}H} NMR (376 MHz, DMSO-*d*$_6$, 25 °C, δ): –139.65 (brs).

^{11}B NMR (128 MHz, DMSO-*d*$_6$, 25 °C, δ): 2.27 (brs).

HRMS ESIpos (m/z) calc'd for $C_9H_7NBNaF_3^+$ [M+Na]$^+$, 220.0516; found, 220.0517. Deviation: +0.5 ppm.

(5-Methoxypyridin-1-ium-3-yl)trifluoroborate (1f-S)

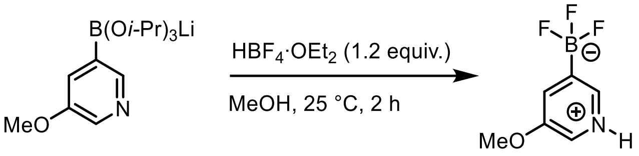

1f-S

Under an ambient atmosphere, triisopropyl(5-methylpyridin-3-yl)lithium borate (1.51 g, 5.00 mmol, 1.00 equiv.), and MeOH (12.5 mL, *c* = 0.2 M) were added to a 100-mL round-bottom flask, followed by $HBF_4 \cdot OEt_2$ (822 µL, 972 mg, 6.00 mmol, 1.20 equiv.). After solvation, the solvent was removed by rotary evaporation. The residue was dissolved in ca. 2.5 mL MeOH, and precipitated with ca. 50 mL Et_2O. The suspension was decanted, and the solid was dried *in vacuo* to afford 571 mg (68% yield) of the title compound as a pale yellow solid.

Melting Point = 195-196 °C.

$\mathbf{R}_f$ = 0.50 (dichloromethane/methanol, 10:1, v/v (UV))

NMR Spectroscopy:

^{1}**H NMR** (400 MHz, CD_3CN, 25 °C, δ): 12.67 (t, *J* = 65.9 Hz, 1H), 8.18 (d, *J* = 6.3 Hz, 1H), 8.12 – 8.04 (m, 2H), 3.95 (s, 3H).

^{13}**C {^{1}H} NMR** (101 MHz, DMSO-d_6, 25 °C, δ): 156.73, 136.39 (q, *J* = 2.1 Hz), 132.45 (q, *J* = 2.0 Hz), 126.50, 56.44. The carbon directly attached to the boron atom was not detected due to quadrupolar broadening.

^{19}**F {^{1}H} NMR** (376 MHz, DMSO-d_6, 25 °C, δ): –140.37 (brs).

^{11}**B NMR** (128 MHz, DMSO-d_6, 25 °C, δ): 1.78 (brs).

HRMS ESIpos (m/z) calc'd for $C_6H_7NBNaOF_3^+$ [M+Na]$^+$, 200.0465; found, 200.0465. Deviation: 0.0 ppm.

(3-Fluoropyridin-1-ium-4-yl)trifluoroborate (1g-S)

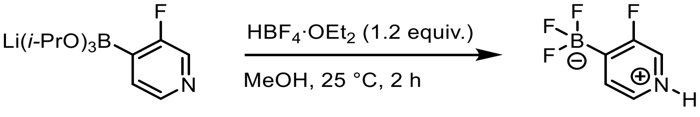

1g-S

Under an ambient atmosphere, triisopropyl(3-fluoropyridin-4-yl)lithium borate (2.91 g, 10.0 mmol, 1.00 equiv.), and MeOH (25.0 mL, *c* = 0.2 M) were added to a 100-mL round-bottom flask, followed by $HBF_4 \cdot OEt_2$ (1.64 mL, 1.94 g, 10.0 mmol, 1.20 equiv.). After solvation, the solvent was removed by rotary evaporation. The residue was dissolved in ca. 5 mL MeOH, and precipitated with ca. 95 mL Et_2O. The

suspension was decanted, and the solid was dried *in vacuo* to afford 1.52 g (92% yield) of the title compound as a pale yellow solid.

Melting Point = 225-226 °C.

R_f = 0.50 (dichloromethane/methanol, 3:1, v/v (UV))

NMR Spectroscopy:

^{1}H NMR (400 MHz, CD_3CN, 25 °C, δ): 8.37 (s, 1H), 8.33 (d, *J* = 5.5 Hz, 1H), 8.07 (s, 1H). The proton directly attached to the nitrogen atom was not detected due to fast exchange.

^{13}C {^{1}H} NMR (101 MHz, DMSO-*d*$_6$, 25 °C, δ): 163.17 (d, *J* = 246.3 Hz), 137.75, 131.59 (d, *J* = 12.3 Hz), 130.10 (d, *J* = 39.7 Hz). The carbon directly attached to the boron atom was not detected due to quadrupolar broadening.

^{19}F {^{1}H} NMR (376 MHz, CD_3CN, 25 °C, δ): –113.81 (1F), –142.79 (q, *J* = 39.2 Hz, 3F).

^{11}B NMR (128 MHz, DMSO-*d*$_6$, 25 °C, δ): 1.50 (brs).

HRMS ESIpos (m/z) calc'd for $C_5H_4NBNaF_4^+$ $[M+Na]^+$, 188.0265; found, 188.0269. Deviation: +2.1 ppm.

(3-Chloropyridin-1-ium-4-yl)trifluoroborate (1h-S)

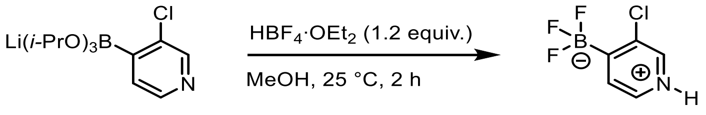

Under an ambient atmosphere, triisopropyl(3-chloropyridin-4-yl)lithium borate (3.07 g, 10.0 mmol, 1.00 equiv.), and MeOH (25.0 mL, *c* = 0.2 M) were added to a 100-mL round-bottom flask, followed by HBF_4·OEt_2 (1.64 mL, 1.94 g, 10.0 mmol, 1.20 equiv.). After solvation, the solvent was removed by rotary evaporation. The residue was dissolved in ca. 5 mL MeOH, and precipitated with ca. 95 mL Et_2O. The suspension was decanted, and the solid was dried *in vacuo* to afford 1.55 g (86% yield) of the title compound as a pale yellow solid.

Melting Point = 203-204 °C.

R_f = 0.50 (dichloromethane/methanol, 3:1, v/v (UV))

NMR Spectroscopy:

^{1}H NMR (400 MHz, CD_3CN, 25 °C, δ): 8.50 (s, 1H), 8.40 (d, *J* = 5.6 Hz, 1H), 8.08 (d, *J* = 5.6 Hz, 1H). The proton directly attached to the nitrogen atom was not detected due to fast exchange.

^{13}C {^{1}H} NMR (101 MHz, DMSO-*d*$_6$, 25 °C, δ): 139.69, 138.19, 137.33, 130.52 (q, *J* = 2.7 Hz). The carbon directly attached to the boron atom was not detected due to quadrupolar broadening.

^{19}F {^{1}H} NMR (376 MHz, DMSO-*d*$_6$, 25 °C, δ): –141.54 (brs).

^{11}B NMR (128 MHz, DMSO-*d*$_6$, 25 °C, δ): 1.31 (q, *J* = 44.8 Hz).

HRMS ESIpos (m/z) calc'd for $C_5H_4NClBNaF_3^+$ $[M+Na]^+$, 203.9970; found, 203.9971. Deviation: +0.5 ppm.

(2-Methylpyridin-1-ium-4-yl)trifluoroborate (1i-S)

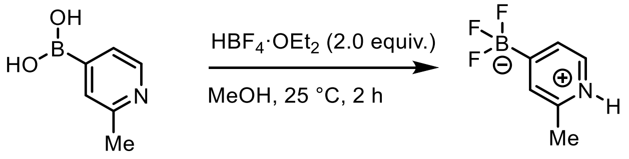

1i-S

Under an ambient atmosphere, (2-methylpyridin-4-yl)boronic acid (1.37 g, 10.0 mmol, 1.00 equiv.), and MeOH (50 mL, *c* = 0.2 M) were added to a 100-mL round-bottom flask, followed by $HBF_4{\cdot}OEt_2$ (2.74 mL, 3.24 g, 20.0 mmol, 2.00 equiv.). After solvation, the solvent was removed by rotary evaporation. The residue was dissolved in ca. 5 mL MeOH, and precipitated with ca. 95 mL Et_2O. The suspension was decanted, and the solid was dried *in vacuo* to afford 1.32 g (82% yield) of the title compound as a white solid.

Melting Point = 211-212 °C.

R*f* = 0.50 (dichloromethane/methanol, 10:1, v/v (UV))

NMR Spectroscopy:

^{1}H NMR (400 MHz, CD_3CN, 25 °C, δ): 12.47 (t, *J* = 65.4 Hz, 1H), 8.21 (t, *J* = 6.1 Hz, 1H), 7.83 (s, 1H), 7.80 (d, *J* = 5.1 Hz, 1H), 2.65 (s, 3H).

^{13}C {^{1}H} NMR (101 MHz, DMSO-*d*$_6$, 25 °C, δ): 149.94, 137.83, 129.69 (q, *J* = 1.8 Hz), 126.46 (q, *J* = 1.8 Hz), 18.92. The carbon directly attached to the boron atom was not detected due to quadrupolar broadening.

^{19}F {^{1}H} NMR (376 MHz, CD_3CN, 25 °C, δ): –145.71 (q, *J* = 46.0 Hz).

^{11}B NMR (128 MHz, CD_3CN, 25 °C, δ): 1.74 (q, *J* = 46.0 Hz).

HRMS ESIpos (m/z) calc'd for $C_6H_7NBNaF_3^+$ $[M+Na]^+$, 184.0516; found, 184.0516. Deviation: 0.0 ppm.

(5-Chloropyridin-1-ium-3-yl)trifluoroborate (1k-S)

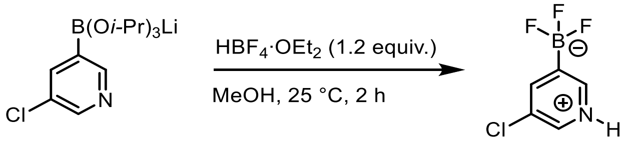

1k-S

Under an ambient atmosphere, triisopropyl(5-chloropyridin-3-yl)lithium borate (3.07 g, 10.0 mmol, 1.00 equiv.), and MeOH (25.0 mL, *c* = 0.2 M) were added to a 100-mL round-bottom flask, followed by

$HBF_4·OEt_2$ (1.64 mL, 1.94 g, 10.0 mmol, 1.20 equiv.). After solvation, the solvent was removed by rotary evaporation. The residue was dissolved in ca. 5 mL MeOH, and precipitated with ca. 95 mL Et_2O. The suspension was decanted, and the solid was dried *in vacuo* to afford 1.68 g (92% yield) of the title compound as a pale yellow solid.

Melting Point = 215-216 °C.

R*f* = 0.20 (dichloromethane/methanol, 10:1, v/v (UV))

NMR Spectroscopy:

^{1}**H NMR** (400 MHz, DMSO-*d*$_6$, 25 °C, δ): 8.58 (d, *J* = 2.5 Hz, 1H), 8.44 (s, 1H), 7.93 (d, *J* = 2.0 Hz, 1H). The proton directly attached to the nitrogen atom was not detected due to fast exchange.

^{13}**C {^{1}H} NMR** (101 MHz, CD_3CN, 25 °C, δ): 150.59, 142.43, 138.44, 134.93. The carbon directly attached to the boron atom was not detected due to quadrupolar broadening.

^{19}**F {^{1}H} NMR** (376 MHz, CD_3CN, 25 °C, δ): –144.38 (q, *J* = 43.6 Hz).

^{11}**B NMR** (128 MHz, CD_3CN, 25 °C, δ): 1.58 (q, *J* = 43.6 Hz).

HRMS ESIpos (m/z) calc'd for $C_5H_4NClBNaF_3^+$ $[M+Na]^+$, 203.9970; found, 203.9972. Deviation: +1.0 ppm.

(5-Fluoropyridin-1-ium-3-yl)trifluoroborate (1m-S)

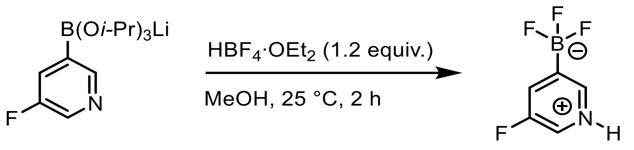

1m-S

Under an ambient atmosphere, triisopropyl(5-fluoropyridin-3-yl)lithium borate (2.91 g, 10.0 mmol, 1.00 equiv.), and MeOH (25.0 mL, *c* = 0.2 M) were added to a 100-mL round-bottom flask, followed by $HBF_4·OEt_2$ (1.64 mL, 1.94 g, 10.0 mmol, 1.20 equiv.). After solvation, the solvent was removed by rotary evaporation. The residue was dissolved in ca. 5 mL MeOH, and precipitated with ca. 95 mL Et_2O. The suspension was decanted, and the solid was dried *in vacuo* to afford 1.56 g (94% yield) of the title compound as a pale yellow solid.

Melting Point = 220-221 °C.

R*f* = 0.20 (dichloromethane/methanol, 10:1, v/v (UV))

NMR Spectroscopy:

^{1}**H NMR** (400 MHz, CD_3CN, 25 °C, δ): 12.70 (brs, 1H), 8.46 (s, 1H), 8.43 (t, *J* = 2.8 Hz, 1H), 8.37 (d, *J* = 7.8 Hz, 1H).

^{13}C {^{1}H} NMR (101 MHz, DMSO-d_6, 25 °C, δ): 159.58 (d, J = 252.4 Hz), 144.09, 131.20 (d, J = 29.7 Hz), 130.23 (dd, J = 13.6, 2.0 Hz).The carbon directly attached to the boron atom was not detected due to quadrupolar broadening.

^{19}F {^{1}H} NMR (376 MHz, DMSO-d_6, 25 °C, δ): –120.99 (1F), –140.63 (q, J = 40.4 Hz, 3F).

^{11}B NMR (128 MHz, DMSO-d_6, 25 °C, δ): 1.95 (q, J = 40.4 Hz).

HRMS ESIpos (m/z) calc'd for $C_5H_4NBNaF_4^+$ [M+Na]$^+$, 188.0265; found, 188.0267. Deviation: +1.1 ppm.

(4-Fluoropyridin-1-ium-2-yl)trifluoroborate (1p-S)

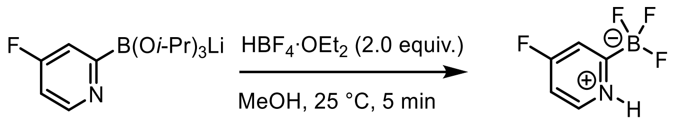

1p-S

Under an ambient atmosphere, triisopropyl(4-fluoropyridine-2-yl)lithium borate (116.4 mg, 0.400 mmol, 1.0 equiv.), and MeOH (2 mL, c = 0.2 M) were added to a 4-mL vial, followed by $HBF_4 \cdot OEt_2$ (108 μL, 129.6 mg, 0.800 mmol, 2.0 equiv.). After solvation, the solvent was removed by rotary evaporation. The residue was purified by chromatography on silica gel, eluting with dichloromethane/methanol (from 100:1 to 50:1, v:v) to afford 49.1 mg (74% yield) of the title compound as a pale yellow solid.

Melting Point = 134-135 °C.

R*f* = 0.50 (dichloromethane/MeOH, 9:1, v/v (UV))

NMR Spectroscopy:

^{1}H NMR (400 MHz, CD_3CN, 25 °C, δ): 12.60 (brs, 1H), 8.53 (t, J = 6.0 Hz, 1H), 7.69 (d, J = 5.9 Hz, 1H), 7.53 (td, J = 7.1, 2.9 Hz, 1H).

^{13}C {^{1}H} NMR (101 MHz, CD_3CN, 25 °C, δ): 173.39 (d, J = 277.7 Hz), 144.85 (d, J = 12.2 Hz), 117.83 (d, J = 17.8 Hz), 114.74 (d, J = 23.2 Hz). The carbon directly attached to the boron atom was not detected due to quadrupolar broadening.

^{19}F {^{1}H} NMR (376 MHz, CD_3CN, 25 °C, δ): –86.65 (1F), –147.53 (q, J = 39.2 Hz, 3F).

^{11}B NMR (128 MHz, CD_3CN, 25 °C, δ): 0.36 (q, J = 39.2 Hz).

HRMS ESIpos (m/z) calc'd for $C_5H_4NBNaF_4^+$ [M+Na]$^+$, 188.0265; found, 188.0261. Deviation: –2.1 ppm.

Stability tests

The stability tests were carried out according to a literature procedure[16]. The solution of **1** was prepared by dissolving **1** (18.5 mg, 0.100 mmol) in the mixed solution of PBS buffer (0.2 mL) and MeCN (0.5 mL). After that, the solution was added D_2O (1.0 mL) and sodium trifluoroacetate (18.5 mg), and 0.5 mL of the resulting mixture was used for NMR measurement. The remaining **1** was determined by ^{19}F NMR.

Entry	Time	Remaining **1a**	Remaining **1b**	Remaining **1c**	Remaining **1m**
1	0 day	100%	100%	100%	100%
2	3 days	100%	94%	86%	70%
3	7 days	100%	84%	77%	67%
4	12 days	99%	74%	66%	59%

Stability test of 1a in PBS buffer:

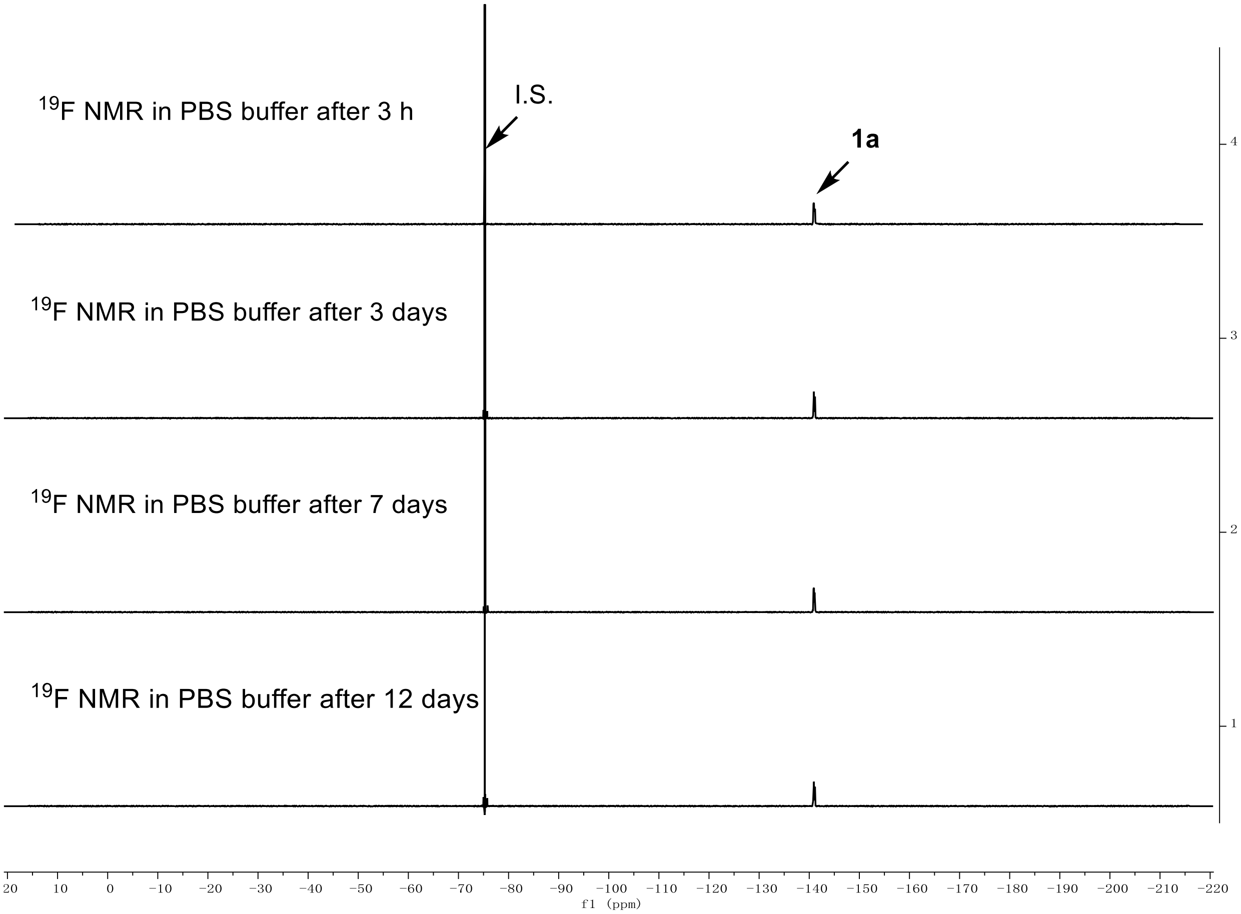

Stability test of 1b in PBS buffer:

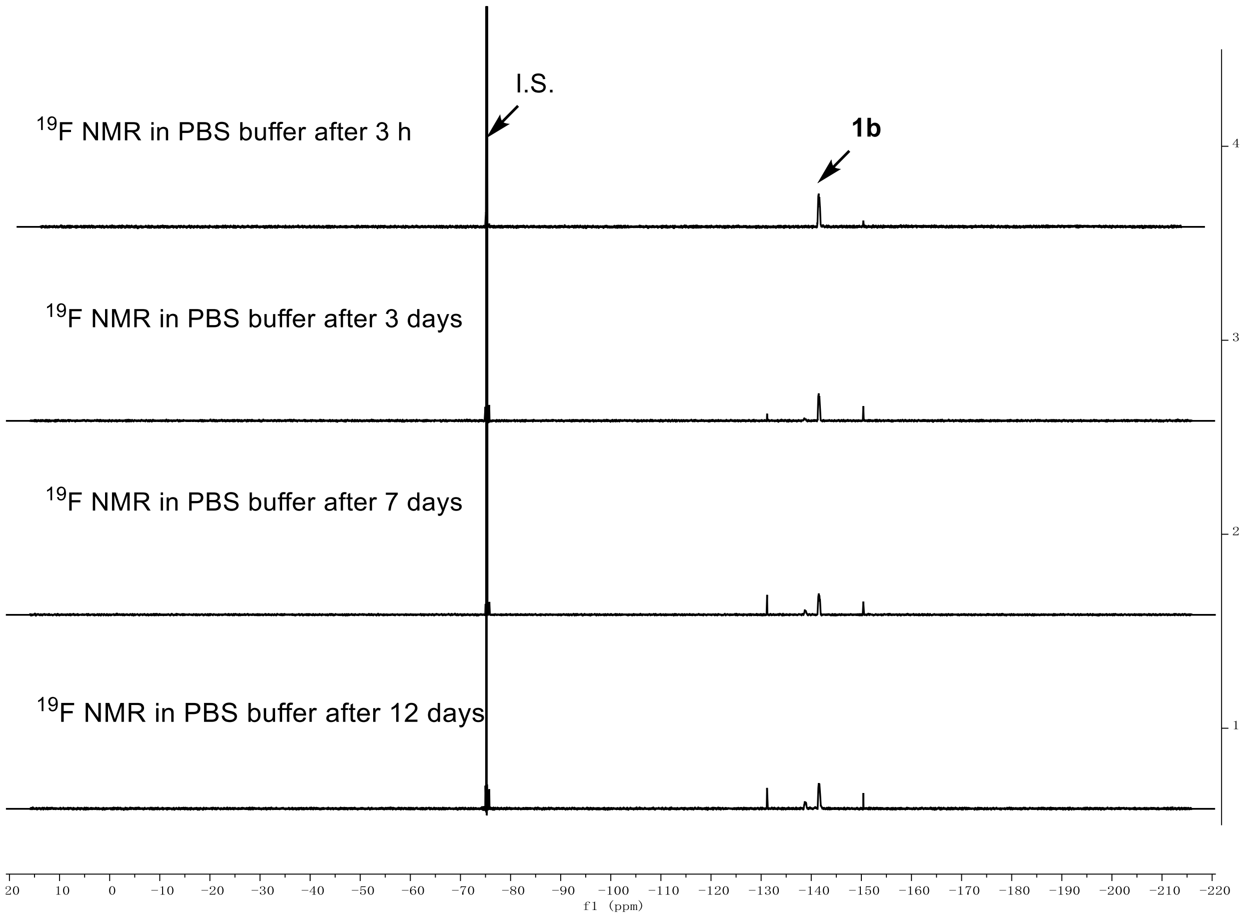

Stability test of 1c in PBS buffer:

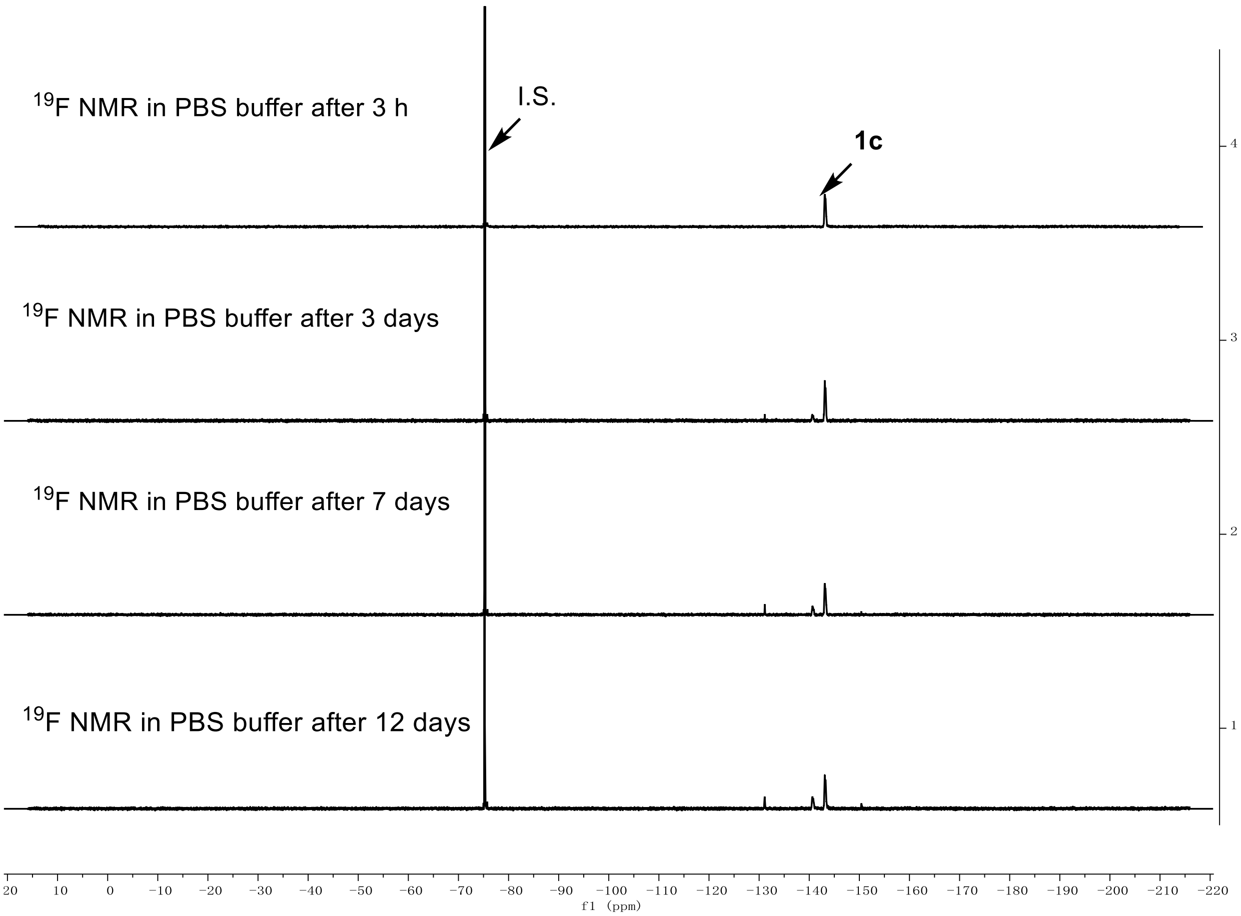

Stability test of 1m in PBS buffer:

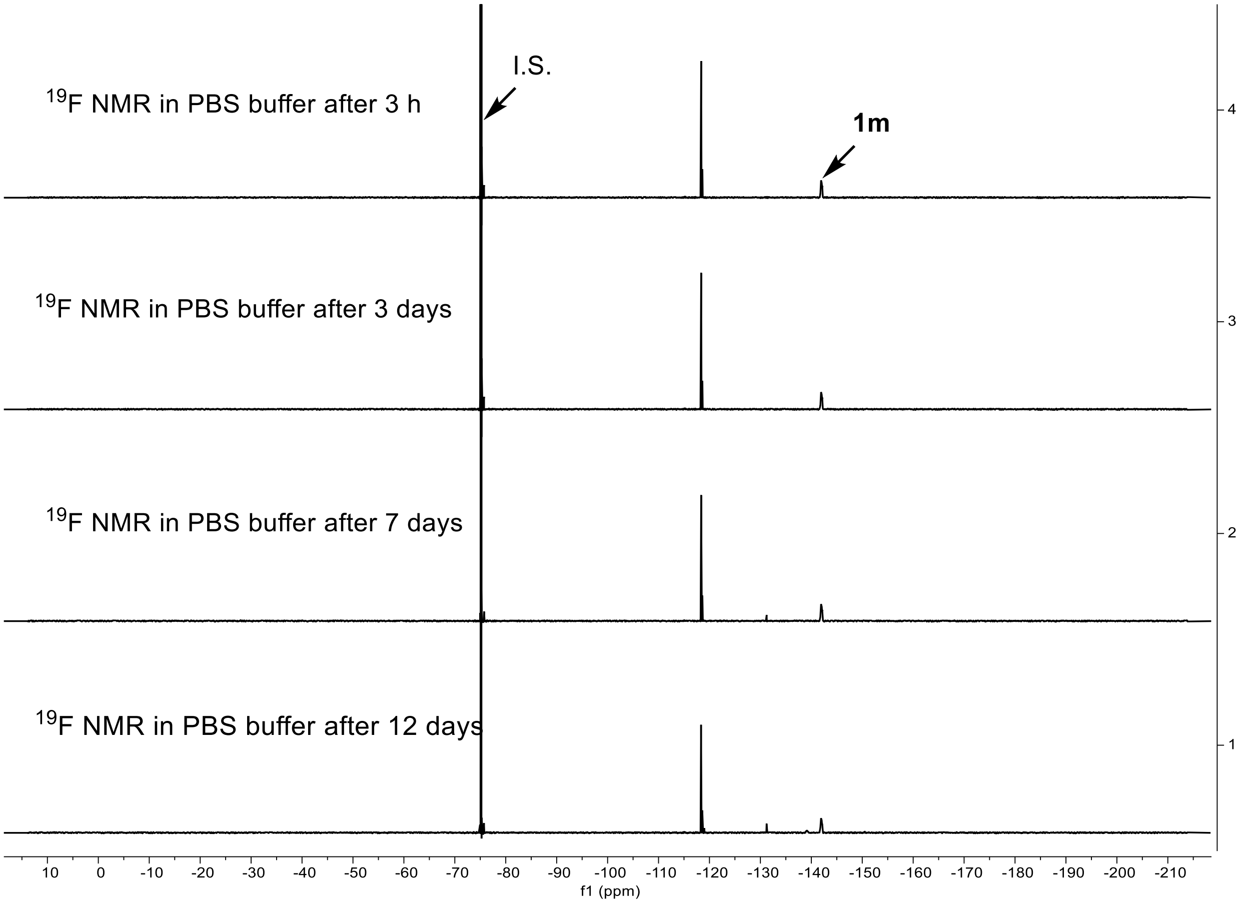

Stability test in pyridazine-HCl buffer

The stability test experiment was carried out according to a literature procedure[16]. The solution of **1m** was prepared by dissolving **1m** (21.7 mg, 0.100 mmol) in the pyridazine-HCl buffer (0.5 mL). After that, the solution was added internal standard sodium trifluoroacetate (21.7 mg), and was used for NMR measurement. The remaining **1m** was determined by ^{19}F NMR.

Entry	1	2	3
Time	0 day	1 day	5 days
Remaining **1m**	100%	98%	96%

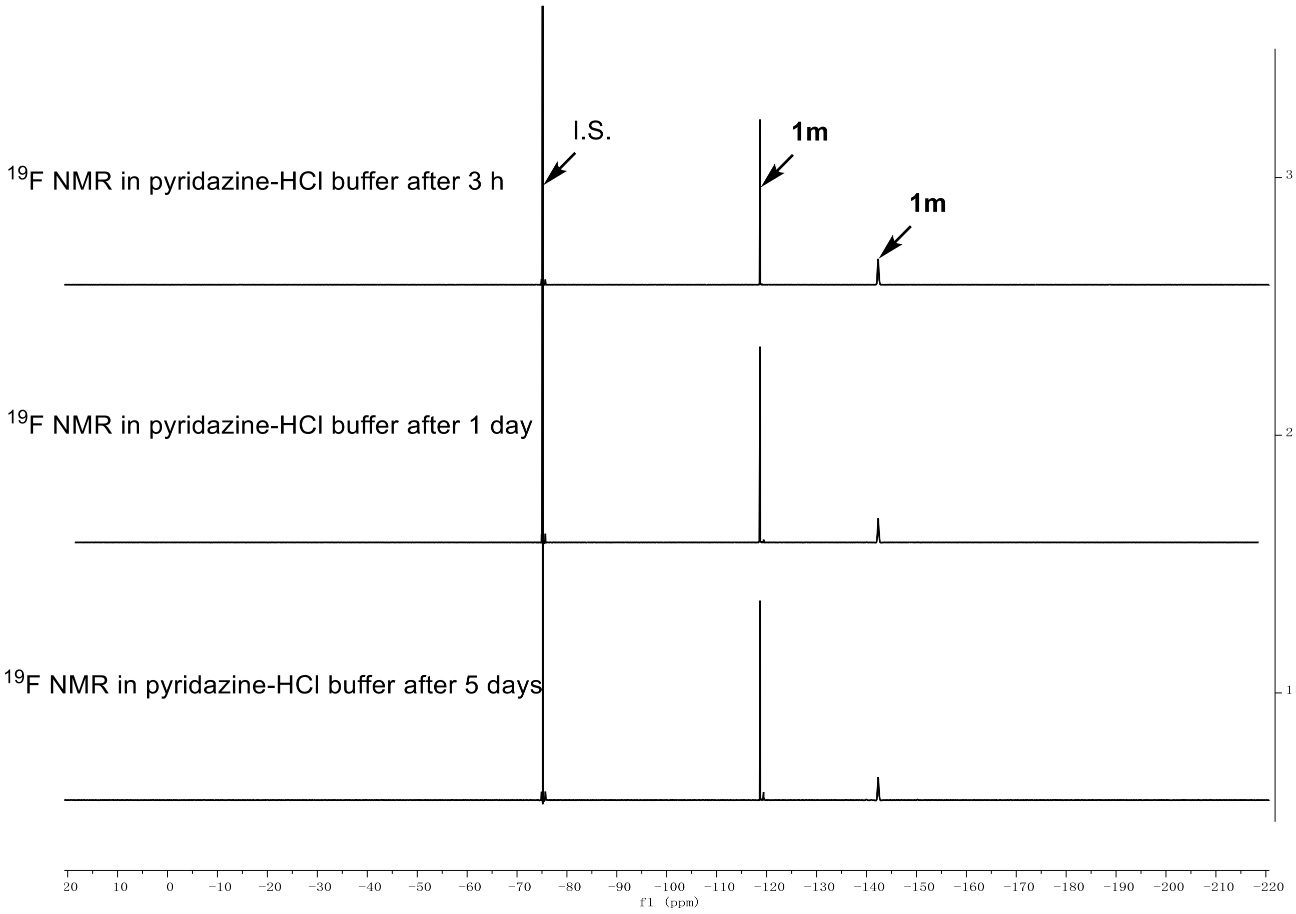

The stability test experiment was carried out according to a literature procedure[16]. The solution of **1m** was prepared by dissolving **1m** (21.7 mg, 0.100 mmol) in 0.5 mL of a mixed solvent (aqueous pyridazine-HCl buffer:DMF = 2:1), followed by heating at 90 °C for 10 min. After that, the solution was added internal standard sodium trifluoroacetate (21.7 mg), and was used for NMR measurement. The remaining **1m** was determined by ^{19}F NMR.

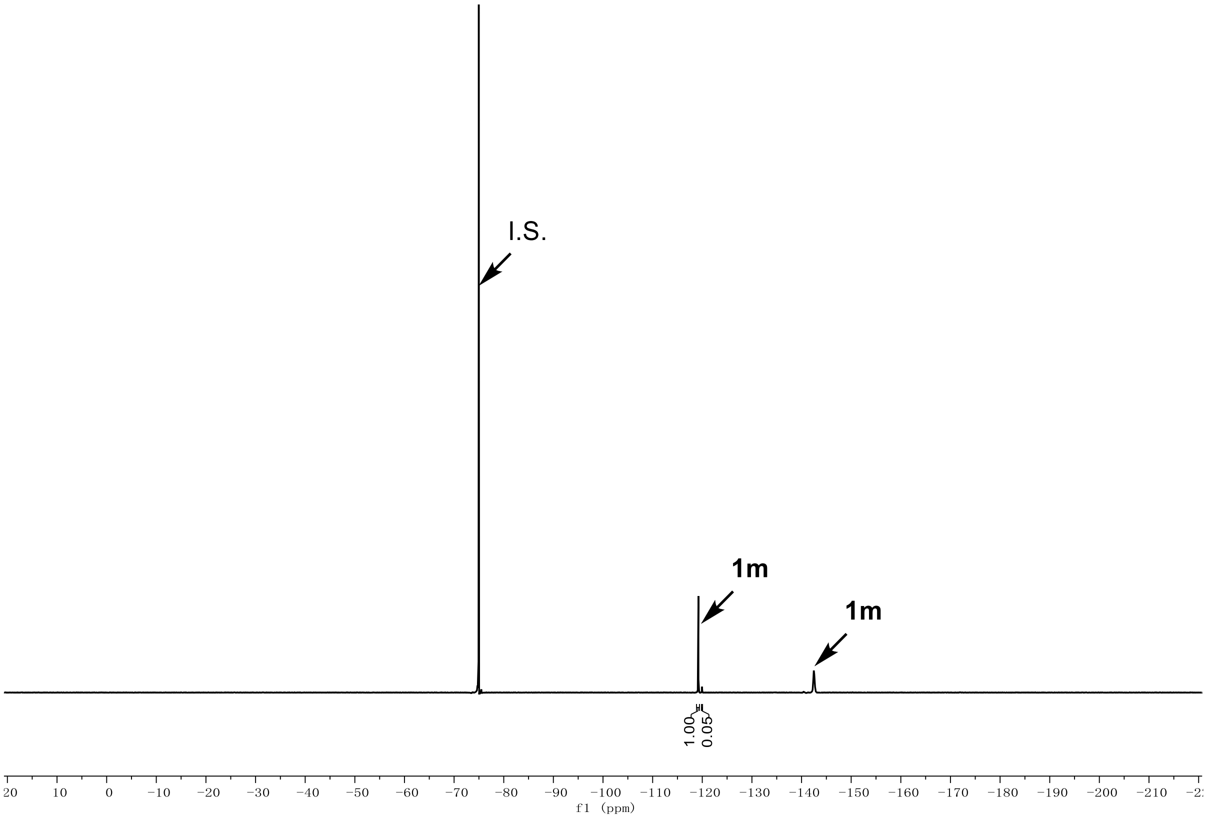

Discussion: An additional stability test of reagent **1m**, conducted at 90 °C for 10 min in a mixed solvent (aqueous pyridazine-HCl buffer:DMF = 2:1, ~60% water), showed <10% decomposition.

The solution was prepared by adding 0.5 mL of the same mixed solvent (aqueous pyridazine-HCl buffer:DMF = 2:1) without reagent **1m**, followed by heating at 90 °C for 10 min. After that, the solution was added internal standard sodium trifluoroacetate (21.7 mg), and was used for NMR measurement. The possible decomposition of DMF was analyzed by ^{1}H NMR.

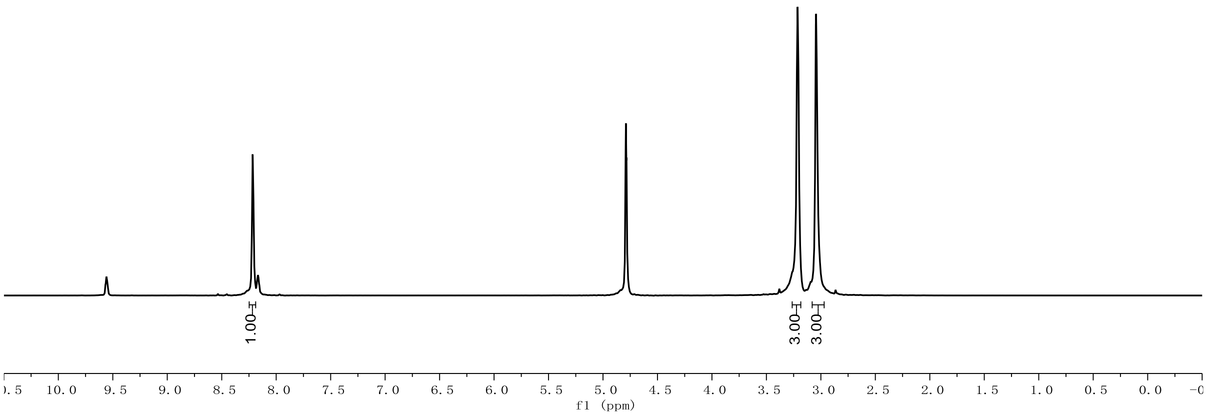

Disussion: Control experiments conducted without reagent **1m**, using only the acidic buffer and DMF at 90 °C, showed no obvious decomposition of DMF.

Lipophilicity (*D*) measurements

The log $D_{7.4}$ value was measured based on a modified procedure from literature[17]. D_2O buffer (pH 7.4) was prepared from D_2O (5 mL), Na_2HPO_4 (95.1mg) and NaH_2PO_4 (18.0 mg). An aliquot (1.5 mL) of the D_2O buffer was saturated with the analyte. The saturated solution was mixed several times and stored at room temperature overnight. Approximately 700 µL of the saturated D_2O phase was transferred to a tarred 5 mm NMR tube. A quantitative ^{1}H-NMR spectrum with solvent (H_2O) suppression pulse sequence was measured and the number of scans was set to a number that resulted in a signal-to-noise ratio of approximately 50:1. Afterwards, 1-octanol (200–300 µL) was directly added to the NMR tube. The mixture was carefully shacked vigourously 3 times for 2 minutes and then stored at room temperature overnight, until the 1-octanol was completely separated from the D_2O phase. After that, a quantitative ^{1}H-NMR spectrum was measured using the same conditions as before.

Entry	Analyte	Buffer volume (mL)	Octanol volume (mL)	$D_{7.4}$	*log* $D_{7.4}$
1	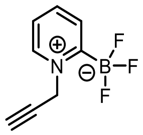 **1a**	0.7	0.2	1.7	0.2
2	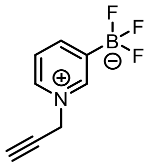 **1b**	0.7	0.3	11.4	1.1
3	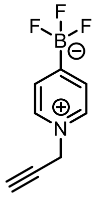 **1c**	0.7	0.3	< 0.1	< –1
4	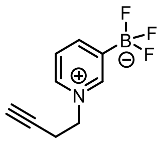 **1j**	0.7	0.3	1.2	0.1

5	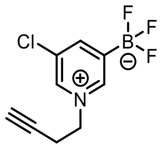 **1k**	0.7	0.3	0.9	–0.1
6	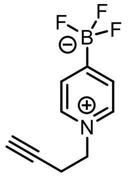 **1l**	0.7	0.3	5.2	0.7
7	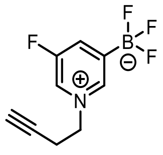 **1m**	0.7	0.3	7.4	0.9

X-Ray crystallographic data

(1-(Prop-2-yn-1-yl)pyridin-1-ium-2-yl)trifluoroborate (CCDC 2428065)

The single crystal of (1-(prop-2-yn-1-yl)pyridin-1-ium-2-yl)trifluoroborate was obtained by recrystallization of **1a** in MeCN (2 mL, *c* = 0.4 M). The atoms are depicted with 50% probability ellipsoids. All hydrogens were found and refined isotropically. The detailed X-ray structure is shown in Figure S1 and crystallographic data are summarized in Table S1.

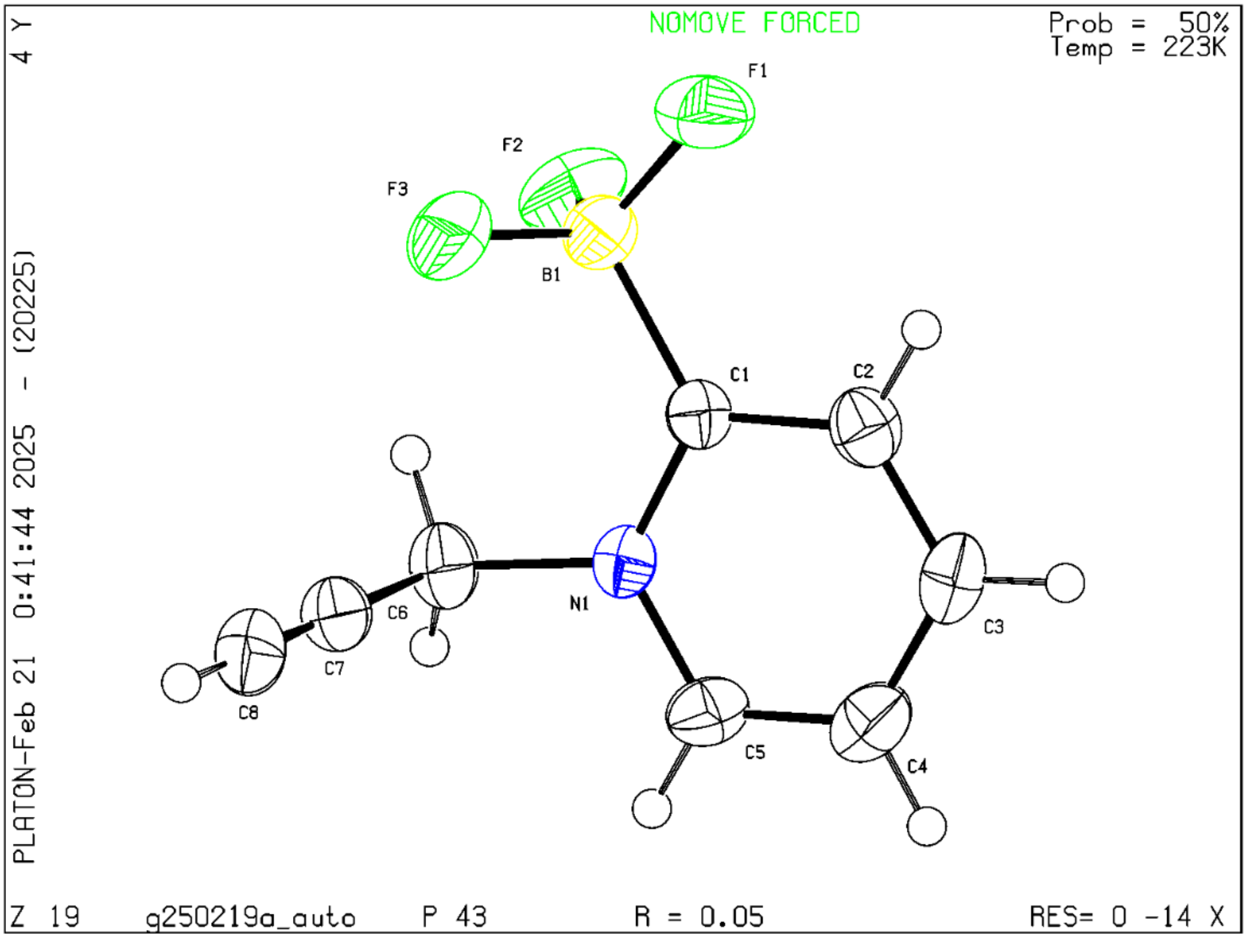

Figure S1. X-ray structure of (1-(prop-2-yn-1-yl)pyridin-1-ium-2-yl)trifluoroborate

Table S1. Crystallographic data

Empirical formula	C8 H7 B F3 N
Color	colourless
Formula weight	184.96 g·mol^{-1}
Temperature	223.0 K
Wavelength	0.71073 Å
Crystal system	TETRAGONAL
Space group	P 4_3
Unit cell dimensions	a = 7.7602 (10) Å α = 90°<br>b = 7.7602 (2) Å β = 90°<br>c = 13.525 (3) Å γ = 90°
Volume	814.5(3) Å^3
Z	4
Density (calculated)	1.508 g/cm^3
Absorption coefficient	0.136 mm^{-1}
Crystal size	0.4 x 0.35 x 0.2 mm^3
F(000)	376.0
θ range for data collection	2.625 to 30.285°
Index ranges	-8 ≤ h ≤ 8, -10 ≤ k ≤ 6, -10 ≤ l ≤ 18
Reflections collected	2510
Independent reflections	1652 [R_{int} =0.0461]
Reflections with I>2σ(I)	1120
Completeness to θ = 25.242°	100 %
Absorption correction	Gaussian
Refinement method	Full-matrix least-squares on F^2
Data / restraints / parameters	1652 / 1 / 118
Goodness-of-fit on F^2	1.056
Final R indices [I>2σ(I)]	R_1 = 0.0534 wR^2 = 0.1084
R indices (all data)	R_1 = 0.0865 wR^2 = 0.1335
Largest diff. peak and hole	0.24 and -0.20 e·Å^{-3}

(1-(Prop-2-yn-1-yl)pyridin-1-ium-3-yl)trifluoroborate (CCDC 2457625)

The single crystal of (1-(prop-2-yn-1-yl)pyridin-1-ium-3-yl)trifluoroborate was obtained by recrystallization of **1b** in MeCN (2 mL, *c* = 0.4 M). The atoms are depicted with 50% probability ellipsoids. All hydrogens were found and refined isotropically. The detailed X-ray structure is shown in Figure S2 and crystallographic data are summarized in Table S2.

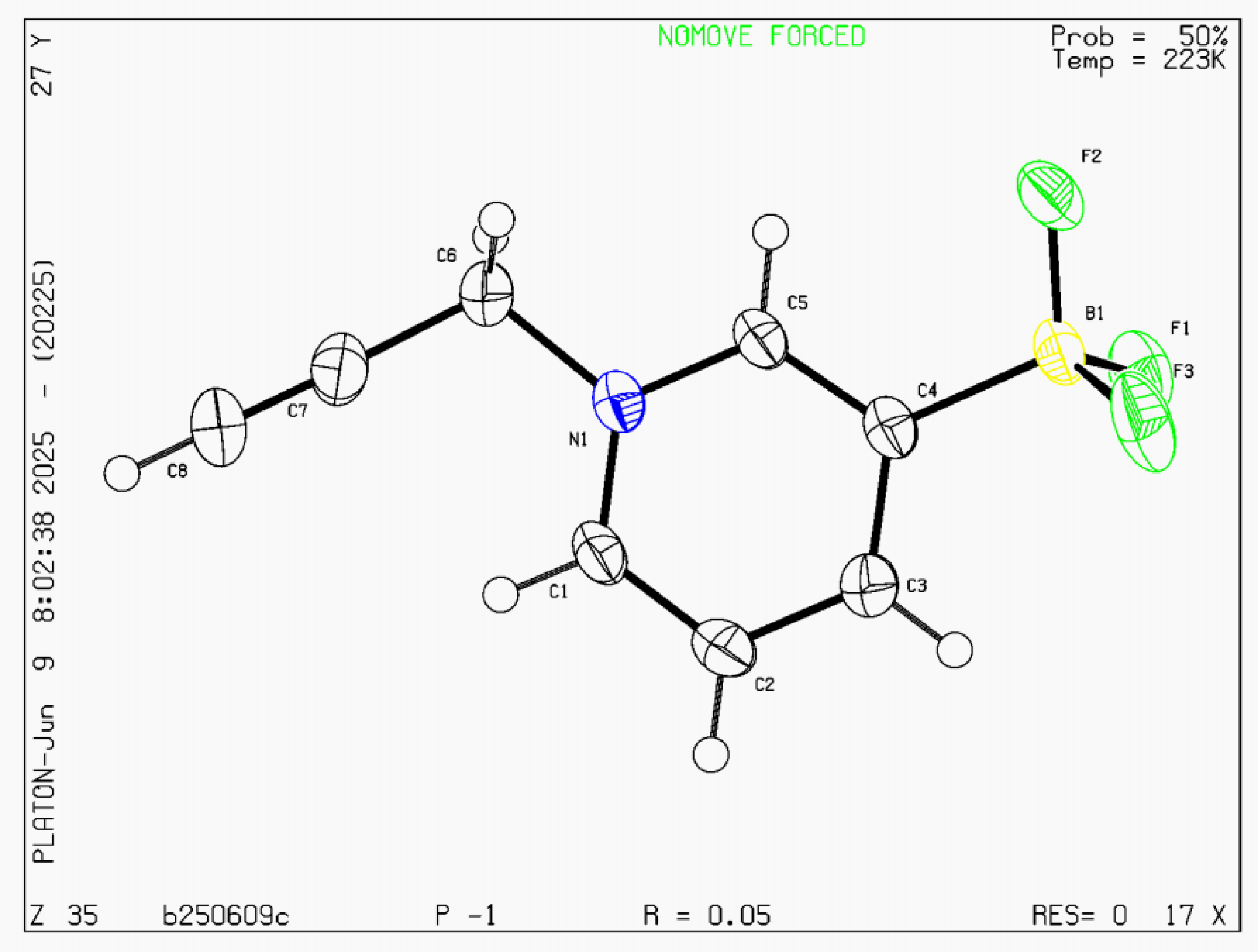

Figure S2. X-ray structure of (1-(prop-2-yn-1-yl)pyridin-1-ium-3-yl)trifluoroborate

Table S2. Crystallographic data

Empirical formula	C8 H7 B F3 N
Color	colourless
Formula weight	184.96 g·mol^{-1}
Temperature	223.0 K
Wavelength	0.71073 Å
Crystal system	TRICLINIC
Space group	P-1
Unit cell dimensions	a = 7.1101 (3) Å α = 72.717(2)°<br>b = 7.7077 (3) Å β = 86.701(2)°<br>c = 7.9353 (3) Å γ = 82.814(2)°
Volume	411.88(3) Å^3
Z	2
Density (calculated)	1.491 g/cm^3
Absorption coefficient	0.135 mm^{-1}
Crystal size	0.3 × 0.3 × 0.3 mm^3
F(000)	188.0
θ range for data collection	2.689 to 27.486°
Index ranges	-9 ≤ h ≤ 9, -10 ≤ k ≤ 10, -10 ≤ l ≤ 10
Reflections collected	7191
Independent reflections	1875 [R_{int} = 0.0575]
Reflections with I>2σ(I)	1523
Completeness to θ = 25.242°	99.5
Absorption correction	Gaussian
Refinement method	Full-matrix least-squares on F^2
Data / restraints / parameters	1875/0/118
Goodness-of-fit on F^2	1.049
Final R indices [I>2σ(I)]	R_1 = 0.0517, wR_2 = 0.1249
R indices (all data)	R_1 = 0.0660, wR_2 = 0.1357
Largest diff. peak and hole	0.36/-0.24

(1-(Prop-2-yn-1-yl)pyridin-1-ium-4-yl)trifluoroborate (CCDC 2456372)

The single crystal of (1-(prop-2-yn-1-yl)pyridin-1-ium-4-yl)trifluoroborate was obtained by recrystallization of **1c** in MeCN (2 mL, *c* = 0.4 M). The atoms are depicted with 50% probability ellipsoids. All hydrogens were found and refined isotropically. The detailed X-ray structure is shown in Figure S3 and crystallographic data are summarized in Table S3.

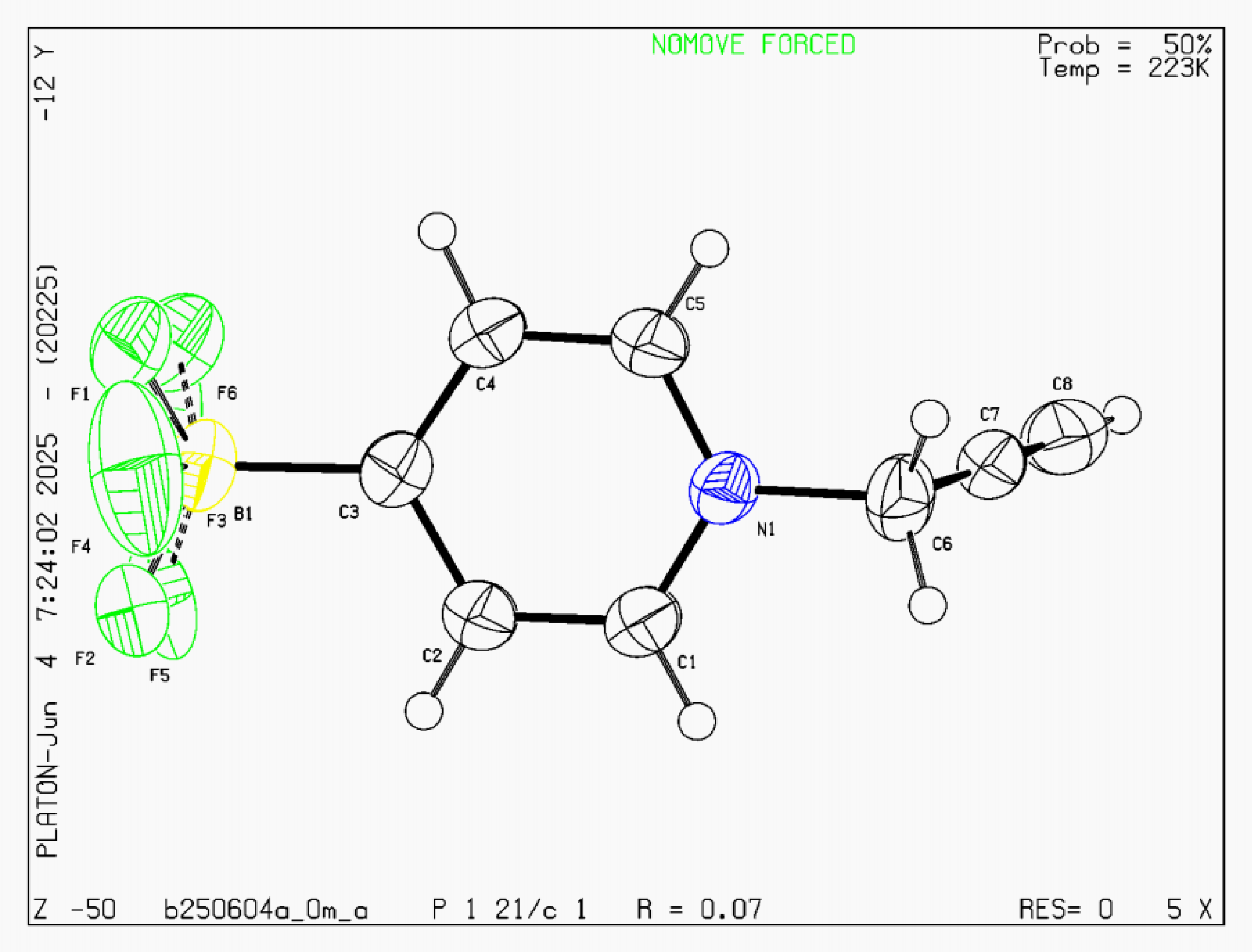

Figure S3. X-ray structure of (1-(prop-2-yn-1-yl)pyridin-1-ium-4-yl)trifluoroborate

Table S3. Crystallographic data

Empirical formula	C8 H7 B F3 N
Color	colourless
Formula weight	184.96 g·mol^{-1}
Temperature	223.0 K
Wavelength	0.71073 Å
Crystal system	MONOCLINC
Space group	$P2_1/c$
Unit cell dimensions	a = 6.6841 (13) Å α = 90°<br>b = 15.985 (3) Å β = 86.701(2)°<br>c = 7.8573 (13) Å γ = 90°
Volume	839.5(3) Å^3
Z	4
Density (calculated)	1.436 g/cm^3
Absorption coefficient	0.132 mm^{-1}
Crystal size	0.2 × 0.2 × 0.2 mm^3
F(000)	376.0
θ range for data collection	2.548 to 27.482
Index ranges	-8 ≤ h ≤ 8, -20 ≤ k ≤ 20, -9 ≤ l ≤ 9
Reflections collected	10135
Independent reflections	1877 [R_{int} = 0.0706]
Reflections with I>2σ(I)	1304
Completeness to θ = 25.242°	1877/9/146
Absorption correction	Gaussian
Refinement method	Full-matrix least-squares on F^2
Data / restraints / parameters	1877/9/146
Goodness-of-fit on F^2	1.107
Final R indices [I>2σ(I)]	R_1 = 0.0699, wR_2 = 0.1632
R indices (all data)	R_1 = 0.1078, wR_2 = 0.1919
Largest diff. peak and hole	0.24/-0.27

(4-Methoxy-1-(prop-2-yn-1-yl)pyridin-1-ium-2-yl)trifluoroborate (CCDC 2440358)

The single crystal of (4-methoxy-1-(prop-2-yn-1-yl)pyridin-1-ium-2-yl)trifluoroborate was obtained by recrystallization of **1t** in $CDCl_3$ (0.5 mL, *c* = 0.8 M). The atoms are depicted with 50% probability ellipsoids. All hydrogens were found and refined isotropically. The detailed X-ray structure is shown in Figure S4 and crystallographic data are summarized in Table S4.

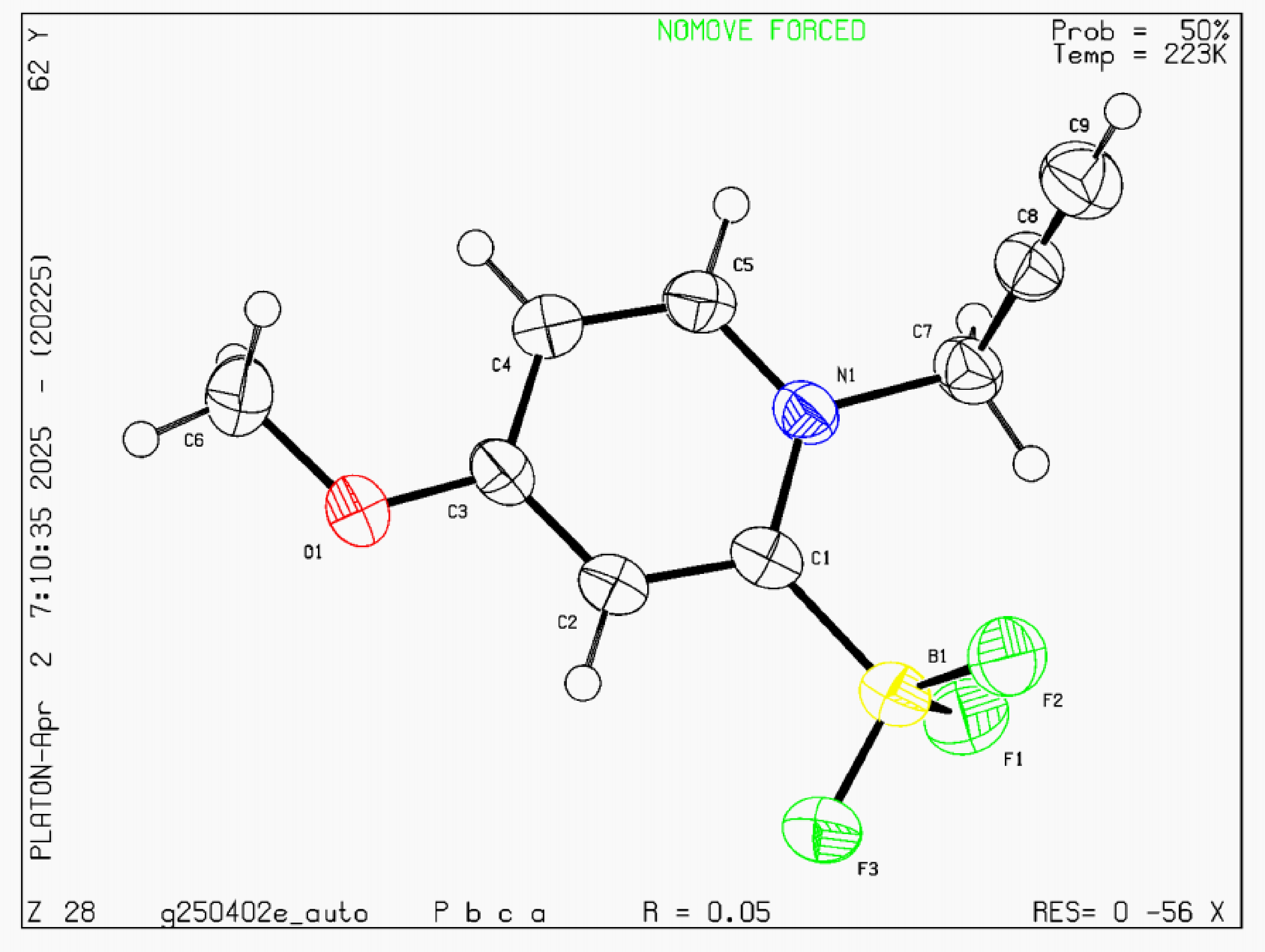

Figure S4. X-ray structure of (4-methoxy-1-(prop-2-yn-1-yl)pyridin-1-ium-2-yl)trifluoroborate

Table S4. Crystallographic data

Empirical formula	C9 H9 B F3 N O
Color	colourless
Formula weight	214.98 g·mol^{-1}
Temperature	223.0 K
Wavelength	0.71073 Å
Crystal system	ORTHORHOMBIC
Space group	*P* bca
Unit cell dimensions	a = 9.0164(13)Å α = 90°<br>b = 13.8720(16) Å β = 90°<br>c = 15.813(2) Å γ = 90°
Volume	1977.8(5) Å^3
Z	8
Density (calculated)	1.444 g/cm^3
Absorption coefficient	0.130 mm^{-1}
Crystal size	0.4 x 0.35 x 0.3 mm^3
F(000)	880.0
θ range for data collection	2.576 to 30.34°
Index ranges	-12 ≤ h ≤ 9, -17 ≤ k ≤ 18, -12 ≤ l ≤ 20
Reflections collected	7745
Independent reflections	2607 [R_{int} =0.0452]
Reflections with I>2σ(I)	1513
Completeness to θ = 25.242°	100 %
Absorption correction	Gaussian
Refinement method	Full-matrix least-squares on F^2
Data / restraints / parameters	2607/ 0 / 137
Goodness-of-fit on F^2	1.054
Final R indices [I>2σ(I)]	R_1 = 0.0500 wR^2 = 0.1107
R indices (all data)	R_1 = 0.0979 wR^2 = 0.1415
Largest diff. peak and hole	0.21 and -0.23 e·Å^{-3}

(4-Chloro-1-(prop-2-yn-1-yl)pyridin-1-ium-2-yl)trifluoroborate (CCDC 2440357)

The single crystal of (4-chloro-1-(prop-2-yn-1-yl)pyridin-1-ium-2-yl)trifluoroborate was obtained by recrystallization of **1s** in MeCN (2 mL, *c* = 0.4 M). The atoms are depicted with 50% probability ellipsoids. All hydrogens were found and refined isotropically. The detailed X-ray structure is shown in Figure S5 and crystallographic data are summarized in Table S5.

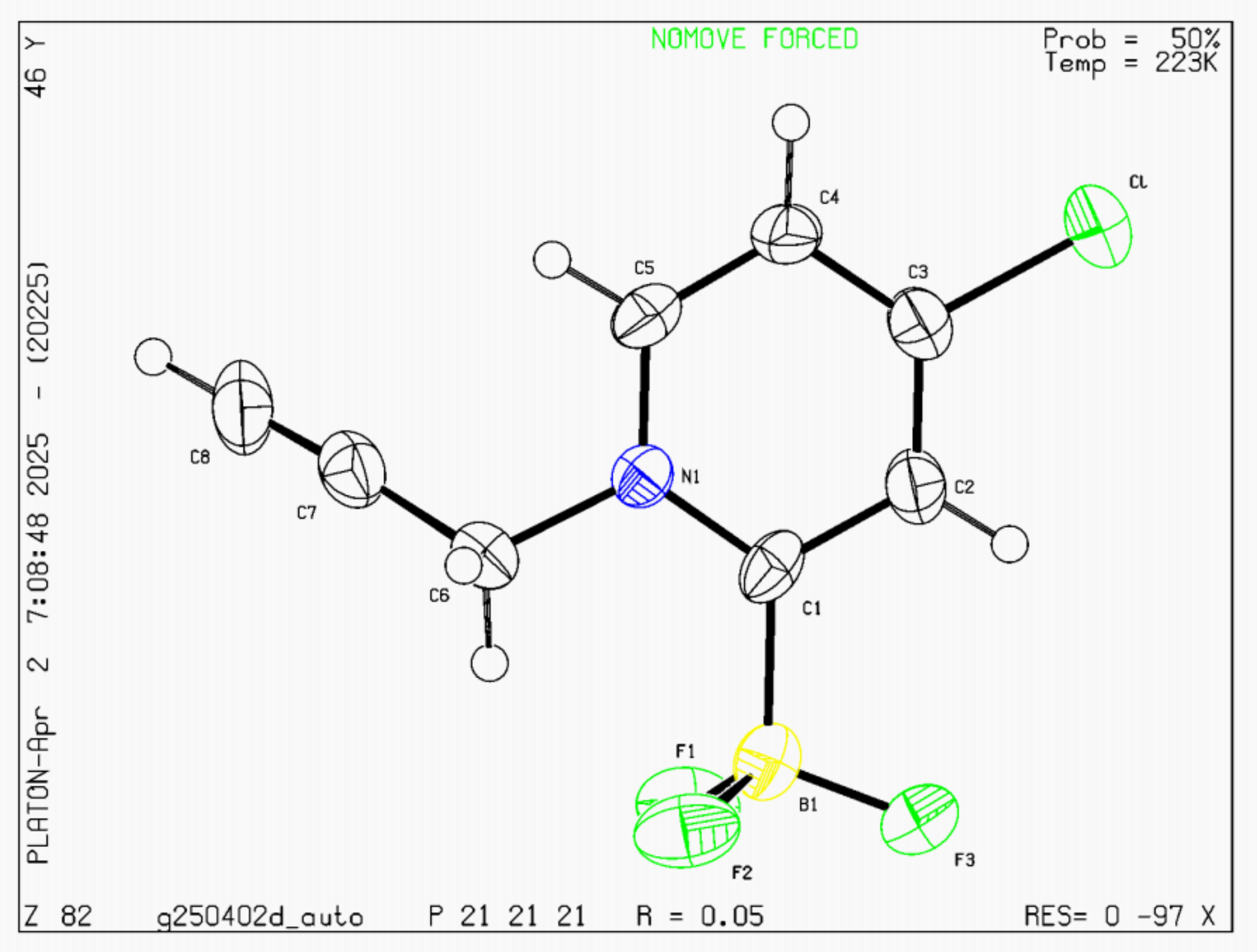

Figure S5. X-ray structure of (4-chloro-1-(prop-2-yn-1-yl)pyridin-1-ium-2-yl)trifluoroborate

Table S5. Crystallographic data

Empirical formula	C8 H6 B Cl F3 N
Color	colourless
Formula weight	219.40 g·mol^{-1}
Temperature	223.0 K
Wavelength	0.71073 Å
Crystal system	ORTHORHOMBIC
Space group	P $2_12_12_1$
Unit cell dimensions	a = 4.9656(14) Å α = 90°<br>b = 11.938(3) Å β = 90°<br>c = 15.785(4) Å γ = 90°
Volume	935.7(4) Å^3
Z	4
Density (calculated)	1.557 g/cm^3
Absorption coefficient	0.408 mm^{-1}
Crystal size	0.4 x 0.35 x 0.3 mm^3
F(000)	440.0
θ range for data collection	2.139 to 25.015°
Index ranges	-5 ≤ h ≤ 5, -10 ≤ k ≤ 14, -18 ≤ l ≤ 17
Reflections collected	3128
Independent reflections	1554 [R_{int} =0.0542]
Reflections with I>2σ(I)	1021
Completeness to θ = 25.242°	100 %
Absorption correction	Gaussian
Refinement method	Full-matrix least-squares on F^2
Data / restraints / parameters	1554 / 0 / 127
Goodness-of-fit on F^2	1.015
Final R indices [I>2σ(I)]	R_1 = 0.0506 wR^2 = 0.0799
R indices (all data)	R_1 = 0.0925 wR^2 = 0.1014
Largest diff. peak and hole	0.19 and -0.23 e·Å^{-3}

General procedure for the click reaction

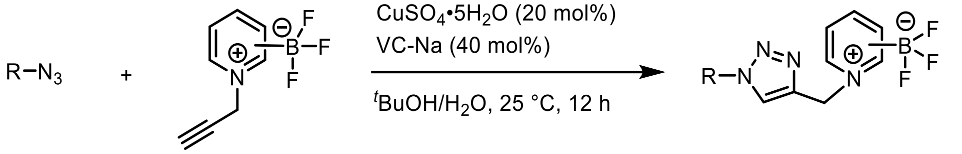

Under an ambient atmosphere, R-N_3 (0.450 mmol, 1.50 equiv.) in ^{t}BuOH:H_2O (1:1, 1.5 mL, 0.2 M) was to a 4 mL vial containing a magnetic stir bar. Subsequently, $CuSO_4{\cdot}5H_2O$ (15 mg, 60 μmol, 20 mol%), and sodium ascorbate (23.8 mg, 0.12 mmol, 40 mol%) were added was added into the tube, followed by pyridinium trifluoroborate salt (0.300 mmol, 1.00 equiv.). The tube was sealed with a Teflon cap, and the reaction mixture was stirred vigorously at 25 °C. After the indicated time, the reaction vessel was opened to air, click products from **1a** were extracted into EtOAc, and click products from **1b** and **1c** were concentrated by rotary evaporation directly. The residue was purified by chromatography on silica gel to obtain the pure product.

Preparation of conjugates

(1-((1-Benzyl-1*H*-1,2,3-triazol-4-yl)methyl)pyridin-1-ium-2-yl)trifluoroborate (3a)

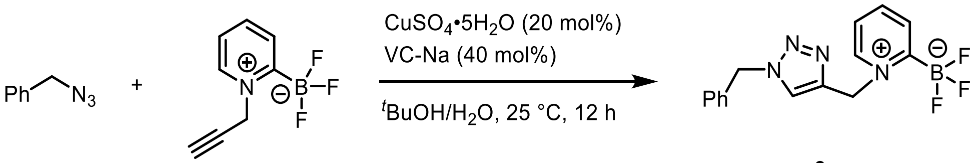

2a **1a** **3a**

Under an ambient atmosphere, (azidomethyl)benzene (59.5 mg, 0.450 mmol, 1.50 equiv.) in ^{t}BuOH:H_2O (1:1, 1.5 mL, 0.2 M) was to a 4 mL vial containing a magnetic stir bar. Subsequently, $CuSO_4{\cdot}5H_2O$ (15 mg, 60 μmol, 20 mol%), and sodium ascorbate (23.8 mg, 120 μmol, 40 mol%) were added was added into the tube, followed by 2-pyridinium trifluoroborate salt (55.5 mg, 0.300 mmol, 1.00 equiv.). The tube was sealed with a Teflon cap, and the reaction mixture was stirred vigorously at 25 °C. After the indicated time, the reaction vessel was opened to air, and the resulting mixture was quenched with saturated $NaHCO_3$ (10 mL), then extracted into EtOAc (3 × 10 mL). The combined organic phase was washed with brine, dried over Na_2SO_4, and concentrated by rotary evaporation. The residue was purified by chromatography on silica gel, eluting with dichloromethane/methanol (from 100:1 to 50:1, v:v) to afford 85.1 mg (89% yield) of the title compound as a pale yellow solid.

R*f* = 0.70 (dichloromethane/methanol, 10:1, v/v (UV))

NMR Spectroscopy:

^{1}H NMR (400 MHz, CD_3CN, 25 °C, δ): 8.75 (d, *J* = 6.3 Hz, 1H), 8.19 (t, *J* = 7.7 Hz, 1H), 8.07 (d, *J* = 7.8 Hz, 1H), 7.95 (s, 1H), 7.71 (ddd, *J* = 7.9, 6.3, 1.8 Hz, 1H), 7.49 – 7.16 (m, 5H), 5.91 (s, 2H), 5.51 (s, 2H).

^{13}C {^{1}H} NMR (101 MHz, $CDCl_3$, 25 °C, δ): 143.27, 142.00, 141.64, 134.03, 132.88 (q, *J* = 2.1 Hz), 129.31, 129.11, 128.38, 125.40(2C), 54.58, 54.05 (q, *J* = 3.1 Hz). The carbon directly attached to the boron atom was not detected due to quadrupolar broadening.

^{19}F {^{1}H} NMR (376 MHz, $CDCl_3$, 25 °C, δ): –142.30 (q, *J* = 40.7 Hz).

^{11}B NMR (128 MHz, $CDCl_3$, 25 °C, δ): 0.77 (q, *J* = 40.7 Hz).

HRMS ESI (m/z) calc'd for $C_{15}H_{14}N_4BNaF_3^+$ [M+Na]$^+$, 341.1156; found, 341.1155. Deviation: –0.3 ppm.

(1-((1-Benzyl-1*H*-1,2,3-triazol-4-yl)methyl)pyridin-1-ium-3-yl)trifluoroborate (4a)

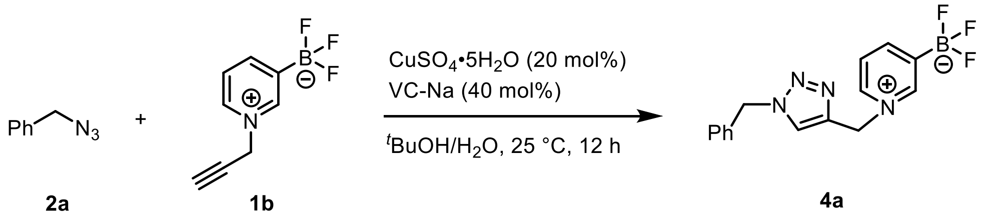

Under an ambient atmosphere, (azidomethyl)benzene (20.0 mg, 0.150 mmol, 1.50 equiv.) in ^{t}BuOH:H_2O (1:1, 0.5 mL, 0.2 M) was to a 4 mL vial containing a magnetic stir bar. Subsequently, $CuSO_4·5H_2O$ (5 mg, 20 µmol, 20 mol%), and sodium ascorbate (7.9 mg, 40 µmol, 40 mol%) were added was added into the tube, followed by 3-pyridinium trifluoroborate salt (18.5 mg, 0.100 mmol, 1.00 equiv.). The tube was sealed with a Teflon cap, and the reaction mixture was stirred vigorously at 25 °C. After the indicated time, the reaction vessel was opened to air, and the resulting mixture was concentrated by rotary evaporation. The residue was purified by chromatography on silica gel, eluting with dichloromethane/methanol (from 100:1 to 50:1, v:v) to afford 30.4 mg (97% yield) of the title compound as a white solid.

R*f* = 0.70 (dichloromethane/methanol, 10:1, v/v (UV))

NMR Spectroscopy:

^{1}H NMR (400 MHz, CD_3CN, 25 °C, δ): 8.68 (s, 1H), 8.54 (d, *J* = 6.1 Hz, 1H), 8.46 (d, *J* = 7.4 Hz, 1H), 8.00 (d, *J* = 2.3 Hz, 1H), 7.79 (t, *J* = 6.8 Hz, 1H), 7.45 – 7.22 (m, 5H), 5.68 (s, 2H), 5.55 (s, 2H).

^{13}C {^{1}H} NMR (101 MHz, CD_3CN, 25 °C, δ): 149.85, 146.69, 142.23, 141.32, 136.37, 129.94, 129.51, 129.13, 127.85, 125.77, 55.98, 54.68. The carbon directly attached to the boron atom was not detected due to quadrupolar broadening.

^{19}F {^{1}H} NMR (376 MHz, CD_3CN, 25 °C, δ): –144.12 (q, *J* = 45.0 Hz).

^{11}B NMR (128 MHz, CD_3CN, 25 °C, δ): 1.80 (q, *J* = 45.0 Hz).

HRMS ESI (m/z) calc'd for $C_{15}H_{14}N_4BNaF_3^+$ [M+Na]$^+$, 341.1156; found, 341.1152. Deviation: –1.2 ppm.

(1-((1-Benzyl-1*H*-1,2,3-triazol-4-yl)methyl)pyridin-1-ium-4-yl)trifluoroborate (5a)

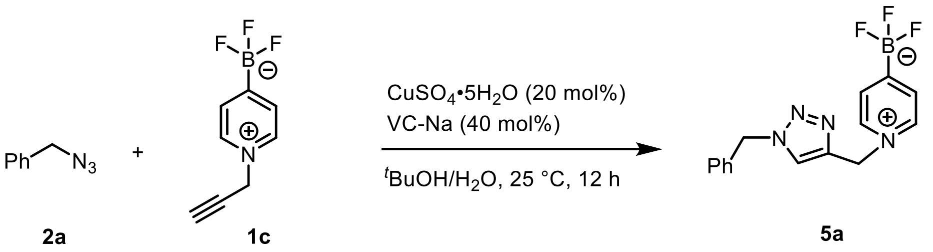

Under an ambient atmosphere, (azidomethyl)benzene (20.0 mg, 0.150 mmol, 1.50 equiv.) in ^{t}BuOH:H_2O (1:1, 0.5 mL, 0.2 M) was to a 4 mL vial containing a magnetic stir bar. Subsequently, $CuSO_4{\cdot}5H_2O$ (5 mg, 20 μmol, 20 mol%), and sodium ascorbate (7.9 mg, 40 μmol, 40 mol%) were added was added into the tube, followed by 4-pyridinium trifluoroborate salt (18.5 mg, 0.100 mmol, 1.00 equiv.). The tube was sealed with a Teflon cap, and the reaction mixture was stirred vigorously at 25 °C. After the indicated time, the reaction vessel was opened to air, and the resulting mixture was concentrated by rotary evaporation. The residue was purified by chromatography on silica gel, eluting with dichloromethane/methanol (from 100:1 to 50:1, v:v) to afford 28 mg (88% yield) of the title compound as a white solid.

R*f* = 0.70 (dichloromethane/methanol, 10:1, v/v (UV))

NMR Spectroscopy:

^{1}H NMR (400 MHz, DMSO-*d*6, 25 °C, δ): 8.80 (d, *J* = 6.2 Hz, 2H), 8.34 (s, 1H), 7.96 (d, *J* = 6.4 Hz, 2H), 7.45 – 7.24 (m, 5H), 5.85 (s, 2H), 5.62 (s, 2H).

^{13}C {^{1}H} NMR (101 MHz, DMSO-*d*6, 25 °C, δ): 141.80, 140.84, 135.61, 130.19 (q, *J* = 1.7 Hz), 128.85, 128.32, 128.11, 124.98, 54.04, 53.07. The carbon directly attached to the boron atom was not detected due to quadrupolar broadening.

^{19}F {^{1}H} NMR (376 MHz, DMSO-*d*6, 25 °C, δ): –142.67 (brs).

^{11}B NMR (128 MHz, DMSO-*d*6, 25 °C, δ): 1.78 (brs).

HRMS ESI (m/z) calc'd for $C_{15}H_{14}N_4BNaF_3^+$ [M+Na]$^+$, 341.1156; found, 341.1160. Deviation: +1.2 ppm.

(1-(2-(1-Benzyl-1*H*-1,2,3-triazol-4-yl)ethyl)pyridin-1-ium-3-yl)trifluoroborate (6a)

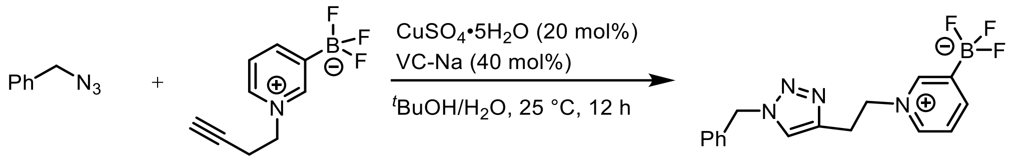

2a **1j** **6a**

Under an ambient atmosphere, (azidomethyl)benzene (59.9 mg, 0.450 mmol, 1.50 equiv.) in ^{t}BuOH:H_2O (1:1, 1.5 mL, 0.2 M) was to a 4 mL vial containing a magnetic stir bar. Subsequently, $CuSO_4{\cdot}5H_2O$ (15 mg, 60 μmol, 20 mol%), and sodium ascorbate (23.8 mg, 120 μmol, 40 mol%) were added was added into the tube, followed by (1-(but-3-yn-1-yl)pyridin-1-ium-3-yl)trifluoroborate (59.7 mg, 0.300 mmol, 1.00 equiv.). The tube was sealed with a Teflon cap, and the reaction mixture was stirred vigorously at 25 °C. After the indicated time, the reaction vessel was opened to air, and the resulting mixture was concentrated by rotary evaporation. The residue was purified by chromatography on silica gel, eluting with dichloromethane/methanol (from 100:1 to 50:1, v:v) to afford 71.9 mg (75% yield) of the title compound as a white solid.

R*f* = 0.55 (dichloromethane/methanol, 10:1, v/v (UV))

NMR Spectroscopy:

^{1}H NMR (400 MHz, CD_3CN, 25 °C, δ): 8.50 (s, 1H), 8.41 (d, *J* = 7.5 Hz, 1H), 8.26 (d, *J* = 6.1 Hz, 1H), 7.67 (t, *J* = 6.8 Hz, 1H), 7.49 (s, 1H), 7.42 – 7.29 (m, 3H), 7.25 – 7.16 (m, 2H), 5.47 (s, 2H), 4.75 (t, *J* = 6.9 Hz, 2H), 3.33 (t, *J* = 6.9 Hz, 2H).

^{13}C {^{1}H} NMR (101 MHz, CD_3CN, 25 °C, δ): 149.46, 146.72, 143.18, 142.31, 136.82, 129.89, 129.30, 128.80, 127.35, 124.06, 60.94, 54.34, 27.93. The carbon directly attached to the boron atom was not detected due to quadrupolar broadening.

^{19}F {^{1}H} NMR (376 MHz, CD_3CN, 25 °C, δ): –143.97 (q, *J* = 45.2 Hz).

^{11}B NMR (128 MHz, CD_3CN, 25 °C, δ): 1.83 (q, *J* = 45.2 Hz).

HRMS ESI (m/z) calc'd for $C_{16}H_{16}BNaN_4F_3^+$ [M+Na]$^+$, 355.1312; found, 355.1316. Deviation: +1.1 ppm.

(1-(2-(1-Benzyl-1*H*-1,2,3-triazol-4-yl)ethyl)-5-chloropyridin-1-ium-3-yl)trifluoroborate (7a)

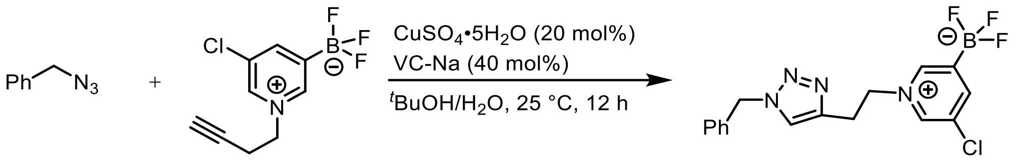

2a 1k 7a

Under an ambient atmosphere, (azidomethyl)benzene (59.9 mg, 0.450 mmol, 1.50 equiv.) in ^{t}BuOH:H_2O (1:1, 1.50 mL, 0.2 M) was to a 4 mL vial containing a magnetic stir bar. Subsequently, $CuSO_4$·$5H_2O$ (15 mg, 60 μmol, 20 mol%), and sodium ascorbate (23.8 mg, 120 μmol, 40 mol%) were added was added into the tube, followed by (1-(but-3-yn-1-yl)-5-chloropyridin-1-ium-3-yl)trifluoroborate (70.2 mg, 0.300 mmol, 1.00 equiv.). The tube was sealed with a Teflon cap, and the reaction mixture was stirred vigorously at 25 °C. After the indicated time, the reaction vessel was opened to air, and the resulting mixture was concentrated by rotary evaporation. The residue was purified by chromatography on silica gel, eluting with dichloromethane/methanol (from 100:1 to 50:1, v:v) to afford 92.8 mg (85% yield) of the title compound as a white solid.

R*f* = 0.60 (dichloromethane/methanol, 10:1, v/v (UV))

NMR Spectroscopy:

^{1}H NMR (400 MHz, DMSO-*d*6, 25 °C, δ): 8.50 (s, 1H), 8.41 (d, *J* = 7.5 Hz, 1H), 8.26 (d, *J* = 6.1 Hz, 1H), 7.67 (t, *J* = 6.8 Hz, 1H), 7.49 (s, 1H), 7.42 – 7.29 (m, 3H), 7.25 – 7.16 (m, 2H), 5.47 (s, 2H), 4.75 (t, *J* = 6.9 Hz, 2H), 3.33 (t, *J* = 6.9 Hz, 2H).

^{13}C {^{1}H} NMR (101 MHz, DMSO-*d*6, 25 °C, δ): 147.01, 145.01, 142.01, 141.14, 136.03, 132.84, 128.78, 128.07, 127.70, 123.41, 59.45, 52.73, 26.76. The carbon directly attached to the boron atom was not detected due to quadrupolar broadening.

^{19}F {^{1}H} NMR (376 MHz, DMSO-d_6, 25 °C, δ): –140.57 (q, J = 41.0 Hz).

^{11}B NMR (128 MHz, DMSO-d_6, 25 °C, δ): 1.65 (q, J = 41.0 Hz).

HRMS ESI (m/z) calc'd for $C_{16}H_{15}BClNaN_4F_3^+$ [M+Na]$^+$, 389.0923; found, 389.0924. Deviation: +0.3 ppm.

(1-(2-(1-Benzyl-1*H*-1,2,3-triazol-4-yl)ethyl)-5-fluoropyridin-1-ium-3-yl)trifluoroborate (8a)

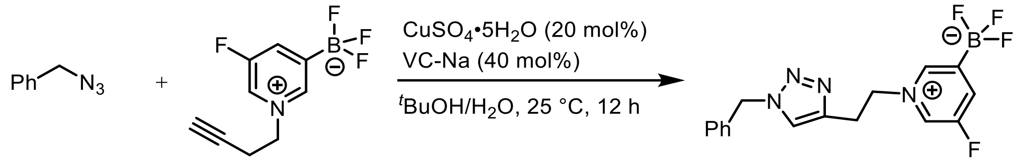

Under an ambient atmosphere, (azidomethyl)benzene (59.9 mg, 0.450 mmol, 1.50 equiv.) in ^{t}BuOH:H_2O (1:1, 1.5 mL, 0.2 M) was to a 4 mL vial containing a magnetic stir bar. Subsequently, $CuSO_4·5H_2O$ (15 mg, 60 μmol, 20 mol%), and sodium ascorbate (23.8 mg, 120 μmol, 40 mol%) were added was added into the tube, followed by (1-(but-3-yn-1-yl)-5-fluoropyridin-1-ium-3-yl)trifluoroborate (65.1 mg, 0.300 mmol, 1.00 equiv.). The tube was sealed with a Teflon cap, and the reaction mixture was stirred vigorously at 25 °C. After the indicated time, the reaction vessel was opened to air, and the resulting mixture was concentrated by rotary evaporation. The residue was purified by chromatography on silica gel, eluting with dichloromethane/methanol (from 100:1 to 50:1, v:v) to afford 82.3 mg (78% yield) of the title compound as a white solid.

R_f = 0.60 (dichloromethane/methanol, 10:1, v/v (UV))

NMR Spectroscopy:

^{1}H NMR (400 MHz, DMSO-d_6, 25 °C, δ): 9.10 (ddd, J = 4.0, 2.5, 1.3 Hz, 1H), 8.64 (s, 1H), 8.23 (dd, J = 7.5, 2.5 Hz, 1H), 7.92 (s, 1H), 7.42 – 7.27 (m, 3H), 7.25 – 7.16 (m, 2H), 5.55 (s, 2H), 4.87 (t, J = 7.2 Hz, 2H), 3.35 (t, J = 7.5 Hz, 2H).

^{13}C {^{1}H} NMR (101 MHz, DMSO-d_6, 25 °C, δ): 159.46 (d, J = 252.4 Hz), 143.28, 142.00, 136.04, 134.36 (d, J = 13.7 Hz), 131.73 (d, J = 38.2 Hz), 128.77, 128.07, 127.70, 123.39, 59.52, 52.74, 26.78. The carbon directly attached to the boron atom was not detected due to quadrupolar broadening.

^{19}F {^{1}H} NMR (376 MHz, DMSO-d_6, 25 °C, δ): –120.37 (1F), –140.55 (q, J = 43.1 Hz, 3F).

^{11}B NMR (128 MHz, DMSO-d_6, 25 °C, δ): 1.61 (q, J = 43.1 Hz).

HRMS ESI (m/z) calc'd for $C_{16}H_{15}BNaN_4F_4^+$ [M+Na]$^+$, 373.1218; found,.373.1220 Deviation: +0.5 ppm.

(1-(2-(1-Benzyl-1*H*-1,2,3-triazol-4-yl)ethyl)pyridin-1-ium-4-yl)trifluoroborate (9a)

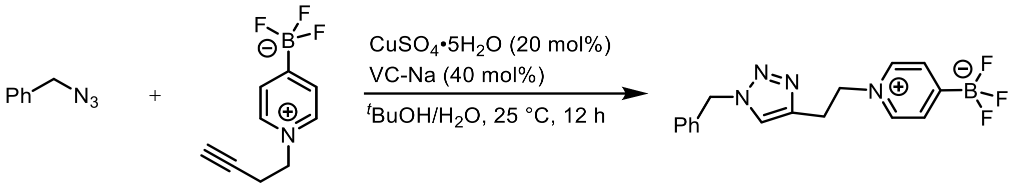

2a **1l** **9a**

Under an ambient atmosphere, (azidomethyl)benzene (59.9 mg, 0.450 mmol, 1.50 equiv.) in ^{t}BuOH:H_2O (1:1, 1.5 mL, 0.2 M) was to a 4 mL vial containing a magnetic stir bar. Subsequently, $CuSO_4{\cdot}5H_2O$ (15 mg, 60 μmol, 20 mol%), and sodium ascorbate (23.8 mg, 120 μmol, 40 mol%) were added was added into the tube, followed by (1-(but-3-yn-1-yl)pyridin-1-ium-4-yl)trifluoroborate (59.7 mg, 0.300 mmol, 1.00 equiv.). The tube was sealed with a Teflon cap, and the reaction mixture was stirred vigorously at 25 °C. After the indicated time, the reaction vessel was opened to air, and the resulting mixture was concentrated by rotary evaporation. The residue was purified by chromatography on silica gel, eluting with dichloromethane/methanol (from 100:1 to 50:1, v:v) to afford 60.1 mg (60% yield) of the title compound as a pale yellow oil.

R*f* = 0.70 (dichloromethane/methanol, 10:1, v/v (UV))

NMR Spectroscopy:

^{1}H NMR (400 MHz, CD_3CN, 25 °C, δ): 8.26 (d, *J* = 6.0 Hz, 2H), 7.89 (d, *J* = 6.0 Hz, 2H), 7.52 (s, 1H), 7.43 – 7.26 (m, 3H), 7.21 – 7.15 (m, 2H), 5.47 (s, 2H), 4.71 (t, *J* = 6.8 Hz, 2H), 3.30 (t, *J* = 6.8 Hz, 2H).

^{13}C {^{1}H} NMR (151 MHz, CD_3CN, 25 °C, δ): 143.09, 142.26, 136.80, 131.16, 129.90, 129.32, 128.70, 124.22, 60.59, 54.35, 27.80. The carbon directly attached to the boron atom was not detected due to quadrupolar broadening.

^{19}F {^{1}H} NMR (376 MHz, CD_3CN, 25 °C, δ): –145.51 (q, *J* = 46.2 Hz).

^{11}B NMR (128 MHz, CD_3CN, 25 °C, δ): 1.70 (q, *J* = 46.2 Hz).

HRMS ESI (m/z) calc'd for $C_{16}H_{16}BNaN_4F_3^+$ $[M+Na]^+$, 355.1312; found, 355.1310. Deviation: –0.6 ppm.

(1-(2-(2-(2-(4-Phenethyl-1*H*-1,2,3-triazol-1-yl)ethoxy)ethoxy)ethyl)pyridin-1-ium-3-yl)trifluoroborate (10a)

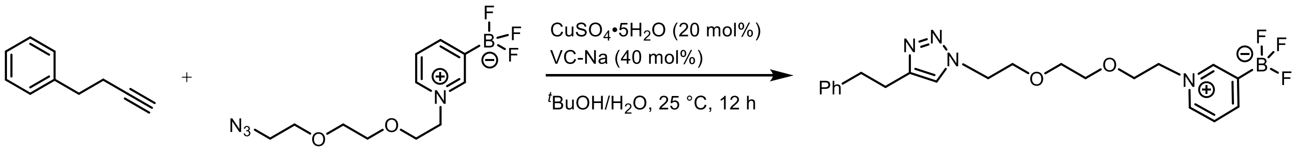

1n **10a**

Under an ambient atmosphere, but-3-yn-1-ylbenzene (58.5 mg, 0.450 mmol, 1.50 equiv.) in ^{t}BuOH:H_2O (1:1, 1.5 mL, 0.2 M) was to a 4 mL vial containing a magnetic stir bar. Subsequently, $CuSO_4{\cdot}5H_2O$ (15 mg, 60 μmol, 20 mol%), and sodium ascorbate (23.8 mg, 120 μmol, 40 mol%) were added was added into the

tube, followed by (1-(2-(2-(2-azidoethoxy)ethoxy)ethyl)pyridin-1-ium-3-yl)trifluoroborate (91.5 mg, 0.300 mmol, 1.00 equiv.). The tube was sealed with a Teflon cap, and the reaction mixture was stirred vigorously at 25 °C. After the indicated time, the reaction vessel was opened to air, and the resulting mixture was concentrated by rotary evaporation. The residue was purified by chromatography on silica gel, eluting with dichloromethane/methanol (from 100:1 to 50:1, v:v) to afford 113 mg (87% yield) of the title compound as a pale yellow oil.

R_f = 0.48 (dichloromethane/methanol, 10:1, v/v (UV))

NMR Spectroscopy:

^{1}H NMR (400 MHz, CD_3CN, 25 °C, δ): 8.56 (s, 1H), 8.47 (d, *J* = 7.5 Hz, 1H), 8.40 (dt, *J* = 6.2, 1.6 Hz, 1H), 7.78 (t, *J* = 6.8 Hz, 1H), 7.54 (brs, 1H), 7.33 – 7.11 (m, 5H), 4.54 (t, *J* = 4.8 Hz, 2H), 4.40 (t, *J* = 5.1 Hz, 2H), 3.82 (t, *J* = 4.9 Hz, 2H), 3.72 (t, *J* = 5.1 Hz, 2H), 3.53 – 3.39 (m, 4H), 2.96 (s, 4H).

^{13}C {^{1}H} NMR (101 MHz, CD_3CN, 25 °C, δ): 149.47, 147.20, 142.77, 142.56, 129.47, 129.31, 127.35, 126.95, 71.20, 70.86, 70.00, 69.98, 61.63, 51.29, 35.71, 28.17. The carbon directly attached to the boron atom was not detected due to quadrupolar broadening. The carbon on triazole ring was not detected due to fast exchange.

^{19}F {^{1}H} NMR (376 MHz, CD_3CN, 25 °C, δ): –143.79 (q, *J* = 45.2 Hz).

^{11}B NMR (128 MHz, CD_3CN, 25 °C, δ): 1.91 (q, *J* = 45.2 Hz).

HRMS ESI (m/z) calc'd for $C_{21}H_{26}BNaN_4O_2F_3^+$ $[M+Na]^+$, 457.1993; found, 457.1995. Deviation: +0.4 ppm.

(1-(2-(1-Benzyl-1*H*-1,2,3-triazol-4-yl)ethyl)quinolin-1-ium-3-yl)trifluoroborate (11a)

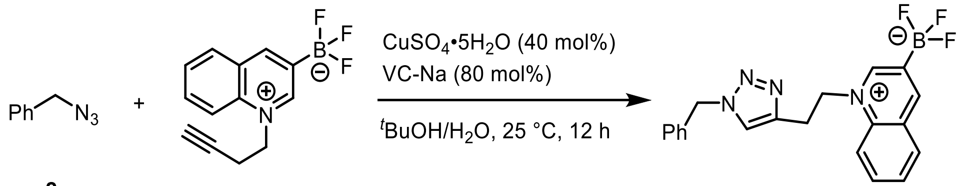

2a **1e** **11a**

Under an ambient atmosphere, (azidomethyl)benzene (59.9 mg, 0.450 mmol, 1.50 equiv.) in $^{t}BuOH:H_2O$ (1:1, 1.5 mL, 0.2 M) was to a 4 mL vial containing a magnetic stir bar. Subsequently, $CuSO_4 \cdot 5H_2O$ (30 mg, 120 µmol, 40 mol%), and sodium ascorbate (47.5 mg, 240 µmol, 80 mol%) were added was added into the tube, followed by (1-(but-3-yn-1-yl)quinolin-1-ium-3-yl)trifluoroborate (74.7 mg, 0.300 mmol, 1.00 equiv.). The tube was sealed with a Teflon cap, and the reaction mixture was stirred vigorously at 25 °C. After the indicated time, the reaction vessel was opened to air, and the resulting mixture was concentrated by rotary evaporation. The residue was purified by chromatography on silica gel, eluting with dichloromethane/methanol (from 100:1 to 50:1, v:v) to afford 106.2 mg (93% yield) of the title compound as a white solid.

R*f* = 0.50 (dichloromethane/methanol, 10:1, v/v (UV))

NMR Spectroscopy:

^{1}H NMR (400 MHz, DMSO-*d*$_6$, 25 °C, δ): 9.09 (s, 1H), 9.04 (s, 1H), 8.45 (d, *J* = 9.0 Hz, 1H), 8.39 (d, *J* = 8.2 Hz, 1H), 8.07 (ddd, *J* = 8.6, 6.7, 1.1 Hz, 1H), 7.91 (s, 1H), 7.88 (d, *J* = 7.5 Hz, 1H), 7.40 – 7.27 (m, 3H), 7.24 – 7.17 (m, 2H), 5.53 (s, 2H), 5.34 (t, *J* = 7.1 Hz, 2H), 3.36 (t, *J* = 8.2 Hz, 2H).

^{13}C {^{1}H} NMR (101 MHz, DMSO-*d*$_6$, 25 °C, δ): 152.08, 149.53, 142.24, 136.35, 135.90, 133.87, 130.21, 129.59, 128.77, 128.75, 128.02, 127.75, 123.50, 118.22, 55.63, 52.73, 25.87. The carbon directly attached to the boron atom was not detected due to quadrupolar broadening.

^{19}F {^{1}H} NMR (376 MHz, CD_3CN, 25 °C, δ): –142.75 (brs).

^{11}B NMR (128 MHz, DMSO-*d*$_6$, 25 °C, δ): 2.25 (brs).

HRMS ESI (m/z) calc'd for $C_{20}H_{18}BNaN_4F_3^+$ [M+Na]$^+$, 405.1469; found, 405.1470. Deviation: +0.2 ppm.

(1-(2-(1-Benzyl-1*H*-1,2,3-triazol-4-yl)ethyl)-5-methoxypyridin-1-ium-3-yl)trifluoroborate (12a)

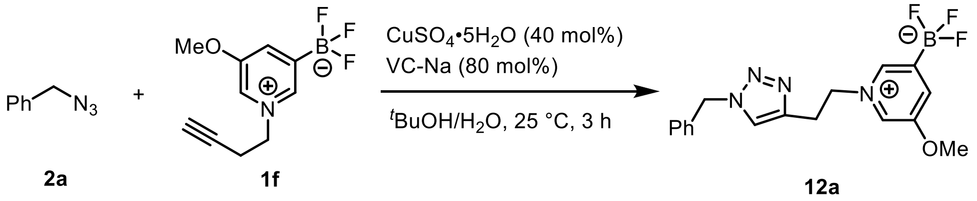

Under an ambient atmosphere, (azidomethyl)benzene (59.9 mg, 0.450 mmol, 1.50 equiv.) in ^{t}BuOH:H_2O (1:1, 1.5 mL, 0.2 M) was to a 4 mL vial containing a magnetic stir bar. Subsequently, $CuSO_4·5H_2O$ (30 mg, 120 μmol, 40 mol%), and sodium ascorbate (47.5 mg, 240 μmol, 80 mol%) were added was added into the tube, followed by (1-(but-3-yn-1-yl)-5-methoxypyridin-1-ium-3-yl)trifluoroborate (68.7 mg, 0.300 mmol, 1.00 equiv.). The tube was sealed with a Teflon cap, and the reaction mixture was stirred vigorously at 25 °C. After the indicated time, the reaction vessel was opened to air, and the resulting mixture was concentrated by rotary evaporation. The residue was purified by chromatography on silica gel, eluting with dichloromethane/methanol (from 100:1 to 50:1, v:v) to afford 80 mg (74% yield) of the title compound as a white solid.

R*f* = 0.50 (dichloromethane/methanol, 10:1, v/v (UV))

NMR Spectroscopy:

^{1}H NMR (400 MHz, DMSO-*d*$_6$, 25 °C, δ): 8.54 (dd, *J* = 2.7, 1.3 Hz, 1H), 8.35 (s, 1H), 7.93 (s, 1H), 7.85 (d, *J* = 2.4 Hz, 1H), 7.41 – 7.30 (m, 3H), 7.25 – 7.20 (m, 2H), 5.56 (s, 2H), 4.82 (t, *J* = 7.2 Hz, 2H), 3.88 (s, 3H), 3.33 (t, *J* = 7.2 Hz, 2H).

^{13}C {^{1}H} NMR (101 MHz, DMSO-*d*$_6$, 25 °C, δ): 157.07, 142.19, 138.96, 136.04, 132.12, 129.28, 128.78, 128.07, 127.72, 123.35, 59.29, 56.66, 52.75, 27.06. The carbon directly attached to the boron atom was not detected due to quadrupolar broadening.

^{19}F {^{1}H} NMR (376 MHz, CD_3CN, 25 °C, δ): –143.78 (q, *J* = 45.2 Hz).

^{11}B NMR (128 MHz, CD_3CN, 25 °C, δ): 1.78 (q, *J* = 45.2 Hz).

HRMS ESI (m/z) calc'd for $C_{17}H_{18}BNaN_4OF_3^+$ [M+Na]$^+$, 385.1418; found, 385.1421. Deviation: +0.8 ppm.

(1-(2-(1-Benzyl-1*H*-1,2,3-triazol-4-yl)ethyl)-3-fluoropyridin-1-ium-4-yl)trifluoroborate (13a)

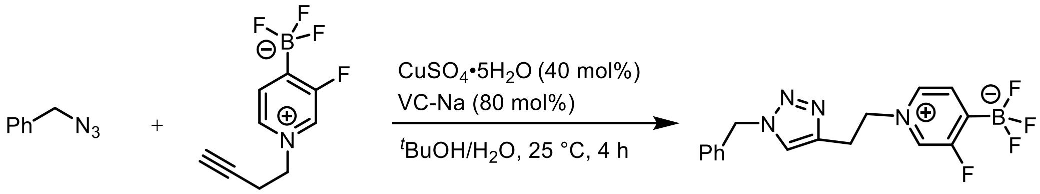

2a **1g** **13a**

Under an ambient atmosphere, (azidomethyl)benzene (59.9 mg, 0.450 mmol, 1.50 equiv.) in ^{t}BuOH:H_2O (1:1, 1.5 mL, 0.2 M) was to a 4 mL vial containing a magnetic stir bar. Subsequently, $CuSO_4·5H_2O$ (30 mg, 120 μmol, 40 mol%), and sodium ascorbate (47.5 mg, 240 μmol, 80 mol%) were added was added into the tube, followed by (1-(but-3-yn-1-yl)-3-fluoropyridin-1-ium-4-yl)trifluoroborate (65.1 mg, 0.300 mmol, 1.00 equiv.). The tube was sealed with a Teflon cap, and the reaction mixture was stirred vigorously at 25 °C. After the indicated time, the reaction vessel was opened to air, and the resulting mixture was concentrated by rotary evaporation. The residue was purified by chromatography on silica gel, eluting with dichloromethane/methanol (from 100:1 to 50:1, v:v) to afford 93.5 mg (89% yield) of the title compound as a white solid.

R_f = 0.60 (dichloromethane/methanol, 10:1, v/v (UV))

NMR Spectroscopy:

^{1}H NMR (400 MHz, DMSO-*d*$_6$, 25 °C, δ): 8.98 (d, *J* = 3.4 Hz, 1H), 8.58 (d, *J* = 5.8 Hz, 1H), 7.93 (s, 1H), 7.87 (t, *J* = 5.7 Hz, 1H), 7.41 – 7.29 (m, 3H), 7.21 – 7.15 (m, 2H), 5.56 (s, 2H), 4.82 (t, *J* = 7.0 Hz, 2H), 3.36 (t, *J* = 7.0 Hz, 2H).

^{13}C {^{1}H} NMR (101 MHz, DMSO-*d*$_6$, 25 °C, δ): 162.71 (d, *J* = 246.6 Hz), 141.95, 139.43 (d, *J* = 3.0 Hz), 136.08, 131.95 (d, *J* = 43.3 Hz), 131.85 (dq, *J* = 13.5, 2.0 Hz), 128.76, 128.09, 127.59, 123.44, 59.24, 52.72, 26.46. The carbon directly attached to the boron atom was not detected due to quadrupolar broadening.

^{19}F {^{1}H} NMR (376 MHz, DMSO-*d*$_6$, 25 °C, δ): –113.26 (1F), –139.70 (brs, 3F).

^{11}B NMR (128 MHz, DMSO-*d*$_6$, 25 °C, δ): 1.22 (brs).

HRMS ESI (m/z) calc'd for $C_{16}H_{15}BNaN_4F_4^+$ [M+Na]$^+$, 373.1218; found, 373.1219. Deviation: +0.3 ppm.

(1-(3-(Benzoyloxy)propyl)-3-chloropyridin-1-ium-4-yl)trifluoroborate (14a)

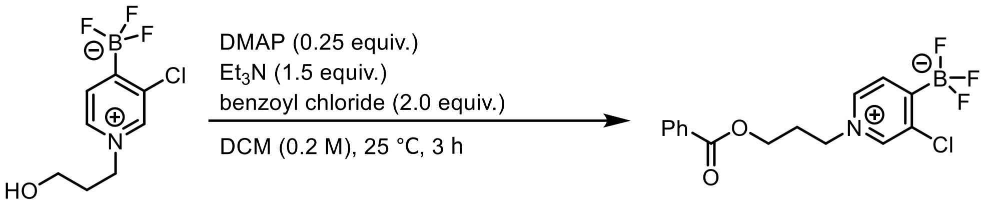

1h **14a**

Under an ambient atmosphere, (3-chloro-1-(3-hydroxypropyl)pyridin-1-ium-4-yl)trifluoroborate (239 mg, 1.00 mmol, 1.00 equiv.), DMAP (30.5 mg, 0.250 mmol, 0.250 equiv.), Et_3N (0.210 mL, 152 mg, 1.50 mmol, 1.50 equiv.) and DCM (5 mL, *c* = 0.2 M) were added to a 20-mL vial, followed by benzoyl chloride (282 mg, 2.00 mmol, 2.00 equiv.). The reaction was stirred at 25 °C for 12 h. The reaction was quenched with 10 mL water, the layers were separated, and the aqueous laver was extracted with DCM (3 × 20 mL). Then the combined organic lavers were dried over Na_2SO_4, and the solvent was concentrated by rotary evaporation. The residue was purified by chromatography on silica gel, eluting with dichloromethane/methanol (from 100:1 to 50:1, v:v) to afford 228 mg (66% yield) of the title compound as a white solid.

$\mathbf{R}_f$ = 0.50 (dichloromethane/methanol, 10:1, v/v (UV))

NMR Spectroscopy:

^{1}H NMR (400 MHz, CD_3CN, 25 °C, δ): 8.63 (s, 1H), 8.45 (d, *J* = 5.9 Hz, 1H), 8.04 (d, *J* = 5.9 Hz, 1H), 7.94 – 7.85 (m, 2H), 7.61 (t, *J* = 7.4 Hz, 1H), 7.48 (t, *J* = 7.7 Hz, 2H), 4.62 (t, *J* = 7.2 Hz, 2H), 4.37 (t, *J* = 5.7 Hz, 2H), 2.43 (p, *J* = 6.8 Hz, 2H).

^{13}C {^{1}H} NMR (101 MHz, CD_3CN, 25 °C, δ): 166.91, 142.47, 141.04, 140.03, 134.23, 132.83 (q, *J* = 2.8 Hz), 130.73, 130.26, 129.56, 62.54, 59.48, 30.66. The carbon directly attached to the boron atom was not detected due to quadrupolar broadening.

^{19}F {^{1}H} NMR (376 MHz, CD_3CN, 25 °C, δ): –143.84 (brs).

^{11}B NMR (128 MHz, CD_3CN, 25 °C, δ): 1.30 (q, *J* = 42.9 Hz).

HRMS ESI (m/z) calc'd for $C_{15}H_{14}NClBNaO_2F_3^+$ $[M+Na]^+$, 366.0650; found,.366.0649. Deviation: –0.3 ppm.

(1-(2-(1-Benzyl-1*H*-1,2,3-triazol-4-yl)ethyl)-2-methylpyridin-1-ium-4-yl)trifluoroborate (15a)

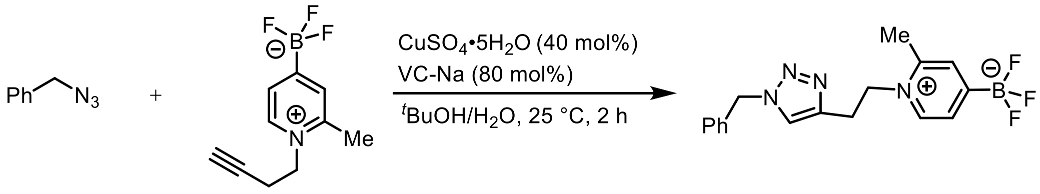

2a **1i** **15a**

Under an ambient atmosphere, (azidomethyl)benzene (59.9 mg, 0.450 mmol, 1.50 equiv.) in ^{t}BuOH:H_2O (1:1, 1.50 mL, 0.2 M) was to a 4 mL vial containing a magnetic stir bar. Subsequently, $CuSO_4{\cdot}5H_2O$ (30 mg, 120 μmol, 40 mol%), and sodium ascorbate (47.5 mg, 240 μmol, 80 mol%) were added was added into the

tube, followed by (1-(but-3-yn-1-yl)-2-methylpyridin-1-ium-4-yl)trifluoroborate (63.9 mg, 0.300 mmol, 1.00 equiv.). The tube was sealed with a Teflon cap, and the reaction mixture was stirred vigorously at 25 °C. After the indicated time, the reaction vessel was opened to air, and the resulting mixture was concentrated by rotary evaporation. The residue was purified by chromatography on silica gel, eluting with dichloromethane/methanol (from 100:1 to 50:1, v:v) to afford 87.7 mg (84% yield) of the title compound as a white solid.

R*f* = 0.50 (dichloromethane/methanol, 10:1, v/v (UV))

NMR Spectroscopy:

^{1}H NMR (400 MHz, DMSO-*d*6, 25 °C, δ): 8.46 (d, *J* = 6.0 Hz, 1H), 7.92 (s, 1H), 7.79 (s, 1H), 7.63 (d, *J* = 5.9 Hz, 1H), 7.42 – 7.28 (m, 3H), 7.24 – 7.17 (m, 2H), 5.56 (s, 2H), 4.73 (t, *J* = 7.3 Hz, 2H), 3.26 (t, *J* = 7.3 Hz, 2H), 2.69 (s, 3H).

^{13}C {^{1}H} NMR (151 MHz, DMSO-*d*6, 25 °C, δ): 151.55, 142.32, 142.05, 136.06, 131.94, 128.76, 128.10, 127.68, 127.50, 123.49, 55.32, 52.73, 25.47, 19.03. The carbon directly attached to the boron atom was not detected due to quadrupolar broadening.

^{19}F {^{1}H} NMR (376 MHz, DMSO-*d*6, 25 °C, δ): –142.43 (brs).

^{11}B NMR (128 MHz, DMSO-*d*6, 25 °C, δ): 1.83 (brs).

HRMS ESI (m/z) calc'd for $C_{17}H_{18}BNaN_4F_3^+$ [M+Na]$^+$, 369.1469; found, 369.1467. Deviation: –0.5 ppm.

(1-(2-(1-Benzyl-1*H*-1,2,3-triazol-4-yl)ethyl)-6-methylpyridin-1-ium-3-yl)trifluoroborate (16a)

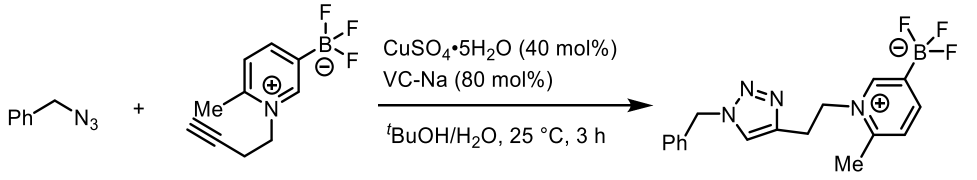

2a 1d 16a

Under an ambient atmosphere, (azidomethyl)benzene (59.9 mg, 0.450 mmol, 1.50 equiv.) in ^{t}BuOH:H_2O (1:1, 1.50 mL, 0.2 M) was to a 4 mL vial containing a magnetic stir bar. Subsequently, $CuSO_4\cdot5H_2O$ (30 mg, 120 µmol, 40 mol%), and sodium ascorbate (47.5 mg, 240 µmol, 80 mol%) were added was added into the tube, followed by (1-(but-3-yn-1-yl)-6-methylpyridin-1-ium-3-yl)trifluoroborate (63.9 mg, 0.300 mmol, 1.00 equiv.). The tube was sealed with a Teflon cap, and the reaction mixture was stirred vigorously at 25 °C. After the indicated time, the reaction vessel was opened to air, and the resulting mixture was concentrated by rotary evaporation. The residue was purified by chromatography on silica gel, eluting with dichloromethane/methanol (from 100:1 to 50:1, v:v) to afford 96.8 mg (93% yield) of the title compound as a white solid.

R*f* = 0.50 (dichloromethane/methanol, 10:1, v/v (UV))

NMR Spectroscopy:

^{1}H NMR (400 MHz, CD_3CN, 25 °C, δ): 8.34 (s, 1H), 8.26 (d, *J* = 7.8 Hz, 1H), 7.58 – 7.50 (m, 2H), 7.41 – 7.28 (m, 3H), 7.27 – 7.19 (m, 2H), 5.48 (s, 2H), 4.69 (t, *J* = 7.2 Hz, 2H), 3.27 (t, *J* = 7.2 Hz, 2H), 2.58 (s, 3H).

^{13}C {^{1}H} NMR (101 MHz, DMSO-*d*$_6$, 25 °C, δ): 151.53, 148.12, 146.43, 142.21, 135.98, 128.77, 128.31, 128.08, 127.81, 123.45, 55.45, 52.77, 25.90, 19.08. The carbon directly attached to the boron atom was not detected due to quadrupolar broadening.

^{19}F {^{1}H} NMR (376 MHz, CD_3CN, 25 °C, δ): –143.58 (q, *J* = 46.1 Hz).

^{11}B NMR (128 MHz, CD_3CN, 25 °C, δ): 1.92 (q, *J* = 46.1 Hz).

HRMS ESI (m/z) calc'd for $C_{17}H_{18}BNaN_4F_3^+$ [M+Na]$^+$, 369.1469; found,.369.1467. Deviation: –0.5 ppm.

(1-((1-(4-Fluorophenyl)-1*H*-1,2,3-triazol-4-yl)methyl)pyridin-1-ium-2-yl)trifluoroborate (3b)

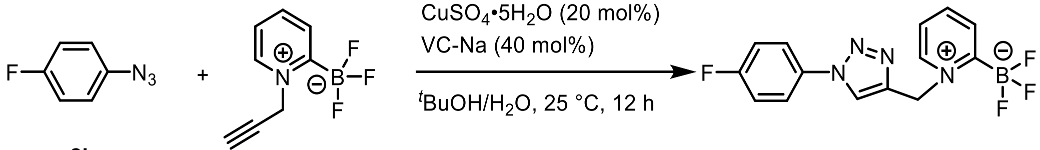

Under an ambient atmosphere, 1-azido-4-fluorobenzene (61.7 mg, 0.450 mmol, 1.50 equiv.) in ^{t}BuOH:H_2O (1:1, 1.5 mL, 0.2 M) was to a 4 mL vial containing a magnetic stir bar. Subsequently, $CuSO_4·5H_2O$ (15 mg, 60 µmol, 20 mol%), and sodium ascorbate (23.8 mg, 120 µmol, 40 mol%) were added was added into the tube, followed by 2-pyridinium trifluoroborate salt (55.5 mg, 0.300 mmol, 1.00 equiv.). The tube was sealed with a Teflon cap, and the reaction mixture was stirred vigorously at 25 °C. After the indicated time, the reaction vessel was opened to air, and the resulting mixture was quenched with saturated $NaHCO_3$ (10 mL), then extracted into EtOAc (3 × 10 mL). The combined organic phase was washed with brine, dried over Na_2SO_4, and concentrated by rotary evaporation. The residue was purified by chromatography on silica gel, eluting with hexanes/ethyl acetate (from 10:1 to 1:1, v:v) to afford 66.5 mg (69% yield) of the title compound as a pale yellow solid.

R*f* = 0.50 (ethyl acetate, (UV))

NMR Spectroscopy:

^{1}H NMR (400 MHz, DMSO-*d*$_6$, 25 °C, δ): 9.00 (d, *J* = 6.3 Hz, 1H), 8.74 (s, 1H), 8.38 (t, *J* = 7.7 Hz, 1H), 8.06 (d, *J* = 7.0 Hz, 1H), 7.98 – 7.90 (m, 3H), 7.48 – 7.40 (m, 2H), 6.06 (s, 2H).

^{13}C {^{1}H} NMR (101 MHz, DMSO-*d*$_6$, 25 °C, δ): 161.79 (d, *J* = 245.9 Hz), 145.37, 143.25, 142.53, 132.90 (q, *J* = 2.7 Hz), 131.28, 125.94, 123.19, 122.71 (d, *J* = 8.9 Hz), 116.75 (d, *J* = 23.3 Hz), 53.16 (q, *J* = 3.7 Hz). The carbon directly attached to the boron atom was not detected due to quadrupolar broadening.

^{19}F {^{1}H} NMR (376 MHz, $CDCl_3$, 25 °C, δ): –111.19 (1F), –142.10 (q, *J* = 36.4 Hz, 3F).

^{11}B NMR (128 MHz, $CDCl_3$, 25 °C, δ): 0.84 (q, *J* = 36.4 Hz).

HRMS ESI (m/z) calc'd for $C_{14}H_{11}N_4BNaF_4^+$ $[M+Na]^+$, 345.0905; found, 345.0908. Deviation: +0.9 ppm.

(1-((1-(2-Acetyl-4-fluorophenyl)-1*H*-1,2,3-triazol-4-yl)methyl)pyridin-1-ium-4-yl)trifluoroborate (5c)

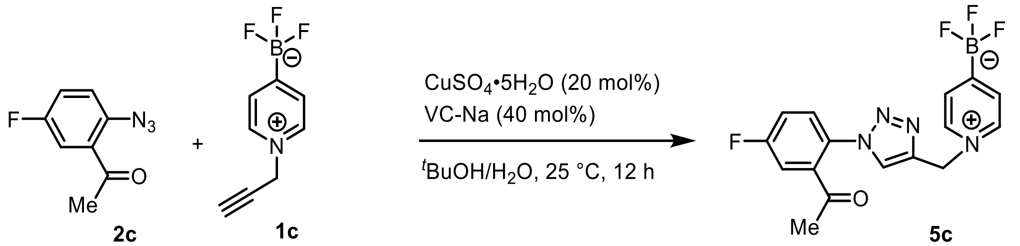

Under an ambient atmosphere, 1-(2-azido-5-fluorophenyl)ethanone (80.6 mg, 0.450 mmol, 1.50 equiv.) in ^{t}BuOH:H_2O (1:1, 1.5 mL, 0.2 M) was to a 4 mL vial containing a magnetic stir bar. Subsequently, $CuSO_4 \cdot 5H_2O$ (15 mg, 60 μmol, 20 mol%), and sodium ascorbate (23.8 mg, 120 μmol, 40 mol%) were added was added into the tube, followed by 4-pyridinium trifluoroborate salt (55.5 mg, 0.300 mmol, 1.00 equiv.). The tube was sealed with a Teflon cap, and the reaction mixture was stirred vigorously at 25 °C. After the indicated time, the reaction vessel was opened to air, and the resulting mixture was concentrated by rotary evaporation. The residue was purified by chromatography on silica gel, eluting with dichloromethane/methanol (from 100:1 to 50:1, v:v) to afford 79.1 mg (72% yield) of the title compound as a pale yellow solid.

R_f = 0.50 (dichloromethane/methanol, 10:1, v/v (UV))

NMR Spectroscopy:

^{1}H NMR (400 MHz, DMSO-*d*$_6$, 25 °C, δ): 8.86 (d, *J* = 6.2 Hz, 2H), 8.75 (s, 1H), 7.99 (d, *J* = 6.5 Hz, 2H), 7.81 (dd, *J* = 8.8, 2.8 Hz, 1H), 7.76 (dd, *J* = 8.8, 4.9 Hz, 1H), 7.65 (td, *J* = 8.4, 2.9 Hz, 1H), 5.98 (s, 2H), 2.28 (s, 3H).

^{13}C {^{1}H} NMR (101 MHz, DMSO-*d*$_6$, 25 °C, δ): 198.13 (d, *J* = 1.0 Hz), 162.07 (d, *J* = 249.7 Hz), 141.84, 141.08, 137.47 (d, *J* = 7.2 Hz), 130.23 (d, *J* = 1.8 Hz), 129.87 (d, *J* = 3.3 Hz), 128.68 (d, *J* = 8.9 Hz), 126.68, 119.03 (d, *J* = 23.0 Hz), 116.23 (d, *J* = 24.7 Hz), 53.78, 29.20. The carbon directly attached to the boron atom was not detected due to quadrupolar broadening.

^{19}F {^{1}H} NMR (376 MHz, DMSO-*d*$_6$, 25 °C, δ): –109.88 (1F), –142.60 (brs, 3F).

^{11}B NMR (128 MHz, DMSO-*d*$_6$, 25 °C, δ): 1.79 (brs).

HRMS ESI (m/z) calc'd for $C_{16}H_{13}N_4BNaOF_4^+$ $[M+Na]^+$, 387.1011; found, 387.1010. Deviation: –0.3 ppm.

(1-((1-(4-(2-Hydroxyethyl)phenyl)-1*H*-1,2,3-triazol-4-yl)methyl)pyridin-1-ium-2-yl)trifluoroborate (3d)

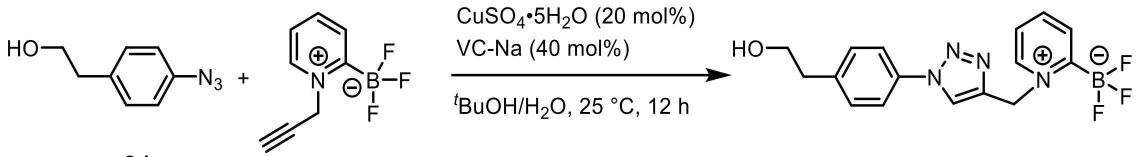

Under an ambient atmosphere, 2-(4-azidophenyl)ethanol (73.4 mg, 0.450 mmol, 1.50 equiv.) in ^{t}BuOH:H_2O (1:1, 1.5 mL, 0.2 M) was to a 4 mL vial containing a magnetic stir bar. Subsequently, $CuSO_4·5H_2O$ (15 mg, 60 μmol, 20 mol%), and sodium ascorbate (23.8 mg, 120 μmol, 40 mol%) were added was added into the tube, followed by 2-pyridinium trifluoroborate salt (55.5 mg, 0.300 mmol, 1.00 equiv.). The tube was sealed with a Teflon cap, and the reaction mixture was stirred vigorously at 25 °C. After the indicated time, the reaction vessel was opened to air, and the resulting mixture was quenched with saturated $NaHCO_3$ (10 mL), then extracted into EtOAc (3 × 10 mL). The combined organic phase was washed with brine, dried over Na_2SO_4, and concentrated by rotary evaporation. The residue was purified by chromatography on silica gel, eluting with dichloromethane/methanol (from 100:1 to 50:1, v:v) to afford 80.4 mg (77% yield) of the title compound as a white solid.

R*f* = 0.40 (dichloromethane/methanol, 10:1, v/v (UV))

NMR Spectroscopy:

^{1}H NMR (400 MHz, CD_3CN, 25 °C, δ): 8.80 (d, *J* = 6.2 Hz, 1H), 8.35 (s, 1H), 8.23 (t, *J* = 7.7 Hz, 1H), 8.12 (d, *J* = 7.9 Hz, 1H), 7.75 (ddd, *J* = 7.9, 6.3, 1.8 Hz, 1H), 7.67 (d, *J* = 8.5 Hz, 2H), 7.41 (d, *J* = 8.5 Hz, 2H), 6.02 (s, 2H), 3.74 (q, *J* = 6.6 Hz, 2H), 2.84 (t, *J* = 6.6 Hz, 2H), 2.74 (t, *J* = 5.5 Hz, 1H).

^{13}C {^{1}H} NMR (101 MHz, DMSO-*d*$_6$, 25 °C, δ): 145.84, 143.69, 142.85, 141.32, 134.93, 131.73 (q, *J* = 2.6 Hz), 130.72, 126.40, 123.26, 120.56, 62.17 (d, *J* = 12.0 Hz), 53.66 (q, *J* = 3.6 Hz), 38.78 (d, *J* = 5.4 Hz).The carbon directly attached to the boron atom was not detected due to quadrupolar broadening.

^{19}F {^{1}H} NMR (376 MHz, CD_3CN, 25 °C, δ): –141.84 (q, *J* = 40.5 Hz).

^{11}B NMR (128 MHz, CD_3CN, 25 °C, δ): 0.81 (q, *J* = 40.5 Hz).

HRMS ESI (m/z) calc'd for $C_{16}H_{16}N_4BNaOF_3^+$ $[M+Na]^+$, 371.1261; found, 371.1257. Deviation: –1.1 ppm.

(1-((1-(4-(Ethoxycarbonyl)phenyl)-1*H*-1,2,3-triazol-4-yl)methyl)pyridin-1-ium-3-yl)trifluoroborate (4e)

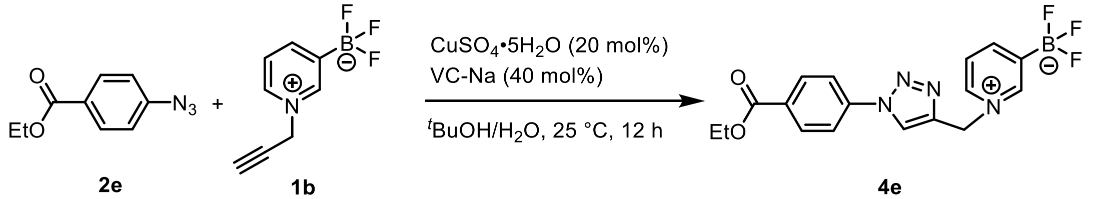

Under an ambient atmosphere, ethyl 4-azidobenzoate (86.0 mg, 0.450 mmol, 1.50 equiv.) in ^{t}BuOH:H_2O (1:1, 1.5 mL, 0.2 M) was to a 4 mL vial containing a magnetic stir bar. Subsequently, $CuSO_4·5H_2O$ (15 mg, 60 μmol, 20 mol%), and sodium ascorbate (23.8 mg, 120 μmol, 40 mol%) were added was added into the

tube, followed by 3-pyridinium trifluoroborate salt (55.5 mg, 0.300 mmol, 1.00 equiv.). The tube was sealed with a Teflon cap, and the reaction mixture was stirred vigorously at 25 °C. After the indicated time, the reaction vessel was opened to air, and the resulting mixture was concentrated by rotary evaporation. The residue was purified by chromatography on silica gel, eluting with dichloromethane/methanol (from 100:1 to 50:1, v:v) to afford 67.2 mg (60% yield) of the title compound as a white solid.

R*f* = 0.60 (dichloromethane/methanol, 10:1, v/v (UV))

NMR Spectroscopy:

^{1}**H NMR** (400 MHz, DMSO-*d*$_6$, 25 °C, δ): 9.11 (s, 1H), 8.92 (dt, *J* = 6.1, 1.6 Hz, 1H), 8.88 (s, 1H), 8.43 (dt, *J* = 7.6, 1.3 Hz, 1H), 8.21 – 8.13 (m, 2H), 8.10 – 8.04 (m, 2H), 7.97 (d, *J* = 6.8 Hz, 1H), 6.06 (s, 2H), 4.35 (q, *J* = 7.1 Hz, 2H), 1.34 (t, *J* = 7.1 Hz, 2H).

^{13}**C {^{1}H} NMR** (101 MHz, DMSO-*d*$_6$, 25 °C, δ): 164.78, 148.53, 145.90 (d, *J* = 2.3 Hz), 142.11, 141.97, 139.45, 130.96, 129.99, 126.95, 123.61, 120.23, 61.15, 54.06, 14.12. The carbon directly attached to the boron atom was not detected due to quadrupolar broadening.

^{19}**F {^{1}H} NMR** (376 MHz, DMSO-*d*$_6$, 25 °C, δ): –140.45 (brs).

^{11}**B NMR** (128 MHz, DMSO-*d*$_6$, 25 °C, δ): 2.03 (brs).

HRMS ESI (m/z) calc'd for $C_{17}H_{16}N_4BNaO_2F_3^+$ [M+Na]$^+$, 399.1211; found, 399.1209. Deviation: –0.5 ppm.

(1-((1-(2-Formylphenyl)-1*H*-1,2,3-triazol-4-yl)methyl)pyridin-1-ium-2-yl)trifluoroborate (3f)

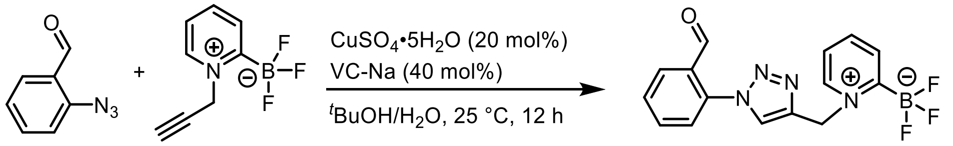

2f **1a** **3f**

Under an ambient atmosphere, 2-azidobenzaldehyde (66.2 mg, 0.450 mmol, 1.50 equiv.) in ^{t}BuOH:H_2O (1:1, 1.5 mL, 0.2 M) was to a 4 mL vial containing a magnetic stir bar. Subsequently, $CuSO_4·5H_2O$ (15 mg, 60 μmol, 20 mol%), and sodium ascorbate (23.8 mg, 120 μmol, 40 mol%) were added was added into the tube, followed by 2-pyridinium trifluoroborate salt (55.5 mg, 0.300 mmol, 1.00 equiv.). The tube was sealed with a Teflon cap, and the reaction mixture was stirred vigorously at 25 °C. After the indicated time, the reaction vessel was opened to air, and the resulting mixture was quenched with saturated $NaHCO_3$ (10 mL), then extracted into EtOAc (3 × 10 mL). The combined organic phase was washed with brine, dried over Na_2SO_4, and concentrated by rotary evaporation. The residue was purified by chromatography on silica gel, eluting with dichloromethane/methanol (from 200:1 to 100:1, v:v) to afford 61.8 mg (62% yield) of the title compound as a pale yellow solid.

R*f* = 0.45 (dichloromethane/methanol, 100:1, v/v (UV))

NMR Spectroscopy:

^{1}**H NMR** (400 MHz, DMSO-*d*$_6$, 25 °C, δ): 9.74 (s, 1H), 9.03 (d, *J* = 6.3 Hz, 1H), 8.75 (s, 1H), 8.38 (t, *J* =

7.7 Hz, 1H), 8.08 (dd, J = 7.9, 1.7 Hz, 1H), 8.03 – 7.94 (m, 2H), 7.89 (td, J = 7.7, 1.6 Hz, 1H), 7.80 – 7.71 (m, 2H), 6.11 (s, 2H).

^{13}C {^{1}H} NMR (101 MHz, DMSO-d_6, 25 °C, δ): 189.40, 145.34, 143.29, 142.06, 137.26, 134.95, 131.33 (q, J = 2.8 Hz), 130.33, 129.94, 129.13, 127.07 (q, J = 2.2 Hz), 126.00, 125.95, 53.11 (q, J = 3.2 Hz). The carbon directly attached to the boron atom was not detected due to quadrupolar broadening.

^{19}F {^{1}H} NMR (376 MHz, DMSO-d_6, 25 °C, δ): –139.23 (q, J = 32.2 Hz).

^{11}B NMR (128 MHz, DMSO-d_6, 25 °C, δ): 0.77 (q, J = 32.9 Hz).

HRMS ESI (m/z) calc'd for $C_{15}H_{12}BF_3N_4NaO^+$ [M+Na]$^+$, 355.0948; found, 355.0951. Deviation: +0.8 ppm.

(1-((1-(4-(Methylthio)phenyl)-1*H*-1,2,3-triazol-4-yl)methyl)pyridin-1-ium-2-yl)trifluoroborate (3g)

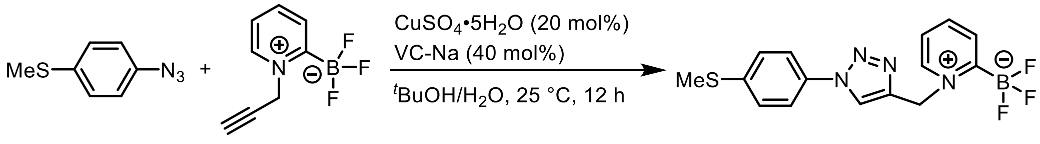

Under an ambient atmosphere, (4-azidophenyl)(methyl)sulfane (74.3 mg, 0.450 mmol, 1.50 equiv.) in ^{t}BuOH:H_2O (1:1, 1.5 mL, 0.2 M) was to a 4 mL vial containing a magnetic stir bar. Subsequently, $CuSO_4·5H_2O$ (15 mg, 60 μmol, 20 mol%), and sodium ascorbate (23.8 mg, 120 μmol, 40 mol%) were added was added into the tube, followed by 2-pyridinium trifluoroborate salt (55.5 mg, 0.300 mmol, 1.00 equiv.). The tube was sealed with a Teflon cap, and the reaction mixture was stirred vigorously at 25 °C. After the indicated time, the reaction vessel was opened to air, and the resulting mixture was quenched with saturated $NaHCO_3$ (10 mL), then extracted into EtOAc (3 × 10 mL). The combined organic phase was washed with brine, dried over Na_2SO_4, and concentrated by rotary evaporation. The residue was purified by chromatography on silica gel, eluting with dichloromethane/methanol (from 200:1 to 50:1, v:v) to afford 66.0 mg (63% yield) of the title compound as a pale yellow solid.

R_f = 0.80 (dichloromethane/methanol, 10:1, v/v (UV))

NMR Spectroscopy:

^{1}H NMR (400 MHz, DMSO-d_6, 25 °C, δ): 8.95 (d, J = 6.3 Hz, 1H), 8.67 (s, 1H), 8.31 (t, J = 7.7 Hz, 1H), 8.01 (d, J = 7.9 Hz, 1H), 7.89 (ddd, J = 7.8, 6.2, 1.8 Hz, 1H), 7.75 (d, J = 8.4 Hz, 2H), 7.37 (d, J = 8.4 Hz, 2H), 6.01 (s, 2H), 2.46 (brs, 3H).

^{13}C {^{1}H} NMR (101 MHz, DMSO-d_6, 25 °C, δ): 145.38, 143.25, 142.46, 139.58, 133.32, 131.31 (q, J = 2.6 Hz), 126.68, 125.96, 122.76 (q, J = 2.0 Hz), 120.78, 53.22 (q, J = 3.0 Hz), 14.55. The carbon directly attached to the boron atom was not detected due to quadrupolar broadening.

^{19}F {^{1}H} NMR (376 MHz, CD_3CN, 25 °C, δ): –141.85 (q, J = 40.2 Hz).

^{11}B NMR (128 MHz, DMSO-d_6, 25 °C, δ): 0.79 (q, J = 40.2 Hz).

HRMS ESI (m/z) calc'd for $C_{15}H_{14}N_4BNaSF_3^+$ [M+Na]$^+$, 373.0877; found, 373.0883. Deviation: +1.6 ppm.

(1-((1-(4-Cyanophenyl)-1*H*-1,2,3-triazol-4-yl)methyl)pyridin-1-ium-2-yl)trifluoroborate (3h)

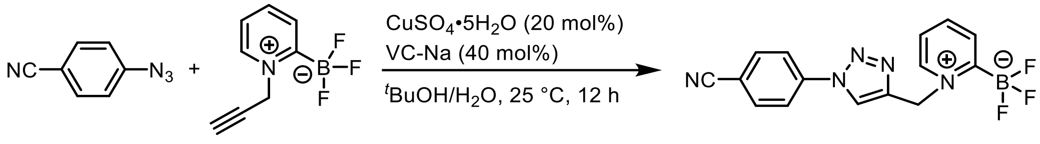

2h 1a 3h

Under an ambient atmosphere, 4-azidobenzonitrile (64.9 mg, 0.450 mmol, 1.50 equiv.) in ^{t}BuOH:H_2O (1:1, 1.5 mL, 0.2 M) was to a 4 mL vial containing a magnetic stir bar. Subsequently, $CuSO_4{\cdot}5H_2O$ (15 mg, 60 μmol, 20 mol%), and sodium ascorbate (23.8 mg, 120 μmol, 40 mol%) were added was added into the tube, followed by 2-pyridinium trifluoroborate salt (55.5 mg, 0.300 mmol, 1.00 equiv.). The tube was sealed with a Teflon cap, and the reaction mixture was stirred vigorously at 25 °C. After the indicated time, the reaction vessel was opened to air, and the resulting mixture was quenched with saturated $NaHCO_3$ (10 mL), then extracted into EtOAc (3 × 10 mL). The combined organic phase was washed with brine, dried over Na_2SO_4, and concentrated by rotary evaporation. The residue was purified by chromatography on silica gel, eluting with dichloromethane/methanol (from 200:1 to 100:1, v:v) to afford 77.5 mg (52% yield) of the title compound as a white solid.

R*f* = 0.30 (dichloromethane/methanol, 100:1, v/v (UV))

NMR Spectroscopy:

^{1}H NMR (400 MHz, DMSO-*d*6, 25 °C, δ): 9.01 (d, *J* = 6.2 Hz, 1H), 8.92 (s, 1H), 8.38 (t, *J* = 7.7 Hz, 1H), 8.14 (d, *J* = 8.9 Hz, 2H), 8.13 – 8.03 (m, 3H), 7.96 (ddd, *J* = 7.9, 6.2, 1.8 Hz, 1H), 6.08 (s, 2H).

^{13}C {^{1}H} NMR (101 MHz, DMSO-*d*6, 25 °C, δ): 145.44, 143.33, 143.05, 139.26, 134.30, 131.30 (q, *J* = 2.7 Hz), 125.97, 123.17, 120.74, 118.04, 111.36, 53.15 (q, *J* = 3.0.Hz). The carbon directly attached to the boron atom was not detected due to quadrupolar broadening.

^{19}F {^{1}H} NMR (376 MHz, DMSO-*d*6, 25 °C, δ): –139.27 (q, *J* = 36.2 Hz).

^{11}B NMR (128 MHz, DMSO-*d*6, 25 °C, δ): 0.74 (q, *J* = 36.2 Hz).

HRMS ESI (m/z) calc'd for $C_{15}H_{11}BF_3NaN_5^+$ [M+Na]$^+$, 352.0952; found, 352.0955. Deviation: +0.9 ppm.

(1-((1-Tosyl-1*H*-1,2,3-triazol-4-yl)methyl)pyridin-1-ium-2-yl)trifluoroborate (3i)

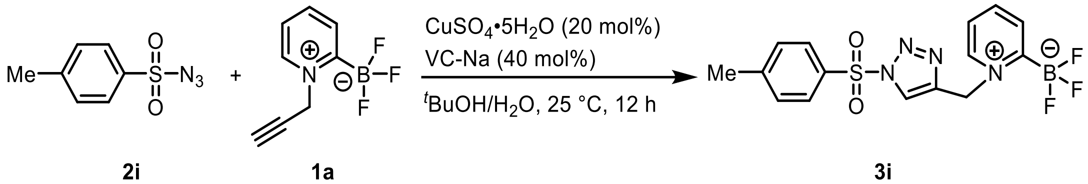

2i 1a 3i

Under an ambient atmosphere, 4-methylbenzenesulfonyl azide (88.7 mg, 0.450 mmol, 1.50 equiv.) in ^{t}BuOH:H_2O (1:1, 1.5 mL, 0.2 M) was to a 4 mL vial containing a magnetic stir bar. Subsequently, $CuSO_4{\cdot}5H_2O$ (15 mg, 60 μmol, 20 mol%), and sodium ascorbate (23.8 mg, 120 μmol, 40 mol%) were added was added into the tube, followed by 2-pyridinium trifluoroborate salt (55.5 mg, 0.300 mmol, 1.00 equiv.). The

tube was sealed with a Teflon cap, and the reaction mixture was stirred vigorously at 25 °C. After the indicated time, the reaction vessel was opened to air, and the resulting mixture was quenched with saturated $NaHCO_3$ (10 mL), then extracted into EtOAc (3 × 10 mL). The combined organic phase was washed with brine, dried over Na_2SO_4, and concentrated by rotary evaporation. The residue was purified by chromatography on silica gel, eluting with dichloromethane/methanol (from 100:1 to 50:1, v:v) to afford 49.4 mg (43% yield) of the title compound as a white solid.

R_f = 0.30 (dichloromethane/methanol, 100:1, v/v (UV))

NMR Spectroscopy:

^{1}H NMR (400 MHz, $CDCl_3$, 25 °C, δ): 8.77 (d, *J* = 6.3 Hz, 1H), 8.51 (s, 1H), 8.26 (d, *J* = 7.7 Hz, 1H), 8.13 (t, *J* = 7.6 Hz, 1H), 7.97 (d, *J* = 8.4 Hz, 2H), 7.67 (ddd, *J* = 7.6, 6.1, 1.5 Hz, 1H), 7.38 (d, *J* = 8.3 Hz, 2H), 5.97 (s, 2H), 2.44 (s, 3H).

^{13}C {^{1}H} NMR (101 MHz, $CDCl_3$, 25 °C, δ): 147.97, 143.24, 142.42, 140.94, 133.08 (q, *J* = 2.4 Hz), 132.52, 130.74, 129.06, 125.64, 125.54 (q, *J* = 4.0 Hz), 53.62 (q, *J* = 2.8 Hz), 22.00. The carbon directly attached to the boron atom was not detected due to quadrupolar broadening.

^{19}F {^{1}H} NMR (376 MHz, $CDCl_3$, 25 °C, δ): –142.15 (q, *J* = 38.5 Hz).

^{11}B NMR (128 MHz, $CDCl_3$, 25 °C, δ): 0.73 (q, *J* = 38.5 Hz).

HRMS ESI (m/z) calc'd for $C_{15}H_{11}N_4BNaO_2SF_3^+$ $[M+Na]^+$, 405.0775; found, 405.0771. Deviation: –1.0 ppm.

(1-((1-(2-Ethoxy-2-oxoethyl)-1*H*-1,2,3-triazol-4-yl)methyl)pyridin-1-ium-4-yl)trifluoroborate (5j)

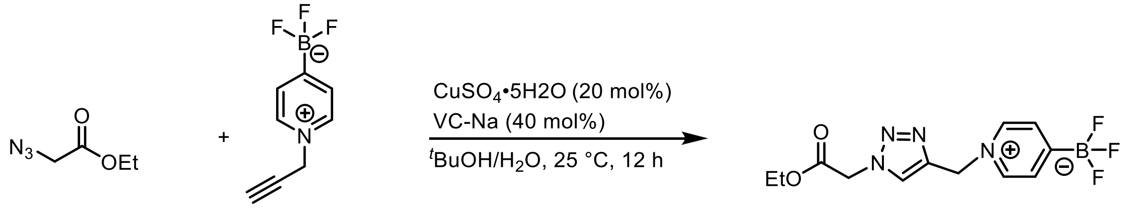

2j **1c** **5j**

Under an ambient atmosphere, ethyl 2-azidoacetate (19.4 mg, 0.150 mmol, 1.50 equiv.) in ^{t}BuOH:H_2O (1:1, 0.5 mL, 0.2 M) was to a 4 mL vial containing a magnetic stir bar. Subsequently, $CuSO_4·5H_2O$ (5 mg, 20 μmol, 20 mol%), and sodium ascorbate (7.9 mg, 40 μmol, 40 mol%) were added was added into the tube, followed by 4-pyridinium trifluoroborate salt (18.5 mg, 0.100 mmol, 1.00 equiv.). The tube was sealed with a Teflon cap, and the reaction mixture was stirred vigorously at 25 °C. After the indicated time, the reaction vessel was opened to air, and the resulting mixture was concentrated by rotary evaporation. The residue was purified by chromatography on silica gel, eluting with dichloromethane/methanol (from 200:1 to 100:1, v:v) to afford 24.7 mg (79% yield) of the title compound as a pale yellow solid.

R*f* = 0.50 (dichloromethane/methanol, 10:1, v/v (UV))

NMR Spectroscopy:

^{1}H NMR (400 MHz, DMSO-*d*$_6$, 25 °C, δ): 8.81 (d, *J* = 6.1 Hz, 2H), 8.33 (s, 1H), 7.96 (d, *J* = 6.4 Hz, 2H), 5.90 (s, 2H), 5.43 (s, 2H), 4.17 (q, *J* = 7.2 Hz, 2H), 1.20 (t, *J* = 7.1 Hz, 3H).

^{13}C {^{1}H} NMR (101 MHz, DMSO-*d*$_6$, 25 °C, δ): 167.09, 141.77, 140.63, 130.23 (q, *J* = 1.9 Hz), 126.39, 61.63, 53.92, 50.60, 13.98. The carbon directly attached to the boron atom was not detected due to quadrupolar broadening.

^{19}F {^{1}H} NMR (376 MHz, DMSO-*d*$_6$, 25 °C, δ): –142.63 (brs).

^{11}B NMR (128 MHz, DMSO-*d*$_6$, 25 °C, δ): 2.02 (brs).

HRMS ESI (m/z) calc'd for $C_{12}H_{14}BF_3N_4NaO_2^+$ [M+Na]$^+$, 337.1054; found, 337.1054. Deviation: 0.0 ppm.

(1-((1-(4-Hydroxyphenyl)-1*H*-1,2,3-triazol-4-yl)methyl)pyridin-1-ium-2-yl)trifluoroborate (3k)

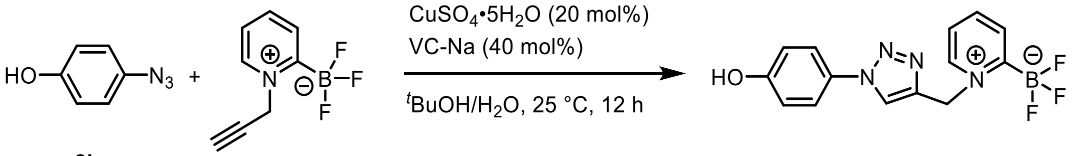

2k 1a 3k

Under an ambient atmosphere, 4-azidophenol (60.8 mg, 0.450 mmol, 1.50 equiv.) in ^{t}BuOH:H_2O (1:1, 1.5 mL, 0.2 M) was to a 4 mL vial containing a magnetic stir bar. Subsequently, $CuSO_4{\cdot}5H_2O$ (15 mg, 60 μmol, 20 mol%), and sodium ascorbate (23.8 mg, 120 μmol, 40 mol%) were added was added into the tube, followed by 2-pyridinium trifluoroborate salt (55.5 mg, 0.300 mmol, 1.00 equiv.). The tube was sealed with a Teflon cap, and the reaction mixture was stirred vigorously at 25 °C. After the indicated time, the reaction vessel was opened to air, and the resulting mixture was quenched with saturated $NaHCO_3$ (10 mL), then extracted into EtOAc (3 × 10 mL). The combined organic phase was washed with brine, dried over Na_2SO_4, and concentrated by rotary evaporation. The residue was purified by chromatography on silica gel, eluting with dichloromethane/methanol (from 100:1 to 50:1, v:v) to afford 69.7 mg (73% yield) of the title compound as a white solid.

R*f* = 0.50 (dichloromethane/methanol, 10:1, v/v (UV))

NMR Spectroscopy:

^{1}H NMR (400 MHz, DMSO-*d*$_6$, 25 °C, δ): 10.00 (s, 1H), 9.00 (d, *J* = 6.2 Hz, 1H), 8.57 (s, 1H), 8.36 (t, *J* = 7.7 Hz, 1H), 8.06 (d, *J* = 7.4 Hz, 1H), 7.94 (ddd, *J* = 7.8, 6.2, 1.7 Hz, 1H), 7.64 (d, *J* = 8.8 Hz, 2H), 6.93 (d, *J* = 8.8 Hz, 2H), 6.05 (s, 2H).

^{13}C {^{1}H} NMR (101 MHz, DMSO-*d*$_6$, 25 °C, δ): 158.00, 145.34, 143.22, 142.14, 131.31 (q, *J* = 2.7 Hz), 128.50, 125.95, 122.81, 122.19, 116.11, 53.26 (q, *J* = 3.7 Hz).The carbon directly attached to the boron atom was not detected due to quadrupolar broadening.

^{19}F {^{1}H} NMR (376 MHz, DMSO-d_6, 25 °C, δ): –139.11 (brs).

^{11}B NMR (128 MHz, DMSO-d_6, 25 °C, δ): 0.94 (brs).

HRMS ESI (m/z) calc'd for $C_{14}H_{12}N_4BNaOF_3^+$ [M+Na]$^+$, 343.0948; found, 343.0944. Deviation: –1.2 ppm.

(1-((1-Benzhydryl-1*H*-1,2,3-triazol-4-yl)methyl)pyridin-1-ium-2-yl)trifluoroborate (3l)

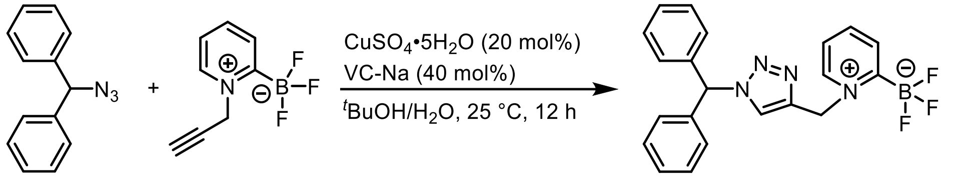

2l **1a** **3l**

Under an ambient atmosphere, (azidomethylene)dibenzene (94.1 mg, 0.450 mmol, 1.50 equiv.) in ^{t}BuOH:H_2O (1:1, 1.5 mL, 0.2 M) was to a 4 mL vial containing a magnetic stir bar. Subsequently, $CuSO_4$·$5H_2O$ (15 mg, 60 μmol, 20 mol%), and sodium ascorbate (23.8 mg, 120 μmol, 40 mol%) were added was added into the tube, followed by 2-pyridinium trifluoroborate salt (55.5 mg, 0.300 mmol, 1.00 equiv.). The tube was sealed with a Teflon cap, and the reaction mixture was stirred vigorously at 25 °C. After the indicated time, the reaction vessel was opened to air, and the resulting mixture was quenched with saturated $NaHCO_3$ (10 mL), then extracted into EtOAc (3 × 10 mL). The combined organic phase was washed with brine, dried over Na_2SO_4, and concentrated by rotary evaporation. The residue was purified by chromatography on silica gel, eluting with dichloromethane/methanol (from 200:1 to 150:1, v:v) to afford 77.6 mg (66% yield) of the title compound as a colorless solid.

R*f* = 0.45 (dichloromethane/methanol, 100:1, v/v (UV))

NMR Spectroscopy:

^{1}H NMR (400 MHz, $CDCl_3$, 25 °C, δ): 8.88 (d, *J* = 6.1 Hz, 1H), 8.22 (d, *J* = 7.8 Hz, 1H), 8.07 (t, *J* = 7.7 Hz, 1H), 7.89 (s, 1H), 7.62 (ddd, *J* = 7.7, 6.2, 1.8 Hz, 1H), 7.36 – 7.29 (m, 6H), 7.12 – 7.05 (m, 4H), 7.01 (s, 1H), 5.95 (s, 2H).

^{13}C {^{1}H} NMR (101 MHz, $CDCl_3$, 25 °C, δ): 143.38, 142.04, 140.97, 137.61, 132.74 (q, *J* = 2.4 Hz), 129.08, 128.83, 128.09, 125.57 (q, *J* = 3.6 Hz), 125.44, 68.71, 53.93 (q, *J* = 3.0 Hz). The carbon directly attached to the boron atom was not detected due to quadrupolar broadening.

^{19}F {^{1}H} NMR (376 MHz, $CDCl_3$, 25 °C, δ): –142.19 (q, *J* = 39.6 Hz).

^{11}B NMR (128 MHz, $CDCl_3$, 25 °C, δ): 0.77 (q, *J* = 39.6 Hz).

HRMS ESI (m/z) calc'd for $C_{21}H_{18}BF_3NaN_4^+$ [M+Na]$^+$, 417.1469; found, 417.1465. Deviation: –1.0 ppm.

Sulfadoxine derivative (4m)

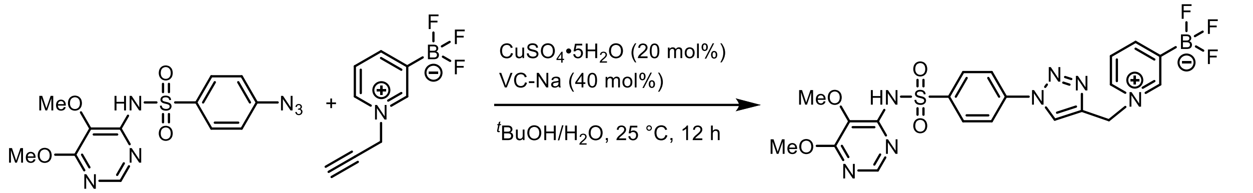

Under an ambient atmosphere, 4-azido-*N*-(5,6-dimethoxypyrimidin-4-yl)benzenesulfonamide (151 mg, 0.450 mmol, 1.50 equiv.) in ^{t}BuOH:H_2O (1:1, 1.5 mL, 0.2 M) was to a 4 mL vial containing a magnetic stir bar. Subsequently, $CuSO_4·5H_2O$ (15 mg, 60 μmol, 20 mol%), and sodium ascorbate (23.8 mg, 120 μmol, 40 mol%) were added was added into the tube, followed by 3-pyridinium trifluoroborate salt (55.5 mg, 0.300 mmol, 1.00 equiv.). The tube was sealed with a Teflon cap, and the reaction mixture was stirred vigorously at 25 °C. After the indicated time, the reaction vessel was opened to air, and the resulting mixture was concentrated by rotary evaporation. The residue was purified by chromatography on silica gel, eluting with dichloromethane/methanol (from 100:1 to 50:1, v:v) to afford 82.6 mg (53% yield) of the title compound as a pale yellow solid.

R*f* = 0.50 (dichloromethane/methanol, 10:1, v/v (UV))

NMR Spectroscopy:

^{1}H NMR (400 MHz, DMSO-*d*6, 25 °C, δ): 11.36 (s, 1H), 9.08 (s, 1H), 8.91 (dt, *J* = 6.1, 1.6 Hz, 1H), 8.87 (s, 1H), 8.42 (d, *J* = 7.5 Hz, 1H), 8.20 (d, *J* = 8.8 Hz, 2H), 8.13 (d, *J* = 7.5 Hz, 3H), 7.96 (t, *J* = 6.8 Hz, 1H), 6.05 (s, 2H), 3.91 (s, 3H), 3.72 (s, 3H).

^{13}C {^{1}H} NMR (101 MHz, DMSO-*d*6, 25 °C, δ): 161.75, 150.39 (2C), 148.55, 145.91 (q, *J* = 1.6 Hz), 142.13, 141.95, 141.08, 138.98, 129.35, 127.62, 126.97, 123.78, 120.58, 60.24, 54.15, 54.05. The carbon directly attached to the boron atom was not detected due to quadrupolar broadening.

^{19}F {^{1}H} NMR (376 MHz, DMSO-*d*6, 25 °C, δ): –140.45 (brs).

^{11}B NMR (128 MHz, DMSO-*d*6, 25 °C, δ): 1.87 (brs).

HRMS ESI (m/z) calc'd for $C_{20}H_{19}N_7BNaO_4SF_3^+$ [M+Na]$^+$, 544.1157; found, 544.1163. Deviation: +1.1 ppm.

Indomethacin derivative (3n)

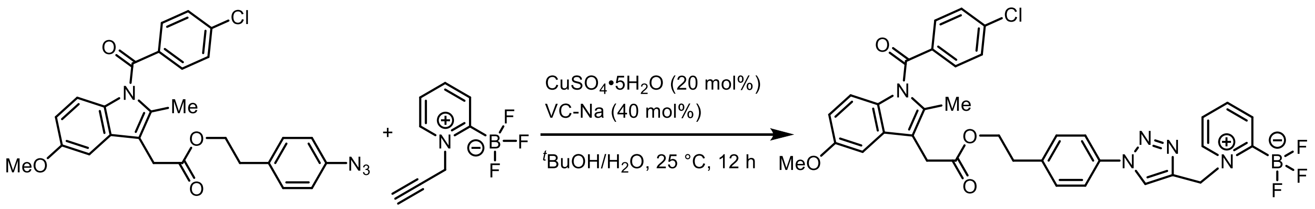

Under an ambient atmosphere, 4-azidophenethyl 2-(1-(4-chlorobenzoyl)-5-methoxy-2-methyl-1*H*-indol-3-yl)acetate (226 mg, 0.450 mmol, 1.50 equiv.) in ^{t}BuOH:H_2O (1:1, 1.5 mL, 0.2 M) was to a 4 mL vial containing

a magnetic stir bar. Subsequently, $CuSO_4·5H_2O$ (15 mg, 60 µmol, 20 mol%), and sodium ascorbate (23.8 mg, 120 µmol, 40 mol%) were added was added into the tube, followed by 2-pyridinium trifluoroborate salt (55.5 mg, 0.300 mmol, 1.00 equiv.). The tube was sealed with a Teflon cap, and the reaction mixture was stirred vigorously at 25 °C. After the indicated time, the reaction vessel was opened to air, and the resulting mixture was quenched with saturated $NaHCO_3$ (10 mL), then extracted into EtOAc (3 × 10 mL). The combined organic phase was washed with brine, dried over Na_2SO_4, and concentrated by rotary evaporation. The residue was purified by chromatography on silica gel, eluting with dichloromethane/methanol (from 100:1 to 50:1, v:v) to afford 195.3 mg (95% yield) of the title compound as a pale yellow solid.

R*f* = 0.50 (dichloromethane/methanol, 10:1, v/v (UV))

NMR Spectroscopy:

^{1}H NMR (400 MHz, DMSO-*d*6, 25 °C, δ): 8.97 (d, *J* = 6.1 Hz, 1H), 8.62 (s, 1H), 8.31 (t, *J* = 7.6 Hz, 1H), 8.03 (d, *J* = 7.5 Hz, 1H), 7.89 (t, *J* = 6.4 Hz, 1H), 7.62 (d, *J* = 8.3 Hz, 2H), 7.58 – 7.52 (m, 4H), 7.26 (d, *J* = 8.3 Hz, 2H), 6.90 (d, *J* = 2.0 Hz, 1H), 6.83 (d, *J* = 9.0 Hz, 1H), 6.60 (dd, *J* = 8.9, 2.1 Hz, 1H), 6.03 (s, 2H), 4.25 (t, *J* = 6.1 Hz, 2H), 3.67 (s, 2H), 3.62 (s, 3H), 2.87 (t, *J* = 6.0 Hz, 2H), 2.10 (s, 3H).

^{13}C {^{1}H} NMR (101 MHz, DMSO-*d*6, 25 °C, δ): 170.39, 167.80, 155.54, 145.31, 143.19, 142.40, 139.04, 137.71, 135.37, 134.71, 134.01, 131.30 (q, *J* = 2.5 Hz), 131.12, 130.48, 130.20, 130.13, 129.01, 125.91, 122.70, 119.95, 114.55, 112.62, 111.23, 101.63, 64.48, 55.29, 53.23 (q, *J* = 3.5 Hz), 33.79, 29.43, 13.12. The carbon directly attached to the boron atom was not detected due to quadrupolar broadening.

^{19}F {^{1}H} NMR (376 MHz, DMSO-*d*6, 25 °C, δ): –139.14 (brs).

^{11}B NMR (128 MHz, DMSO-*d*6, 25 °C, δ): 0.91 (brs).

HRMS ESI (m/z) calc'd for $C_{35}H_{30}N_5BNaO_4ClF_3^+$ [M+Na]$^+$, 710.1924; found, 710.1917. Deviation: –1.0 ppm.

Afatinib intermediate derivative (3o)

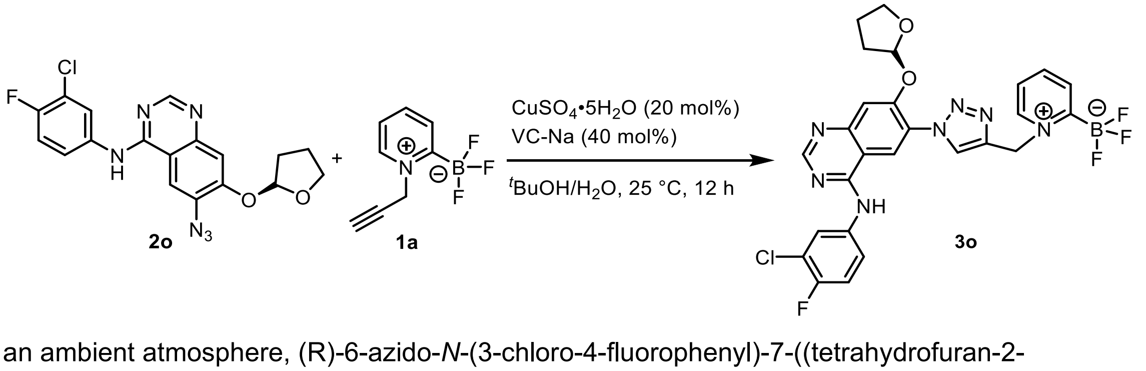

Under an ambient atmosphere, (R)-6-azido-*N*-(3-chloro-4-fluorophenyl)-7-((tetrahydrofuran-2-yl)oxy)quinazolin-4-amine (120 mg, 0.300 mmol, 1.00 equiv.) in ^{t}BuOH:H_2O (1:1, 1.5 mL, 0.2 M) was to a 4 mL vial containing a magnetic stir bar. Subsequently, $CuSO_4·5H_2O$ (15 mg, 60 µmol, 20 mol%), and sodium ascorbate (23.8 mg, 120 µmol, 40 mol%) were added was added into the tube, followed by 2-pyridinium trifluoroborate salt (55.5 mg, 0.300 mmol, 1.00 equiv.). The tube was sealed with a Teflon cap, and the reaction mixture was stirred vigorously at 25 °C. After the indicated time, the reaction vessel was opened to

air, and the resulting mixture was quenched with saturated $NaHCO_3$ (10 mL), then extracted into EtOAc (3 × 10 mL). The combined organic phase was washed with brine, dried over Na_2SO_4, and concentrated by rotary evaporation. The residue was purified by chromatography on silica gel, eluting with dichloromethane/methanol (from 100:1 to 50:1, v:v) to afford 127.2 mg (72% yield) of the title compound as a pale yellow solid.

R*f* = 0.50 (dichloromethane/methanol, 10:1, v/v (UV))

NMR Spectroscopy:

^{1}H NMR (400 MHz, DMSO-*d*$_6$, 25 °C, δ): 9.98 (s, 1H), 9.05 (d, *J* = 6.2 Hz, 1H), 8.91 (s, 1H), 8.62 (s, 1H), 8.54 (s, 1H), 8.40 (t, *J* = 7.7 Hz, 1H), 8.16 (dd, *J* = 6.9, 2.6 Hz, 1H), 8.09 (dd, *J* = 7.8, 1.7 Hz, 1H), 7.98 (ddd, *J* = 7.8, 6.3, 1.8 Hz, 1H), 7.80 (ddd, *J* = 9.0, 4.2, 2.7 Hz, 1H), 7.48 – 7.36 (m, 2H), 6.13 (s, 2H), 5.34 (t, *J* = 5.0 Hz, 1H), 3.92 (dd, *J* = 10.5, 4.4 Hz, 1H), 3.79 (d, *J* = 10.4 Hz, 1H), 3.71 (td, *J* = 8.3, 4.4 Hz, 1H), 3.64 (q, *J* = 8.1 Hz, 1H), 2.26 (td, *J* = 14.3, 8.2 Hz, 1H), 1.99 (dt, *J* = 12.3, 5.4 Hz, 1H).

^{13}C {^{1}H} NMR (101 MHz, DMSO-*d*$_6$, 25 °C, δ): 157.31, 155.82, 154.56, 152.86, 152.15, 151.58, 145.24, 143.31, 141.84, 136.30 (d, *J* = 3.1 Hz), 131.37 (q, *J* = 2.3 Hz), 126.91 (d, *J* = 3.0 Hz), 125.99 (d, *J* = 5.2 Hz), 123.42, 122.21 (d, *J* = 6.9 Hz), 120.82, 118.79 (d, *J* = 18.4 Hz), 116.52 (d, *J* = 21.6 Hz), 109.90, 108.85, 79.35, 71.75, 66.35, 53.35 (q, *J* = 3.3 Hz), 32.19. The carbon directly attached to the boron atom was not detected due to quadrupolar broadening.

^{19}F {^{1}H} NMR (376 MHz, DMSO-*d*$_6$, 25 °C, δ): –122.58 (1F), –139.15 (brs, 3F).

^{11}B NMR (128 MHz, DMSO-*d*$_6$, 25 °C, δ): 0.90 (brs).

HRMS ESI (m/z) calc'd for $C_{26}H_{21}N_7BNaO_2ClF_4^+$ [M+Na]$^+$, 608.1367; found, 608.1374. Deviation: +1.2 ppm.

Aminoglutethimide derivative (5p)

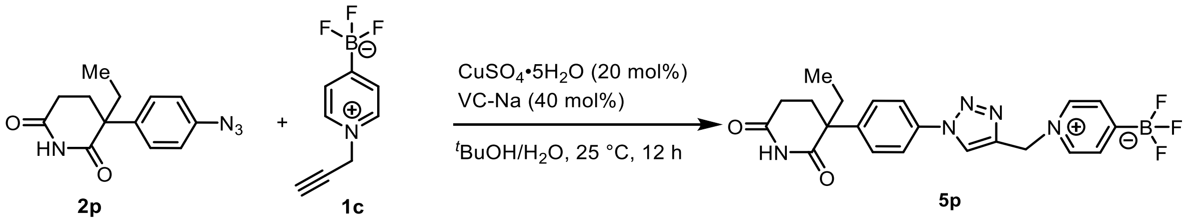

Under an ambient atmosphere, 3-(4-azidophenyl)-3-ethylpiperidine-2,6-dione (116 mg, 0.450 mmol, 1.50 equiv.) in ^{t}BuOH:H_2O (1:1, 1.5 mL, 0.2 M) was to a 4 mL vial containing a magnetic stir bar. Subsequently, $CuSO_4{\cdot}5H_2O$ (15 mg, 60 μmol, 20 mol%), and sodium ascorbate (23.8 mg, 120 μmol, 40 mol%) were added was added into the tube, followed by 4-pyridinium trifluoroborate salt (55.5 mg, 0.300 mmol, 1.00 equiv.). The tube was sealed with a Teflon cap, and the reaction mixture was stirred vigorously at 25 °C. After the indicated time, the reaction vessel was opened to air, and the resulting mixture was concentrated by rotary evaporation. The residue was purified by chromatography on silica gel, eluting with dichloromethane/methanol (from 100:1 to 50:1, v:v) to afford 111.4 mg (84% yield) of the title compound as a pale yellow solid.

R*f* = 0.50 (dichloromethane/methanol, 10:1, v/v (UV))

NMR Spectroscopy:

^{1}H NMR (400 MHz, CD_3CN, 25 °C, δ): 8.80 (s, 1H), 8.55 (d, *J* = 6.2 Hz, 2H), 8.44 (s, 1H), 8.02 (d, *J* = 6.0 Hz, 2H), 7.83 – 7.73 (m, 2H), 7.57 – 7.45 (m, 2H), 5.76 (s, 2H), 2.60 – 2.38 (m, 2H), 2.33 – 2.25 (m, 2H), 2.09 – 1.87 (m, 2H), 0.84 (t, *J* = 7.4 Hz, 3H).

^{13}C {^{1}H} NMR (101 MHz, CD_3CN, 25 °C, δ): 176.16, 173.25, 142.30, 142.17, 141.85, 136.72, 131.55 (q, *J* = 1.8 Hz), 129.13, 124.33, 121.92, 55.57, 51.79, 33.17, 29.91, 27.66, 9.32. The carbon directly attached to the boron atom was not detected due to quadrupolar broadening.

^{19}F {^{1}H} NMR (376 MHz, CD_3CN, 25 °C, δ): –145.60 (q, *J* = 47.6 Hz).

^{11}B NMR (128 MHz, CD_3CN, 25 °C, δ): 1.66 (q, *J* = 47.6 Hz).

HRMS ESI (m/z) calc'd for $C_{21}H_{21}N_5BNaO_2F_3^+$ [M+Na]$^+$, 466.1633; found, 466.1633. Deviation: 0.0 ppm.

Linezolid derivative (3q)

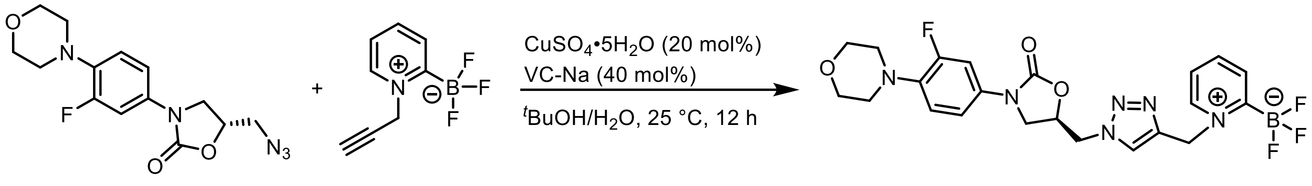

2q **1a** **3q**

Under an ambient atmosphere, (*R*)-5-(azidomethyl)-3-(3-fluoro-4-morpholinophenyl)oxazolidin-2-one (144 mg, 0.450 mmol, 1.50 equiv.) in ^{t}BuOH:H_2O (1:1, 1.5 mL, 0.2 M) was to a 4 mL vial containing a magnetic stir bar. Subsequently, $CuSO_4 \cdot 5H_2O$ (15 mg, 60 μmol, 20 mol%), and sodium ascorbate (23.8 mg, 120 μmol, 40 mol%) were added was added into the tube, followed by 2-pyridinium trifluoroborate salt (55.5 mg, 0.300 mmol, 1.00 equiv.). The tube was sealed with a Teflon cap, and the reaction mixture was stirred vigorously at 25 °C. After the indicated time, the reaction vessel was opened to air, and the resulting mixture was quenched with saturated $NaHCO_3$ (10 mL), then extracted into EtOAc (3 × 10 mL). The combined organic phase was washed with brine, dried over Na_2SO_4, and concentrated by rotary evaporation. The residue was purified by chromatography on silica gel, eluting with dichloromethane/methanol (from 200:1 to 50:1, v:v) to afford 141.5 mg (93% yield) of the title compound as a pale yellow solid.

R*f* = 0.10 (dichloromethane/methanol, 100:1, v/v (UV))

NMR Spectroscopy:

^{1}H NMR (400 MHz, DMSO-*d*$_6$, 25 °C, δ): 8.88 (d, *J* = 6.2 Hz, 1H), 8.41 – 8.28 (m, 1H), 8.22 (s, 1H), 8.03 (dd, *J* = 7.8, 1.8 Hz, 1H), 7.88 (ddd, *J* = 7.9, 6.2, 1.8 Hz, 1H), 7.38 (dd, *J* = 14.9, 2.5 Hz, 1H), 7.19 – 6.96 (m, 2H), 5.96 (s, 2H), 5.10 (dq, *J* = 10.9, 5.5 Hz, 1H), 4.90 – 4.72 (m, 2H), 4.18 (t, *J* = 9.2 Hz, 1H), 3.82 (dd, *J* = 9.4, 5.7 Hz, 1H), 3.78 – 3.65 (m, 4H), 3.02 – 2.91 (m, 4H).

^{13}C {^{1}H} NMR (101 MHz, DMSO-*d*$_6$, 25 °C, δ): 155.71, 153.37 (d, *J* = 15.8 Hz), 144.97, 143.10, 141.43,

135.73 (d, J = 8.8 Hz), 132.97 (d, J = 10.6 Hz), 131.22 (q, J = 2.6 Hz), 125.88, 119.19 (d, J = 4.2 Hz), 114.27 (d, J = 3.1 Hz), 106.79 (d, J = 26.1 Hz), 70.73, 66.17, 54.91, 53.05 (q, J = 2.6 Hz), 52.13, 50.67 (d, J = 3.0 Hz), 47.11. The carbon directly attached to the boron atom was not detected due to quadrupolar broadening.

^{19}F {^{1}H} NMR (376 MHz, DMSO-d_6, 25 °C, δ): –121.21 (1F), –139.11 (q, J = 47.2 Hz, 3F).

^{11}B NMR (128 MHz, DMSO-d_6, 25 °C, δ): 0.86 (q, J = 47.2 Hz).

HRMS ESI (m/z) calc'd for $C_{22}H_{23}BF_4N_6NaO_3^+$ [M+Na]$^+$, 529.1753; found, 529.1753. Deviation: 0.0 ppm.

Dipeptide derivative (3r)

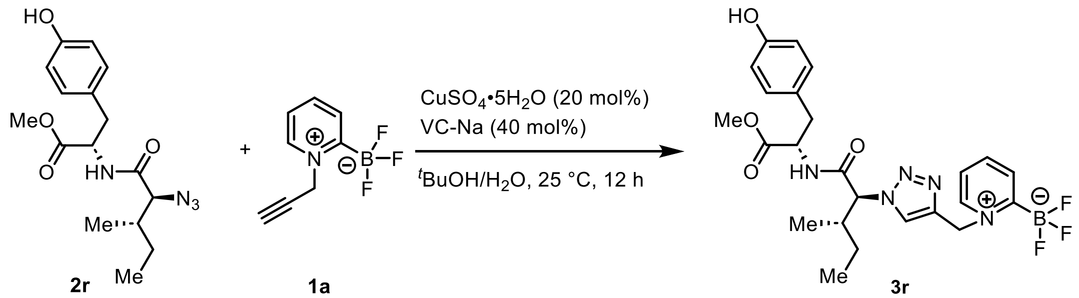

2r 1a 3r

Under an ambient atmosphere, methyl ((2*S*,3*S*)-2-azido-3-methylpentanoyl)-*L*-tyrosinate (117 mg, 0.350 mmol, 1.17 equiv.) in ^{t}BuOH:H_2O (1:1, 1.5 mL, 0.2 M) was to a 4 mL vial containing a magnetic stir bar. Subsequently, $CuSO_4\cdot 5H_2O$ (15 mg, 60 μmol, 20 mol%), and sodium ascorbate (23.8 mg, 120 μmol, 40 mol%) were added was added into the tube, followed by 2-pyridinium trifluoroborate salt (55.5 mg, 0.300 mmol, 1.00 equiv.). The tube was sealed with a Teflon cap, and the reaction mixture was stirred vigorously at 25 °C. After the indicated time, the reaction vessel was opened to air, and the resulting mixture was quenched with saturated $NaHCO_3$ (10 mL), then extracted into EtOAc (3 × 10 mL). The combined organic phase was washed with brine, dried over Na_2SO_4, and concentrated by rotary evaporation. The residue was purified by chromatography on silica gel, eluting with dichloromethane/methanol (from 100:1 to 50:1, v:v) to afford 98.7 mg (63% yield) of the title compound as a colourless solid.

R_f = 0.70 (dichloromethane/methanol, 10:1, v/v (UV))

NMR Spectroscopy:

^{1}H NMR (400 MHz, DMSO-d_6, 25 °C, δ): 9.23 (s, 1H), 9.02 (d, J = 6.9 Hz, 1H), 8.91 (d, J = 6.1 Hz, 1H), 8.34 (t, J = 7.6 Hz, 1H), 8.12 (s, 1H), 8.04 (d, J = 7.6 Hz, 1H), 7.91 (t, J = 6.4 Hz, 1H), 6.89 (d, J = 8.2 Hz, 2H), 6.57 (d, J = 8.2 Hz, 2H), 5.96 (s, 2H), 5.74 (s, 1H), 5.19 (d, J = 10.4 Hz, 1H), 4.36 (q, J = 7.0 Hz, 1H), 3.56 (s, 3H), 2.84 (qd, J = 13.9, 7.2 Hz, 2H), 2.24 – 2.13 (m, 1H), 0.92 (d, J = 6.6 Hz, 3H), 0.85 – 0.75 (m, 1H), 0.71 (t, J = 7.1 Hz, 3H).

^{13}C {^{1}H} NMR (101 MHz, DMSO-d_6, 25 °C, δ): 171.50, 167.47, 156.06, 145.12, 143.16, 141.55, 131.32 (q, J = 2.7 Hz), 129.93, 126.46, 125.94, 123.48 (q, J = 2.8 Hz), 115.03, 66.58, 54.17, 53.30 (q, J = 3.4 Hz), 51.82, 37.03, 35.75, 24.17, 14.65, 9.74. The carbon directly attached to the boron atom was not

detected due to quadrupolar broadening.

^{19}F {^{1}H} NMR (376 MHz, CD_3CN, 25 °C, δ): –139.21 (brs).

^{11}B NMR (128 MHz, DMSO-*d*$_6$, 25 °C, δ): 0.68 (brs).

HRMS ESI (m/z) calc'd for $C_{24}H_{29}N_5BNaO_4F_3^+$ [M+Na]$^+$, 542.2157; found, 542.2159. Deviation: +0.4 ppm.

Zidovudine derivative (4s)

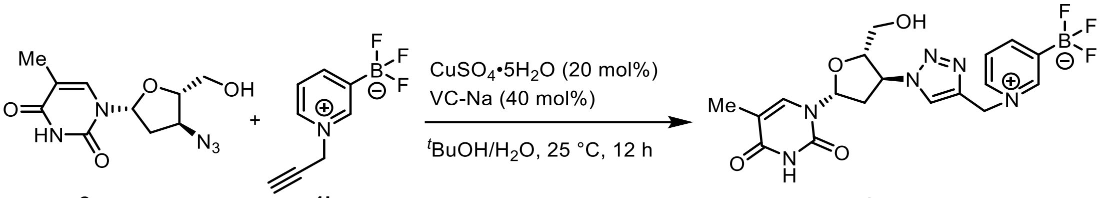

Under an ambient atmosphere, 1-((2*R*,4*S*,5*S*)-4-azido-5-(hydroxymethyl)tetrahydrofuran-2-yl)-5-methylpyrimidine-2,4(1*H,*3*H*)-dione (120 mg, 0.450 mmol, 1.50 equiv.) in ^{t}BuOH:H_2O (1:1, 1.5 mL, 0.2 M) was to a 4 mL vial containing a magnetic stir bar. Subsequently, $CuSO_4 \cdot 5H_2O$ (15 mg, 60 µmol, 20 mol%), and sodium ascorbate (23.8 mg, 120 µmol, 40 mol%) were added was added into the tube, followed by 3-pyridinium trifluoroborate salt (55.5 mg, 0.300 mmol, 1.00 equiv.). The tube was sealed with a Teflon cap, and the reaction mixture was stirred vigorously at 25 °C. After the indicated time, the reaction vessel was opened to air, and the resulting mixture was concentrated by rotary evaporation. The residue was purified by chromatography on silica gel, eluting with dichloromethane/methanol (from 100:1 to 50:1, v:v) to afford 72.8 mg (54% yield) of the title compound as a white solid.

R*f* = 0.30 (dichloromethane/methanol, 100:1, v/v (UV))

NMR Spectroscopy:

^{1}H NMR (400 MHz, DMSO-*d*$_6$, 25 °C, δ): 11.35 (s, 1H), 8.88 (dt, *J* = 6.1, 1.6 Hz, 1H), 8.83 (s, 1H), 8.50 (s, 1H), 8.40 (d, *J* = 7.4 Hz, 1H), 7.94 (t, *J* = 6.8 Hz, 1H), 7.80 (d, *J* = 1.4 Hz, 1H), 6.41 (t, *J* = 6.7 Hz, 1H), 5.95 (s, 2H), 5.41 (dt, *J* = 8.4, 5.0 Hz, 1H), 5.29 (t, *J* = 5.2 Hz, 1H), 4.21 (dt, *J* = 5.1, 3.6 Hz, 1H), 3.74 – 3.57 (m, 2H), 2.78 – 2.57 (m, 2H), 1.80 (s, 3H).

^{13}C {^{1}H} NMR (101 MHz, DMSO-*d*$_6$, 25 °C, δ): 163.71, 150.44, 148.39, 145.75 (q, *J* = 1.9 Hz), 142.01, 140.73, 136.18, 126.89, 124.74, 109.66, 84.37, 83.90, 60.81, 59.72, 54.10, 37.06, 12.25. The carbon directly attached to the boron atom was not detected due to quadrupolar broadening.

^{19}F {^{1}H} NMR (376 MHz, DMSO-*d*$_6$, 25 °C, δ): –140.46 (brs).

^{11}B NMR (128 MHz, DMSO-*d*$_6$, 25 °C, δ): 1.92 (brs).

HRMS ESI (m/z) calc'd for $C_{18}H_{20}N_6BNaO_4F_3^+$ [M+Na]$^+$, 475.1483; found, 475.1483. Deviation: 0.0 ppm.

Amlodipine derivative (3t)

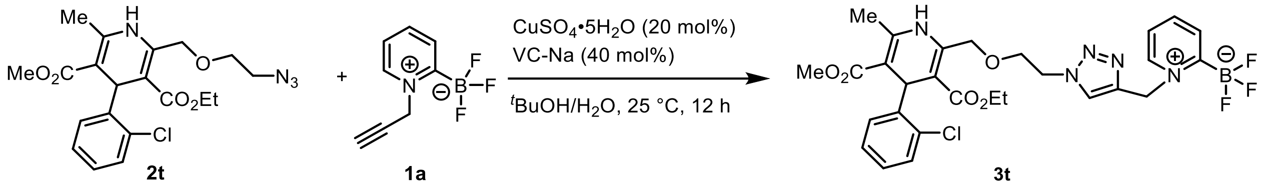

Under an ambient atmosphere, 3-ethyl 5-methyl 2-((2-azidoethoxy)methyl)-4-(2-chlorophenyl)-6-methyl-1,4-dihydropyridine-3,5-dicarboxylate (43.5 mg, 0.100 mmol, 1.00 equiv.) in ^{t}BuOH:H_2O (1:1, 0.5 mL, 0.2 M) was to a 4 mL vial containing a magnetic stir bar. Subsequently, $CuSO_4 \cdot 5H_2O$ (5 mg, 20 μmol, 20 mol%), and sodium ascorbate (7.9 mg, 40 μmol, 40 mol%) were added was added into the tube, followed by 2-pyridinium trifluoroborate salt (18.5 mg, 0.100 mmol, 1.00 equiv.). The tube was sealed with a Teflon cap, and the reaction mixture was stirred vigorously at 25 °C. After the indicated time, the reaction vessel was opened to air, and the resulting mixture was quenched with saturated $NaHCO_3$ (5 mL), then extracted into EtOAc (3 × 5 mL). The combined organic phase was washed with brine, dried over Na_2SO_4, and concentrated by rotary evaporation. The residue was purified by chromatography on silica gel, eluting with dichloromethane/methanol (from 100:1 to 50:1, v:v) to afford 48.5 mg (78% yield) of the title compound as a pale yellow solid.

R*f* = 0.40 (dichloromethane/methanol, 10:1, v/v (UV))

NMR Spectroscopy:

^{1}H NMR (400 MHz, CD_3CN, 25 °C, δ): 9.33 (d, *J* = 6.2 Hz, 1H), 8.75 (t, *J* = 7.7 Hz, 1H), 8.65 (d, *J* = 7.5 Hz, 1H), 8.60 (s, 1H), 8.27 (ddd, *J* = 7.7, 6.2, 1.7 Hz, 1H), 7.91 (dd, *J* = 7.8, 1.6 Hz, 1H), 7.88 (s, 1H), 7.80 (dd, *J* = 7.9, 1.2 Hz, 1H), 7.74 (td, *J* = 7.5, 1.4 Hz, 1H), 7.65 (td, *J* = 7.6, 1.8 Hz, 1H), 6.51 (s, 2H), 5.88 (s, 1H), 5.18 (d, *J* = 3.8 Hz, 2H), 5.14 (t, *J* = 5.0 Hz, 2H), 4.54 (qt, *J* = 7.1, 3.5 Hz, 2H), 4.48 – 4.44 (m, 2H), 4.10 (s, 3H), 2.79 (s, 3H), 1.68 (t, *J* = 7.1 Hz, 3H).

^{13}C {^{1}H} NMR (101 MHz, CD_3CN, 25 °C, δ): 168.63, 167.65, 146.92, 145.91, 145.68, 145.37, 143.75, 142.53, 132.76, 132.67 (q, *J* = 2.8 Hz), 132.45, 130.07, 128.73, 128.23, 126.73 (d, *J* = 2.8 Hz), 126.59, 103.83, 102.63, 69.93, 68.27, 60.60, 54.66 (q, *J* = 3.8 Hz), 51.19, 50.97, 38.26, 19.08, 14.61. The carbon directly attached to the boron atom was not detected due to quadrupolar broadening.

^{19}F {^{1}H} NMR (376 MHz, CD_3CN, 25 °C, δ): –141.66 (q, *J* = 40.6 Hz).

^{11}B NMR (128 MHz, CD_3CN, 25 °C, δ): 0.80 (q, *J* = 40.6 Hz).

HRMS ESI (m/z) calc'd for $C_{28}H_{30}N_5BNaO_5ClF_3^{+}$ [M+Na]$^{+}$, 642.1873; found, 642.1867. Deviation: –0.9 ppm.

Indomethacin derivative (4u)

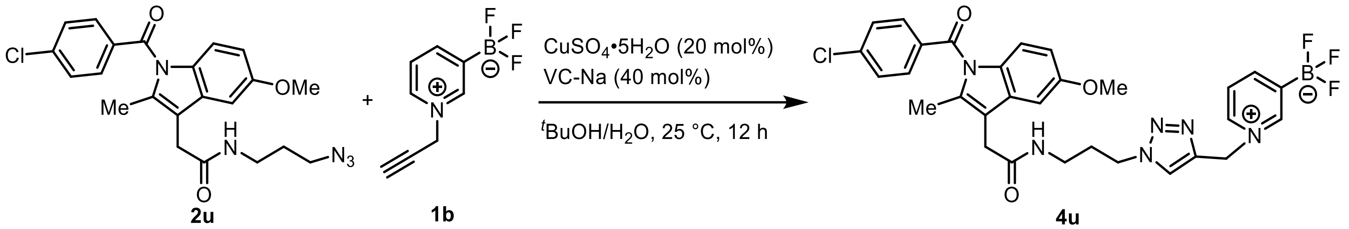

Under an ambient atmosphere, *N*-(3-azidopropyl)-2-(1-(4-chlorobenzoyl)-5-methoxy-2-methyl-1*H*-indol-3-yl)acetamide (198 mg, 0.450 mmol, 1.50 equiv.) in ^{t}BuOH:H$_2$O (1:1, 1.5 mL, 0.2 M) was to a 4 mL vial containing a magnetic stir bar. Subsequently, $CuSO_4 \cdot 5H_2O$ (15 mg, 60 μmol, 20 mol%), and sodium ascorbate (23.8 mg, 120 μmol, 40 mol%) were added was added into the tube, followed by 3-pyridinium trifluoroborate salt (55.5 mg, 0.300 mmol, 1.00 equiv.). The tube was sealed with a Teflon cap, and the reaction mixture was stirred vigorously at 25 °C. After the indicated time, the reaction vessel was opened to air, and the resulting mixture was quenched with saturated $NaHCO_3$ (10 mL), then extracted into EtOAc (3 × 10 mL). The combined organic phase was washed with brine, dried over Na_2SO_4, and concentrated by rotary evaporation. The residue was purified by chromatography on silica gel, eluting with dichloromethane/methanol (from 100:1 to 50:1, v:v) to afford 91.1 mg (49% yield) of the title compound as a pale yellow solid.

R*f* = 0.50 (dichloromethane/methanol, 10:1, v/v (UV))

NMR Spectroscopy:

^{1}H NMR (400 MHz, CD_3CN, 25 °C, δ): 8.69 (s, 1H), 8.53 (dt, *J* = 6.1, 1.6 Hz, 1H), 8.46 (d, *J* = 7.5 Hz, 1H), 7.98 (s, 1H), 7.78 (t, *J* = 6.8 Hz, 1H), 7.69 – 7.64 (m, 2H), 7.56 – 7.52 (m, 2H), 7.02 – 6.95 (m, 2H), 6.67 (dd, *J* = 9.0, 2.5 Hz, 1H), 6.56 (t, *J* = 5.5 Hz, 1H), 5.68 (s, 2H), 4.29 (t, *J* = 7.0 Hz, 2H), 3.76 (s, 3H), 3.54 (s, 2H), 3.12 (q, *J* = 6.5 Hz, 2H), 2.25 (s, 3H), 2.01 – 1.96 (m, 2H).

^{13}C {^{1}H} NMR (101 MHz, CD_3CN, 25 °C, δ): 171.10, 169.32, 157.09, 149.81, 146.68 (q, *J* = 1.6 Hz), 142.18, 140.85, 139.39, 137.06, 135.47, 132.13, 131.97, 131.90, 130.00, 127.82, 125.93, 116.06, 114.64, 112.49, 102.20, 56.26, 55.96, 48.73, 36.91, 32.44, 30.92, 13.80. The carbon directly attached to the boron atom was not detected due to quadrupolar broadening.

^{19}F {^{1}H} NMR (376 MHz, CD_3CN, 25 °C, δ): –144.08 (q, *J* = 45.2 Hz).

^{11}B NMR (128 MHz, CD_3CN, 25 °C, δ): 1.81 (q, *J* = 45.2 Hz).

HRMS ESI (m/z) calc'd for $C_{30}H_{29}N_6BClNaO_3F_3^+$ [M+Na]$^+$, 647.1927; found, 647.1936. Deviation: +1.4 ppm.

Indomethacin derivative (5u)

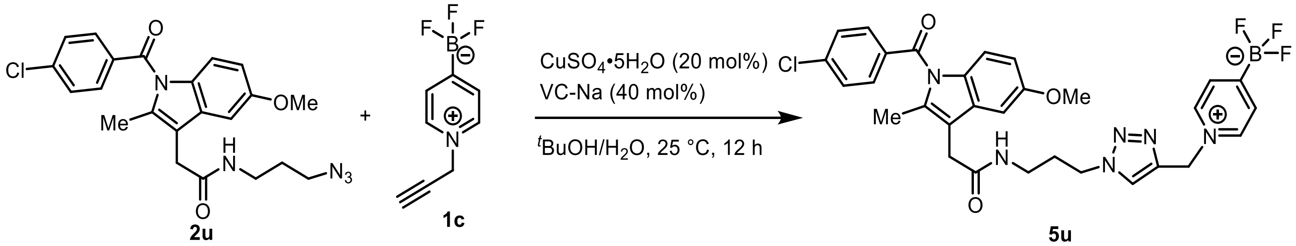

2u 1c 5u

Under an ambient atmosphere, *N*-(3-azidopropyl)-2-(1-(4-chlorobenzoyl)-5-methoxy-2-methyl-1*H*-indol-3-yl)acetamide (65.9 mg, 0.150 mmol, 1.50 equiv.) in ^{t}BuOH:H_2O (1:1, 0.5 mL, 0.2 M) was to a 4 mL vial containing a magnetic stir bar. Subsequently, $CuSO_4$·$5H_2O$ (5 mg, 20 μmol, 20 mol%), and sodium ascorbate (7.9 mg, 40 μmol, 40 mol%) were added was added into the tube, followed by 4-pyridinium trifluoroborate salt (18.5 mg, 0.100 mmol, 1.00 equiv.). The tube was sealed with a Teflon cap, and the reaction mixture was stirred vigorously at 25 °C. After the indicated time, the reaction vessel was opened to air, and the resulting mixture was concentrated by rotary evaporation. The residue was purified by chromatography on silica gel, eluting with dichloromethane/methanol (from 100:1 to 50:1, v:v) to afford 42.1 mg (67% yield) of the title compound as a pale yellow solid.

R*f* = 0.50 (dichloromethane/methanol, 10:1, v/v (UV))

NMR Spectroscopy:

^{1}H NMR (400 MHz, DMSO-*d*6, 25 °C, δ): 8.79 (d, *J* = 6.1 Hz, 2H), 8.29 (s, 1H), 8.12 (t, *J* = 5.7 Hz, 1H), 7.94 (d, *J* = 6.4 Hz, 2H), 7.73 – 7.65 (m, 2H), 7.67 – 7.60 (m, 2H), 7.10 (d, *J* = 2.6 Hz, 1H), 6.95 (d, *J* = 9.0 Hz, 1H), 6.70 (dd, *J* = 9.0, 2.5 Hz, 1H), 5.85 (s, 2H), 4.37 (t, *J* = 7.1 Hz, 2H), 3.74 (s, 3H), 3.51 (s, 2H), 3.07 (q, *J* = 6.5 Hz, 2H), 2.23 (s, 3H), 1.95 (p, *J* = 7.0 Hz, 2H).

^{13}C {^{1}H} NMR (101 MHz, DMSO-*d*6, 25 °C, δ): 169.64, 167.87, 155.57, 141.74, 140.37, 137.59, 135.20, 134.25, 131.17, 130.87, 130.30, 130.15 (q, *J* = 1.2 Hz), 129.05, 124.94, 114.60, 114.23, 111.27, 101.80, 55.43, 53.95, 47.49, 35.93, 31.17, 29.91, 13.39. The carbon directly attached to the boron atom was not detected due to quadrupolar broadening.

^{19}F {^{1}H} NMR (376 MHz, DMSO-*d*6, 25 °C, δ): –142.67 (brs).

^{11}B NMR (128 MHz, DMSO-*d*6, 25 °C, δ): 1.26 (brs).

HRMS ESI (m/z) calc'd for $C_{30}H_{29}N_6BClNaO_3F_3^+$ [M+Na]$^+$, 647.1927; found, 647.1934. Deviation: +1.1 ppm.

Preparation of starting materials

1-(2-Azido-5-fluorophenyl)ethanone (2c)

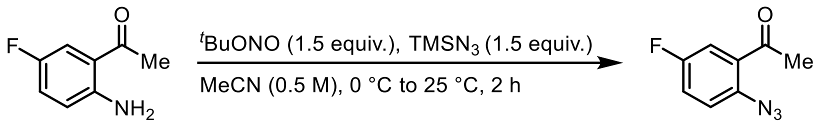

2c

Under an ambient atmosphere, 1-(2-amino-5-fluorophenyl)ethanone (1.53 g, 10.0 mmol) was dissolved in MeCN (20 mL) in a 100 mL round-bottomed flask, and cooled to 0 °C in an ice bath. To this stirred mixture was added ^{t}BuONO (1.55 g, 15 mmol), followed by $TMSN_3$ (1.73 g, 15 mmol) dropwise. The resulting solution was stirred at 25 °C for 2 h. The reaction mixture was concentrated by rotary evaporation, and the crude product was purified by chromatography on silica gel, eluting with hexanes/ethyl acetate (from 50:1 to 10:1, v:v) to afford 1.70 g (96% yield) of the title compound as a pale yellow solid.

R*f* = 0.60 (hexanes/ethyl acetate, 10:1, v/v (UV))

NMR Spectroscopy:

^{1}H NMR (400 MHz, $CDCl_3$, 25 °C, δ): 7.41 (dd, *J* = 8.9, 2.9 Hz, 1H), 7.28 – 7.10 (m, 2H), 2.63 (s, 3H).

^{13}C {^{1}H} NMR (101 MHz, $CDCl_3$, 25 °C, δ): 197.66 (d, *J* = 1.4 Hz), 159.52 (d, *J* = 246.5 Hz), 134.77 (d, *J* = 2.9 Hz), 132.33 (d, *J* = 6.0 Hz), 120.88 (d, *J* = 7.8 Hz), 120.24 (d, *J* = 23.6 Hz), 117.05 (d, *J* = 24.0 Hz), 31.21.

^{19}F {^{1}H} NMR (376 MHz, $CDCl_3$, 25 °C, δ): –116.78.

HRMS EI (m/z) calc'd for $C_8H_6FN_3O^+$ $[M]^+$, 179.0495; found, 179.0497. Deviation: +1.1 ppm.

Indomethacin azide derivative (2n)

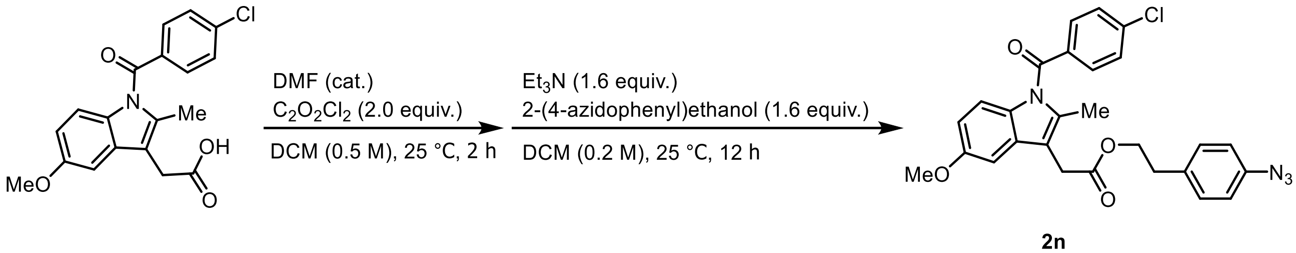

2n

Under an ambient atmosphere, a 100 mL round bottom flask equipped with a magnetic stir bar was charged with 2-(1-(4-chlorobenzoyl)-5-methoxy-2-methyl-1*H*-indol-3-yl)acetic acid (1.79 g, 5.00 mmol, 1.00 equiv.), followed by dry DCM (10 mL, *c* = 0.5 M).Subsequently, three drop of DMF followed by oxalyl chloride (0.840 mL, 1.27 g, 10.0 mmol, 2.00 equiv.) were added to the flask. The reaction was stirred at 25 °C until gas evolution ceased. The volatiles were removed by a rotary evaporator, then the residue was redissolved in dry CH_2Cl_2 (20 mL, *c* = 0.2 M). After that, 2-(4-azidophenyl)ethanol (1.31 g, 8.00 mmol, 1.60 equiv.), and Et_3N (1.15 mL, 0.809 g, 8.00 mmol, 1.60 equiv.) were added. The reaction was stirred at 25 °C for 12 h. The

reaction was quenched with 10 mL water, the layers were separated, and the aqueous laver was extracted with CH_2Cl_2 (3 × 10 mL). Then the combined organic lavers were dried over Na_2SO_4, and the solvent was removed in vacuo. The residue was then purified by flash column chromatography on silica gel, eluting with hexanes/ethyl acetate (from 20:1 to 5:1+ 1% Et_3N, v:v:v) to afford 1.90 g (72% yield) of the title compound as a white solid.

R*f* = 0.50 (hexanes/ethyl acetate, 5:1, v/v (UV))

NMR Spectroscopy:

^{1}H NMR (400 MHz, $CDCl_3$, 25 °C, δ): 7.70 – 7.61 (m, 2H), 7.50 – 7.45 (m, 2H), 7.08 – 6.99 (m, 2H), 6.91 – 6.87 (m, 2H), 6.85 – 6.81 (m, 2H), 6.69 (dd, *J* = 9.0, 2.6 Hz, 1H), 4.30 (t, *J* = 6.6 Hz, 2H), 3.81 (s, 3H), 3.63 (s, 2H), 2.87 (t, *J* = 6.6 Hz, 2H), 2.33 (s, 3H).

^{13}C {^{1}H} NMR (101 MHz, $CDCl_3$, 25 °C, δ): 170.83, 168.41, 156.20, 139.46, 138.44, 136.06, 134.49, 134.02, 131.31, 130.94, 130.77, 130.27, 129.29, 119.11, 115.07, 112.58, 111.68, 101.57, 65.32, 55.85, 34.52, 30.54, 13.45.

HRMS EI (m/z) calc'd for $C_{27}H_{23}ClN_4O_4^+$ $[M]^+$, 502.1408; found, 502.1407. Deviation: –0.2 ppm.

Dipeptide azide derivative (2r)

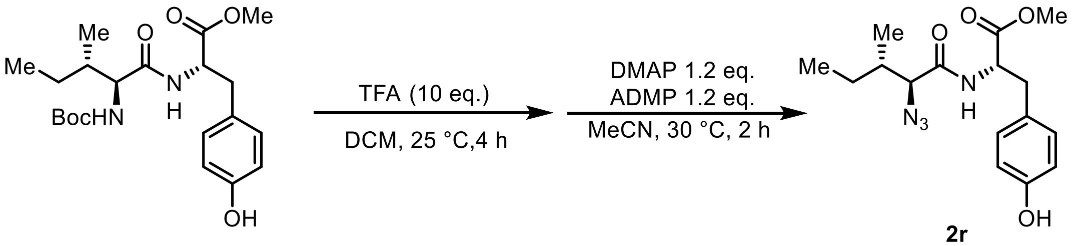

2r

Under an ambient atmosphere, methyl (*tert*-butoxycarbonyl)-*L*-isoleucyl-*L*-tyrosinate (408 mg, 1.00 mmol) was stirred in dichloromethane (5 mL) in an ice bath. To this was added trifluoroacetic acid (1.14 g, 10.0 mmol, 10.0 equiv.). The reaction mixture was stirred in an ice bath for 2 h. The reaction mixture was carefully neutralized with saturated $NaHCO_3$ in an ice bath until pH 8, then was concentrated under vacuum and the crude product was purified by chromatography on silica gel, eluting with dichloromethane/methanol (from 100:1 to 50:1, v:v) to afford 282.2 mg (69% yield) of methyl *L*-isoleucyltyrosinate as a pale yellow oil. Then 2-azido-1,3-dimethylimidazolinium hexafluorophosphate (228 mg, 0.800 mmol, 1.20 equiv.) was added to a solution of methyl *L*-isoleucyltyrosinate (203 mg, 0.660 mmol, 1.00 equiv.), and DMAP (97.6 mg, 0.800 mol, 1.20 equiv.) in CH_3CN (5.0 mL) at room temperature. The mixture was stirred for 2 h at 25 °C. The reaction was quenched with $NaHCO_3$ aqueous solution (20 mL), and the organic materials were extracted with CH_2Cl_2 (3 × 15 mL). The combined extracts were washed with brine (30 mL) and then dried with anhydrous Na_2SO_4. was concentrated by rotary evaporation. The residue was purified by chromatography on silica gel, eluting with dichloromethane/methanol (from 100:1 to 50:1, v:v) to afford 148.1 mg (67% yield) of the title compound as a pale yellow oil.

R$_f$ = 0.70 (dichloromethane/methanol, 10:1, v/v (UV))

NMR Spectroscopy:

^{1}H NMR (400 MHz, DMSO-d_6, 25 °C, δ): 9.22 (s, 1H), 8.58 (d, *J* = 7.6 Hz, 1H), 7.00 (d, *J* = 8.4 Hz, 2H), 6.65 (d, *J* = 8.5 Hz, 2H), 4.51 – 4.40 (m, 1H), 3.59 (s, 3H), 3.53 (d, *J* = 8.4 Hz, 1H), 2.94 (dd, *J* = 13.9, 5.7 Hz, 1H), 2.83 (dd, *J* = 13.9, 9.2 Hz, 1H), 1.87 – 1.74 (m, 1H), 1.45 (ddt, *J* = 21.0, 10.8, 3.4 Hz, 1H), 1.12 (ddd, *J* = 13.4, 8.6, 7.1 Hz, 1H), 0.83 (dt, *J* = 7.4, 4.0 Hz, 6H).

^{13}C {^{1}H} NMR (101 MHz, DMSO-d_6, 25 °C, δ): 171.70, 169.24, 156.06, 129.95, 126.87, 115.05, 65.78, 53.84, 51.85, 35.81, 35.69, 24.50, 14.96, 10.45.

HRMS EI (m/z) calc'd for $C_{16}H_{22}N_4O_4^+$ [M]$^+$, 334.1641; found, 334.1637. Deviation: –1.2 ppm.

Indomethacin azide derivative (2u)

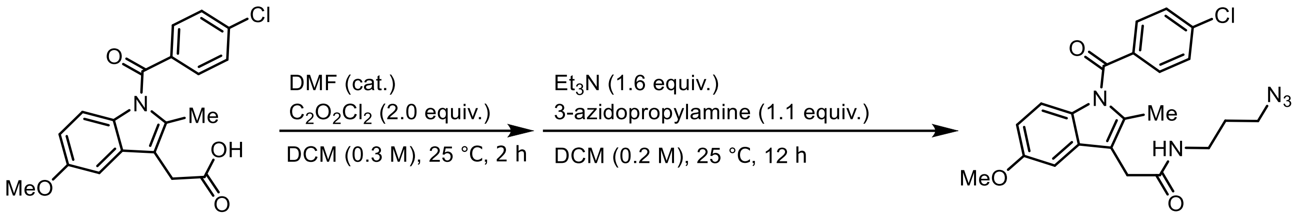

2u

Under an ambient atmosphere, a 100 mL round bottom flask equipped with a magnetic stir bar was charged with 2-(1-(4-chlorobenzoyl)-5-methoxy-2-methyl-1*H*-indol-3-yl)acetic acid (1.07g, 3.00 mmol, 1.00 equiv.), followed by dry DCM (10 mL, *c* = 0.30 M).Subsequently, two drop of DMF followed by oxalyl chloride (0.51 mL, 0.76 g, 6.00 mmol, 2.00 equiv.) were added to the flask. The reaction was stirred at 25 °C until gas evolution ceased. The volatiles were removed by a rotary evaporator, then the residue was redissolved in dry CH_2Cl_2 (15 mL, *c* = 0.20 M). After that, 3-azidopropylamine (0.32 mL, 0.33 g, 3.30 mmol, 1.10 equiv.), and Et_3N (0.67 mL, 0.49 g, 4.80 mmol, 1.60 equiv.) were added. The reaction was stirred at 25 °C for 12 h. The reaction was quenched with 10 mL water, the layers were separated, and the aqueous laver was extracted with CH_2Cl_2 (3 × 10 mL). Then the combined organic lavers were dried over Na_2SO_4, and the solvent was removed in vacuo. The residue was then purified by flash column chromatography on silica gel, eluting with hexanes/ethyl acetate (from 10:1 to 5:1, v:v) to afford 1.02 g (77% yield) of the title compound as a pale yellow solid.

R$_f$ = 0.50 (hexanes/ethyl acetate, 5:1, v/v (UV))

NMR Spectroscopy:

^{1}H NMR (400 MHz, $CDCl_3$, 25 °C, δ): 7.63 (d, *J* = 8.5 Hz, 2H), 7.47 (d, *J* = 8.5 Hz, 2H), 6.87 (d, *J* = 2.5 Hz, 1H), 6.84 (d, *J* = 9.0 Hz, 1H), 6.69 (dd, *J* = 9.0, 2.5 Hz, 1H), 5.89 (s, 1H), 3.81 (s, 3H), 3.64 (s, 2H), 3.28 (q, *J* = 6.5 Hz, 2H), 3.25 (t, *J* = 6.5 Hz, 2H) 2.39 (s, 3H), 1.69 (m, 2H).

^{13}C {^{1}H} NMR (101 MHz, $CDCl_3$, 25 °C, δ): 170.14, 168.33, 156.33, 139.53, 136.42, 133.56, 131.13, 130.92, 130.38, 129.21, 115.16, 112.83, 112.20, 100.92, 55.77, 49.41, 37.41, 32.19, 28.61, 13.29.

HRMS ESI (m/z) calc'd for $C_{22}H_{23}ClN_5O_3^+$ [M+H]$^+$, 440.1484; found, 440.1483. Deviation: –0.2 ppm.

^{18}F-^{19}F isotope exchange of of the conjugates

General Information

[^{18}F]Fluoride was produced by cyclotron via the $^{18}O(p,n)\,^{18}F$ reaction and delivered as [^{18}F]fluoride in [^{18}O]H_2O. Radiosynthesis and azeotropic drying was performed on a Reacti-Therm (Thermo Scientific, USA). Analytical high-performance liquid chromatography (HPLC) system consists of 1525 binary HPLC pump, 2998 photodiode array detector (Waters, USA), and Sockel 2" GABI Nova with mild energy probe (Elysia Raytest, Germany). The radio-TLC was used to evaluate the radiochemical conversion (RCC). ^{18}F-radiolabeled compound was characterized by comparing the radio-HPLC trace with the HPLC UV-trace of an authentic reference sample. HPLC method: analytical C18 column (5 µm, 4.6×150 mm, Phenomenex); mobile phase: A = MeCN, B = H_2O, A/B = 3/7; flow rate 1.0 mL/min; detection wavelength is 220 nm or 254 nm.

[^{18}F](1-((1-Benzyl-1*H*-1,2,3-triazol-4-yl)methyl)pyridin-1-ium-3-yl)trifluoroborate ([^{18}F]4a)

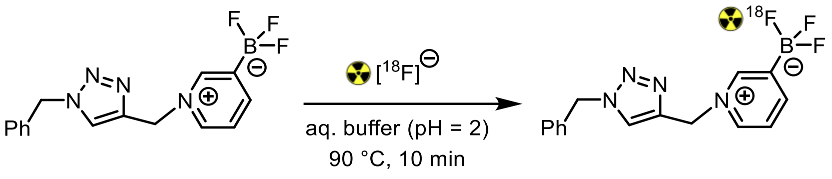

4a **[^{18}F]4a**, RCC = 34 ± 1%

No-carrier-added (NCA) [^{18}F]fluoride ion (10–12 mCi) was trapped onto a QMA cartridge, and further eluted with aqueous pyridazine-HCl buffer (1 mL, pH = 2.0) into an EP tube to obtain a [^{18}F]fluoride solution (10–12 µCi/µL). For labeling, precursor (**4a**, 300 nmol) in DMF (20 µL) was added in an EP tube, followed by the addition of the above [^{18}F]fluoride solution (40 µL). The mixture was placed in a heating block at 90 °C for 10 min. Then the reaction was quenched by addition of MeCN/H_2O (3:2, 500 µL). An aliquot was removed for analysis by radio-TLC and radio-HPLC for product identity and calculation of radiochemical conversion (RCC). The product identity was determined by comparison of the HPLC radio-trace with the HPLC UV-trace of the authentic reference sample Bn-PyBF$_3$ conjugate (**4a**).

Radio-TLC

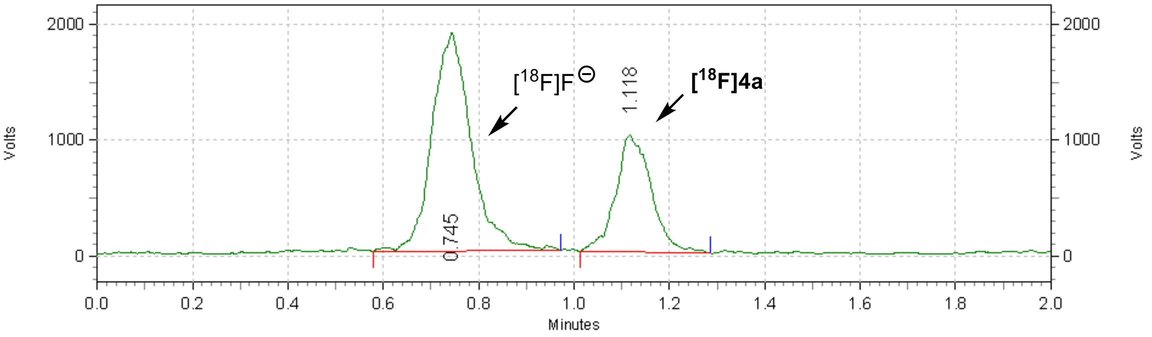

检 测 器 1
Results

保 留 时 间	面 积	面 积 百 分 比	峰 高	高 度 百 分 比
0.745	10779348	67.350	1877638	65.026
1.118	5225724	32.650	1009896	34.974
Totals	16005072	100.000	2887534	100.000

UV-220 nm

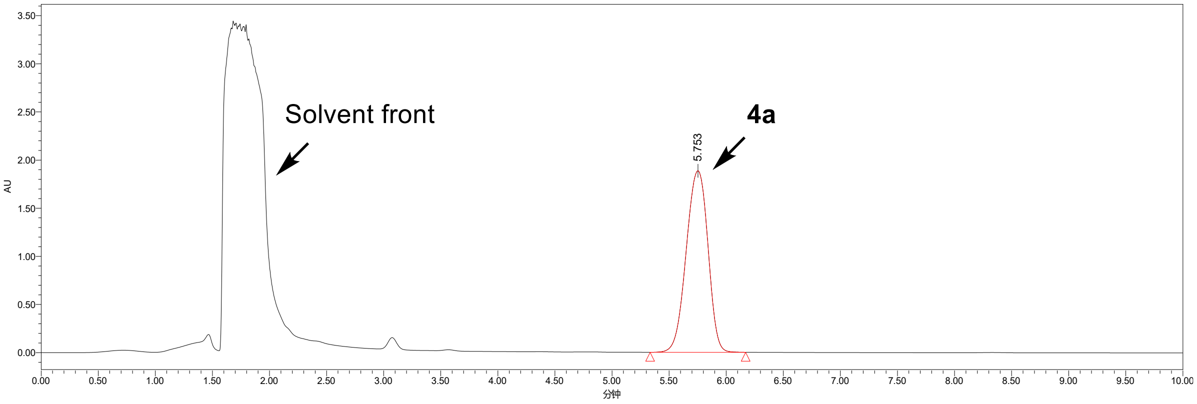

Radio-HPLC-test

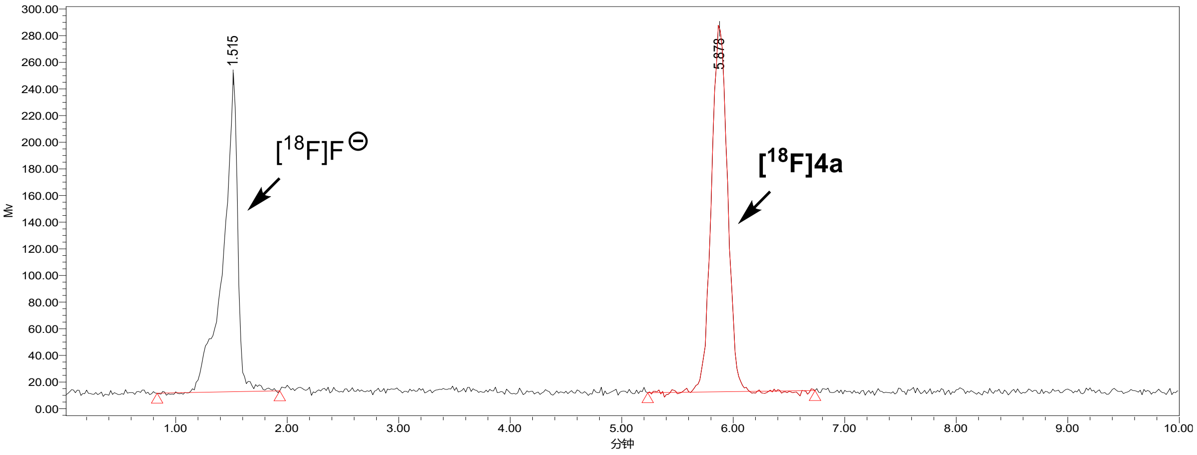

Figure S6. Radio-TLC and radio-HPLC trace of the ^{18}F-^{19}F isotope exchange reaction yielding **[^{18}F]4a**.

Entry	rTLC RCC (%)	rHPLC RCP (%)	RCC × RCP (%)
1	33	> 99	33
2	36	> 99	36
3	33	> 99	33
	Mean ± SD		34 ± 1

rTLC run in MeOH:DCM (1:10, v/v)

[18F](1-((1-Benzyl-1*H*-1,2,3-triazol-4-yl)methyl)pyridin-1-ium-4-yl)trifluoroborate ([18F]5a)

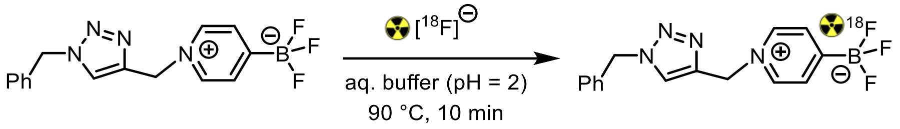

5a **[18F]5a**, RCC = 36 ± 2%

No-carrier-added (NCA) [18F]fluoride ion (10–12 mCi) was trapped onto a QMA cartridge, and further eluted with aqueous pyridazine-HCl buffer (1 mL, pH = 2.0) into an EP tube to obtain a [18F]fluoride solution (10–12 μCi/μL). For labeling, precursor (**5a**, 300 nmol) in DMF (20 μL) was added in an EP tube, followed by the addition of the above [18F]fluoride solution (40 μL). The mixture was placed in a heating block at 90 °C for 10 min. Then the reaction was quenched by addition of MeCN/H_2O (3:2, 500 μL). An aliquot was removed for analysis by radio-TLC and radio-HPLC for product identity and calculation of radiochemical conversion (RCC). The product identity was determined by comparison of the HPLC radio-trace with the HPLC UV-trace of the authentic reference sample Bn-PyBF_3 conjugate (**5a**).

Radio-TLC

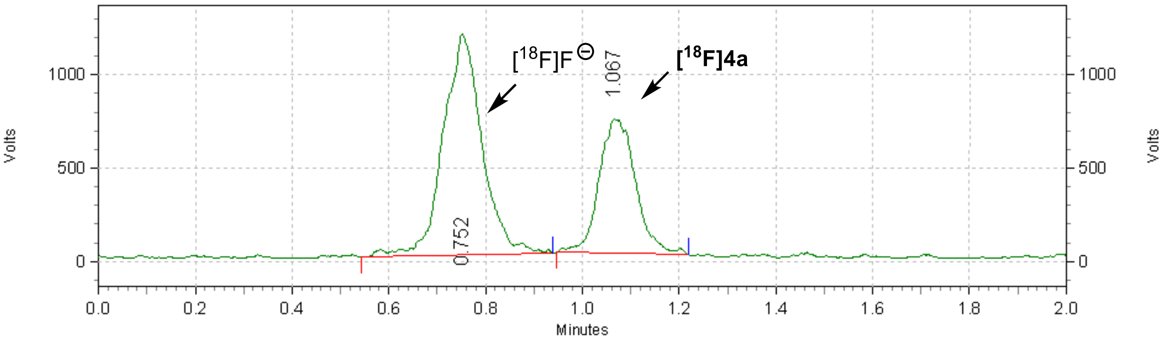

检 测 器 1
Results

保留时间	面积	面积百分比	峰高	高度百分比
0.752	6640670	63.626	1184082	62.149
1.067	3796446	36.374	721149	37.851
Totals	10437116	100.000	1905231	100.000

UV-220 nm

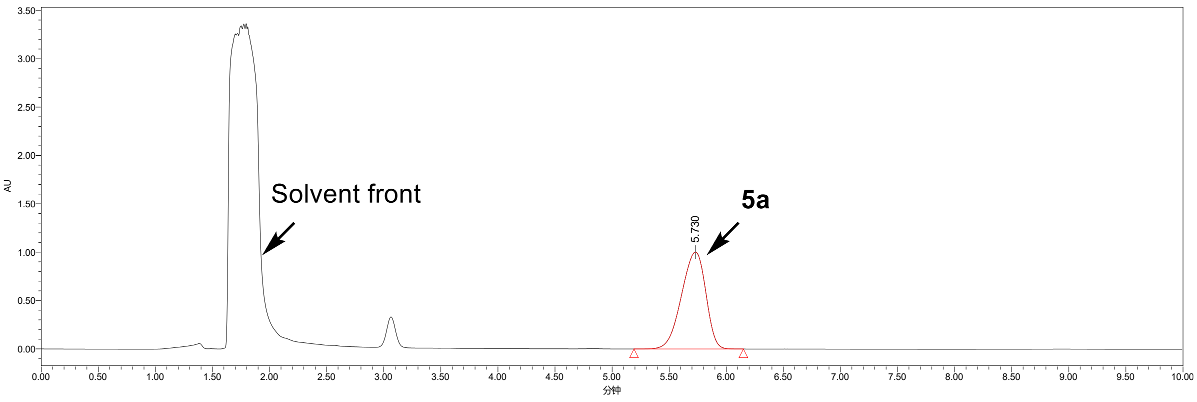

Radio-HPLC-test

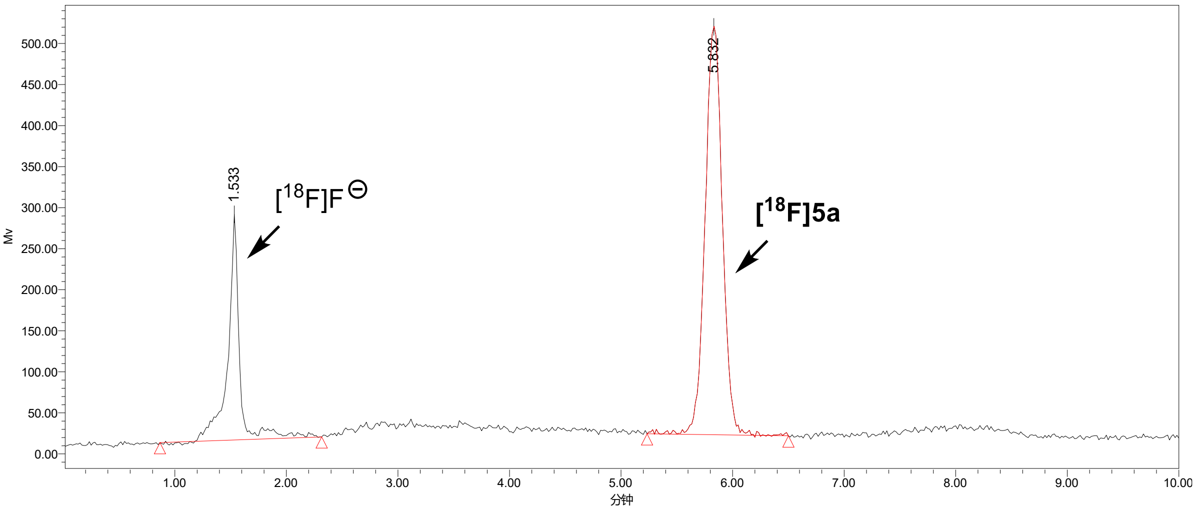

Figure S7. Radio-TLC and radio-HPLC trace of the ^{18}F-^{19}F isotope exchange reaction yielding **[^{18}F]5a**.

Entry	rTLC RCC (%)	rHPLC RCP (%)	RCC × RCP (%)
1	36	> 99	36
2	38	> 99	38
3	33	> 99	33
	Mean ± SD		36 ± 2

rTLC run in MeOH:DCM (1:10, v/v)

[18F](1-(2-(1-Benzyl-1*H*-1,2,3-triazol-4-yl)ethyl)pyridin-1-ium-3-yl)trifluoroborate ([18F]6a)

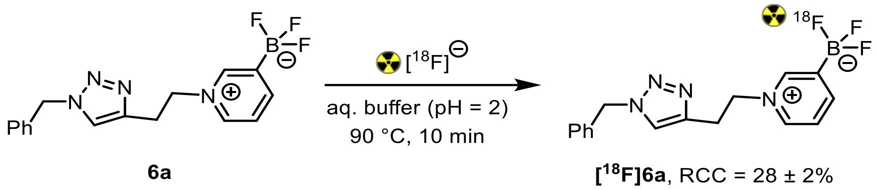

No-carrier-added (NCA) [18F]fluoride ion (10–12 mCi) was trapped onto a QMA cartridge, and further eluted with aqueous pyridazine-HCl buffer (1 mL, pH = 2.0) into an EP tube to obtain a [18F]fluoride solution (10–12 μCi/μL). For labeling, precursor (**6a**, 300 nmol) in DMF (20 μL) was added in an EP tube, followed by the addition of the above [18F]fluoride solution (40 μL). The mixture was placed in a heating block at 90 °C for 10 min. Then the reaction was quenched by addition of MeCN/H_2O (3:2, 500 μL). An aliquot was removed for analysis by radio-TLC and raido-HPLC for product identity and calculation of radiochemical conversion (RCC). The product identity was determined by comparison of the HPLC radio-trace with the HPLC UV-trace of the authentic reference sample Bn-PyBF_3 conjugate (**6a**).

Radio-TLC

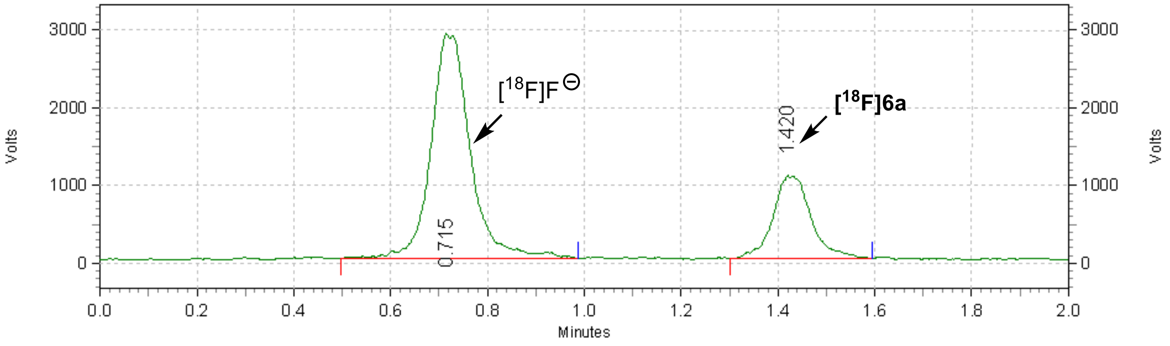

检 测 器 1
Results

保留时间	面积	面积百分比	峰高	高度百分比
0.715	16383817	73.838	2896232	73.062
1.420	5804951	26.162	1067860	26.938
Totals	22188768	100.000	3964092	100.000

UV-220 nm

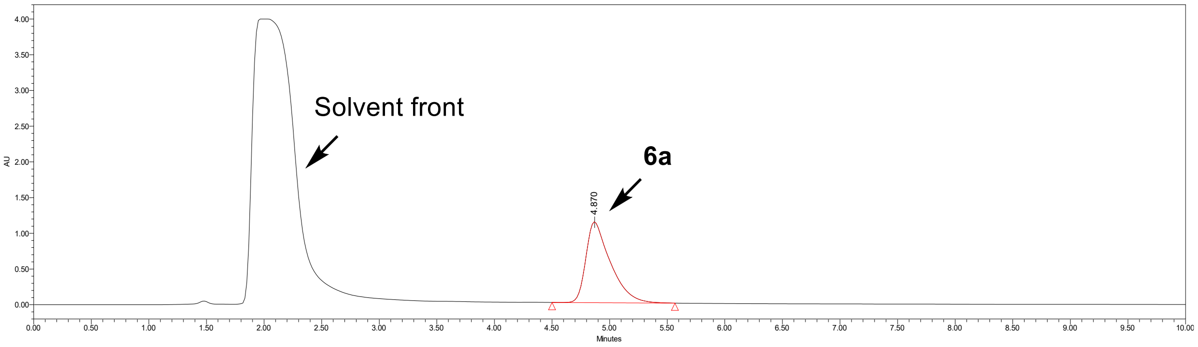

Radio-HPLC-test

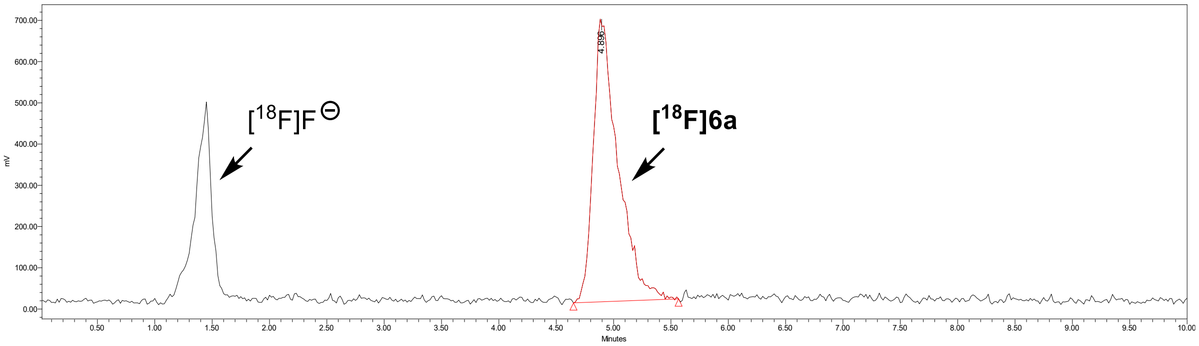

Figure S8. Radio-TLC and radio-HPLC trace of the ^{18}F-^{19}F isotope exchange reaction yielding **[^{18}F]6a**.

Entry	rTLC RCC (%)	rHPLC RCP (%)	RCC × RCP (%)
1	26	> 99	26
2	30	> 99	30
3	27	> 99	27
	Mean ± SD		28 ± 2

rTLC run in MeOH:DCM (1:10, v/v)

[^{18}F](1-(2-(1-Benzyl-1*H*-1,2,3-triazol-4-yl)ethyl)-5-chloropyridin-1-ium-3-yl)trifluoroborate ([^{18}F]7a)

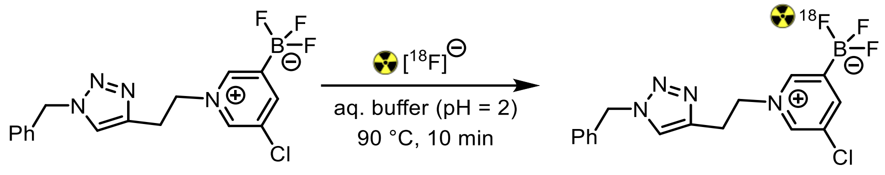

No-carrier-added (NCA) [^{18}F]fluoride ion (10–12 mCi) was trapped onto a QMA cartridge, and further eluted with aqueous pyridazine-HCl buffer (1 mL, pH = 2.0) into an EP tube to obtain a [^{18}F]fluoride solution (10–12 μCi/μL). For labeling, precursor (**7a**, 300 nmol) in DMF (20 μL) was added in an EP tube, followed by the addition of the above [^{18}F]fluoride solution (40 μL). The mixture was placed in a heating block at 90 °C for 10 min. Then the reaction was quenched by addition of MeCN/H_2O (3:2, 500 μL). An aliquot was removed for analysis by radio-TLC and raido-HPLC for product identity and calculation of radiochemical conversion (RCC). The product identity was determined by comparison of the HPLC radio-trace with the HPLC UV-trace of the authentic reference sample Bn-PyBF_3 conjugate (**7a**).

Radio-TLC

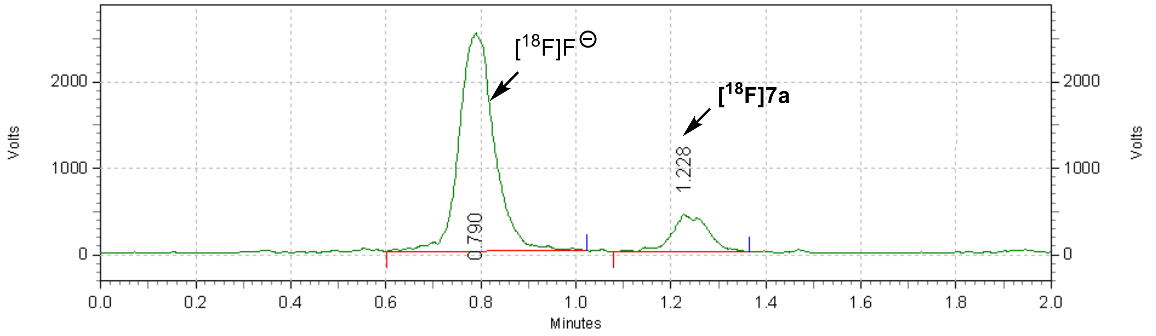

检 测 器 1

Results

保留时间	面积	面积百分比	峰高	高度百分比
0.790	12952375	84.540	2521094	85.213
1.228	2368681	15.460	437497	14.787
Totals	15321056	100.000	2958591	100.000

UV-220 nm

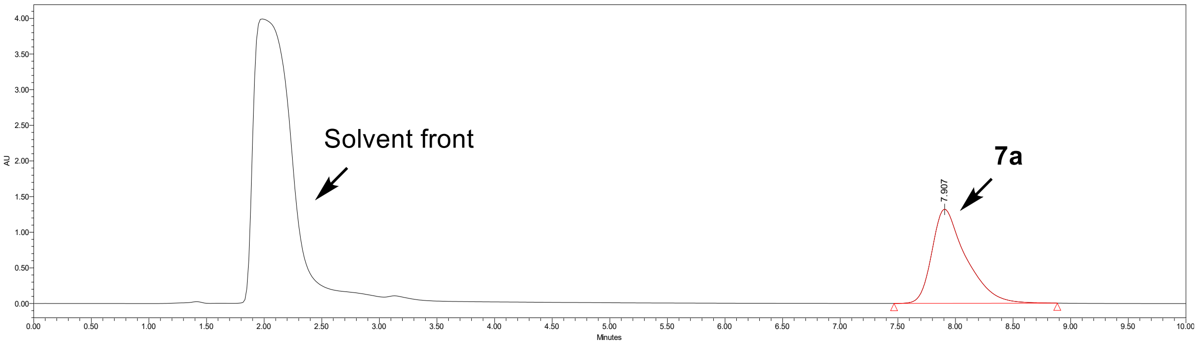

Radio-HPLC-test

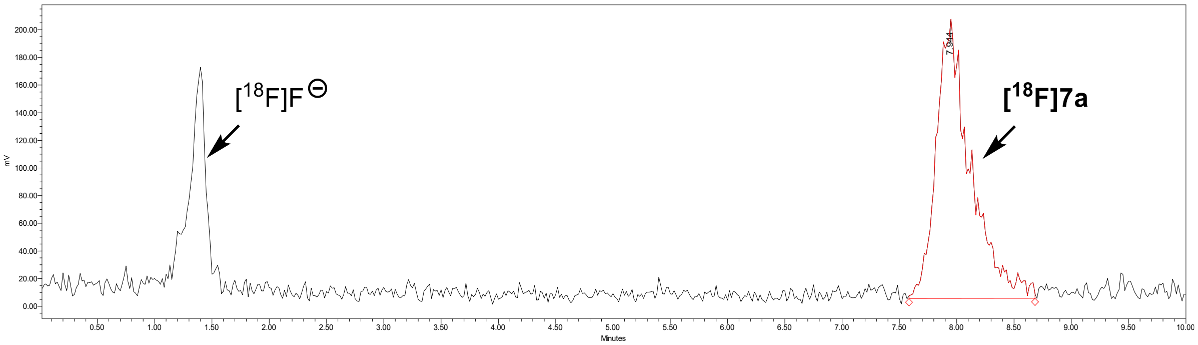

Figure S9. Radio-TLC and radio-HPLC trace of the ^{18}F-^{19}F isotope exchange reaction yielding **[^{18}F]7a**.

Entry	rTLC RCC (%)	rHPLC RCP (%)	RCC × RCP (%)
1	15	> 99	15
2	18	> 99	18
3	13	> 99	13
	Mean ± SD		15 ± 2

rTLC run in MeOH:DCM (1:10, v/v)

[^{18}F](1-(2-(1-Benzyl-1*H*-1,2,3-triazol-4-yl)ethyl)-5-fluoropyridin-1-ium-3-yl)trifluoroborate ([^{18}F]8a)

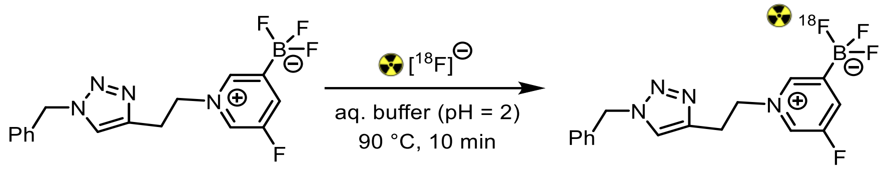

No-carrier-added (NCA) [^{18}F]fluoride ion (10–12 mCi) was trapped onto a QMA cartridge, and further eluted with aqueous pyridazine-HCl buffer (1 mL, pH = 2.0) into an EP tube to obtain a [^{18}F]fluoride solution (10–12 µCi/µL). For labeling, precursor (**8a**, 300 nmol) in DMF (20 µL) was added in an EP tube, followed by the addition of the above [^{18}F]fluoride solution (40 µL). The mixture was placed in a heating block at 90 ^{o}C for 10 min. Then the reaction was quenched by addition of MeCN/H_2O (3:2, 500 µL). An aliquot was removed for analysis by radio-TLC and raido-HPLC for product identity and calculation of radiochemical conversion (RCC). The product identity was determined by comparison of the HPLC radio-trace with the HPLC UV-trace of the authentic reference sample Bn-PyBF_3 conjugate (**8a**).

Radio-TLC

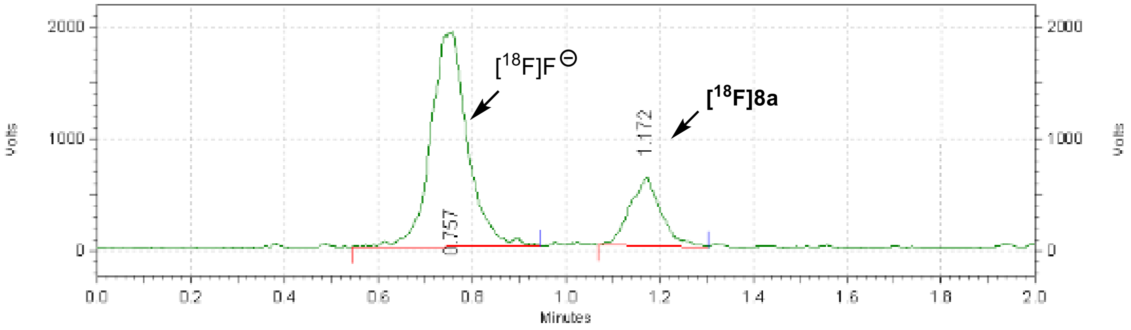

检 测 器 1
Results

保留时间	面积	面积百分比	峰高	高度百分比
0.757	10508947	79.034	1920910	75.844
1.172	2787817	20.966	611812	24.156
Totals	13296764	100.000	2532722	100.000

UV-220 nm

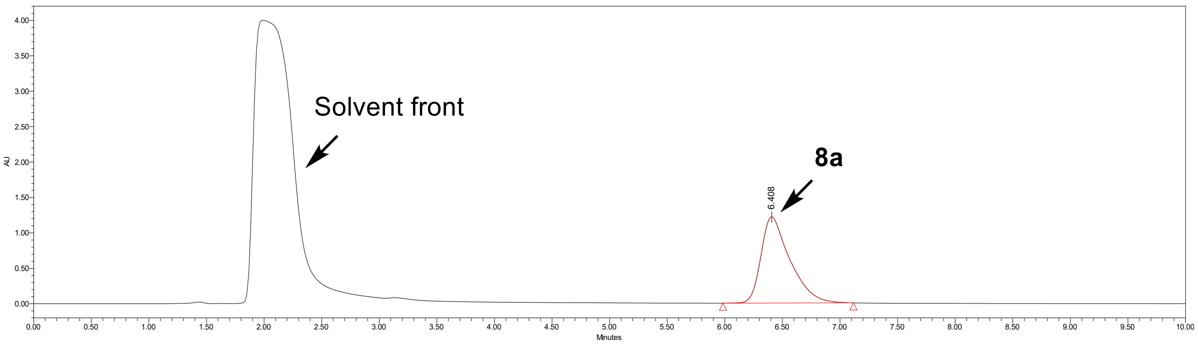

Radio-HPLC-test

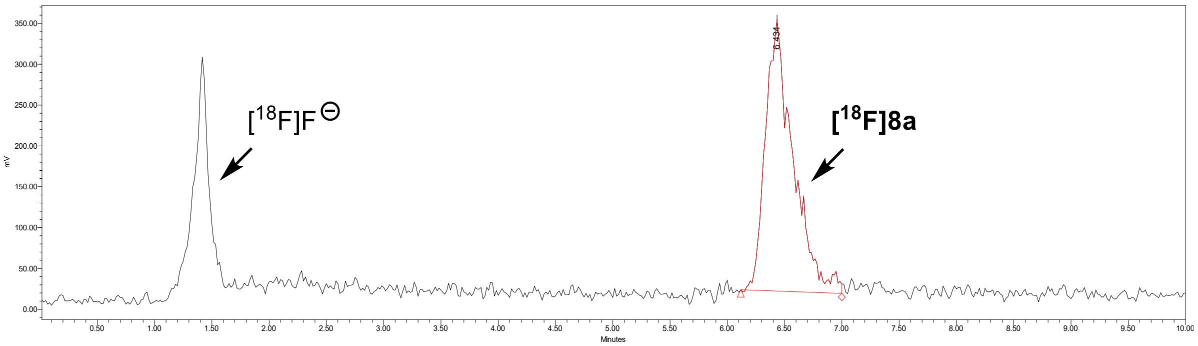

Figure S10. Radio-TLC and radio-HPLC trace of the ^{18}F-^{19}F isotope exchange reaction yielding **[^{18}F]8a**.

Entry	rTLC RCC (%)	rHPLC RCP (%)	RCC × RCP (%)
1	21	> 99	21
2	25	> 99	25
3	22	> 99	22
	Mean ± SD		23 ± 2

rTLC run in MeOH:DCM (1:10, v/v)

[^{18}F] (1-(2-(1-Benzyl-1*H*-1,2,3-triazol-4-yl)ethyl)pyridin-1-ium-4-yl)trifluoroborate ([^{18}F]9a)

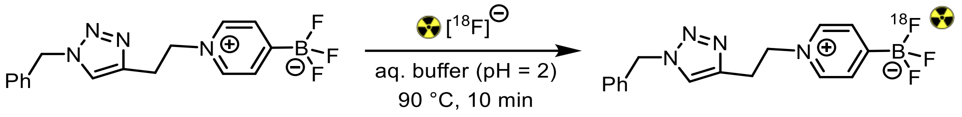

9a **[^{18}F]9a**, RCC = 38 ± 1%

No-carrier-added (NCA) [^{18}F]fluoride ion (10–12 mCi) was trapped onto a QMA cartridge, and further eluted with aqueous pyridazine-HCl buffer (1 mL, pH = 2.0) into an EP tube to obtain a [^{18}F]fluoride solution (10–12 μCi/μL). For labeling, precursor (**9a**, 300 nmol) in DMF (20 μL) was added in an EP tube, followed by the addition of the above [^{18}F]fluoride solution (40 μL). The mixture was placed in a heating block at 90 °C for 10 min. Then the reaction was quenched by addition of MeCN/H_2O (3:2, 500 μL). An aliquot was removed for analysis by radio-TLC and raido-HPLC for product identity and calculation of radiochemical conversion (RCC). The product identity was determined by comparison of the HPLC radio-trace with the HPLC UV-trace of the authentic reference sample Bn-PyBF$_3$ conjugate (**9a**).

Radio-TLC

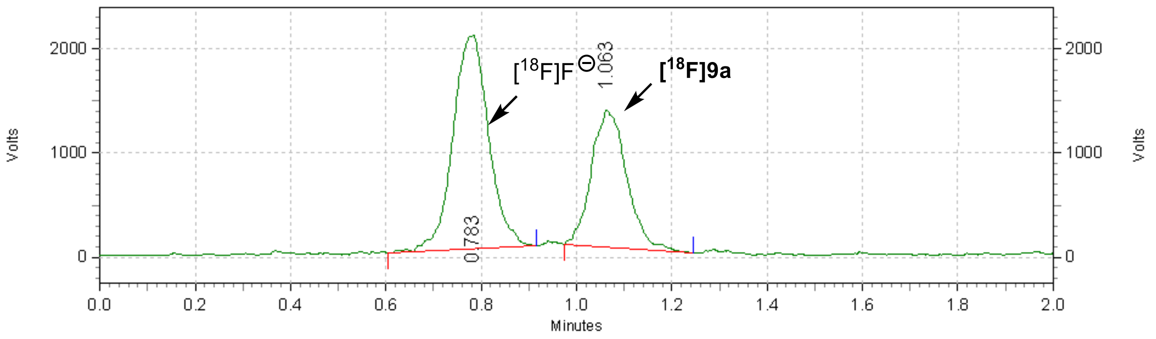

检 测 器 1

Results

保留时间	面积	面积百分比	峰高	高度百分比
0.783	10754441	61.304	2048703	60.822
1.063	6788359	38.696	1319674	39.178
Totals	17542800	100.000	3368377	100.000

UV-220 nm

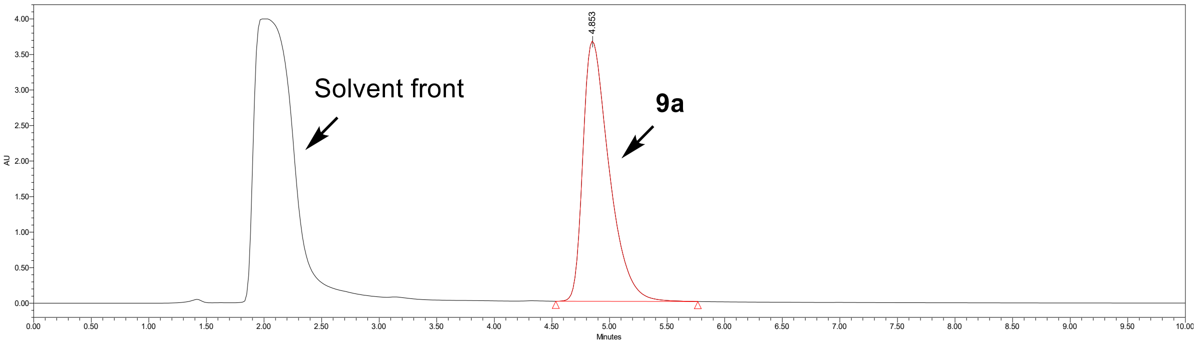

Radio-HPLC-test

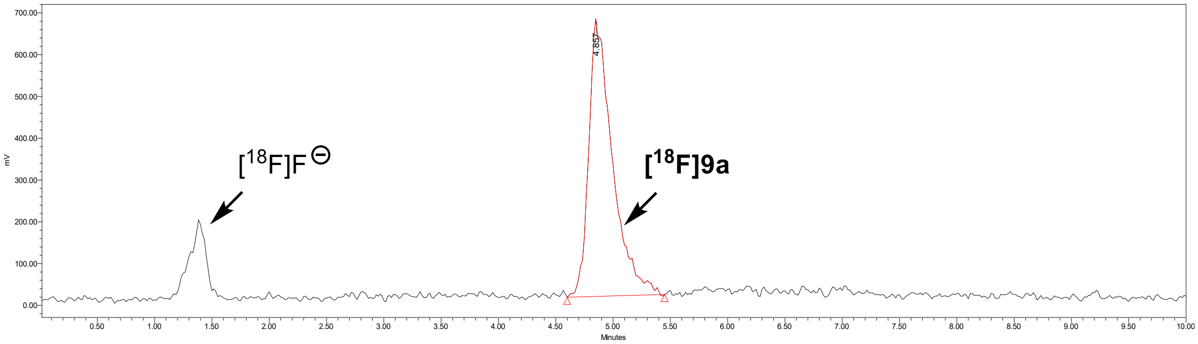

Figure S11. Radio-TLC and radio-HPLC trace of the ^{18}F-^{19}F isotope exchange reaction yielding **[^{18}F]9a**.

Entry	rTLC RCC (%)	rHPLC RCP (%)	RCC × RCP (%)
1	39	> 99	39
2	36	> 99	36
3	38	> 99	38
	Mean ± SD		38 ± 1

rTLC run in MeOH:DCM (1:10, v/v)

[^{18}F](1-(2-(2-(2-(4-Phenethyl-1*H*-1,2,3-triazol-1-yl)ethoxy)ethoxy)ethyl)pyridin-1-ium-3-yl)trifluoroborate ([^{18}F]10a)

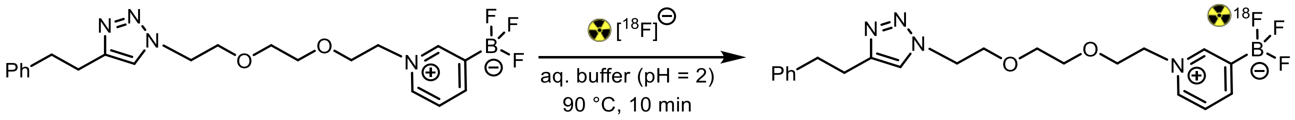

10a **[^{18}F]10a**, RCC = 47 ± 2%

No-carrier-added (NCA) [^{18}F]fluoride ion (10–12 mCi) was trapped onto a QMA cartridge, and further eluted with aqueous pyridazine-HCl buffer (1 mL, pH = 2.0) into an EP tube to obtain a [^{18}F]fluoride solution (10–12 μCi/μL). For labeling, precursor (**10a**, 300 nmol) in DMF (20 μL) was added in an EP tube, followed by the addition of the above [^{18}F]fluoride solution (40 μL). The mixture was placed in a heating block at 90 °C for 10 min. Then the reaction was quenched by addition of MeCN/H_2O (3:2, 500 μL). An aliquot was removed for analysis by radio-TLC and raido-HPLC for product identity and calculation of radiochemical conversion (RCC). The product identity was determined by comparison of the HPLC radio-trace with the HPLC UV-trace of the authentic reference sample (**10a**).

Radio-TLC

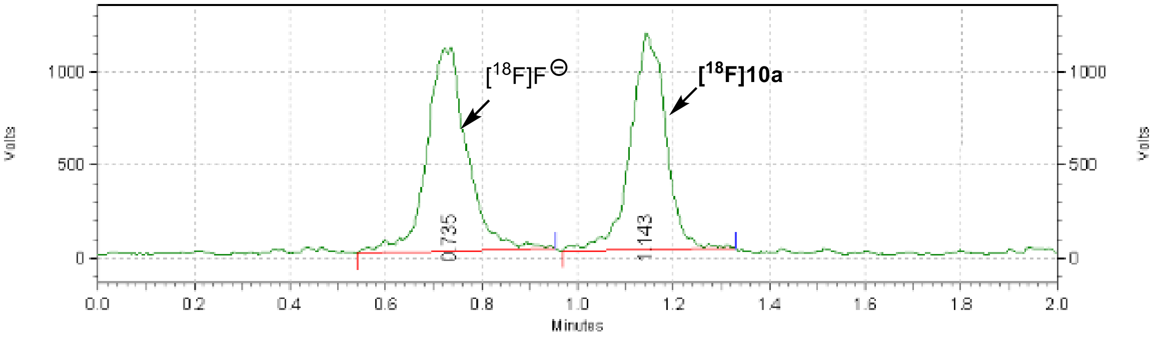

检测器 1
Results

保留时间	面积	面积百分比	峰高	高度百分比
0.735	6432547	51.961	1096340	48.530
1.143	5947028	48.039	1162774	51.470
Totals	12379575	100.000	2259114	100.000

UV-220 nm

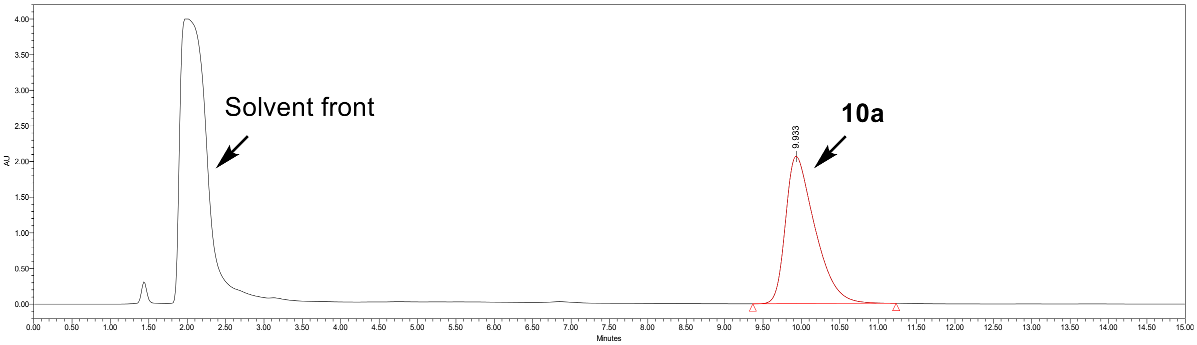

Radio-HPLC-test

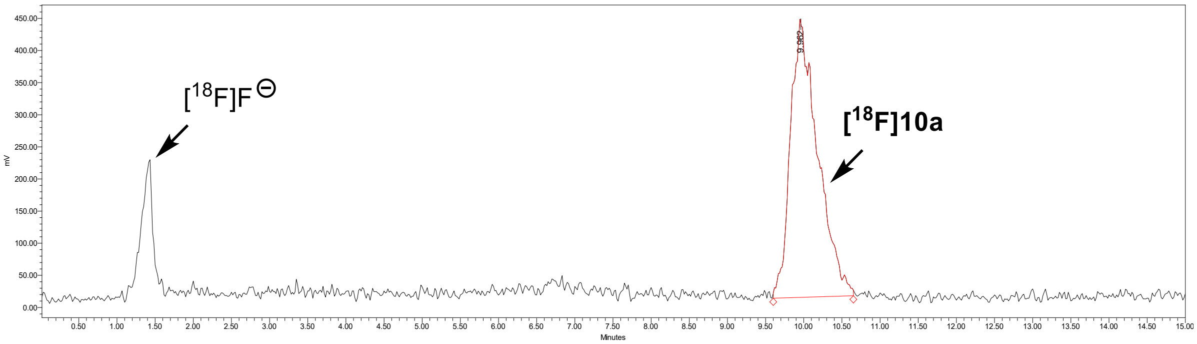

Figure S12. Radio-TLC and radio-HPLC trace of the ^{18}F-^{19}F isotope exchange reaction yielding **[18F]10a**.

Entry	rTLC RCC (%)	rHPLC RCP (%)	RCC × RCP (%)
1	48	> 99	48
2	45	> 99	45
3	49	> 99	49
	Mean ± SD		47 ± 2

rTLC run in MeOH:DCM (1:10, v/v)

[^{18}F](1-(2-(1-Benzyl-1*H*-1,2,3-triazol-4-yl)ethyl)quinolin-1-ium-3-yl)trifluoroborate ([^{18}F]11a)

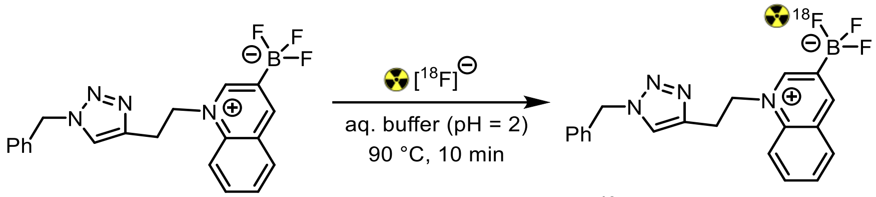

No-carrier-added (NCA) [^{18}F]fluoride ion (10–12 mCi) was trapped onto a QMA cartridge, and further eluted with aqueous pyridazine-HCl buffer (1 mL, pH = 2.0) into an EP tube to obtain a [^{18}F]fluoride solution (10–12 μCi/μL). For labeling, precursor (**11a**, 300 nmol) in DMF (20 μL) was added in an EP tube, followed by the addition of the above [^{18}F]fluoride solution (40 μL). The mixture was placed in a heating block at 90 ^{o}C for 10 min. Then the reaction was quenched by addition of MeCN/H_2O (3:2, 500 μL). An aliquot was removed for analysis by radio-TLC and raido-HPLC for product identity and calculation of radiochemical conversion (RCC). The product identity was determined by comparison of the HPLC radio-trace with the HPLC UV-trace of the authentic reference sample Bn-PyBF$_3$ conjugate (**11a**).

Radio-TLC

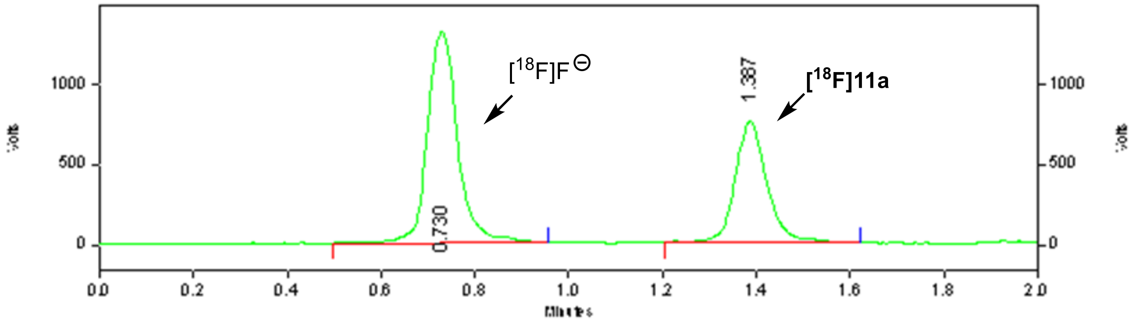

检测器 1
Results

保留时间	面积	面积百分比	峰高	高度百分比
0.730	5841285	61.926	1310254	63.454
1.387	3591379	38.074	754629	36.546
Totals	9432664	100.000	2064883	100.000

UV-254 nm

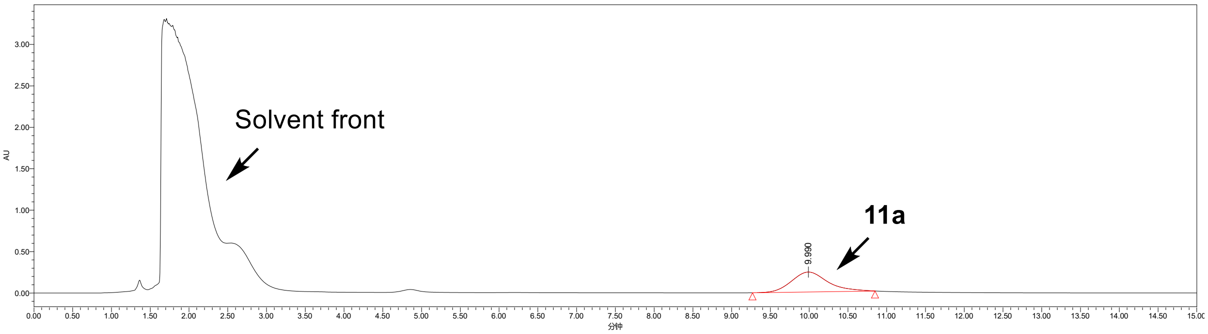

Radio-HPLC-test

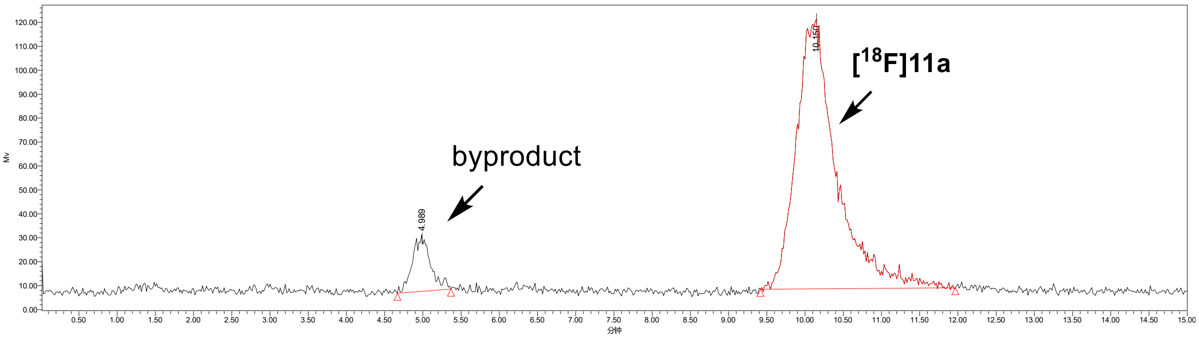

Figure S13. Radio-TLC and radio-HPLC trace of the ^{18}F-^{19}F isotope exchange reaction yielding **[^{18}F]11a**.

Entry	rTLC RCC (%)	rHPLC RCP (%)	RCC × RCP (%)
1	38	91	34
2	36	92	36
3	41	91	37
	Mean ± SD		35 ± 1

rTLC run in MeOH:DCM (1:10, v/v)

[18F](1-(2-(1-Benzyl-1*H*-1,2,3-triazol-4-yl)ethyl)-5-methoxypyridin-1-ium-3-yl)trifluoroborate ([18F]12a)

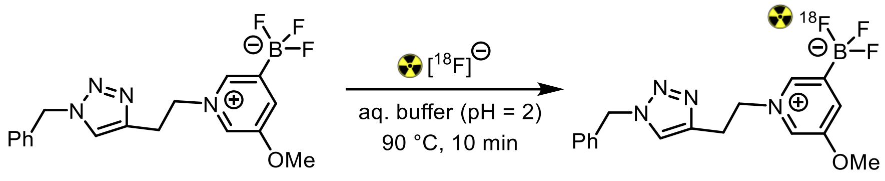

12a **[18F]12a**, RCC = 39 ± 2%

No-carrier-added (NCA) [18F]fluoride ion (10–12 mCi) was trapped onto a QMA cartridge, and further eluted with aqueous pyridazine-HCl buffer (1 mL, pH = 2.0) into an EP tube to obtain a [18F]fluoride solution (10–12 μCi/μL). For labeling, precursor (**12a**, 300 nmol) in DMF (20 μL) was added in an EP tube, followed by the addition of the above [18F]fluoride solution (40 μL). The mixture was placed in a heating block at 90 °C for 10 min. Then the reaction was quenched by addition of MeCN/H_2O (3:2, 500 μL). An aliquot was removed for analysis by radio-TLC and raido-HPLC for product identity and calculation of radiochemical conversion (RCC). The product identity was determined by comparison of the HPLC radio-trace with the HPLC UV-trace of the authentic reference sample Bn-PyBF_3 conjugate (**12a**).

Radio-TLC

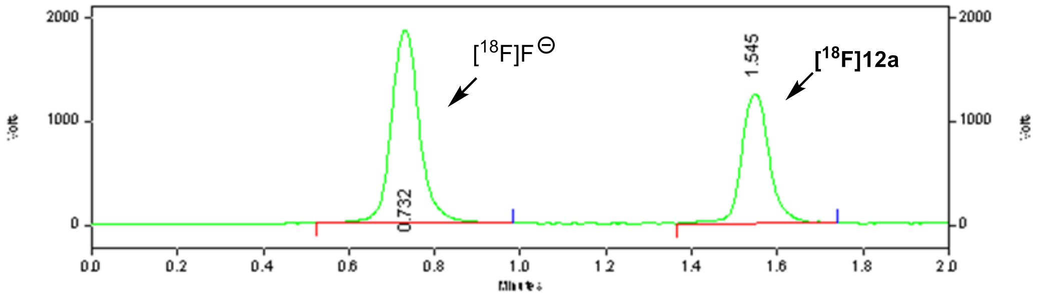

检测器 1
Results

保留时间	面积	面积百分比	峰高	高度百分比
0.732	8434921	59.956	1856139	59.838
1.545	5633604	40.044	1245807	40.162
Totals	14068525	100.000	3101946	100.000

UV-254 nm

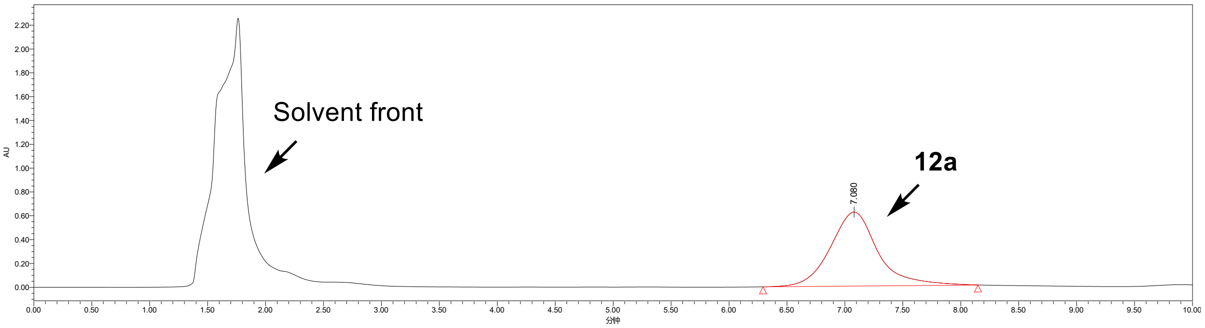

Radio-HPLC-test

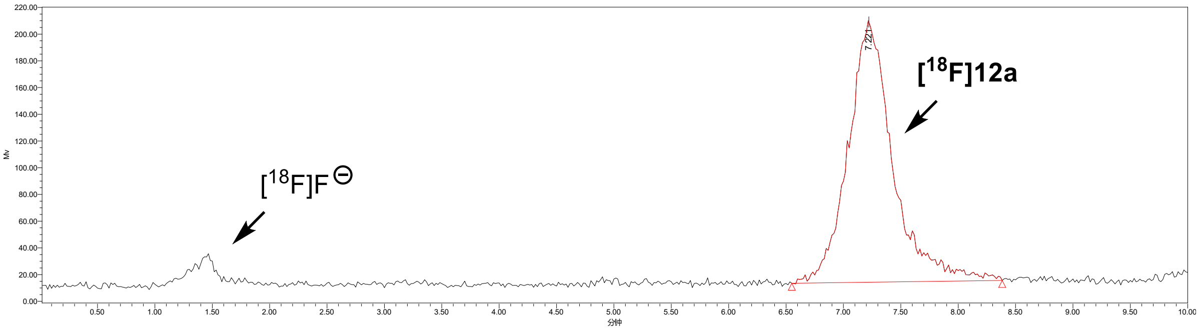

Figure S14. Radio-TLC and radio-HPLC trace of the ^{18}F-^{19}F isotope exchange reaction yielding **[^{18}F]12a**.

Entry	rTLC RCC (%)	rHPLC RCP (%)	RCC × RCP (%)
1	40	> 99	40
2	36	> 99	36
3	42	> 99	42
	Mean ± SD		39 ± 2

rTLC run in MeOH:DCM (1:10, v/v)

[18F](1-(2-(1-Benzyl-1*H*-1,2,3-triazol-4-yl)ethyl)-2-methylpyridin-1-ium-4-yl)trifluoroborate ([18F]15a)

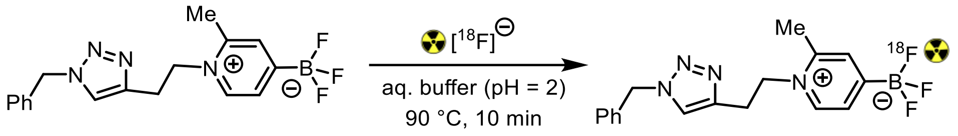

15a **[18F]15a**, RCC = 40 ± 1%

No-carrier-added (NCA) [18F]fluoride ion (10–12 mCi) was trapped onto a QMA cartridge, and further eluted with aqueous pyridazine-HCl buffer (1 mL, pH = 2.0) into an EP tube to obtain a [18F]fluoride solution (10–12 μCi/μL). For labeling, precursor (**15a**, 300 nmol) in DMF (20 μL) was added in an EP tube, followed by the addition of the above [18F]fluoride solution (40 μL). The mixture was placed in a heating block at 90 °C for 10 min. Then the reaction was quenched by addition of MeCN/H_2O (3:2, 500 μL). An aliquot was removed for analysis by radio-TLC and raido-HPLC for product identity and calculation of radiochemical conversion (RCC). The product identity was determined by comparison of the HPLC radio-trace with the HPLC UV-trace of the authentic reference sample Bn-PyBF_3 conjugate (**15a**).

Radio-TLC

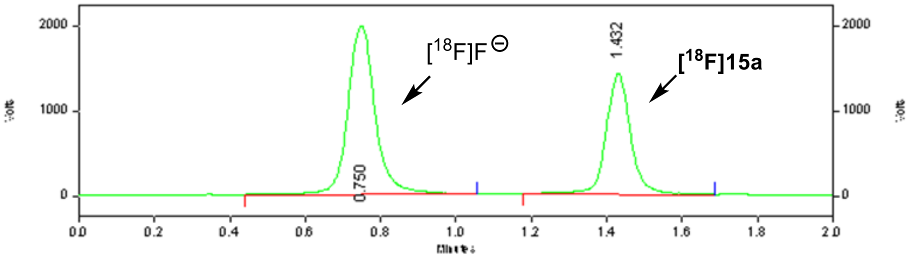

检测器 1
Results

保留时间	面积	面积百分比	峰高	高度百分比
0.750	9970329	60.870	1976057	58.276
1.432	6409339	39.130	1414824	41.724
Totals	16379668	100.000	3390881	100.000

UV-254 nm

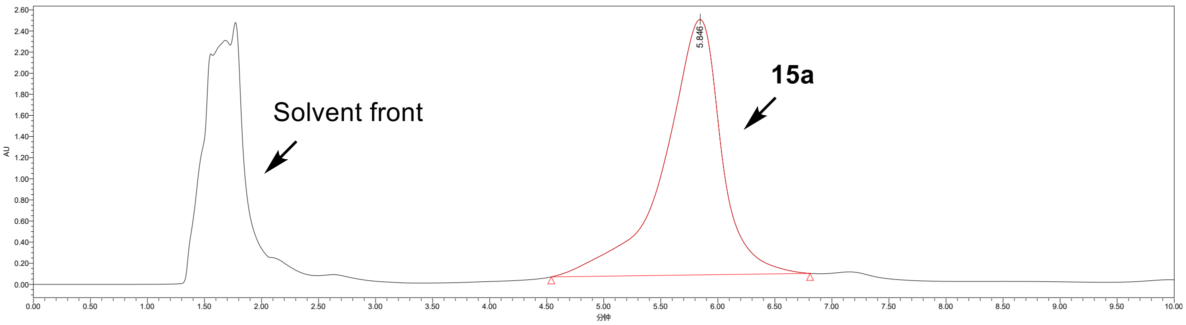

Radio-HPLC-test

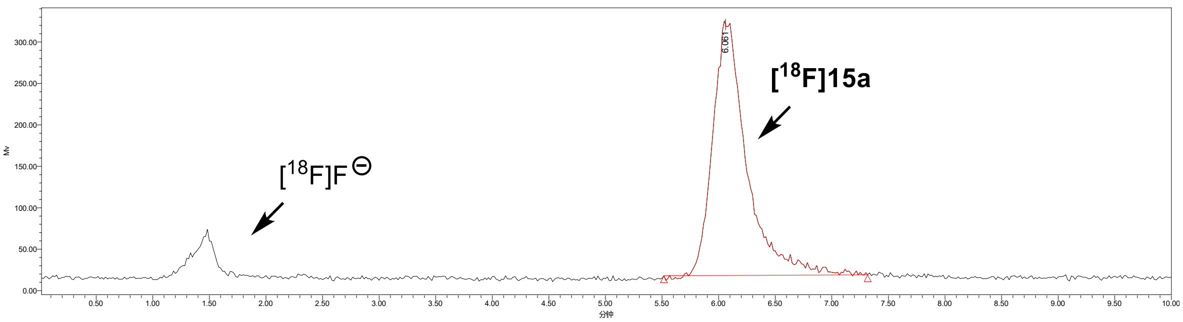

Figure S15. Radio-TLC and radio-HPLC trace of the ^{18}F-^{19}F isotope exchange reaction yielding **[^{18}F]15a**.

Entry	rTLC RCC (%)	rHPLC RCP (%)	RCC × RCP (%)
1	39	> 99	39
2	41	> 99	41
3	40	> 99	40
	Mean ± SD		40 ± 1

rTLC run in MeOH:DCM (1:10, v/v)

Failed substrate

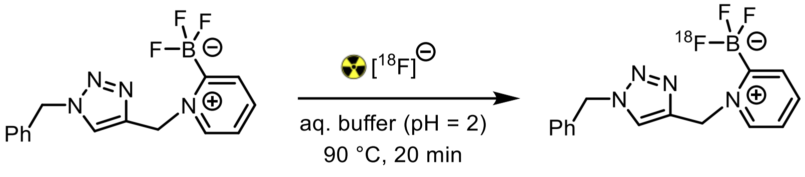

3a **[18F]3a**, RCC <1%

No-carrier-added (NCA) [18F]fluoride ion (10–12 mCi) was trapped onto a QMA cartridge, and further eluted with aqueous pyridazine-HCl buffer (1 mL, pH = 2.0) into an EP tube to obtain a [18F]fluoride solution (10–12 μCi/μL). For labeling, precursor (**3a**, 300 nmol) in DMF (20 μL) was added in an EP tube, followed by the addition of the above [18F]fluoride solution (40 μL). The mixture was placed in a heating block at 90 °C for 10 min. Then the reaction was quenched by addition of MeCN/H_2O (3:2, 500 μL). An aliquot was removed for analysis by raido-HPLC for product identity and calculation of radiochemical conversion (RCC). The product identity was determined by comparison of the HPLC radio-trace with the HPLC UV-trace of the authentic reference sample Bn-PyBF_3 conjugate (**3a**).

UV-220 nm

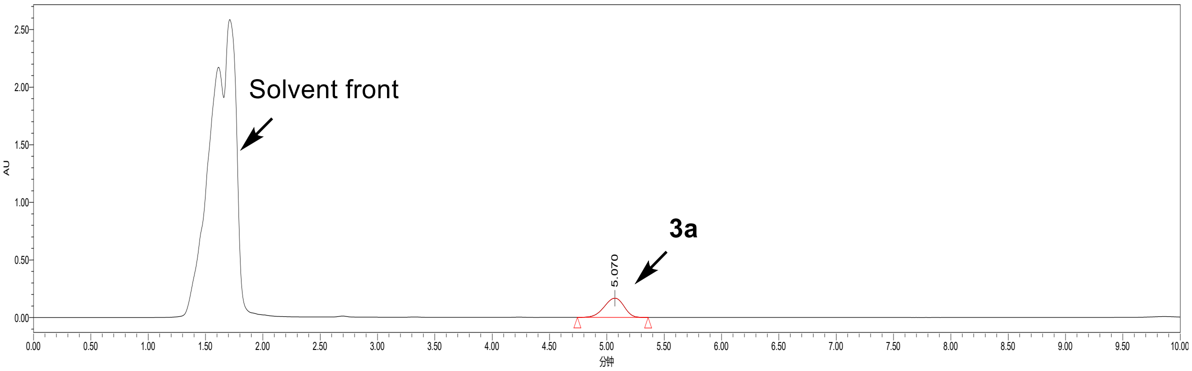

Radio-HPLC-test

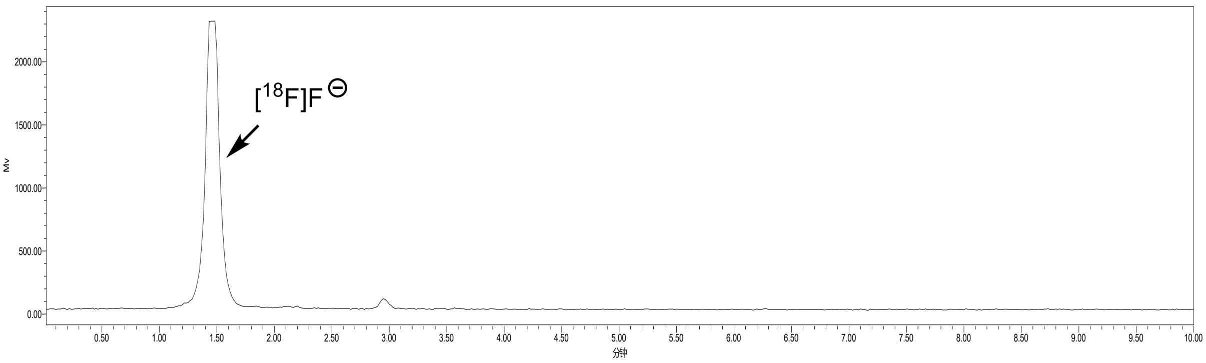

Figure S16. Radio-HPLC trace of the ^{18}F-^{19}F isotope exchange reaction of **3a**.

Discussion: The click product **3a** exhibited negligible RCC under mild exchange conditions, despite pyridinium-2-yl trifluoroborate displaying the highest hydrolytic stability among the three pyridinium orientations (C2, C3, C4). We attribute this behavior to the stronger electronic stabilization of the N^+–BF_3^- ion

pair in **3a**, which likely results in stronger B–F bond character and a higher activation barrier for fluoride exchange. The enhanced Coulombic interaction and more rigid ion pairing in **3a** may suppress formation of the transient intermediate required for ^{18}F–^{19}F exchange. This observation highlights a trade-off between maximal aqueous stability and exchange reactivity within this structural series.

[^{18}F]Indomethacin derivative ([^{18}F]4u)

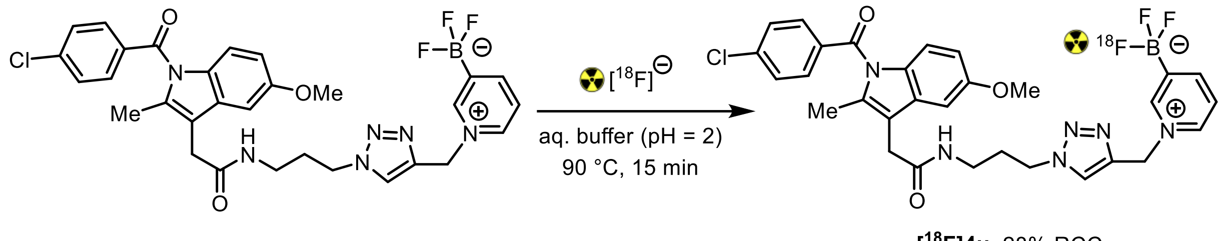

No-carrier-added (NCA) [^{18}F]fluoride ion (80 mCi) was trapped onto a QMA cartridge, and further eluted with saline (1 mL) into an EP tube. Anhydrous MeCN (1 mL) was added to the tube and dried with N_2 flow at 105 °C, and this process was repeated twice. After complete dryness, saline (60 μL), aqueous pyridazine-HCl buffer (40 μL, pH = 2.0), and precursor (**4u**, 300 nmol) in DMF (20 μL) was added to the EP tube. The mixture was placed in a heating block at 90 °C for 15 min. Then the reaction was quenched by addition of PBS (500 μL, pH = 8.0) and further diluted with H_2O (3 mL). An aliquot was removed for analysis by radio-TLC and radio-HPLC for product identity and calculation of radiochemical conversion (RCC). Furthermore, the reaction mixture was loaded onto a C18 light cartridge which was washed with water (3 mL). Radiochemically pure product was eluted with ethanol (1.0 mL) into a glass vial to provide 10~12 mCi tracer, total time: 30 min. The tracer was formulated in isotonic saline for PET/MR imaging studies.

Radio-TLC

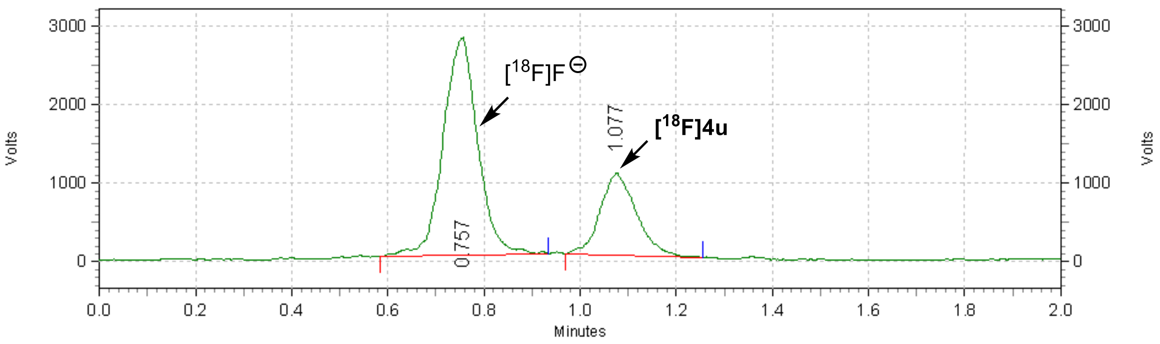

检 测 器 1
Results

保 留 时 间	面 积	面 积 百 分 比	峰 高	高 度 百 分 比
0.757	13900630	71.584	2771143	72.561
1.077	5518053	28.416	1047893	27.439
Totals	19418683	100.000	3819036	100.000

UV-254 nm

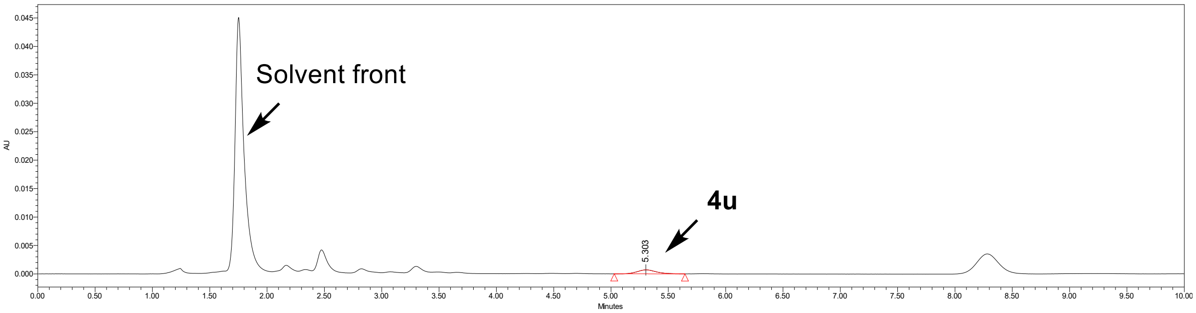

Radio-HPLC-test

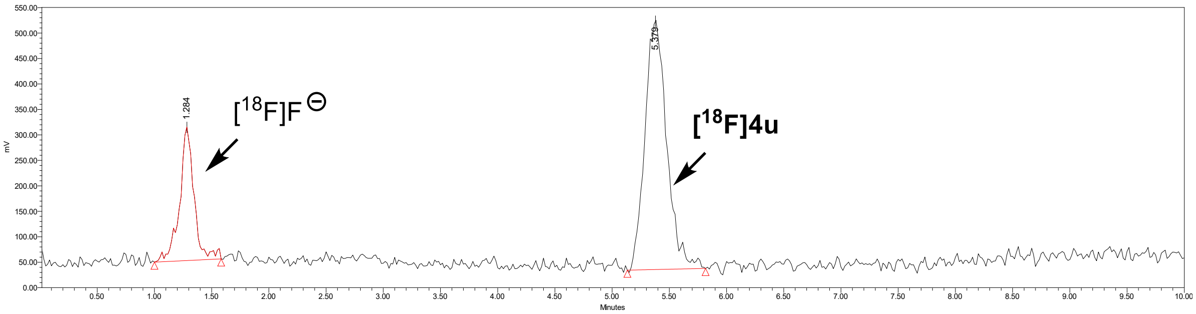

Figure S17. Radio-TLC and radio-HPLC trace of the ^{18}F-^{19}F isotope exchange reaction yielding **[^{18}F]4u**.

Entry	rTLC RCC (%)	rHPLC RCP (%)	RCC × RCP (%)
1	28	> 99	28

rTLC run in MeOH:DCM (1:10, v/v)

After purification

Co-injection with standard compound 4u

UV-254 nm

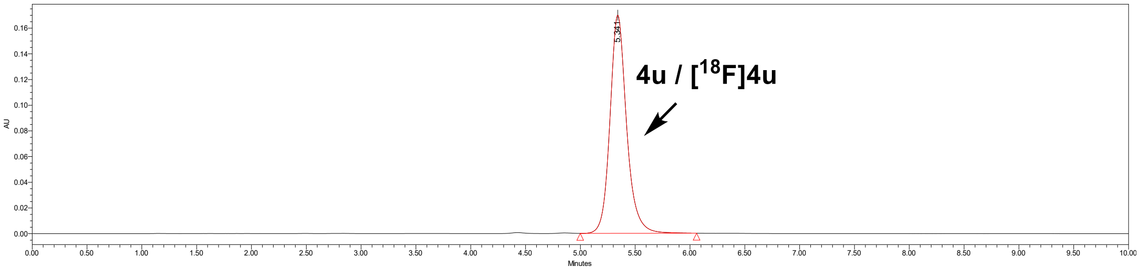

Radio-HPLC

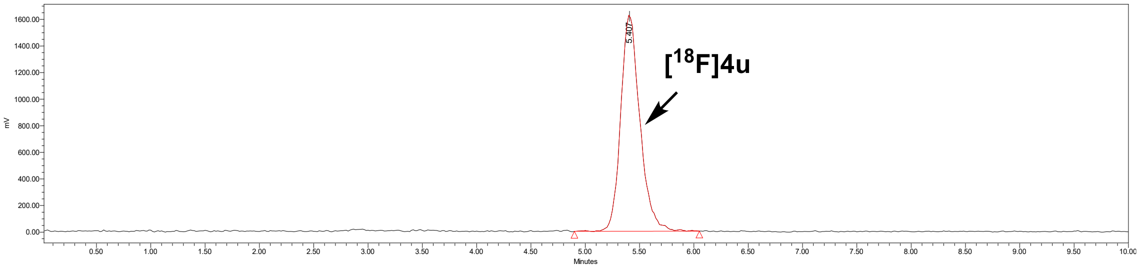

Figure S18. Radio-HPLC trace of isolated **[^{18}F]4u** after purification.

[18F]Indomethacin derivative ([18F]5u)

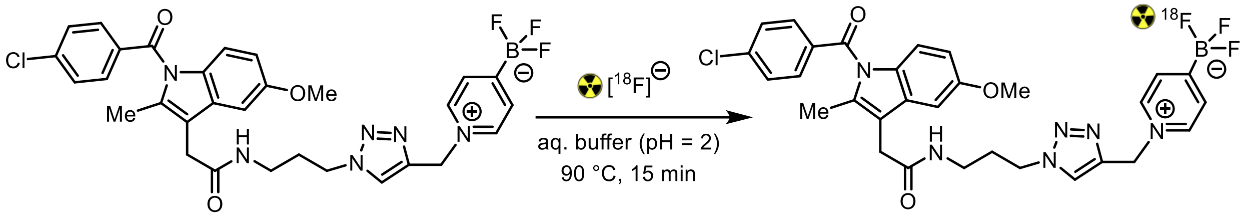

5u

[18F]5u, 24% RCC

No-carrier-added (NCA) [18F]fluoride ion (80 mCi) was trapped onto a QMA cartridge, and further eluted with saline (1 mL) into an EP tube. Anhydrous MeCN (1 mL) was added to the tube and dried with N_2 flow at 105 °C, and this process was repeated twice. After complete dryness, saline (60 μL), aqueous pyridazine-HCl buffer (40 μL, pH = 2.0), and precursor (**5u**, 300 nmol) in DMF (20 μL) was added to the EP tube. The mixture was placed in a heating block at 90 °C for 15 min. Then the reaction was quenched by addition of PBS (500 μL, pH = 8.0) and further diluted with H_2O (3 mL). An aliquot was removed for analysis by radio-TLC and radio-HPLC for product identity and calculation of radiochemical conversion (RCC). Furthermore, the reaction mixture was loaded onto a C18 light cartridge which was washed with water (3 mL). Radiochemically pure product was eluted with ethanol (1.0 mL) into a glass vial to provide 10~12 mCi tracer, total time: 30 min. The tracer was formulated in isotonic saline for PET/MR imaging studies.

Radio-TLC

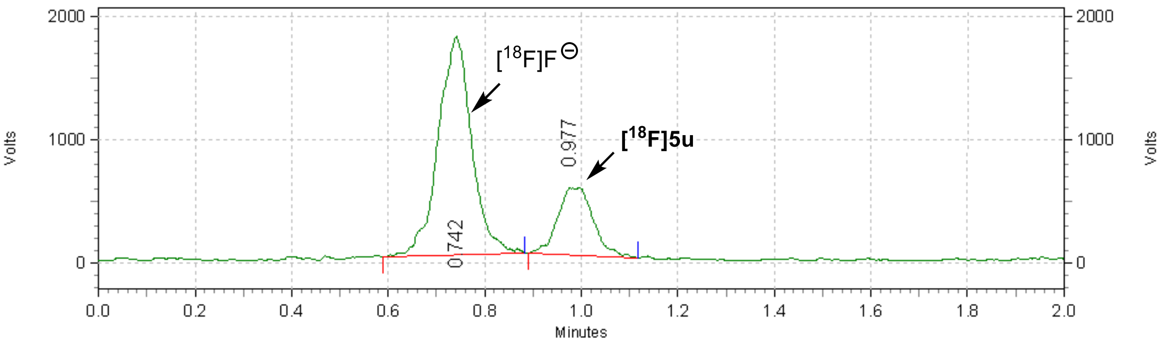

检 测 器 1

Results

保留时间	面积	面积百分比	峰高	高度百分比
0.742	8524954	75.426	1772399	76.475
0.977	2777443	24.574	545235	23.526
Totals	11302397	100.000	2317634	100.000

UV-254 nm

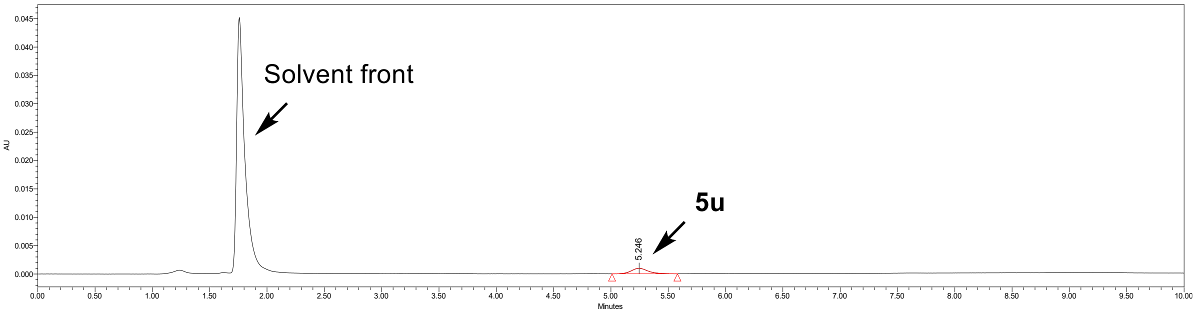

Radio-HPLC-test

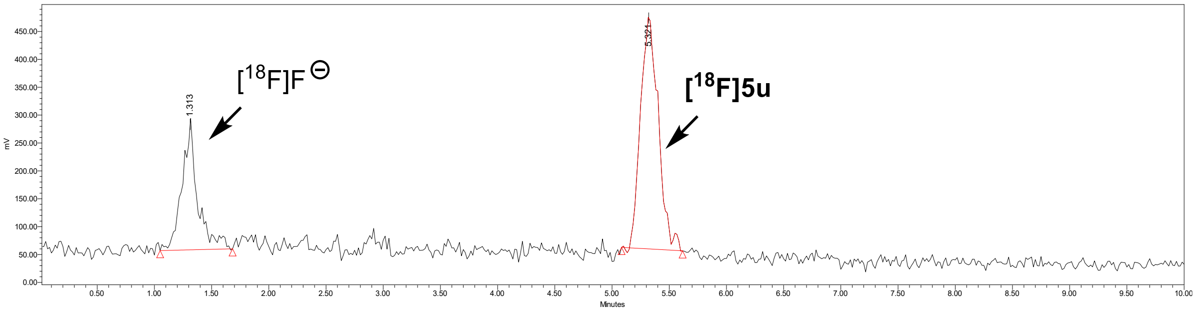

Figure S19. Radio-TLC and radio-HPLC trace of the ^{18}F-^{19}F isotope exchange reaction yielding **[^{18}F]5u**.

Entry	rTLC RCC (%)	rHPLC RCP (%)	RCC × RCP (%)
1	24	> 99	24

rTLC run in MeOH:DCM (1:10, v/v)

After purification

Co-injection with standard compound 5u

UV-254 nm

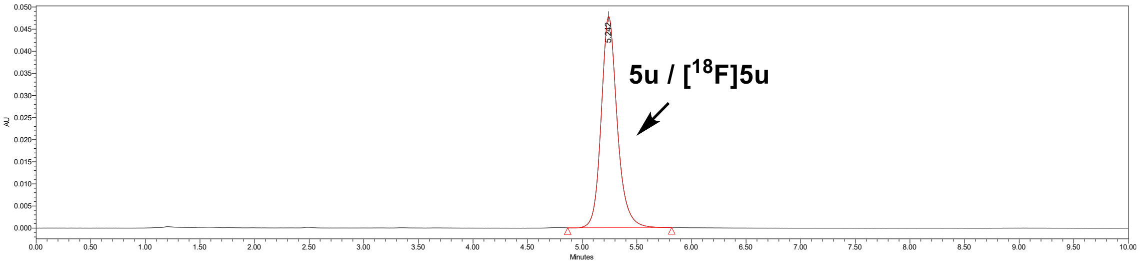

Radio-HPLC

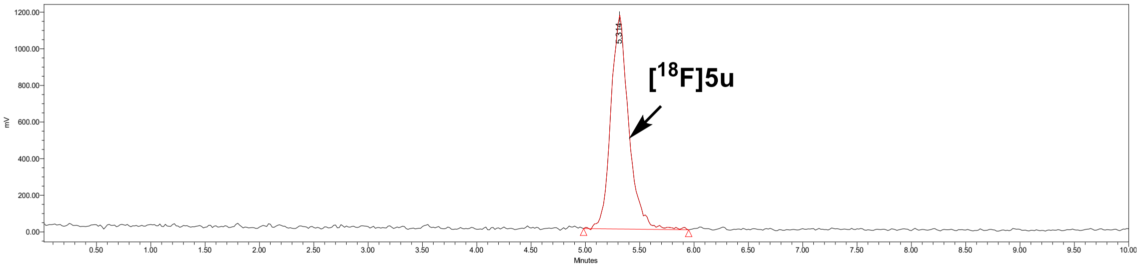

Figure S20. Radio-HPLC trace of isolated **[^{18}F]5u** after purification.

In vivo microPET imaging studies

Animal research was conducted in accordance with the guidelines and approved by the Animal Care and Ethics Committee of Jiangsu Institute of Nuclear Medicine.

In vivo distribution of radioactive probe **[^{18}F]4u** or **[^{18}F]5u** in ICR mice was assessed by a microPET/MR system (Bruker BioSpec 94/30 USR + PET insert) under 2.0% isoflurane anesthesia. The probe **[^{18}F]4u** or **[^{18}F]5u** (~7.4 MBq, 0.20 mL in 10 % ethanol/saline) was injected via the tail vein. The static microPET/MR studies were conducted at 1 h and 2 h post injection, respectively. The images were reconstructed and analyzed by PMOD 4.3 software.

MECHANISTIC STUDIES

DFT calculation

Methods

Density Functional Theory (DFT) calculations were performed with the Gaussian 16 program[18]. Structural optimizations and frequency calculations were performed with the PBE0 functional[19] with D3 dispersion correction[20] and Becke-Johnson damping (BJ)[21], utilizing the def2-SVP basis set[22] on all atoms. All the reported minima are from an unrestricted optimization. Tight SCF convergence and geometry optimization criterions were chosen. Frequency calculations at the same level had been performed to confirm each stationary point to be either a minimum or a transition structure. To obtain more accurate energies, single-point energy calculations were performed on all optimized structures applying the def2-TZVPP basis set[22] for all atoms. Solvent effects of acetonitrile were taken into account using the conductor-like polarized continuum model (CPCM).[23] The reported energies are zero-point energy corrected Gibbs free energies in methanol. All the transition states are subjected to IRC calculation to confirm the accuracy of the bond formation and cleavage processes.

Results and discussion

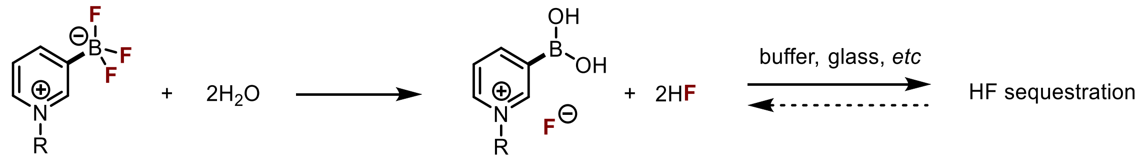

The overall reaction of the hydrolysis is shown above, and three defluorination steps were shown in potential surface. The subsequent fluoride and HF sequestration processes, experimentally confirmed in previous work[24], are expected to be highly exergonic. In previous ^{18}F-^{19}F isotope exchange reaction[25], $SnCl_4$ was usually used to promote B–F bond cleavage. Here, we also use $SnCl_4$ as a mediator for fluoride transfer.

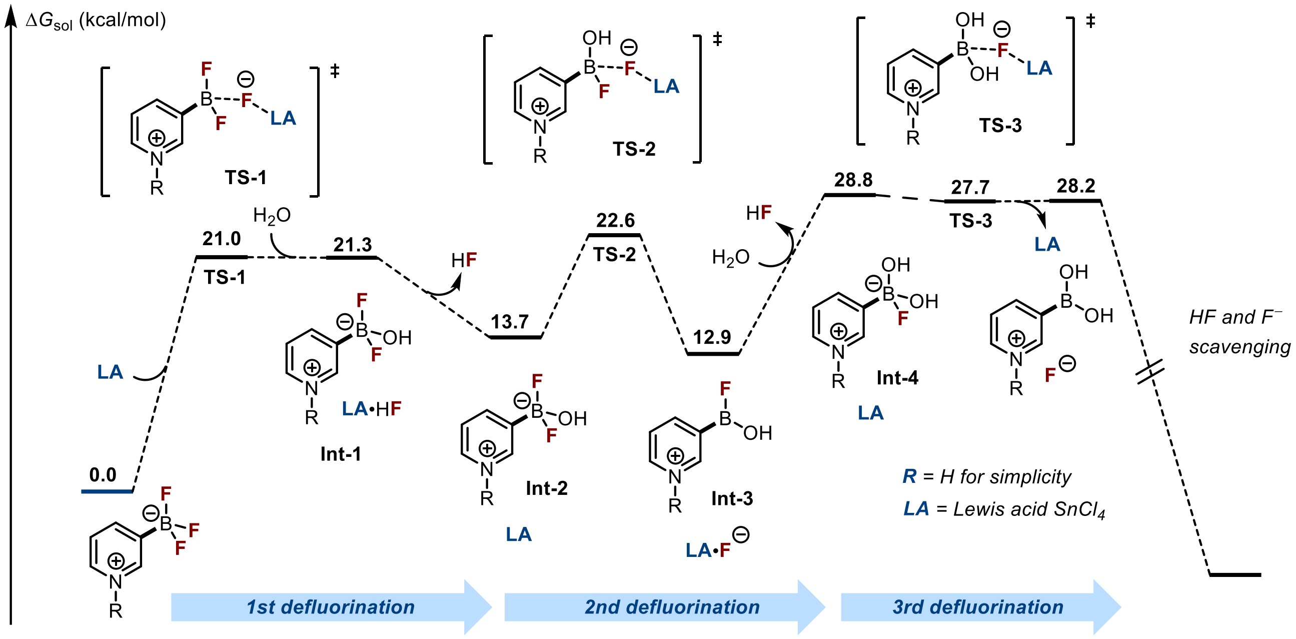

Experimentally, only the zwitterionic trifluoroborate substrate and the boronic acid decomposition product were observed by NMR, indicating the short-lived nature of the mono- and difluorinated species[26]. Our calculation results show that, partially fluorinated intermediates **Int-1** to **Int-4** are all transient intermediates, which is consistent with the experimental observations.

Calculated coordinates

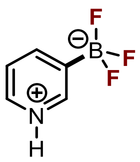

PyH-BF$_3$

G = -572.4313315 Hartree

C	2.82289700	-0.00442800	0.00887900
C	2.17184400	1.21330700	0.00297500
C	0.77332200	1.23488300	-0.00883000
C	0.02019500	0.05398700	-0.00736800
C	0.74029000	-1.13064500	-0.00989700
N	2.08799600	-1.13217200	-0.00016600
H	0.24497900	2.19262000	-0.02453400
H	3.90724700	-0.11807800	0.01719500
H	2.76026700	2.13240800	0.00475200
H	0.23574200	-2.09959200	-0.02667900
H	2.57289700	-2.02607900	-0.00260500
B	-1.63586500	0.00476200	0.00070000
F	-2.08500100	0.85021200	-1.00592400
F	-2.06190400	0.43244400	1.25227400
F	-2.00076900	-1.32515600	-0.23357400

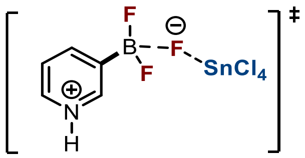

TS-1

G = -2627.18201 Hartree

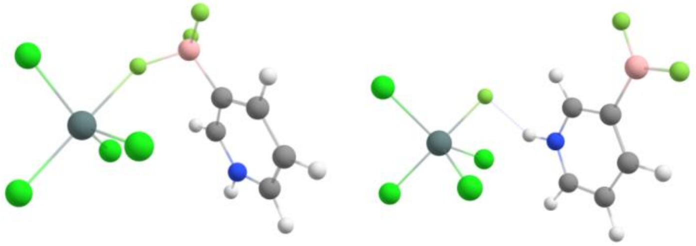

IRC backward result ***IRC forward result***

C	2.07510700	-2.60604200	-0.12393200
C	3.02281500	-2.23026000	0.80403500
C	3.45761200	-0.90141300	0.81608900
C	2.99118000	0.01043300	-0.13165600
C	2.07223100	-0.44622700	-1.07068000
N	1.64775300	-1.71140700	-1.03147400
H	4.15201000	-0.56566100	1.59052700
H	1.61972000	-3.59624300	-0.15879700
H	3.37007500	-2.95146500	1.54403500
H	1.60718600	0.19396300	-1.81879000
H	0.82938600	-1.91524000	-1.62959400
B	3.31159500	1.54130100	-0.09147800
F	0.32100000	1.19419400	-0.31772600
F	3.87749800	2.06254100	0.97432200
F	3.07390900	2.30351100	-1.12996100
Sn	-1.37698900	0.20352700	0.07021200
Cl	-2.43963500	2.26539300	0.40388500
Cl	-1.05101400	-0.79862500	-2.13783000
Cl	-0.12835700	-0.94223700	1.75041600
Cl	-3.32137500	-1.11685500	0.40334600

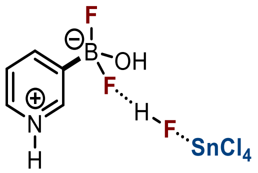

Int-1

G = -2703.565318 Hartree

C	-2.12361100	0.79086800	0.76326300
C	-3.12219300	0.04928400	0.14728900
C	-4.09133800	0.76594300	-0.55958400
C	-4.03966900	2.16111100	-0.65400200

C	-2.98940300	2.82975000	-0.05889200
N	-2.07710100	2.12435600	0.63151600
H	-4.88888100	0.20197100	-1.05115800
H	-1.32464700	0.33167300	1.35020900
H	-4.79073600	2.72591100	-1.20810700
H	-2.83763900	3.90781000	-0.11927200
H	-1.24422500	2.59794100	0.98551200
B	-3.08303200	-1.57229900	0.17589400
F	-2.70584300	-2.07549200	1.39742100
F	-1.83323100	-1.91098200	-0.74831000
O	-4.21882500	-2.10574600	-0.42212300
H	-4.27784900	-3.05409900	-0.27570800
Sn	1.74502300	-0.03370000	-0.06950300
Cl	1.04796700	2.23878500	-0.05267600
Cl	0.77120500	-1.27220000	1.66690100
Cl	3.85088900	0.34296700	0.91060700
Cl	2.44876200	-1.01609800	-2.05888300
F	-0.24097000	-0.30647800	-0.98391900
H	-0.98404600	-1.12312600	-0.86571300

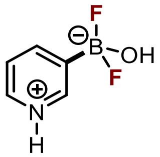

Int-2

G = -548.3868978 Hartree

C	-0.74300500	-1.13413800	-0.00089700
C	-0.02981000	0.05575000	-0.00922500
C	-0.78770600	1.23212400	-0.02534600
C	-2.18684400	1.20272600	-0.00820900
C	-2.83155700	-0.01754600	0.01121400
N	-2.08980100	-1.14226500	0.00987400
H	-0.26185300	2.19071100	-0.05947800
H	-0.22186100	-2.09482200	-0.01583200
H	-2.78032900	2.11860100	-0.01519900
H	-3.91516600	-0.13720500	0.02309400
H	-2.56978200	-2.03856800	0.01346000
B	1.61999800	0.00625700	-0.00088200
F	2.05326400	0.38918600	1.28255700
F	2.07501000	0.95630800	-0.93288900
O	1.96011900	-1.34650800	-0.33130300
H	2.91571500	-1.44503400	-0.31255800

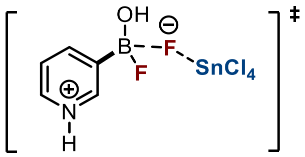

TS-2

G = -2603.156855 Hartree

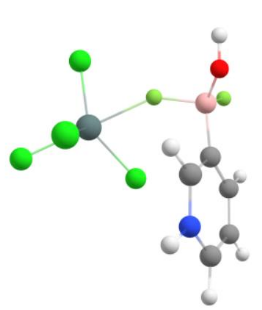

IRC backward result

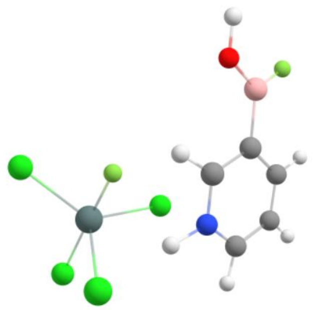

IRC forward result

C	-2.23153900	-2.61309100	-0.10835000
C	-3.12913100	-2.15587800	0.83234400
C	-3.45585500	-0.79533800	0.84536300
C	-2.94202100	0.07134700	-0.11834500
C	-2.08595200	-0.46570600	-1.07265100
N	-1.75184000	-1.75807900	-1.02846300
H	-4.09717200	-0.40208900	1.63827600
H	-1.85471900	-3.63561700	-0.14216300
H	-3.51712200	-2.84258800	1.58498600
H	-1.58498100	0.13874300	-1.82756800
H	-0.96818000	-2.02881100	-1.63901700
B	-3.08427800	1.63702500	-0.08844000
F	-0.30506900	1.24043700	-0.32934900
F	-3.36522900	2.22699400	1.07011600
O	-2.97532700	2.32576400	-1.23020300
H	-2.96137500	3.28249200	-1.10994800
Sn	1.34457400	0.17186400	0.06181000
Cl	0.05368600	-0.91049900	1.75005100
Cl	2.49619200	2.19556000	0.36019100
Cl	3.24248200	-1.21046400	0.42767800
Cl	0.99242900	-0.84194400	-2.12904200

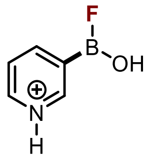

Int-3

G = -448.393061 Hartree

C	0.49934300	-1.15480000	-0.00015500
C	-0.20798500	0.04009600	-0.00007800
C	0.53190300	1.23127300	-0.00004800
C	1.92877400	1.20720900	0.00012400
C	2.57374800	-0.01438200	0.00009000
H	2.33416100	-2.03937800	-0.00013900
H	0.00756500	-2.13041100	-0.00015300
H	0.00466400	2.18951100	-0.00012000
H	2.51684600	2.12634100	0.00032400
H	3.65950200	-0.12677100	0.00013200
N	1.84250800	-1.14549600	-0.00007400
B	-1.79018400	0.01747700	0.00000200

F	-2.43023500	1.17331000	-0.00010500
O	-2.39842000	-1.16947900	0.00018900
H	-3.36460600	-1.14853200	0.00029000

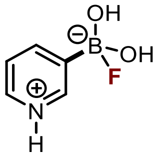

Int-4

G = -524.3401083 Hartree

C	0.77522000	-1.13174200	-0.01824100
C	0.03836800	0.04451000	0.00355500
C	0.77352200	1.23270500	0.05144500
C	2.17327900	1.23327400	0.03857800
C	2.84203600	0.02694400	-0.00620000
N	2.12188000	-1.11259600	-0.02724500
H	0.21513600	2.17129900	0.11982200
H	0.28099600	-2.10628800	-0.00308800
H	2.74881000	2.16020400	0.07049300
H	3.92767600	-0.07210700	-0.01722600
B	-1.61675500	-0.02273000	0.01207400
H	2.62014200	-1.99866600	-0.04210100
O	-1.90868700	-1.29873600	0.61301700
H	-2.81250800	-1.27426800	0.93793400
O	-2.14616400	1.11383300	0.72863200
H	-2.68430000	1.61526000	0.11052000
F	-2.02678700	0.05016300	-1.35489100

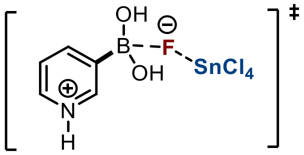

TS-3

G = -2579.126014 Hartree

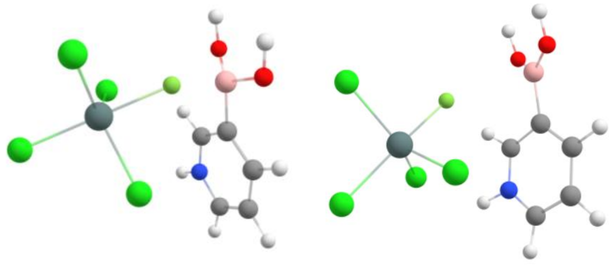

IRC backward result ***IRC forward result***

C	-3.06558800	-2.34167100	-0.03887700
C	-3.55206800	-1.54871900	-1.05583300

C	-3.33196900	-0.16795900	-1.00258300
C	-2.67620900	0.41191600	0.08265000
C	-2.26089000	-0.43458000	1.10011600
N	-2.44554400	-1.75791200	1.00401400
H	-3.63919900	0.46959500	-1.83529300
H	-3.13548600	-3.42947600	-0.02713500
H	-4.05084000	-2.01506600	-1.90616800
H	-1.70020700	-0.07808700	1.96467900
B	-2.17776800	1.92419800	0.13971600
O	-2.23292200	2.50774600	1.37747700
H	-1.82264300	3.37547700	1.42703300
O	-2.17942500	2.56544500	-1.06726500
H	-1.81076400	3.45255700	-1.05334000
F	-0.24478400	1.32388500	0.25517900
Sn	1.29050100	0.00279200	-0.04256500
Cl	-0.08782000	-1.04367600	-1.66815300
Cl	2.58803900	1.91626300	-0.46398400
Cl	0.79902300	-0.80735200	2.15909900
Cl	3.08215600	-1.52998600	-0.33163400
H	-2.01986100	-2.34386400	1.72006900

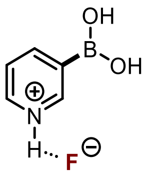

$^{\oplus}$PyHB(OH)$_2$ $^{\ominus}$F

G = -524.3411518 Hartree

C	1.80956500	1.48092500	-0.00010200
C	0.65961100	2.26641100	0.00010300
C	-0.57689300	1.62727400	0.00008300
C	-0.63980600	0.22818700	-0.00011500
C	0.58048700	-0.45689400	-0.00031100
H	2.85140300	-1.09610100	-0.00009200
H	2.80466000	1.93764200	-0.00005300
H	0.73843800	3.35538100	0.00027800
H	-1.50359000	2.20738800	0.00024800
H	0.61325900	-1.55089400	-0.00046800
N	1.76655200	0.14976800	-0.00032500
F	3.19964900	-1.98524700	0.00039500
B	-2.00524400	-0.54184200	-0.00002800
O	-1.96285100	-1.90130200	-0.00017400
H	-2.81013100	-2.35470000	-0.00007200
O	-3.14443600	0.20627900	0.00025500
H	-3.97000100	-0.28590300	0.00042200

H_2O

G = -76.3838251 Hartree

O	0.00000000	0.00000000	0.11964500
H	0.00000000	0.75408900	-0.47858200

H	0.00000000	-0.75408900	-0.47858200

$SnCl_4$

G = -2054.784105 Hartree

Sn	0.00000000	0.00000000	0.00000000
Cl	-1.33667000	1.33667000	1.33666900
Cl	1.33667000	-1.33667000	1.33666900
Cl	1.33667000	1.33667000	-1.33666900
Cl	-1.33667000	-1.33667000	-1.33666900

HF

G = -100.4064694 Hartree

F	0.00000000	0.00000000	0.09202300
H	0.00000000	0.00000000	-0.82820600

$^{\ominus}F{-}SnCl_4$

G = -2154.77922 Hartree

Sn	0.00000000	0.00000000	0.17039100
F	0.00000000	0.00000000	2.13146000
Cl	0.00000000	2.38618600	0.20727200
Cl	-2.06649700	-1.19309300	0.20727200
Cl	2.06649700	-1.19309300	0.20727200
Cl	0.00000000	0.00000000	-2.25138800

SPECTROSCOPIC DATA

^{1}H NMR of 3a

CD3CN, 400 MHz, 25 °C

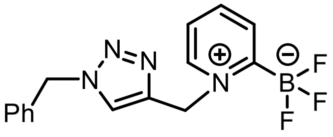

3a

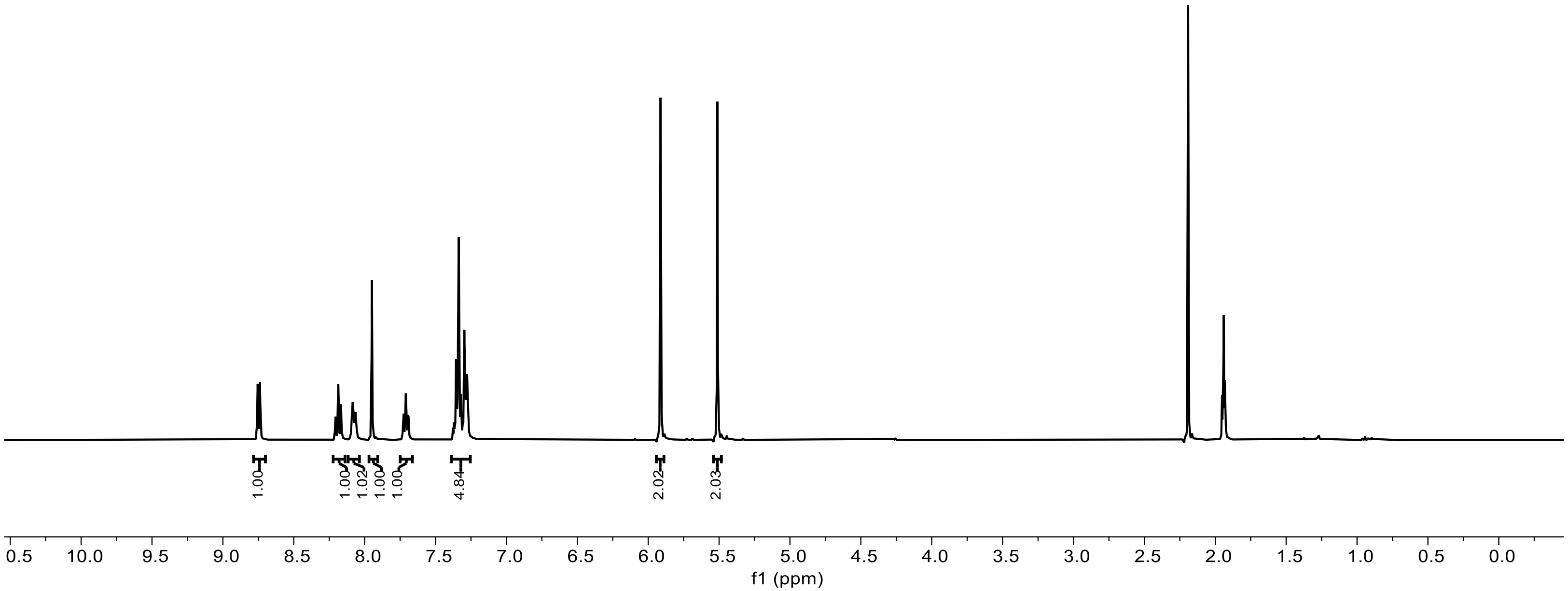

^{13}C NMR of 3a

$CDCl_3$, 101 MHz, 25 °C

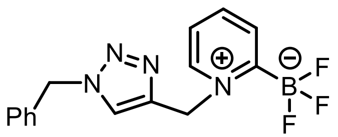

3a

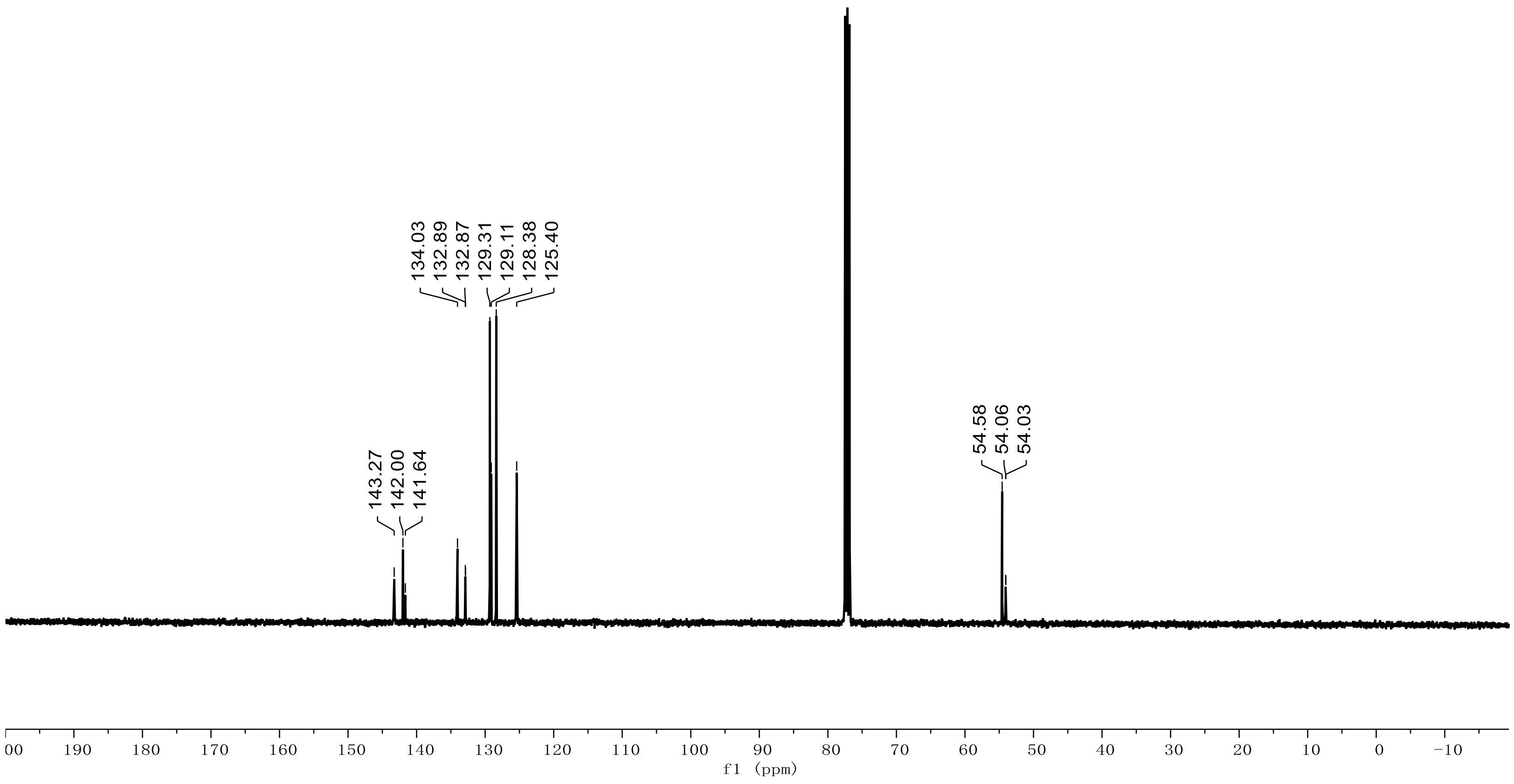

^{19}F NMR of 3a

CDCl$_3$, 376 MHz, 25 °C

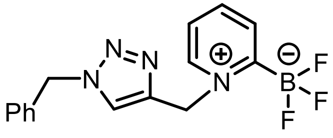

3a

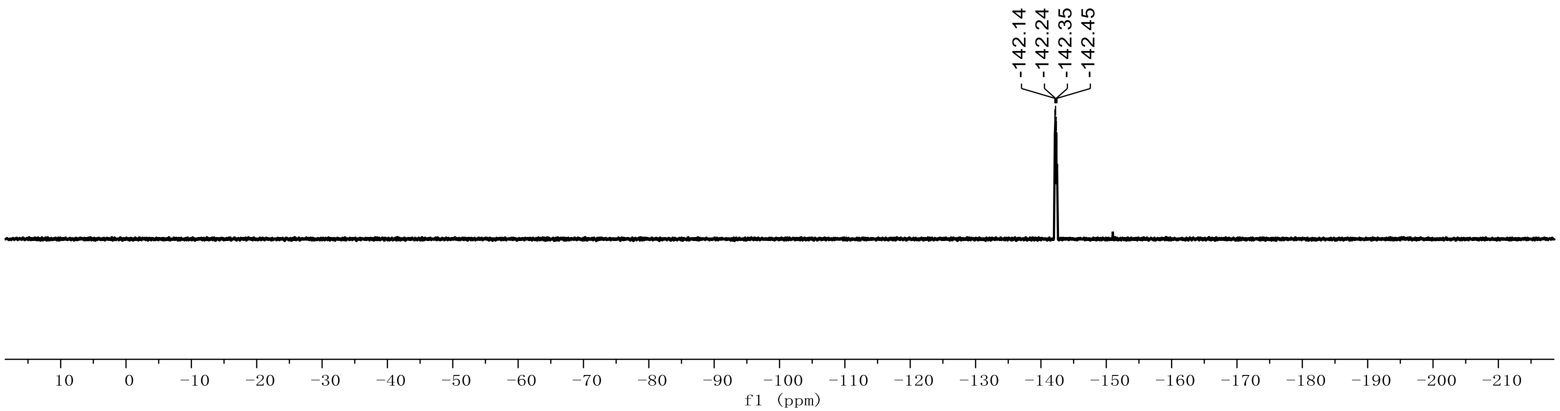

^{11}B NMR of 3a

$CDCl_3$, 128 MHz, 25 °C

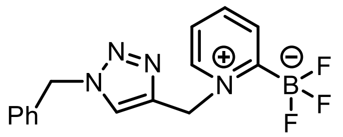

3a

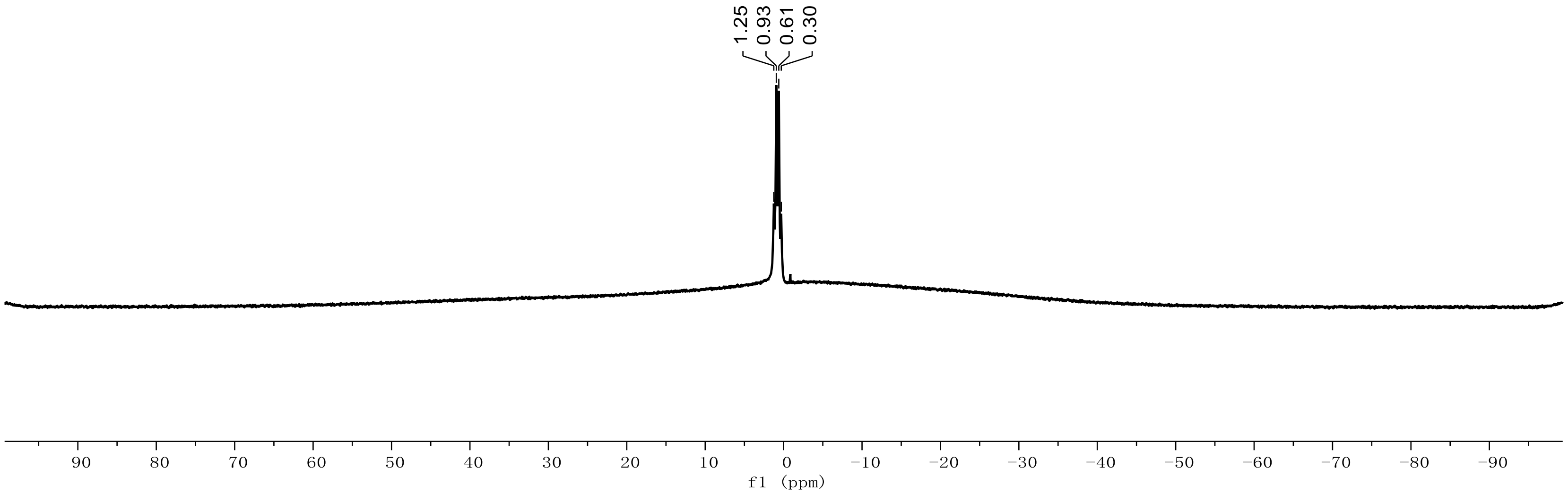

^{1}H NMR of 4a

CD3CN, 400 MHz, 25 °C

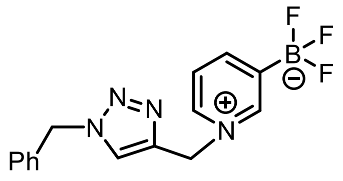

4a

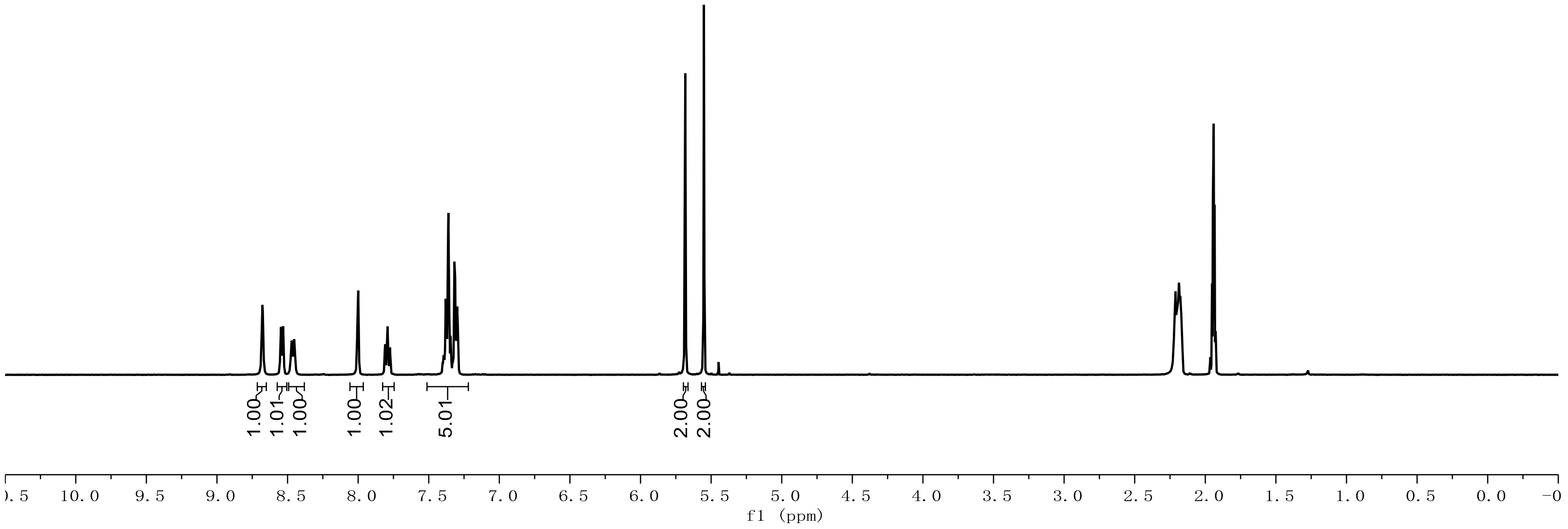

^{13}C NMR of 4a

CD_3CN, 101 MHz, 25 °C

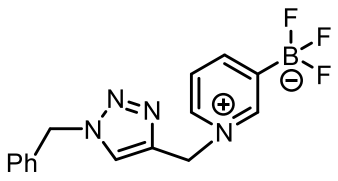

4a

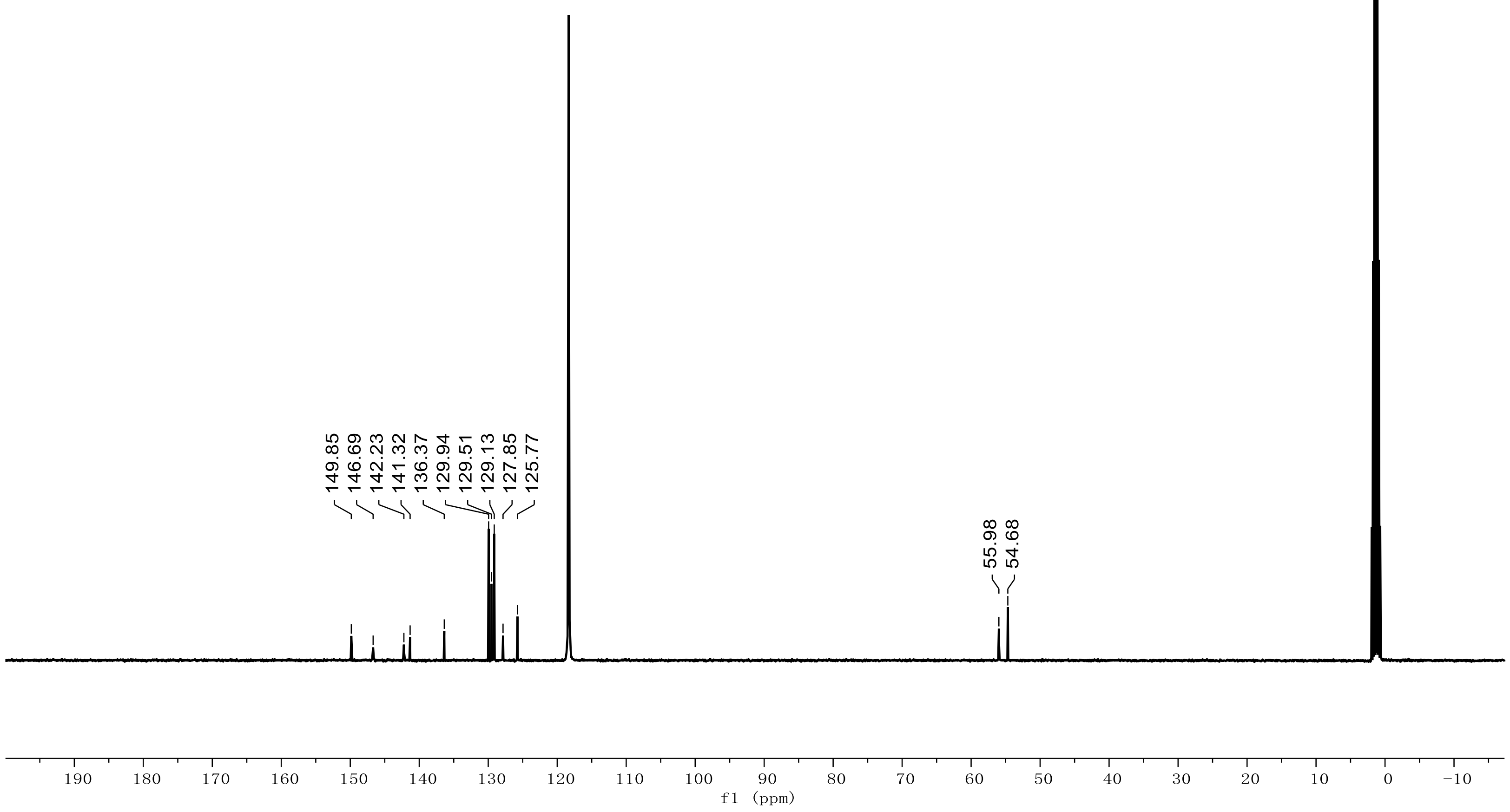

^{19}F NMR of 4a

CD_3CN, 376 MHz, 25 °C

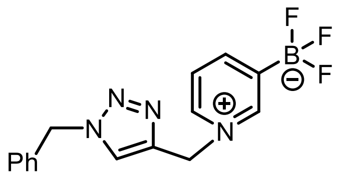

4a

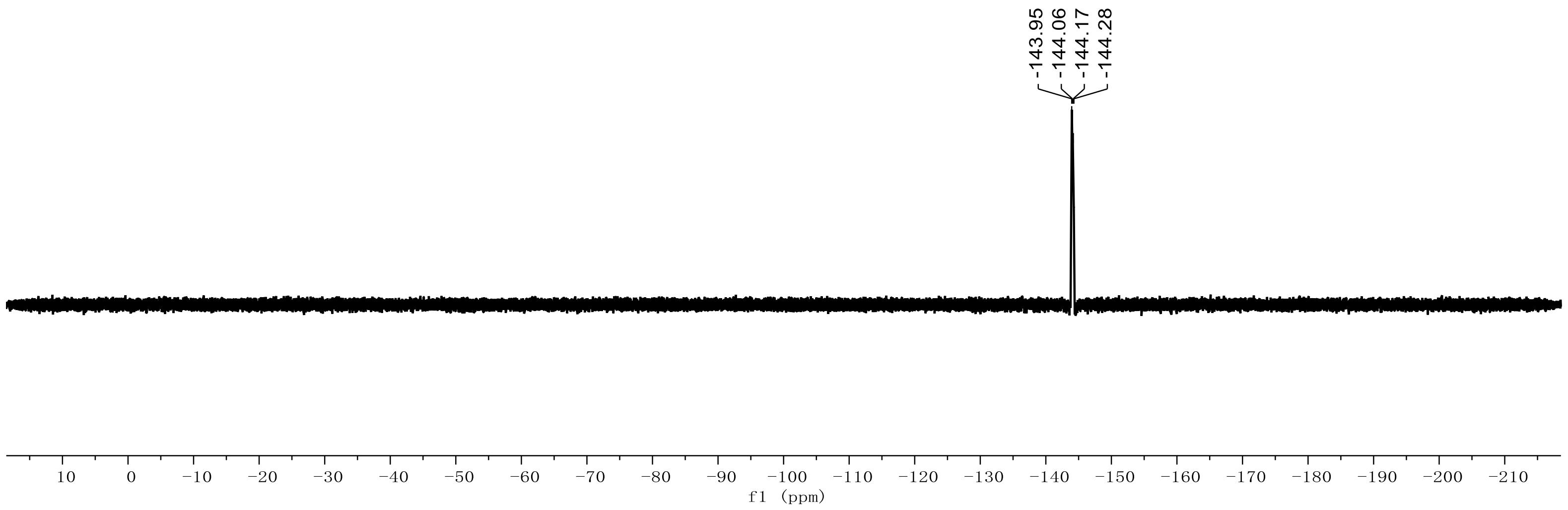

^{11}B NMR of 4a

CD_3CN, 128 MHz, 25 °C

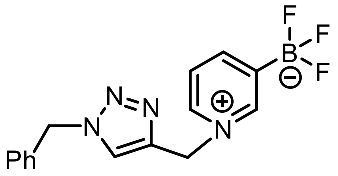

4a

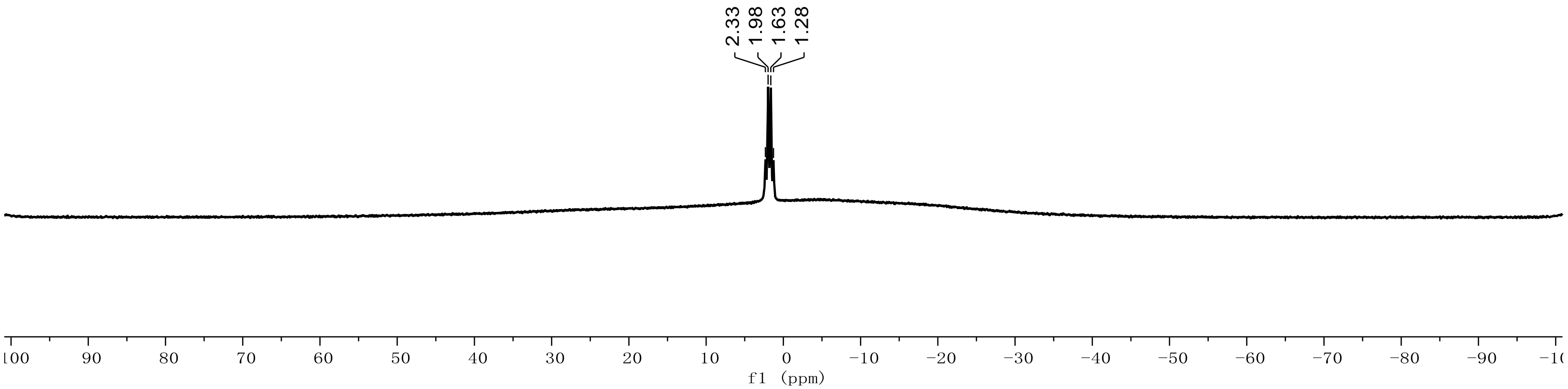

^{1}H NMR of 5a

DMSO-d_6, 400 MHz, 25 °C

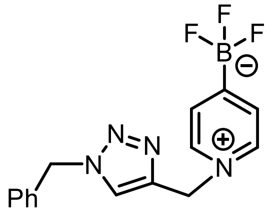

5a

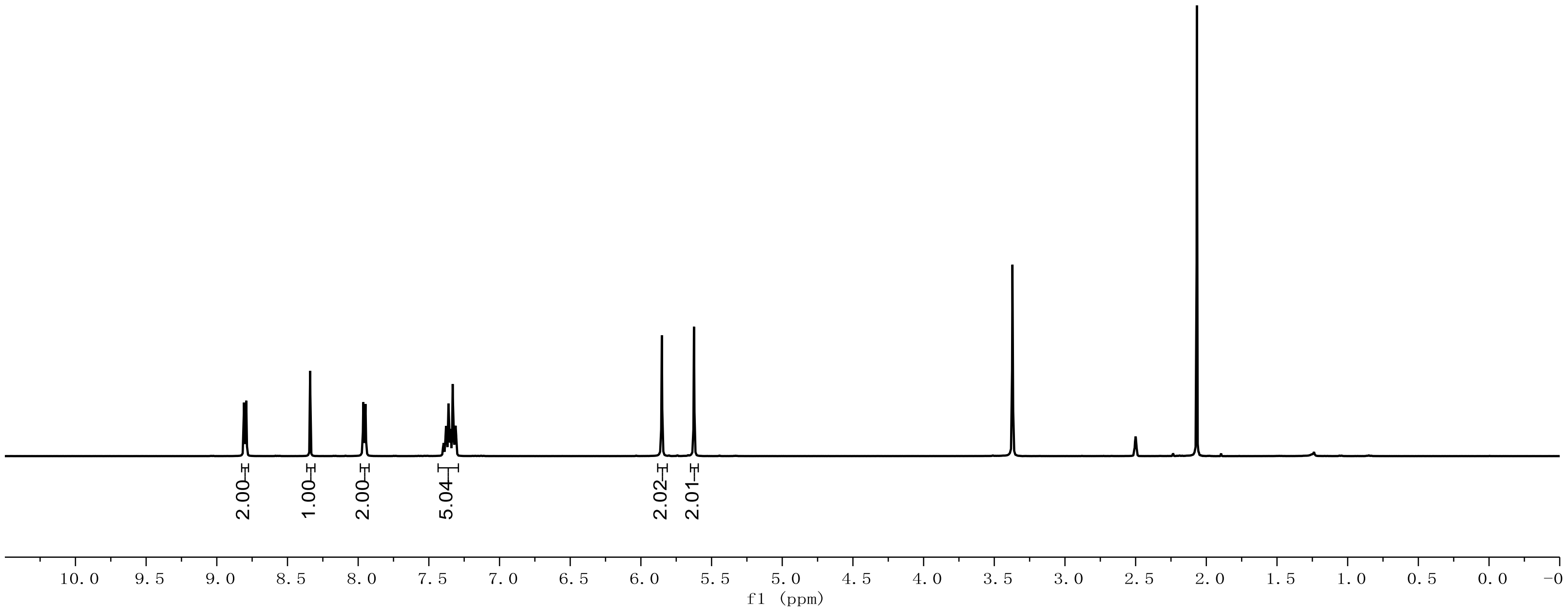

^{13}C NMR of 5a

DMSO-d_6, 101 MHz, 25 °C

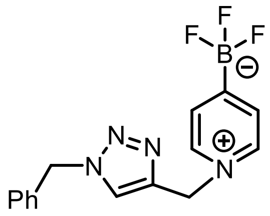

5a

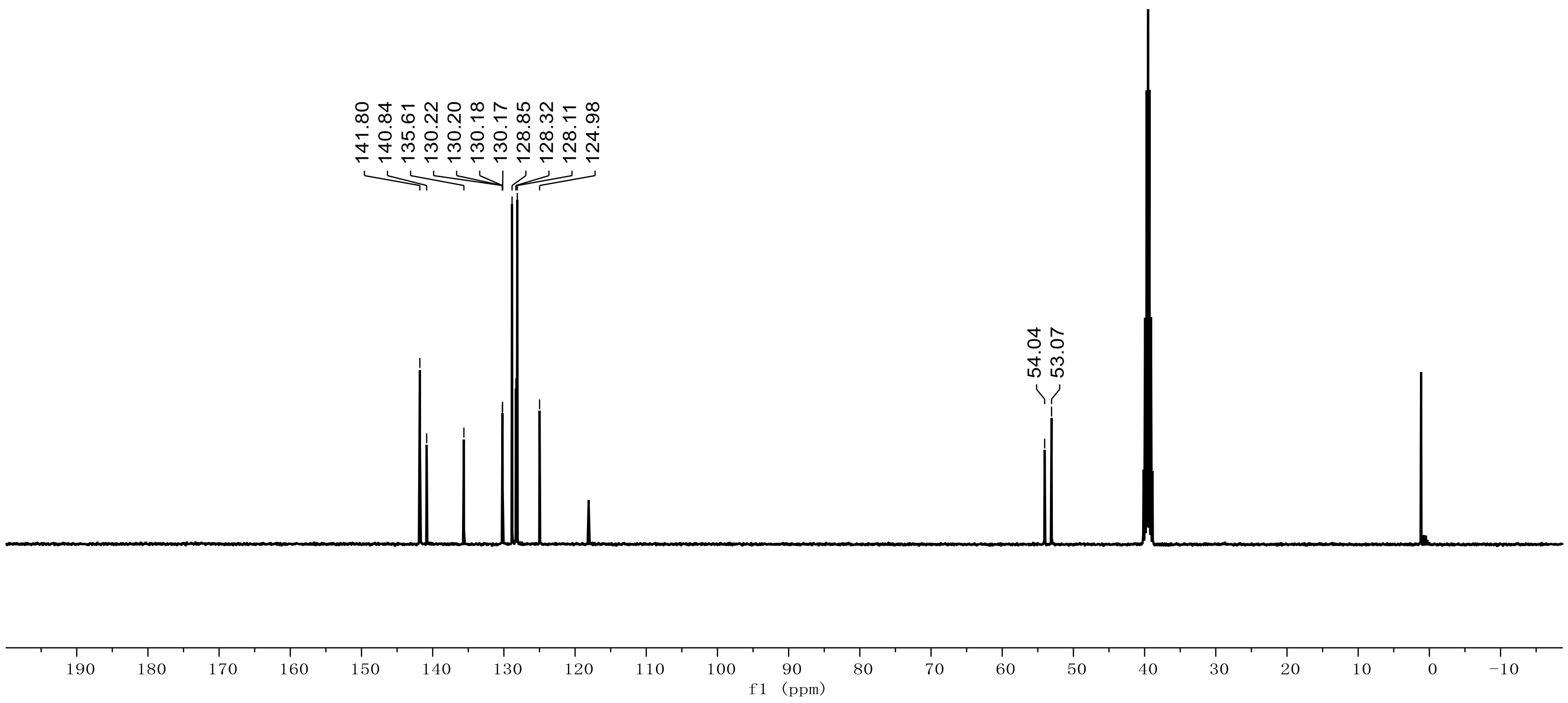

^{19}F NMR of 5a

DMSO-d_6, 376 MHz, 25 °C

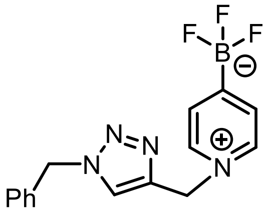

5a

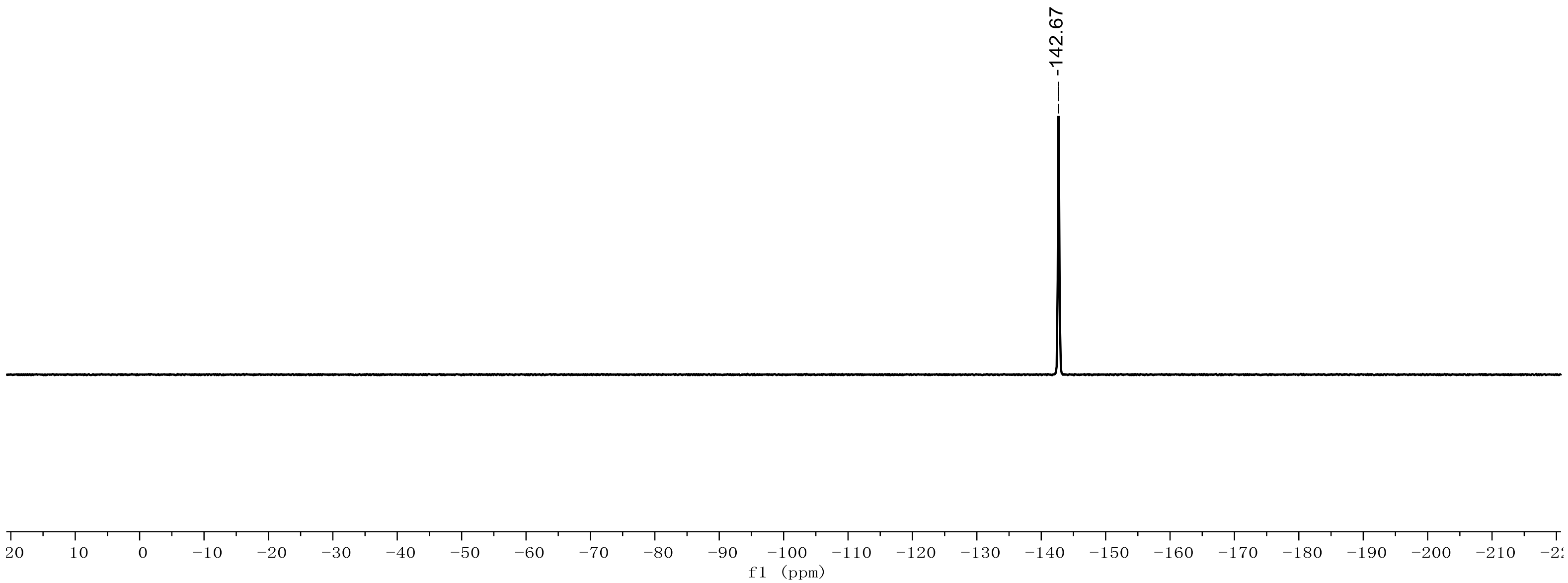

^{11}B NMR of 5a

DMSO-d_6, 128 MHz, 25 °C

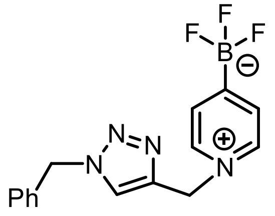

5a

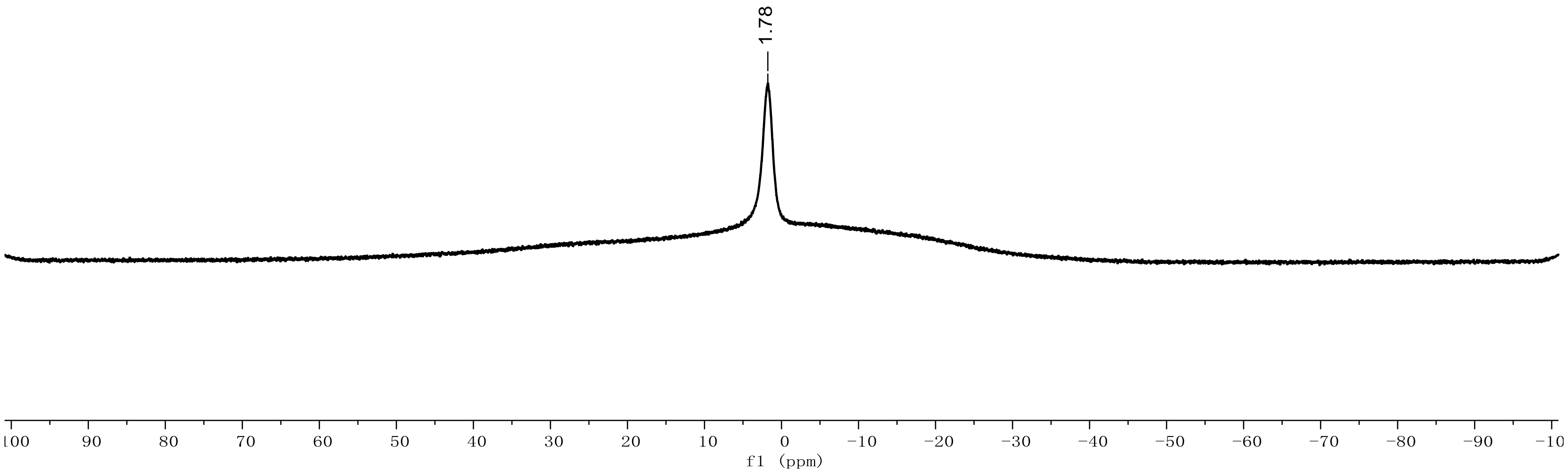

^{1}H NMR of 6a

CD_3CN, 400 MHz, 25 °C

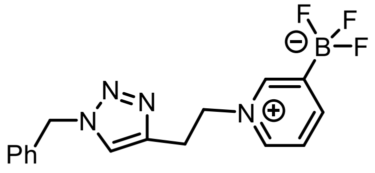

6a

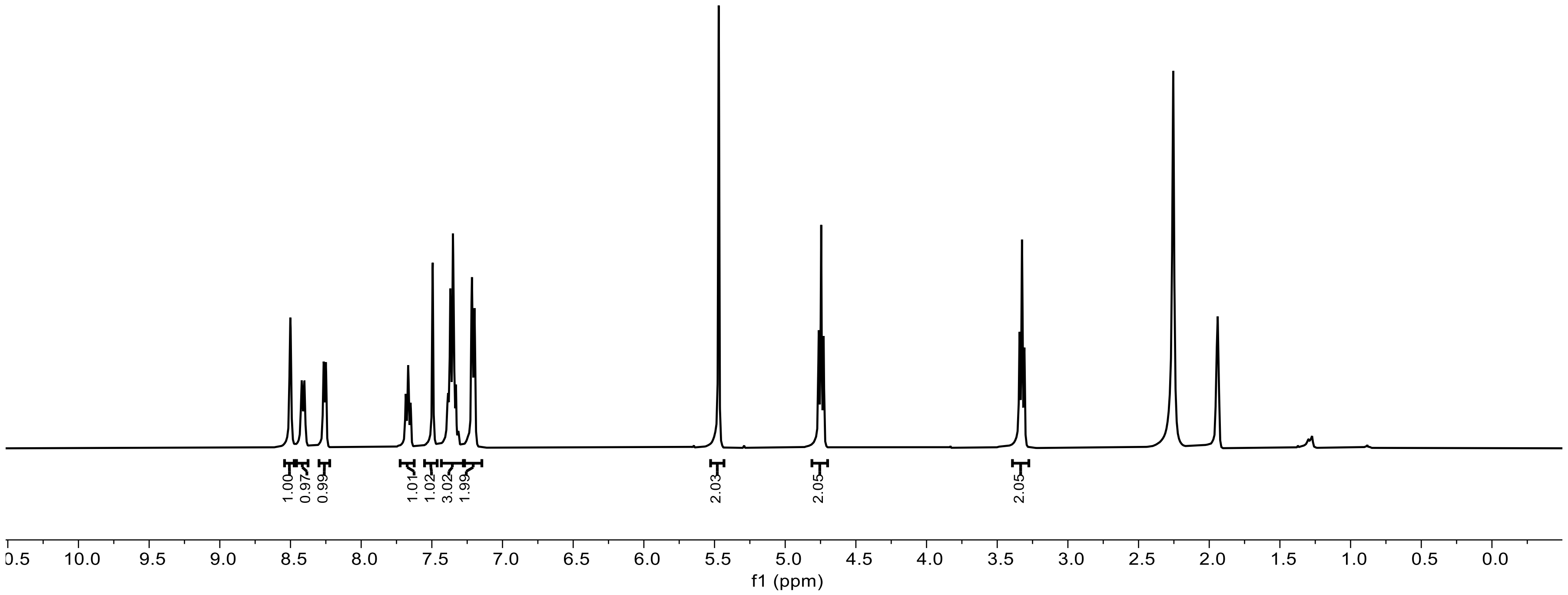

^{13}C NMR of 6a

CD_3CN, 101 MHz, 25 °C

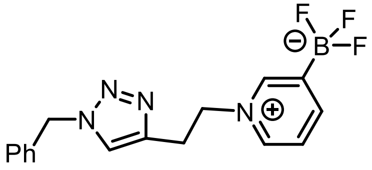

6a

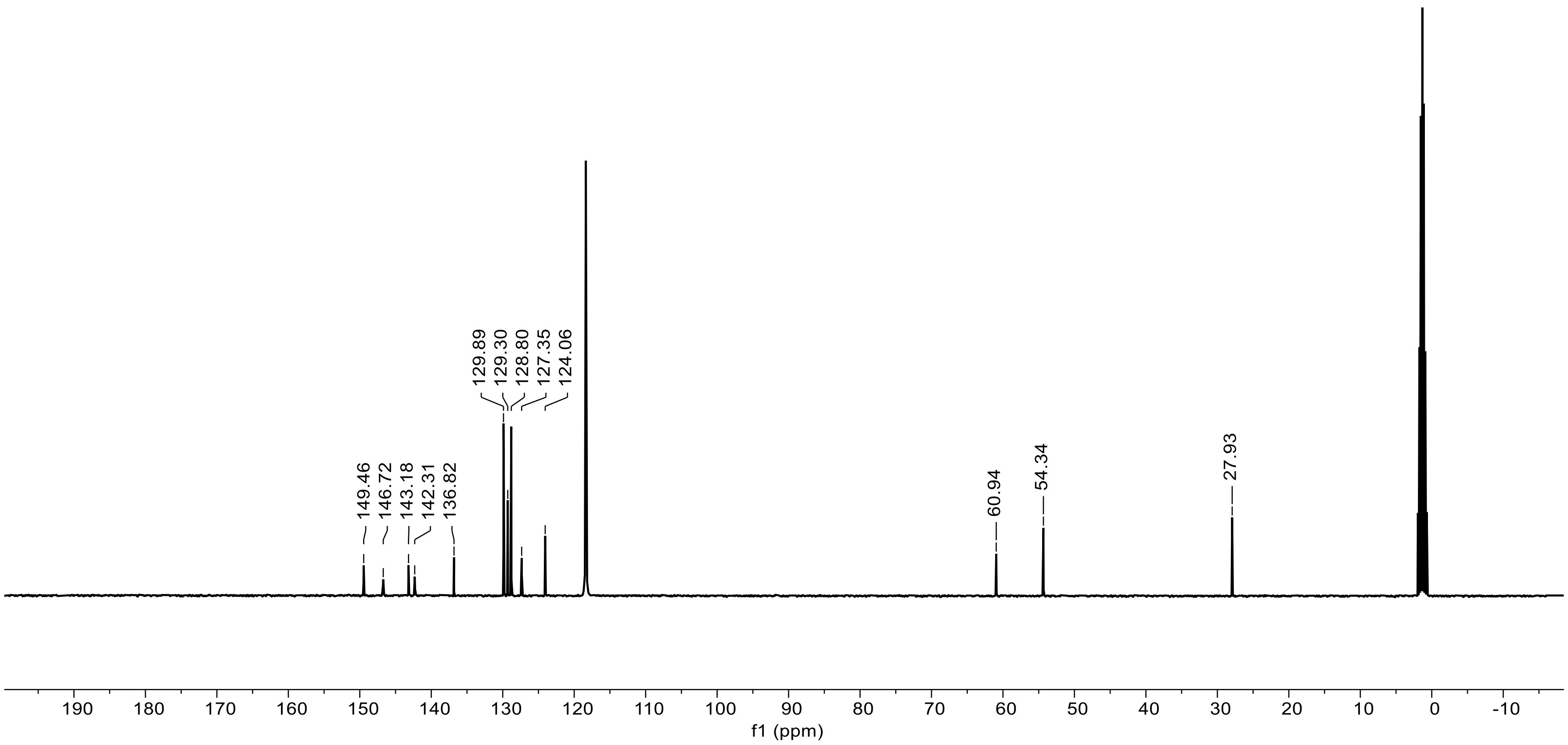

^{19}F NMR of 6a

CD_3CN, 376 MHz, 25 °C

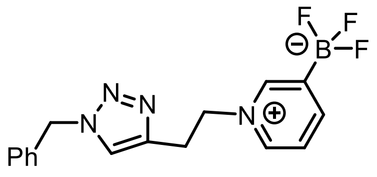

6a

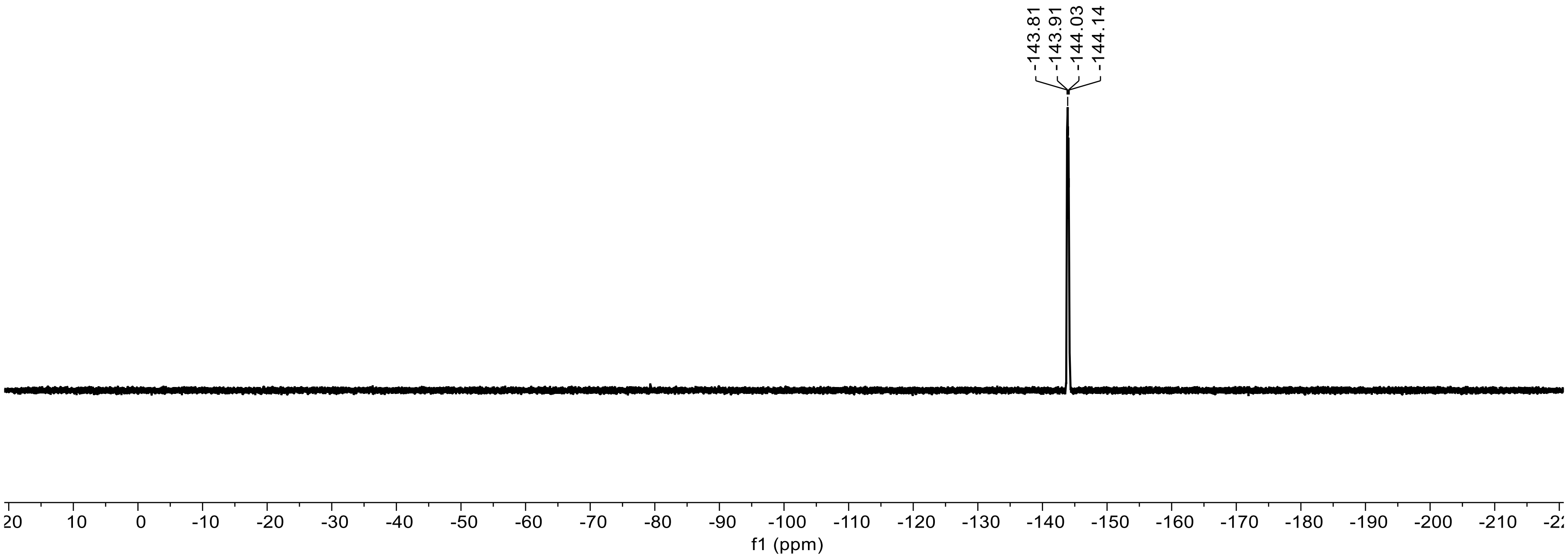

^{11}B NMR of 6a

CD_3CN, 128 MHz, 25 °C

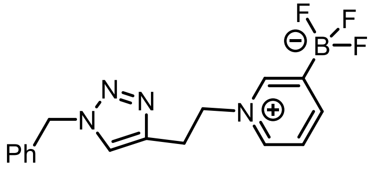

6a

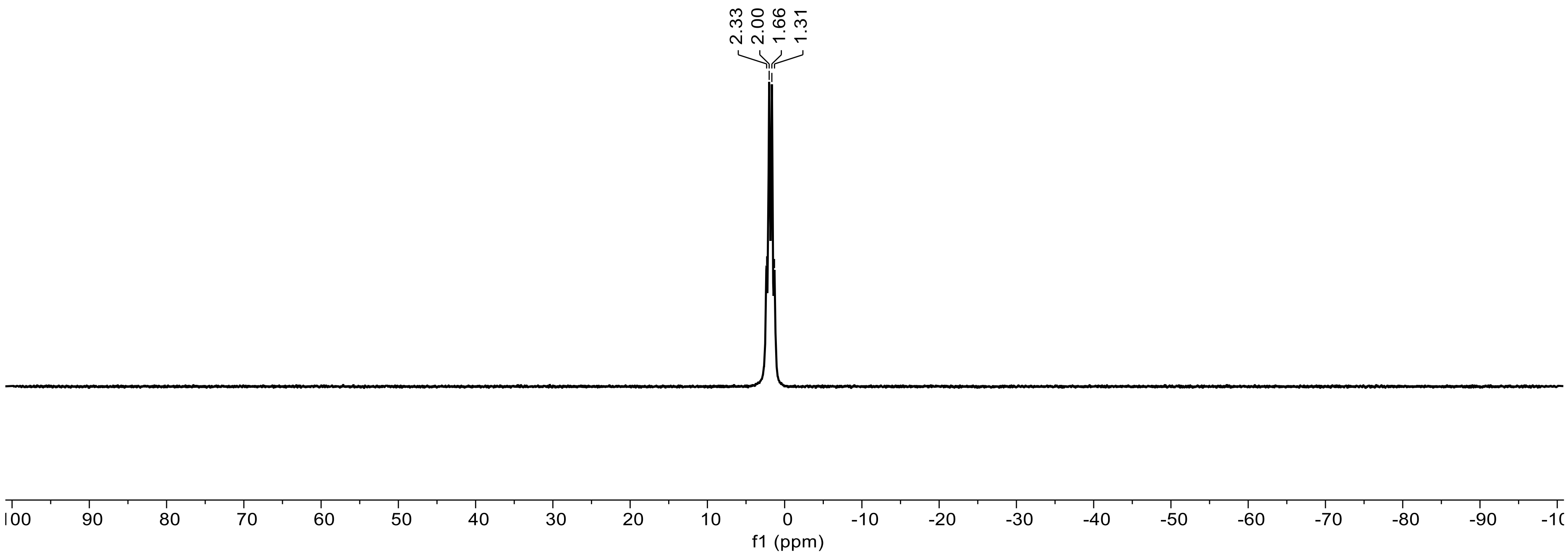

^{1}H NMR of 7a

DMSO-d_6, 400 MHz, 25 °C

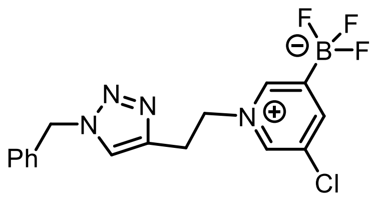

7a

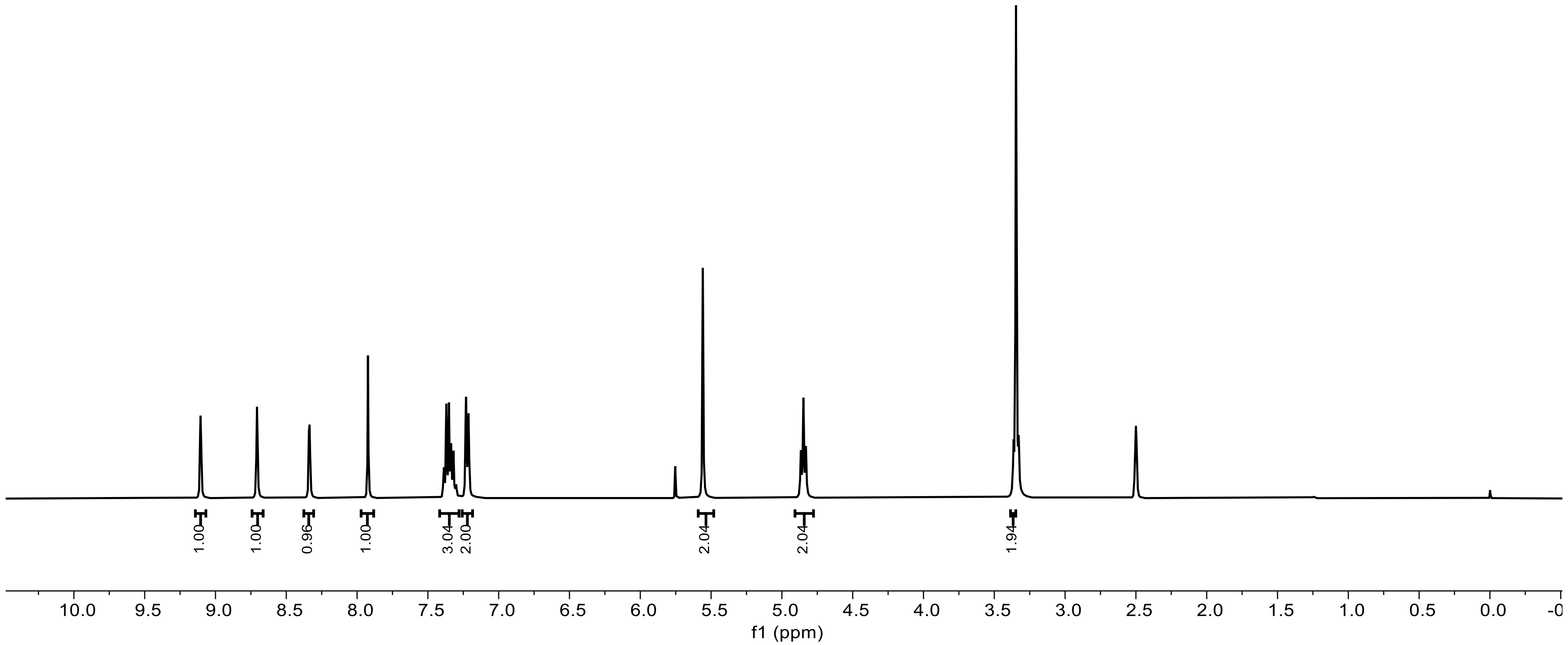

^{13}C NMR of 7a

DMSO-d_6, 101 MHz, 25 °C

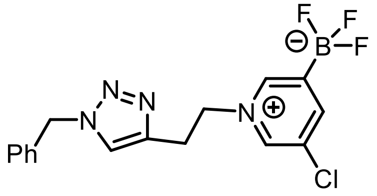

7a

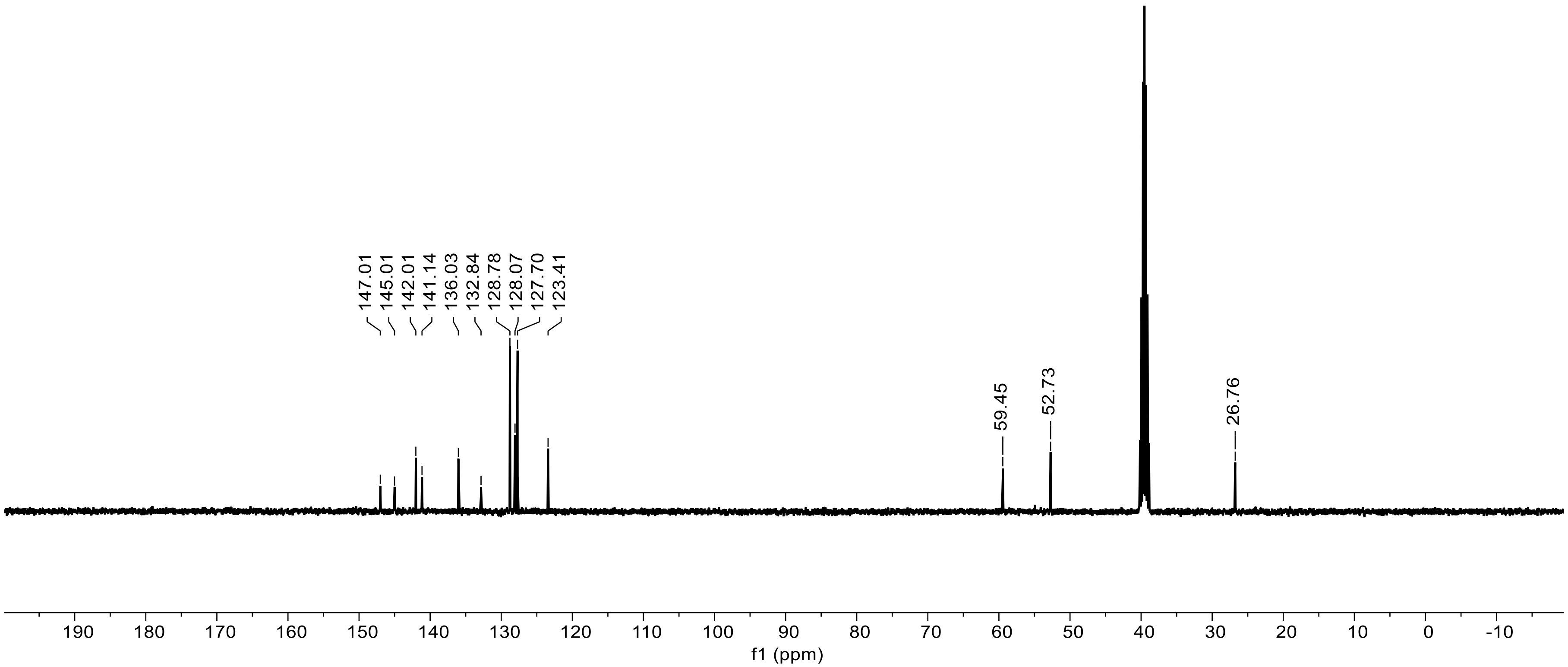

^{19}F NMR of 7a

DMSO-d_6, 376 MHz, 25 °C

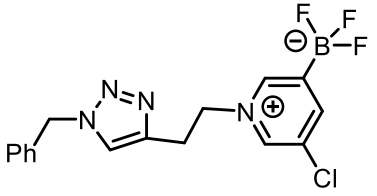

7a

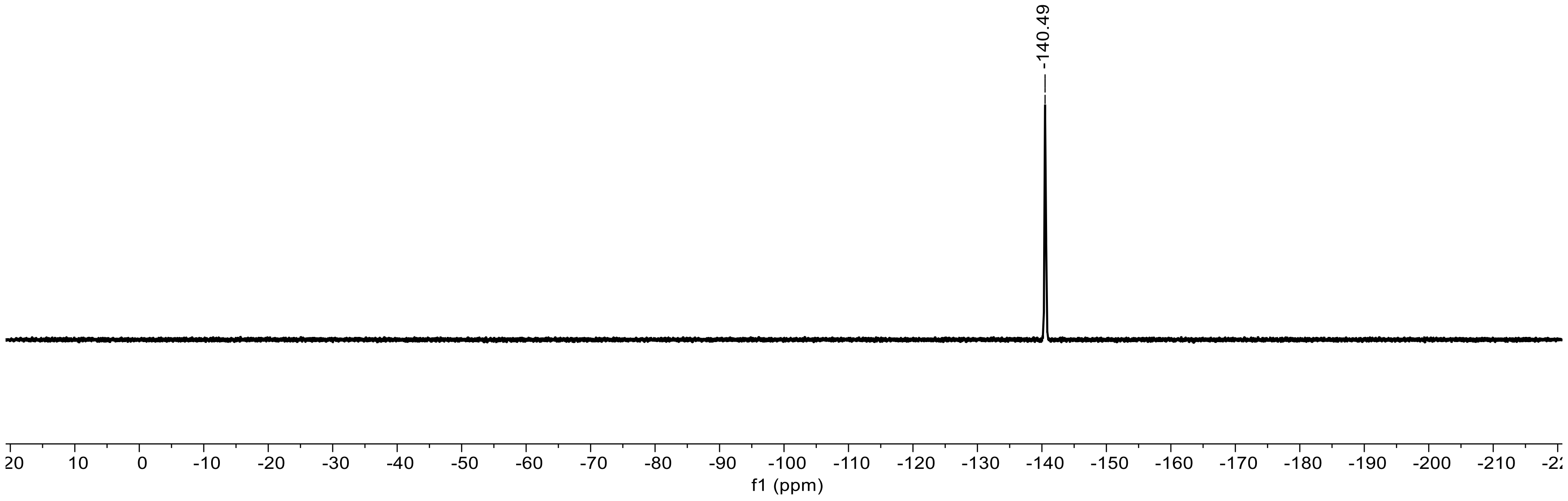

^{11}B NMR of 7a

DMSO-d_6, 128 MHz, 25 °C

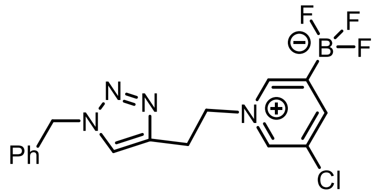

7a

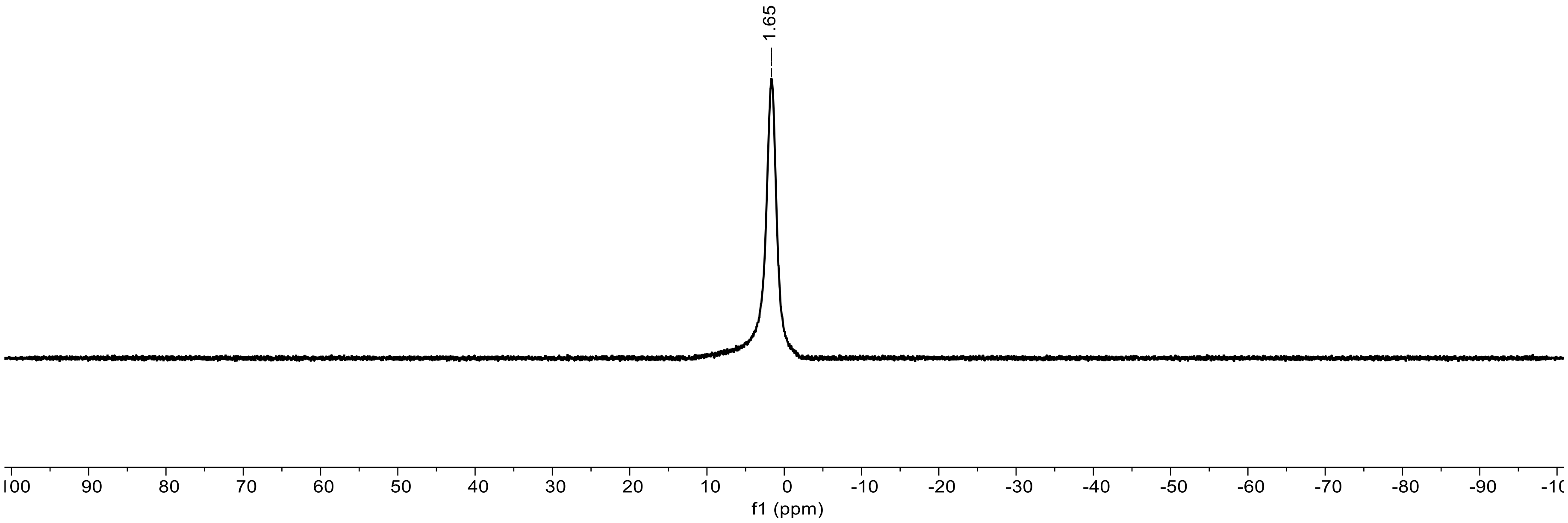

^{1}H NMR of 8a

DMSO-d_6, 400 MHz, 25 °C

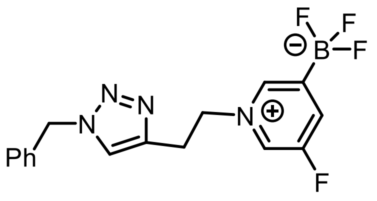

8a

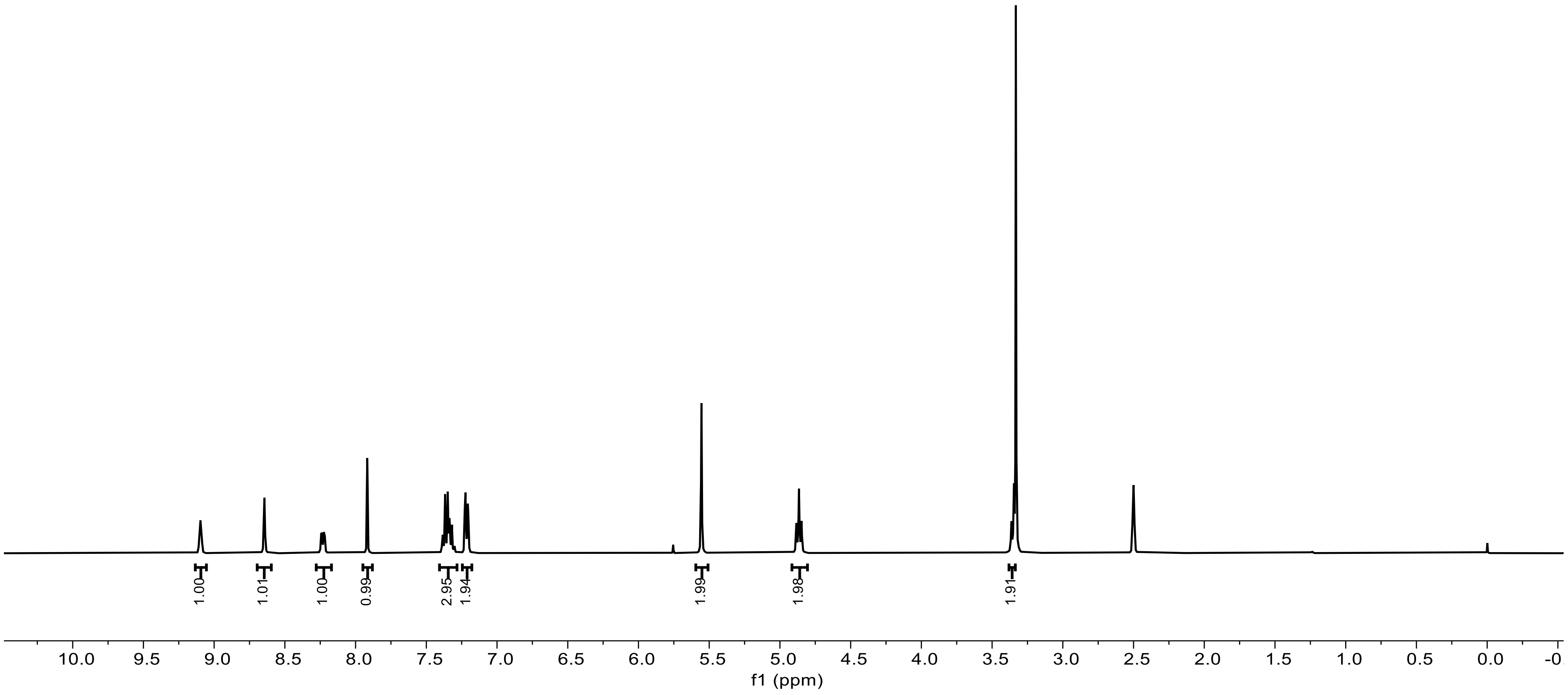

^{13}C NMR of 8a

DMSO-d_6, 101 MHz, 25 °C

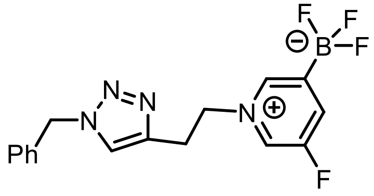

8a

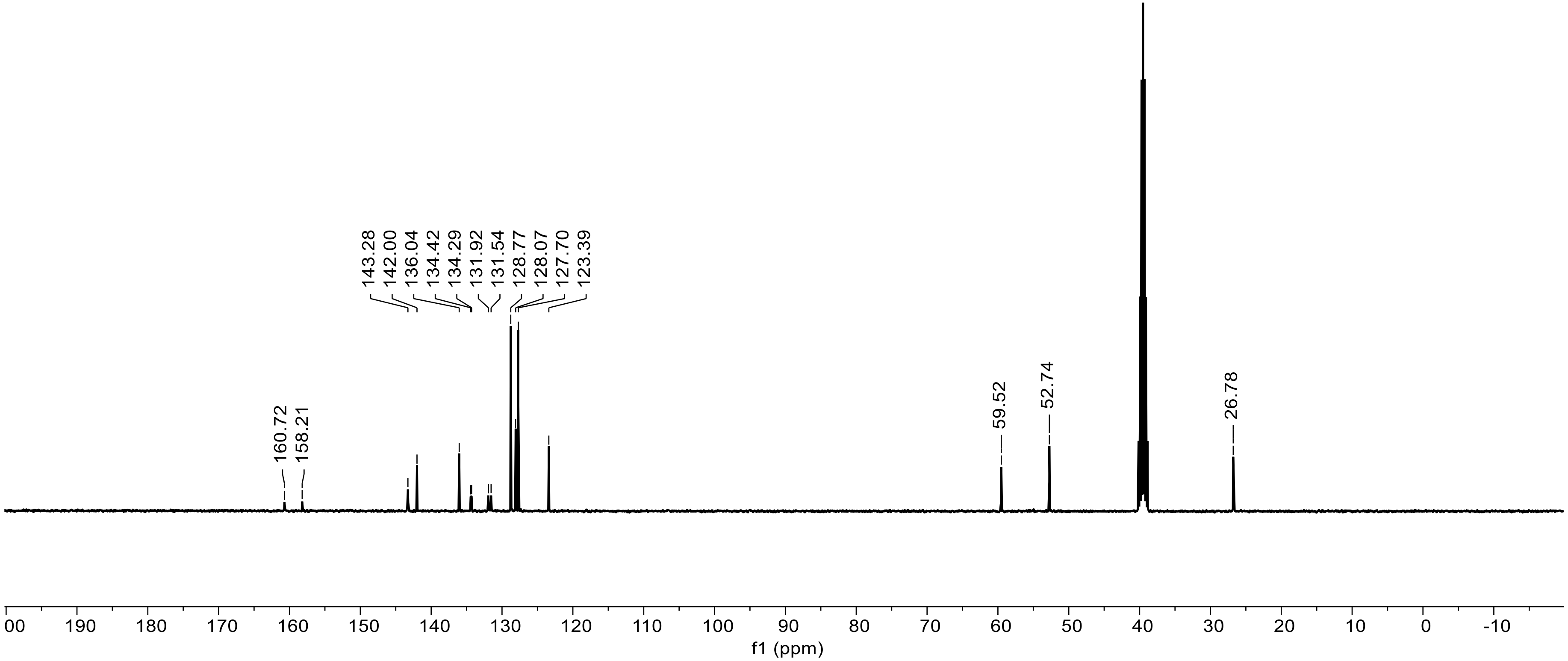

^{19}F NMR of 8a

DMSO-d_6, 376 MHz, 25 °C

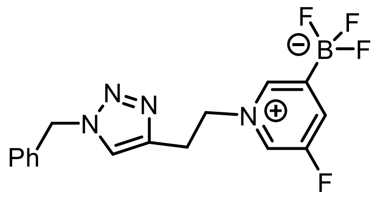

8a

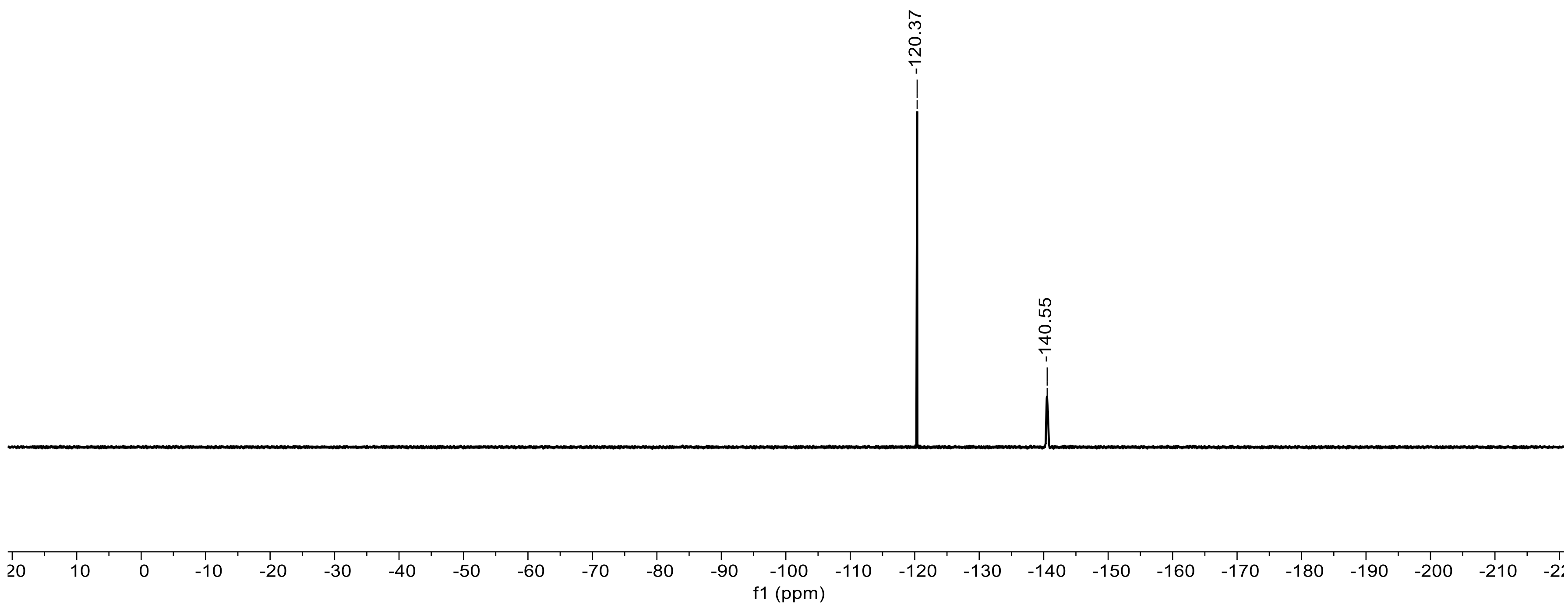

^{11}B NMR of 8a

DMSO-d_6, 128 MHz, 25 °C

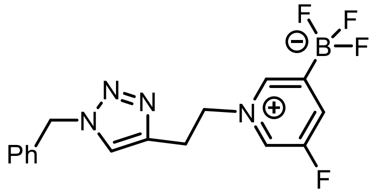

8a

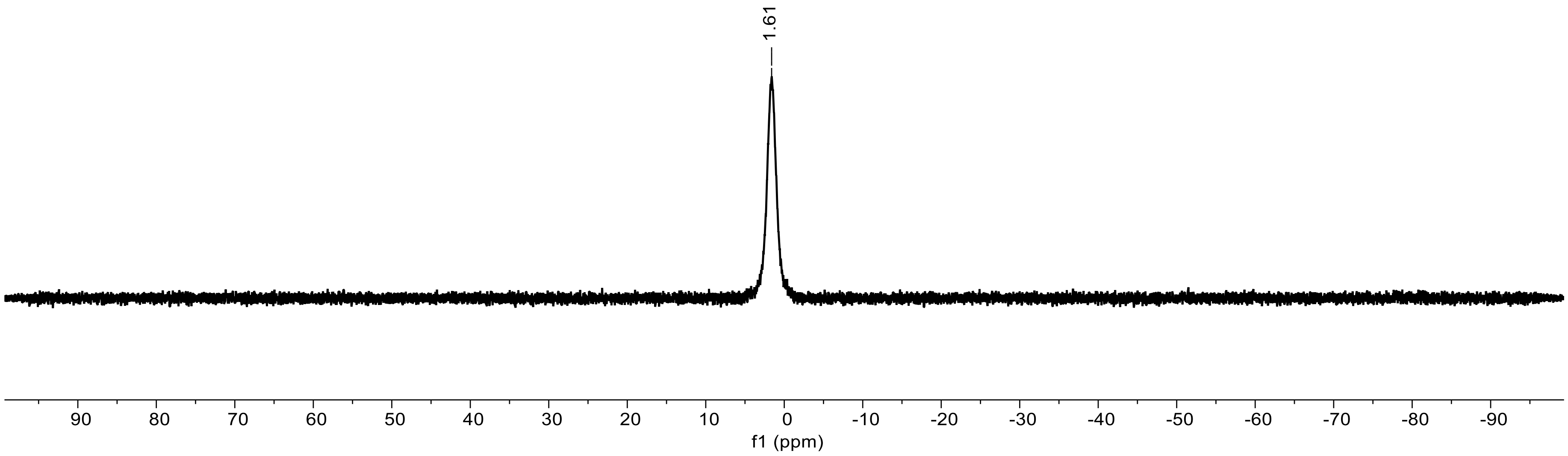

^{1}H NMR of 9a

CD_3CN, 400 MHz, 25 °C

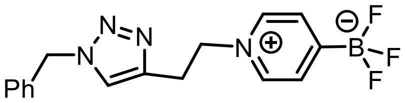

9a

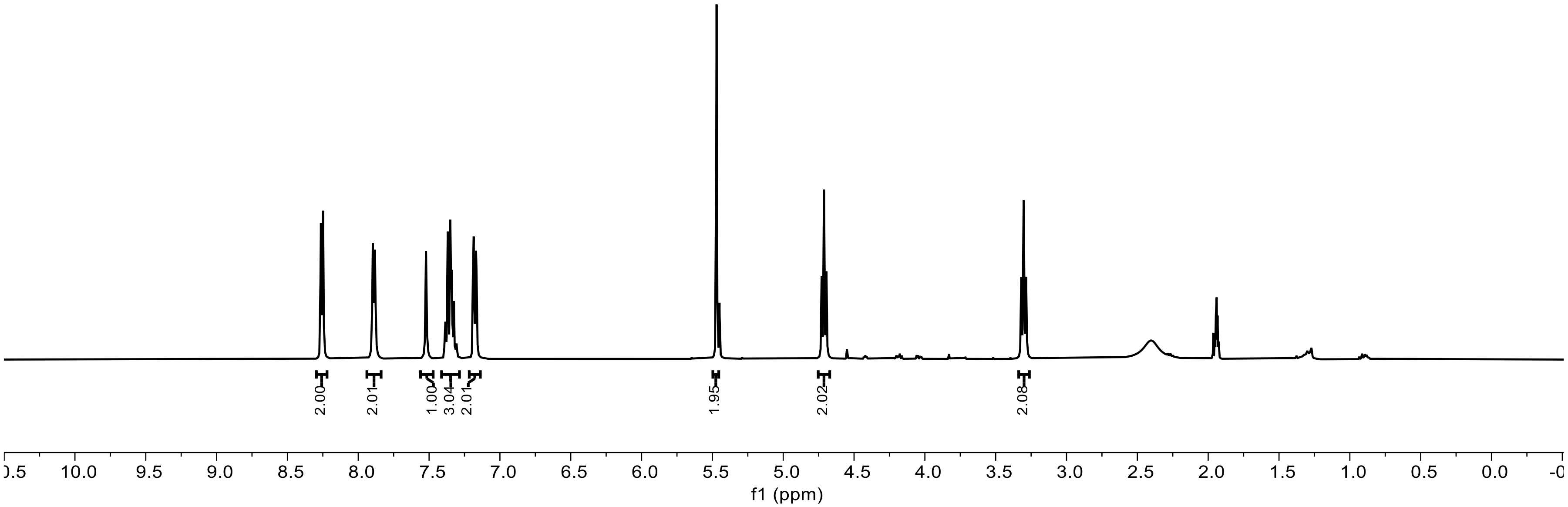

^{13}C NMR of 9a

CD_3CN, 151 MHz, 25 °C

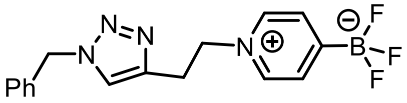

9a

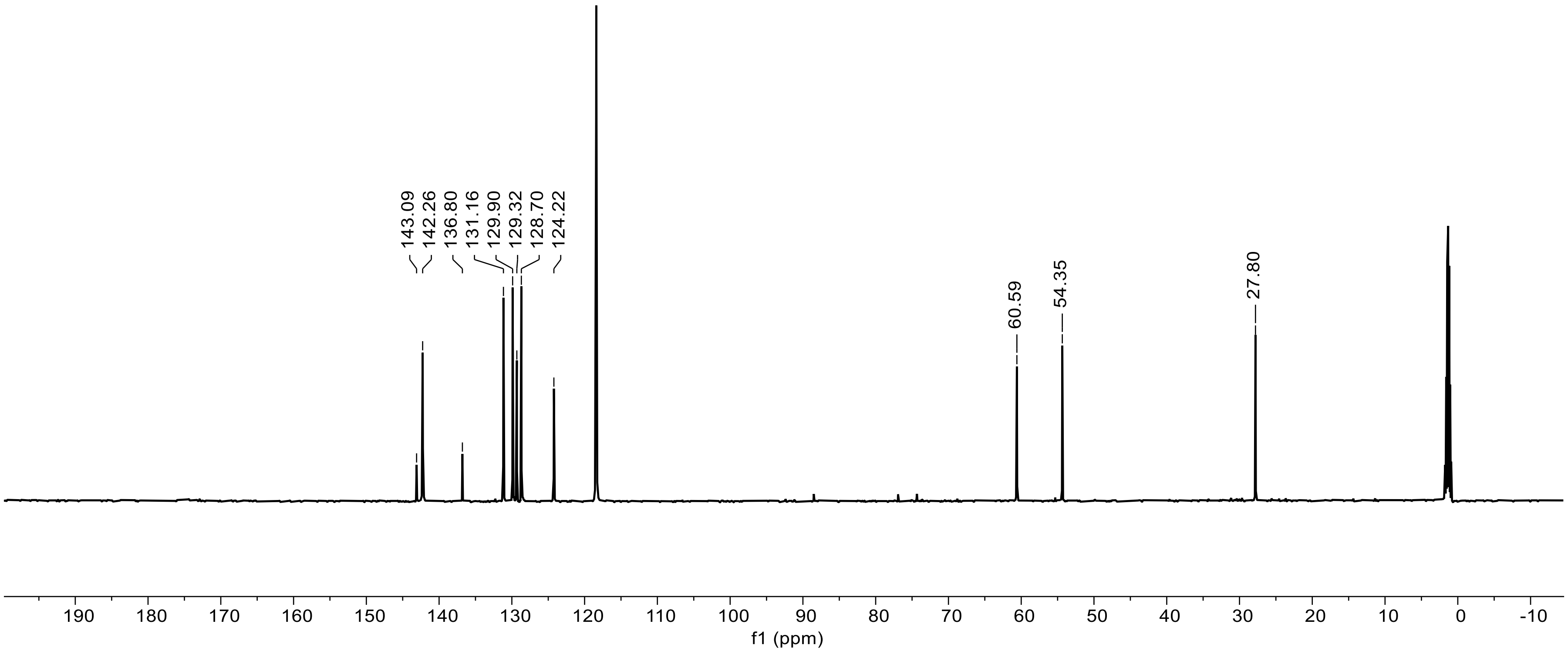

^{19}F NMR of 9a

CD_3CN, 376 MHz, 25 °C

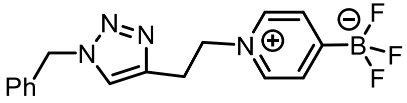

9a

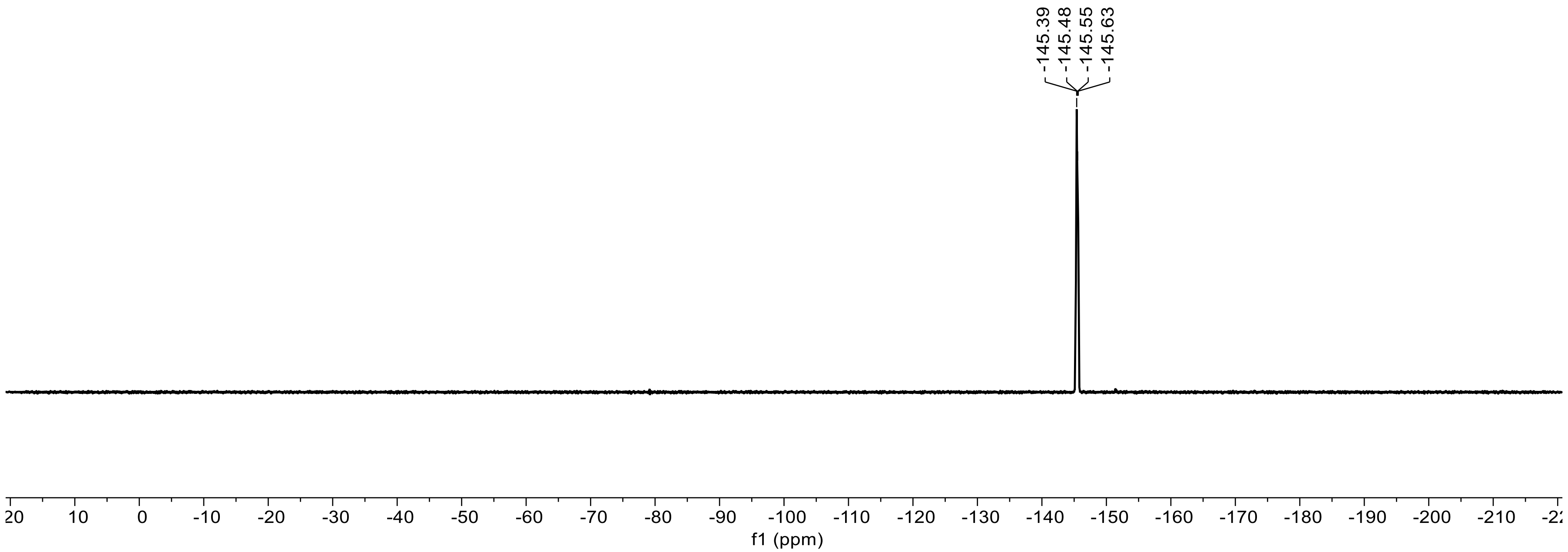

^{11}B NMR of 9a

CD3CN, 128 MHz, 25 °C

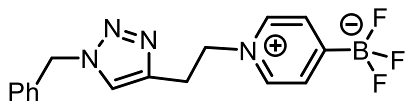

9a

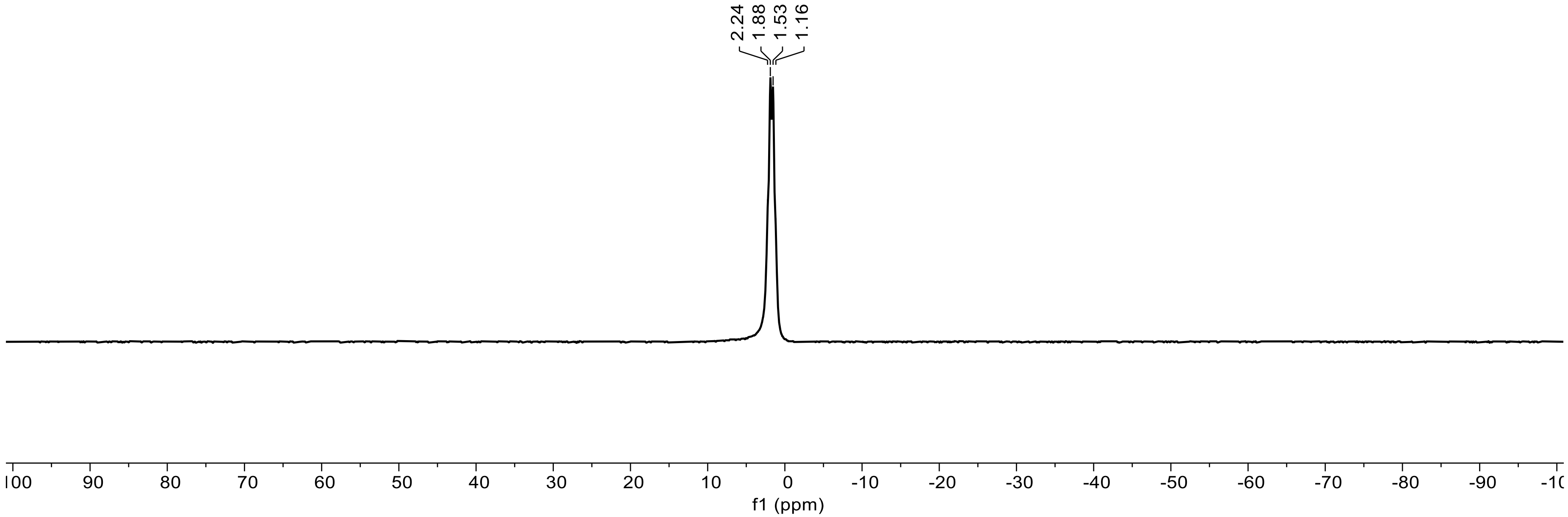

^{1}H NMR of 10a

CD_3CN, 400 MHz, 25 °C

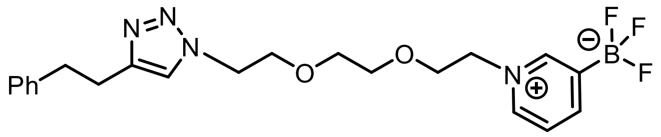

10a

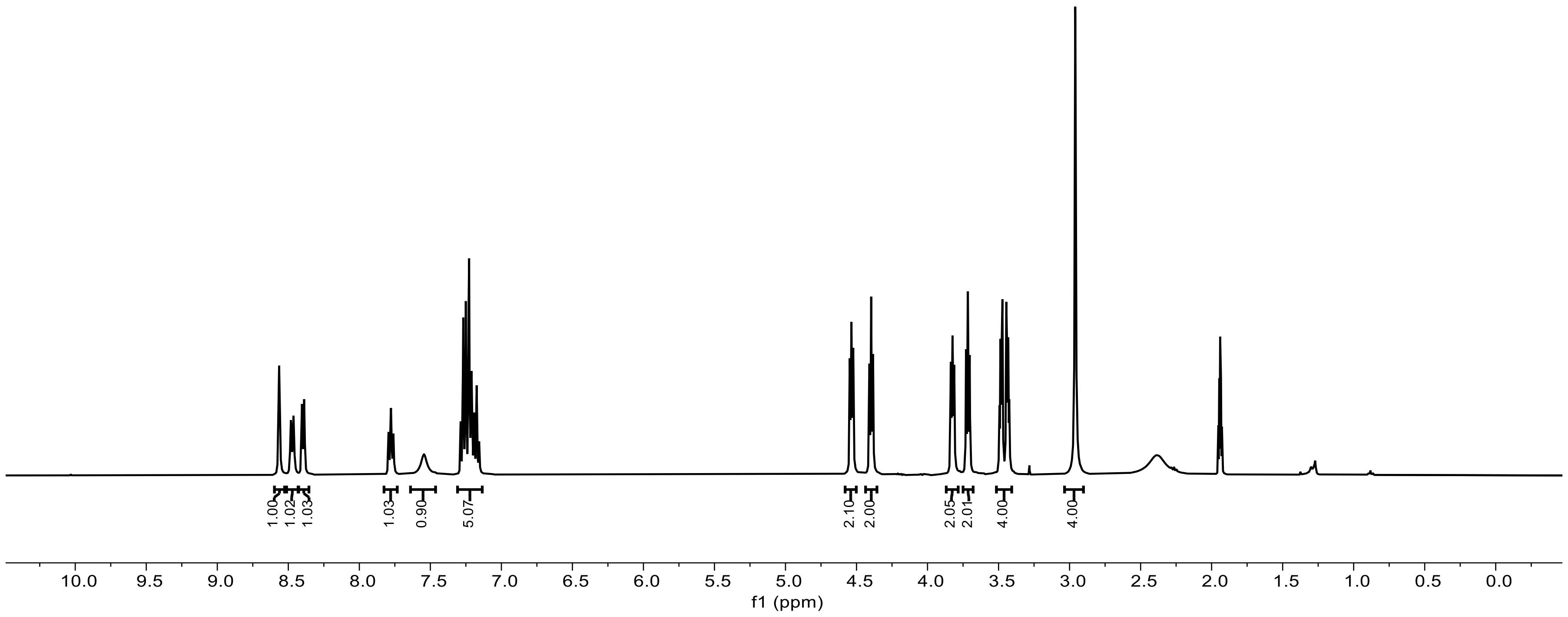

^{13}C NMR of 10a

CD_3CN, 101 MHz, 25 °C

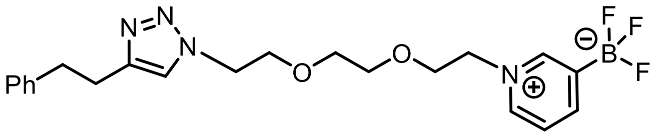

10a

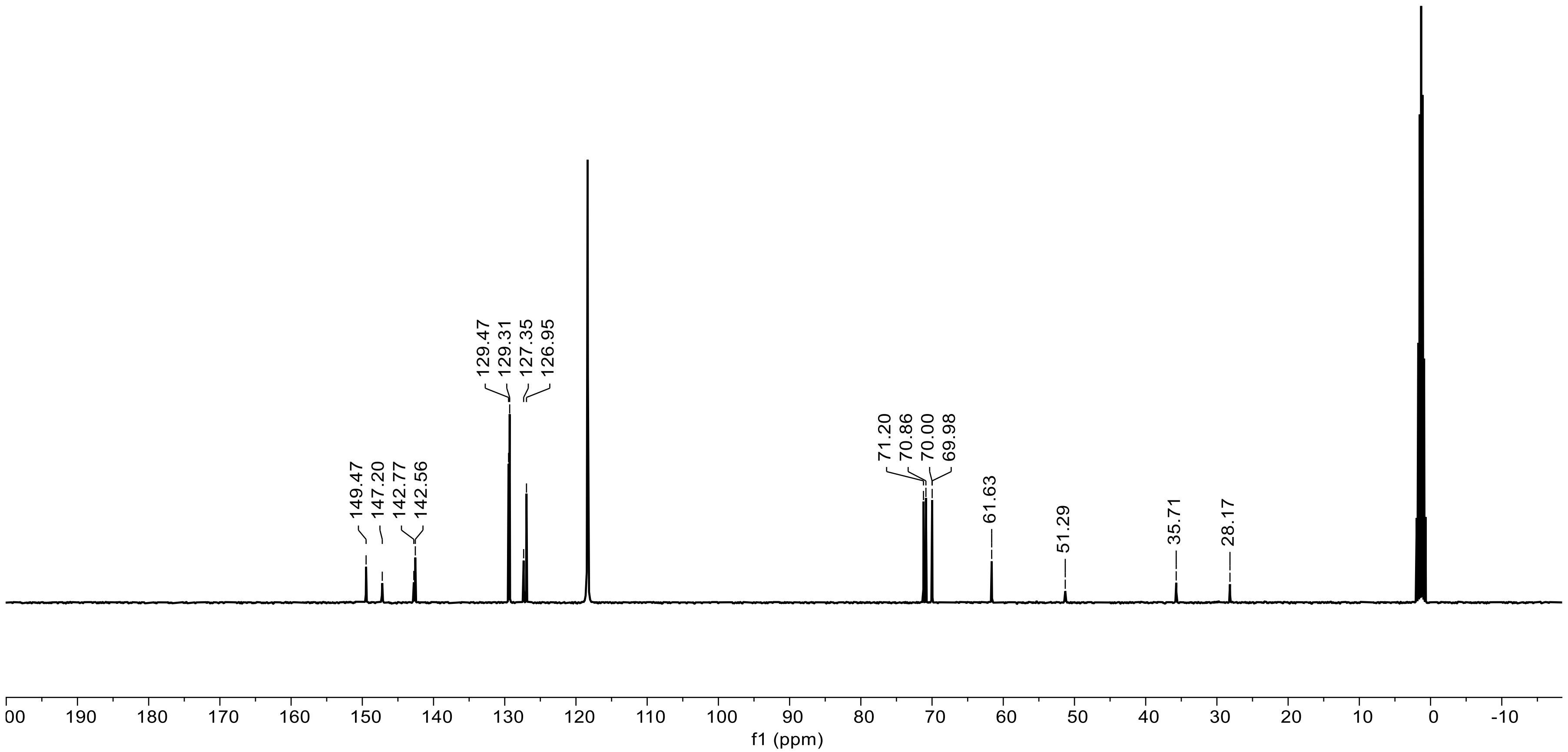

^{19}F NMR of 10a

CD_3CN, 376 MHz, 25 °C

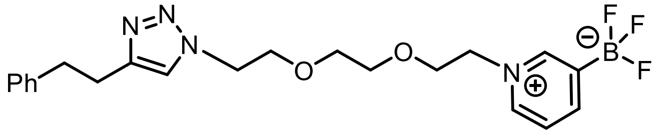

10a

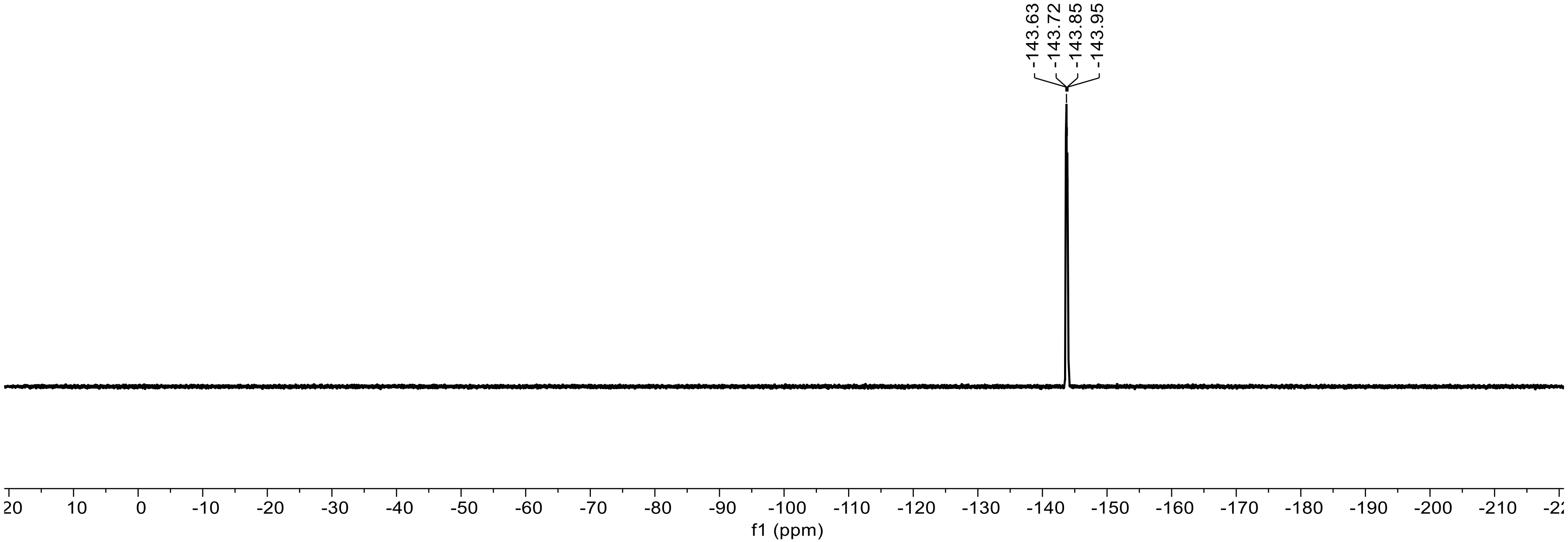

^{11}B NMR of 10a

CD_3CN, 128 MHz, 25 °C

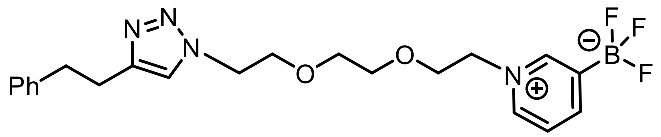

10a

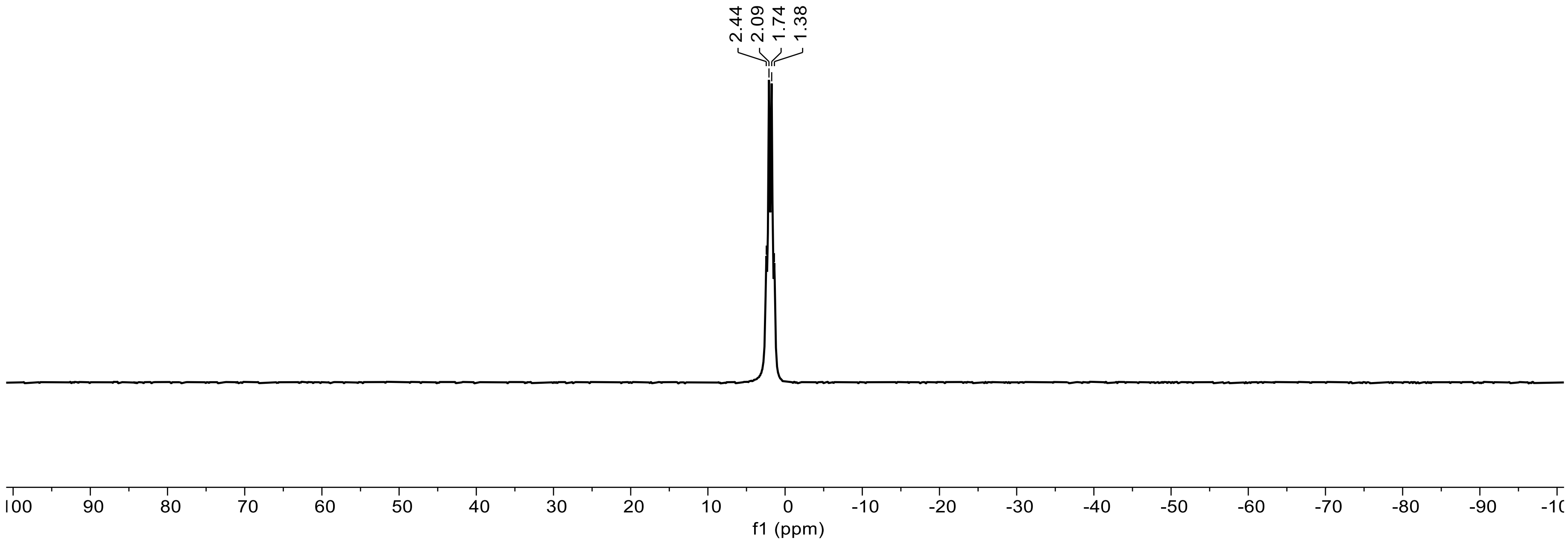

^{1}H NMR of 11a

DMSO-d_6, 400 MHz, 25 °C

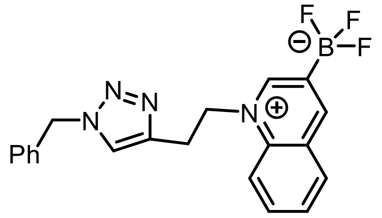

11a

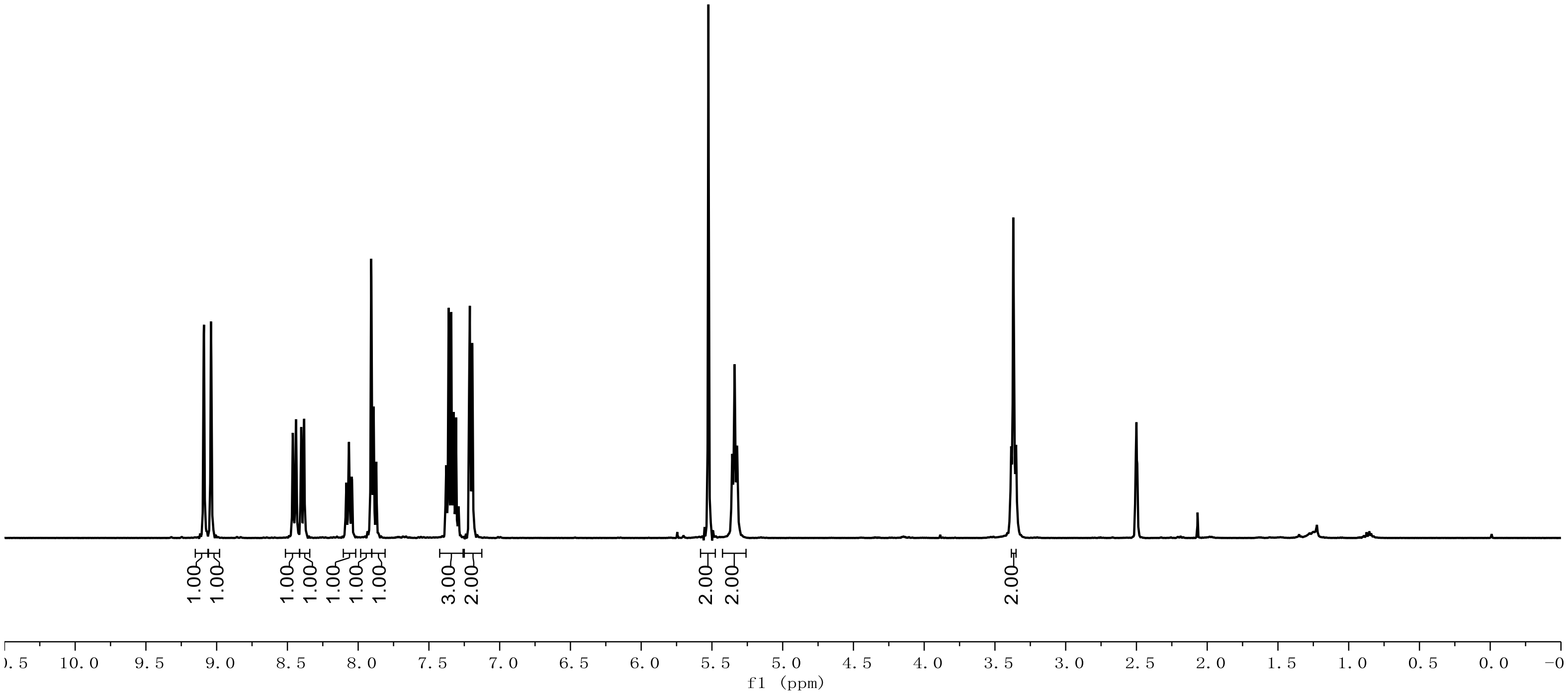

^{13}C NMR of 11a

DMSO-d_6, 101 MHz, 25 °C

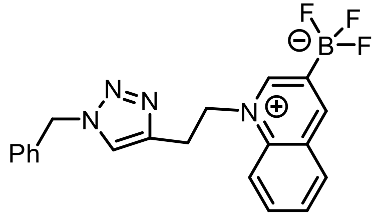

11a

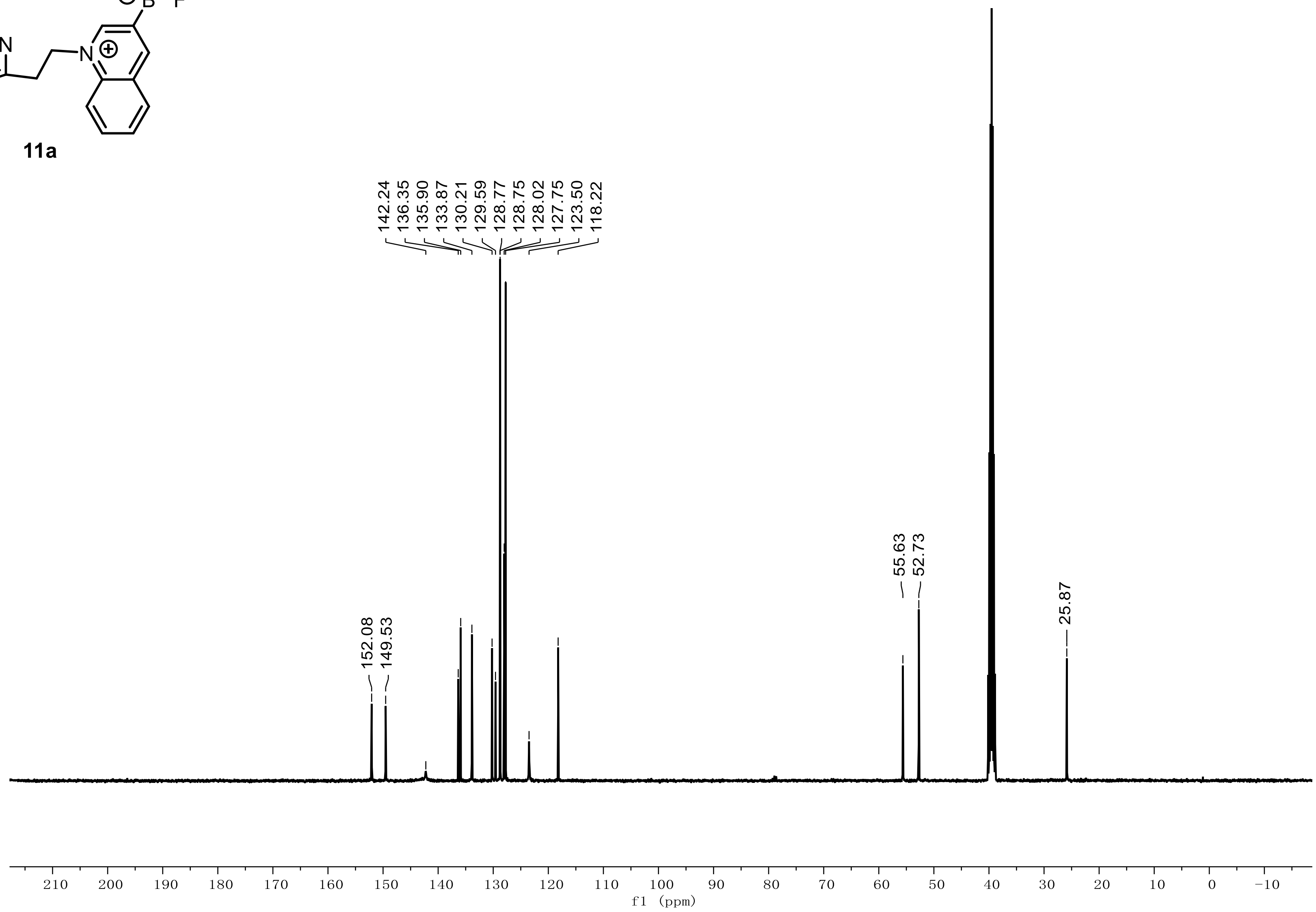

^{19}F NMR of 11a

CD_3CN, 376 MHz, 25 °C

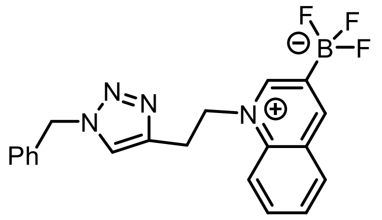

11a

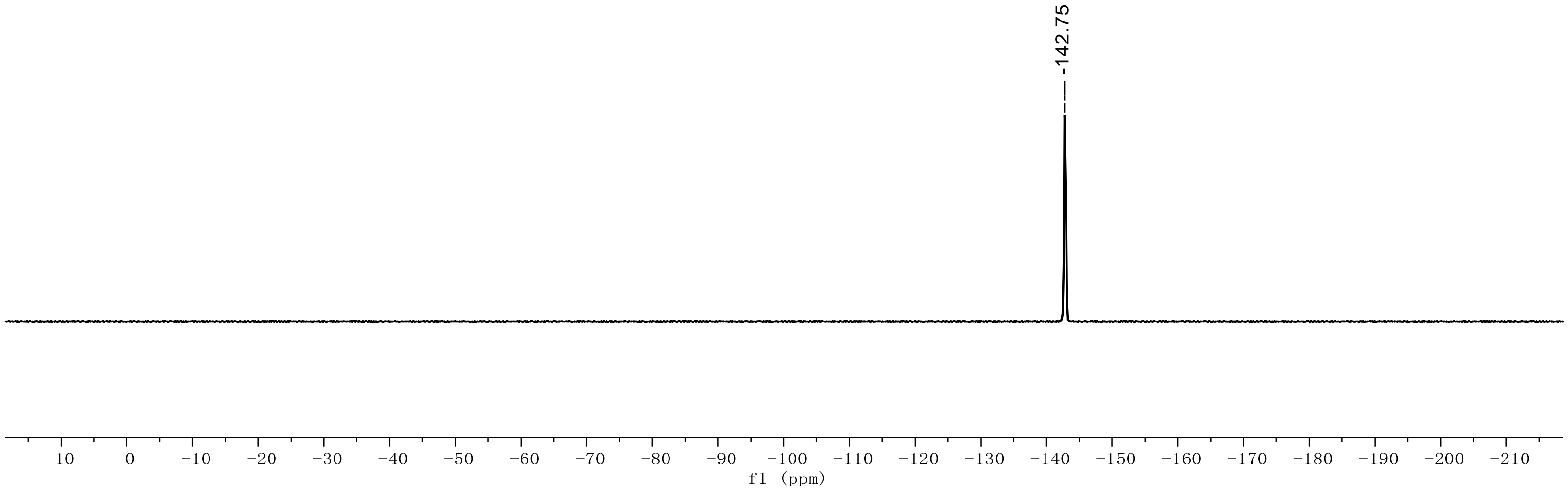

^{11}B NMR of 11a

DMSO-d_6, 128 MHz, 25 °C

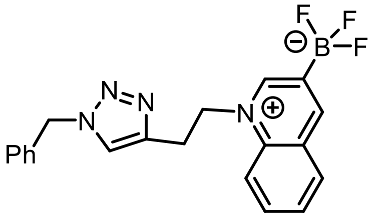

11a

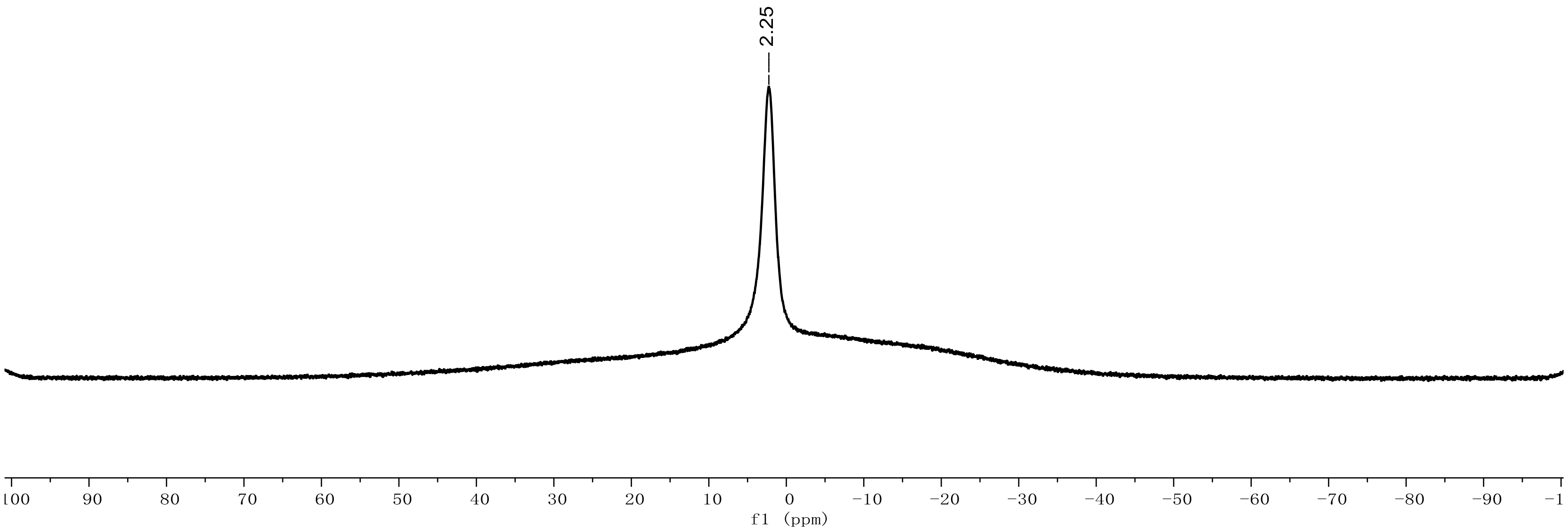

^{1}H NMR of 12a

DMSO-d_6, 400 MHz, 25 °C

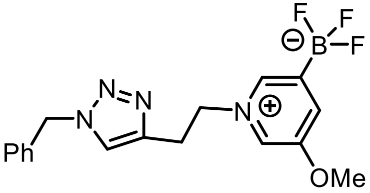

12a

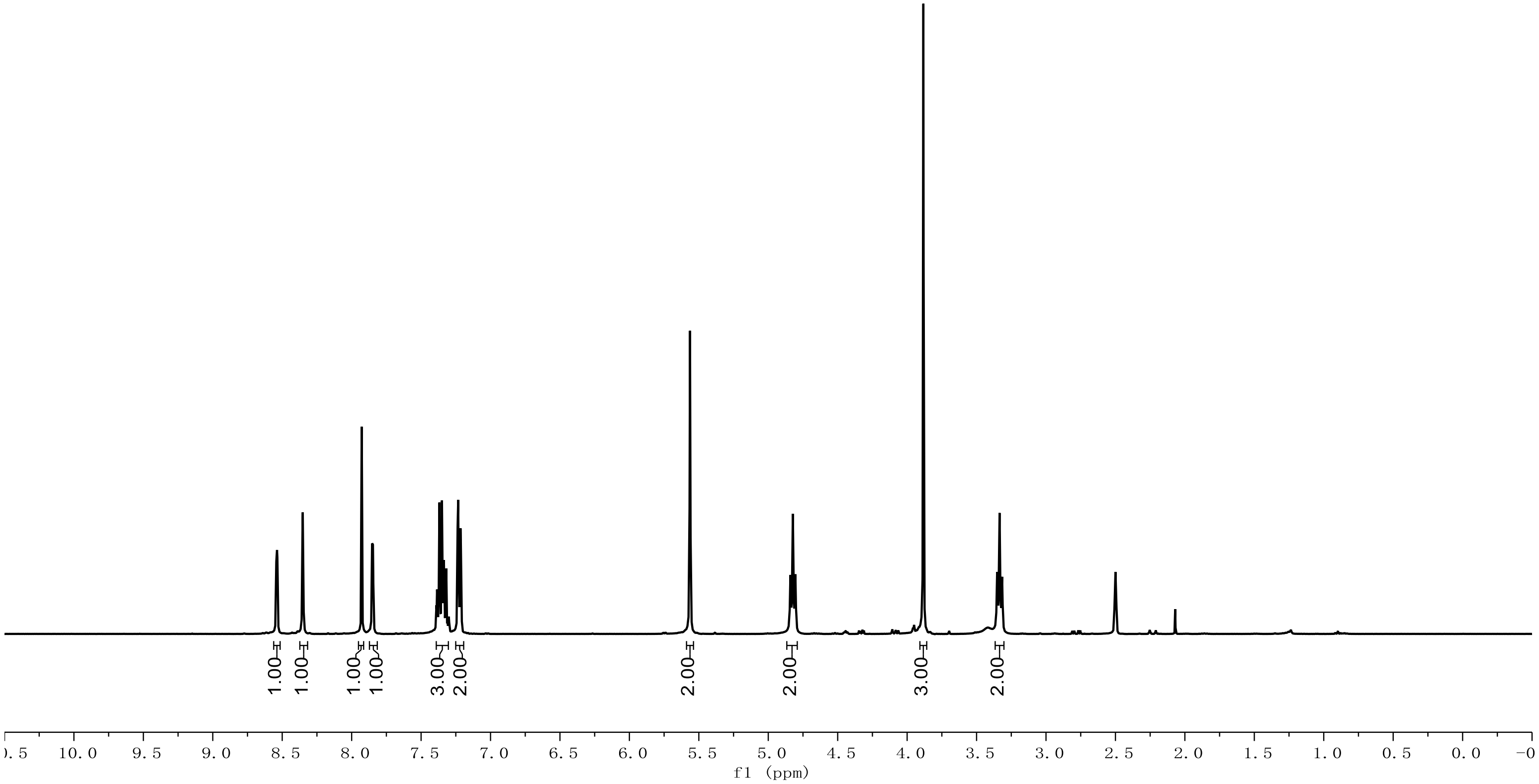

^{13}C NMR of 12a

DMSO-d_6, 101 MHz, 25 °C

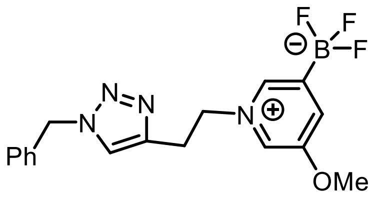

12a

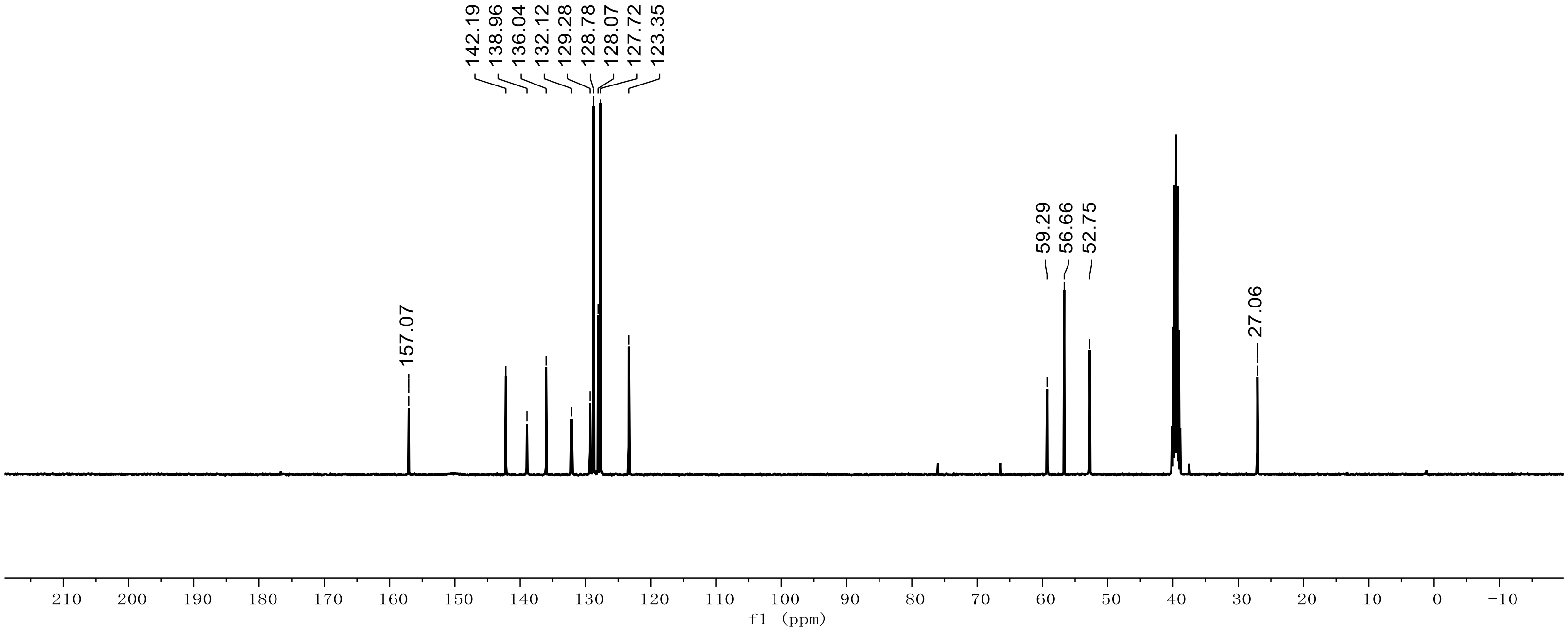

^{19}F NMR of 12a

CD_3CN, 376 MHz, 25 °C

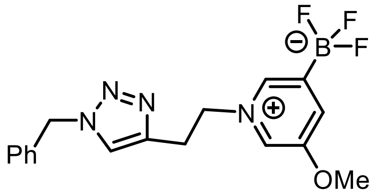

12a

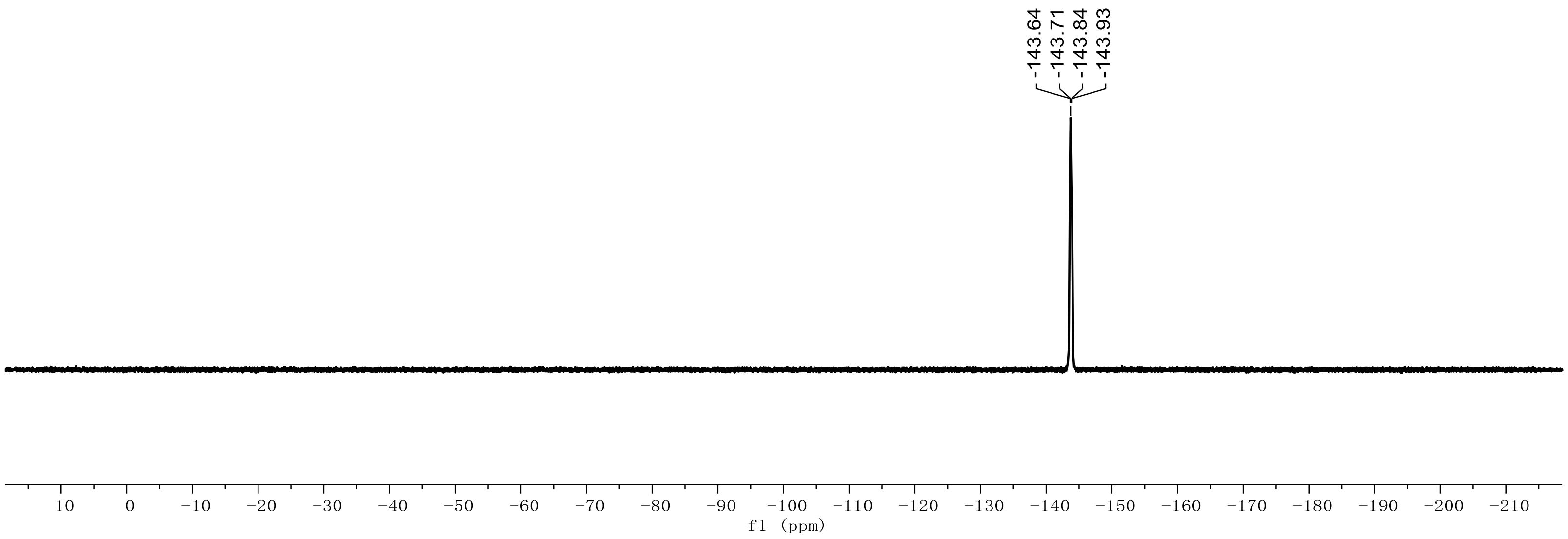

^{11}B NMR of 12a

CD_3CN, 128 MHz, 25 °C

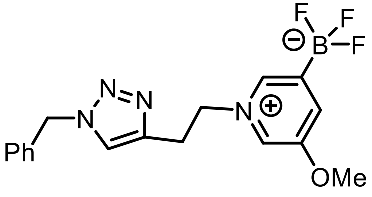

12a

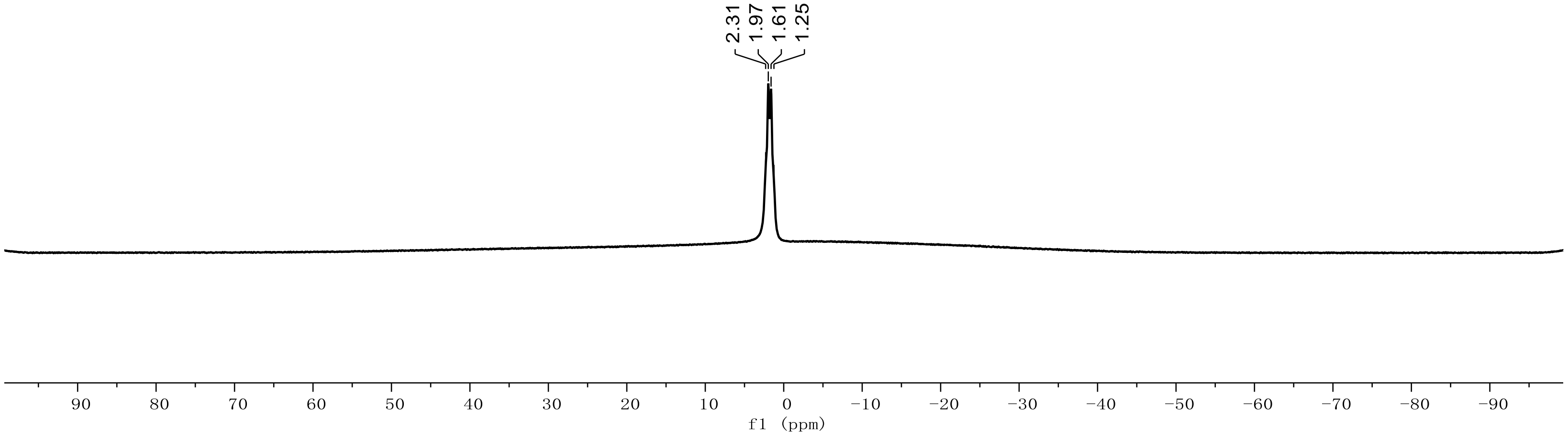

^{1}H NMR of 13a

DMSO-d_6, 400 MHz, 25 °C

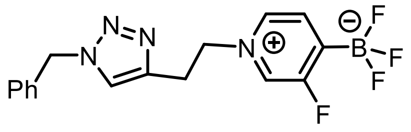

13a

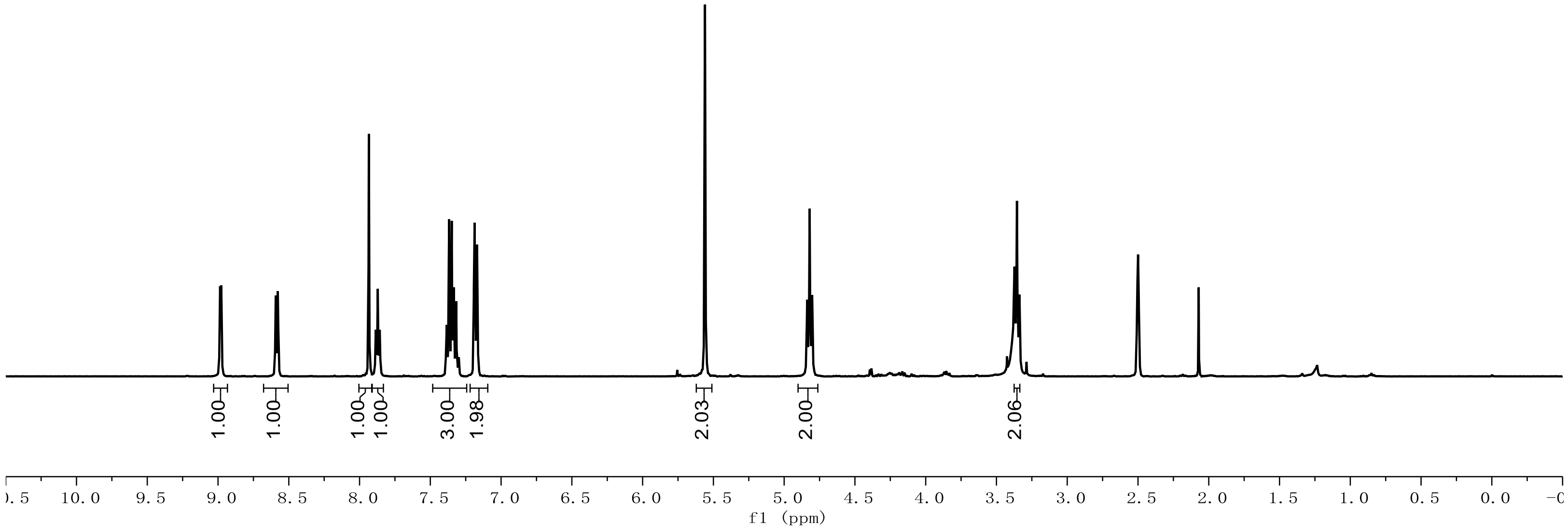

^{13}C NMR of 13a

DMSO-d_6, 101 MHz, 25 °C

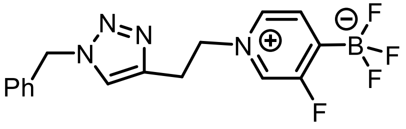

13a

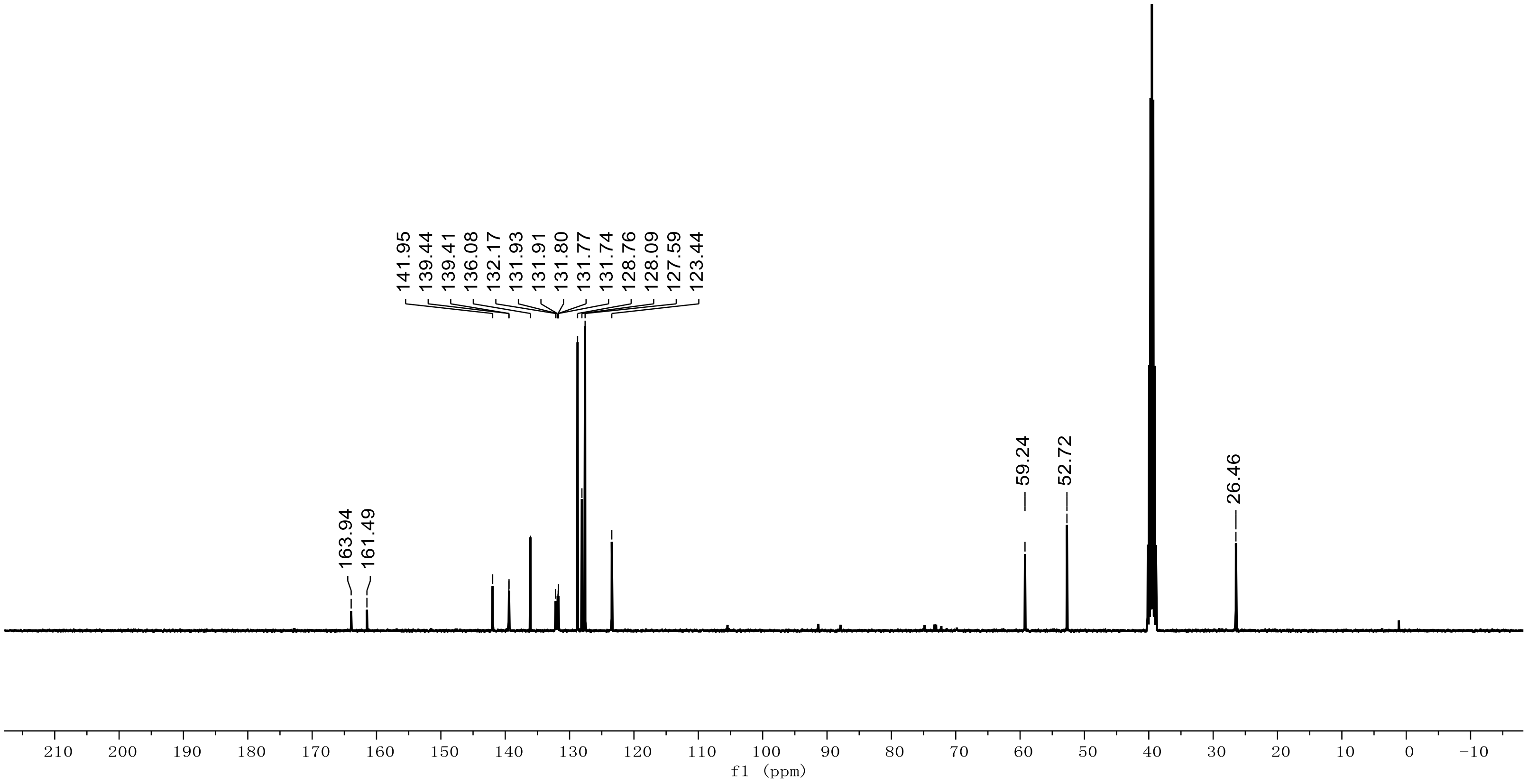

^{19}F NMR of 13a

DMSO-d_6, 376 MHz, 25 °C

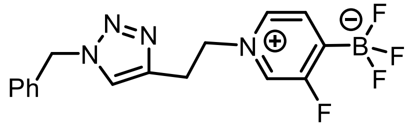

13a

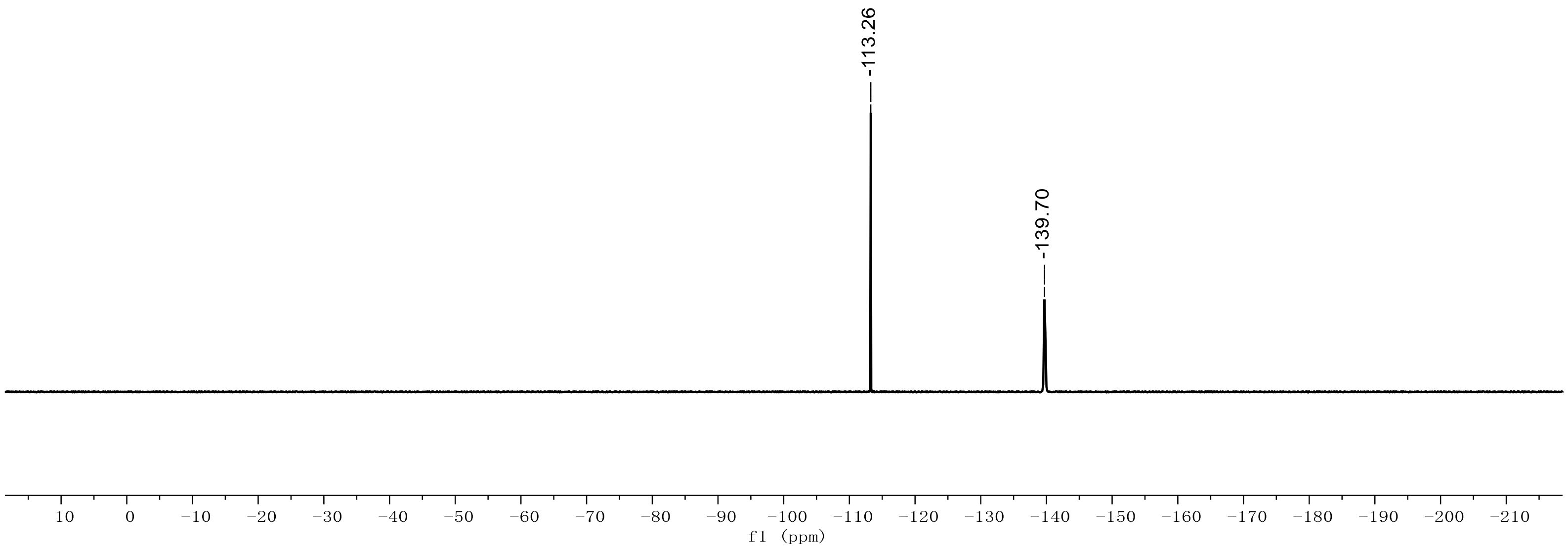

^{11}B NMR of 13a

DMSO-d_6, 128 MHz, 25 °C

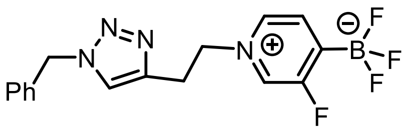

13a

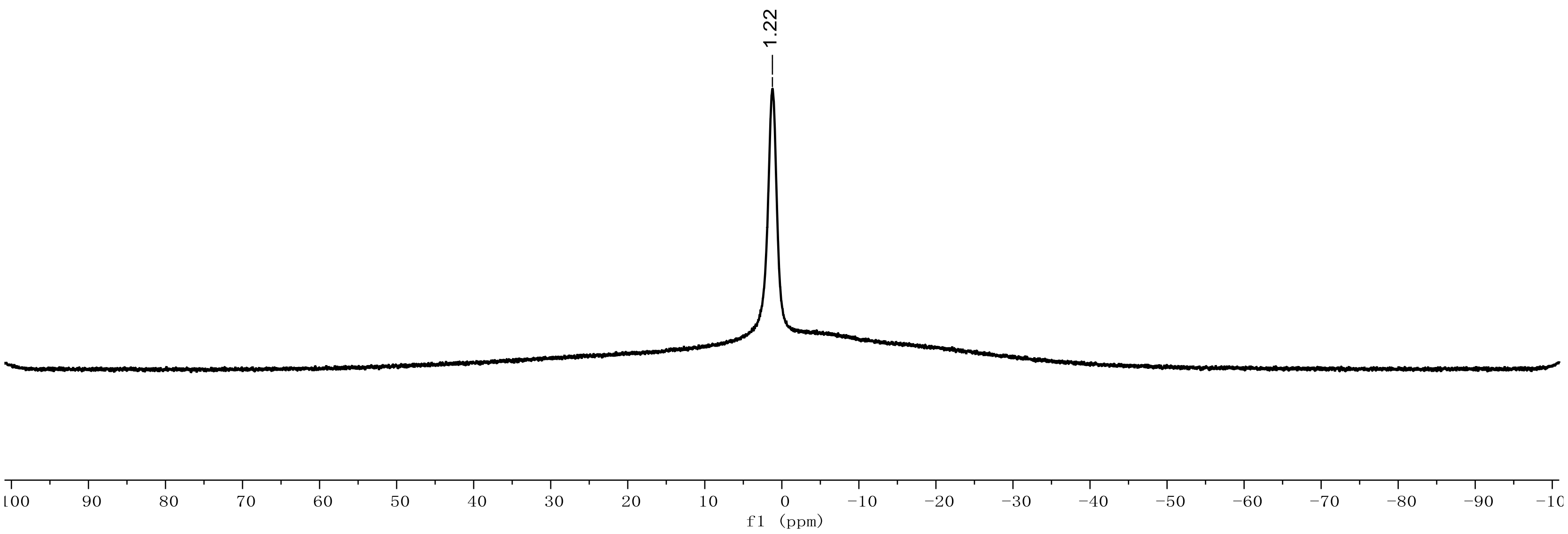

^{1}H NMR of 14a

CD_3CN, 400 MHz, 25 °C

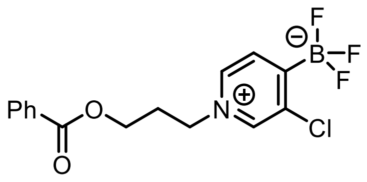

14a

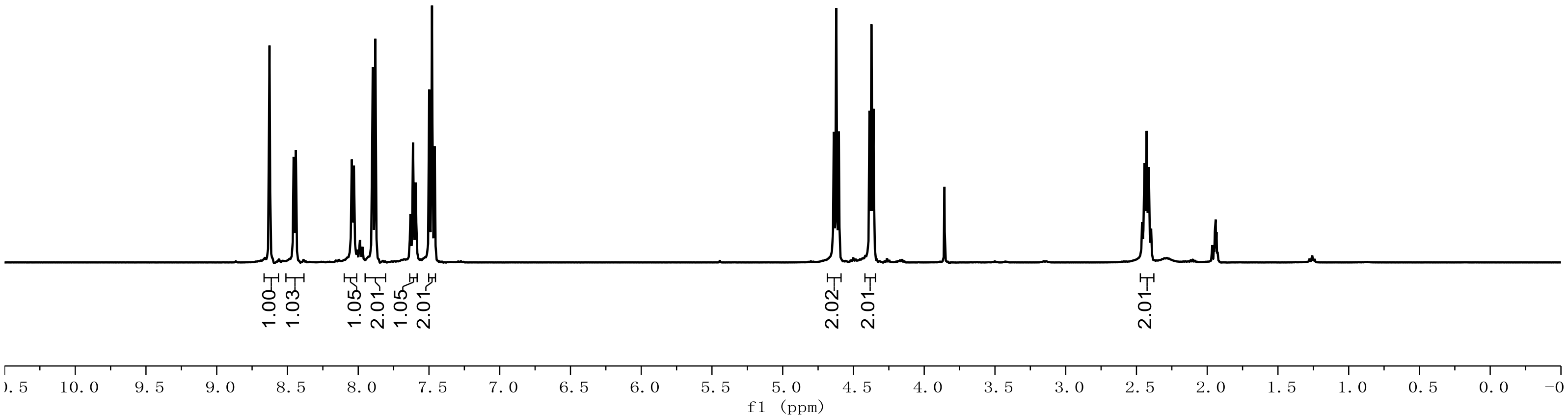

^{13}C NMR of 14a

CD_3CN, 101 MHz, 25 °C

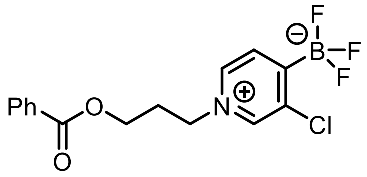

14a

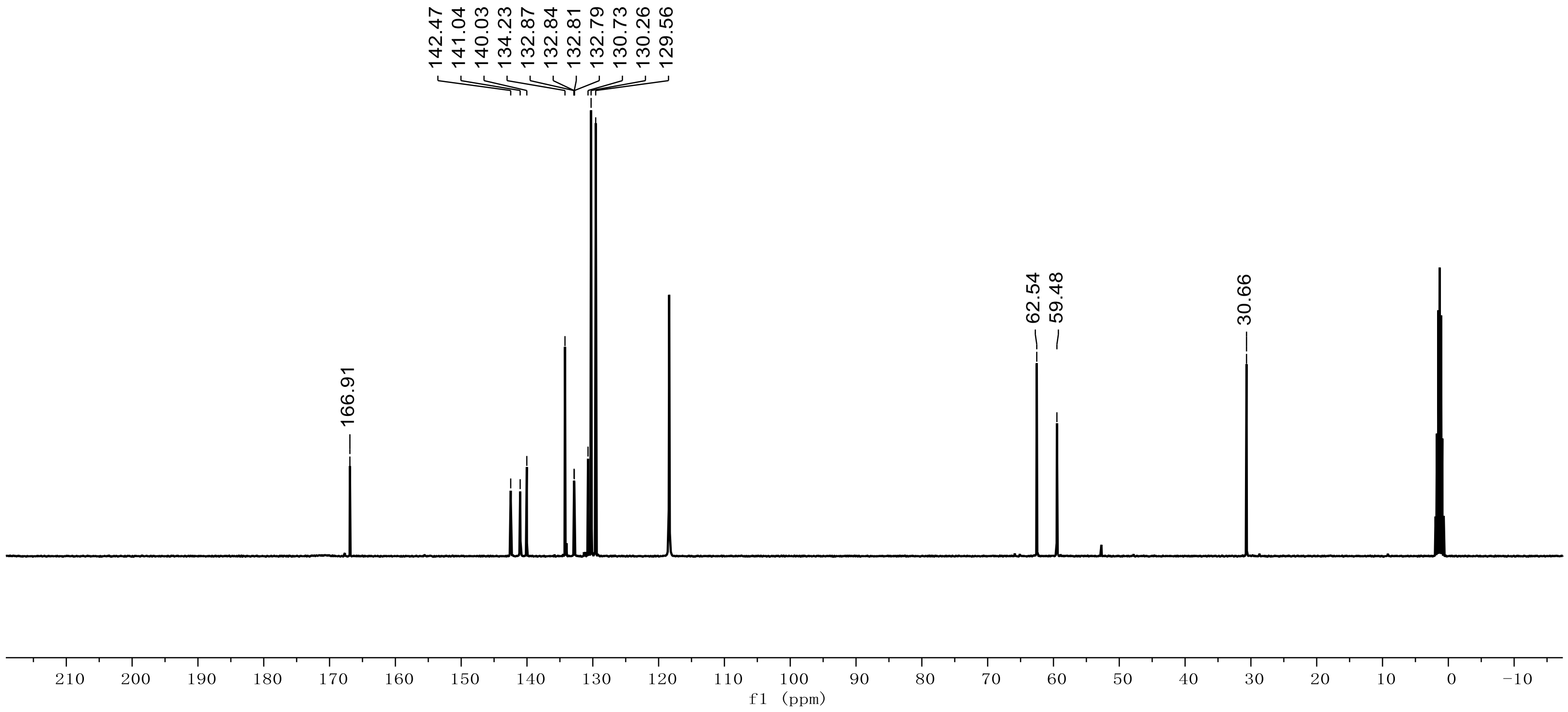

^{19}F NMR of 14a

CD_3CN, 376 MHz, 25 °C

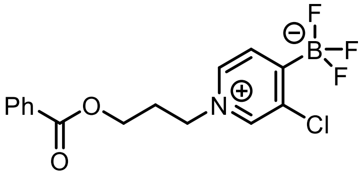

14a

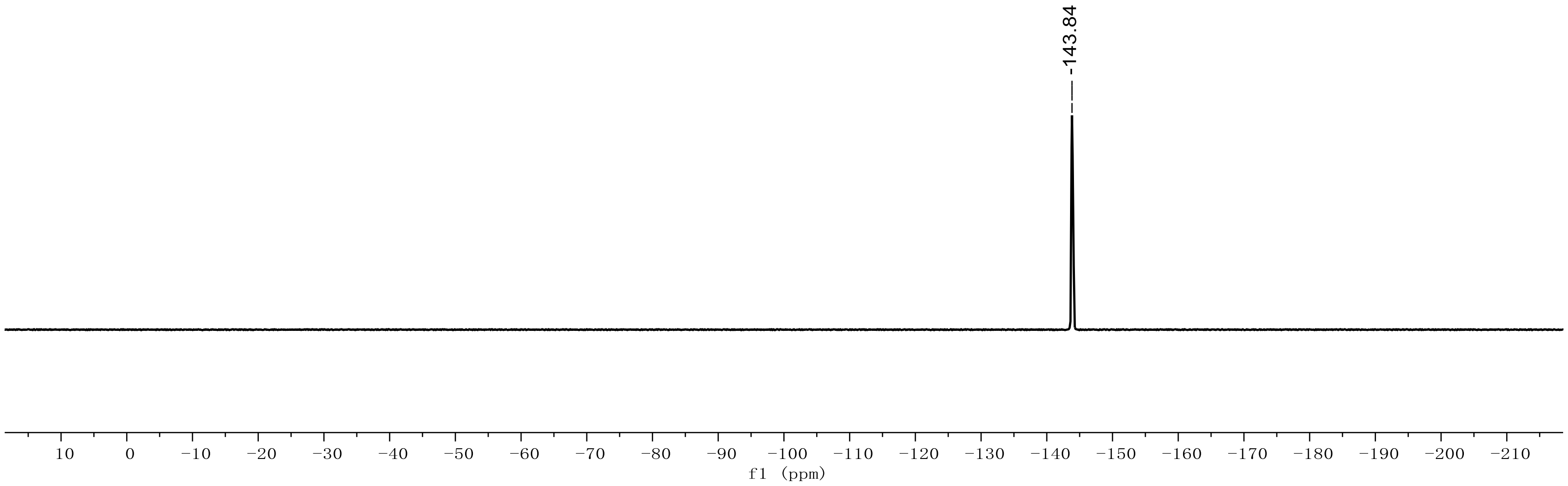

^{11}B NMR of 14a

CD_3CN, 128 MHz, 25 °C

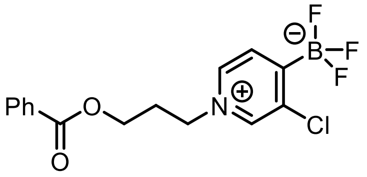

14a

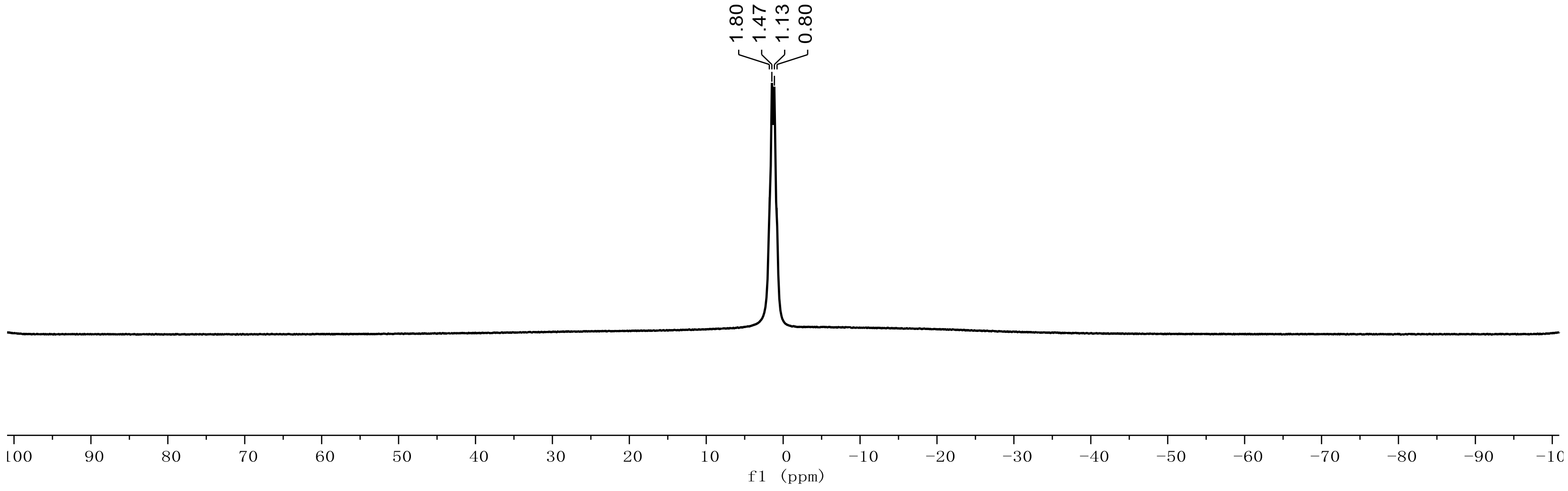

^{1}H NMR of 15a

DMSO-d_6, 400 MHz, 25 °C

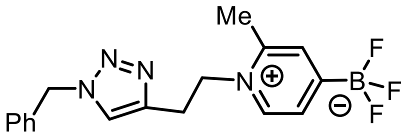

15a

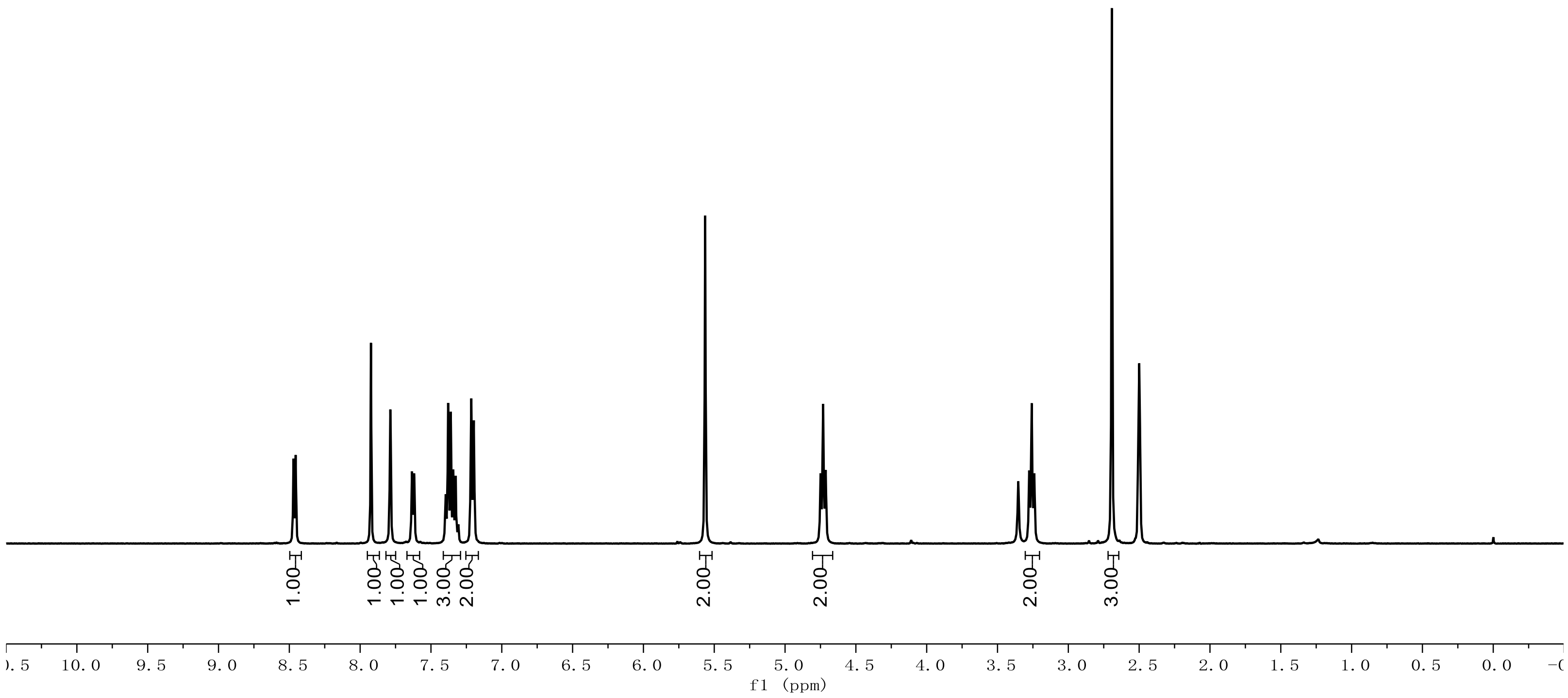

^{13}C NMR of 15a

DMSO-d_6, 101 MHz, 25 °C

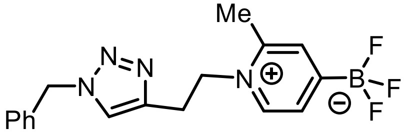

15a

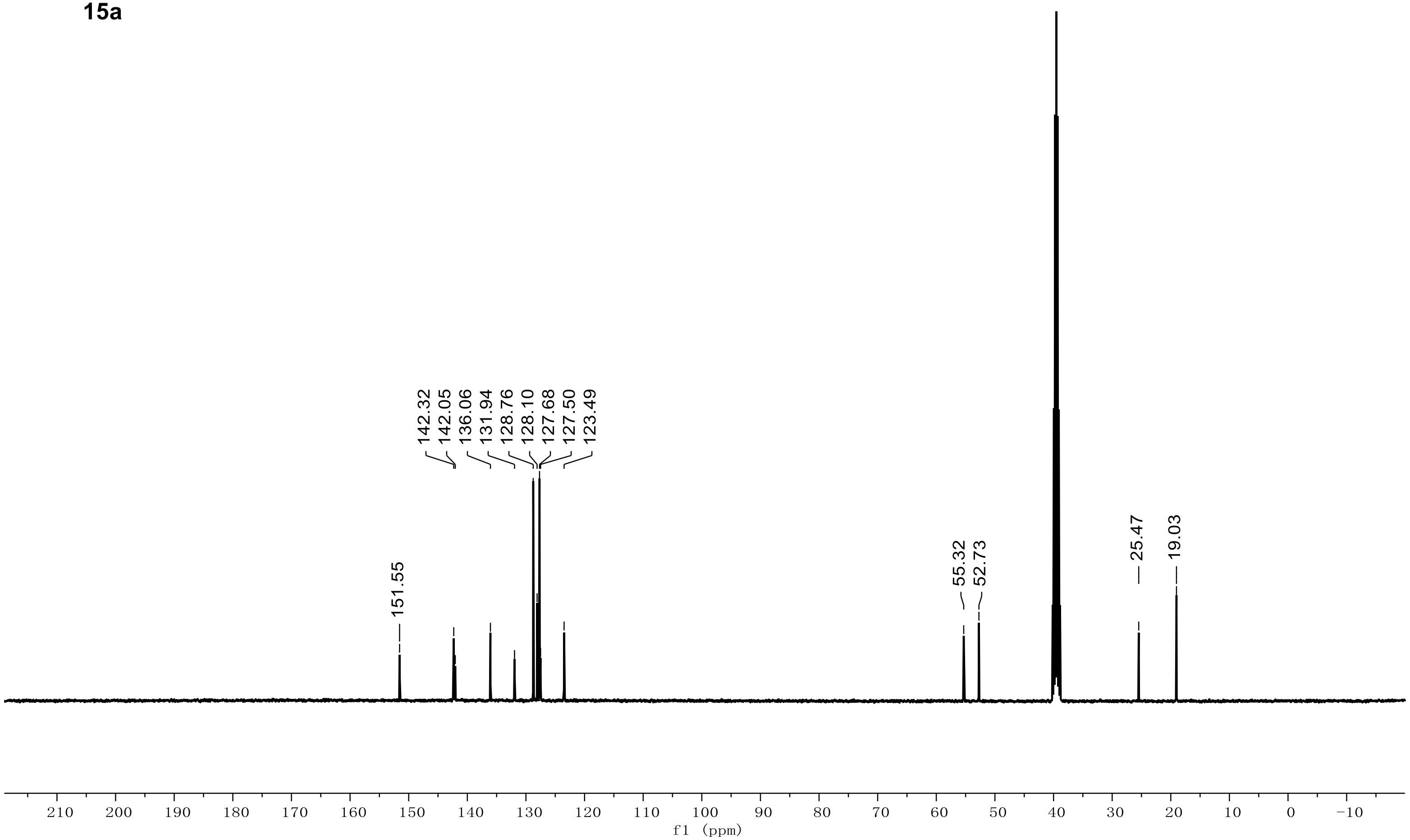

^{19}F NMR of 15a

DMSO-d_6, 376 MHz, 25 °C

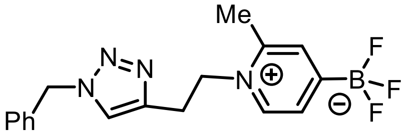

15a

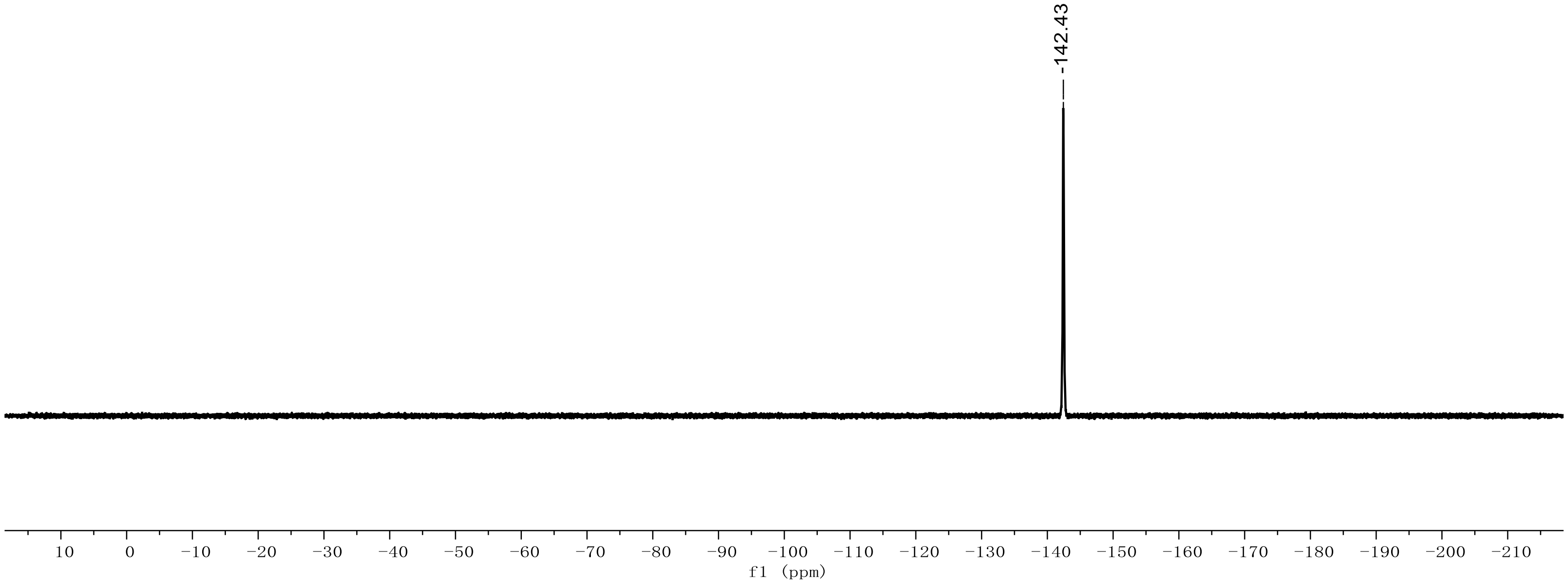

^{11}B NMR of 15a

DMSO-d_6, 128 MHz, 25 °C

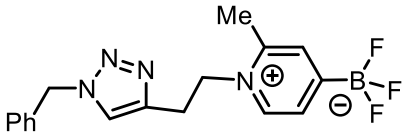

15a

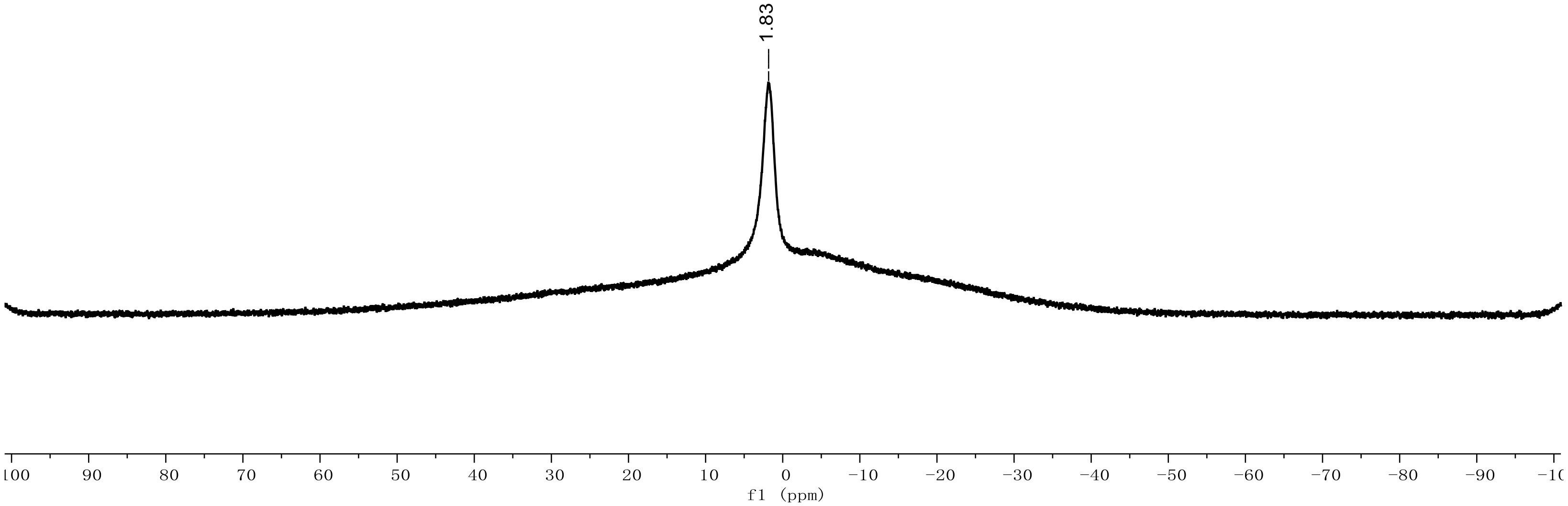

^{1}H NMR of 16a

CD_3CN, 400 MHz, 25 °C

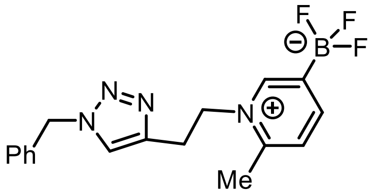

16a

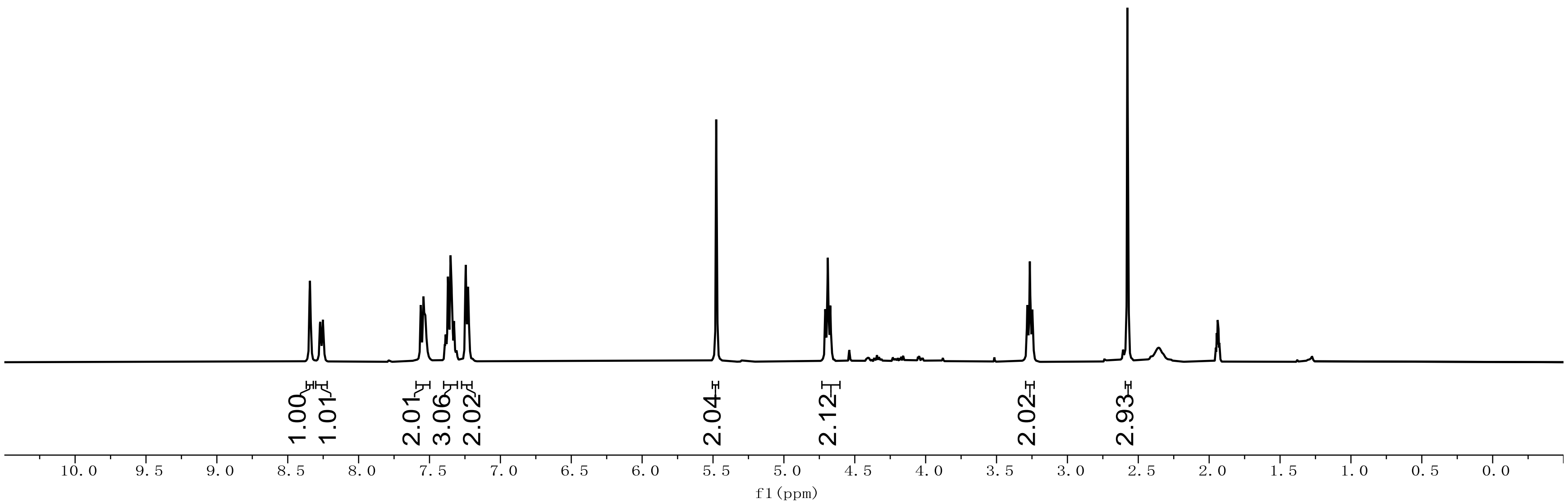

^{13}C NMR of 16a

DMSO-d_6, 101 MHz, 25 °C

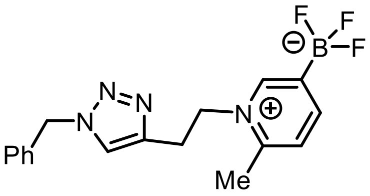

16a

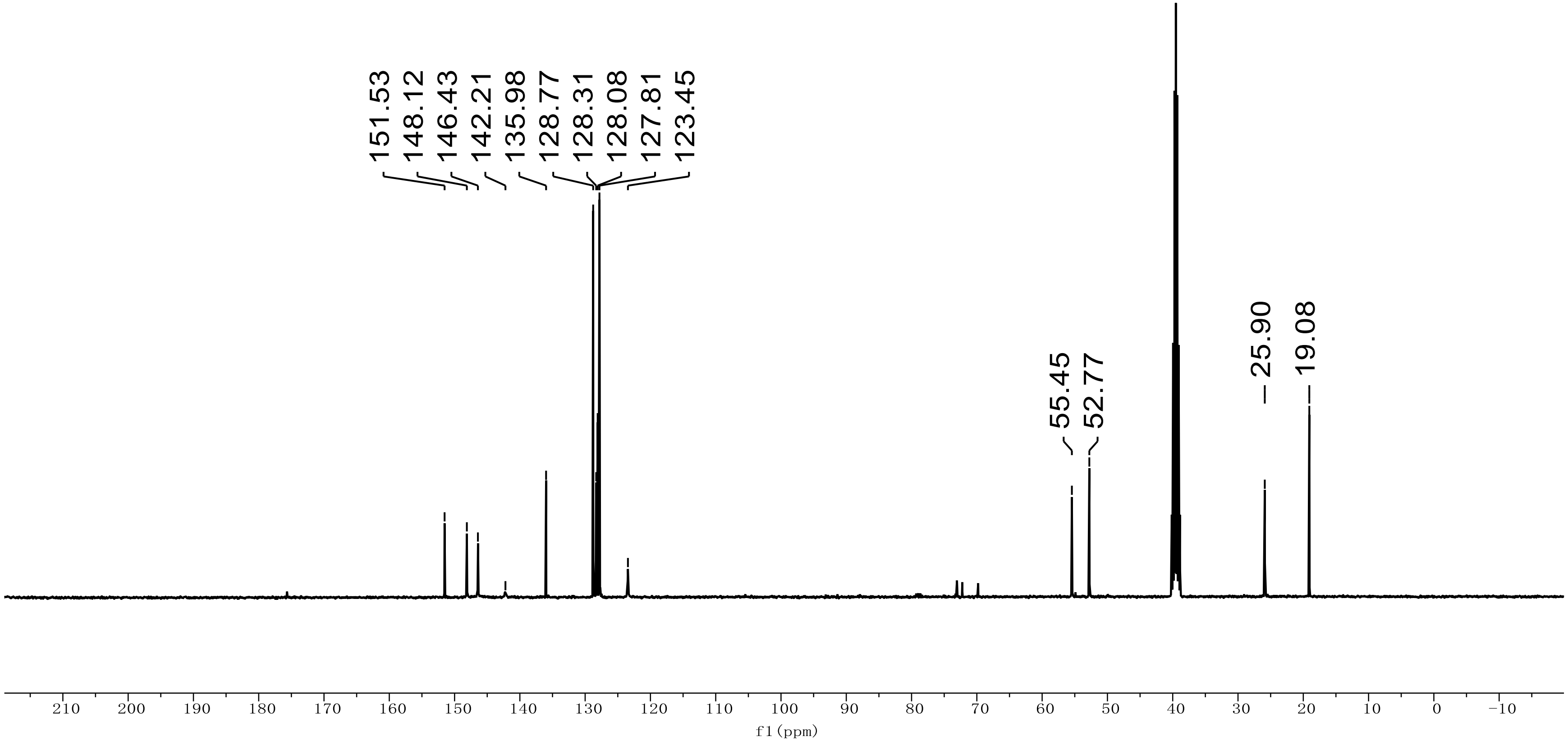

^{19}F NMR of 16a

CD_3CN, 376 MHz, 25 °C

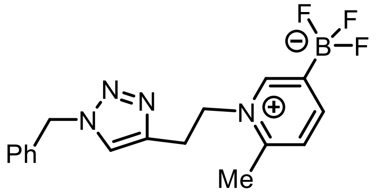

16a

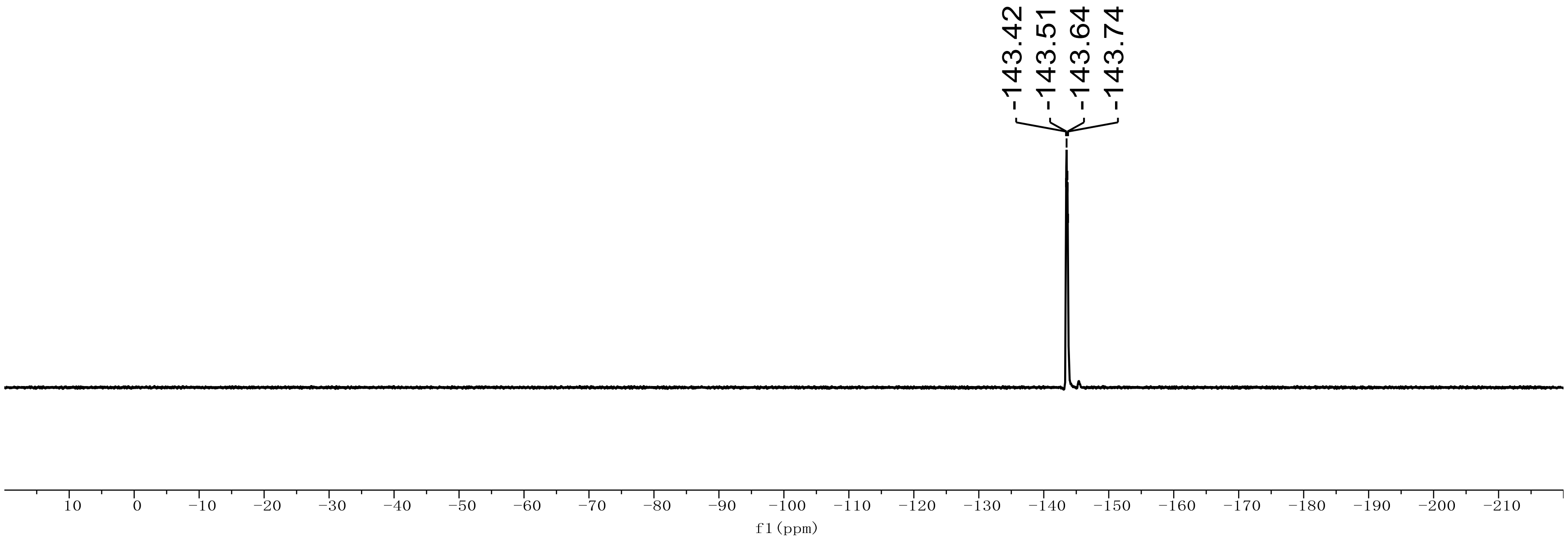

^{11}B NMR of 16a

CD_3CN, 128 MHz, 25 °C

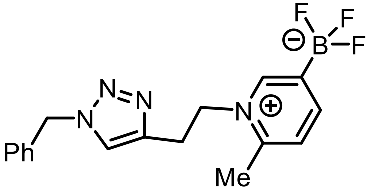

16a

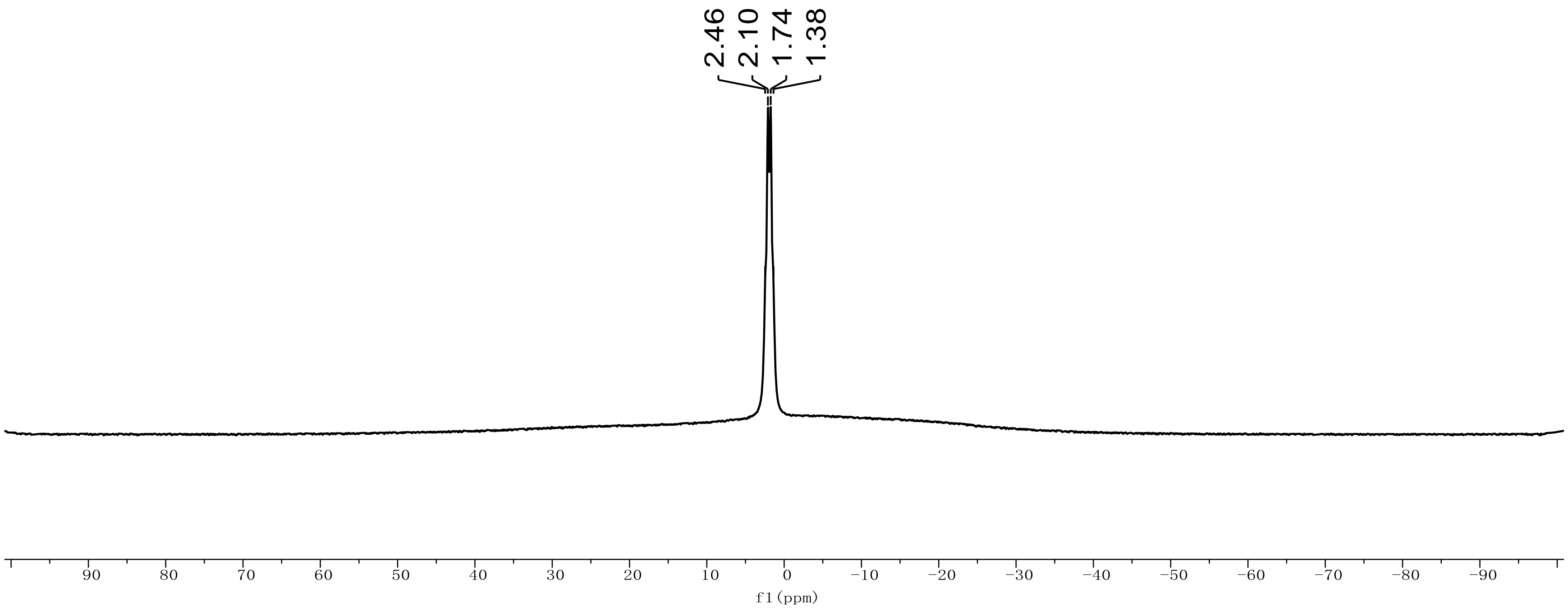

^{1}H NMR of 3b

CDCl$_3$, 400 MHz, 25 °C

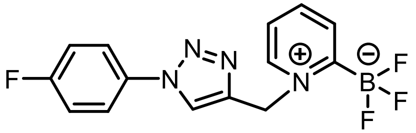

3b

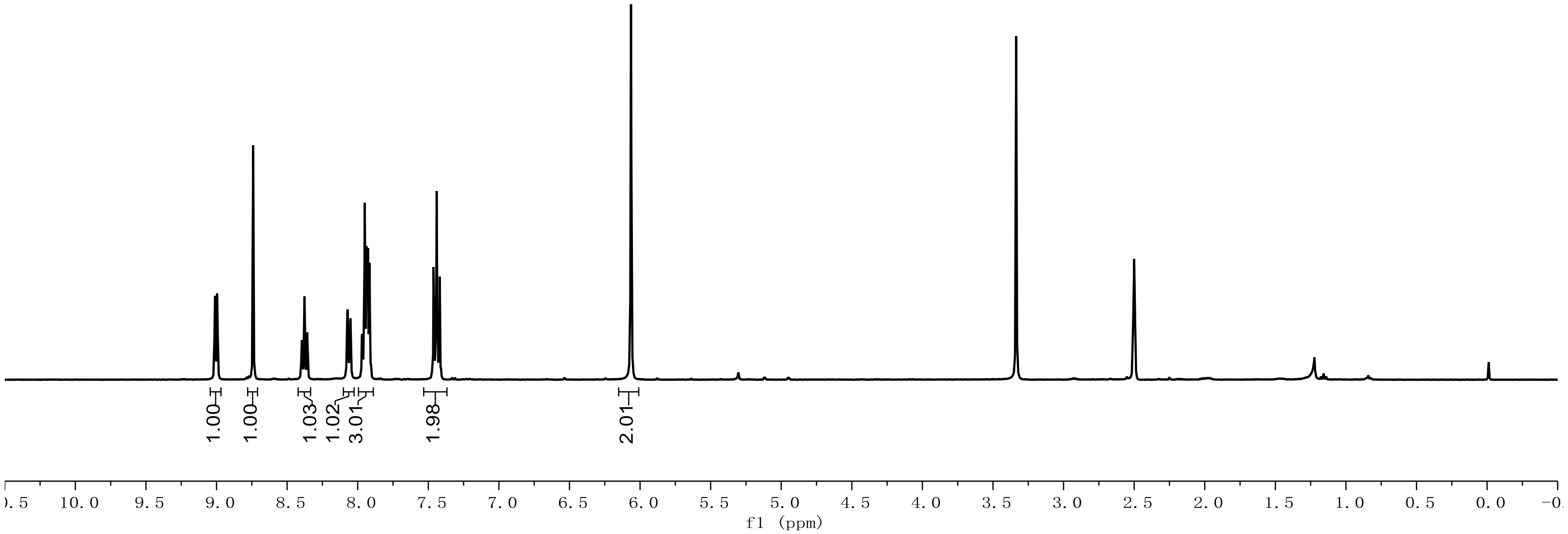

^{13}C NMR of 3b

DMSO-d_6, 101 MHz, 25 °C

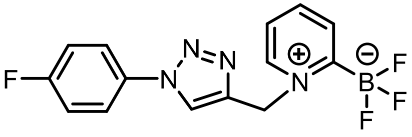

3b

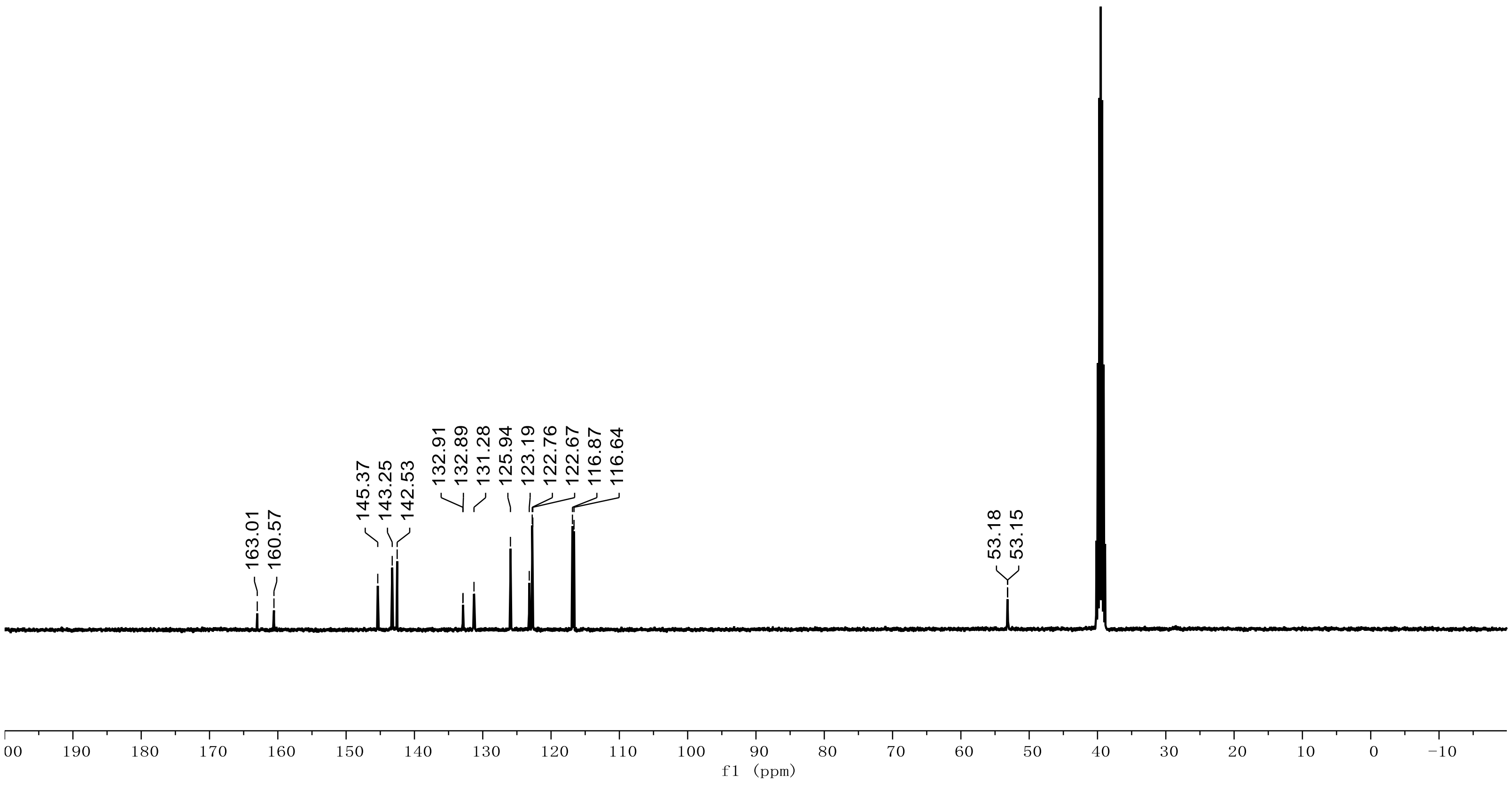

^{19}F NMR of 3b

CDCl$_3$, 376 MHz, 25 °C

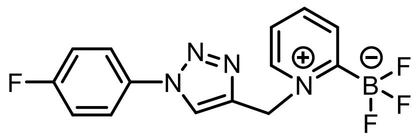

3b

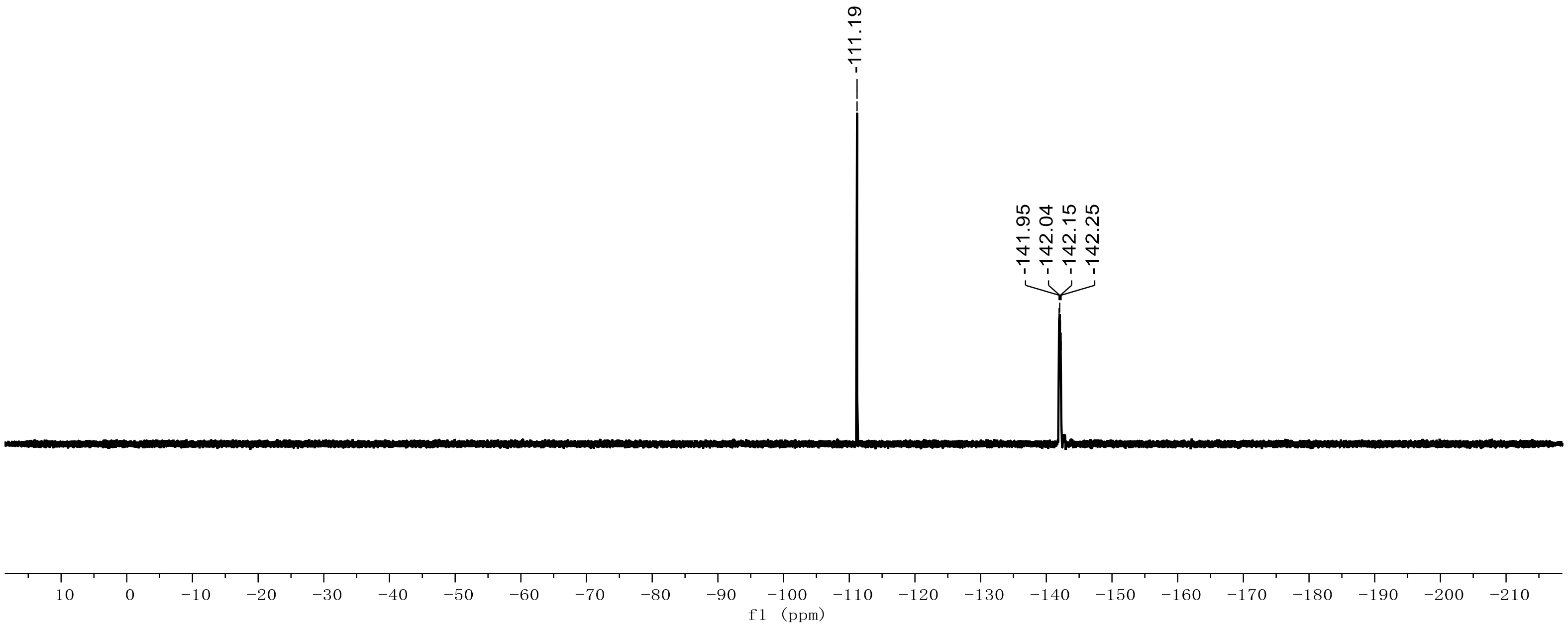

^{11}B NMR of 3b

$CDCl_3$, 128 MHz, 25 °C

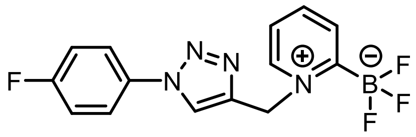

3b

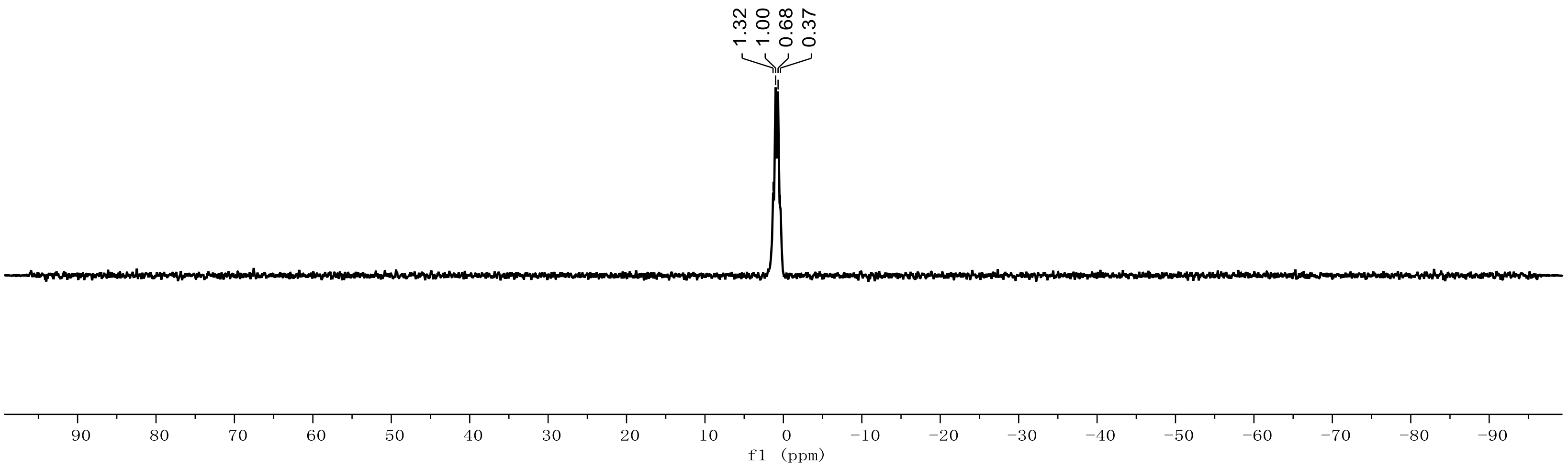

^{1}H NMR of 5c

DMSO-d_6, 400 MHz, 25 °C

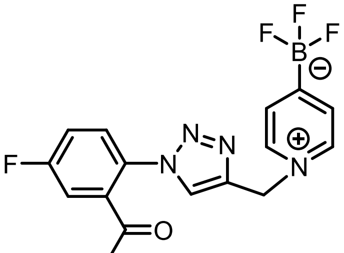

5c

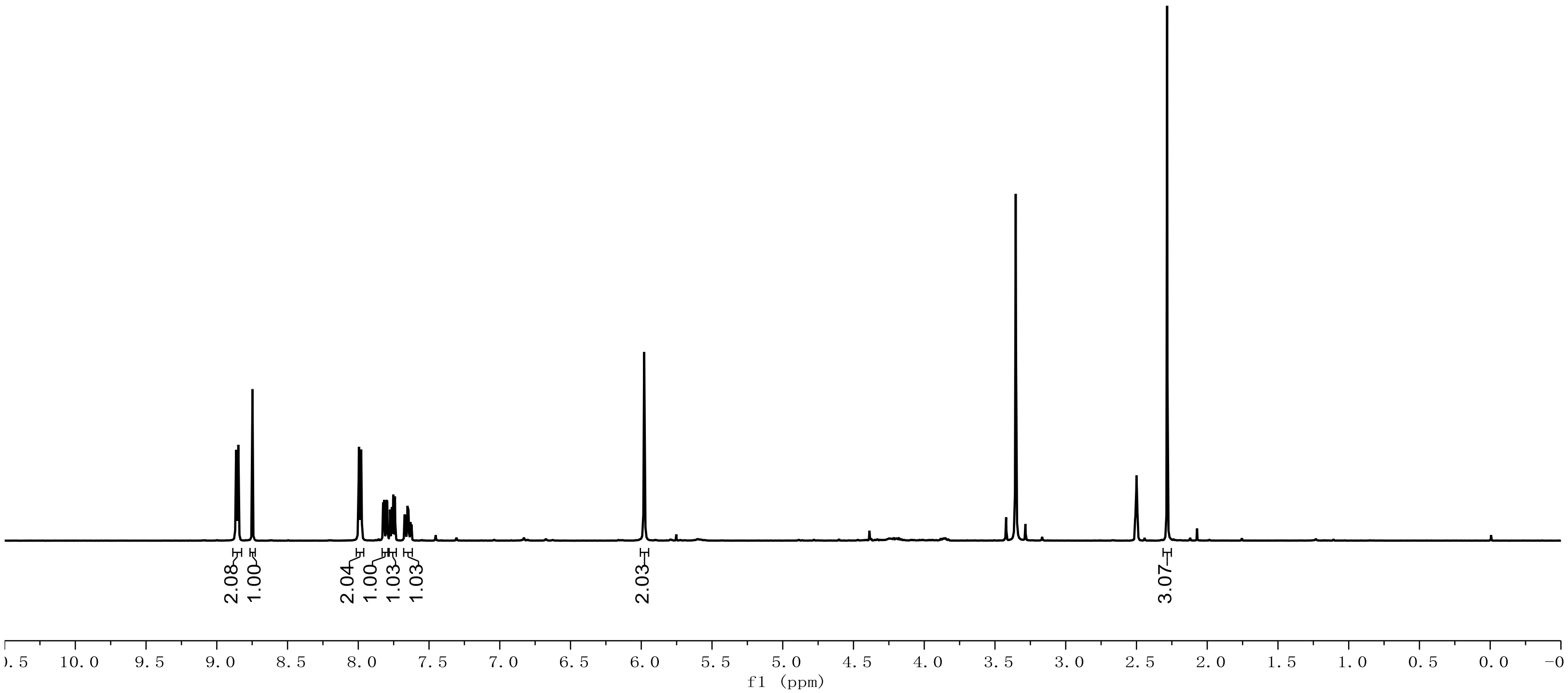

^{13}C NMR of 5c

DMSO-d_6, 101 MHz, 25 °C

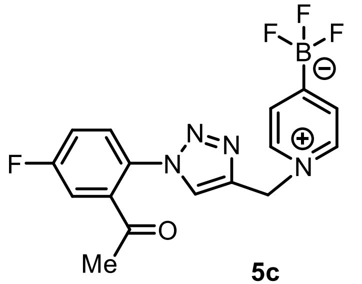

5c

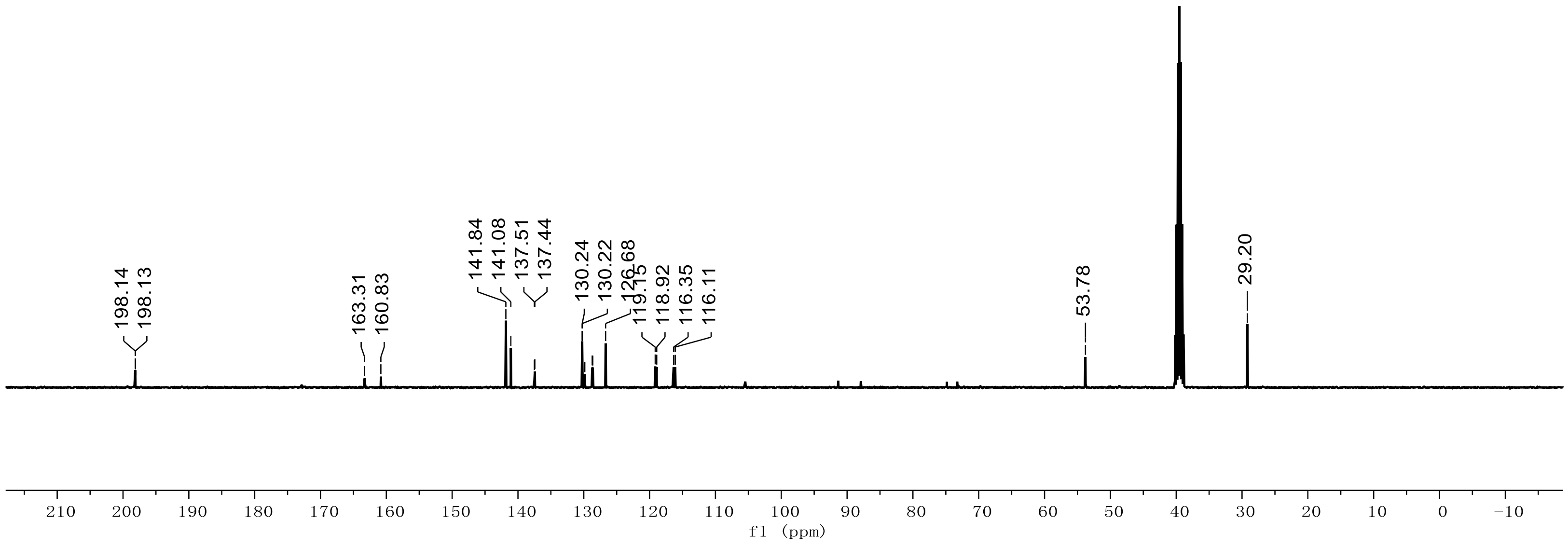

^{19}F NMR of 5c

DMSO-d_6, 376 MHz, 25 °C

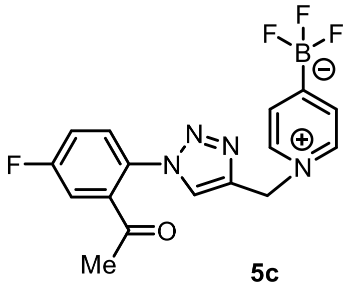

5c

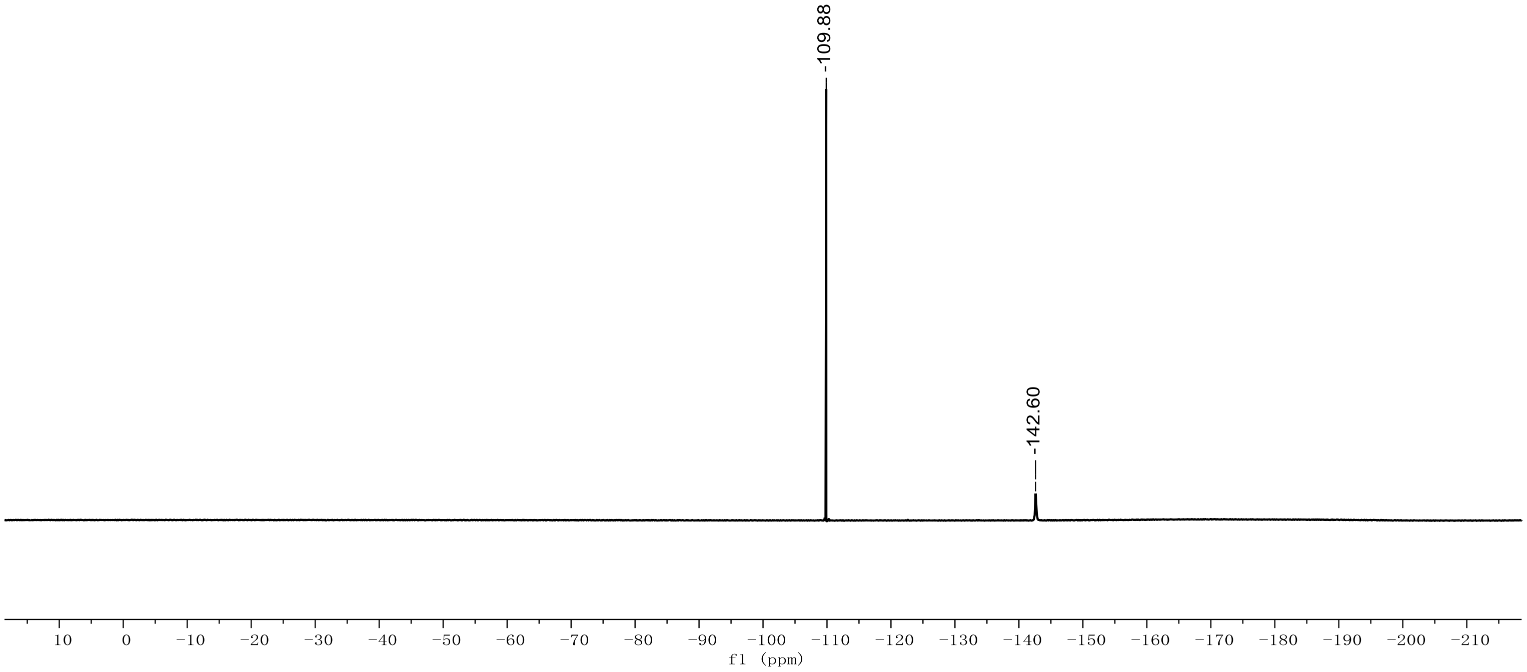

^{11}B NMR of 5c

DMSO-d_6, 128 MHz, 25 °C

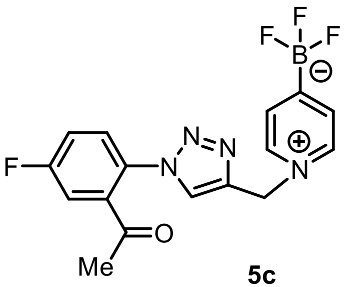

5c

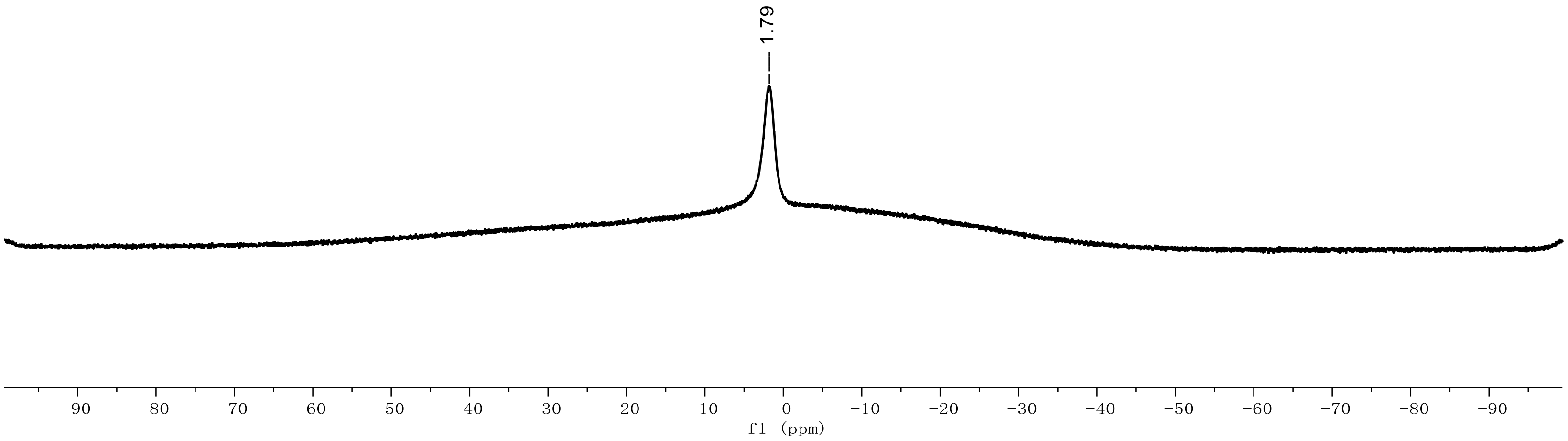

¹H NMR of 3d

CD₃CN, 400 MHz, 25 °C

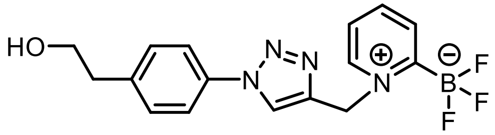

3d

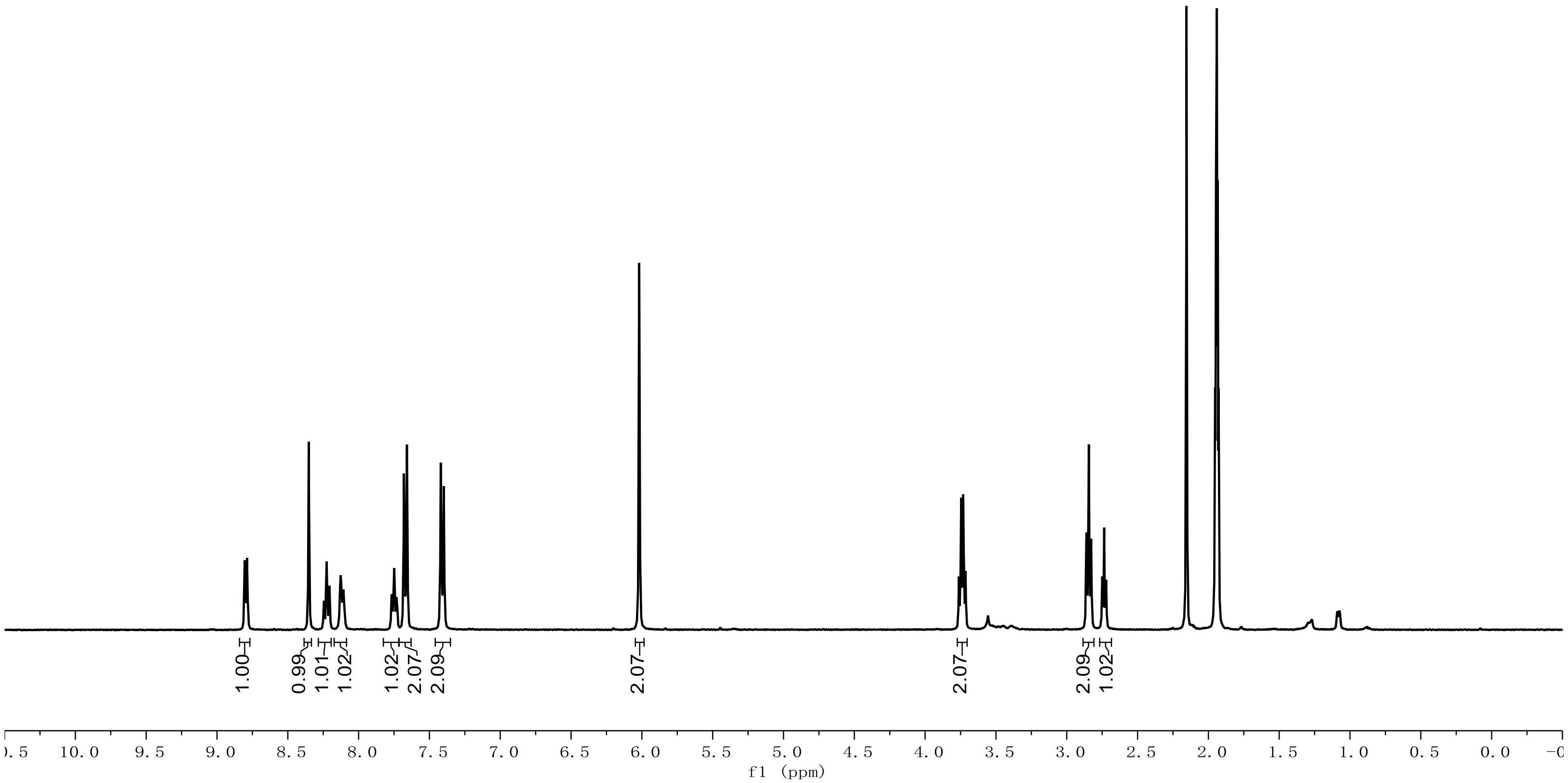

^{13}C NMR of 3d

CD_3CN, 101 MHz, 25 °C

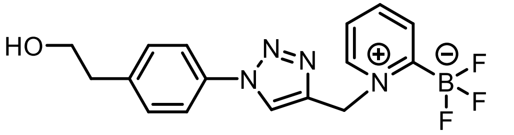

3d

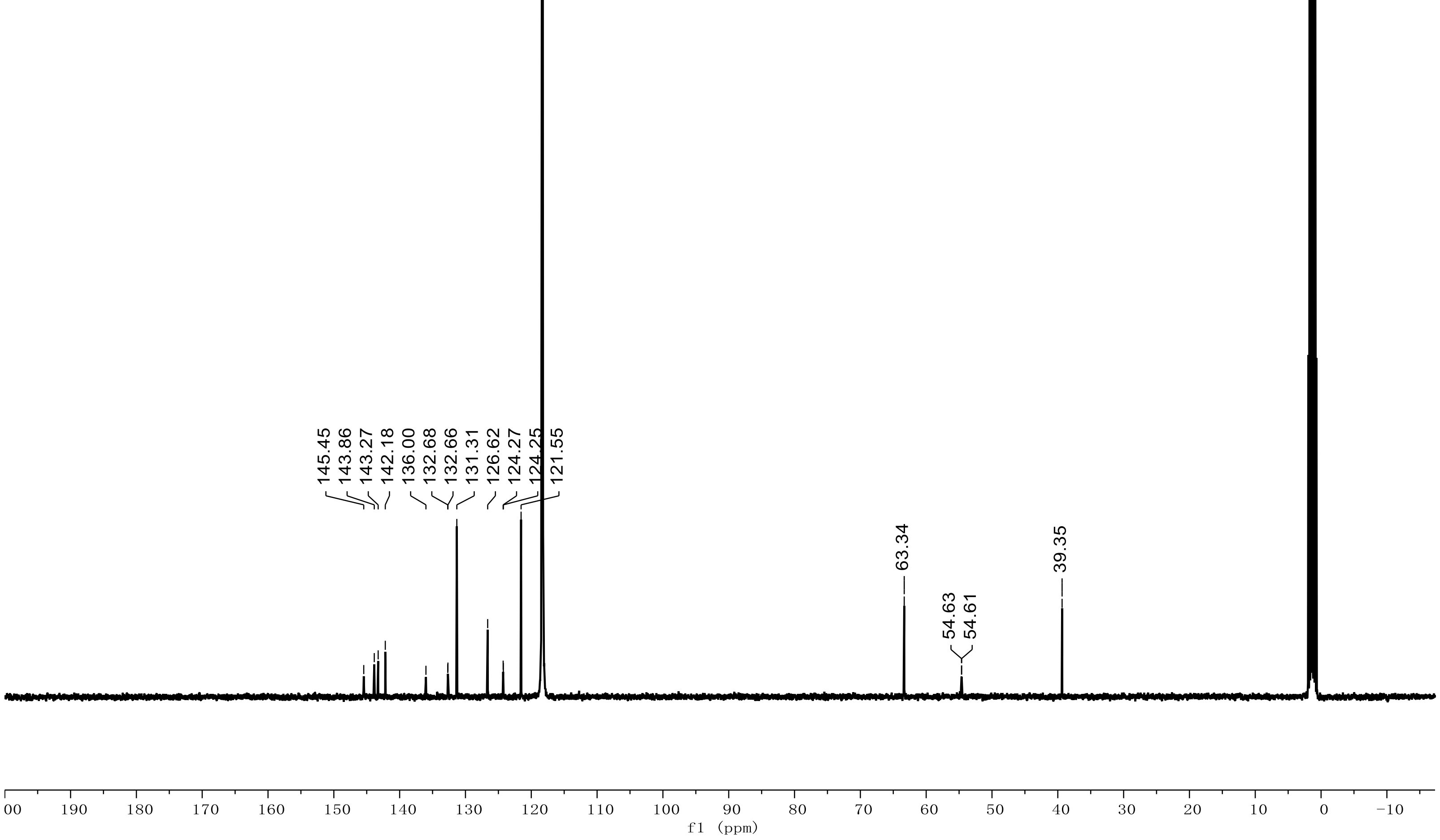

^{19}F NMR of 3d

CD_3CN, 376 MHz, 25 °C

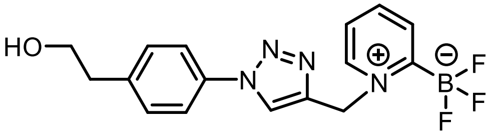

3d

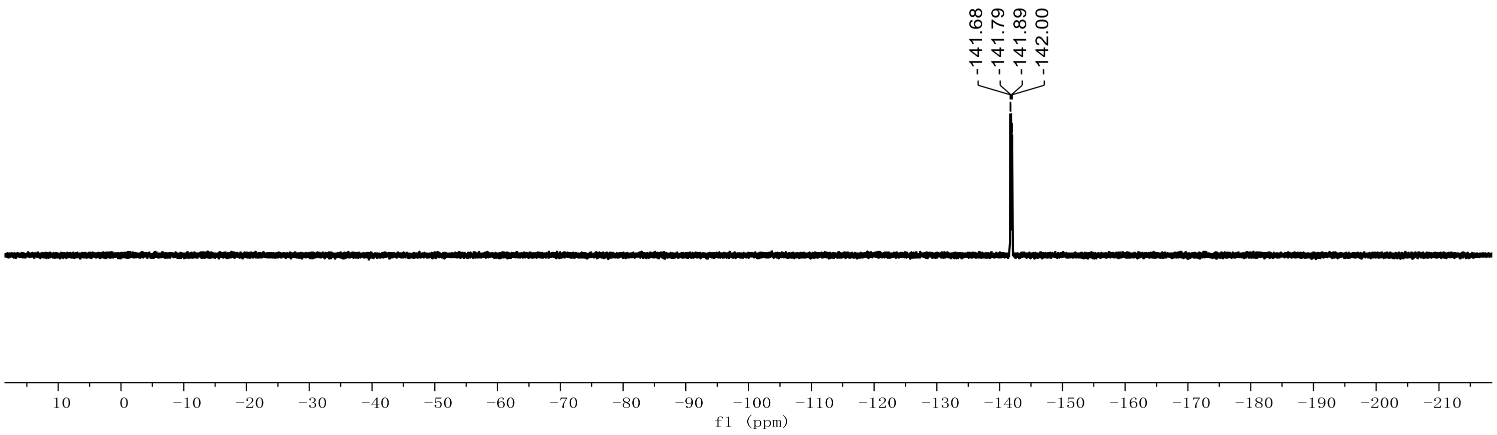

^{11}B NMR of 3d

CD_3CN, 128 MHz, 25 °C

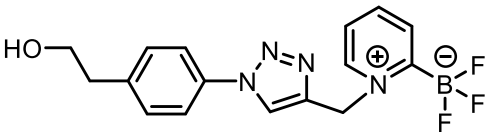

3d

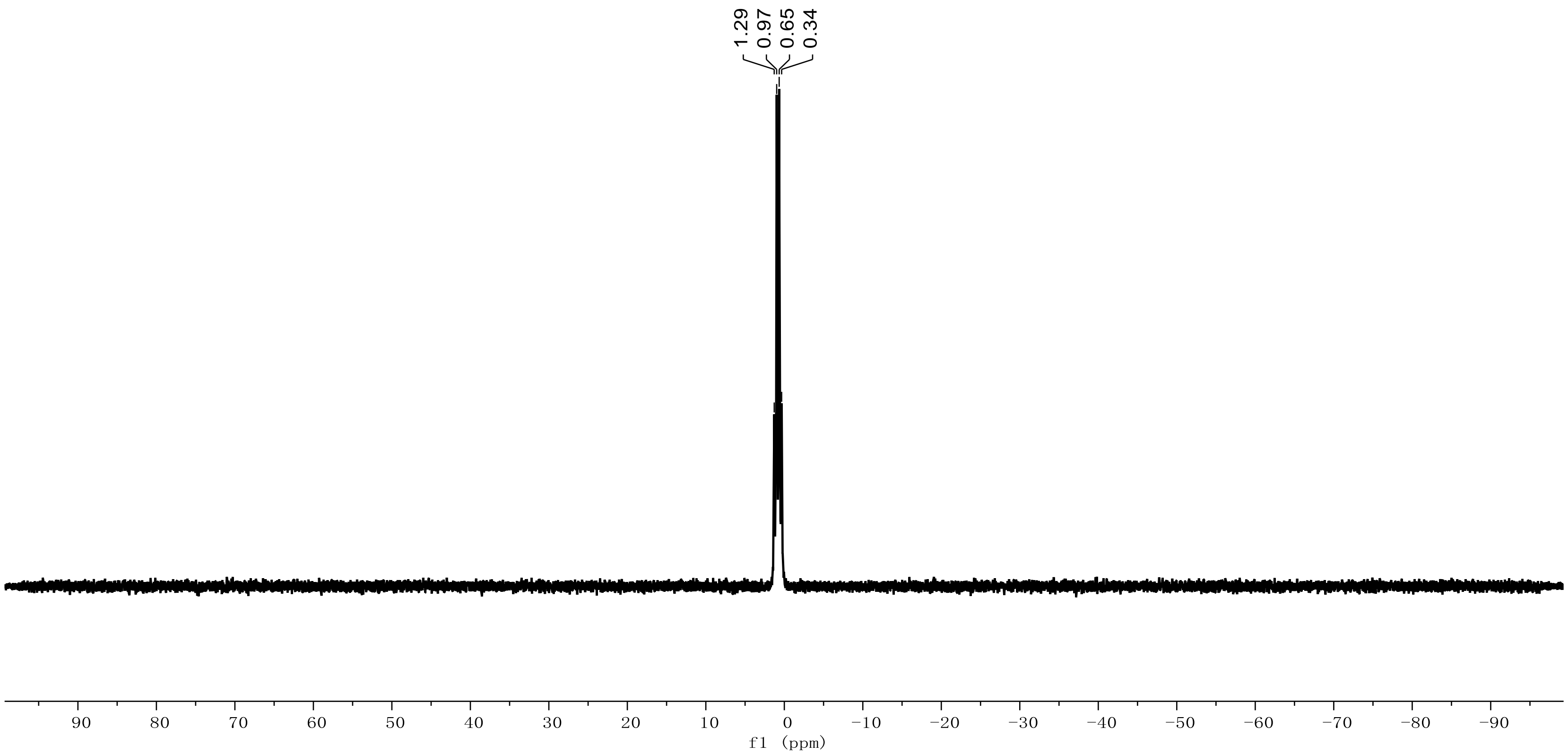

^{1}H NMR of 4e

DMSO-d_6, 400 MHz, 25 °C

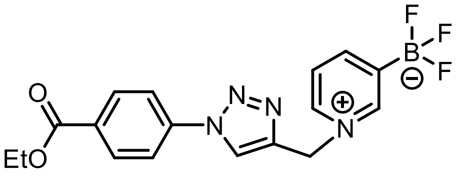

4e

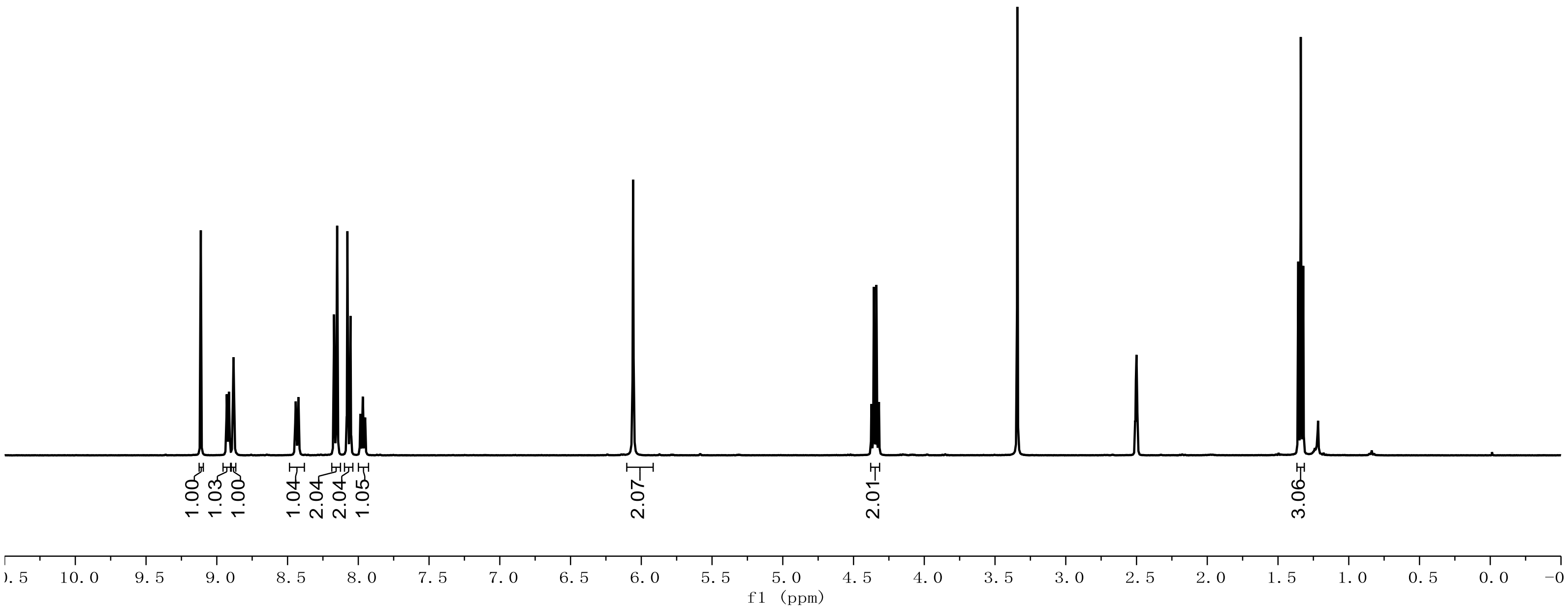

^{13}C NMR of 4e

DMSO-d_6, 101 MHz, 25 °C

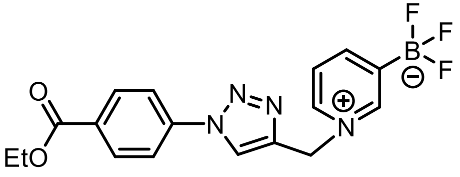

4e

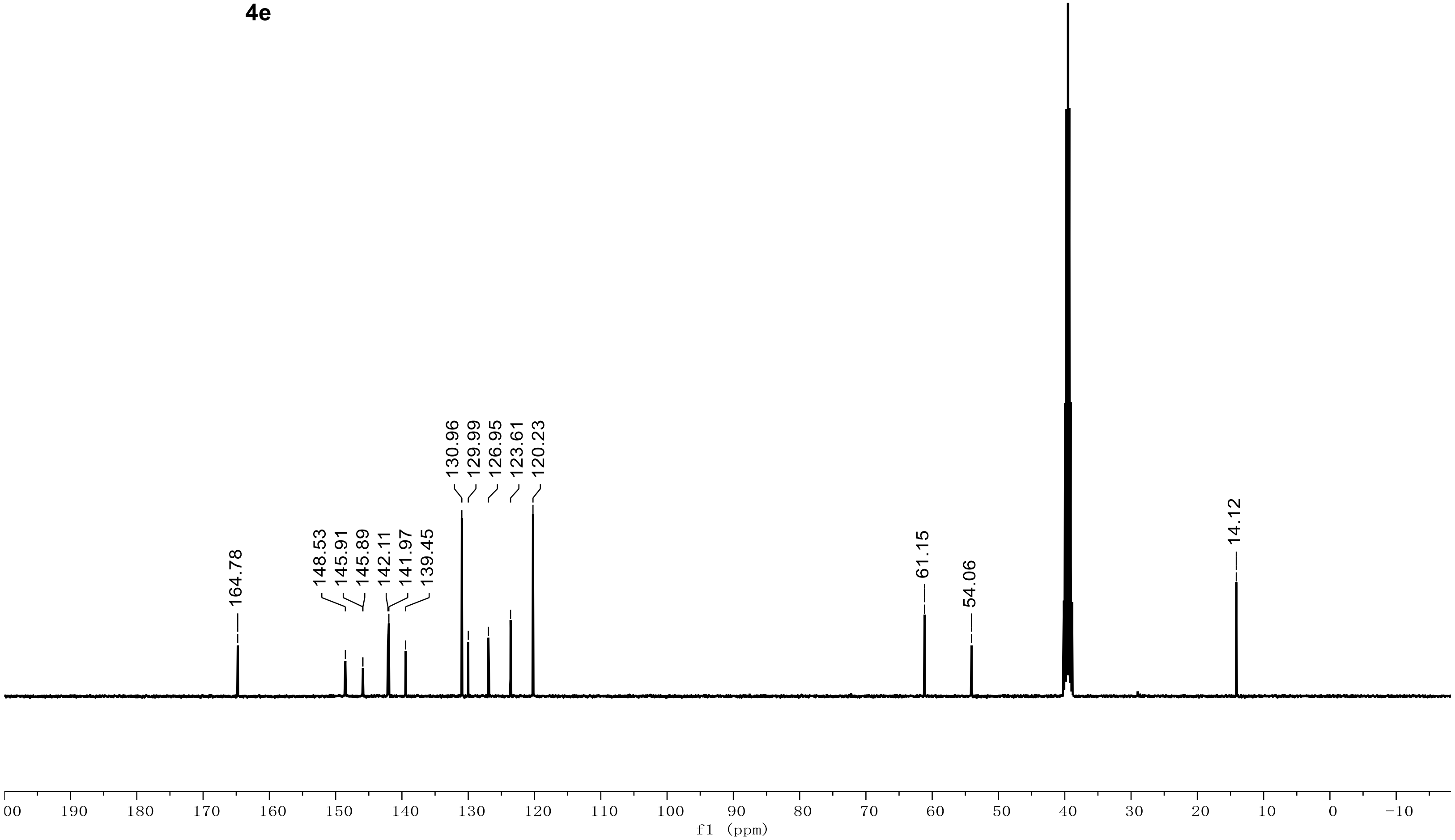

^{19}F NMR of 4e

DMSO-d_6, 376 MHz, 25 °C

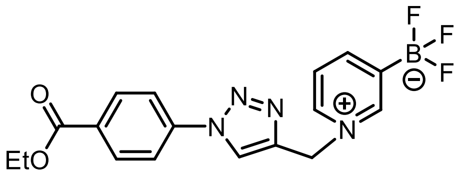

4e

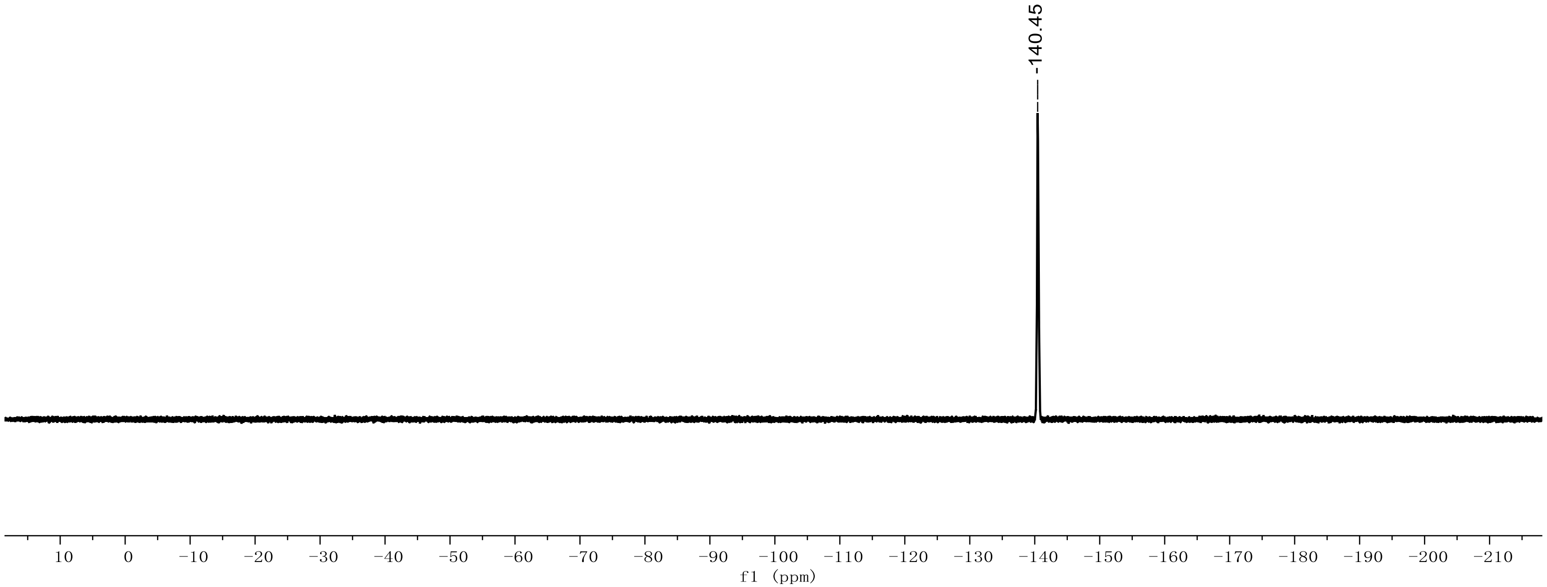

^{11}B NMR of 4e

DMSO-d_6, 128 MHz, 25 °C

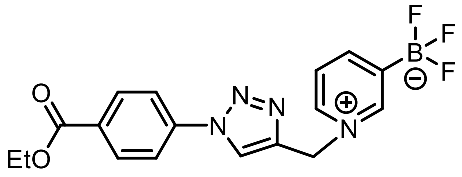

4e

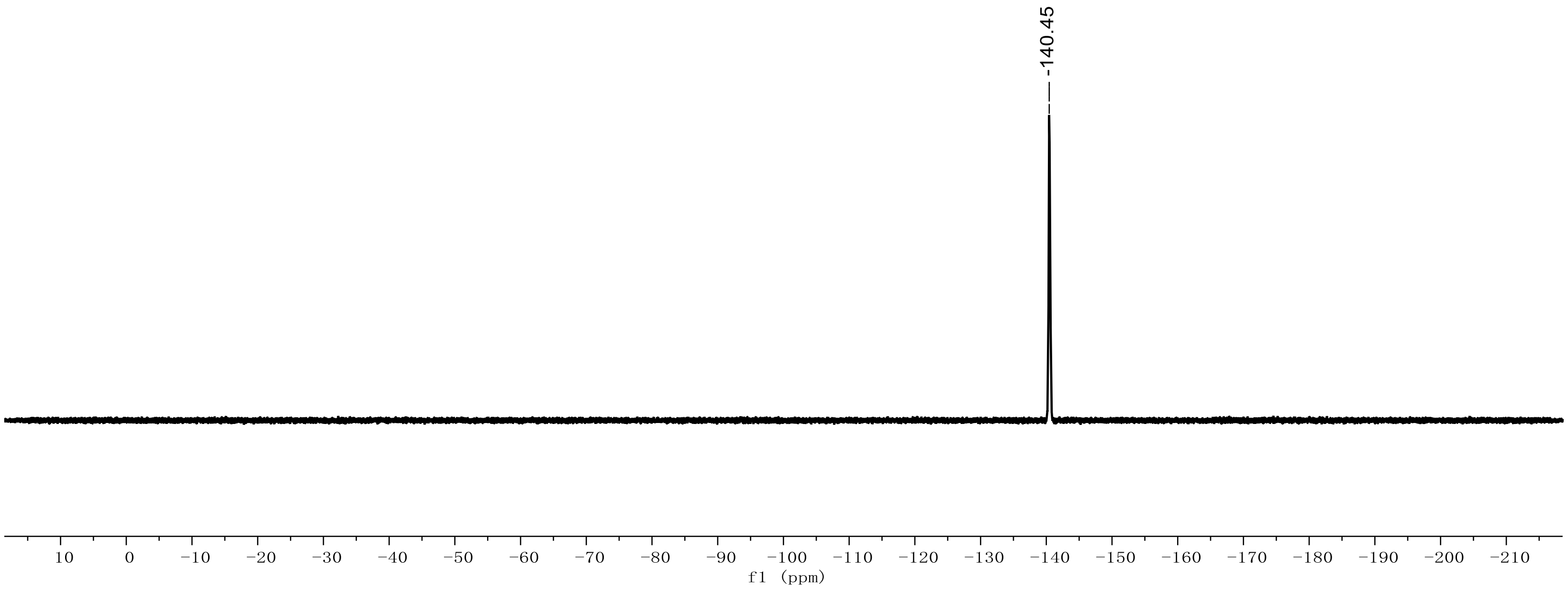

^{1}H NMR of 3f

DMSO-d_6, 400 MHz, 25 °C

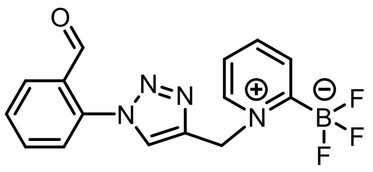

3f

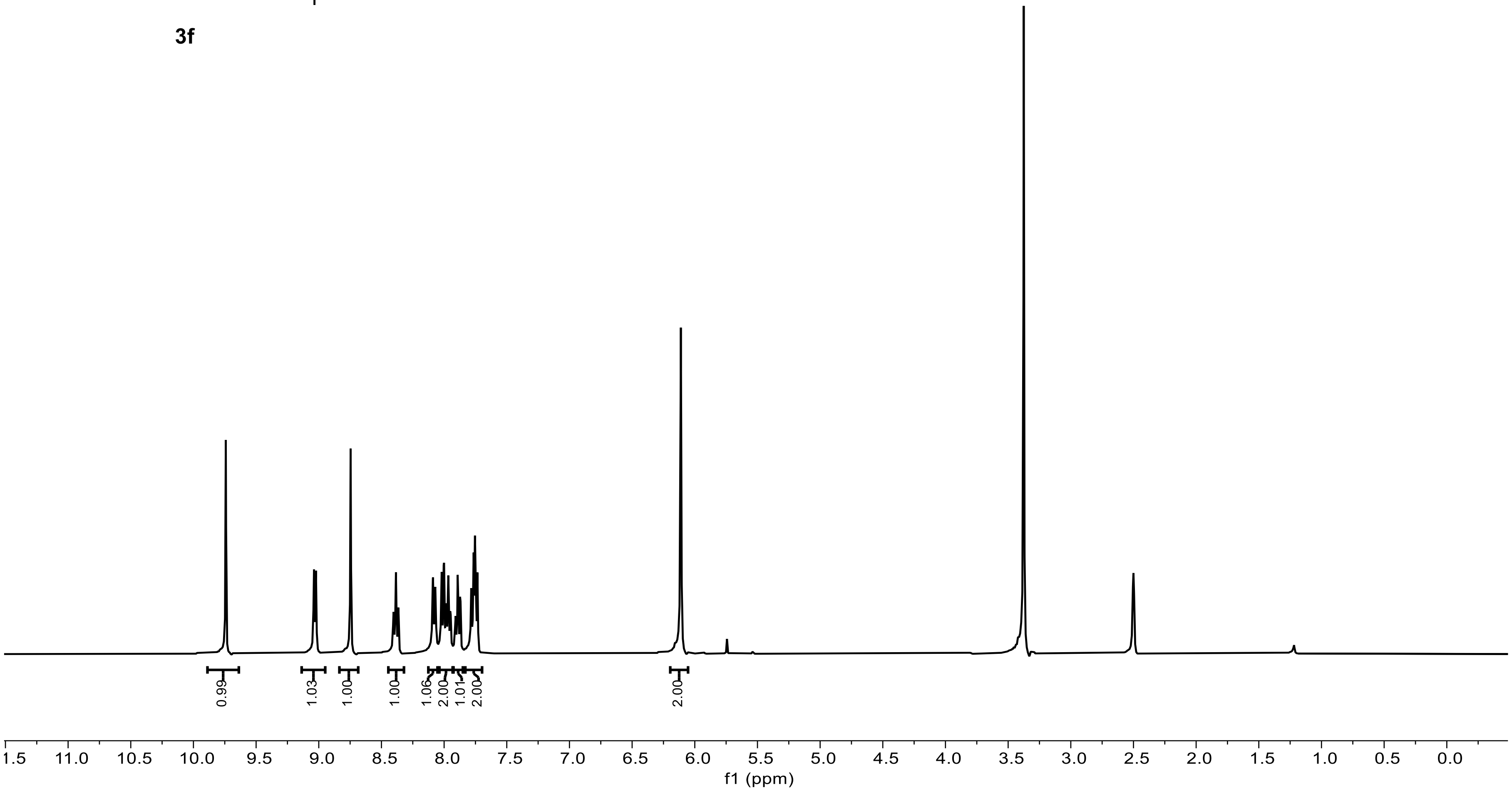

^{13}C NMR of 3f

DMSO-d_6, 101 MHz, 25 °C

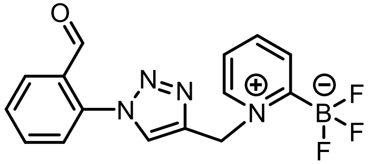

3f

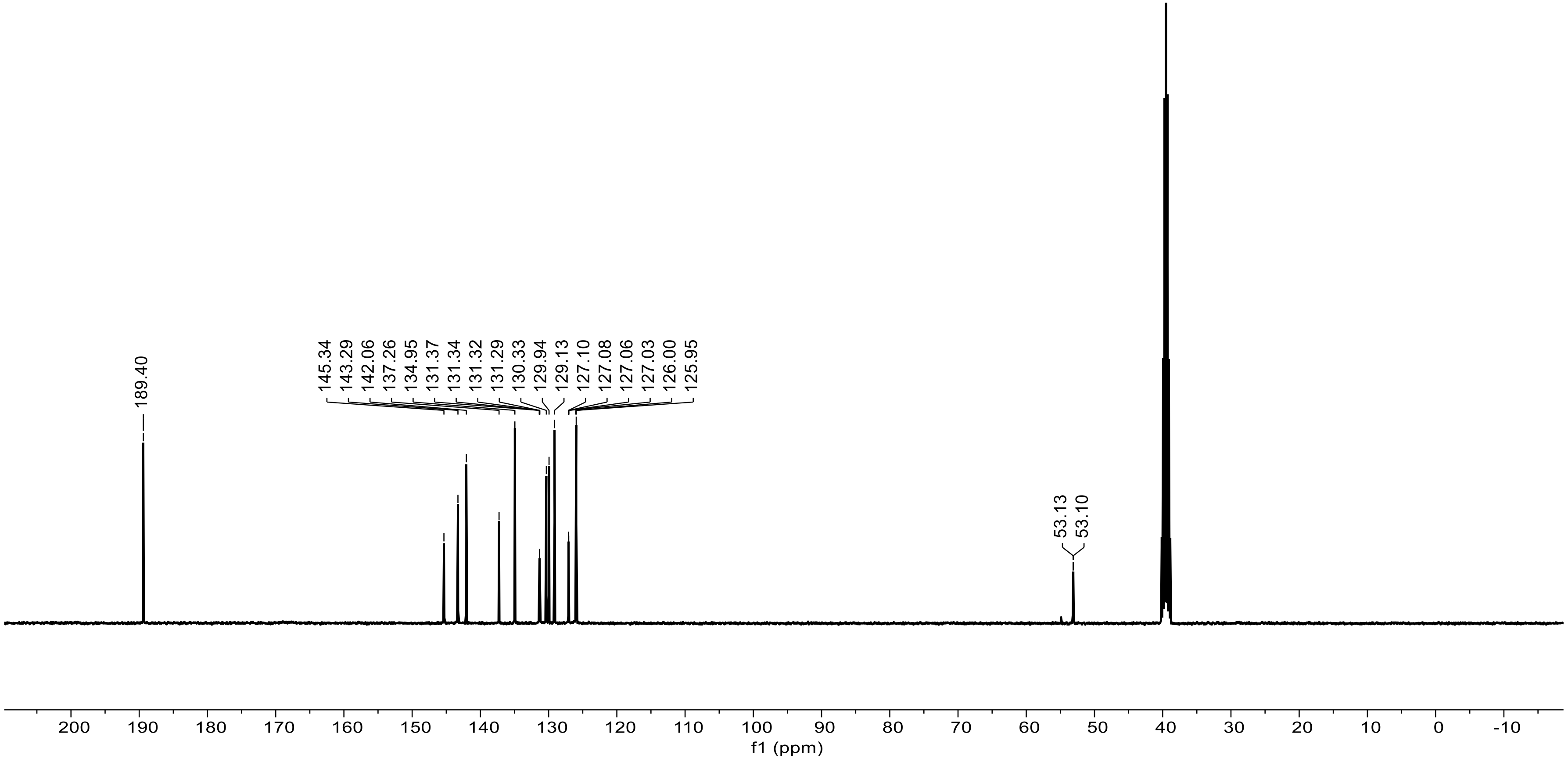

^{19}F NMR of 3f

DMSO-d_6, 376 MHz, 25 °C

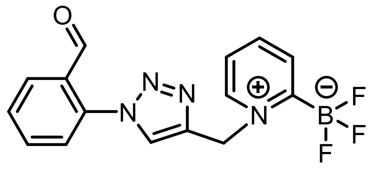

3f

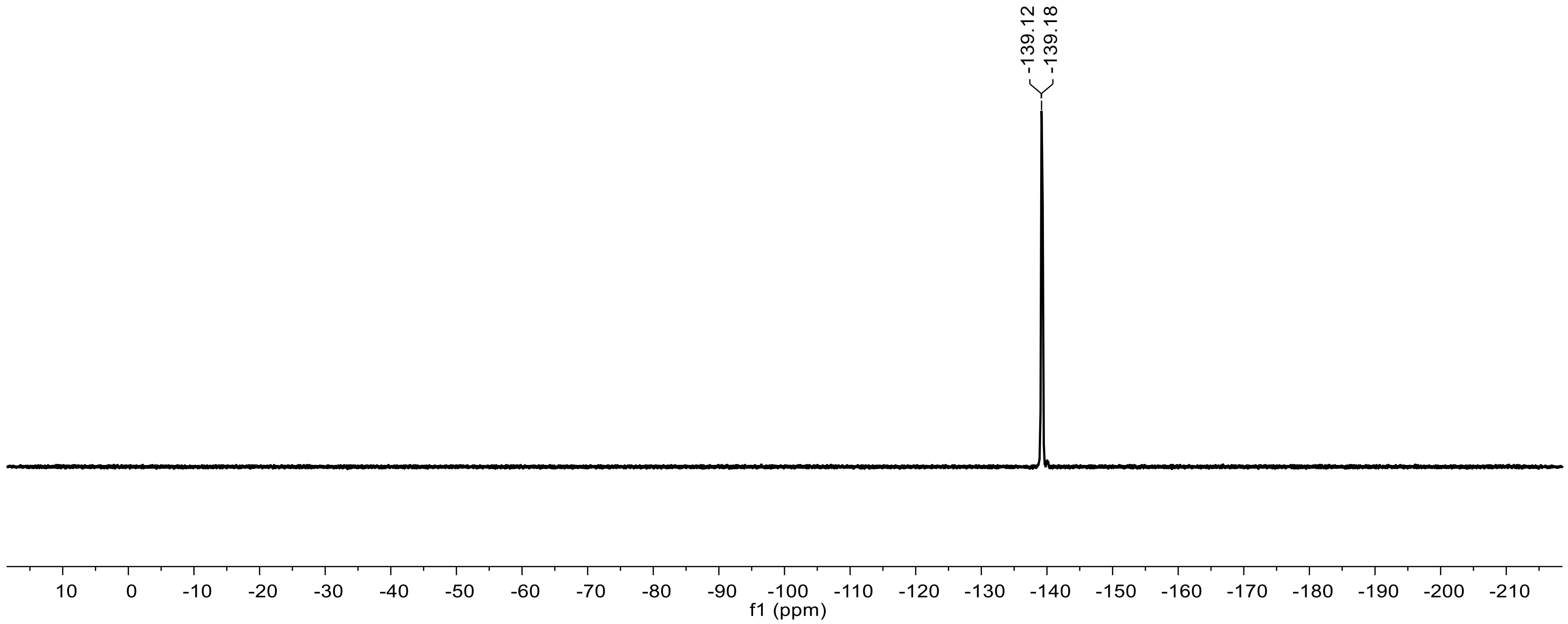

^{11}B NMR of 3f

DMSO-d_6, 128 MHz, 25 °C

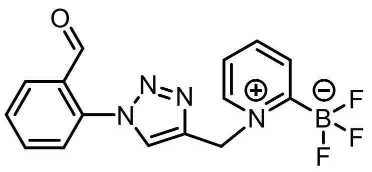

3f

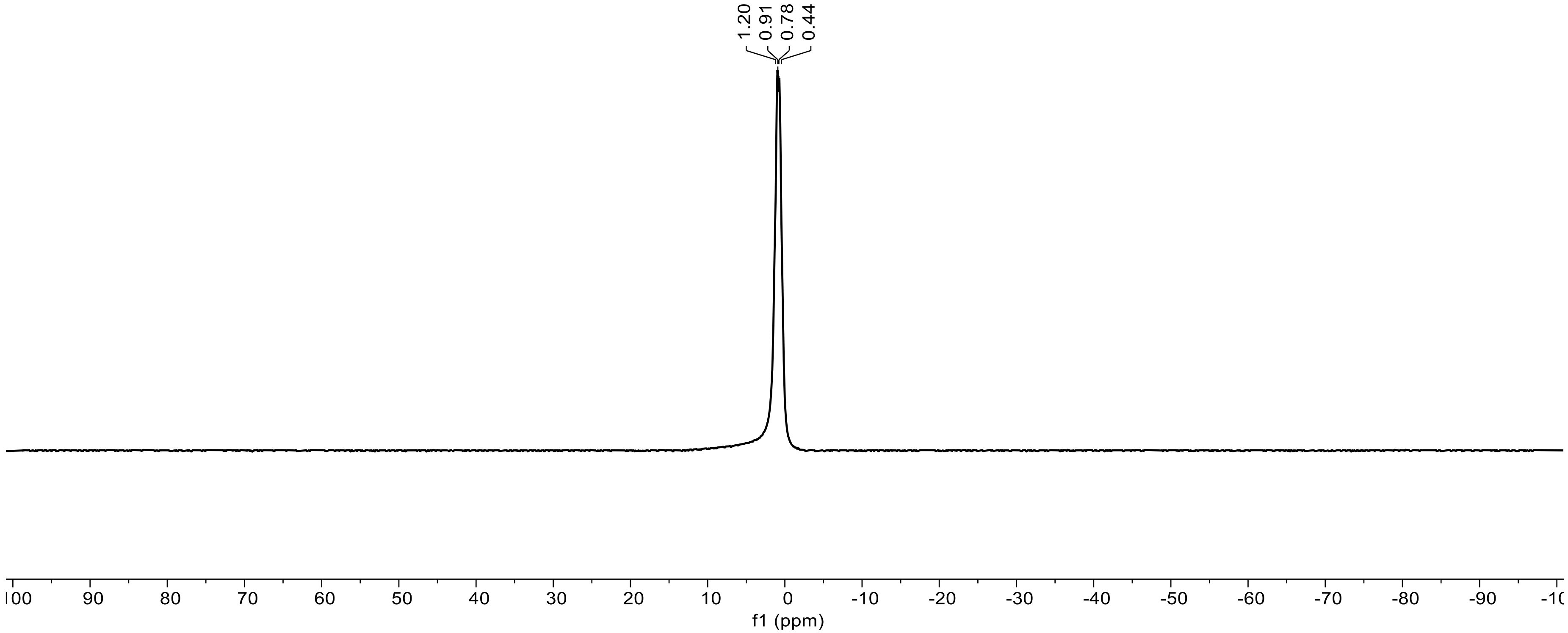

^{1}H NMR of 3g

DMSO-d_6, 400 MHz, 25 °C

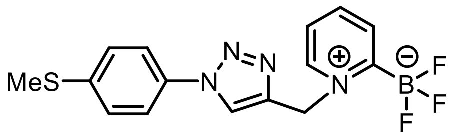

3g

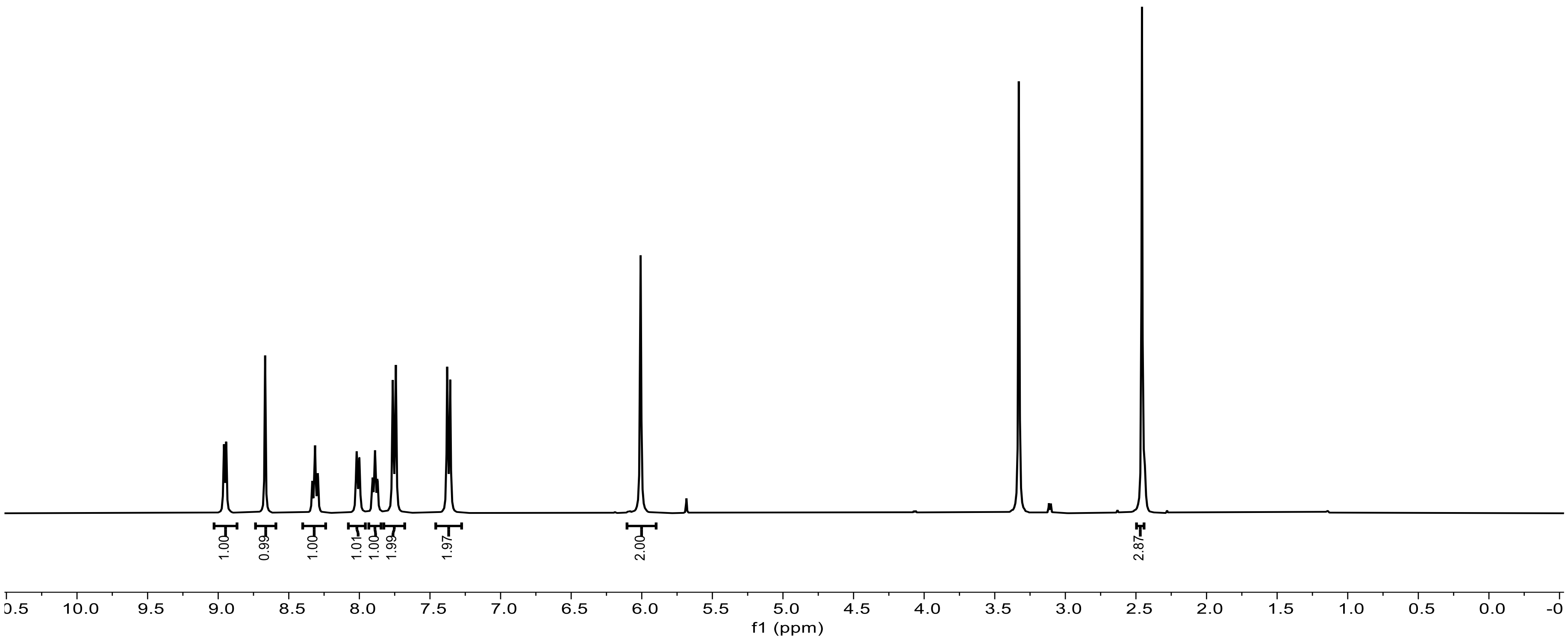

^{13}C NMR of 3g

DMSO-d_6, 101 MHz, 25 °C

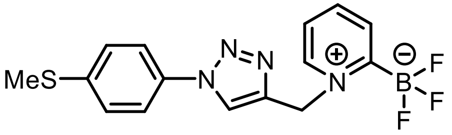

3g

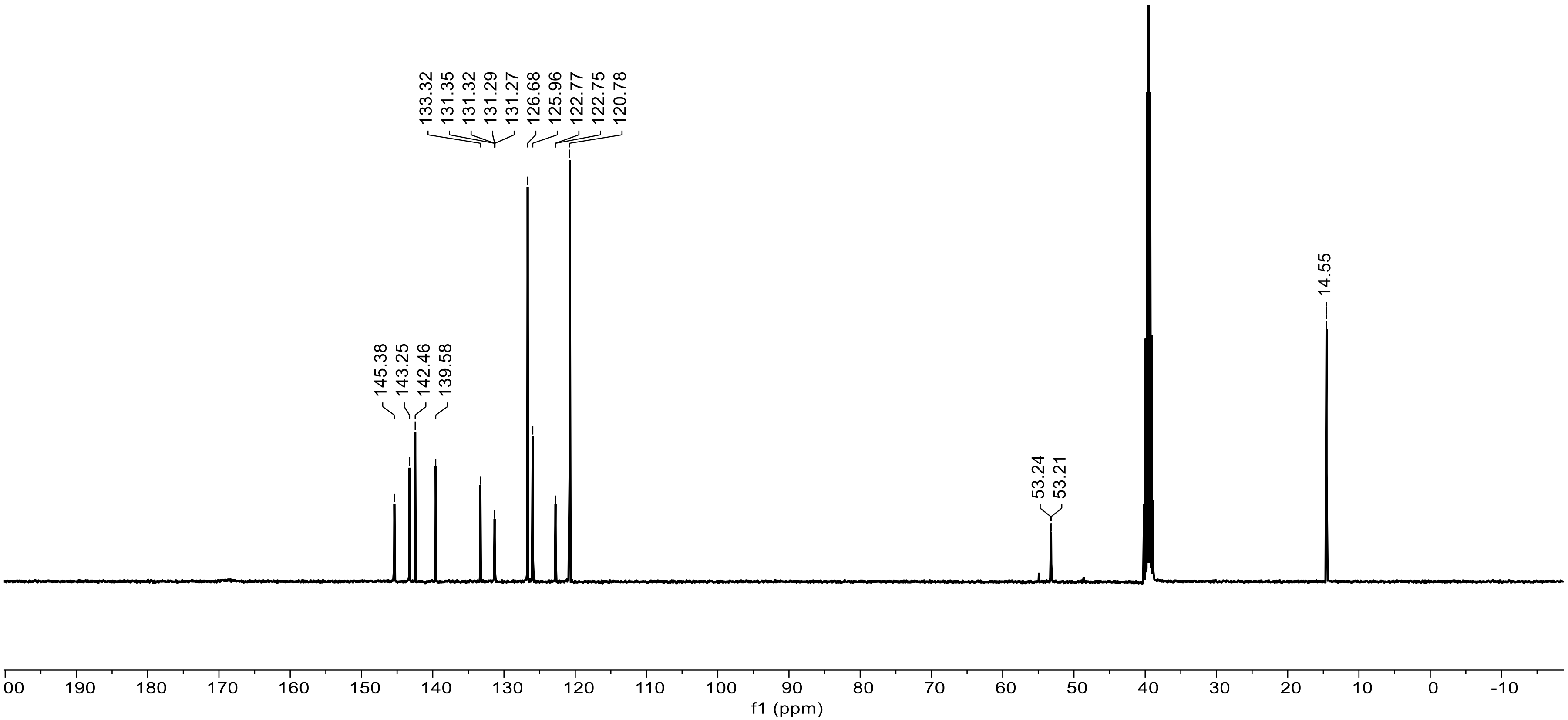

^{19}F NMR of 3g

CD_3CN, 376 MHz, 25 °C

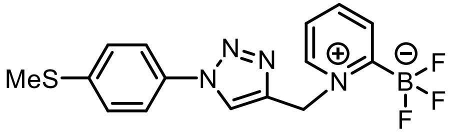

3g

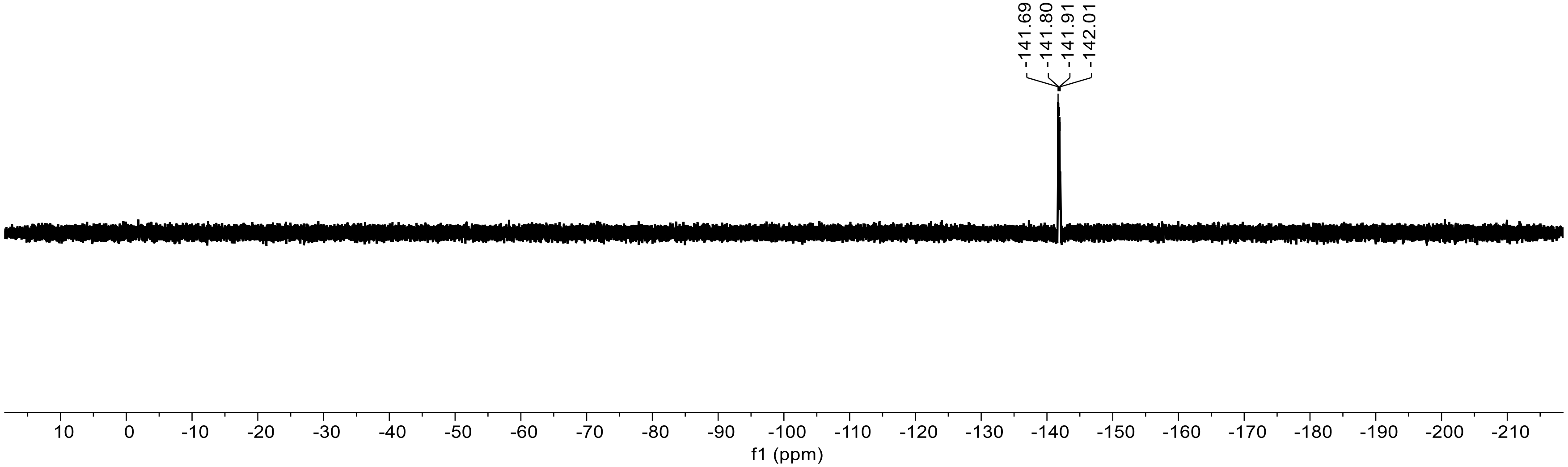

^{11}B NMR of 3g

DMSO-d_6, 128 MHz, 25 °C

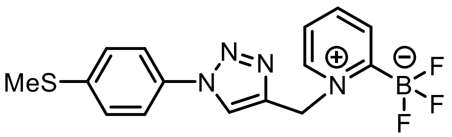

3g

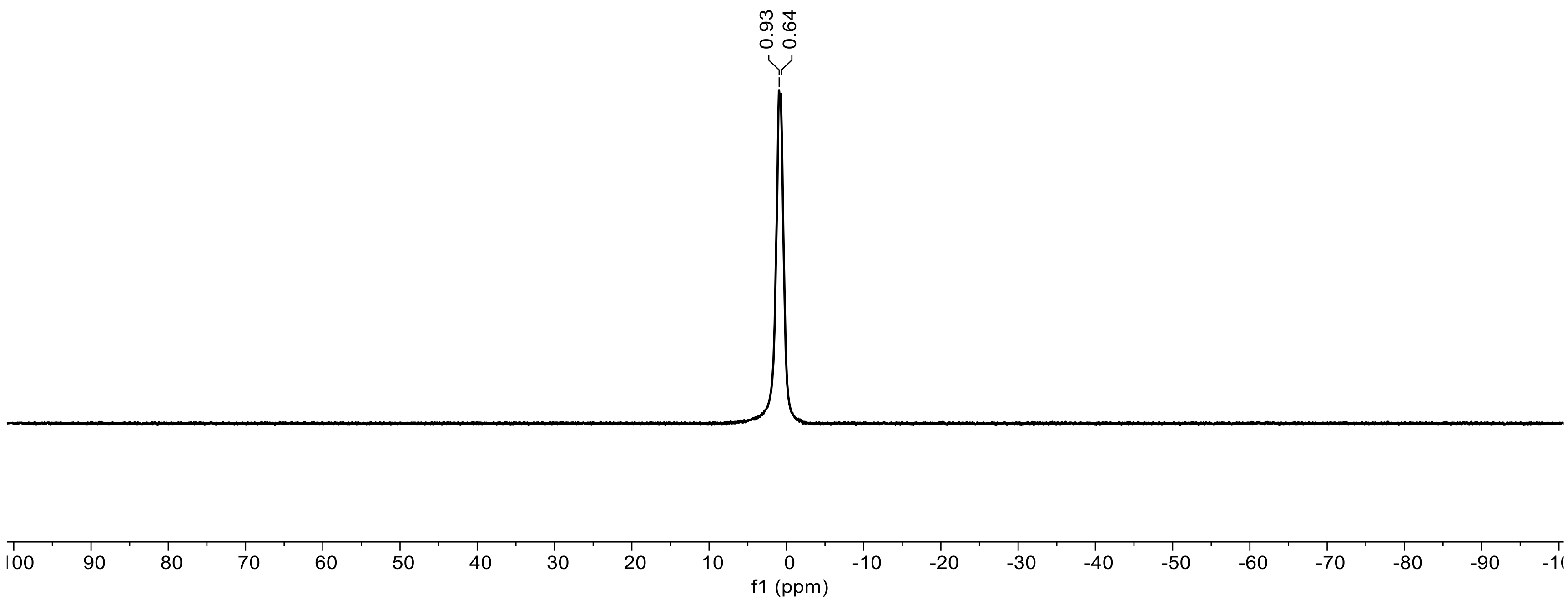

^{1}H NMR of 3h

DMSO-d_6, 400 MHz, 25 °C

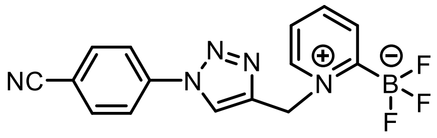

3h

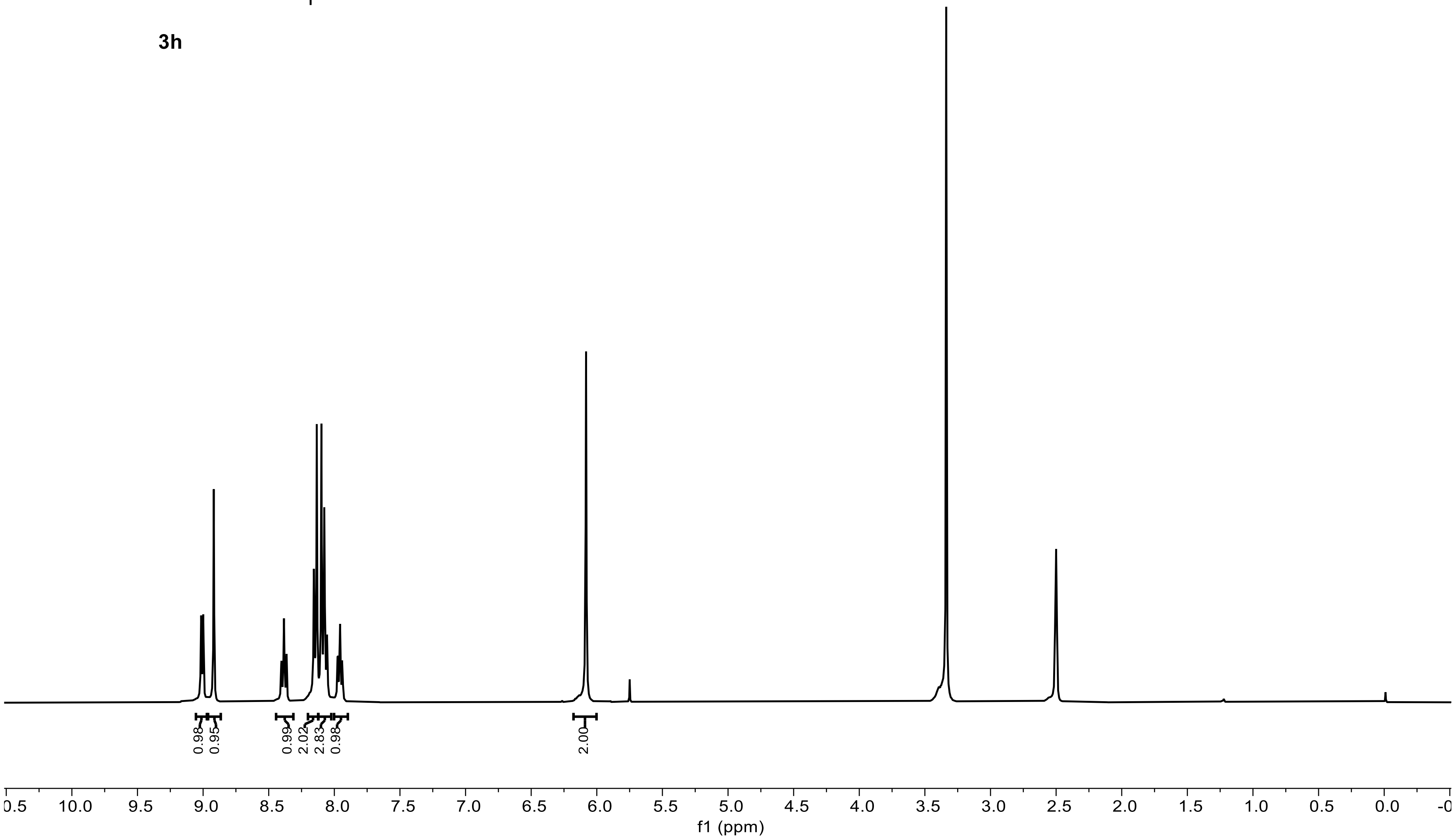

^{13}C NMR of 3h

DMSO-d_6, 101 MHz, 25 °C

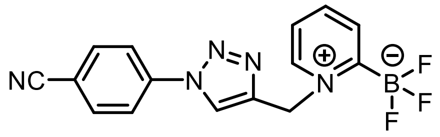

3h

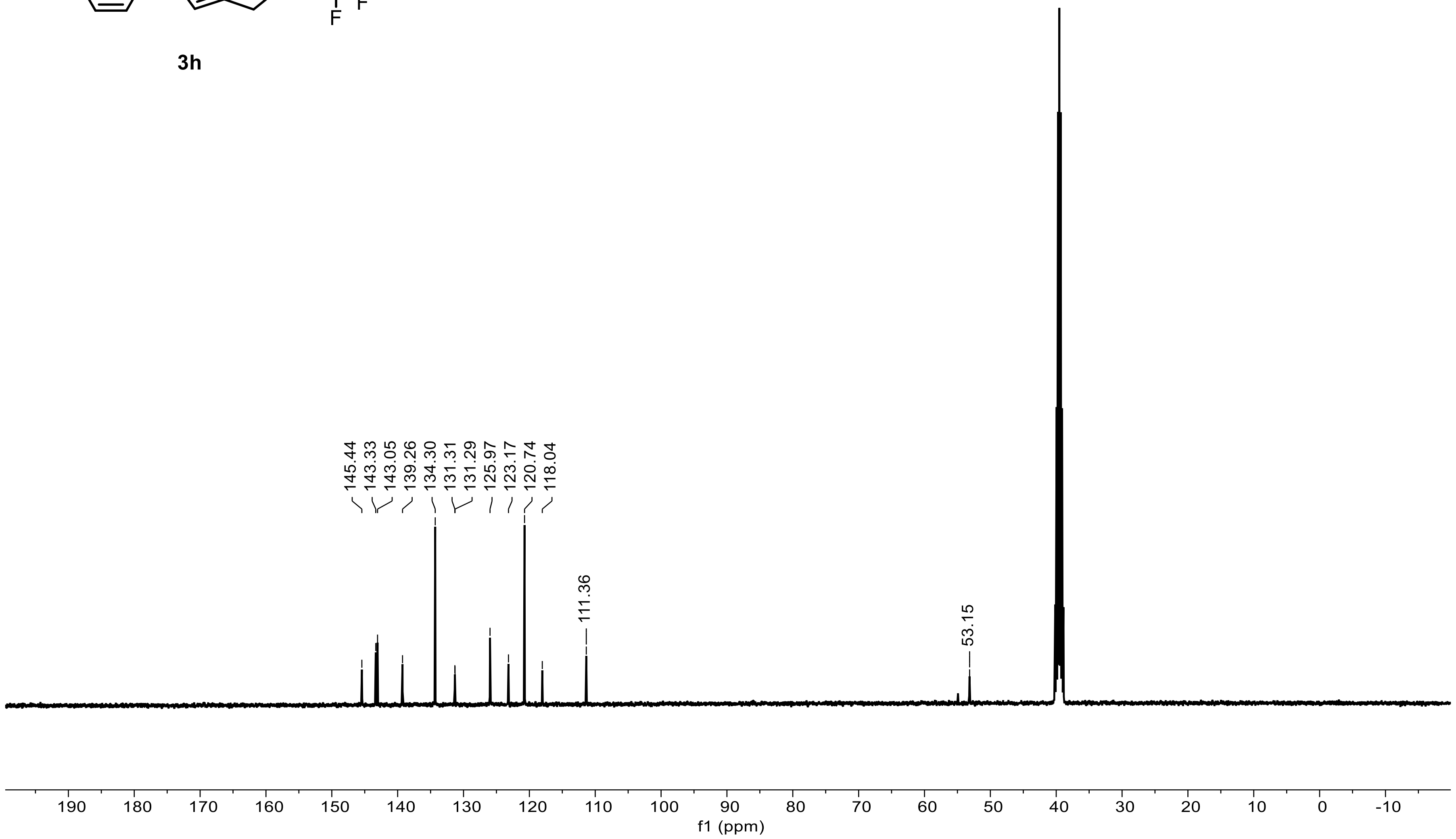

^{19}F NMR of 3h

DMSO-d_6, 376 MHz, 25 °C

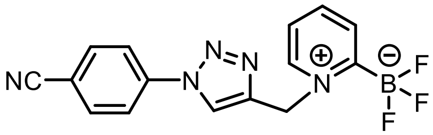

3h

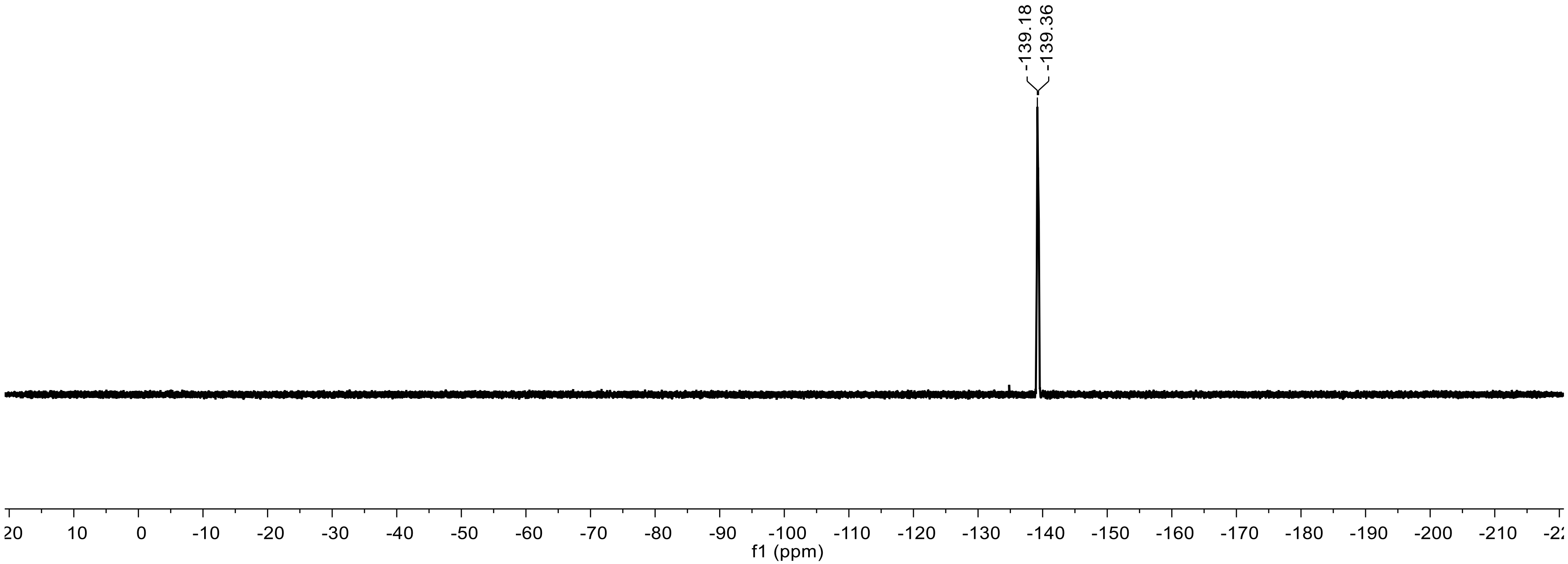

^{11}B NMR of 3h

DMSO-d_6, 128 MHz, 25 °C

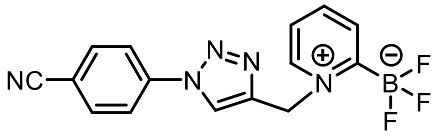

3h

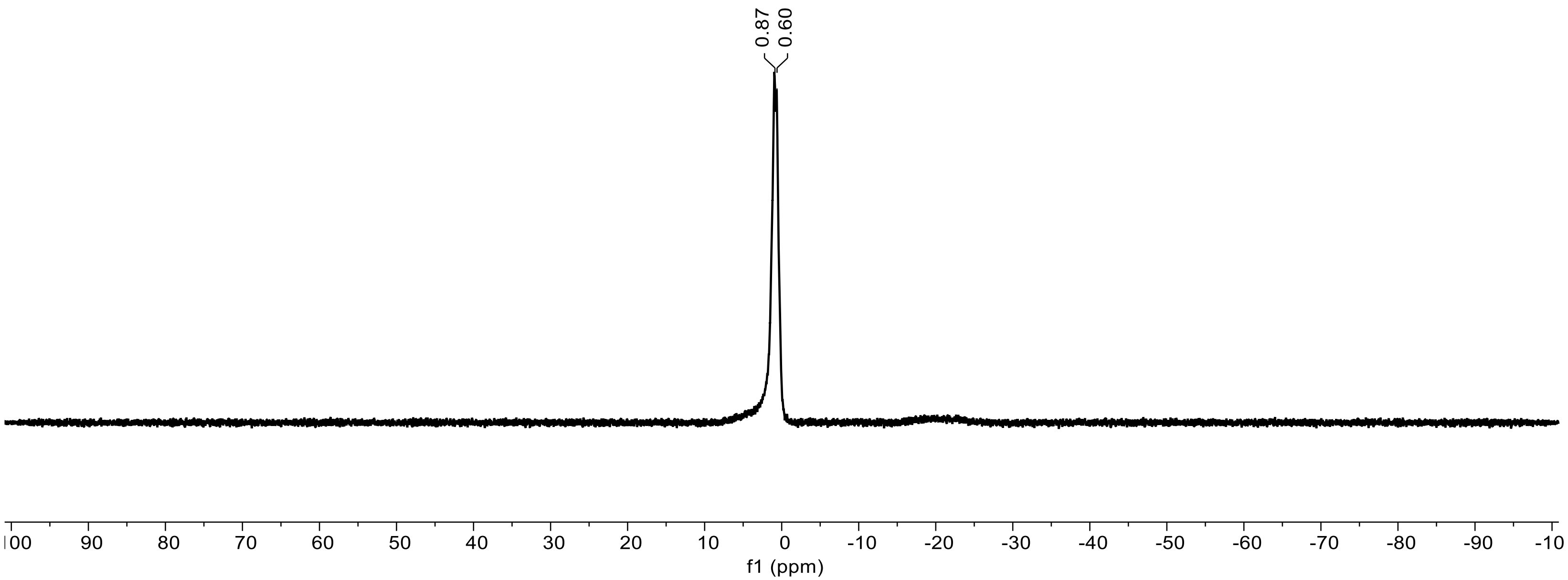

^{1}H NMR of 3i

CDCl$_3$, 400 MHz, 25 °C

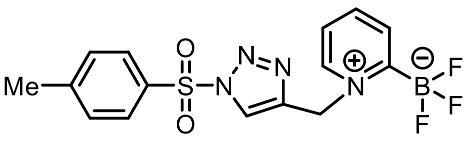

3i

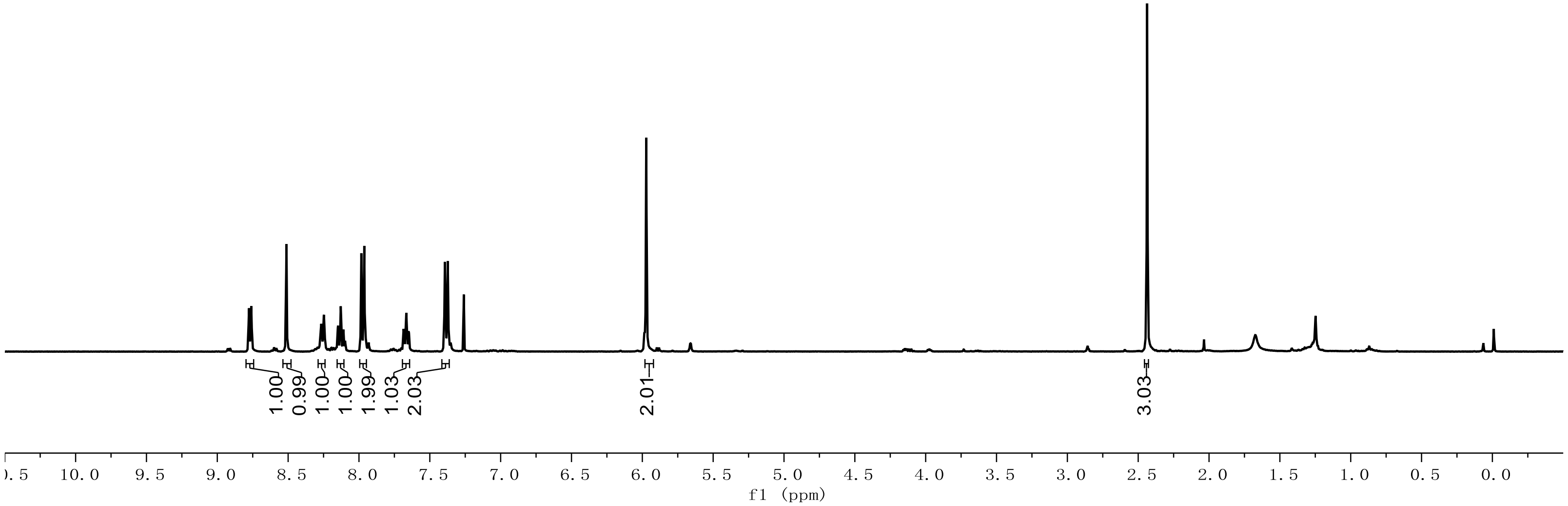

^{13}C NMR of 3i

CDCl$_3$, 101 MHz, 25 °C

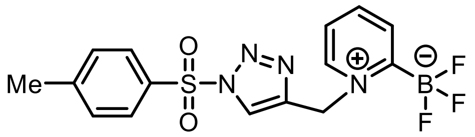

3i

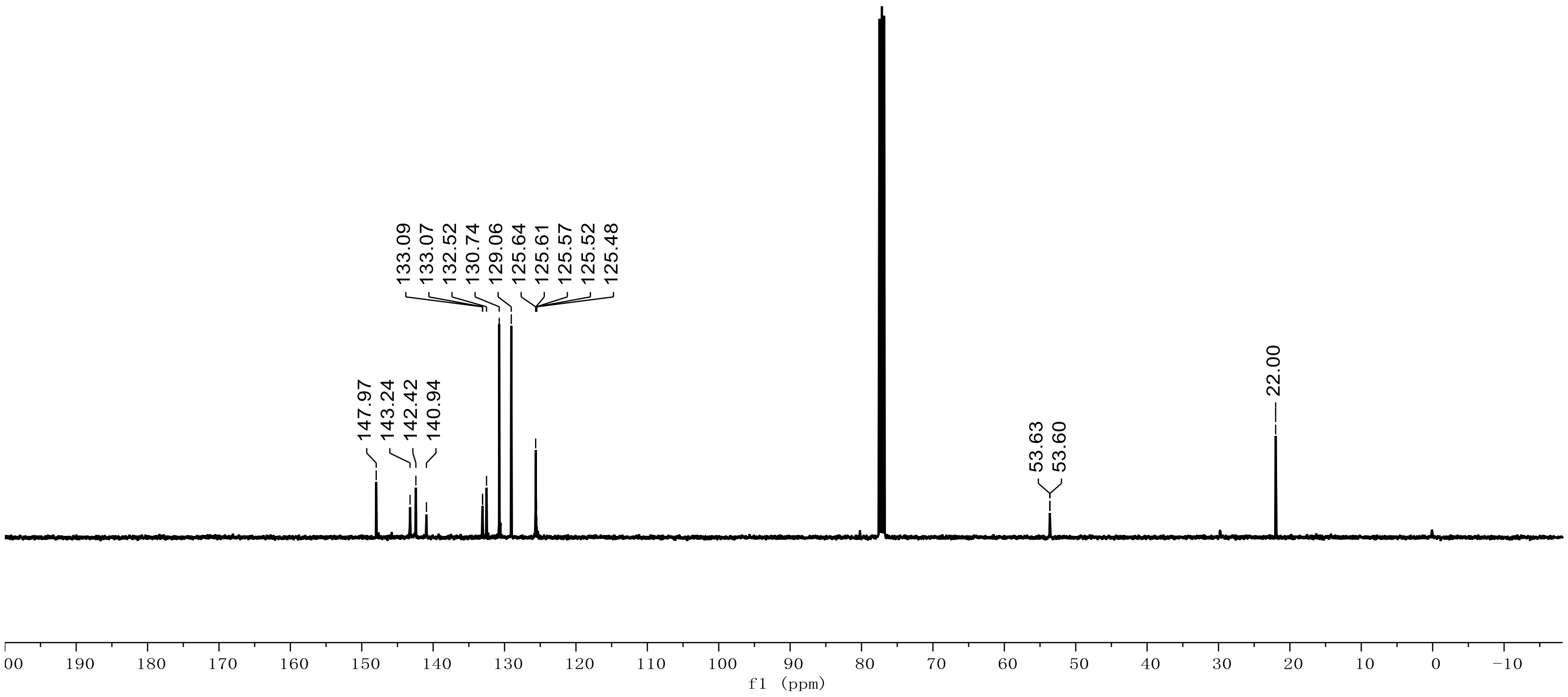

^{19}F NMR of 3i

CDCl$_3$, 376 MHz, 25 °C

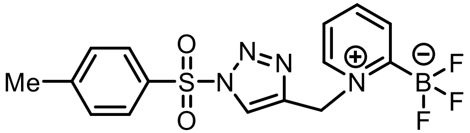

3i

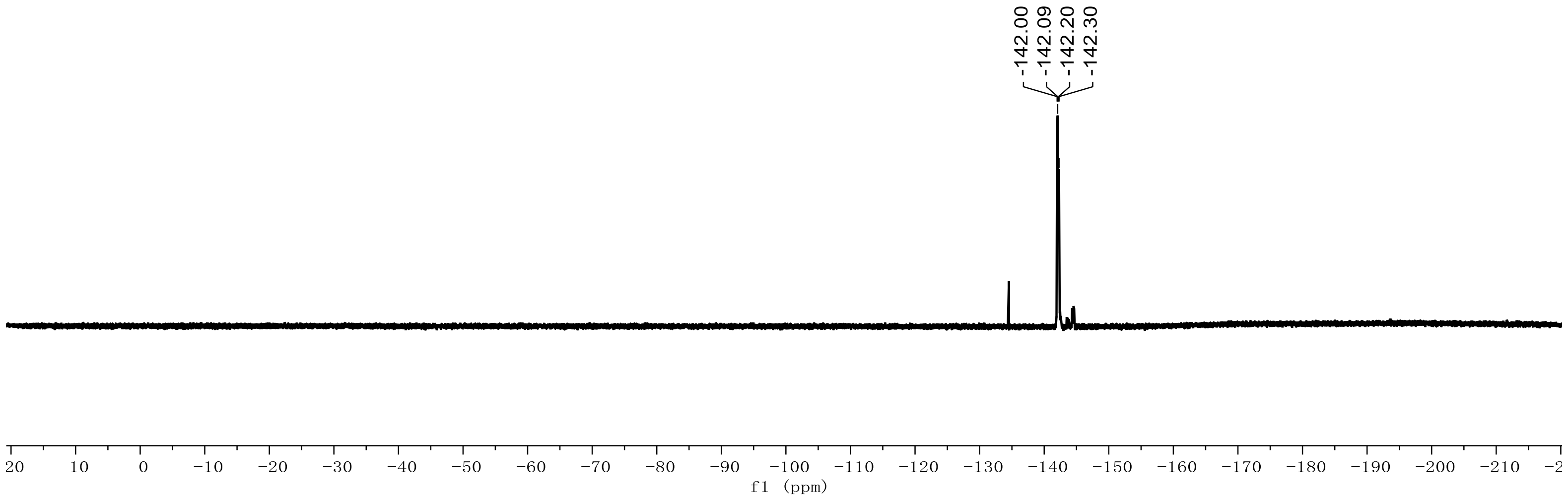

^{11}B NMR of 3i

$CDCl_3$, 128 MHz, 25 °C

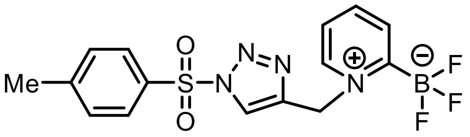

3i

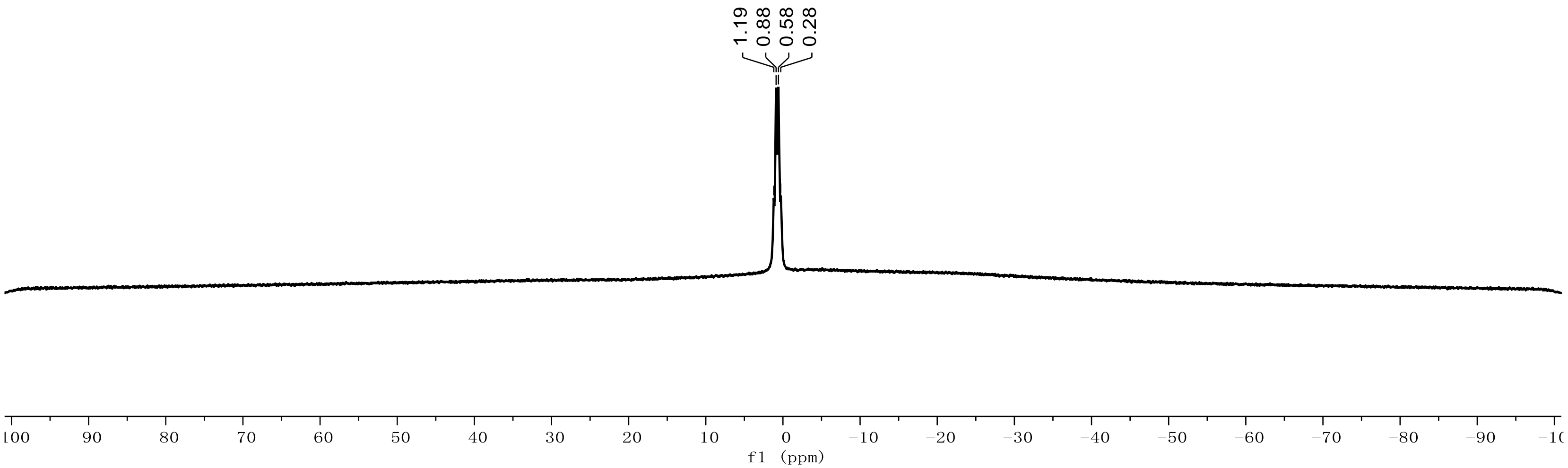

^{1}H NMR of 5j

DMSO-d_6, 400 MHz, 25 °C

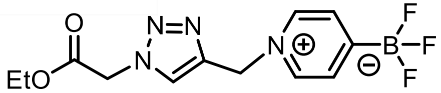

5j

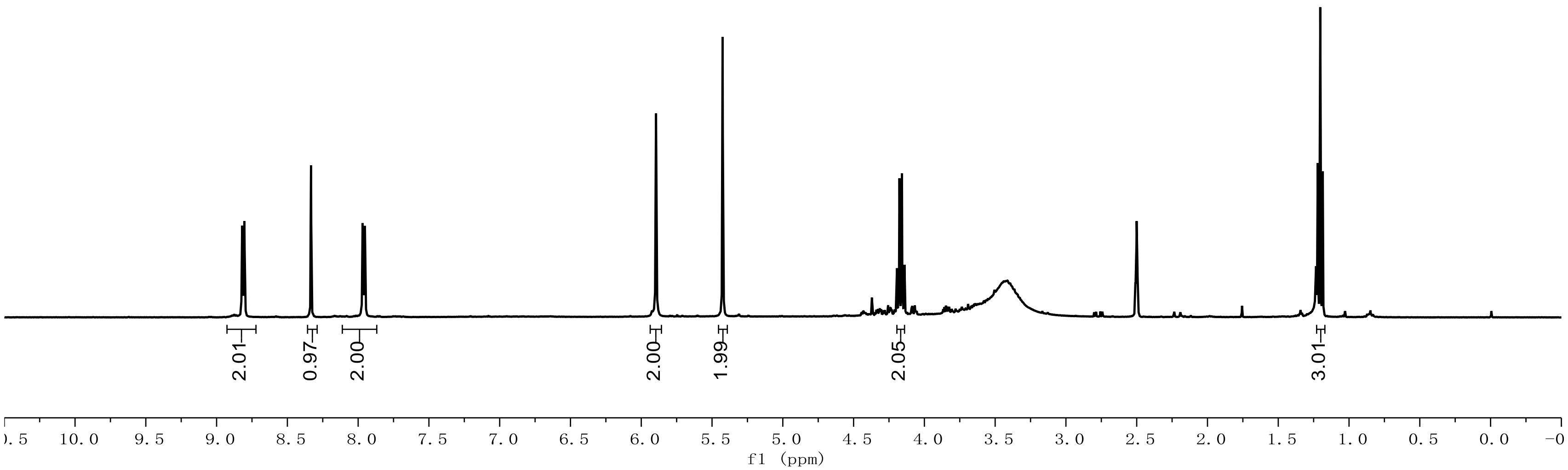

^{13}C NMR of 5j

DMSO-d_6, 101 MHz, 25 °C

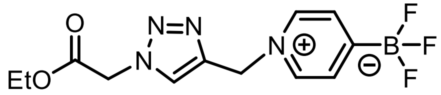

5j

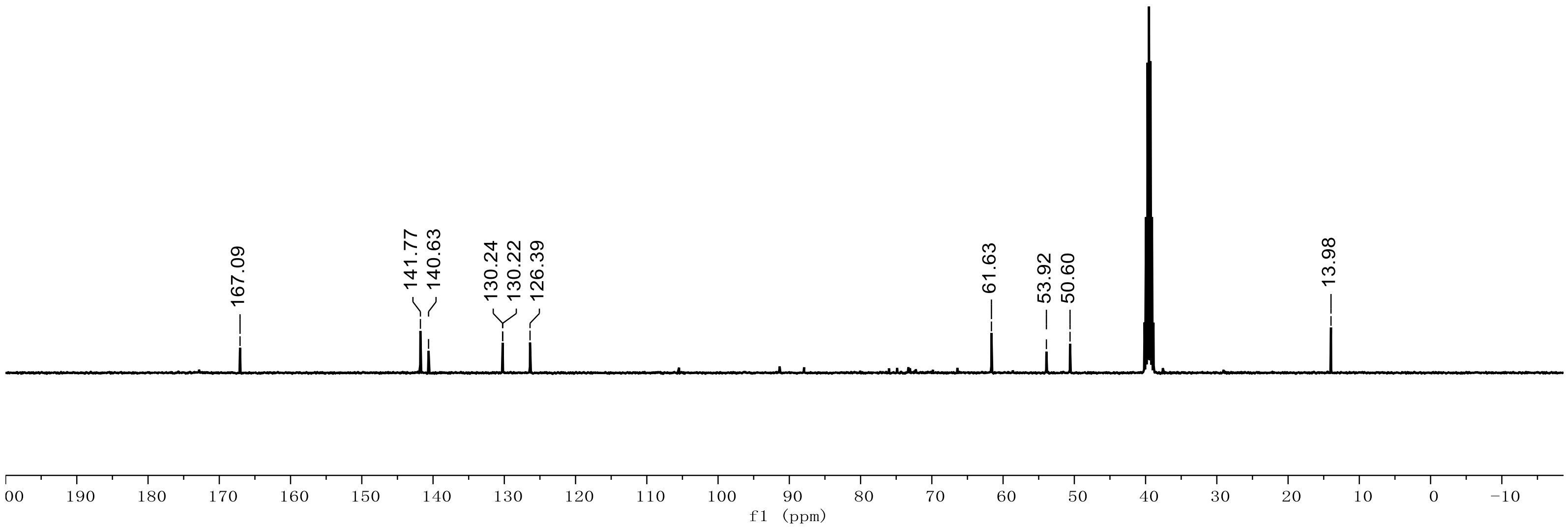

^{19}F NMR of 5j

DMSO-d_6, 376 MHz, 25 °C

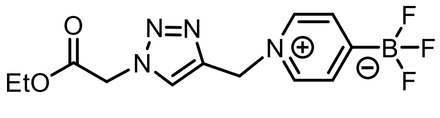

5j

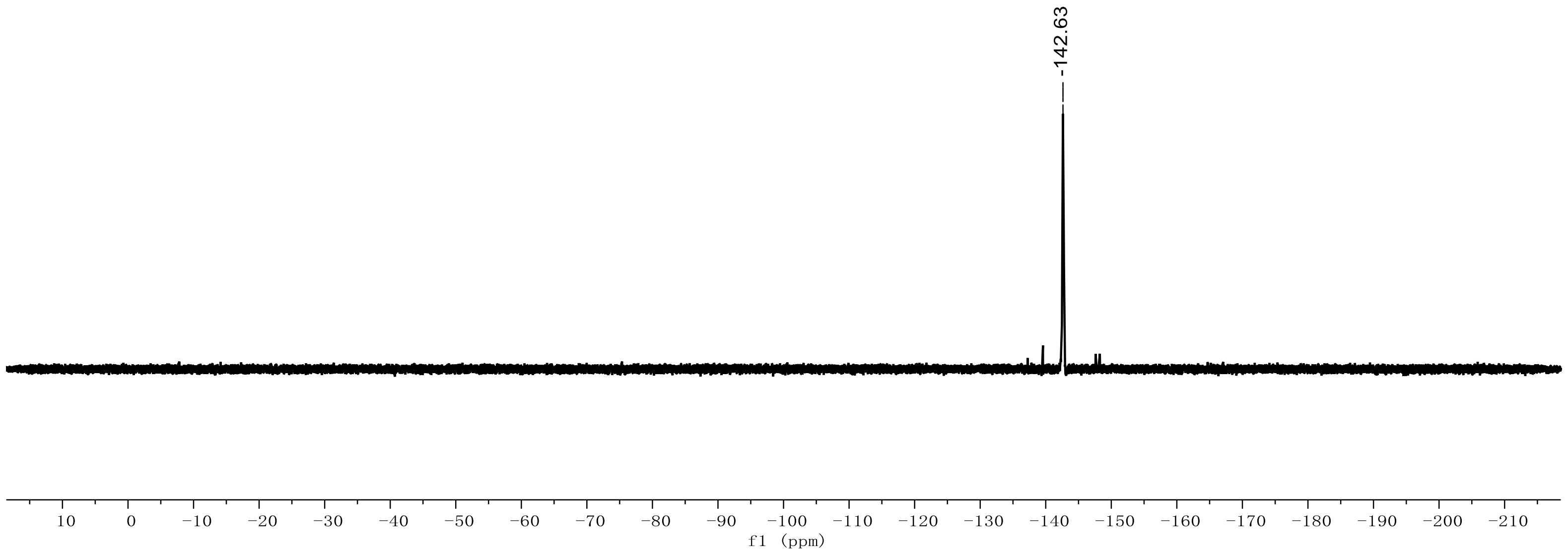

^{11}B NMR of 5j

DMSO-d_6, 128 MHz, 25 °C

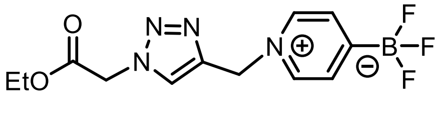

5j

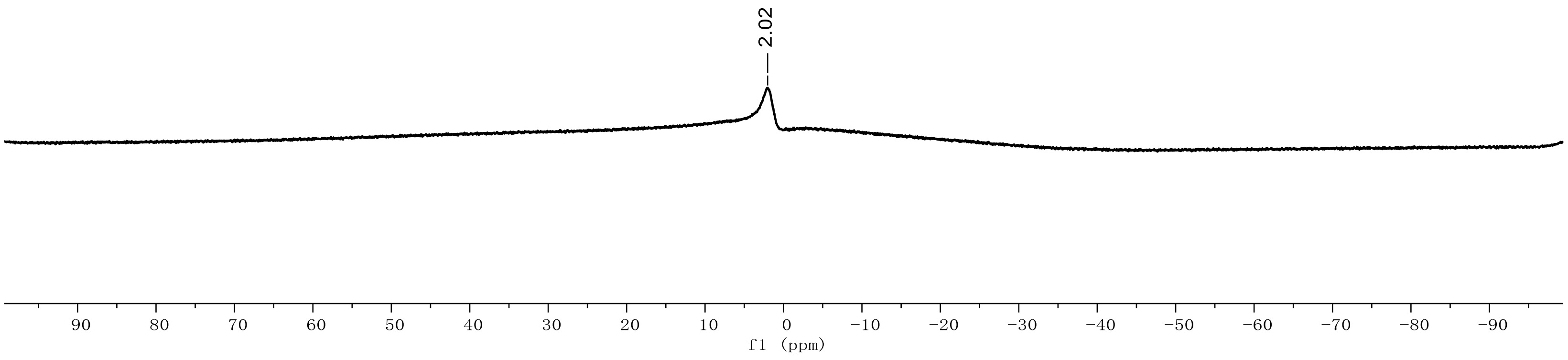

^{1}H NMR of 3k

DMSO-d_6, 400 MHz, 25 °C

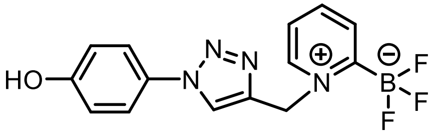

3k

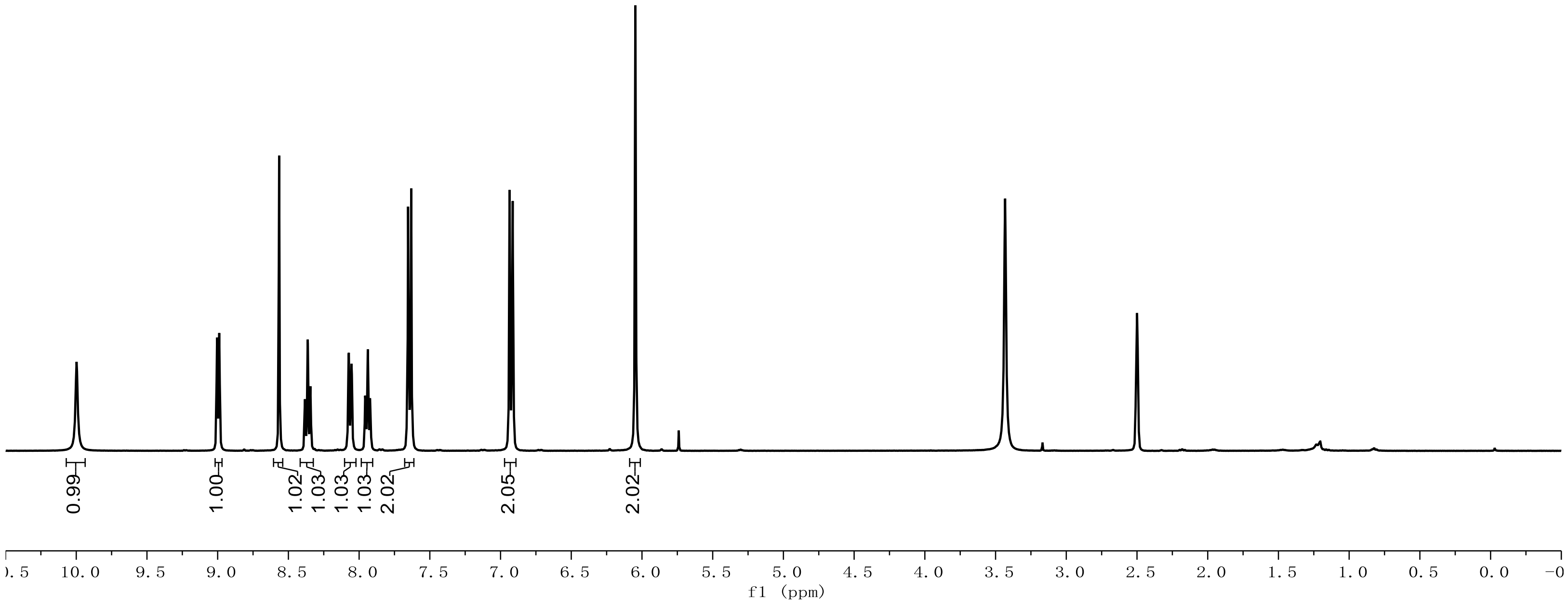

^{13}C NMR of 3k

DMSO-d_6, 101 MHz, 25 °C

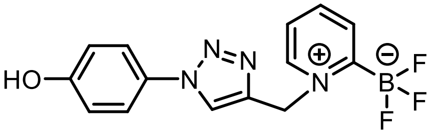

3k

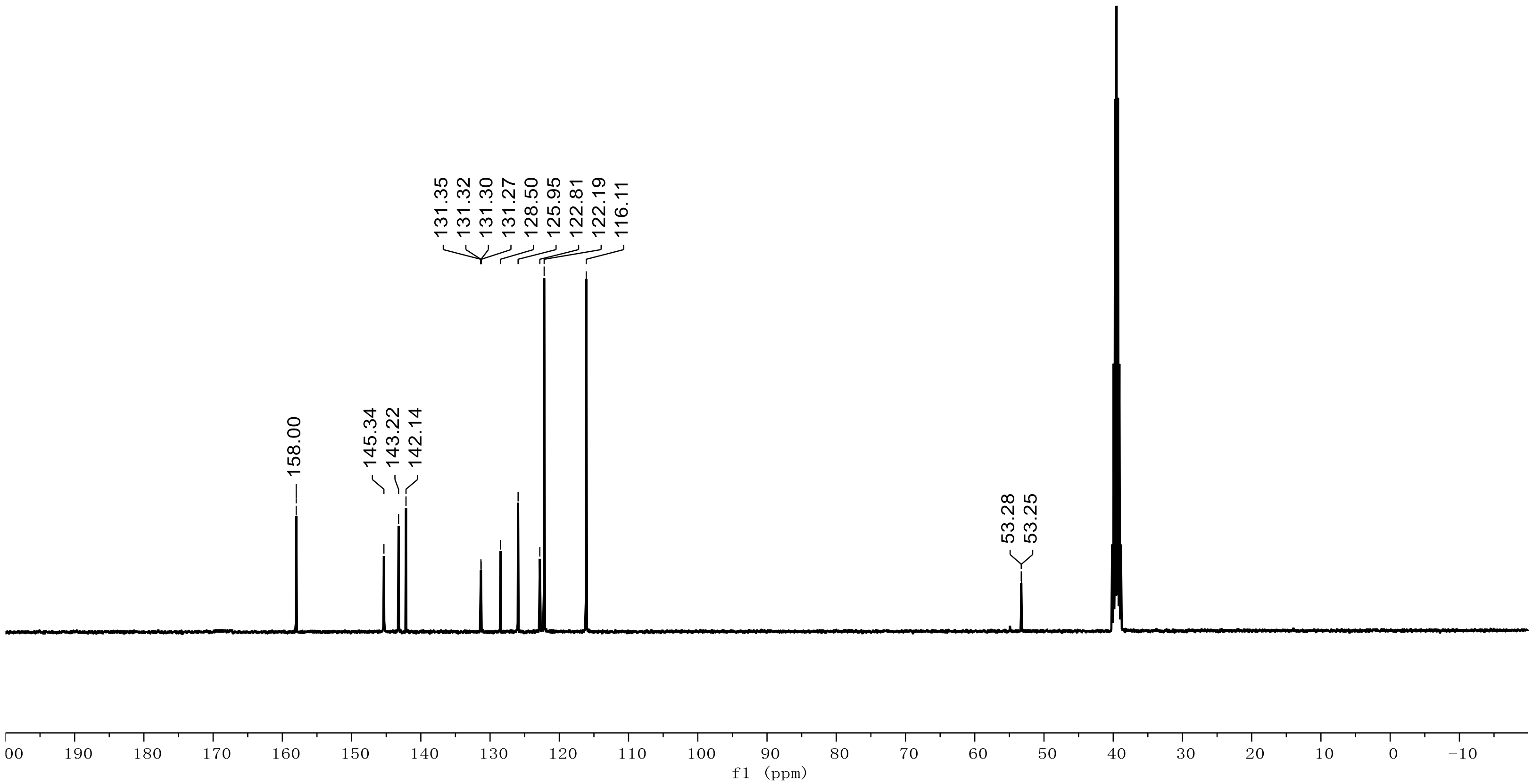

^{19}F NMR of 3k

DMSO-d_6, 376 MHz, 25 °C

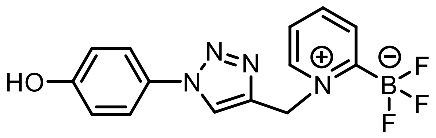

3k

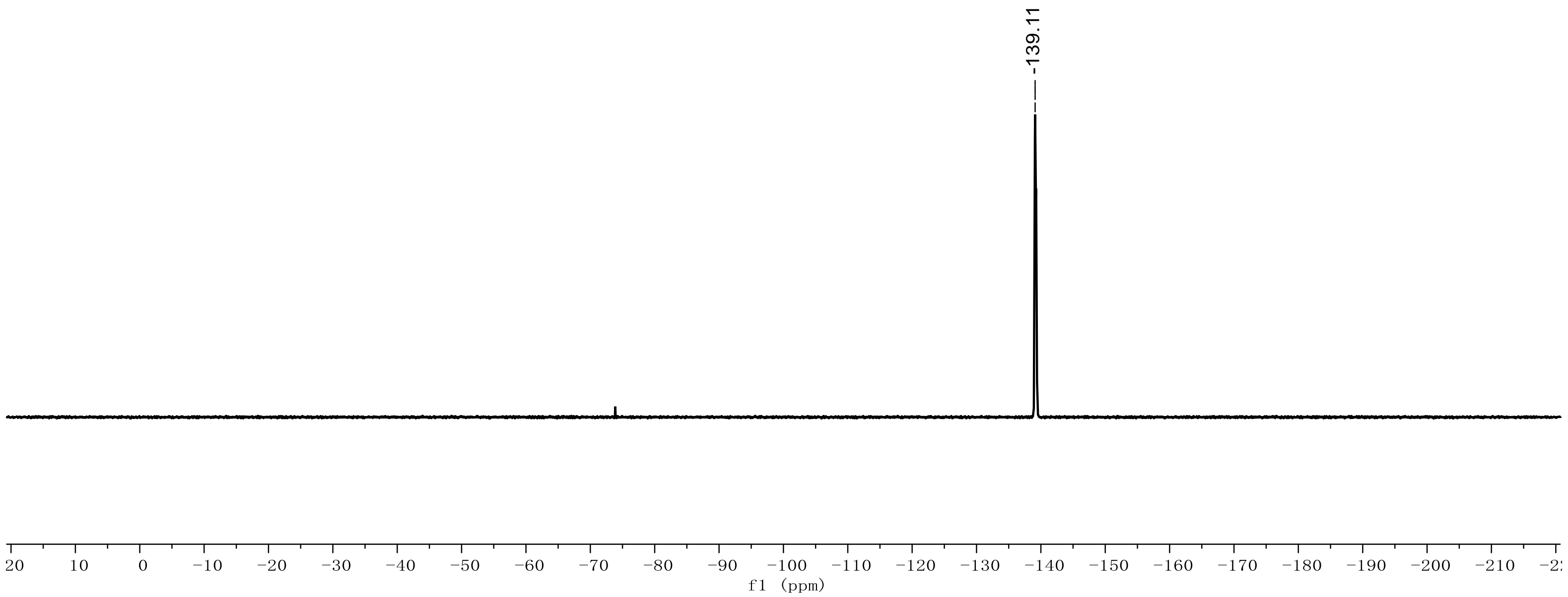

^{11}B NMR of 3k

DMSO-d_6, 128 MHz, 25 °C

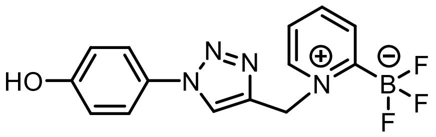

3k

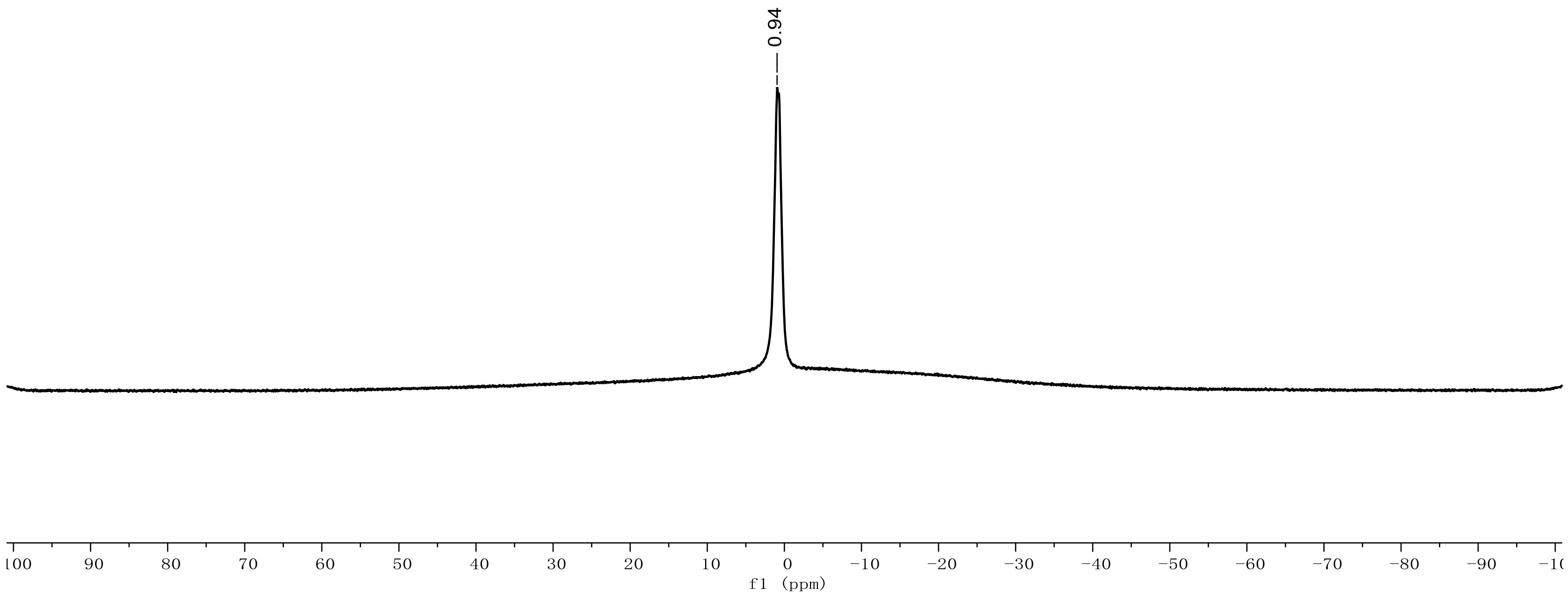

^{1}H NMR of 3l

$CDCl_3$, 400 MHz, 25 °C

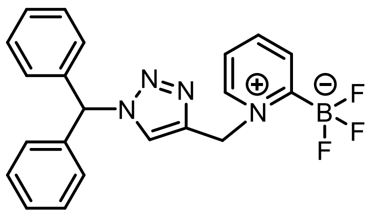

3l

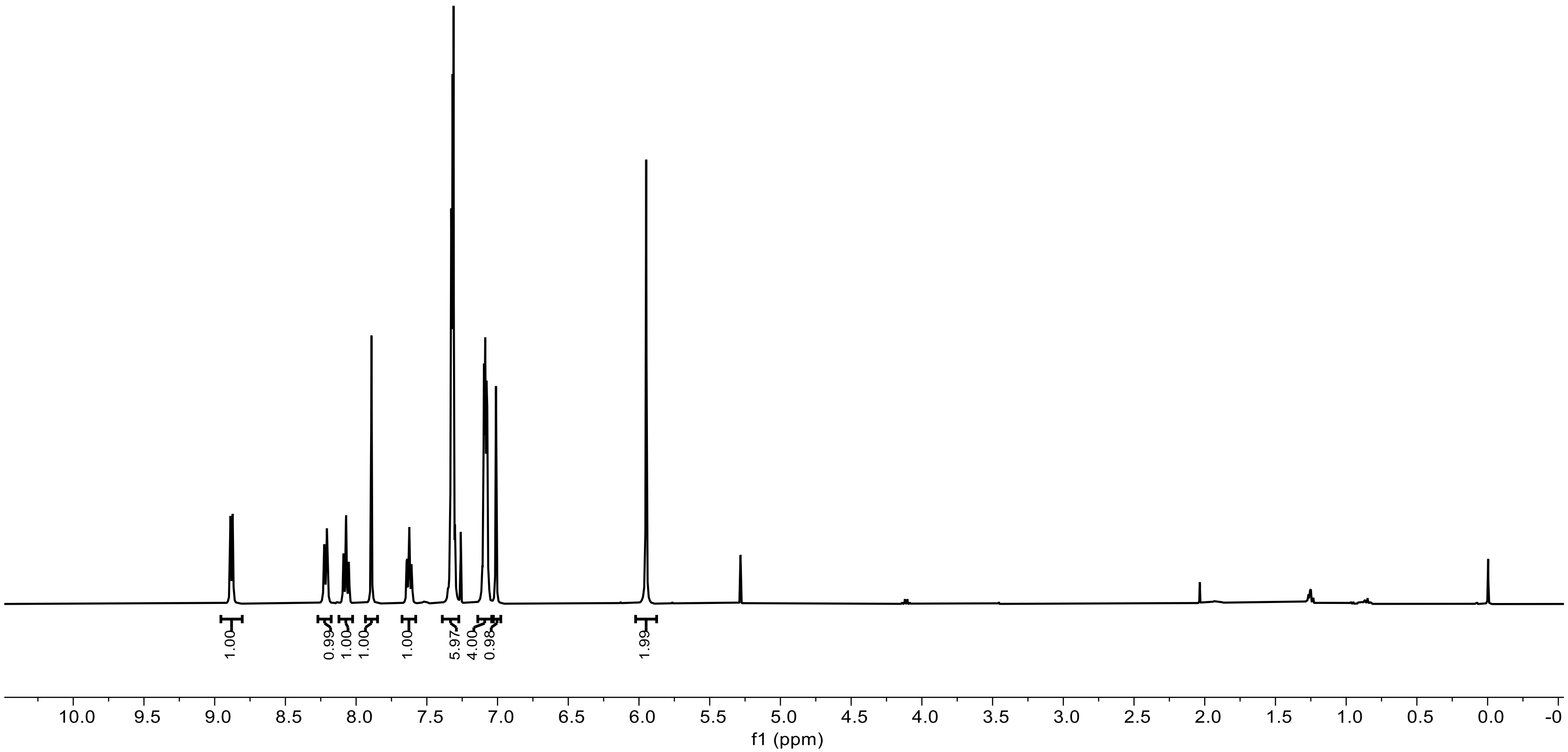

^{13}C NMR of 3l

$CDCl_3$, 101 MHz, 25 °C

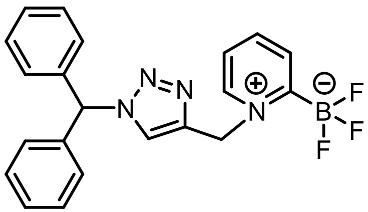

3l

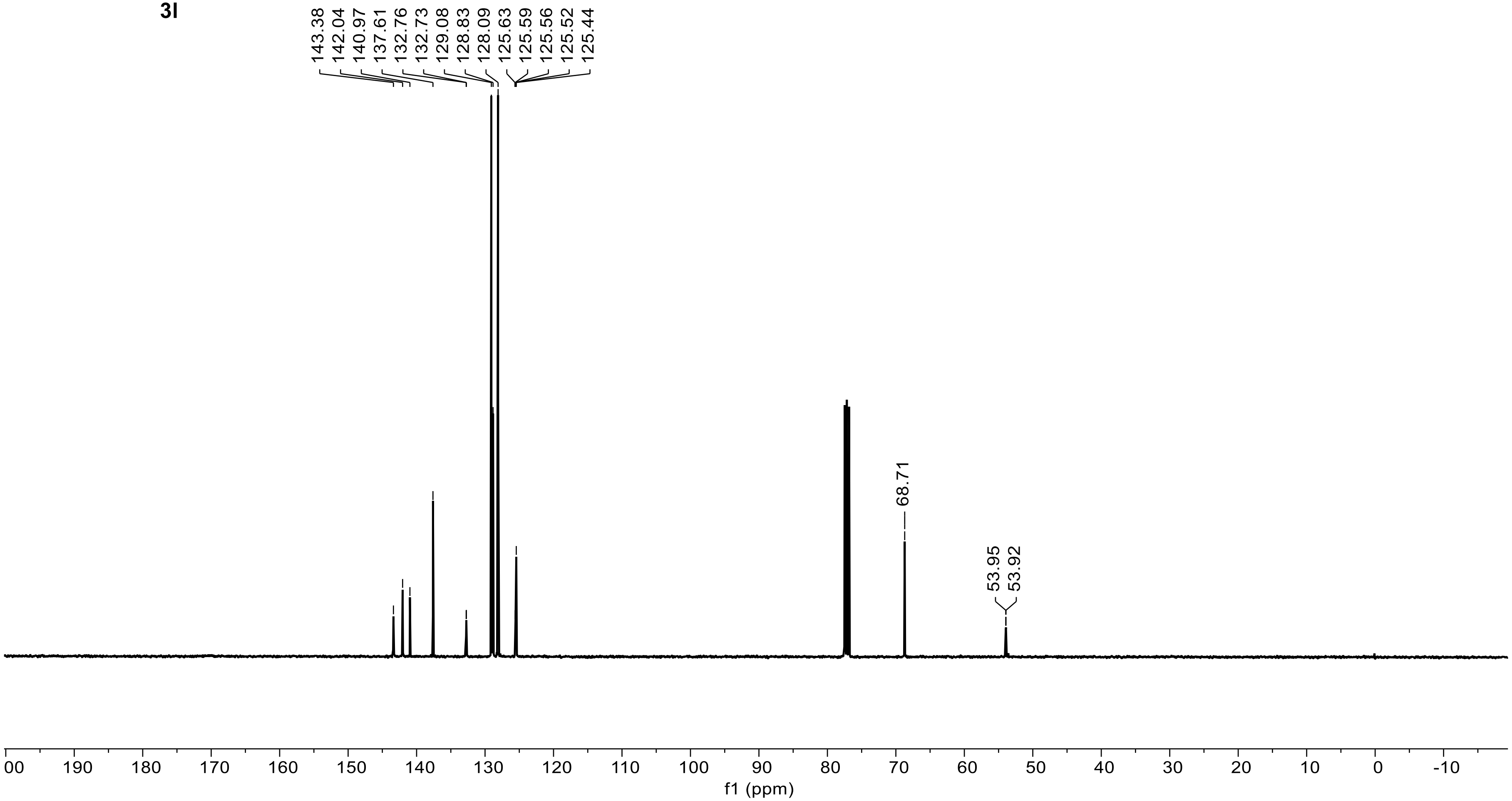

^{19}F NMR of 3l

CDCl$_3$, 376 MHz, 25 °C

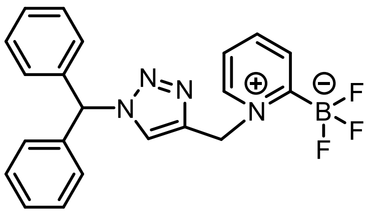

3l

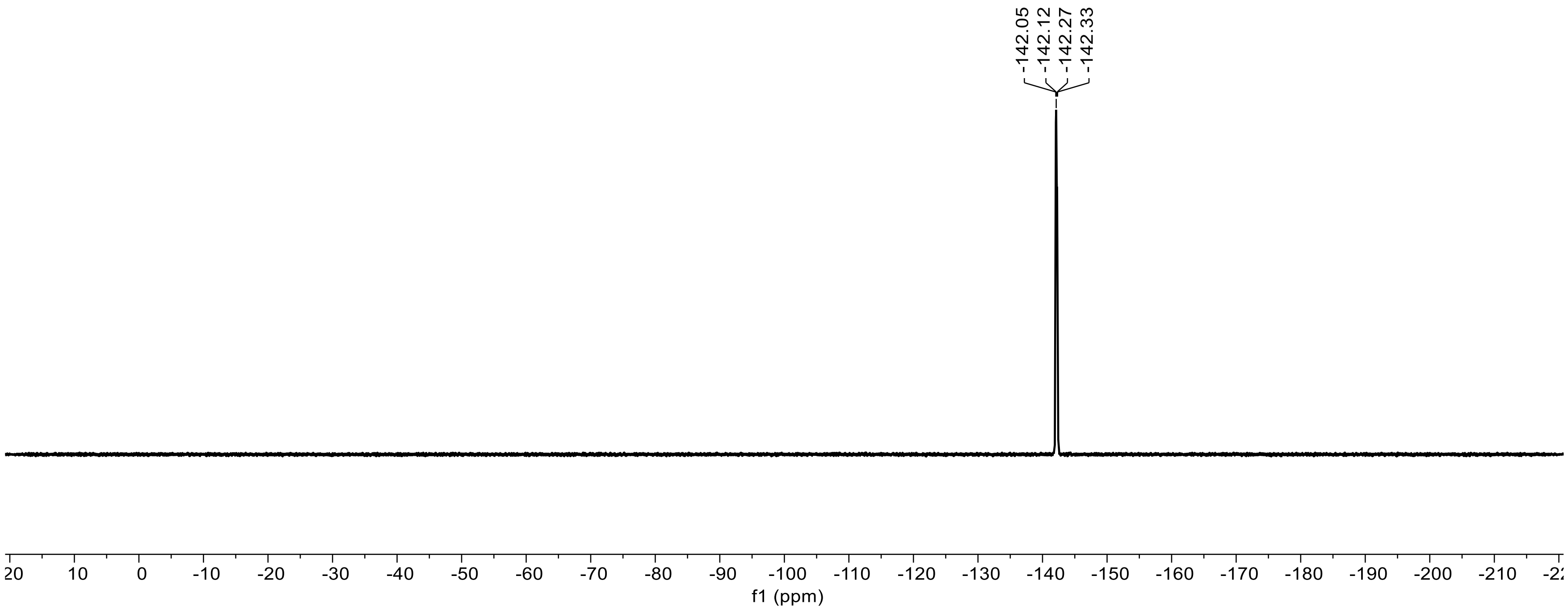

^{11}B NMR of 3l

$CDCl_3$, 128 MHz, 25 °C

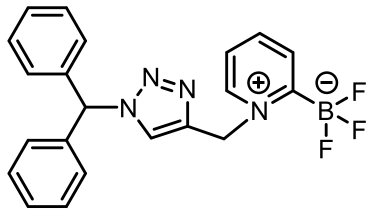

3l

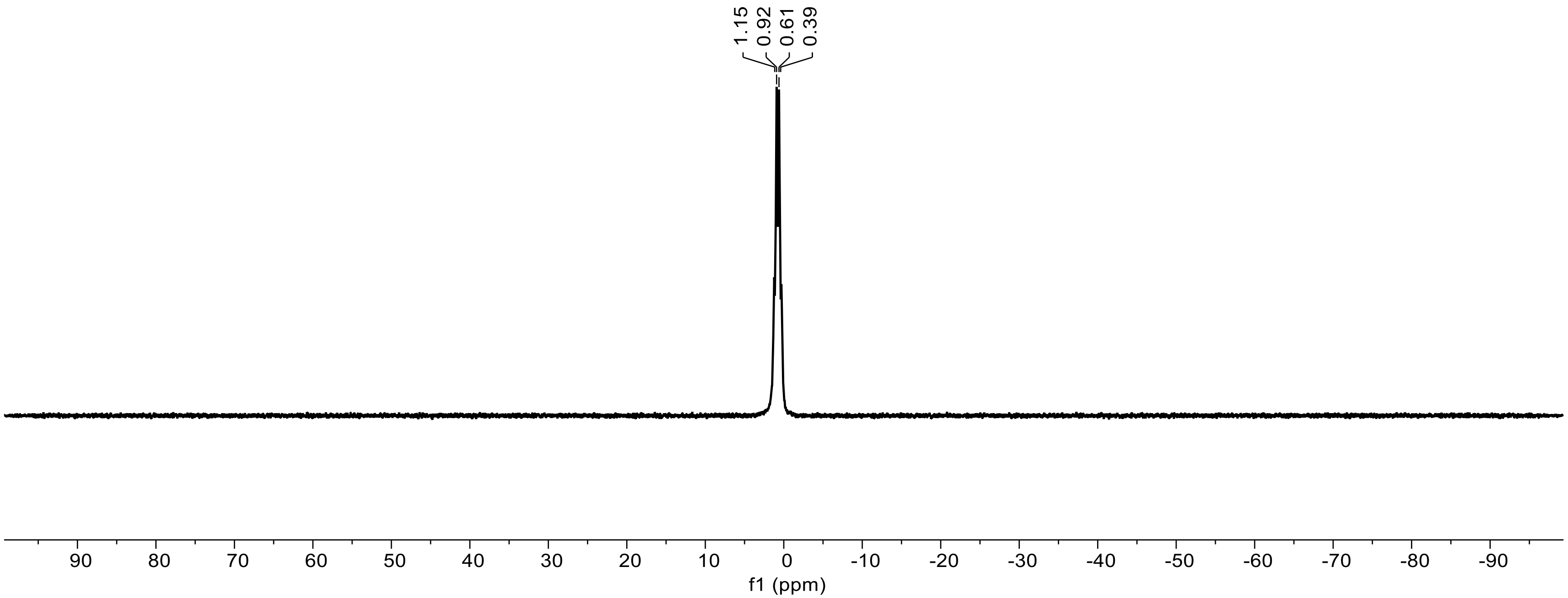

^{1}H NMR of 4m

DMSO-d_6, 400 MHz, 25 °C

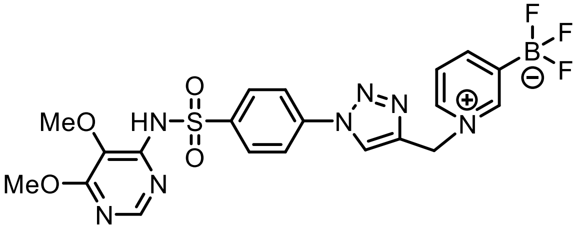

4m

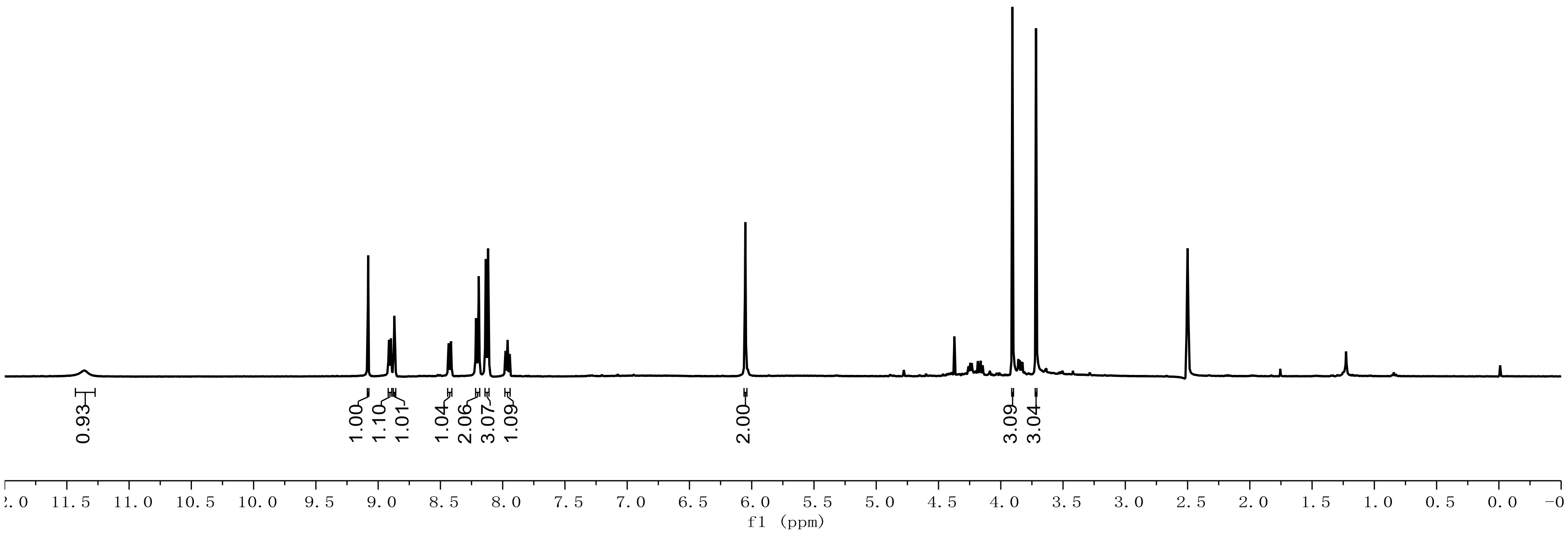

^{13}C NMR of 4m

DMSO-d_6, 101 MHz, 25 °C

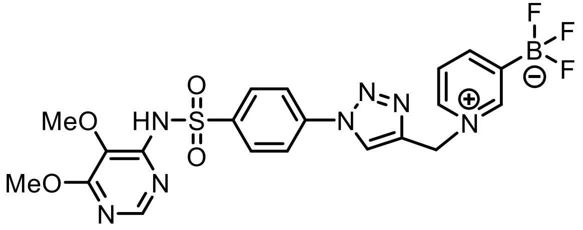

4m

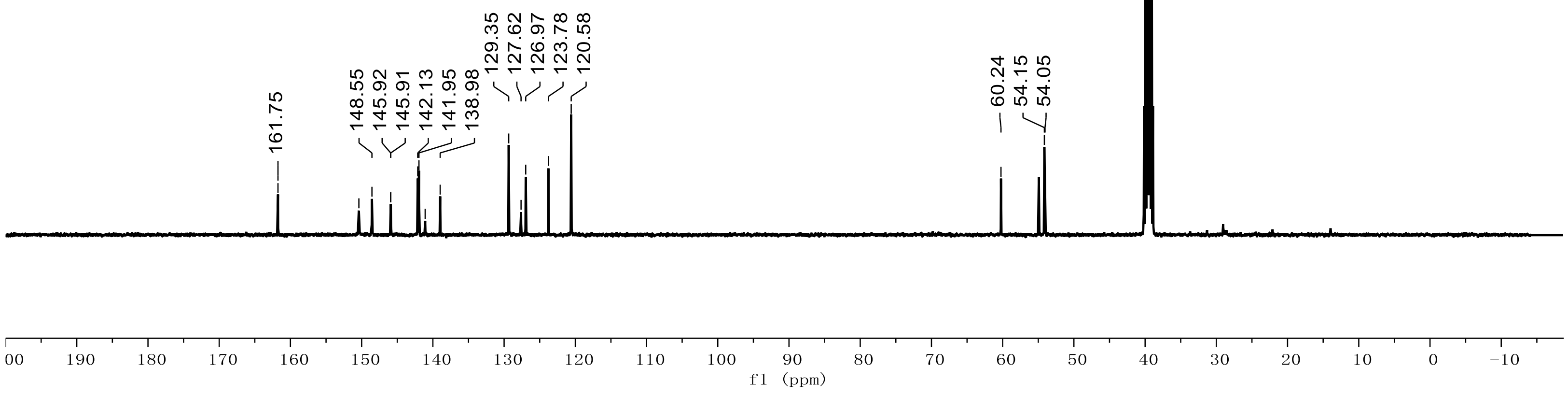

^{19}F NMR of 4m

DMSO-d_6, 376 MHz, 25 °C

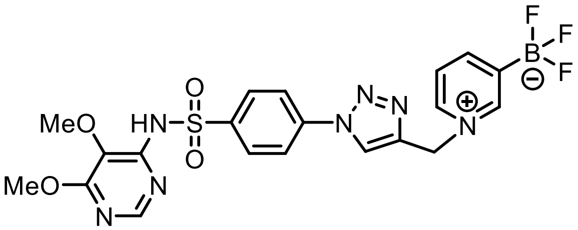

4m

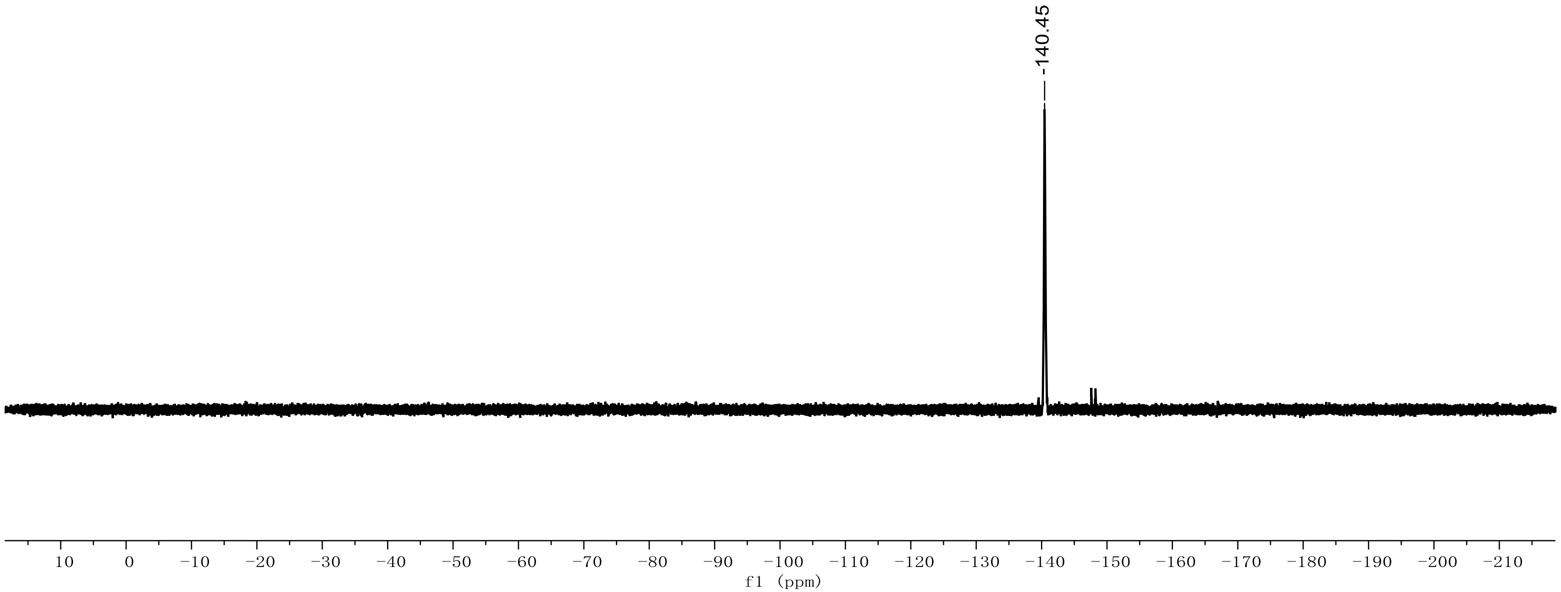

^{11}B NMR of 4m

DMSO-d_6, 128 MHz, 25 °C

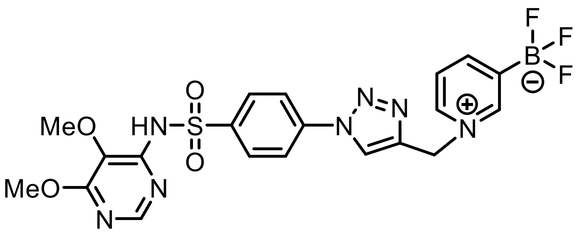

4m

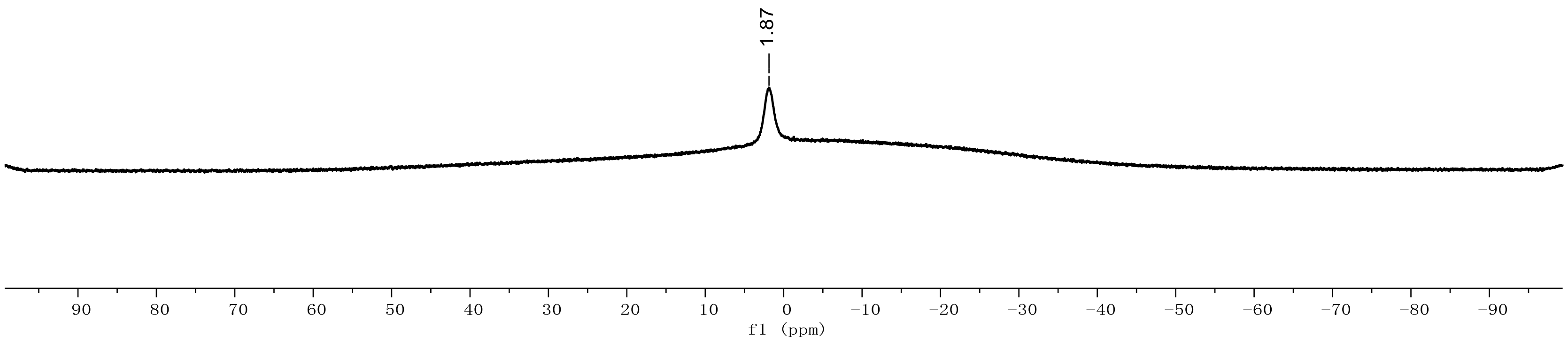

^{1}H NMR of 3n

DMSO-d_6, 400 MHz, 25 °C

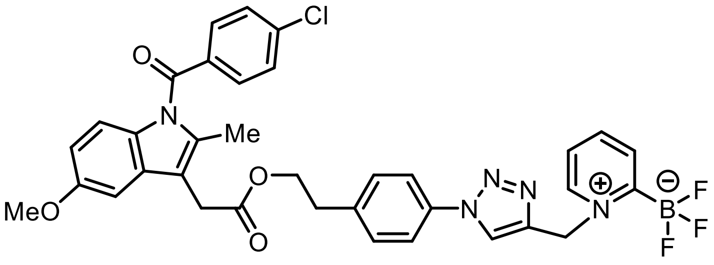

3n

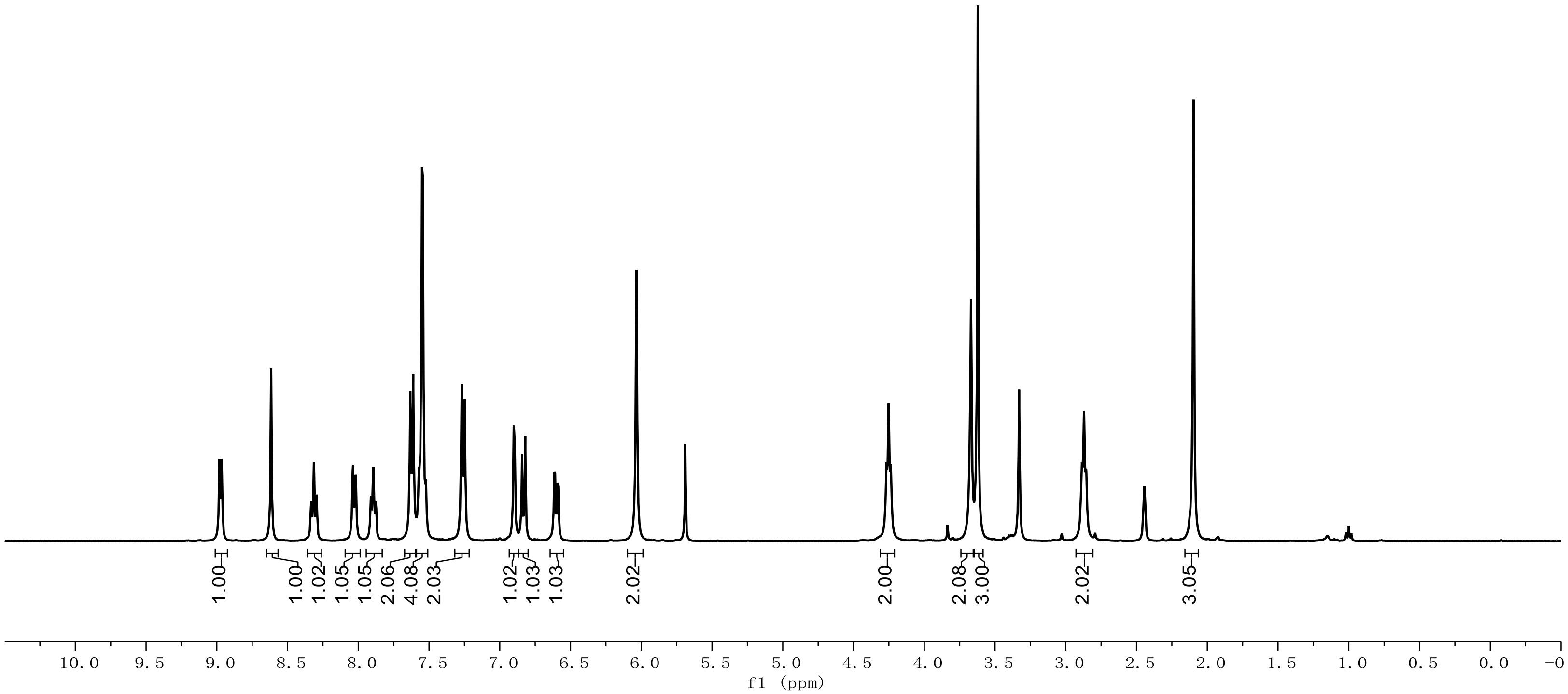

^{13}C NMR of 3n

DMSO-d_6, 101 MHz, 25 °C

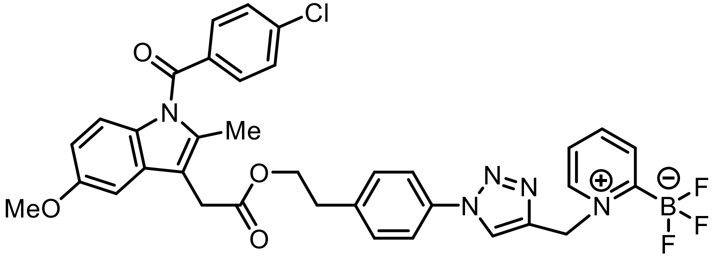

3n

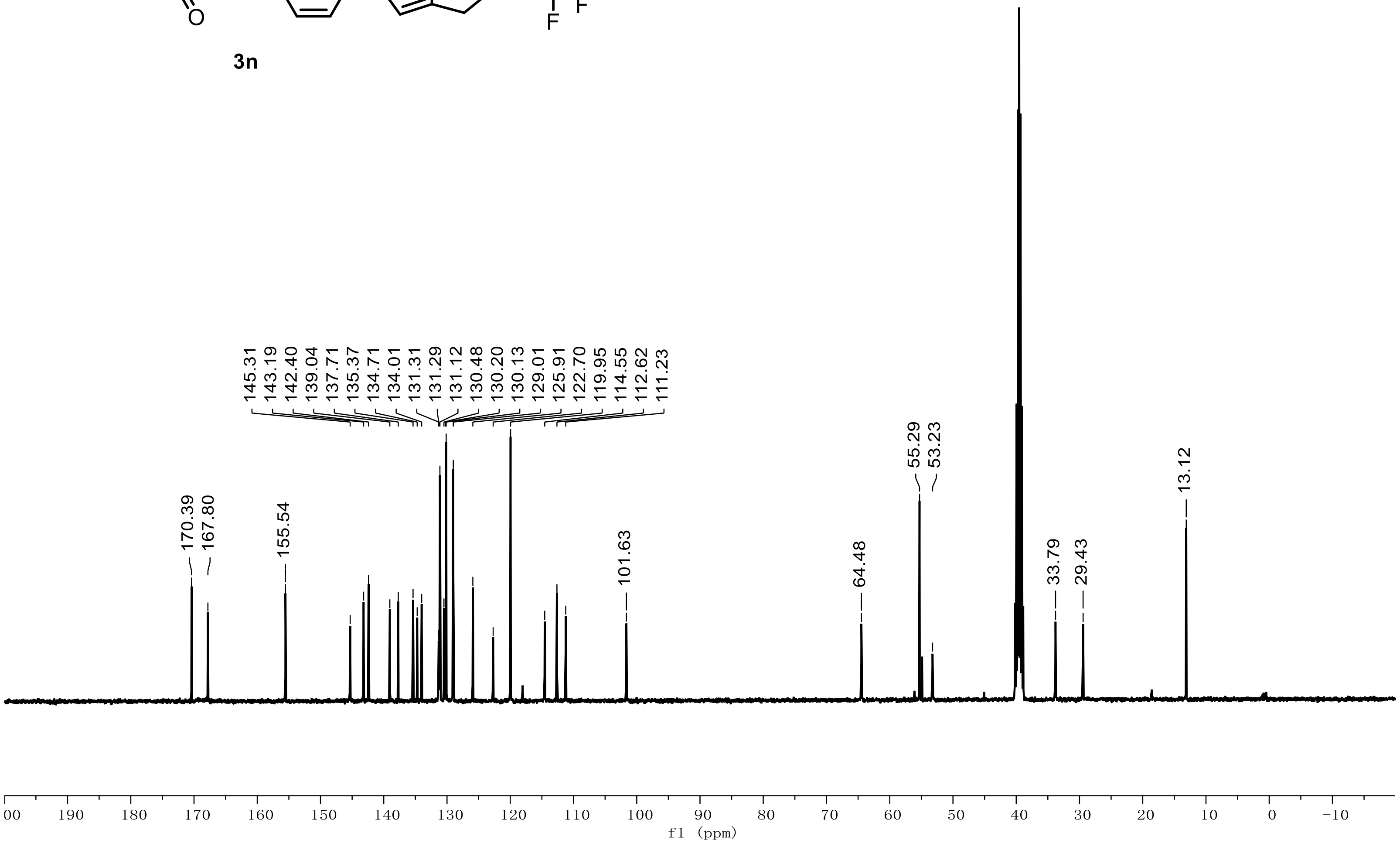

^{19}F NMR of 3n

DMSO-d_6, 376 MHz, 25 °C

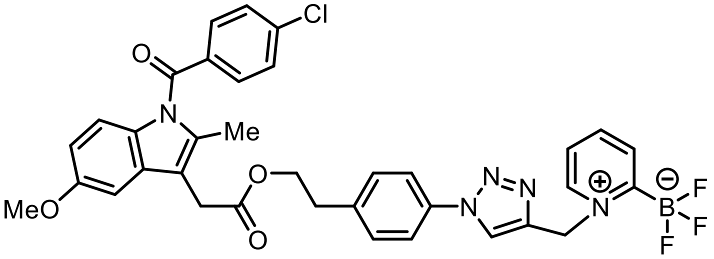

3n

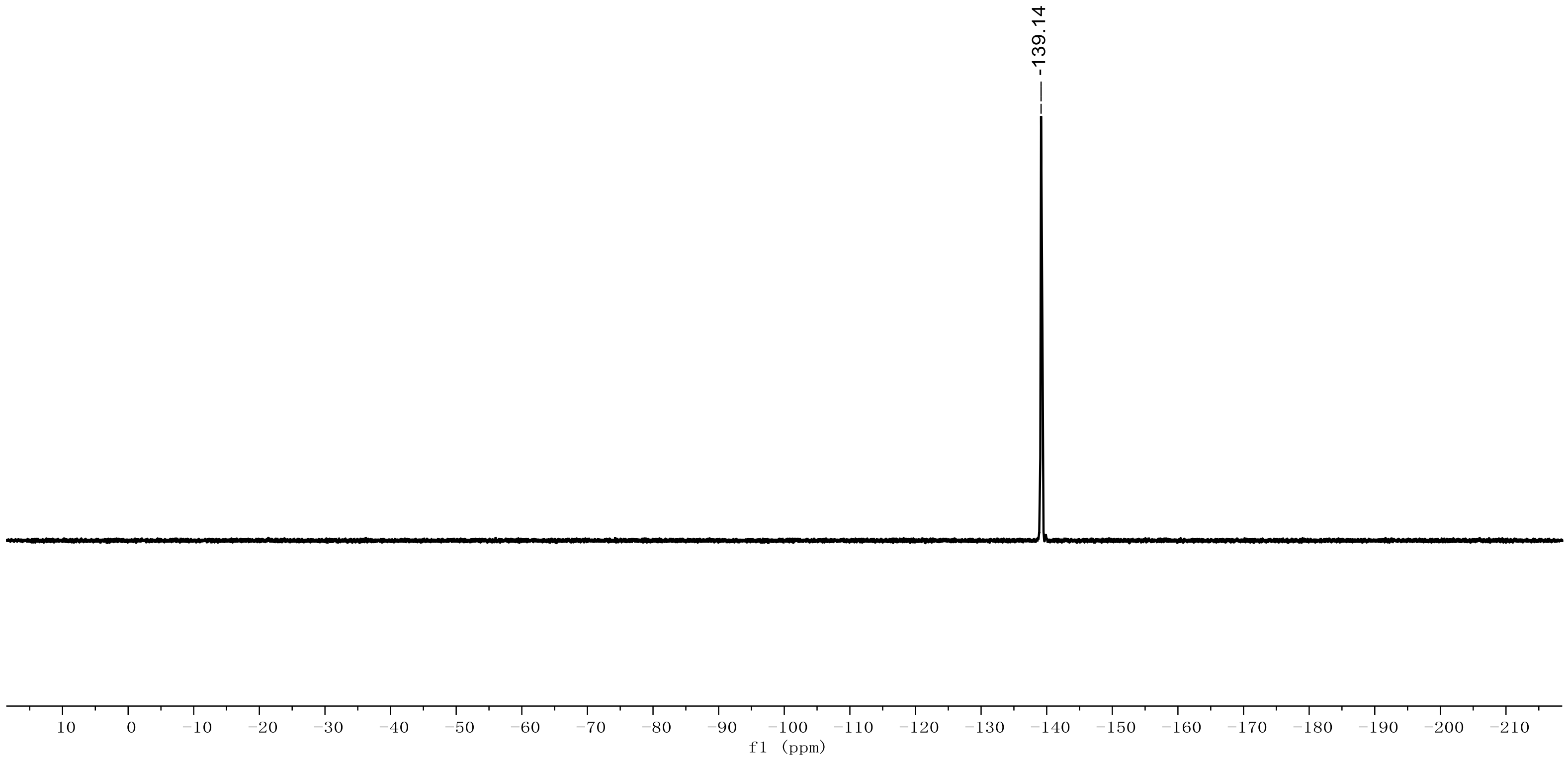

^{11}B NMR of 3n

DMSO-d_6, 128 MHz, 25 °C

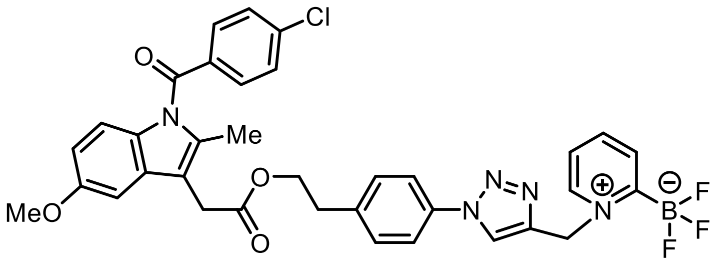

3n

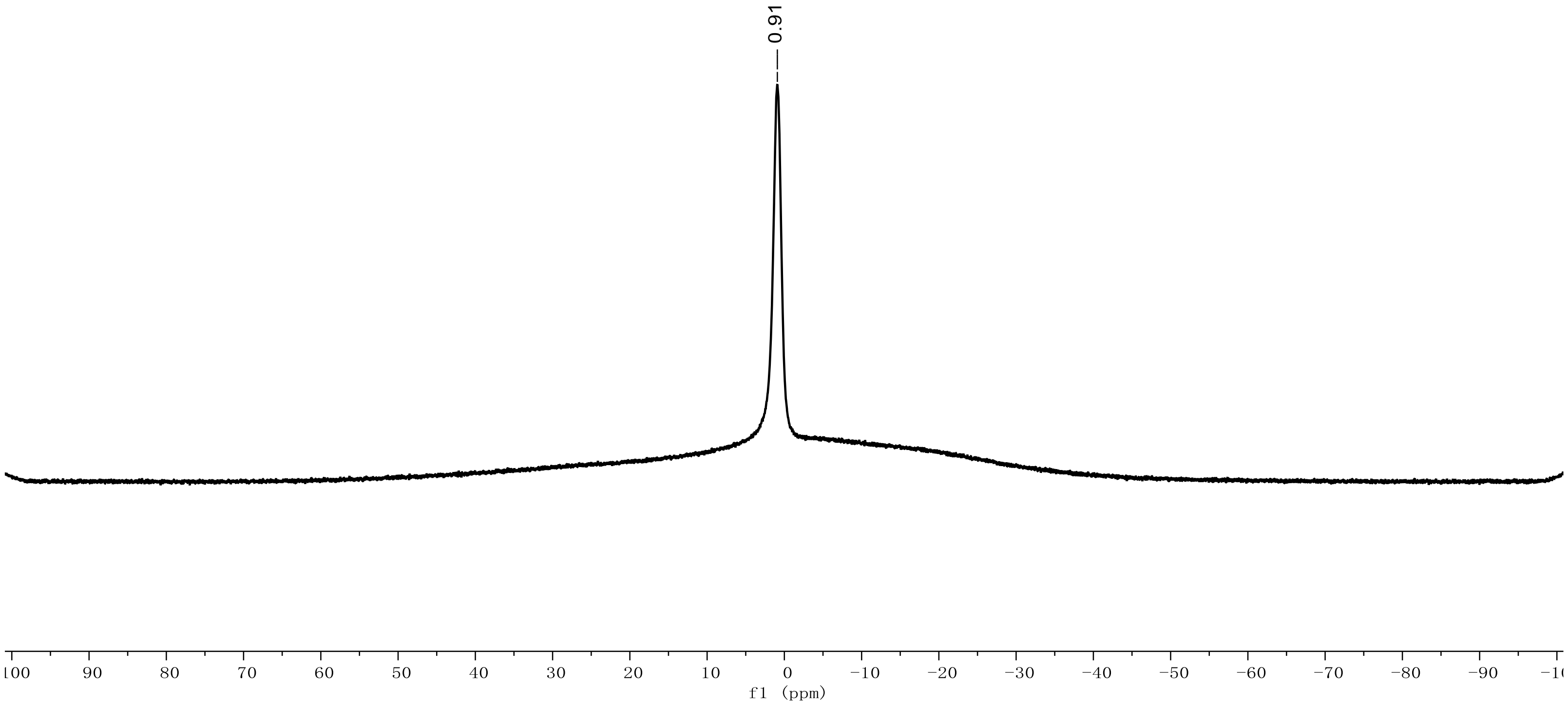

^{1}H NMR of 3o

DMSO-d_6, 400 MHz, 25 °C

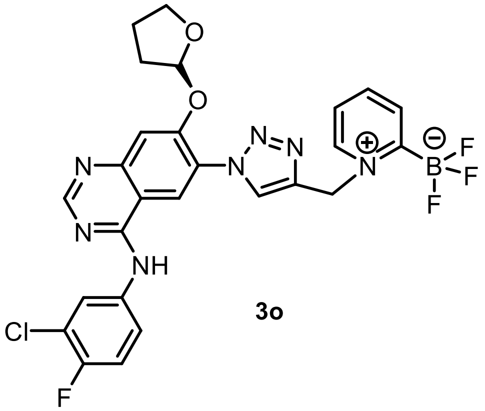

3o

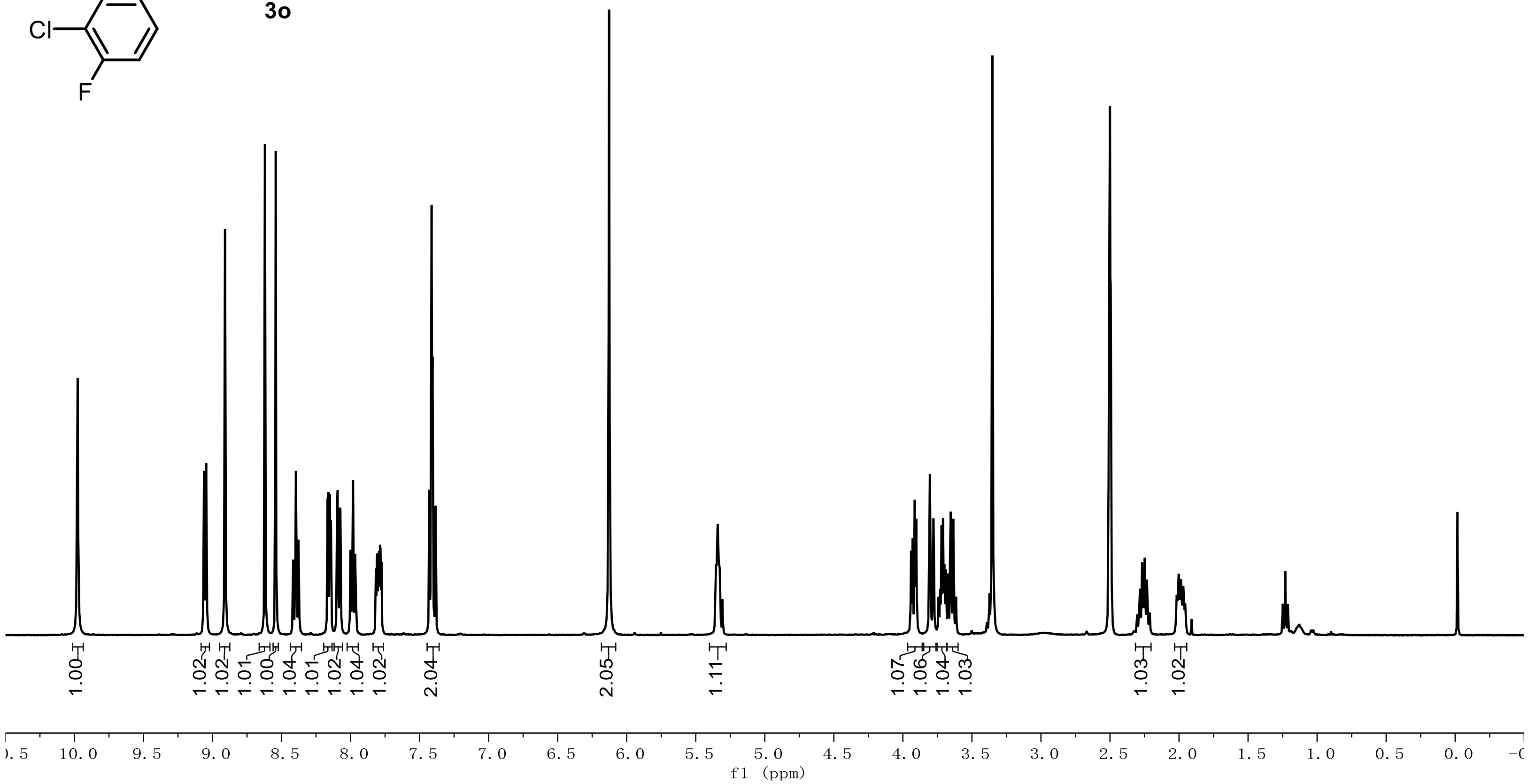

^{13}C NMR of 3o

DMSO-d_6, 101 MHz, 25 °C

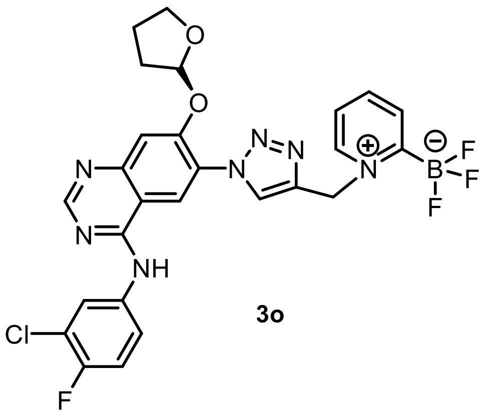

3o

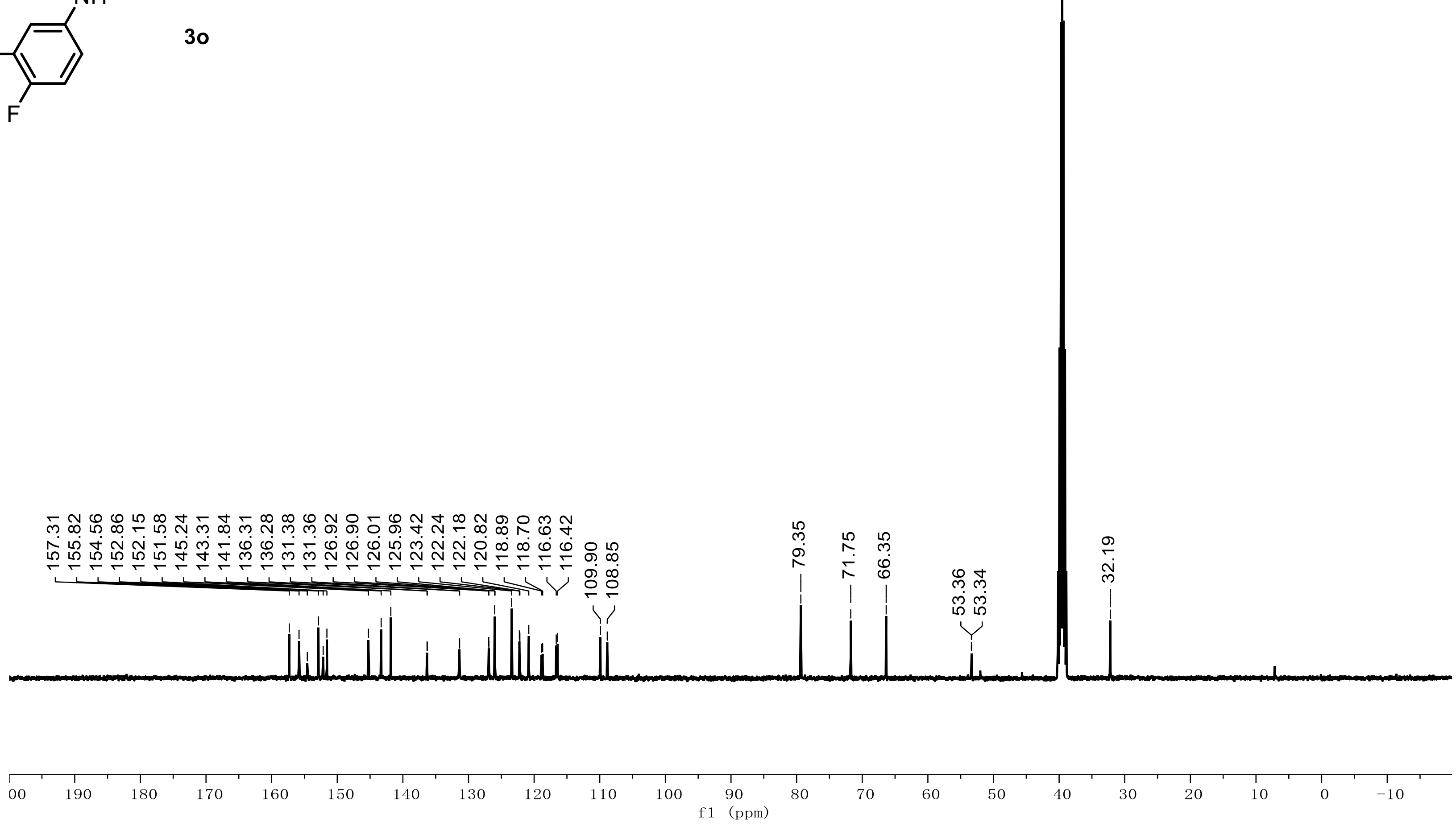

^{19}F NMR of 3o

DMSO-d_6, 376 MHz, 25 °C

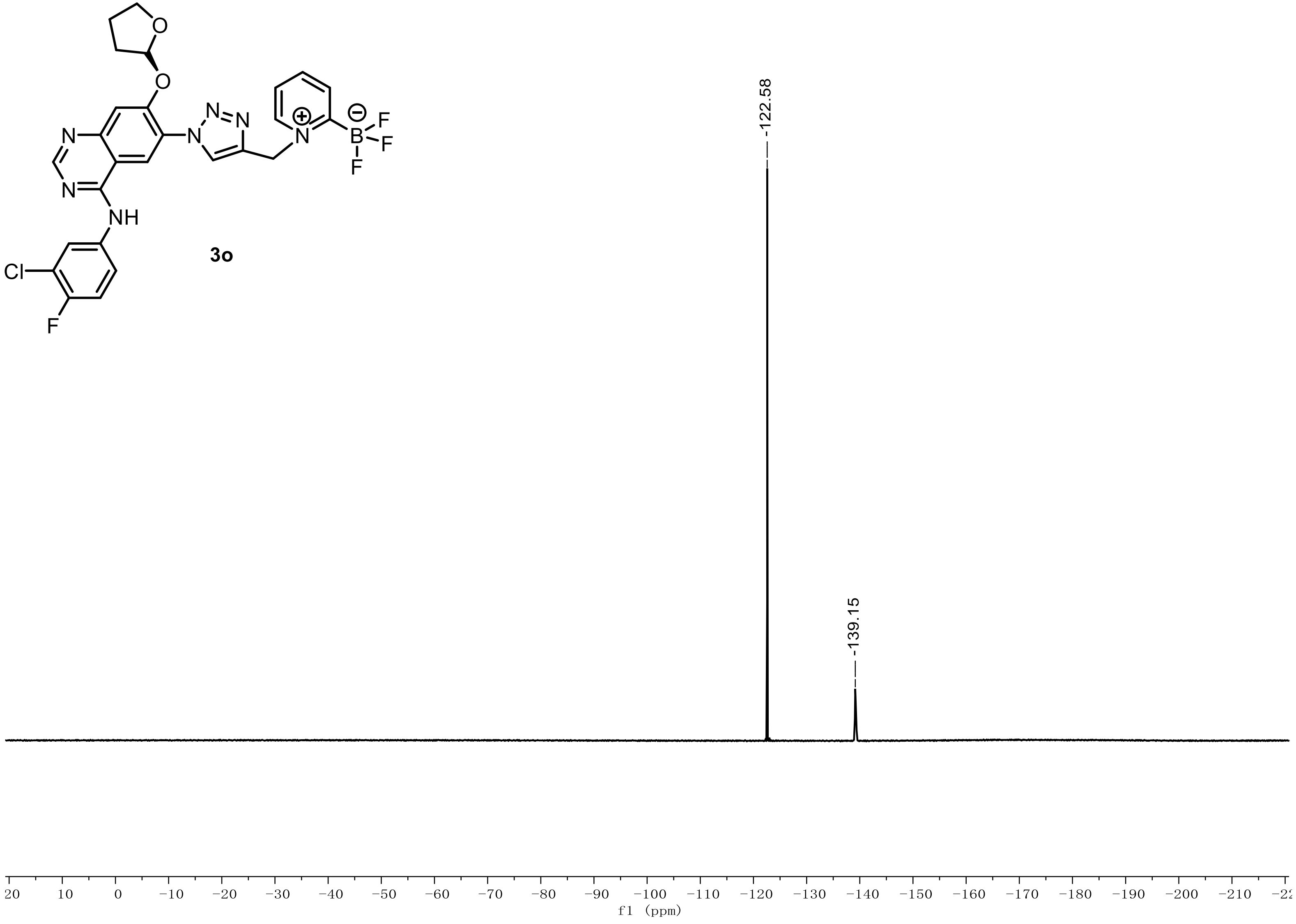

^{11}B NMR of 3o

DMSO-d_6, 128 MHz, 25 °C

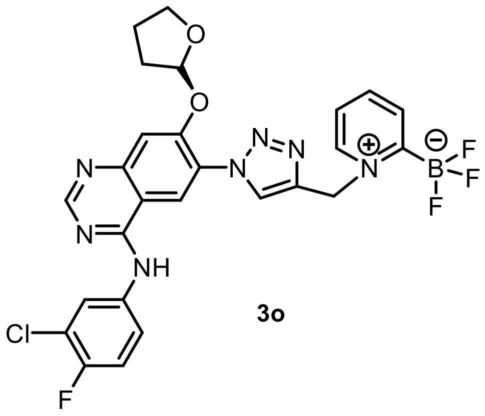

3o

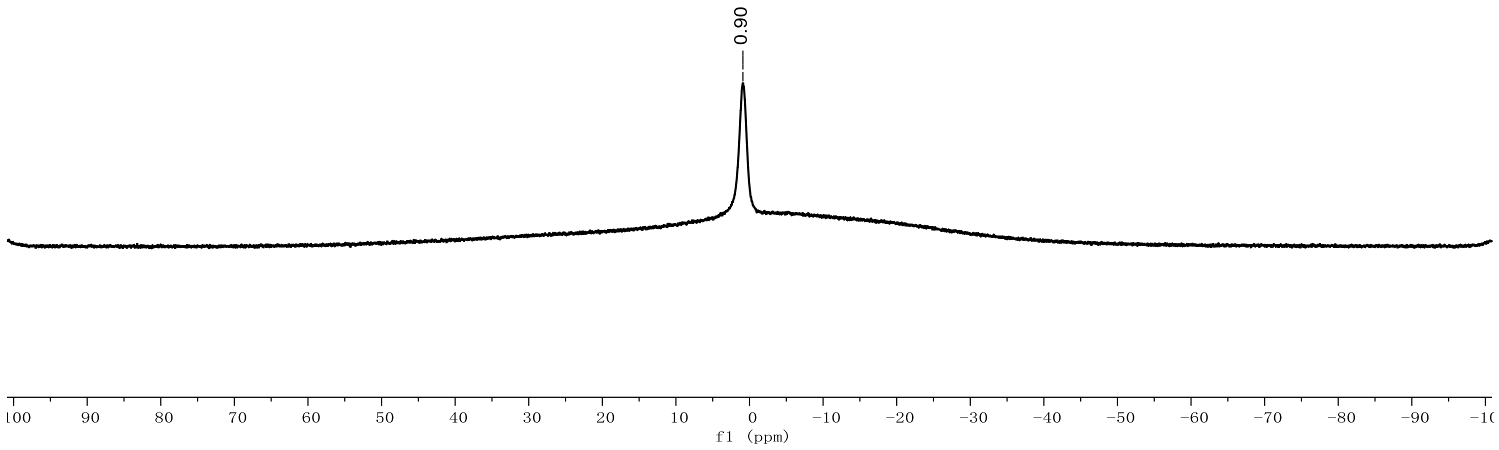

^1H NMR of 5p

CD_3CN, 400 MHz, 25 °C

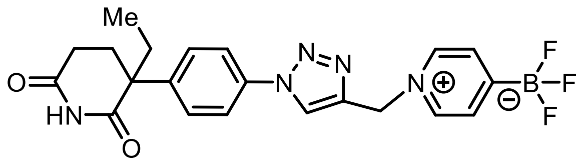

5p

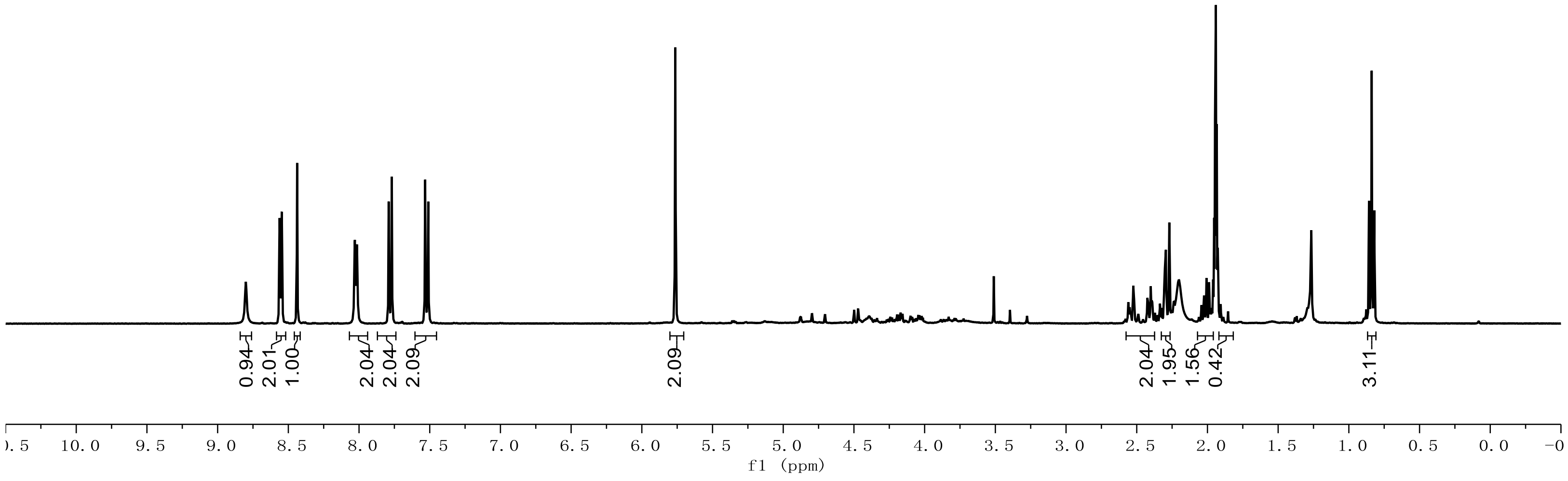

^{13}C NMR of 5p

CD_3CN, 101 MHz, 25 °C

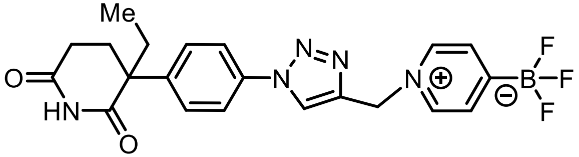

5p

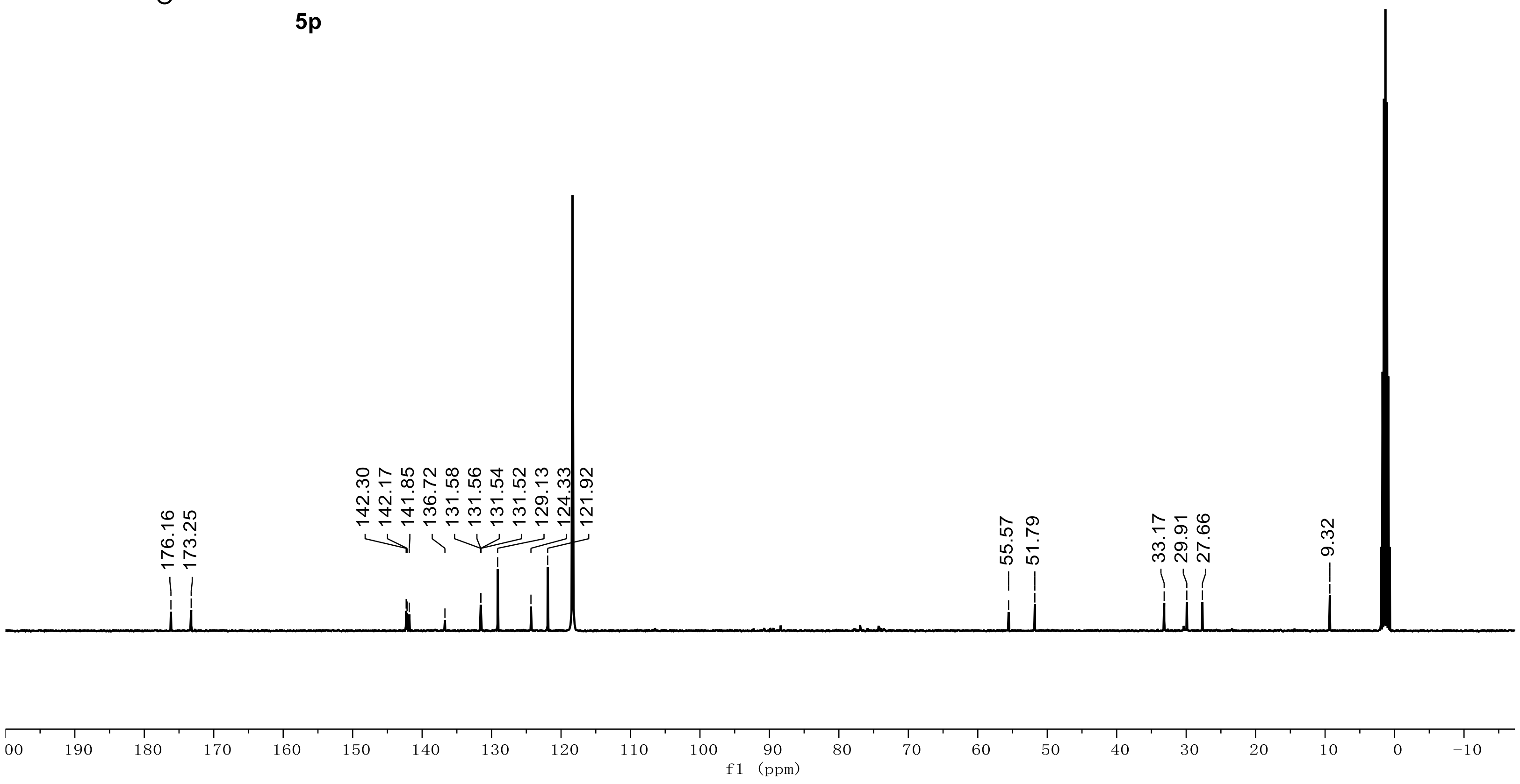

^{19}F NMR of 5p

CD_3CN, 376 MHz, 25 °C

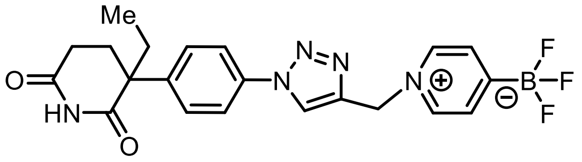

5p

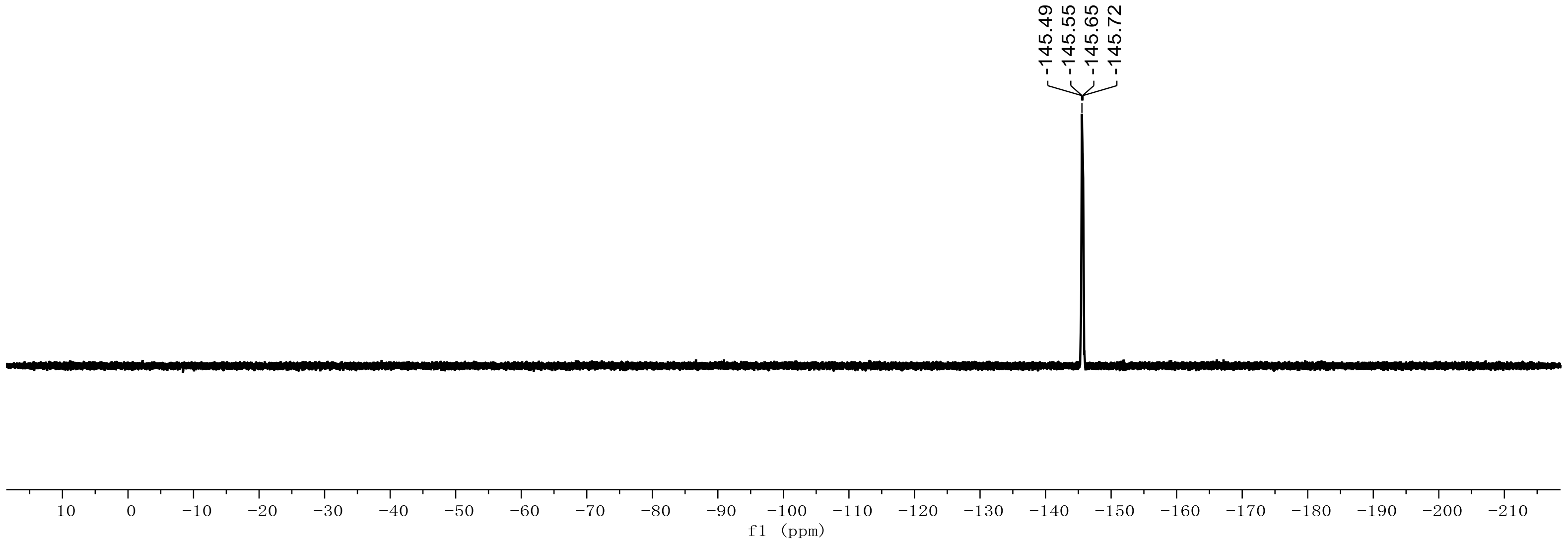

^{11}B NMR of 5p

CD_3CN, 128 MHz, 25 °C

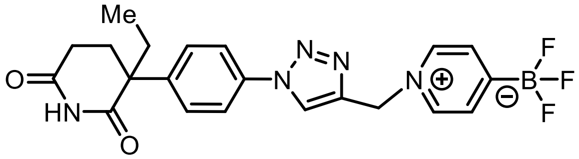

5p

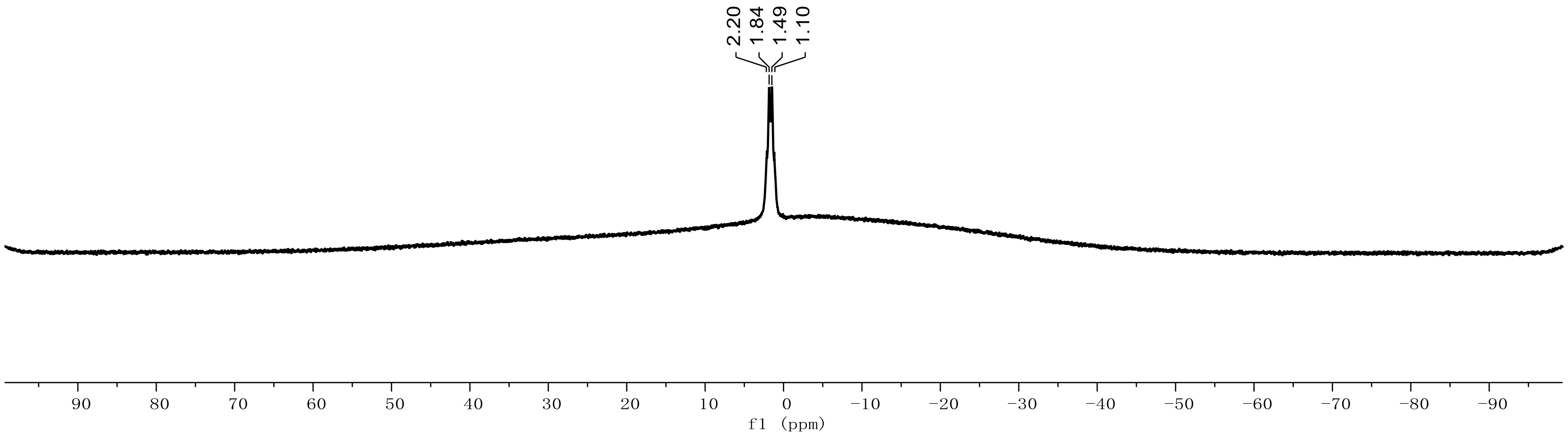

¹H NMR of 3q

DMSO-d_6, 400 MHz, 25 °C

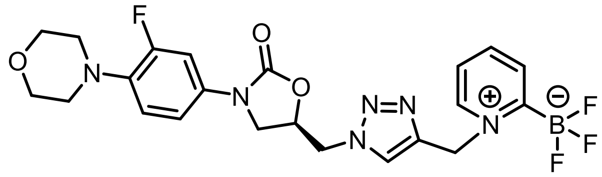

3q

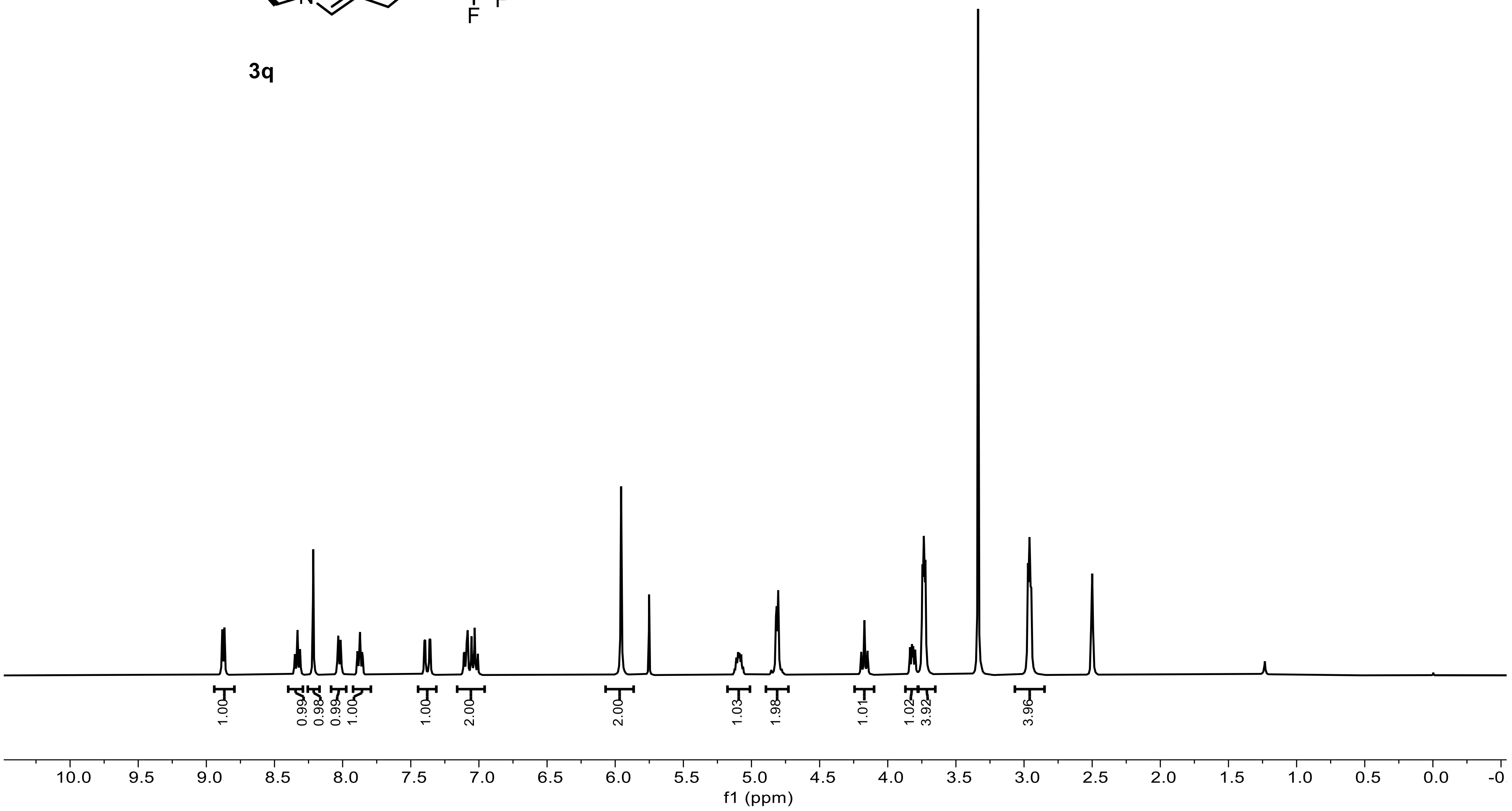

^{13}C NMR of 3q

DMSO-d_6, 101 MHz, 25 °C

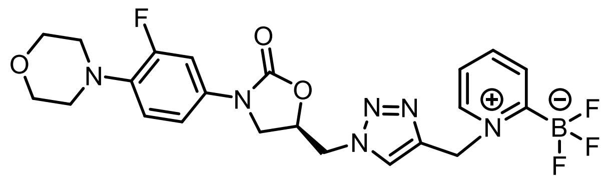

3q

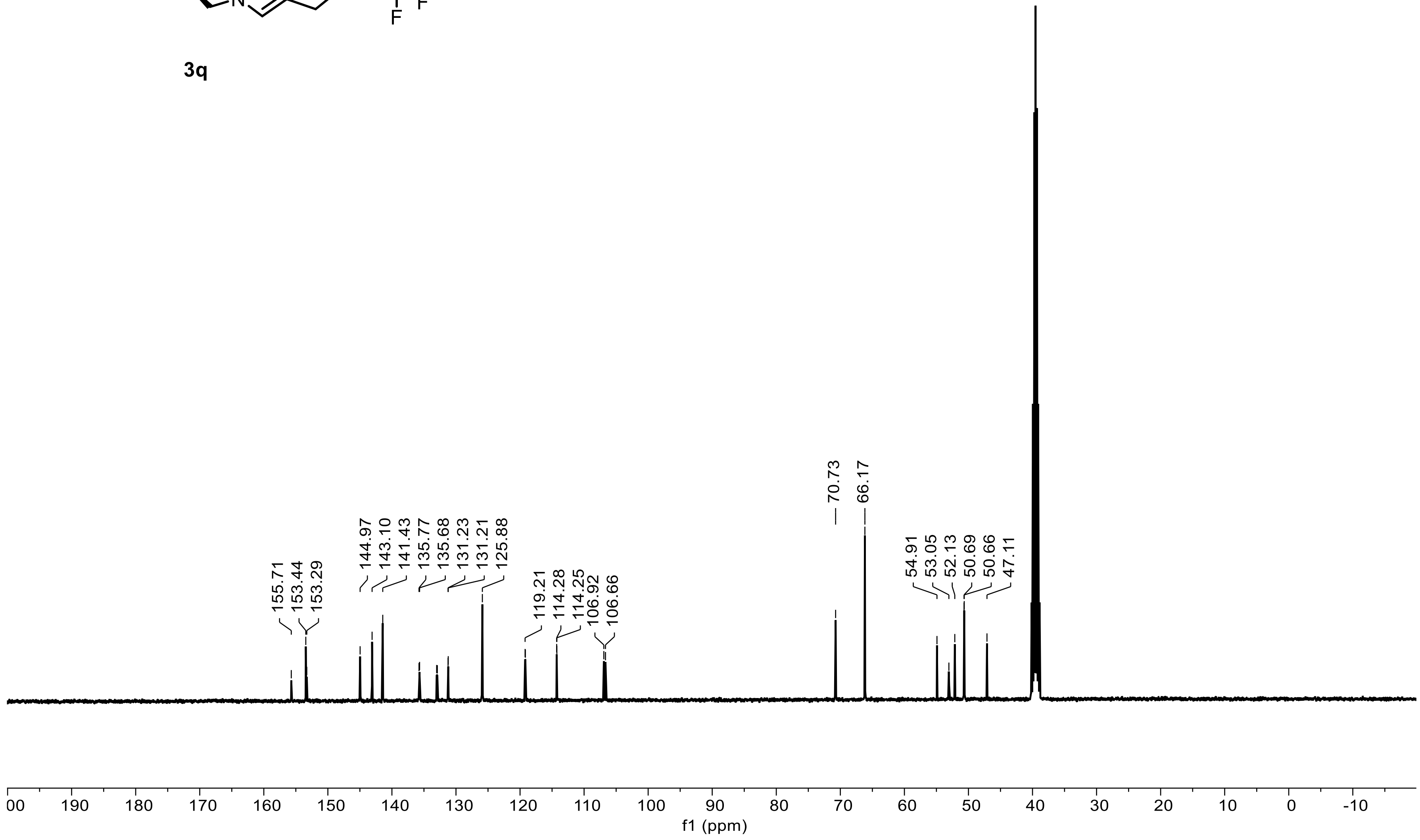

^{19}F NMR of 3q

DMSO-d_6, 376 MHz, 25 °C

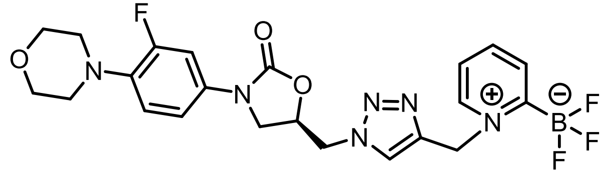

3q

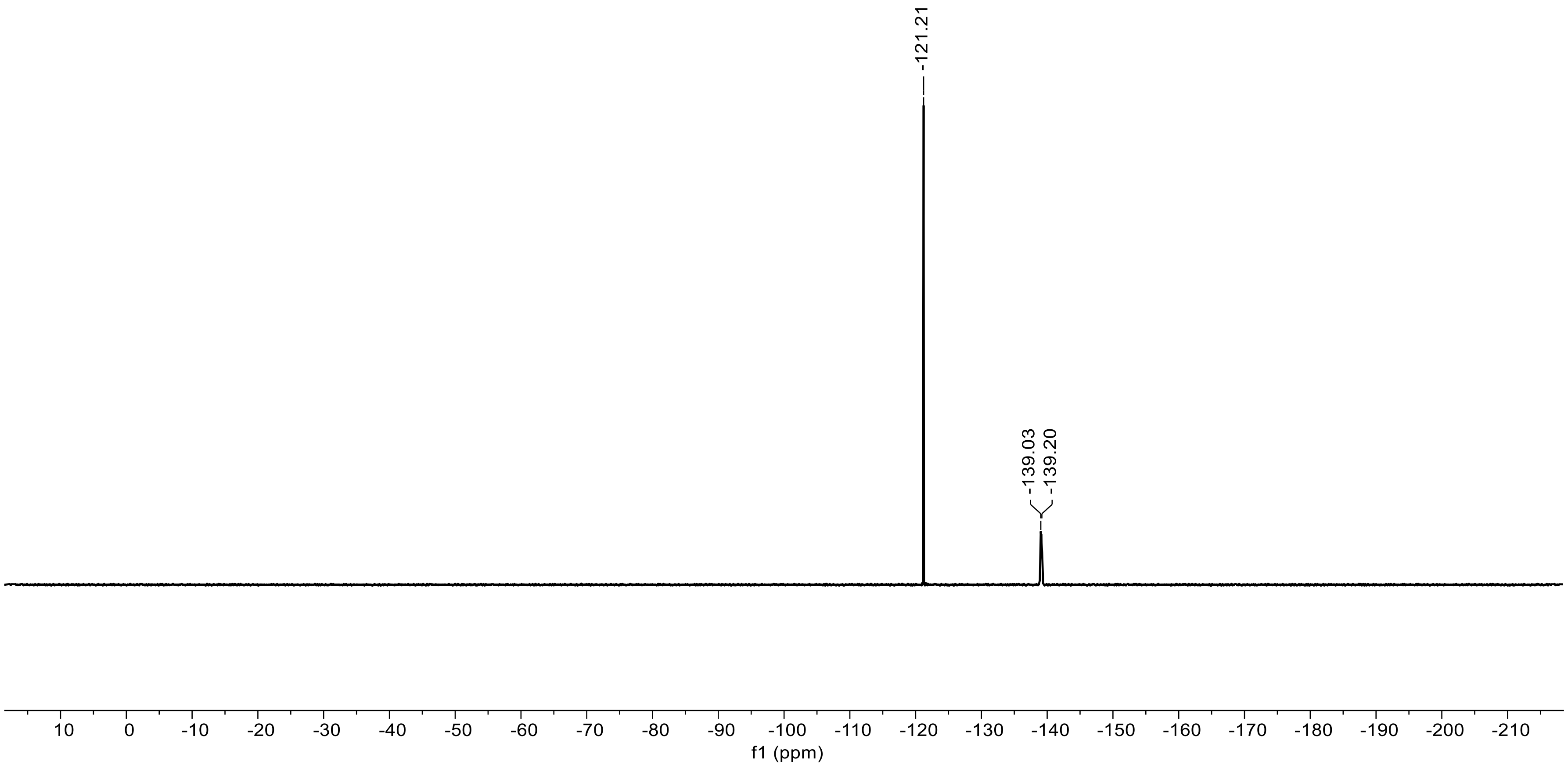

^{11}B NMR of 3q

DMSO-d_6, 128 MHz, 25 °C

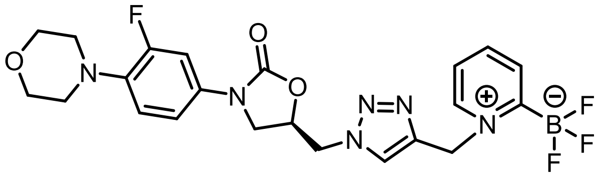

3q

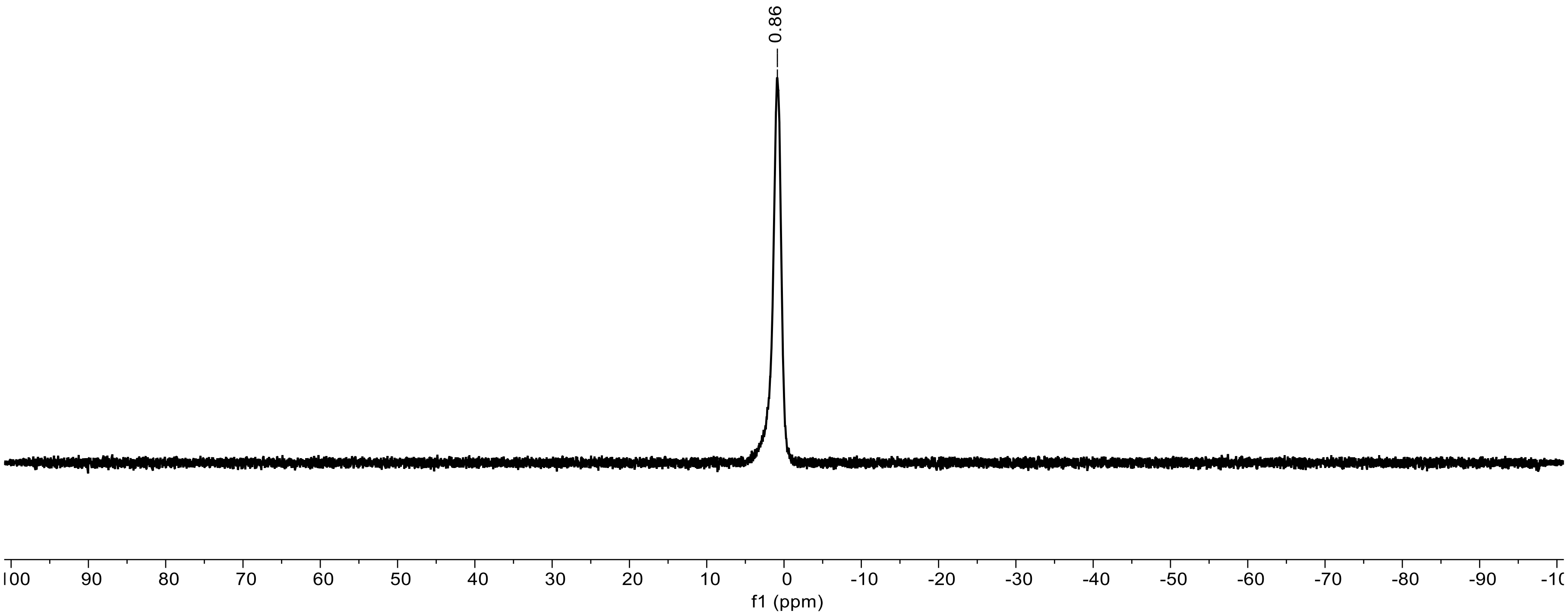

^{1}H NMR of 3r

DMSO-d_6, 400 MHz, 25 °C

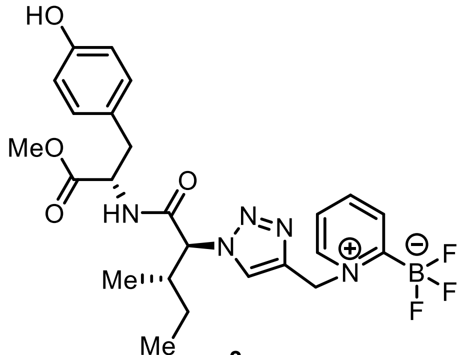

3r

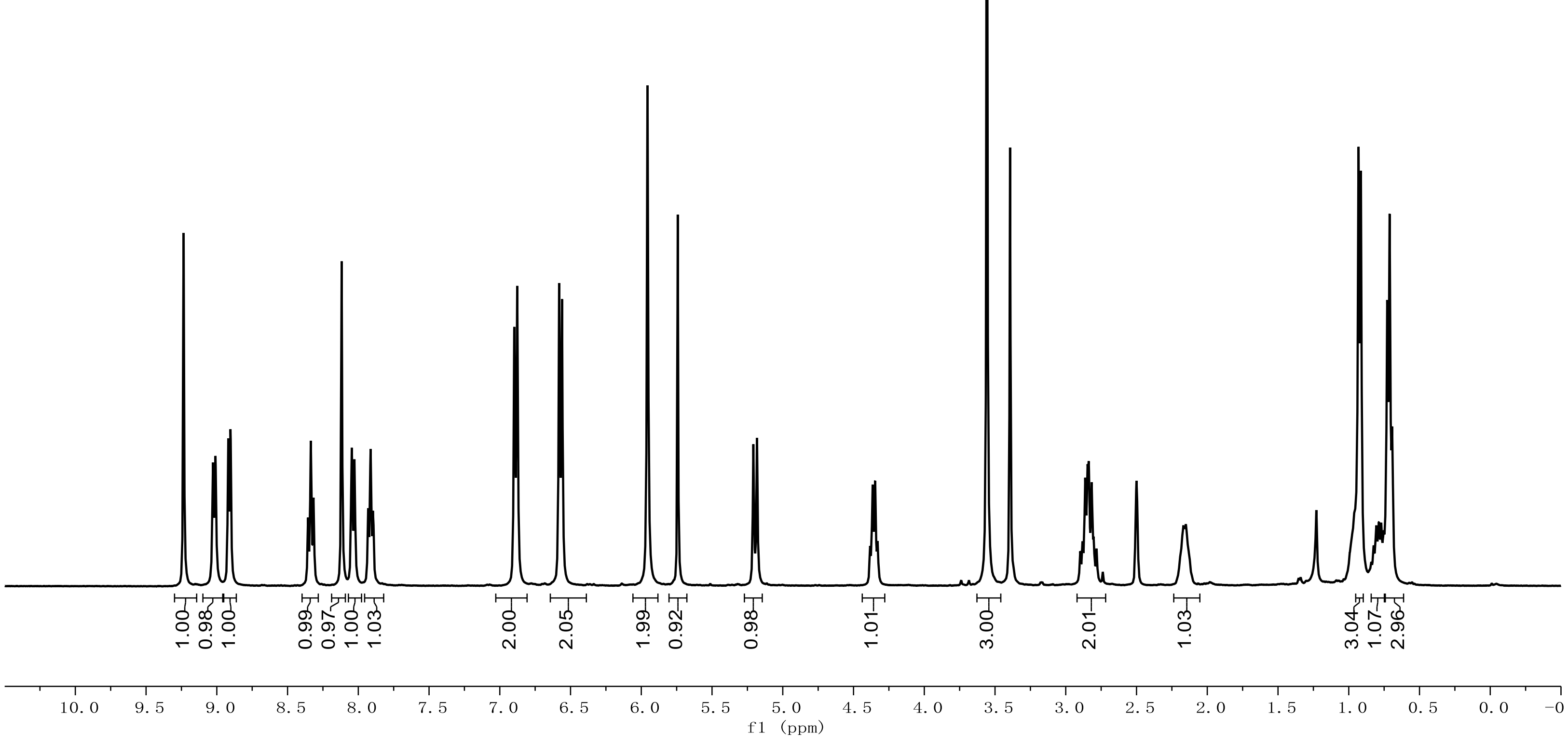

^{13}C NMR of 3r

DMSO-d_6, 101 MHz, 25 °C

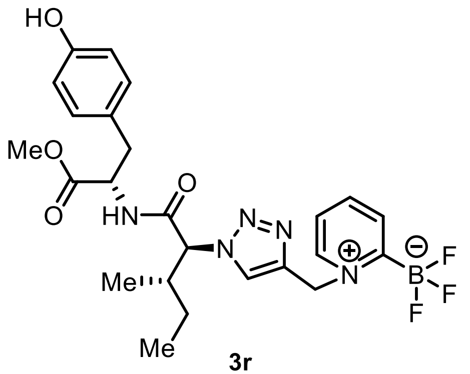

3r

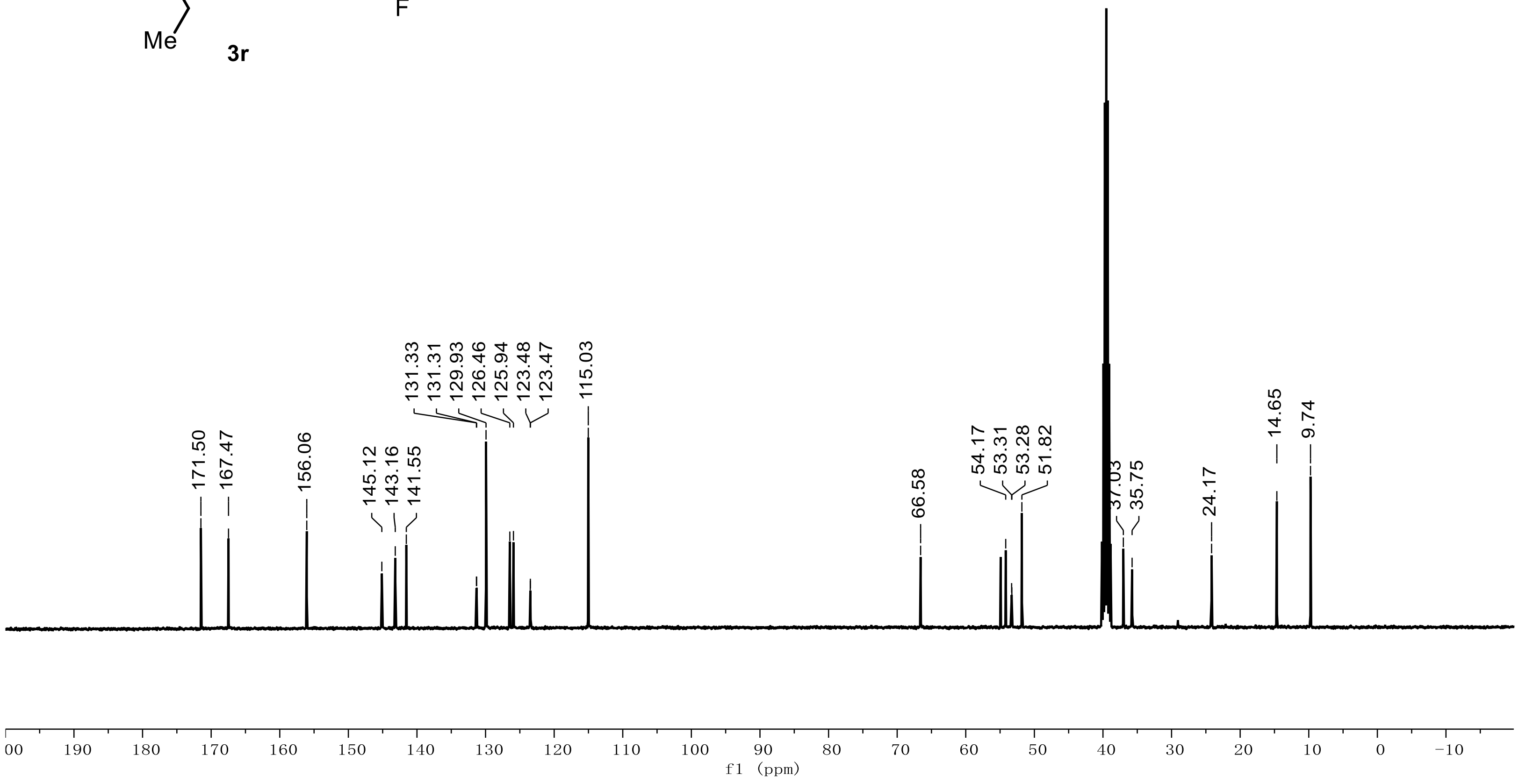

^{19}F NMR of 3r

DMSO-d_6, 376 MHz, 25 °C

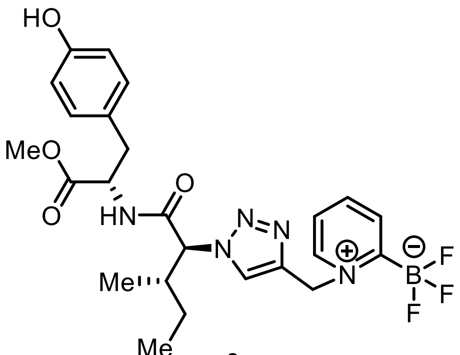

3r

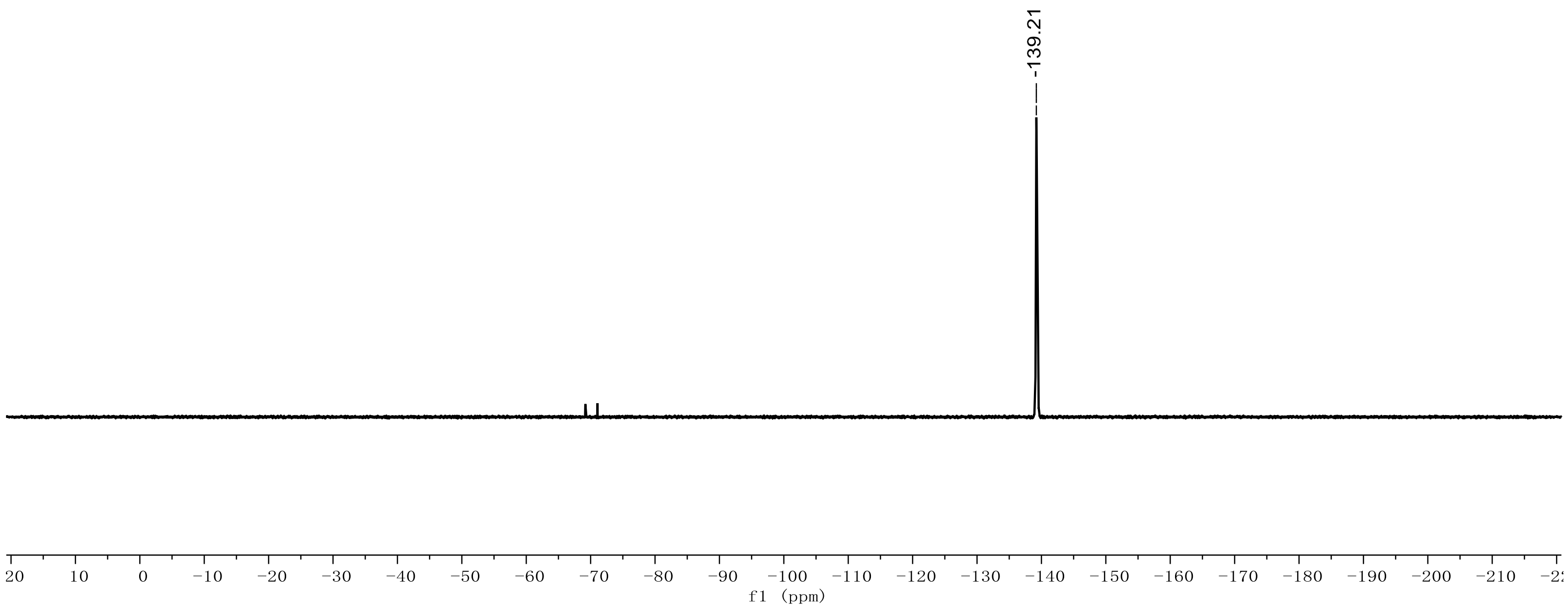

^{11}B NMR of 3r

DMSO-d_6, 128 MHz, 25 °C

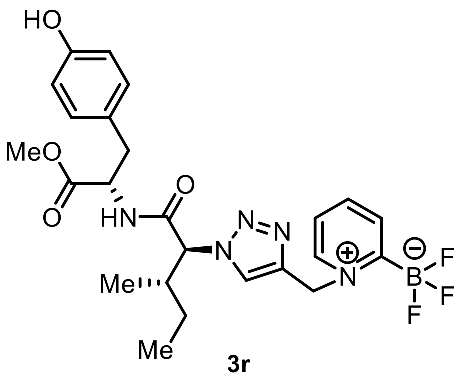

3r

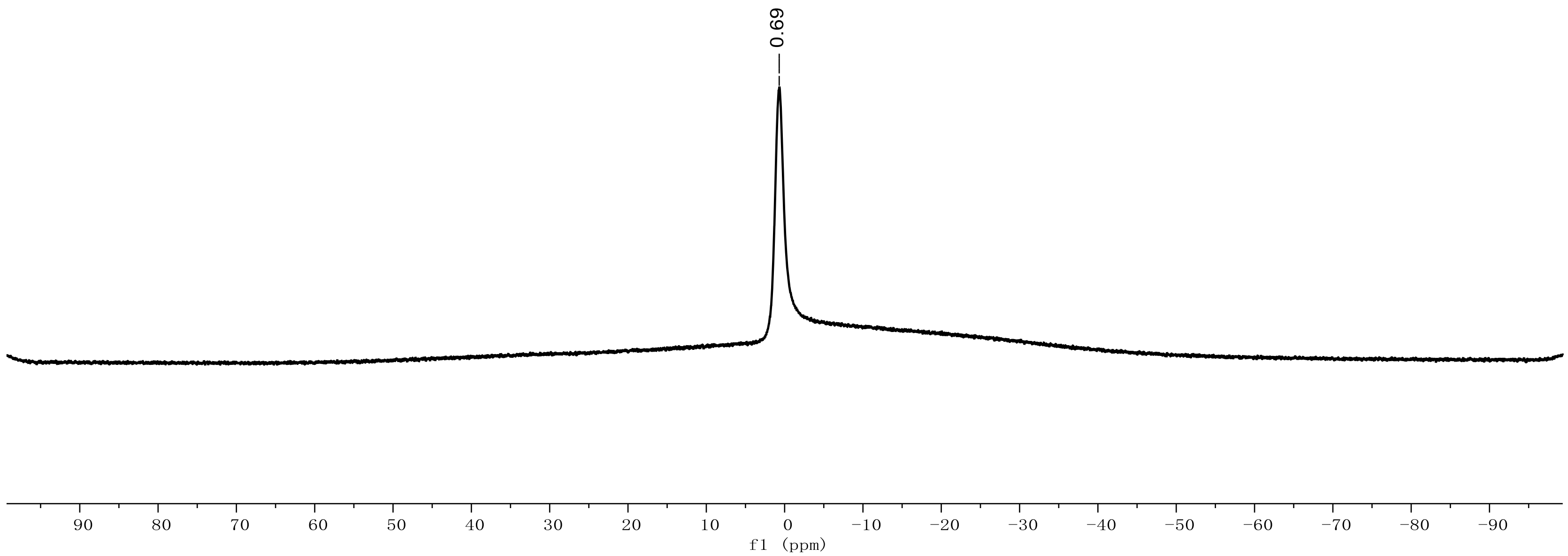

^{1}H NMR of 4s

DMSO-d_6, 400 MHz, 25 °C

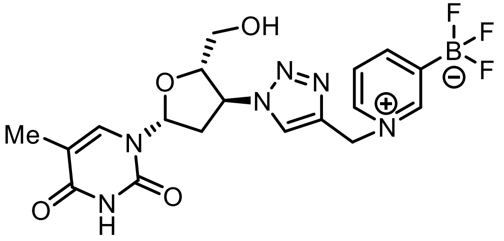

4s

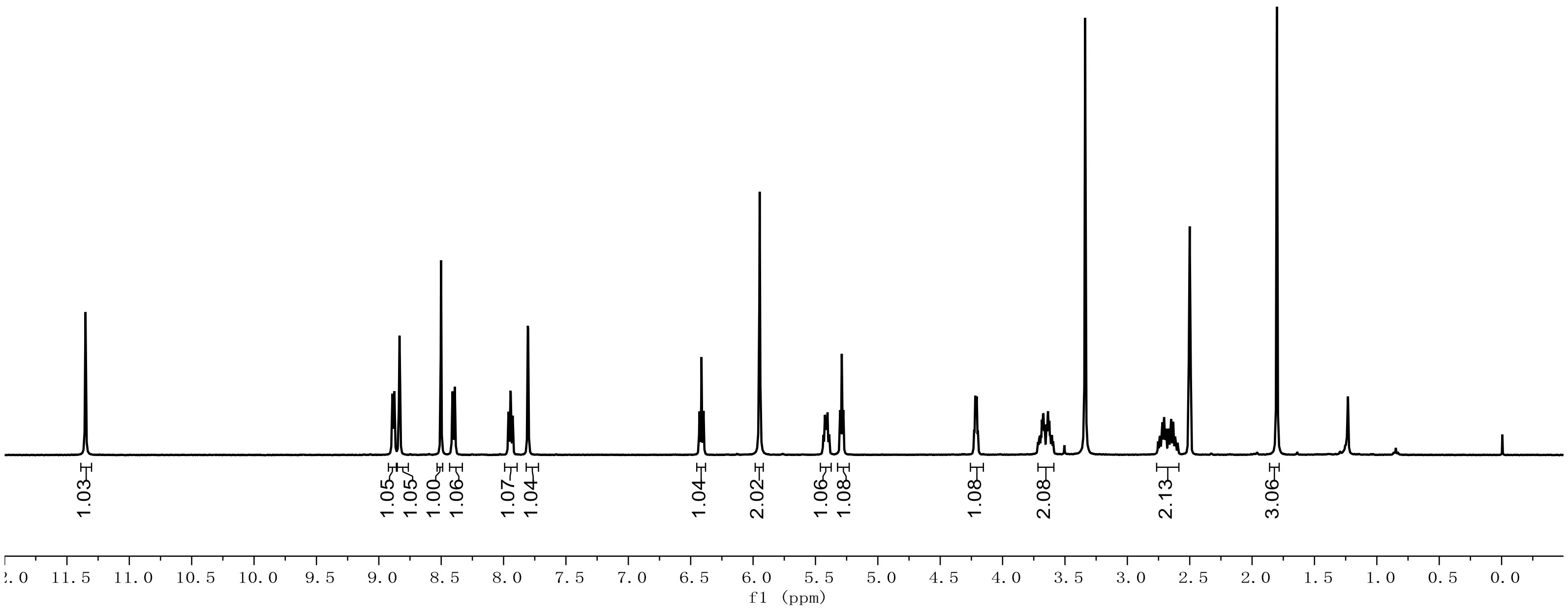

^{13}C NMR of 4s

$CDCl_3$, 101 MHz, 25 °C

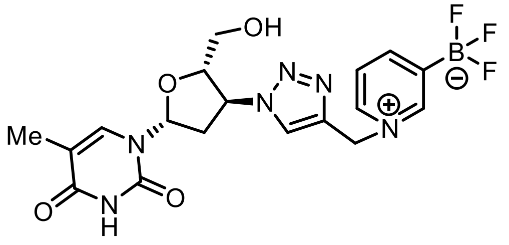

4s

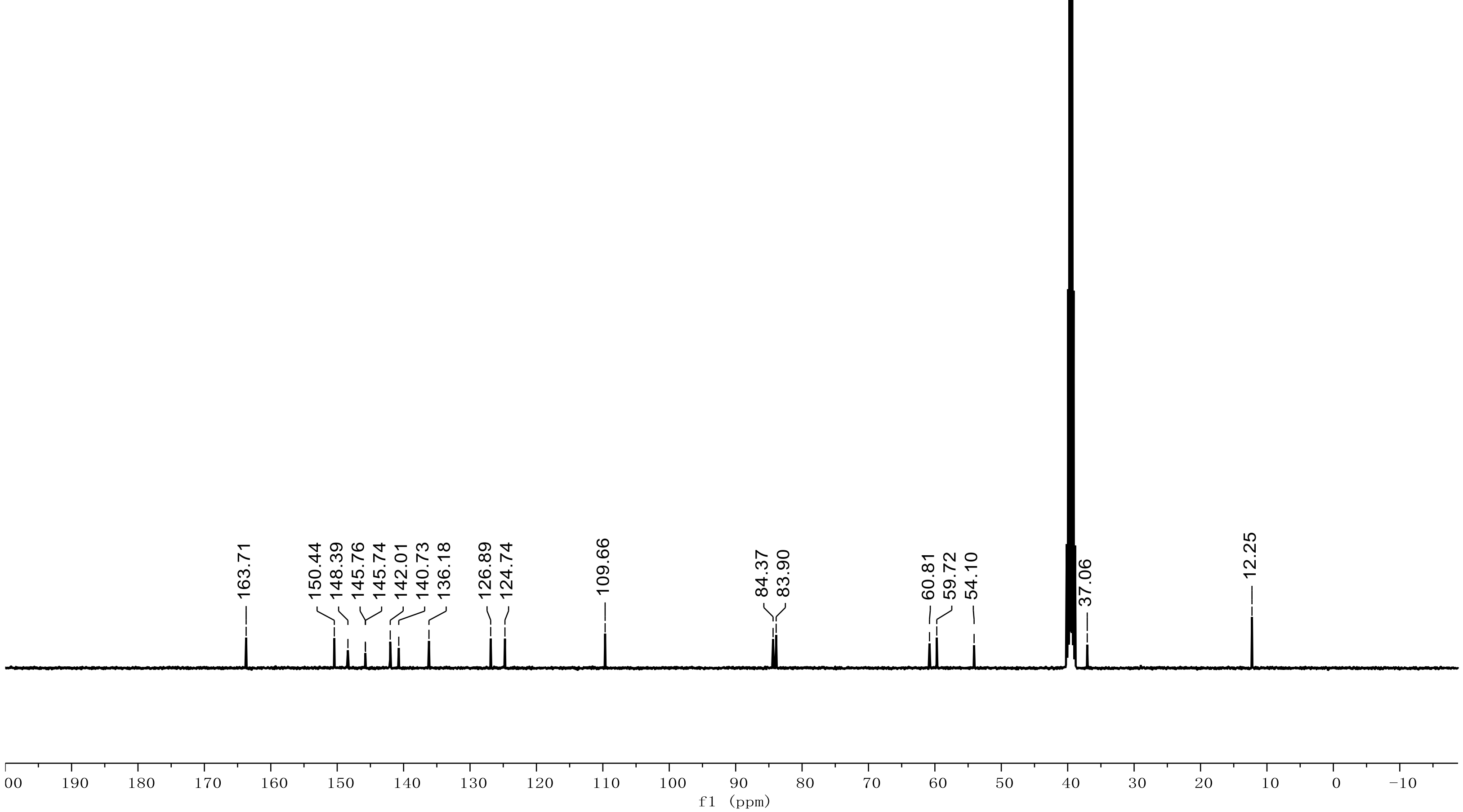

^{19}F NMR of 4s

DMSO-d_6, 376 MHz, 25 °C

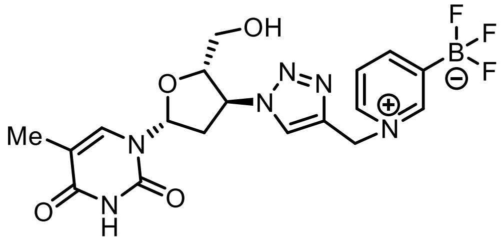

4s

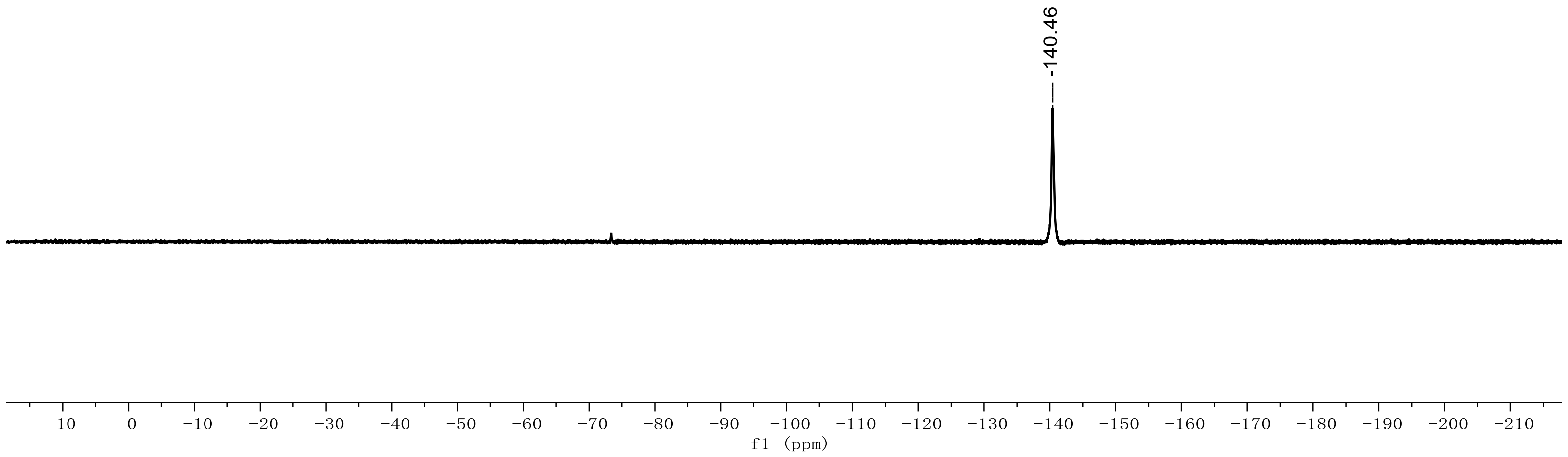

^{11}B NMR of 4s

DMSO-d_6, 128 MHz, 25 °C

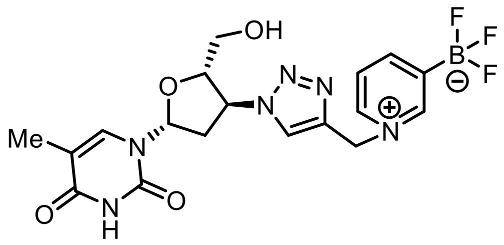

4s

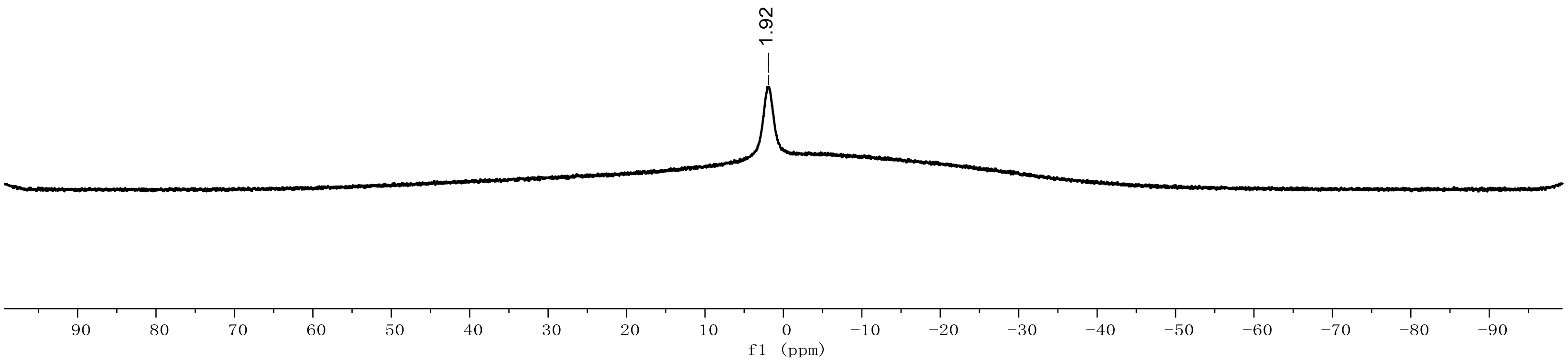

^{1}H NMR of 3t

CD$_3$CN, 400 MHz, 25 °C

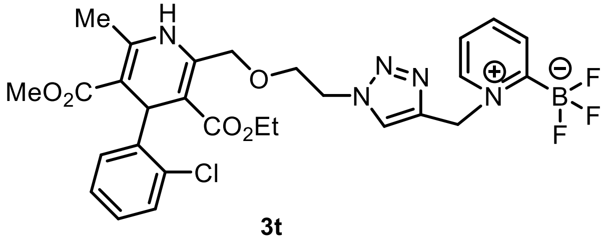

3t

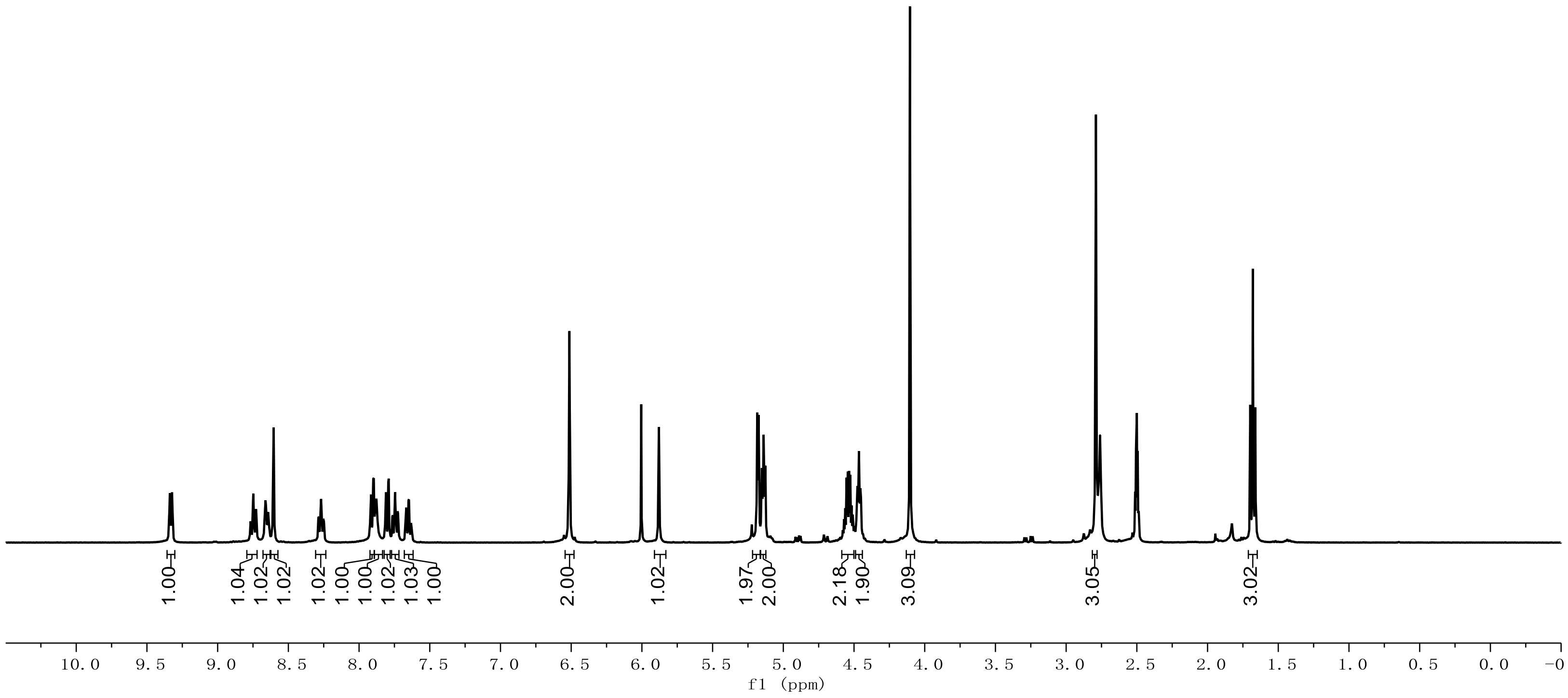

^{13}C NMR of 3t

CD_3CN, 101 MHz, 25 °C

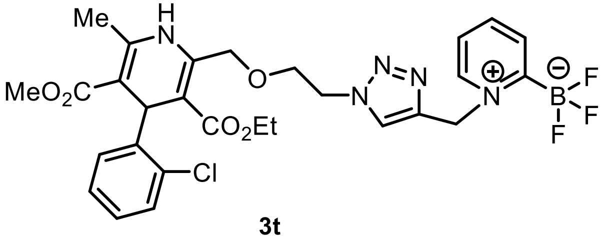

3t

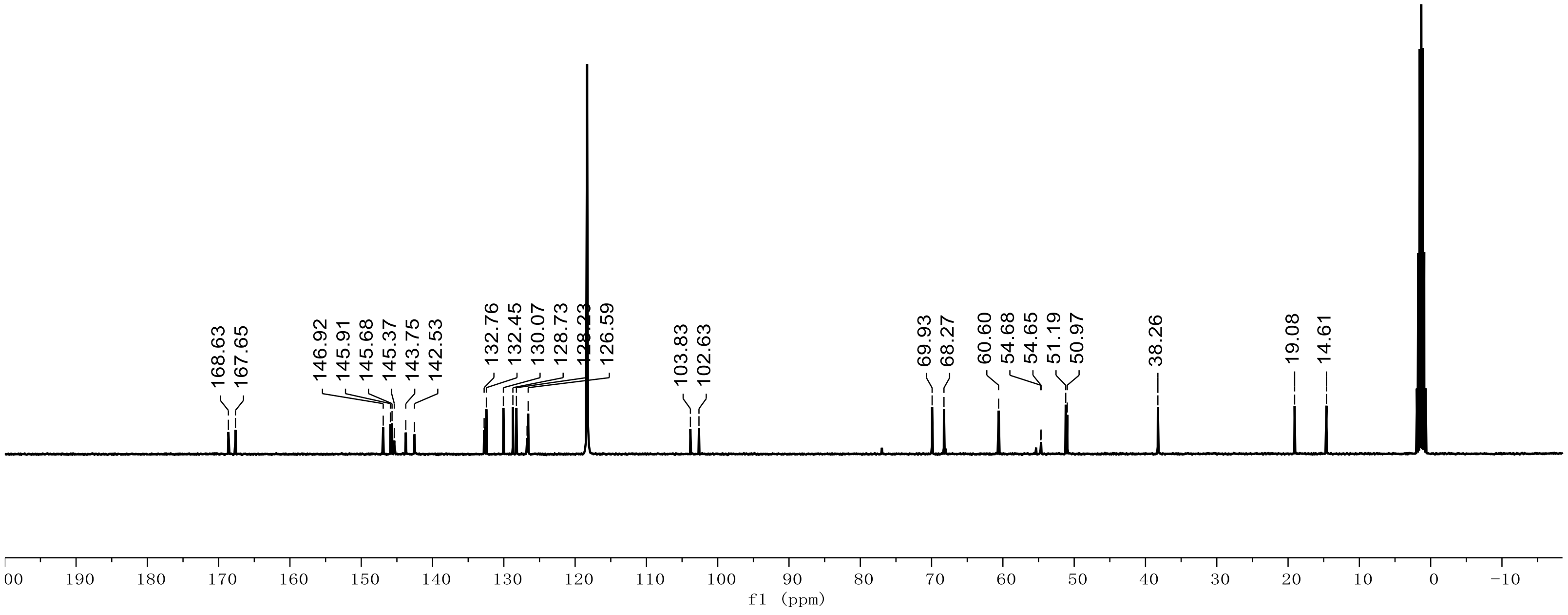

^{19}F NMR of 3t

CD_3CN, 376 MHz, 25 °C

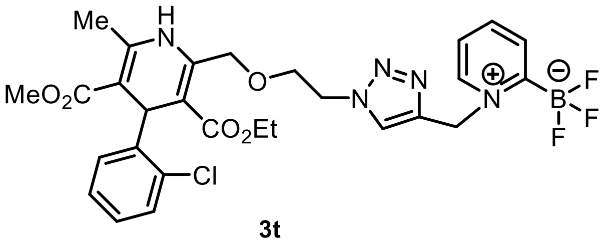

3t

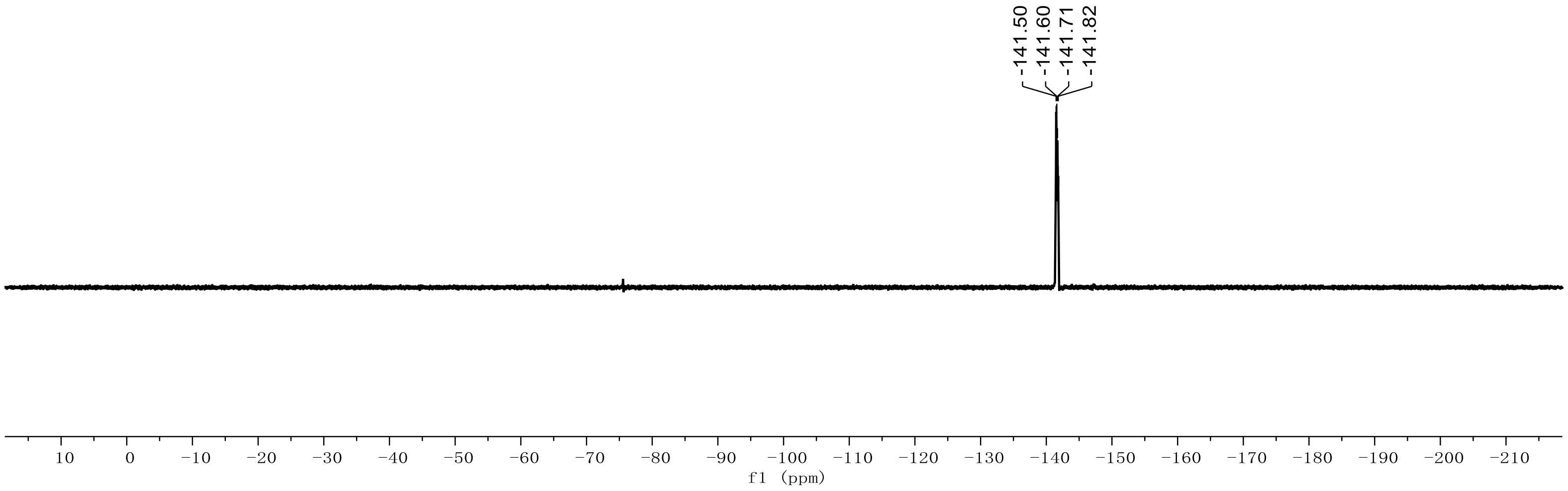

^{11}B NMR of 3t

CD_3CN, 128 MHz, 25 °C

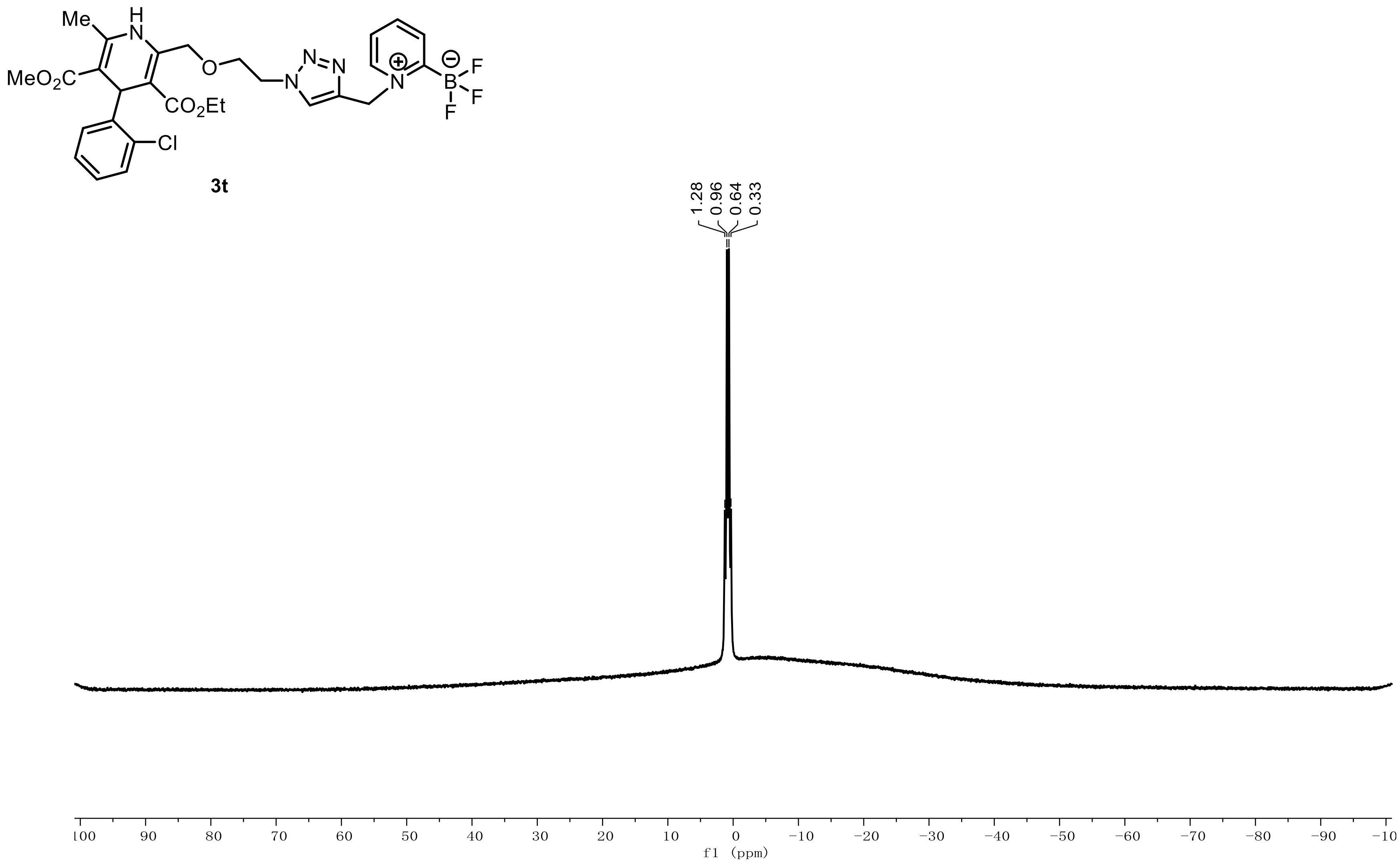

^{1}H NMR of 4u

CD_3CN, 400 MHz, 25 °C

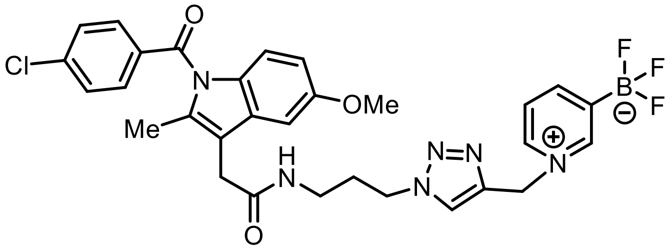

4u

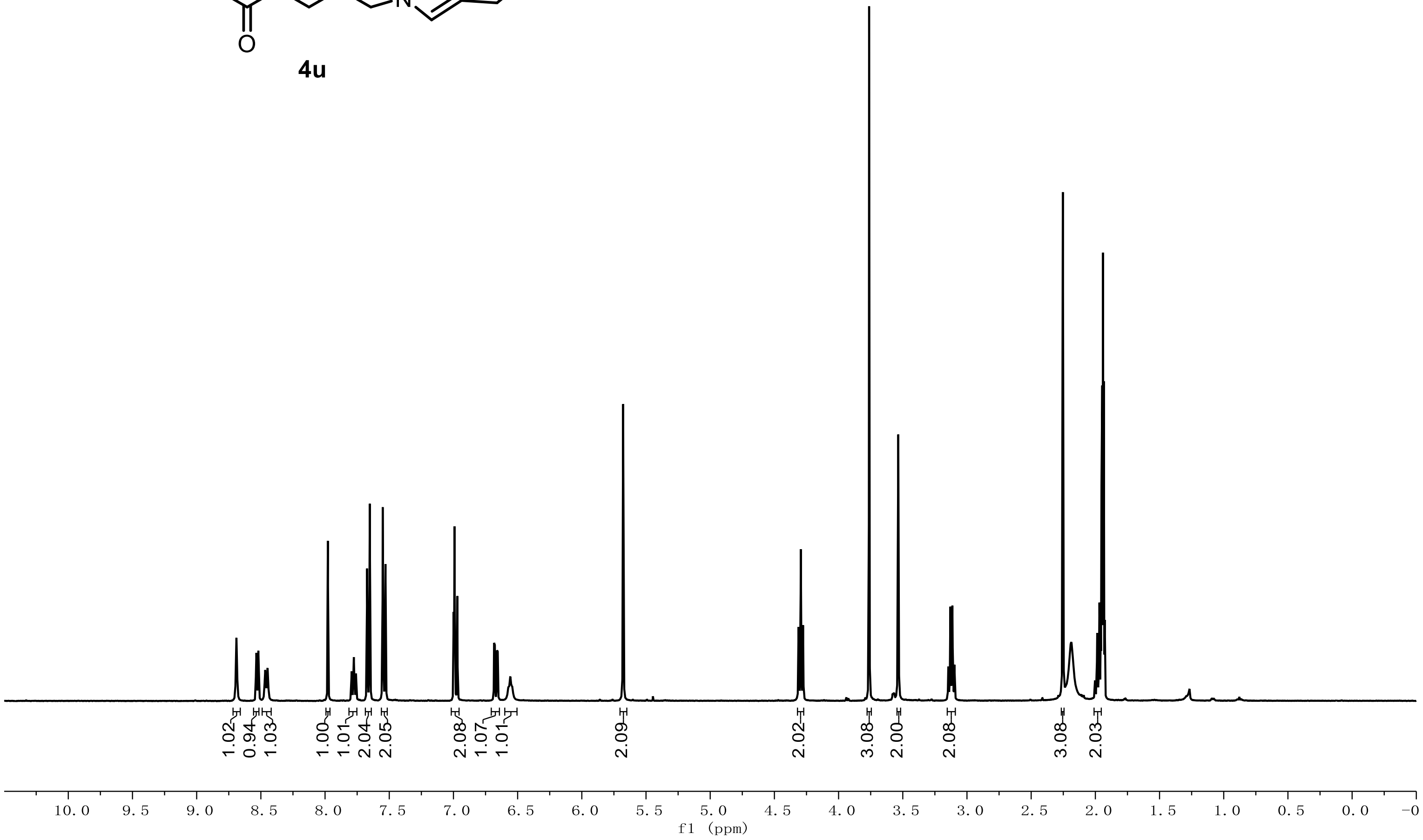

^{13}C NMR of 4u

CD_3CN, 101 MHz, 25 °C

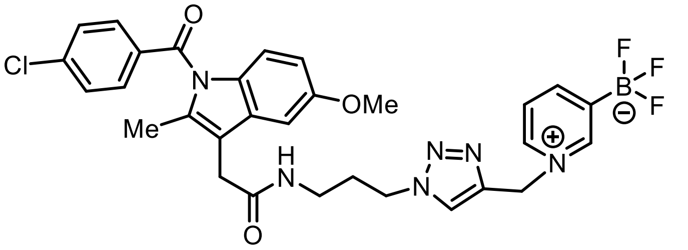

4u

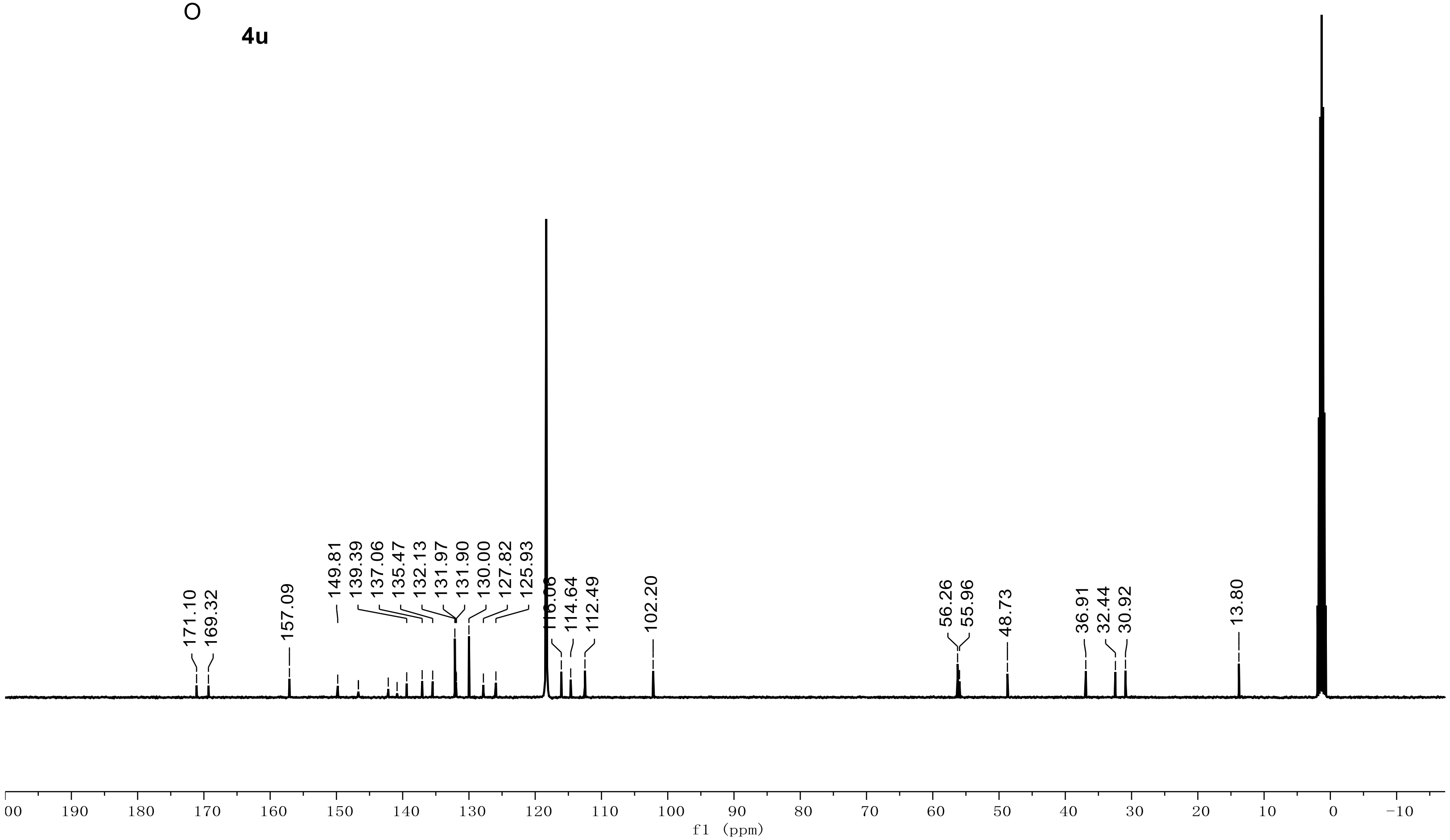

^{19}F NMR of 4u

CD_3CN, 376 MHz, 25 °C

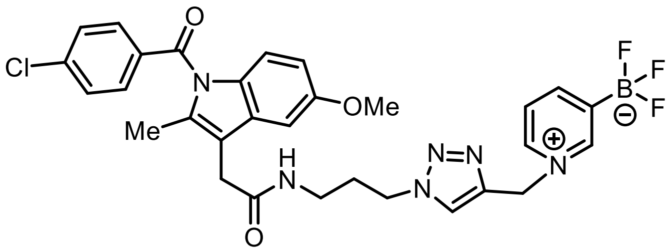

4u

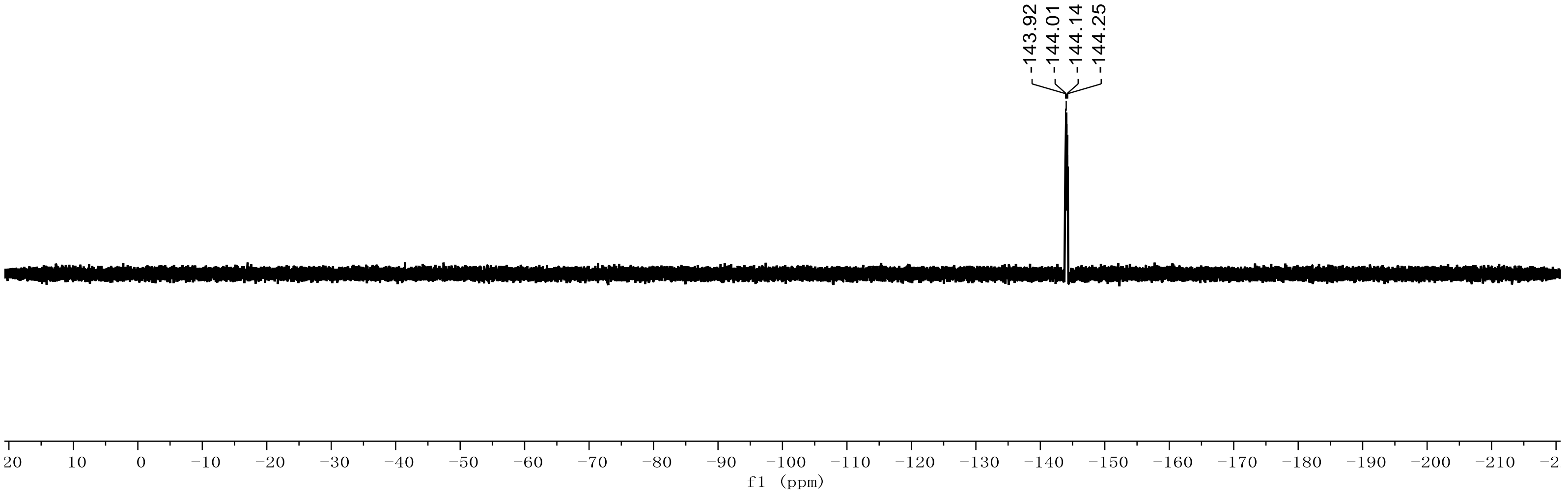

^{11}B NMR of 4u

CD_3CN, 128 MHz, 25 °C

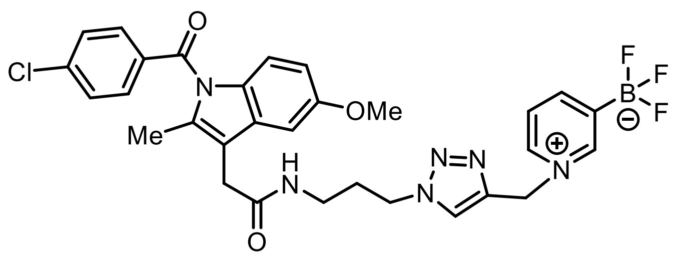

4u

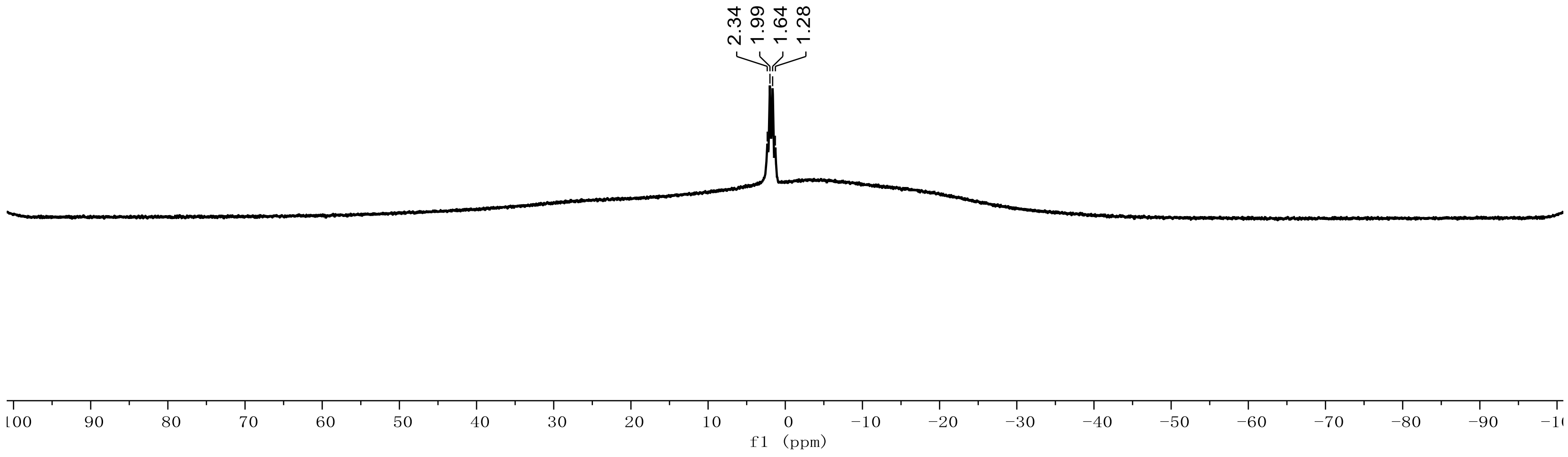

^{1}H NMR of 5u

CD$_3$CN, 400 MHz, 25 °C

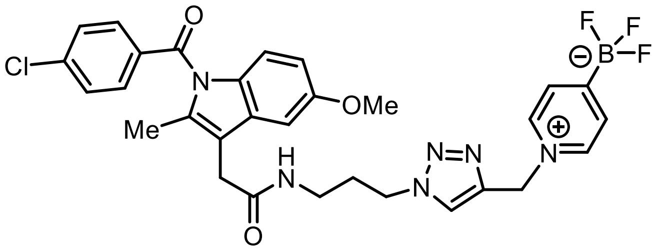

5u

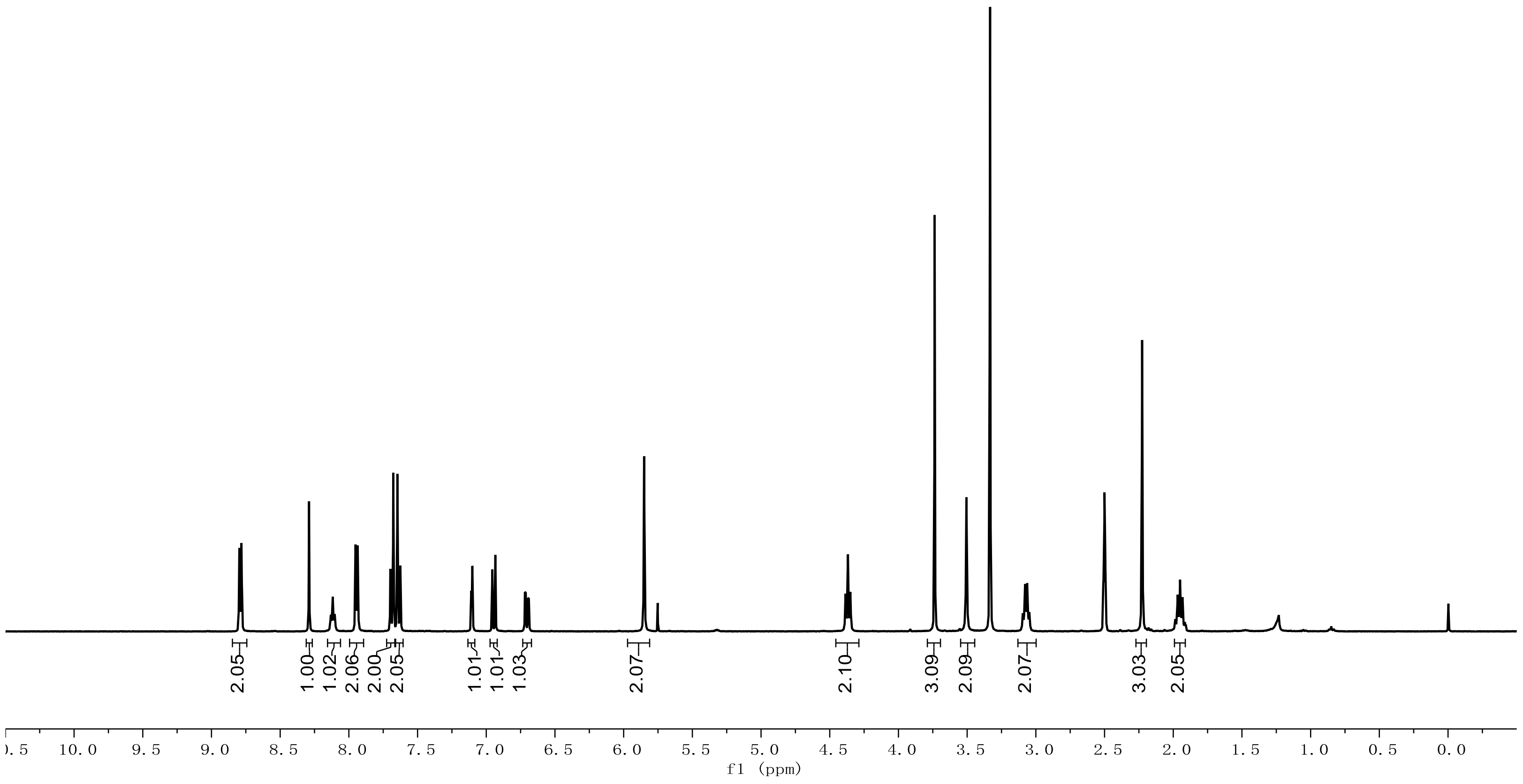

^{13}C NMR of 5u

CD_3CN, 101 MHz, 25 °C

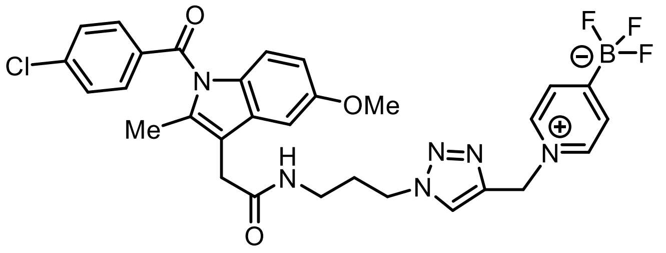

5u

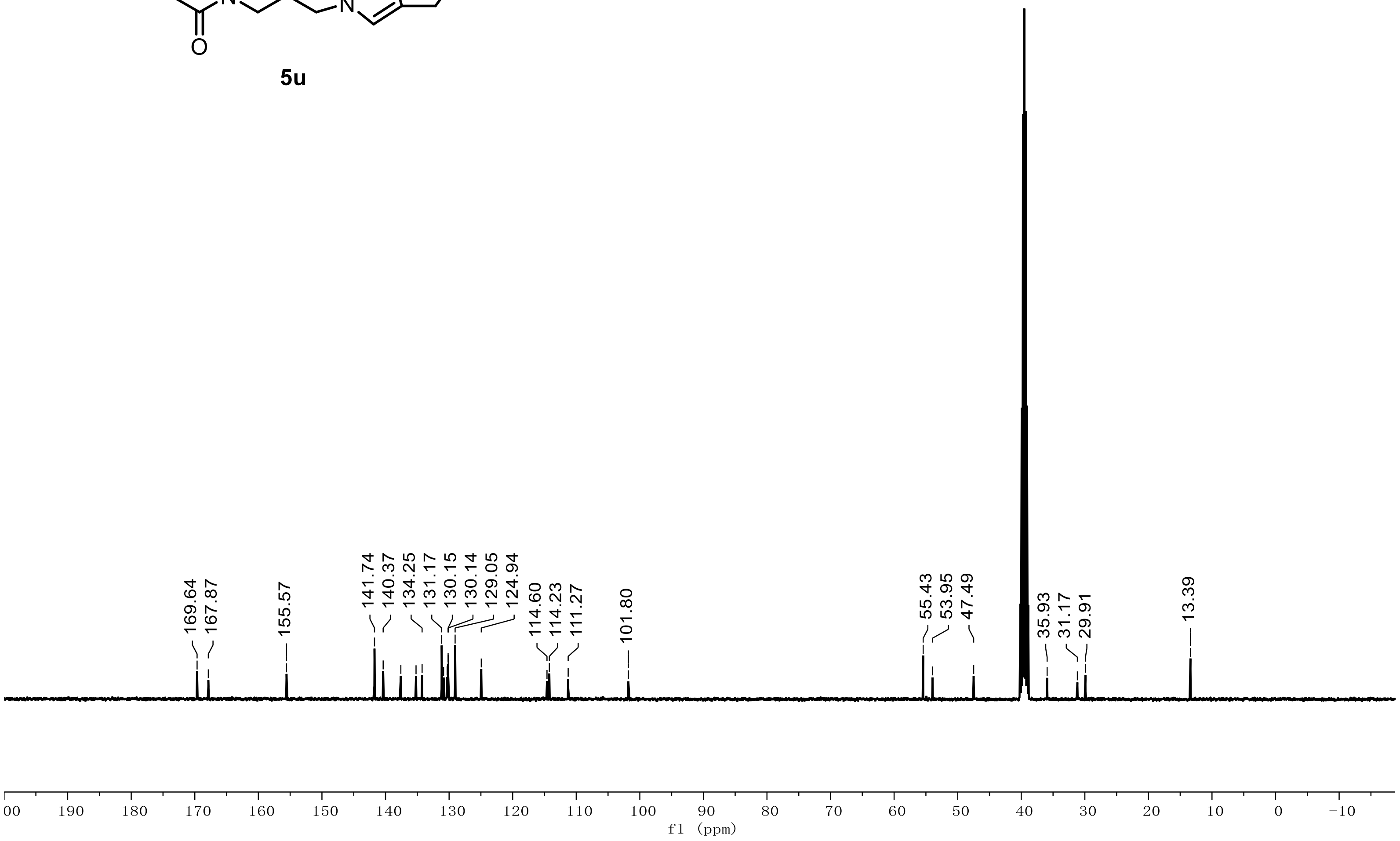

^{19}F NMR of 5u

CD$_3$CN, 376 MHz, 25 °C

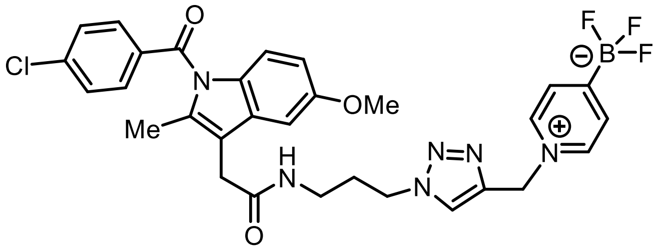

5u

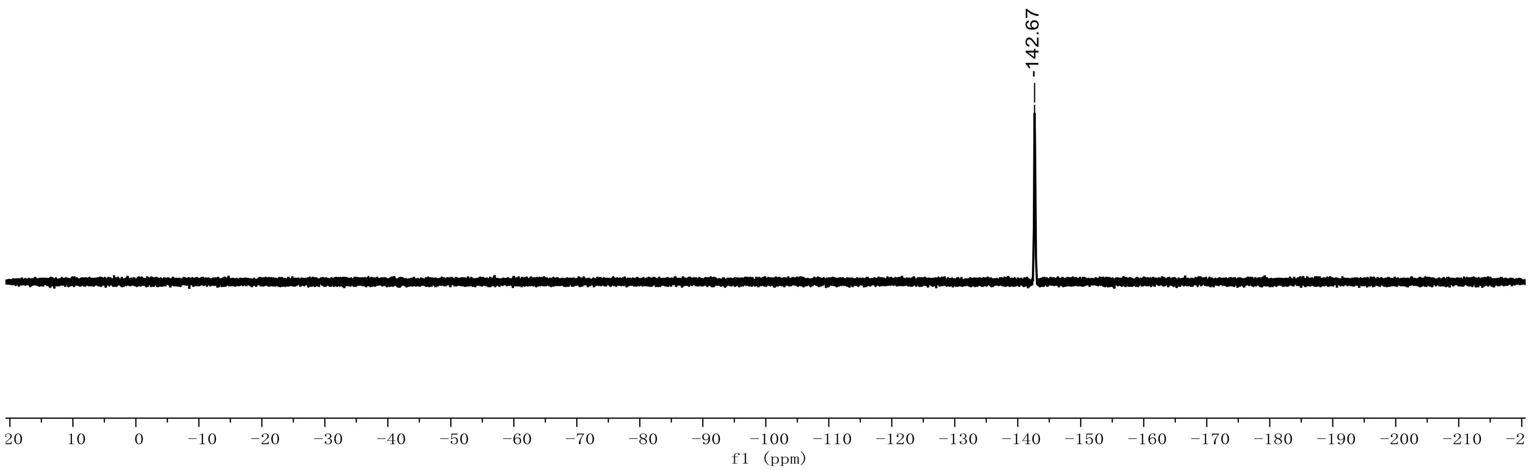

^{11}B NMR of 5u

CD_3CN, 128 MHz, 25 °C

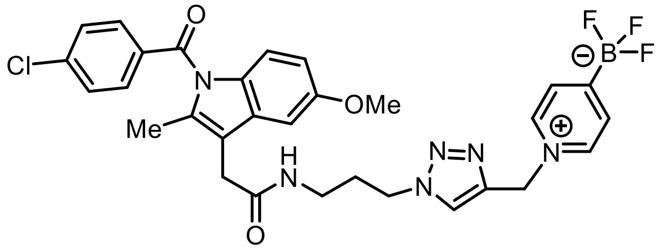

5u

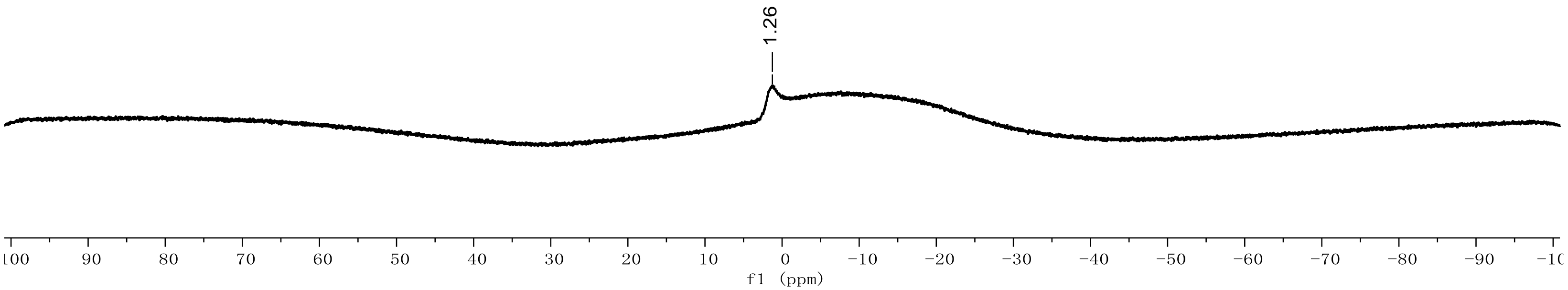

^{1}H NMR of 2c

$CDCl_3$, 400 MHz, 25 °C

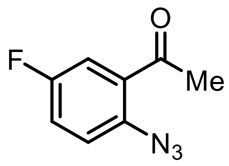

2c

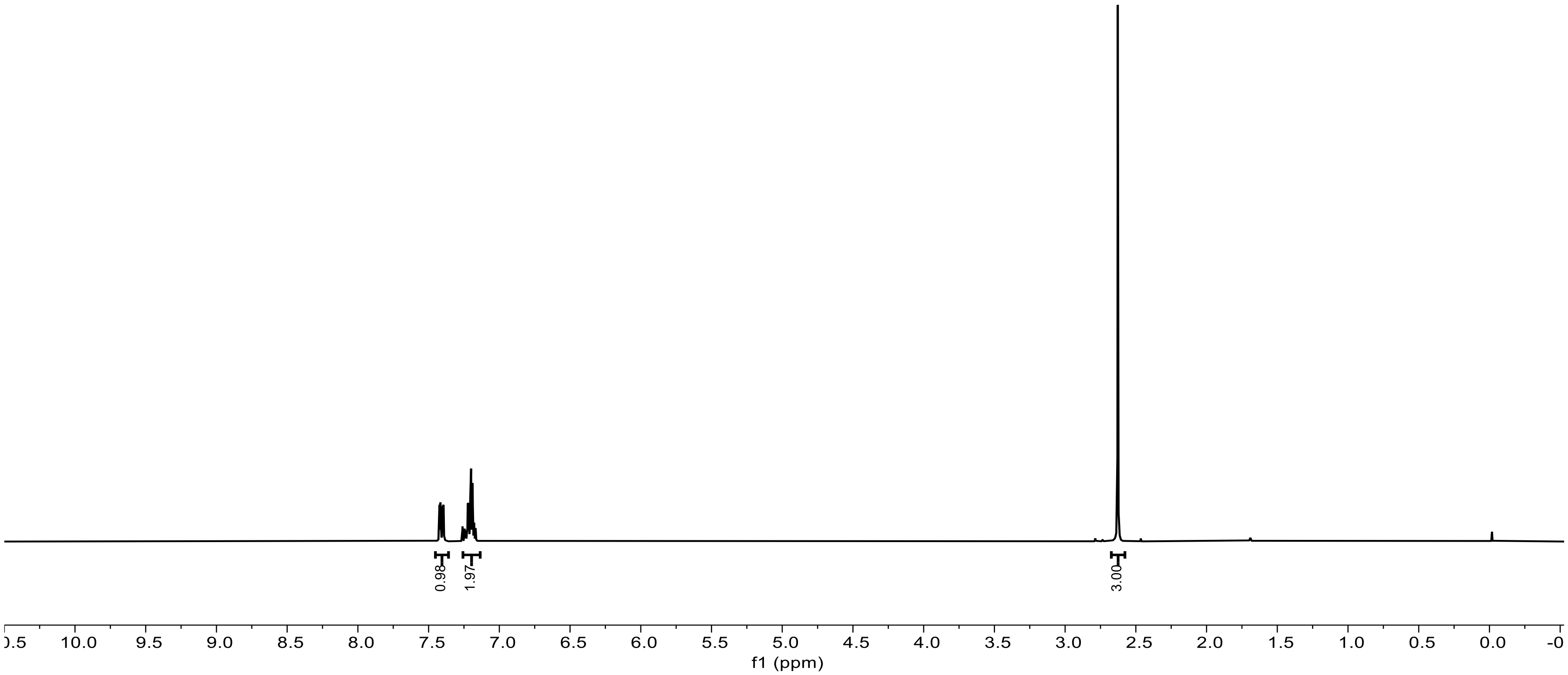

^{13}C NMR of 2c

$CDCl_3$, 101 MHz, 25 °C

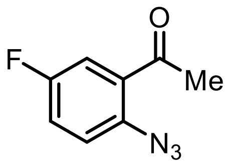

2c

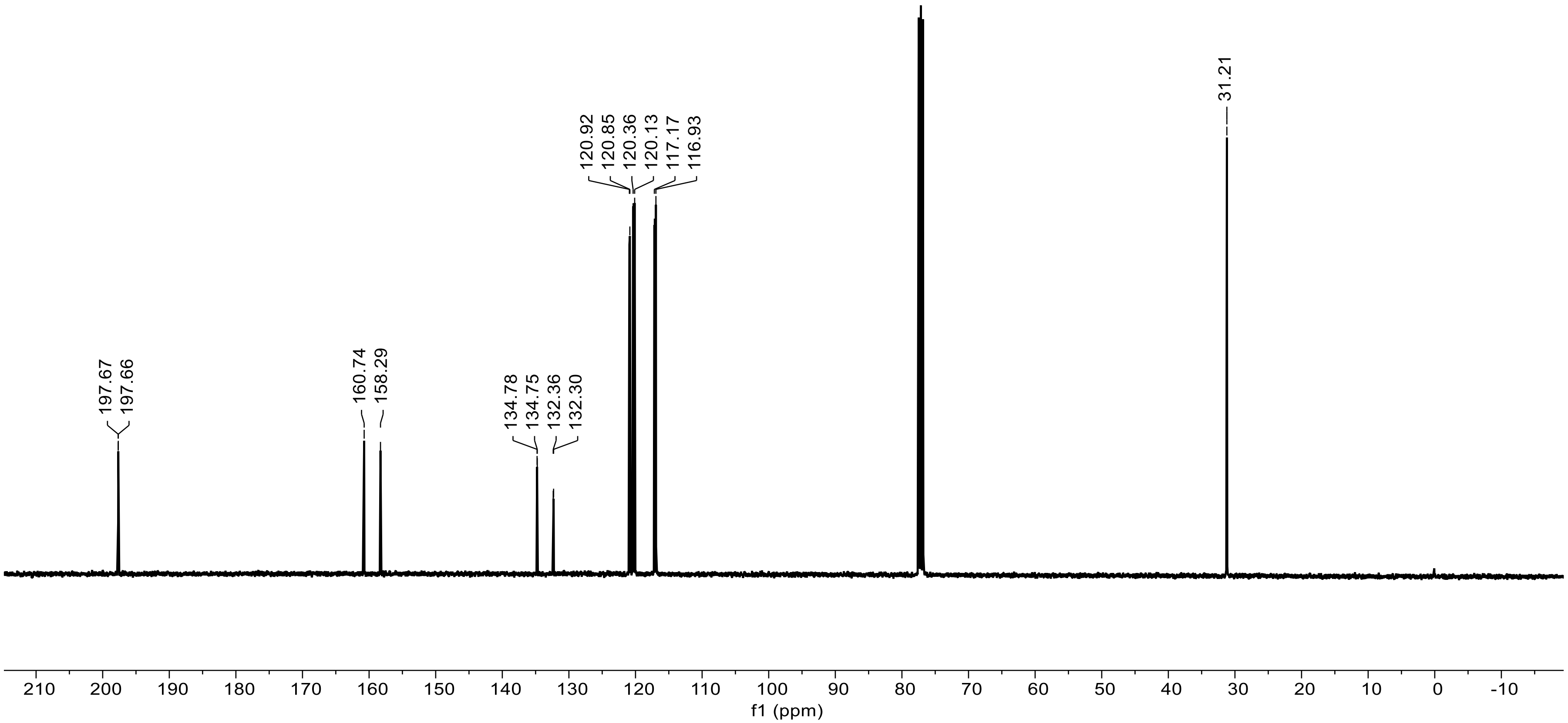

^{19}F NMR of 2c

$CDCl_3$, 376 MHz, 25 °C

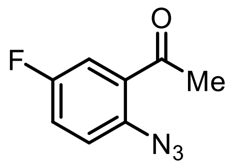

2c

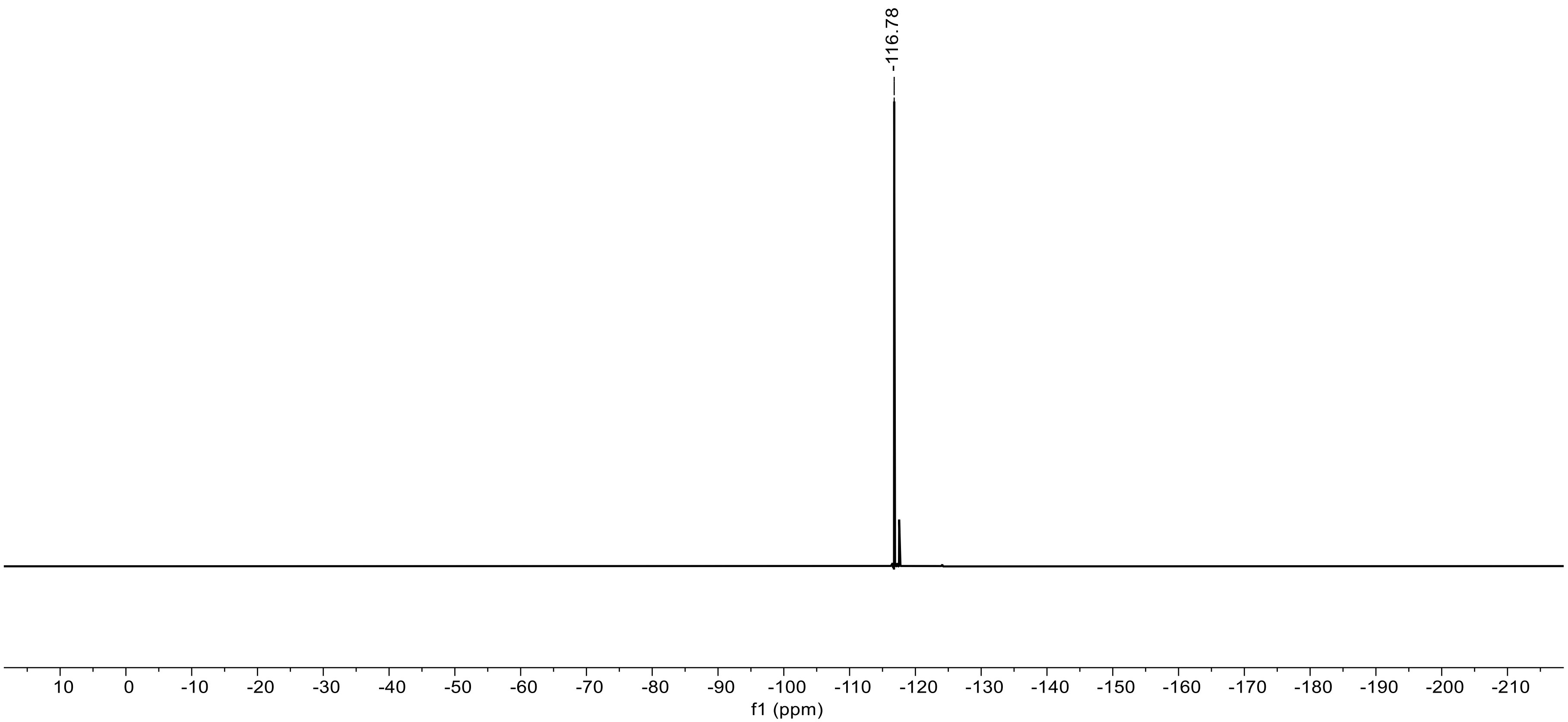

^{1}H NMR of 2n

CDCl$_3$, 400 MHz, 25 °C

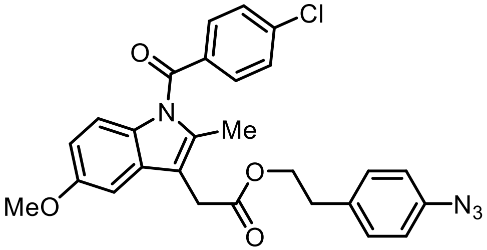

2n

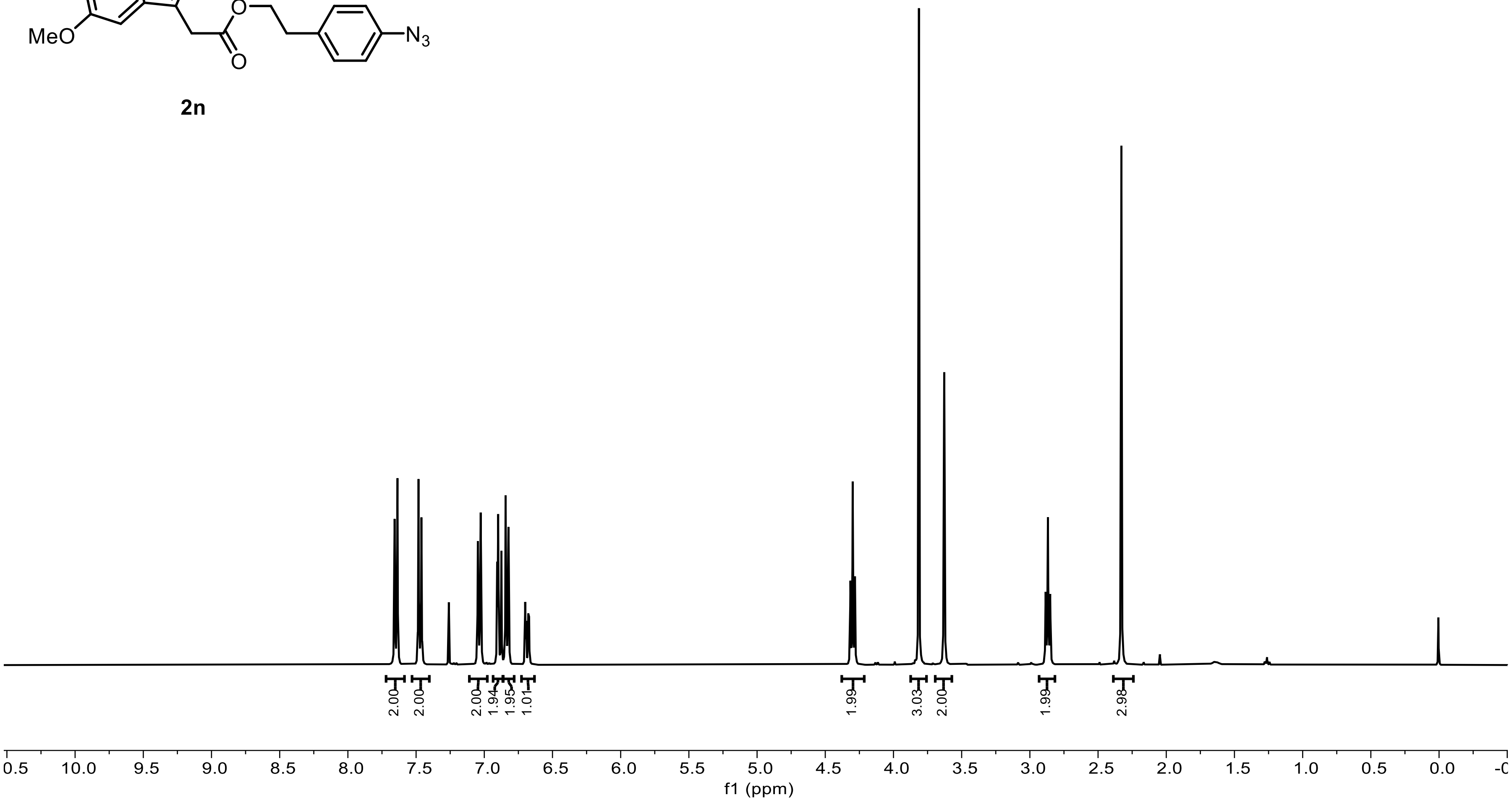

^{13}C NMR of 2n

CDCl$_3$, 101 MHz, 25 °C

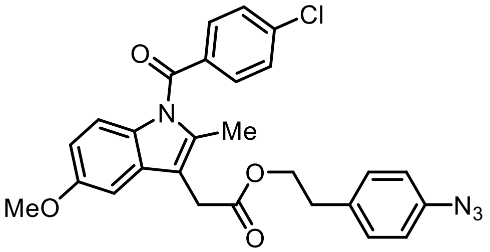

2n

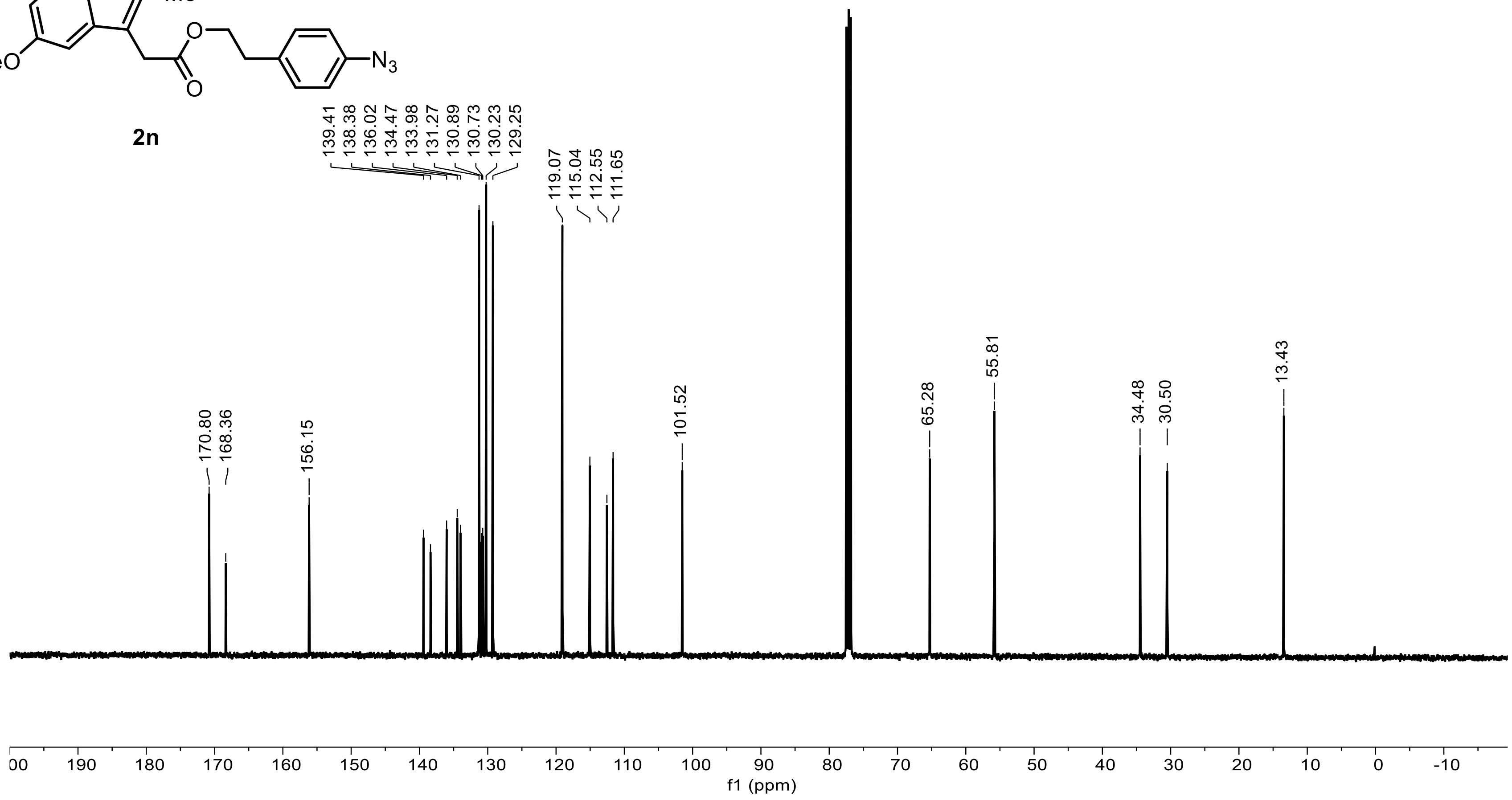

¹H NMR of 2r

DMSO-d_6, 400 MHz, 25 °C

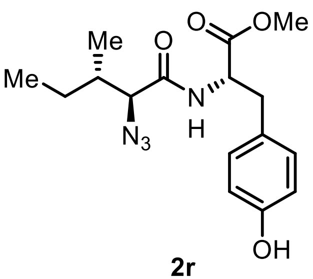

2r

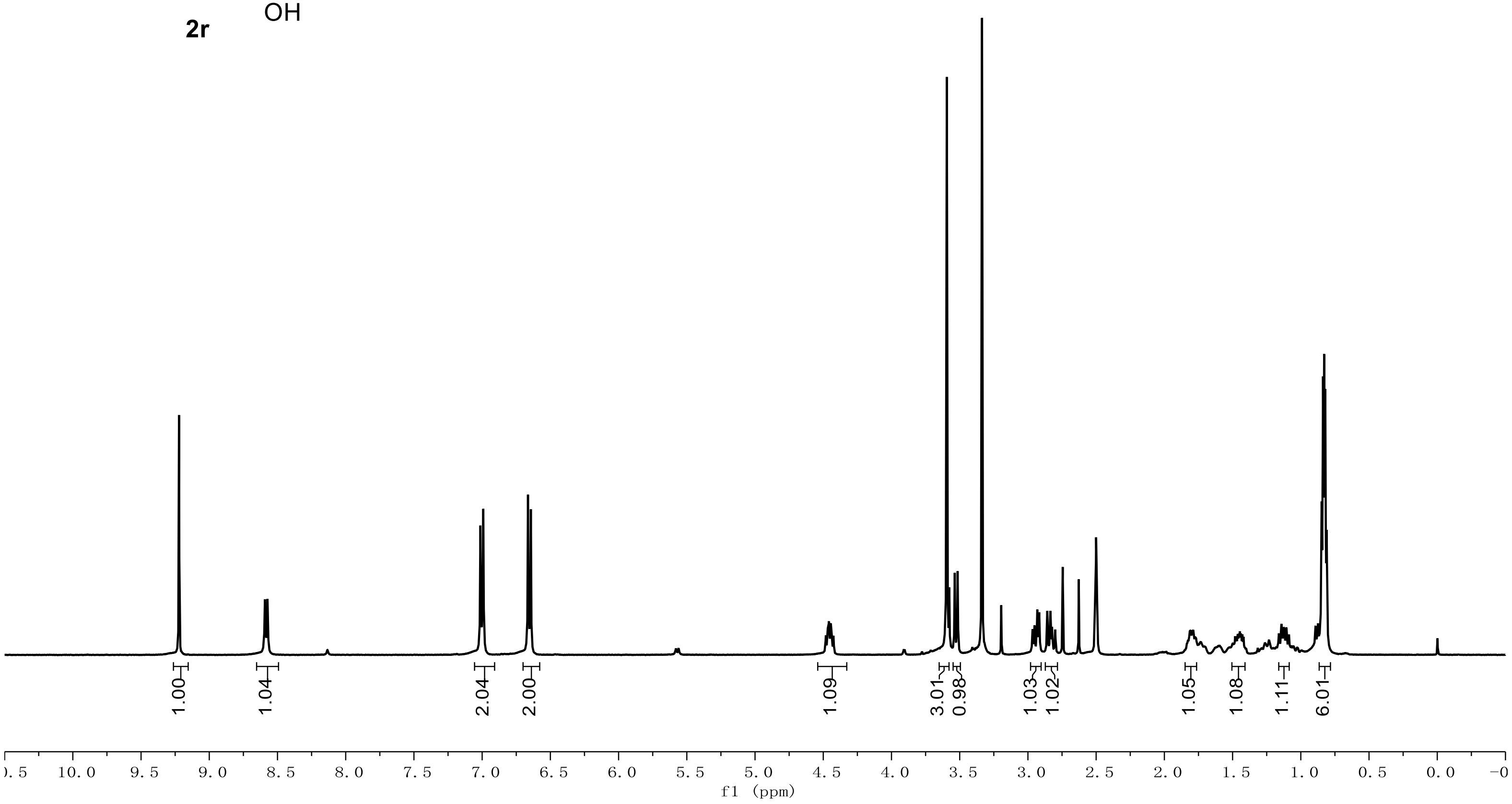

^{13}C NMR of 2r

DMSO-d_6, 101 MHz, 25 °C

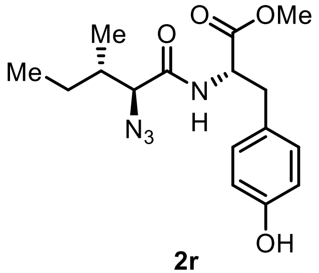

2r

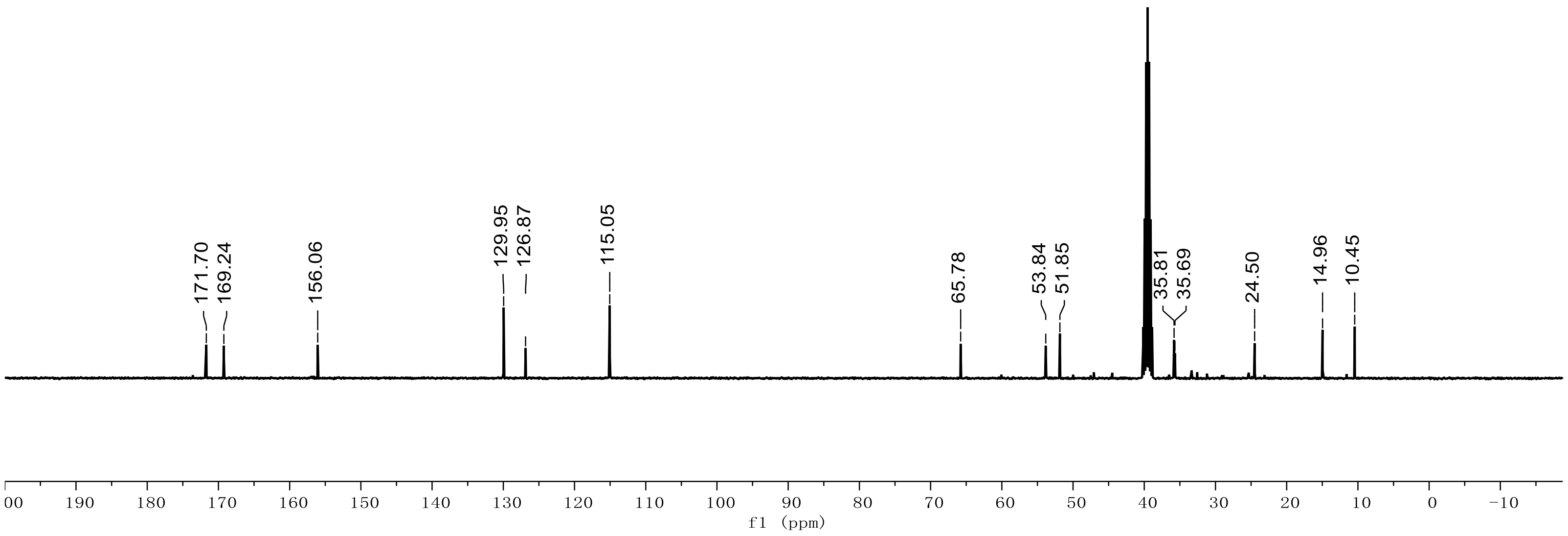

^{1}H NMR of 2u

$CDCl_3$, 400 MHz, 25 °C

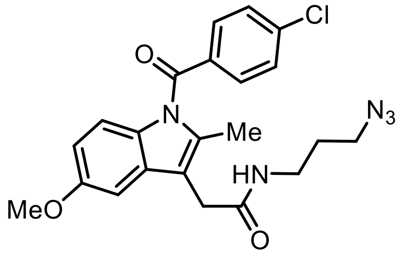

2u

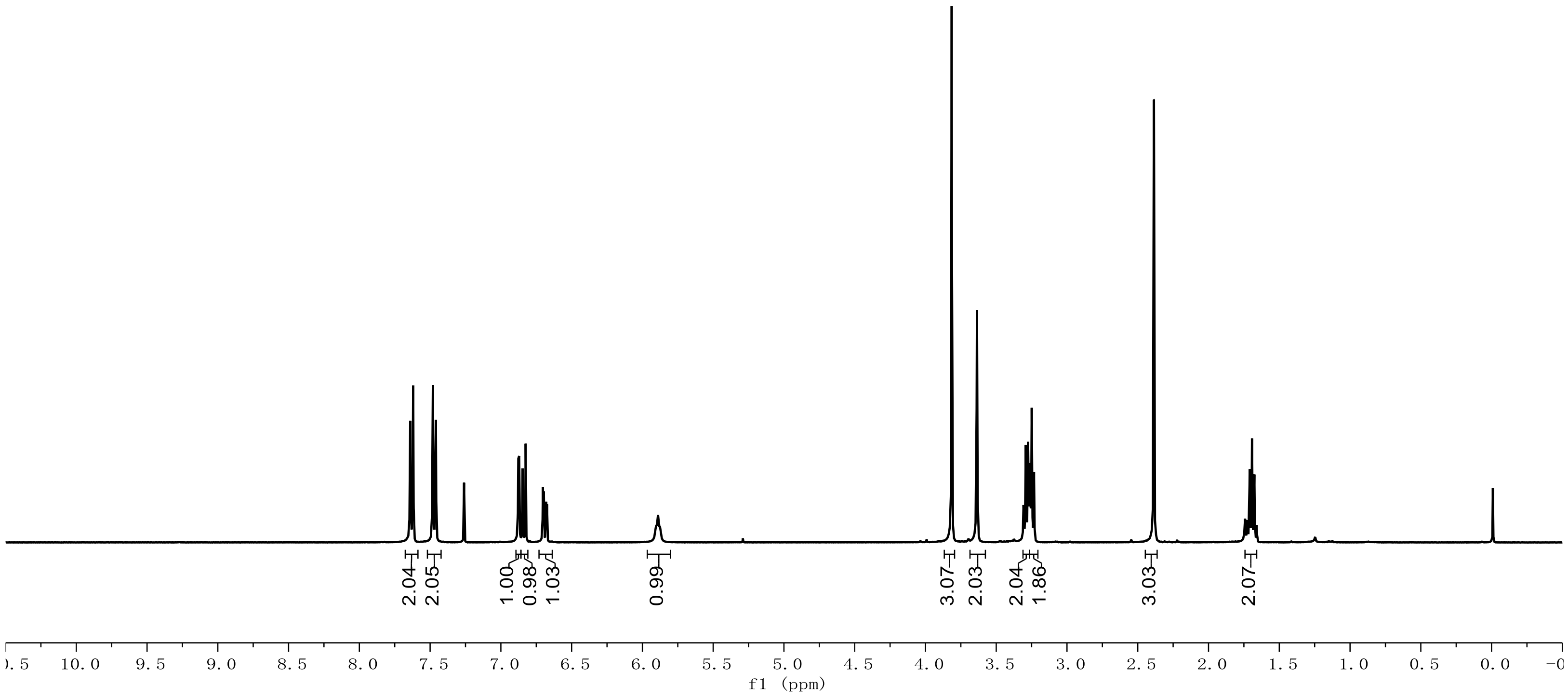

^{13}C NMR of 2u

$CDCl_3$, 101 MHz, 25 °C

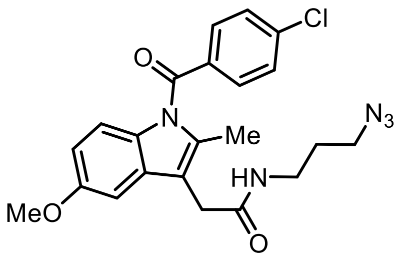

2u

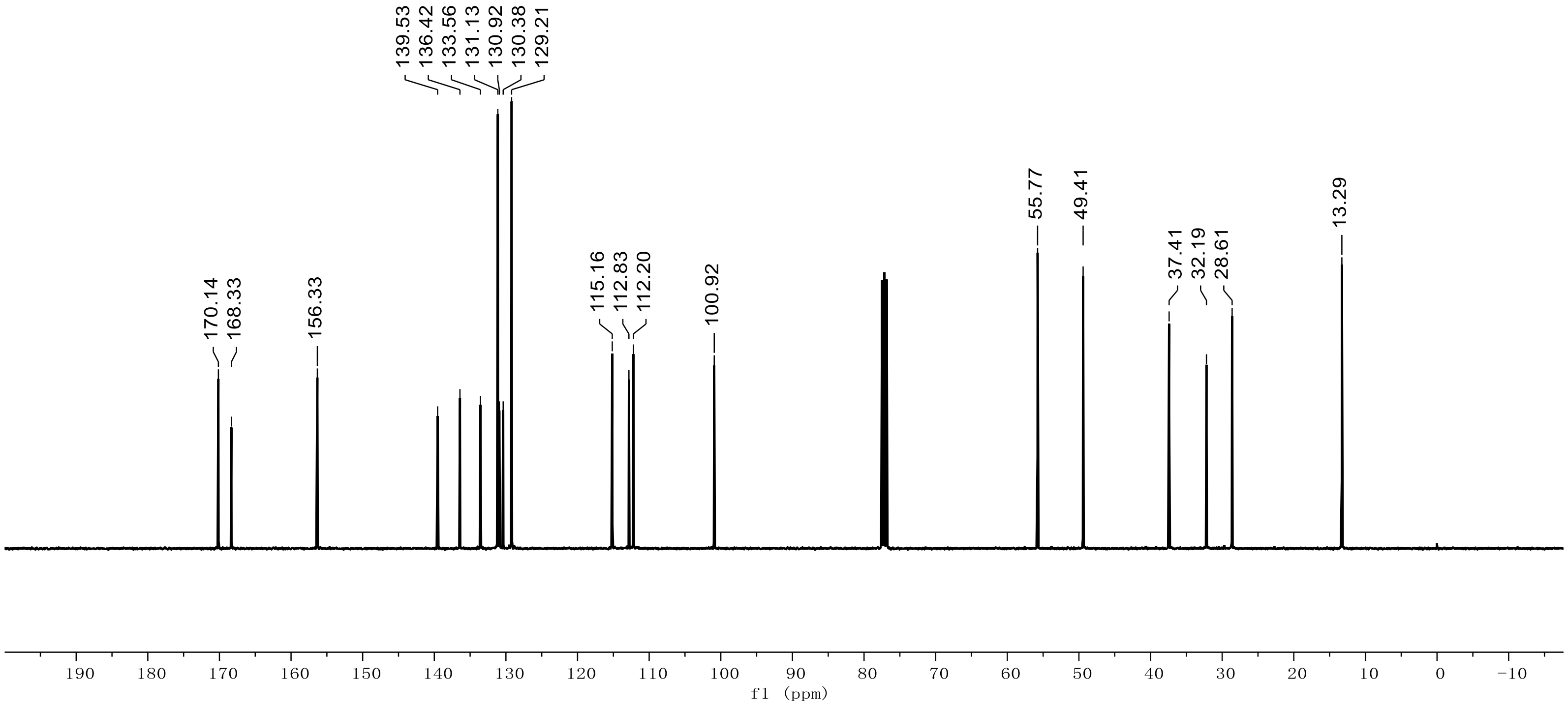

^{1}H NMR of 1a

CD_3CN, 400 MHz, 25 °C

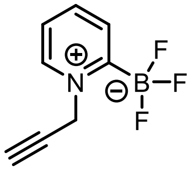

1a

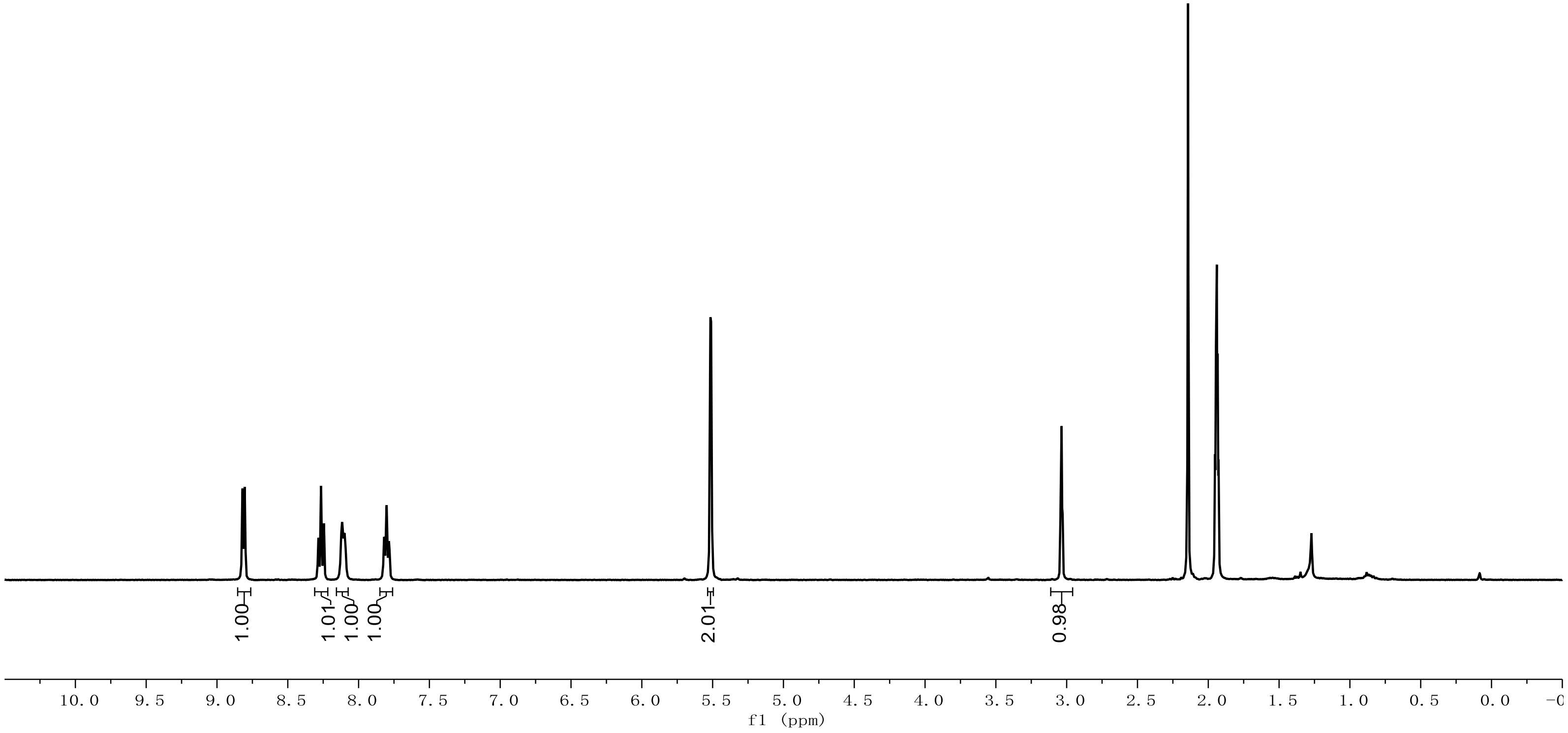

^{19}F NMR of 1a

CD_3CN, 376 MHz, 25 °C

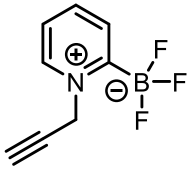

1a

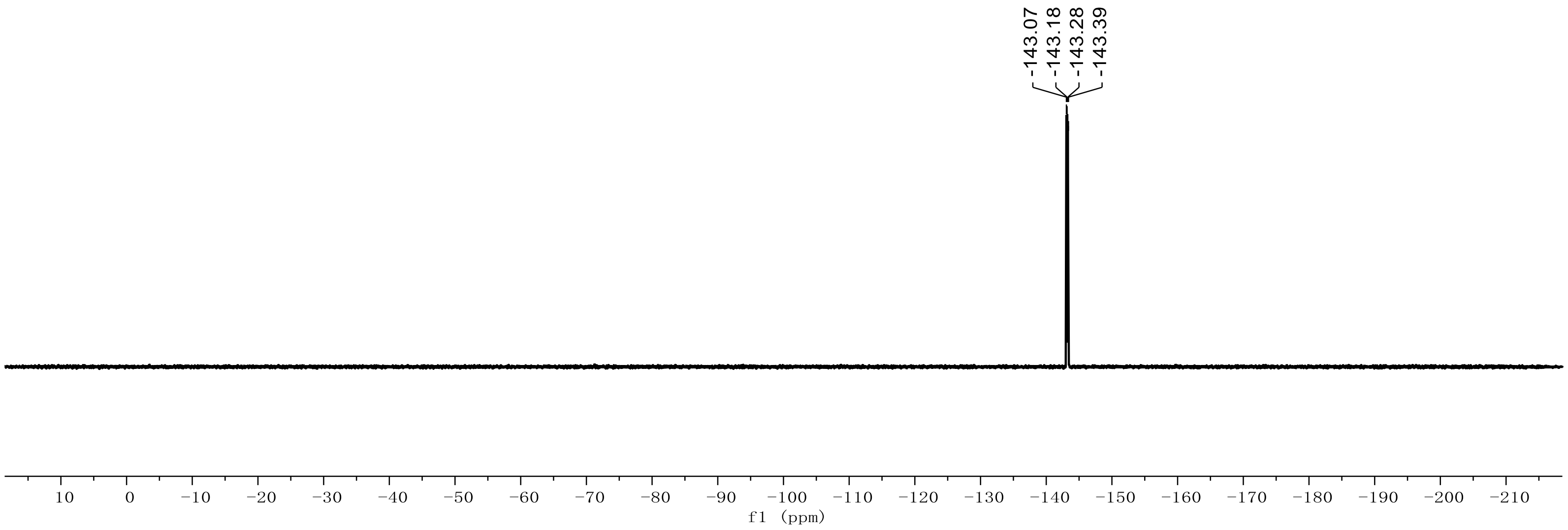

^{11}B NMR of 1a

CD_3CN, 128 MHz, 25 °C

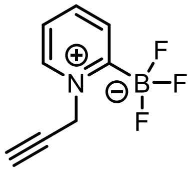

1a

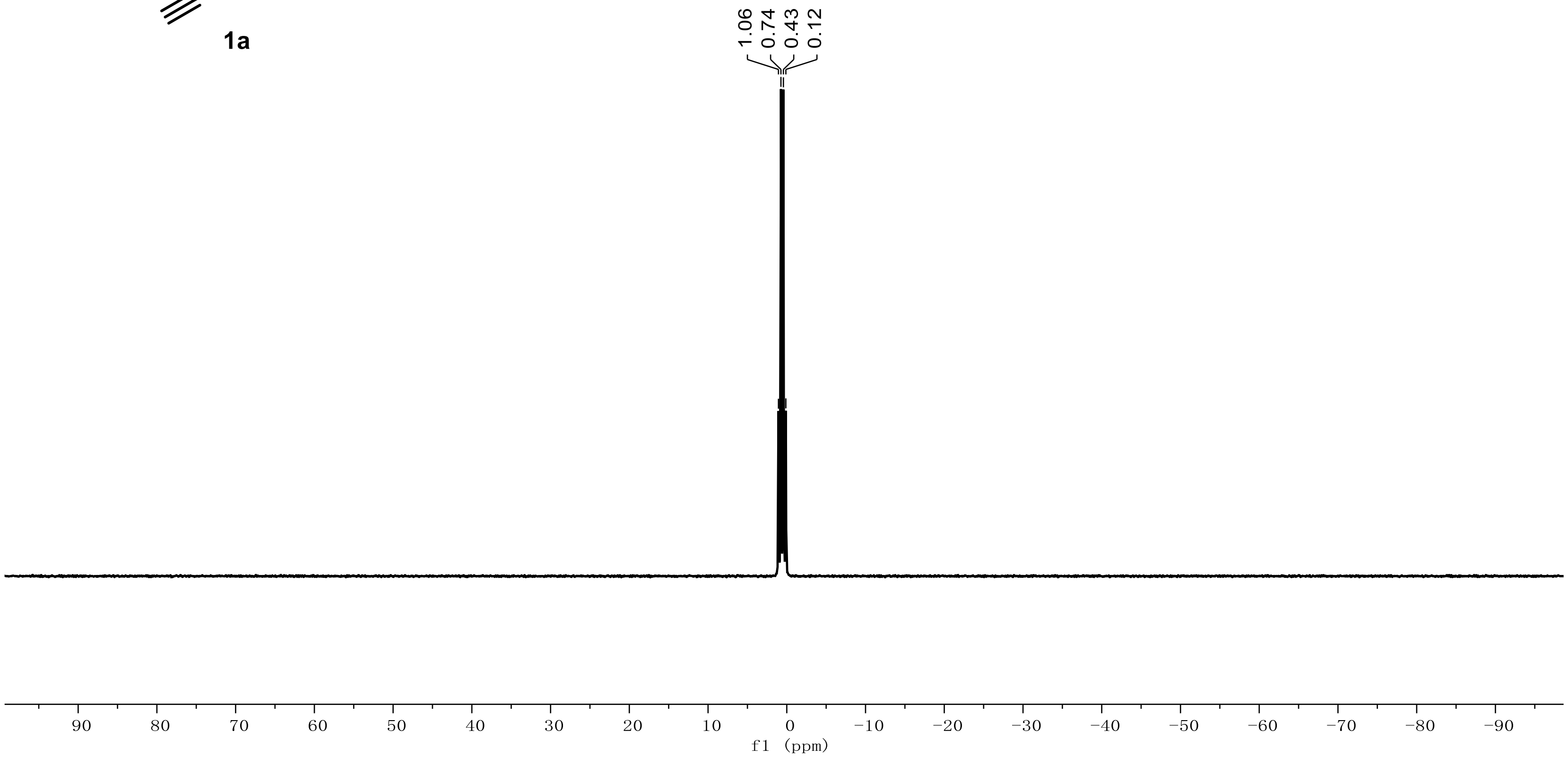

^{13}C NMR of 1a

CD_3CN, 101 MHz, 25 °C

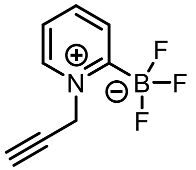

1a

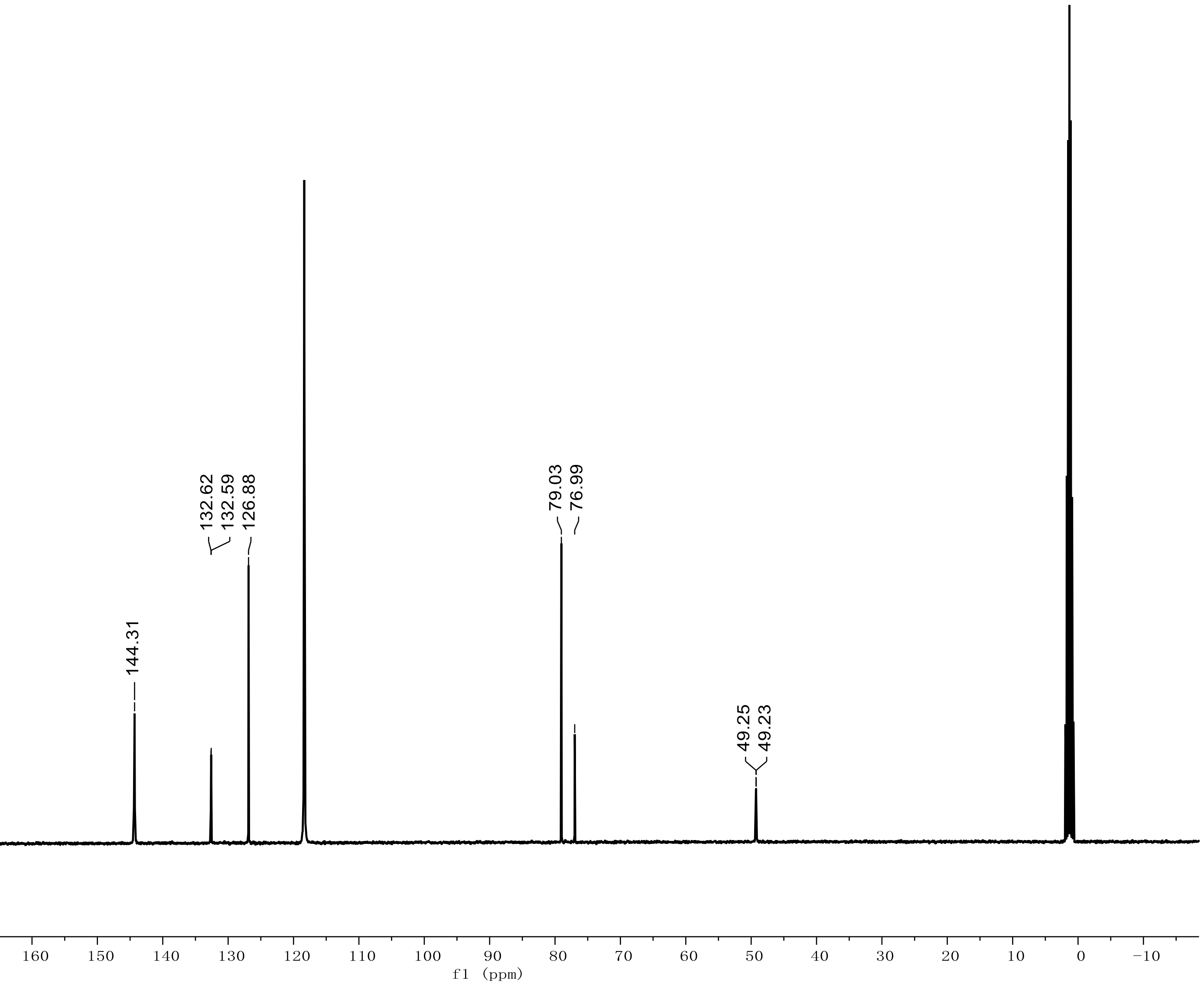

^{1}H NMR of 1b

CD_3CN, 400 MHz, 25 °C

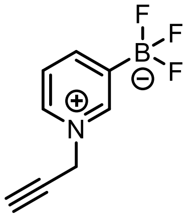

1b

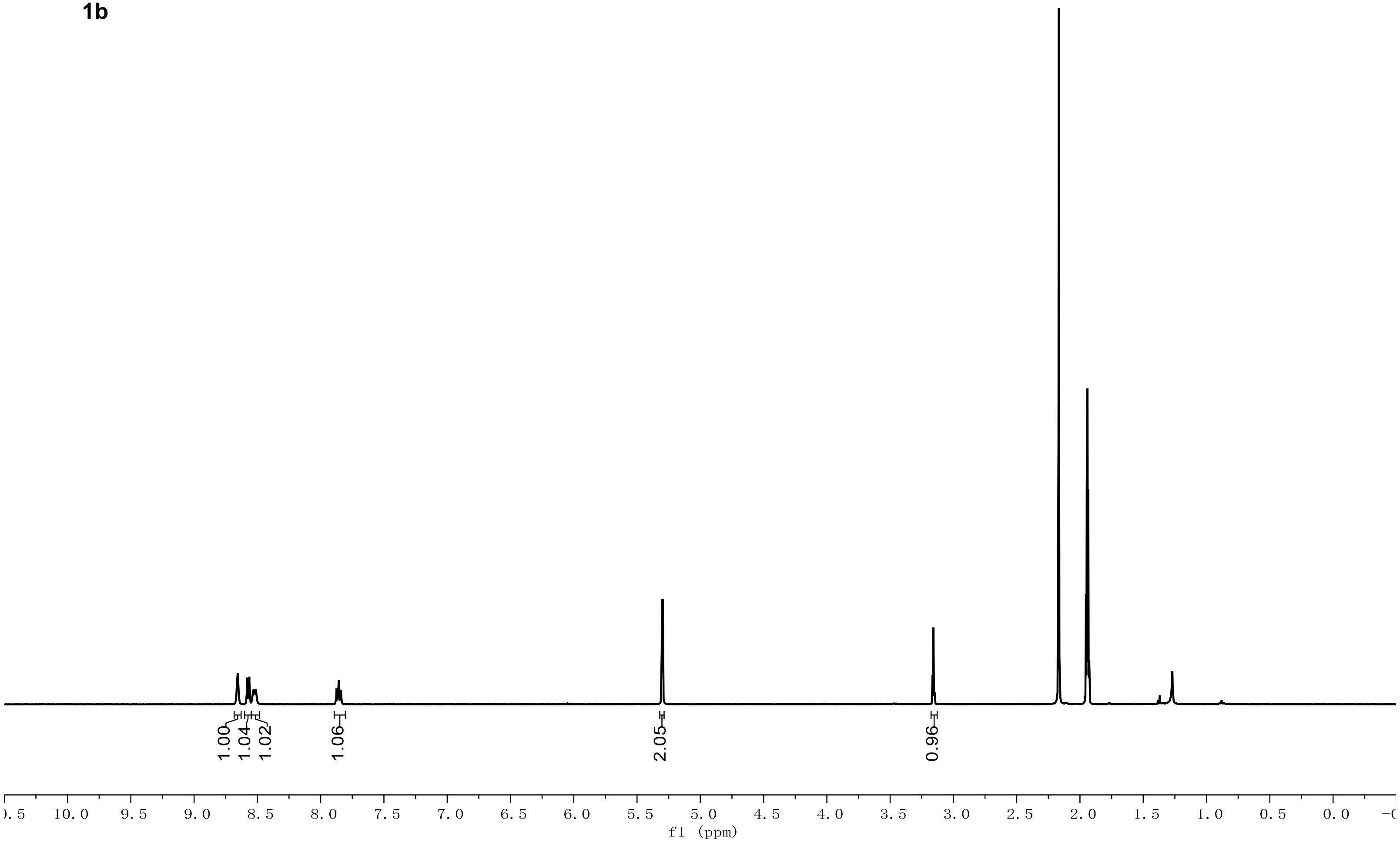

^{19}F NMR of 1b

CD_3CN, 376 MHz, 25 °C

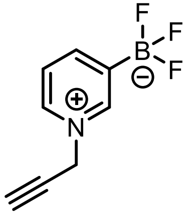

1b

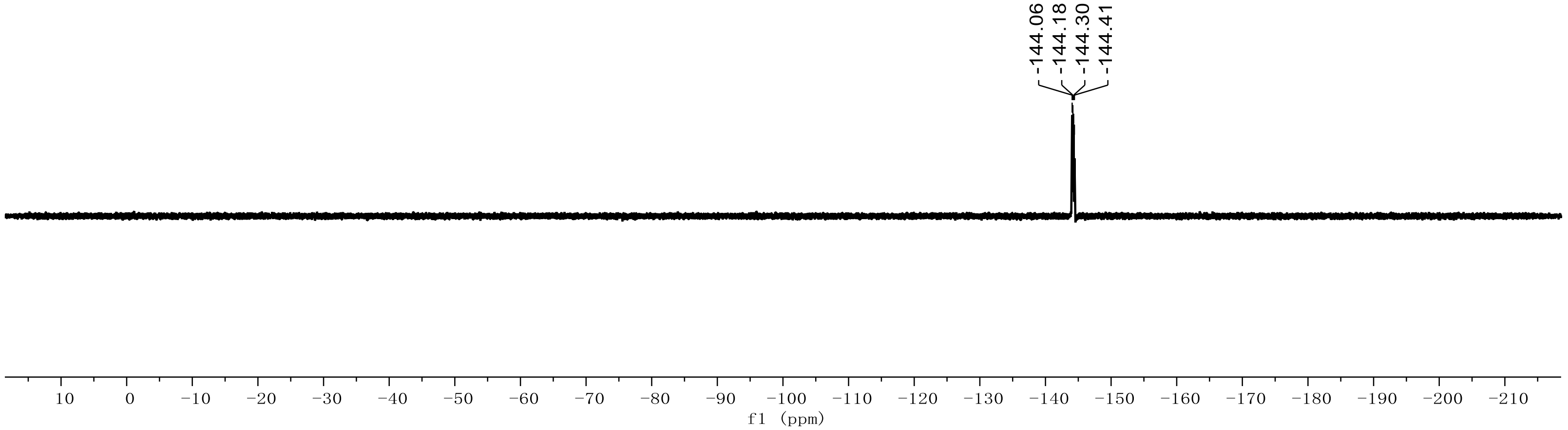

^{11}B NMR of 1b

CD_3CN, 128 MHz, 25 °C

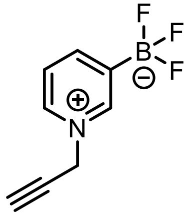

1b

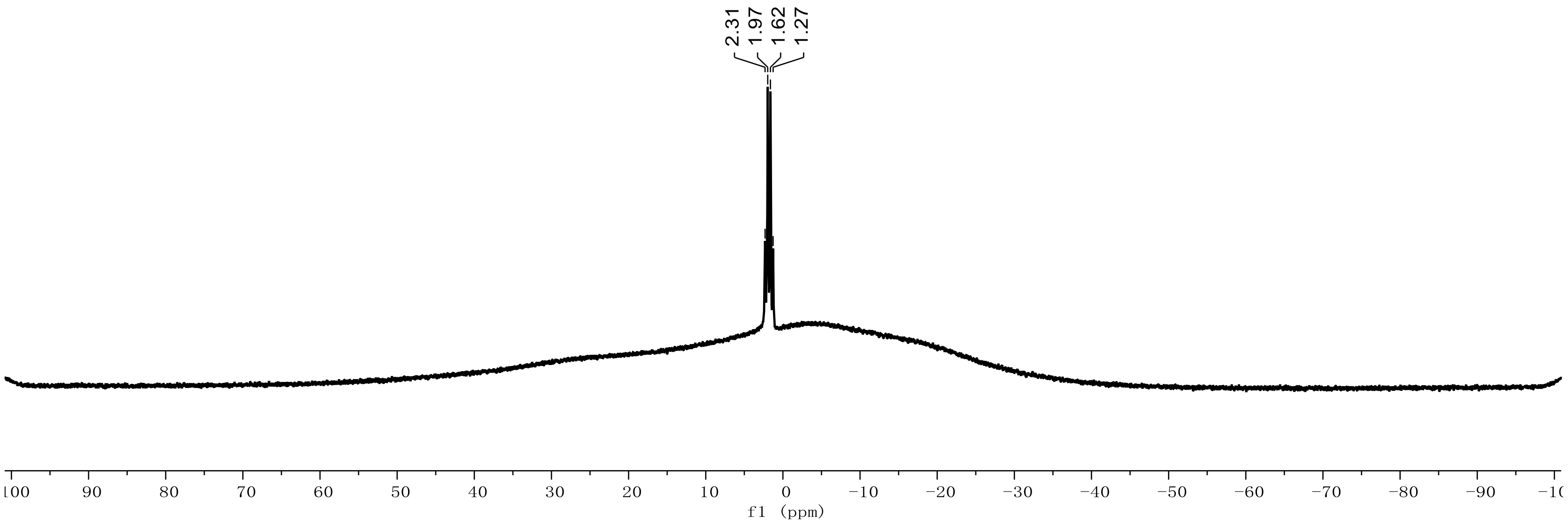

^{13}C NMR of 1b

CD_3CN, 101 MHz, 25 °C

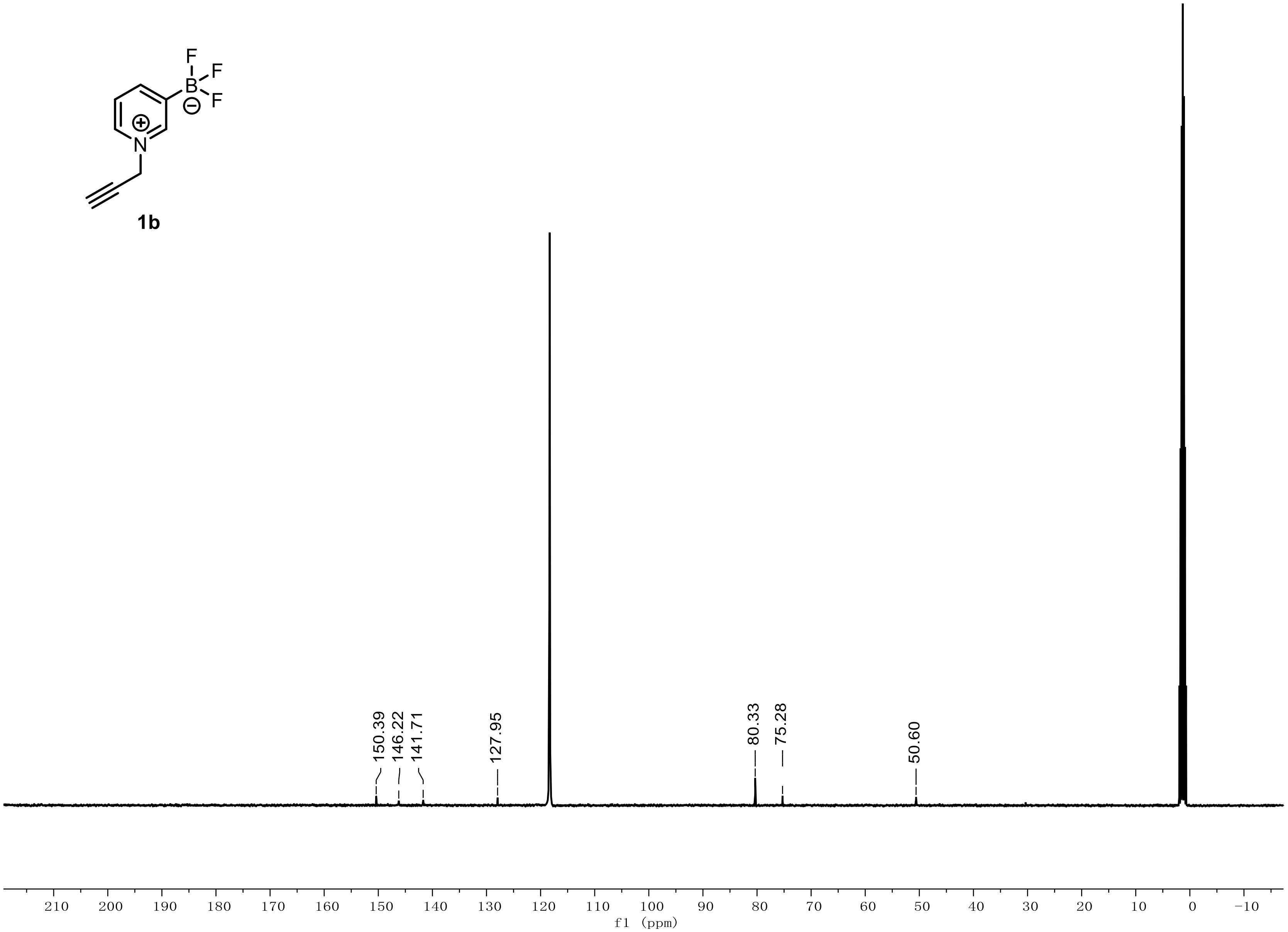

^{1}H NMR of 1c

CD_3CN, 400 MHz, 25 °C

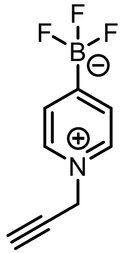

1c

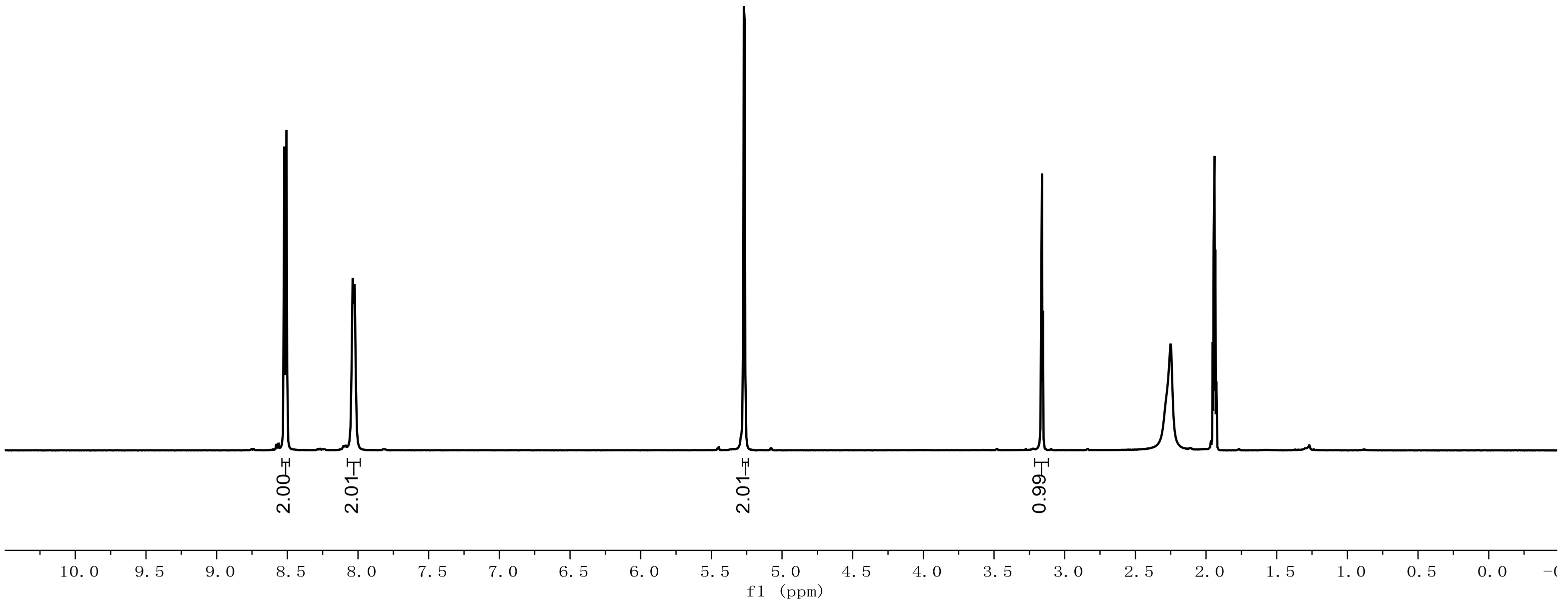

^{19}F NMR of 1c

CD_3CN, 376 MHz, 25 °C

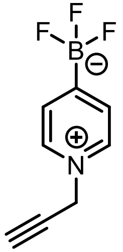

1c

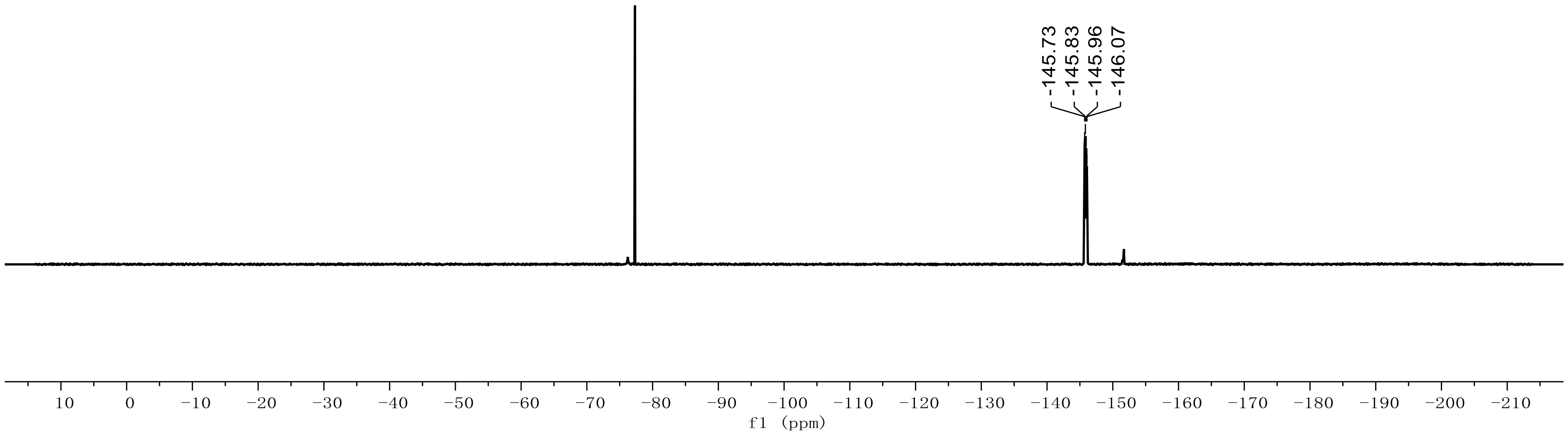

^{11}B NMR of 1c

CD_3CN, 128 MHz, 25 °C

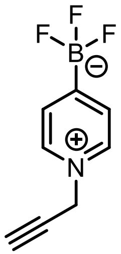

1c

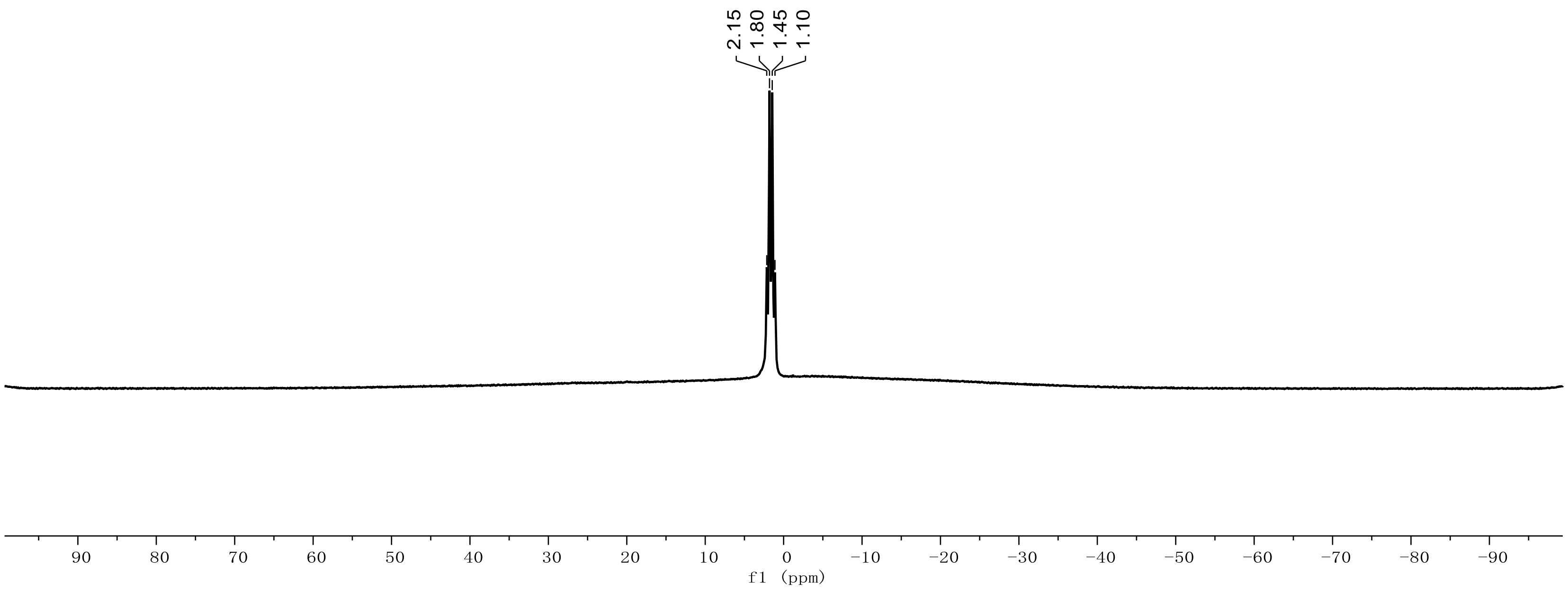

^{13}C NMR of 1c

CD_3CN, 101 MHz, 25 °C

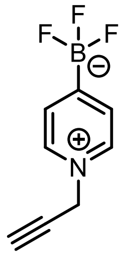

1c

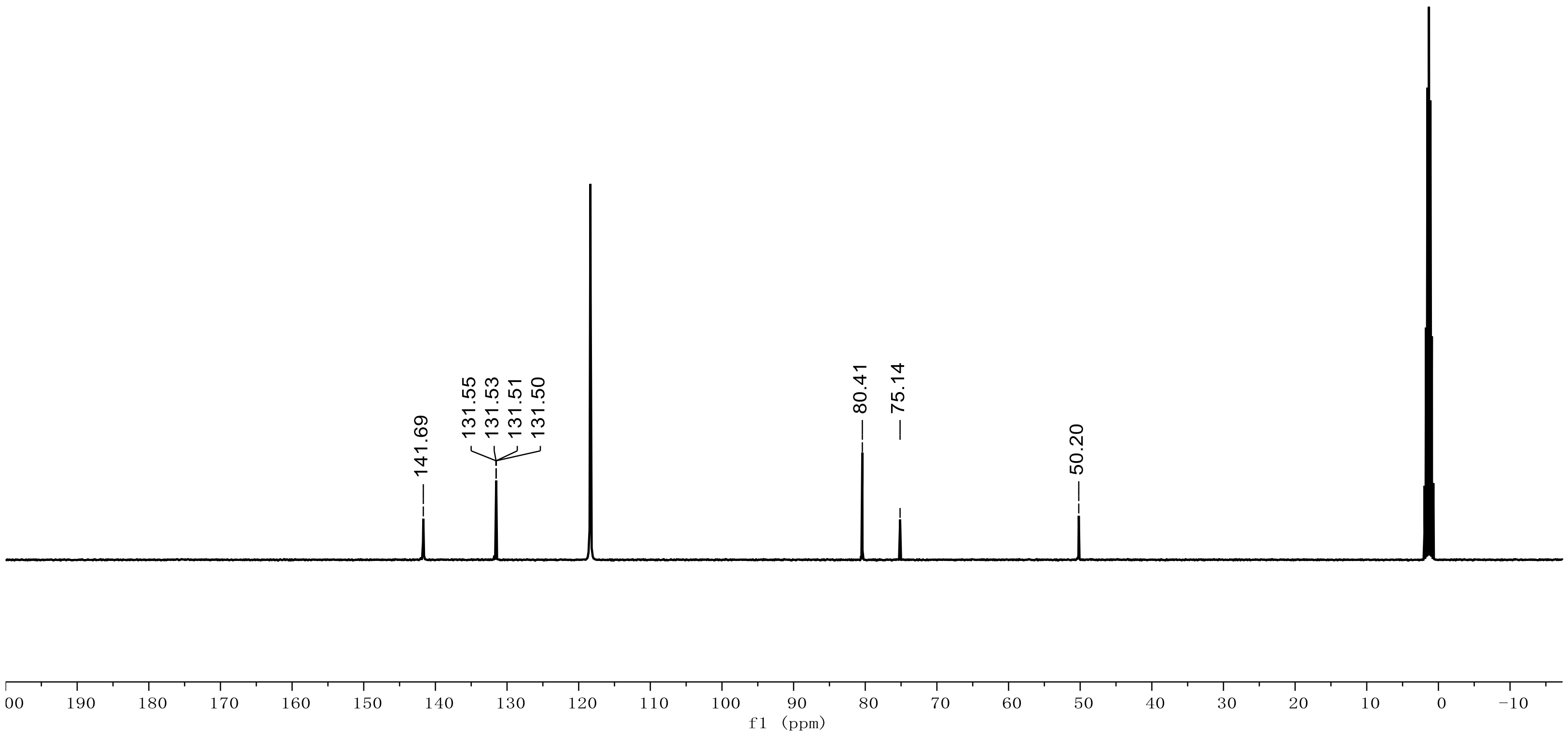

^{1}H NMR of 1d

DMSO-d_6, 400 MHz, 25 °C

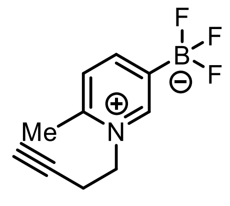

1d

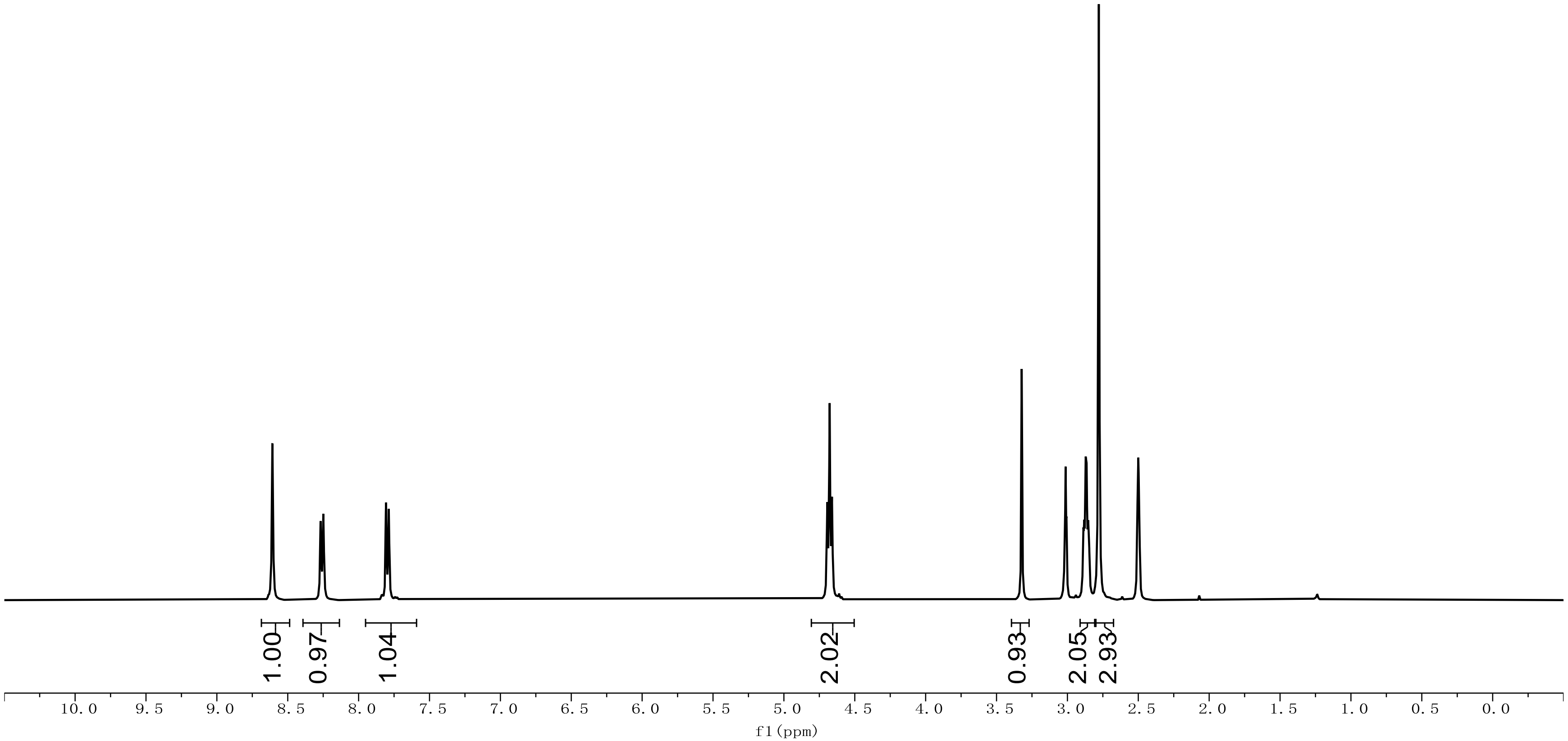

^{19}F NMR of 1d

DMSO-d_6, 376 MHz, 25 °C

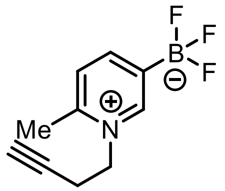

1d

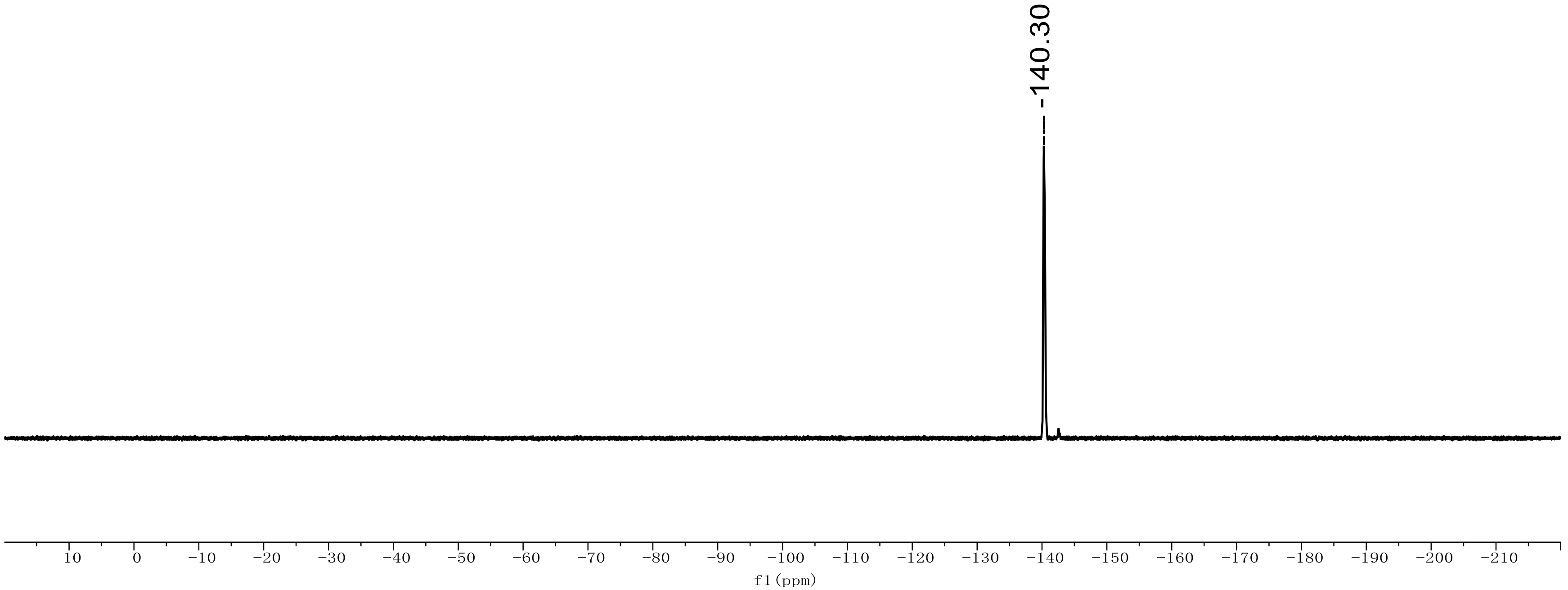

^{11}B NMR of 1d

DMSO-d_6, 128 MHz, 25 °C

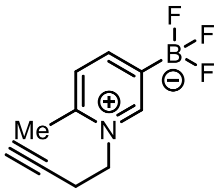

1d

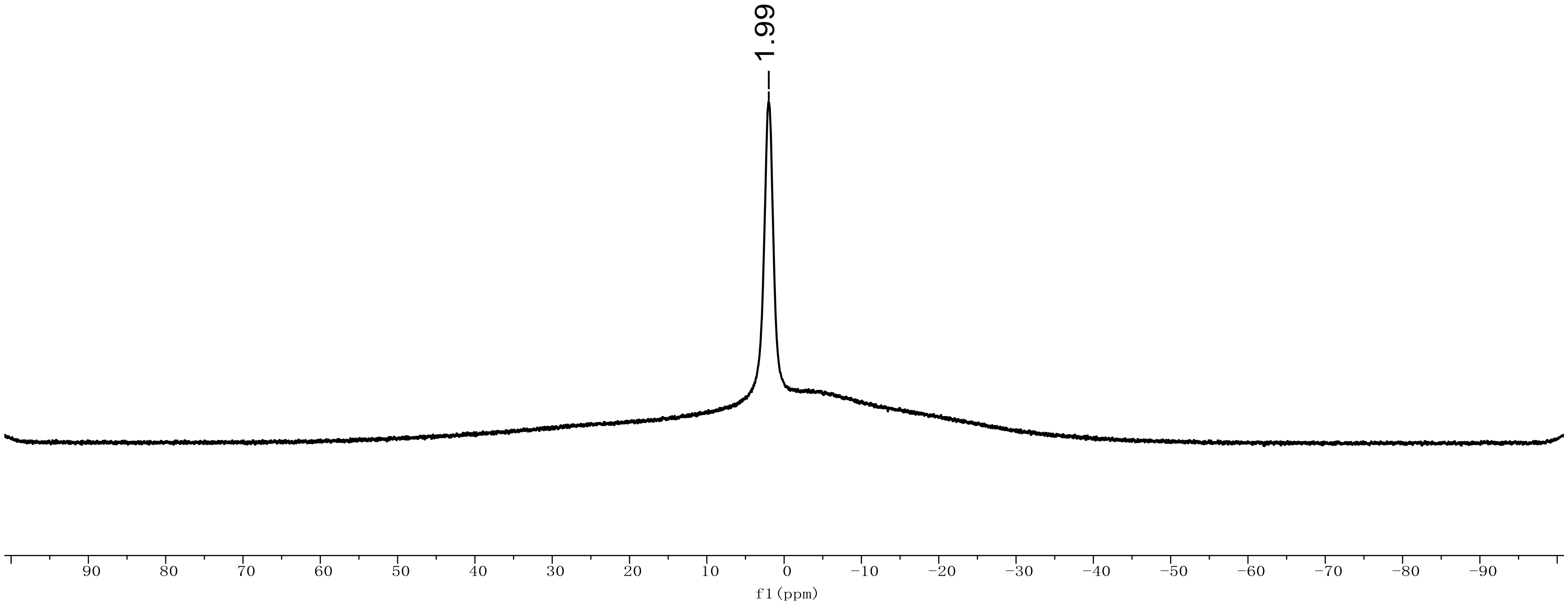

^{13}C NMR of 1d

DMSO-d_6, 101 MHz, 25 °C

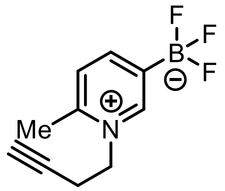

1d

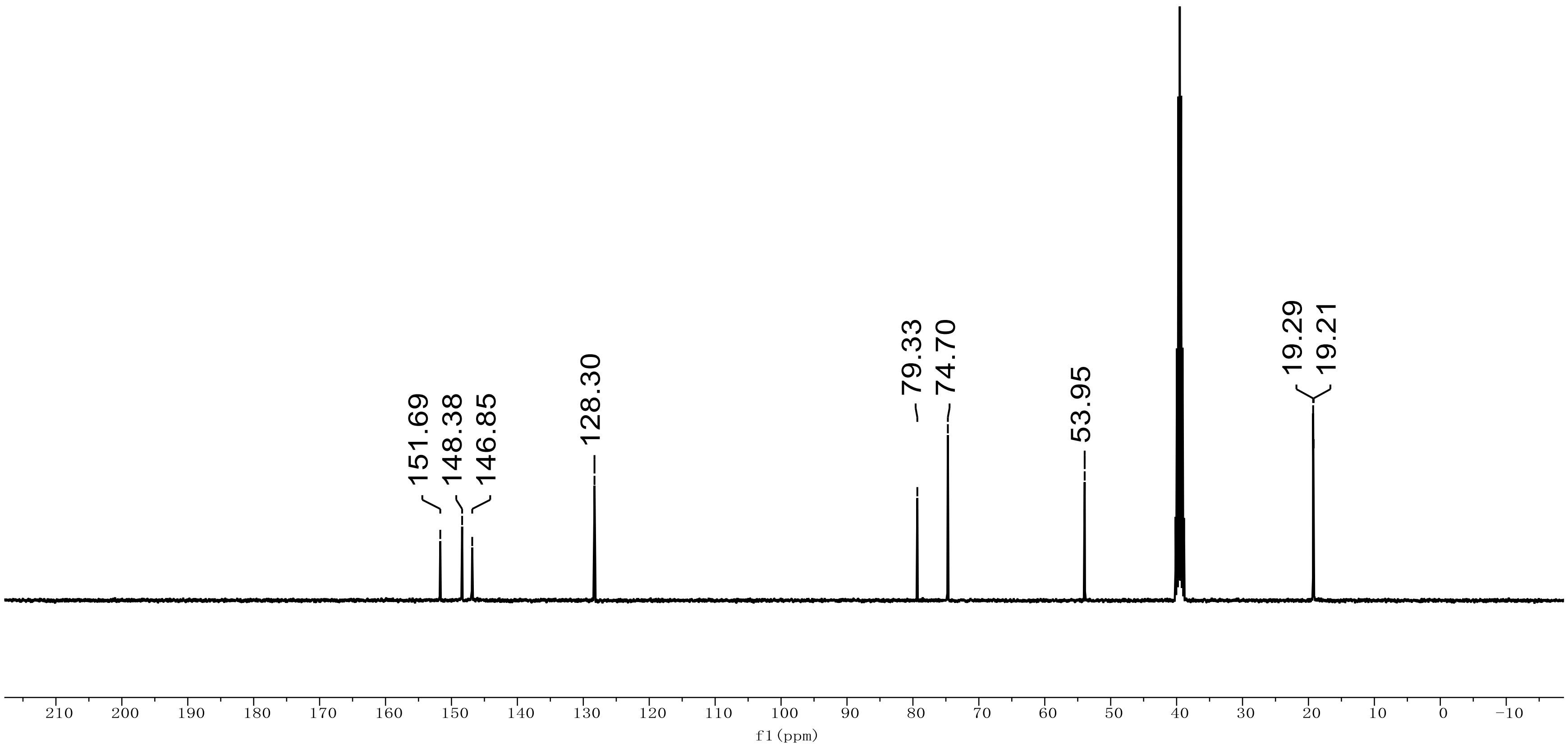

^{1}H NMR of 1e

DMSO-d_6, 400 MHz, 25 °C

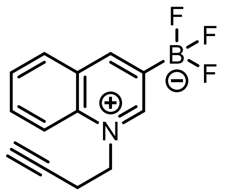

1e

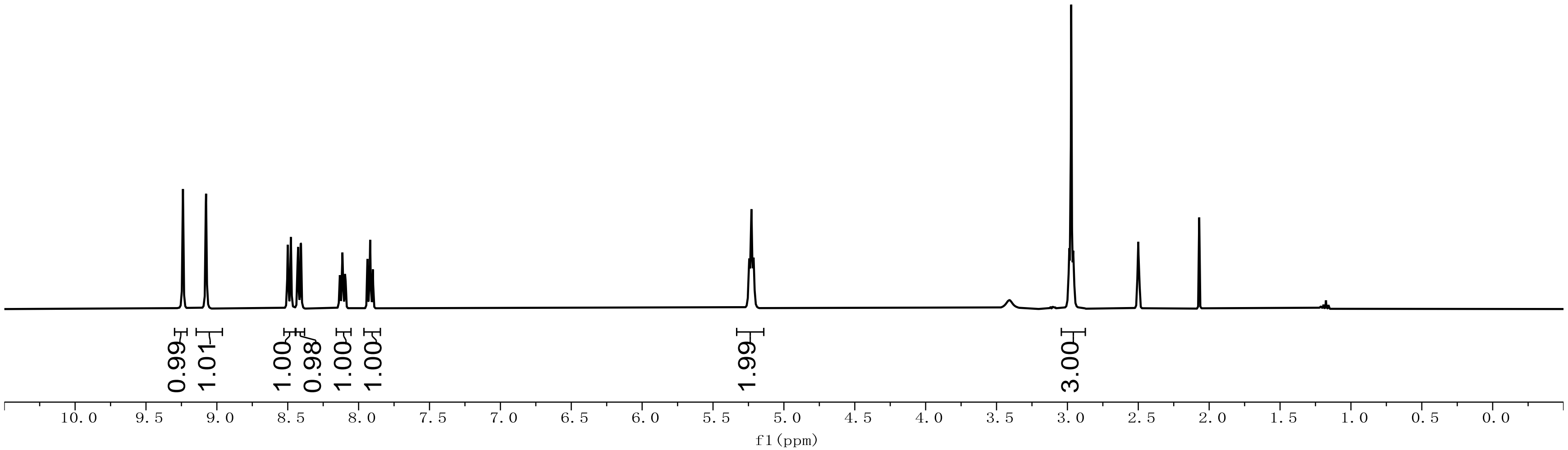

^{19}F NMR of 1e

CD_3CN, 376 MHz, 25 °C

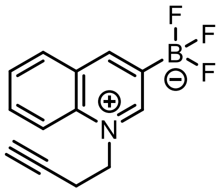

1e

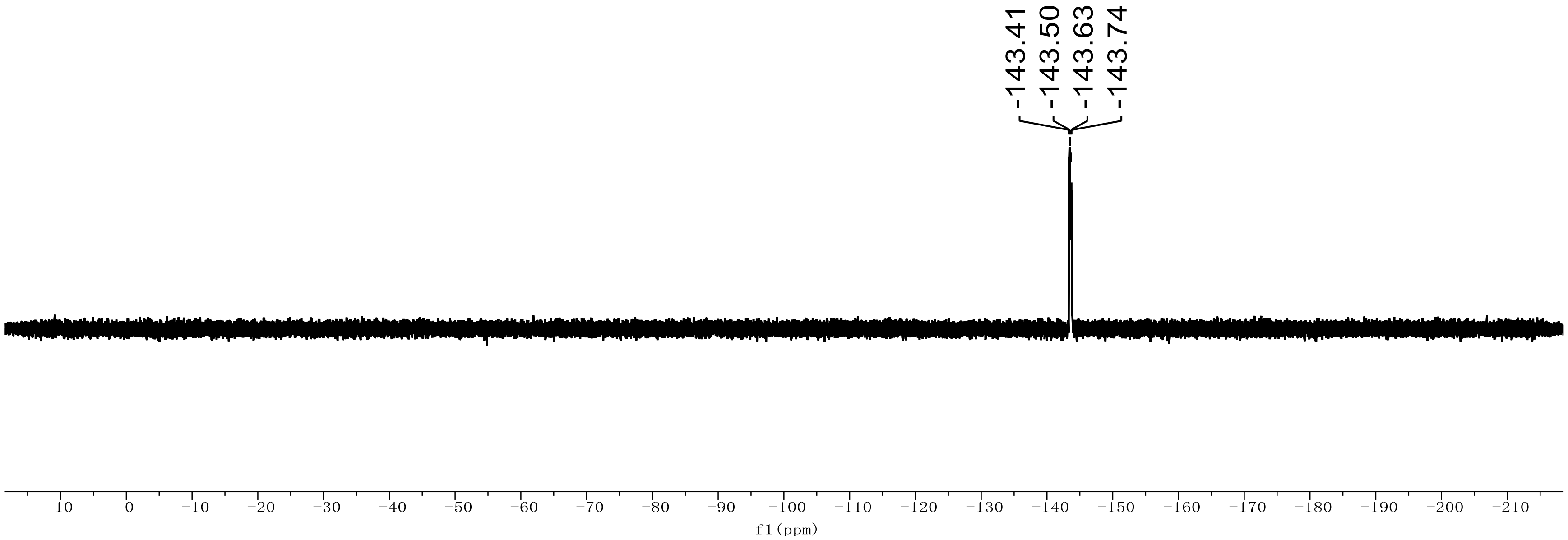

^{11}B NMR of 1e

CD_3CN, 128 MHz, 25 °C

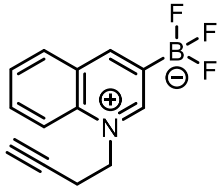

1e

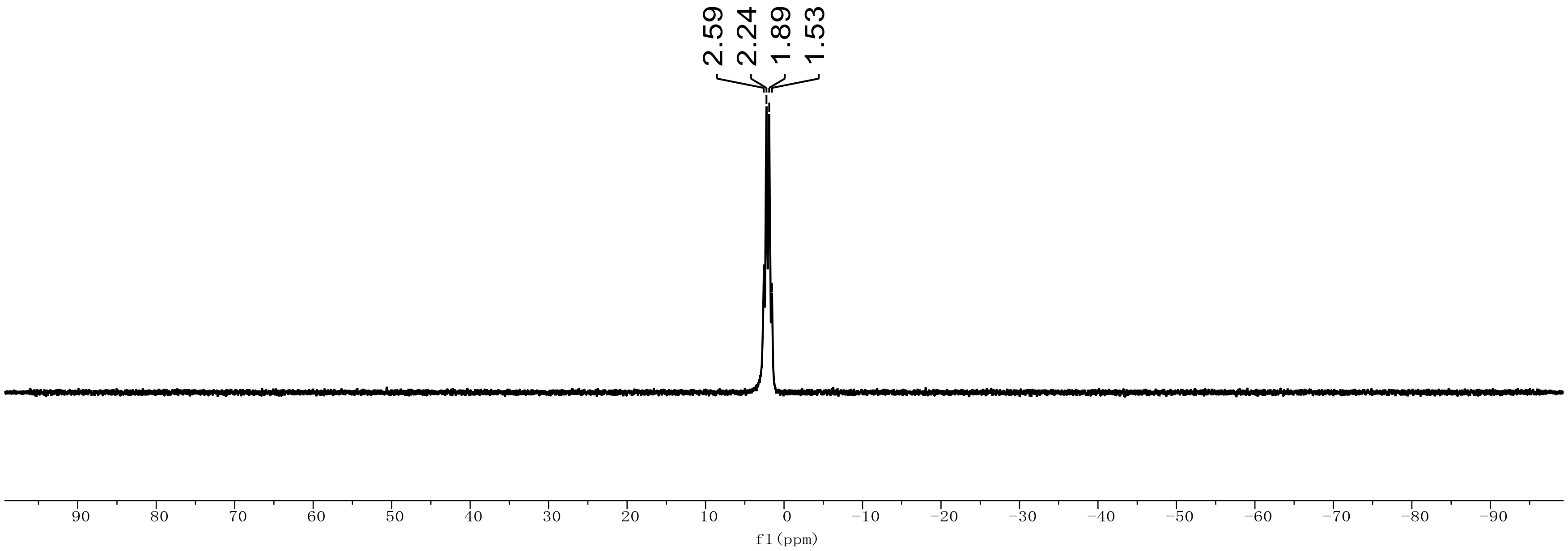

^{13}C NMR of 1e

DMSO-d_6, 101 MHz, 25 °C

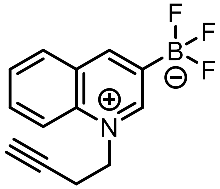

1e

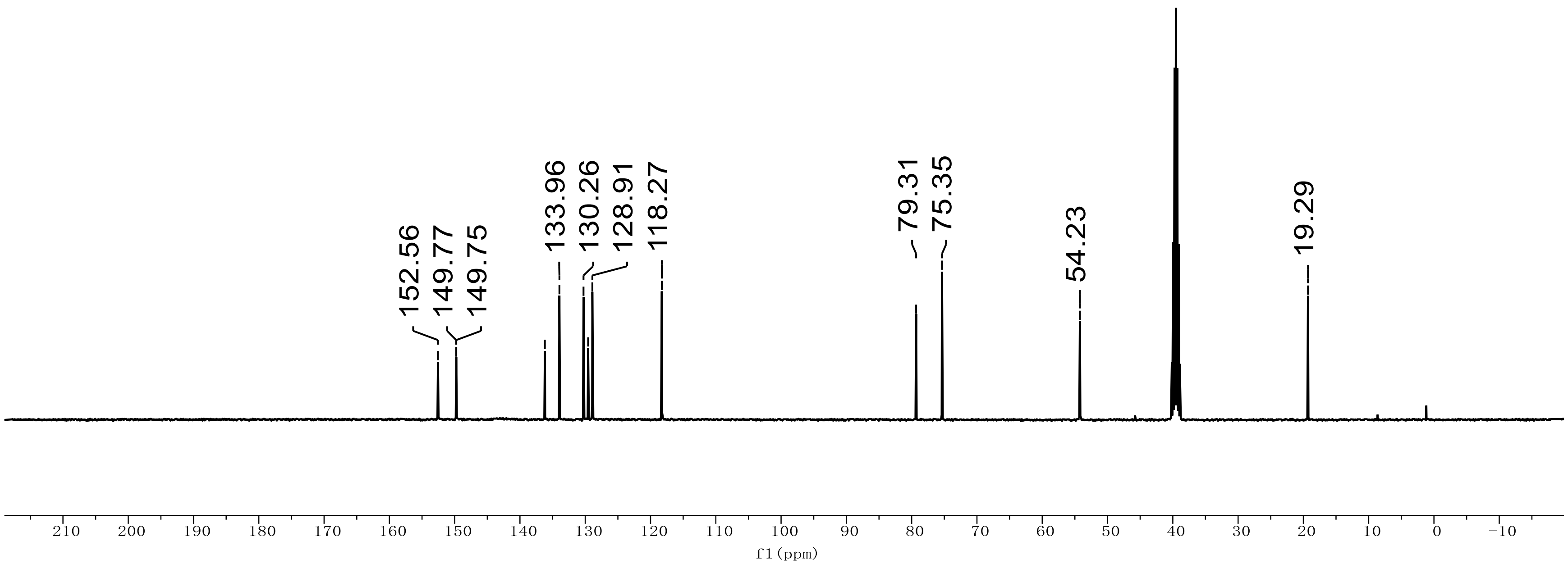

^{1}H NMR of 1f

CD_3CN, 400 MHz, 25 °C

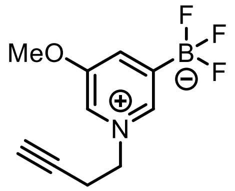

1f

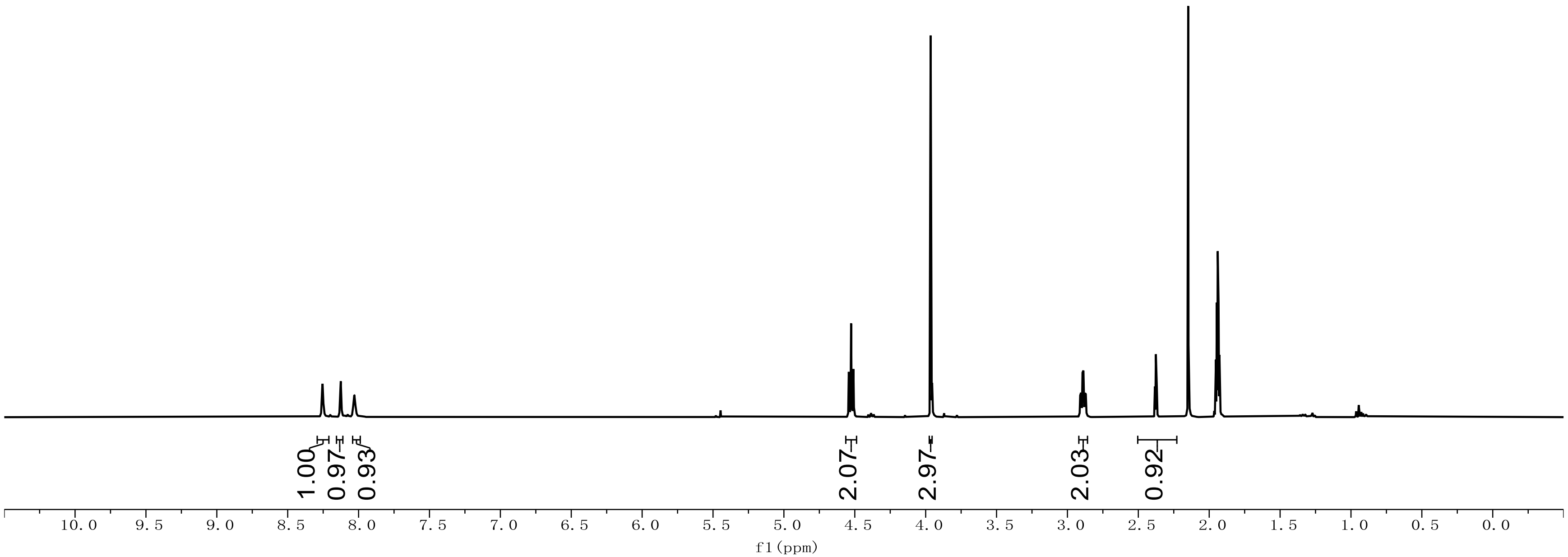

^{19}F NMR of 1f

CD_3CN, 376 MHz, 25 °C

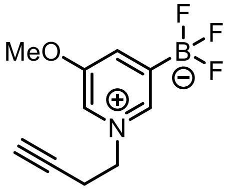

1f

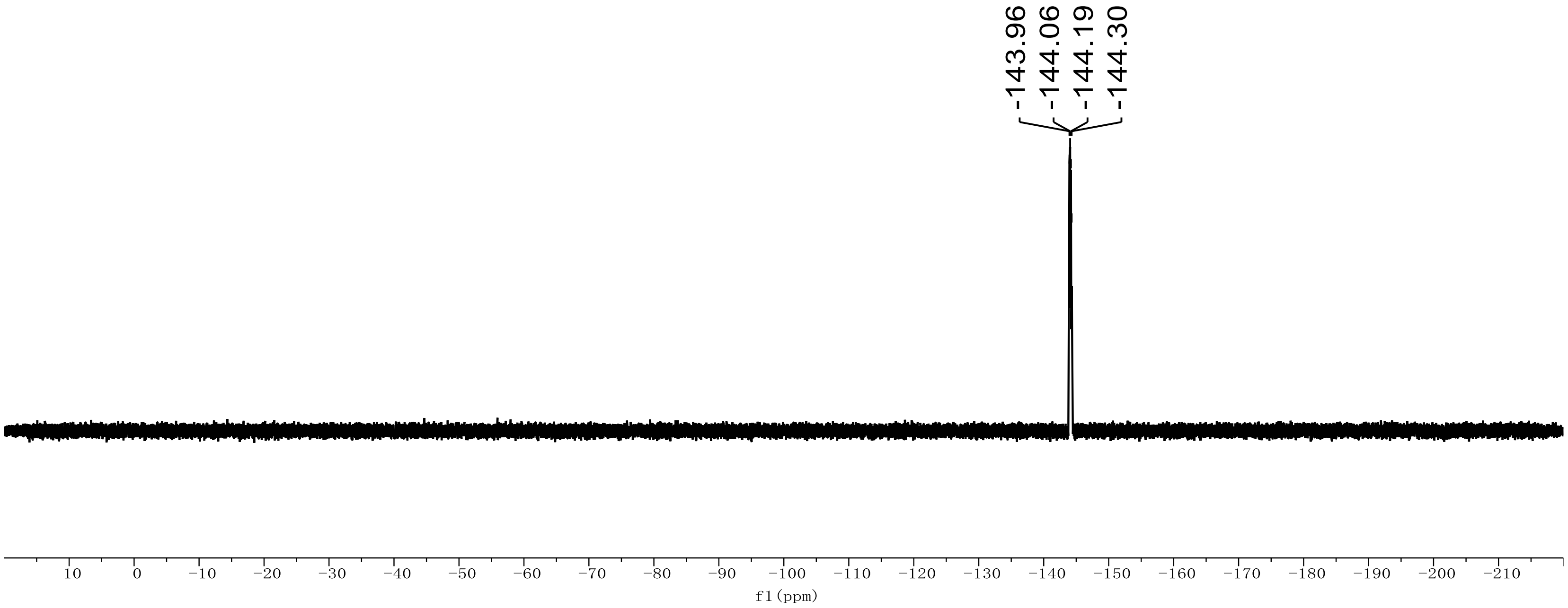

^{11}B NMR of 1f

CD_3CN, 128 MHz, 25 °C

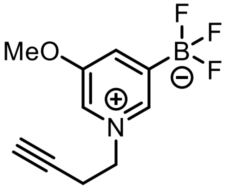

1f

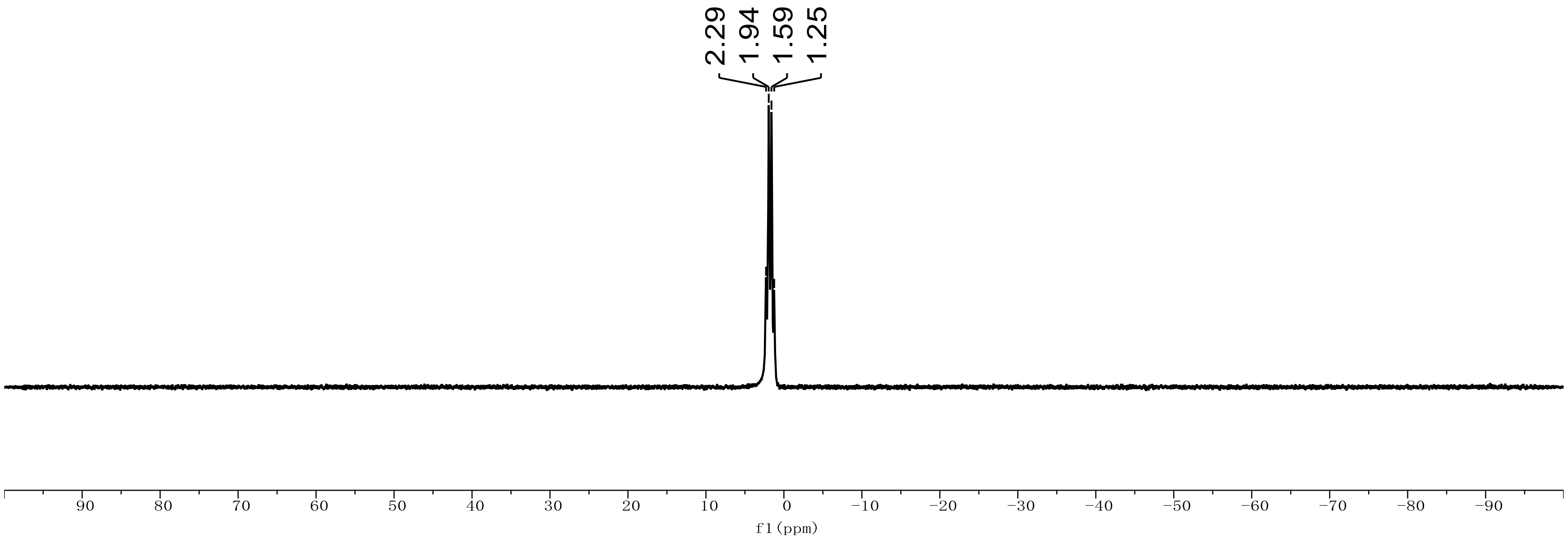

^{13}C NMR of 1f

DMSO-d_6, 101 MHz, 25 °C

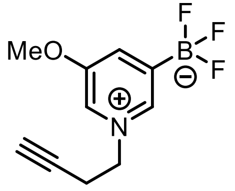

1f

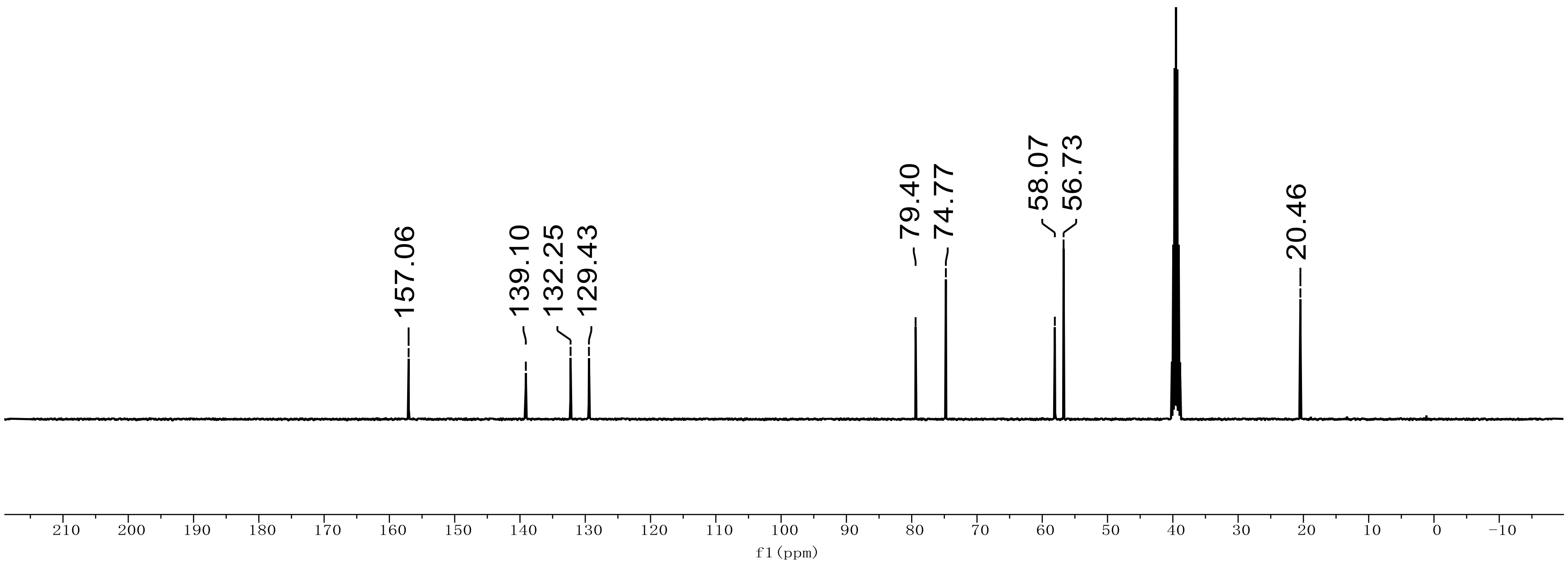

^{1}H NMR of 1g

CD_3CN, 400 MHz, 25 °C

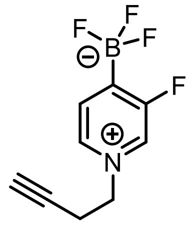

1g

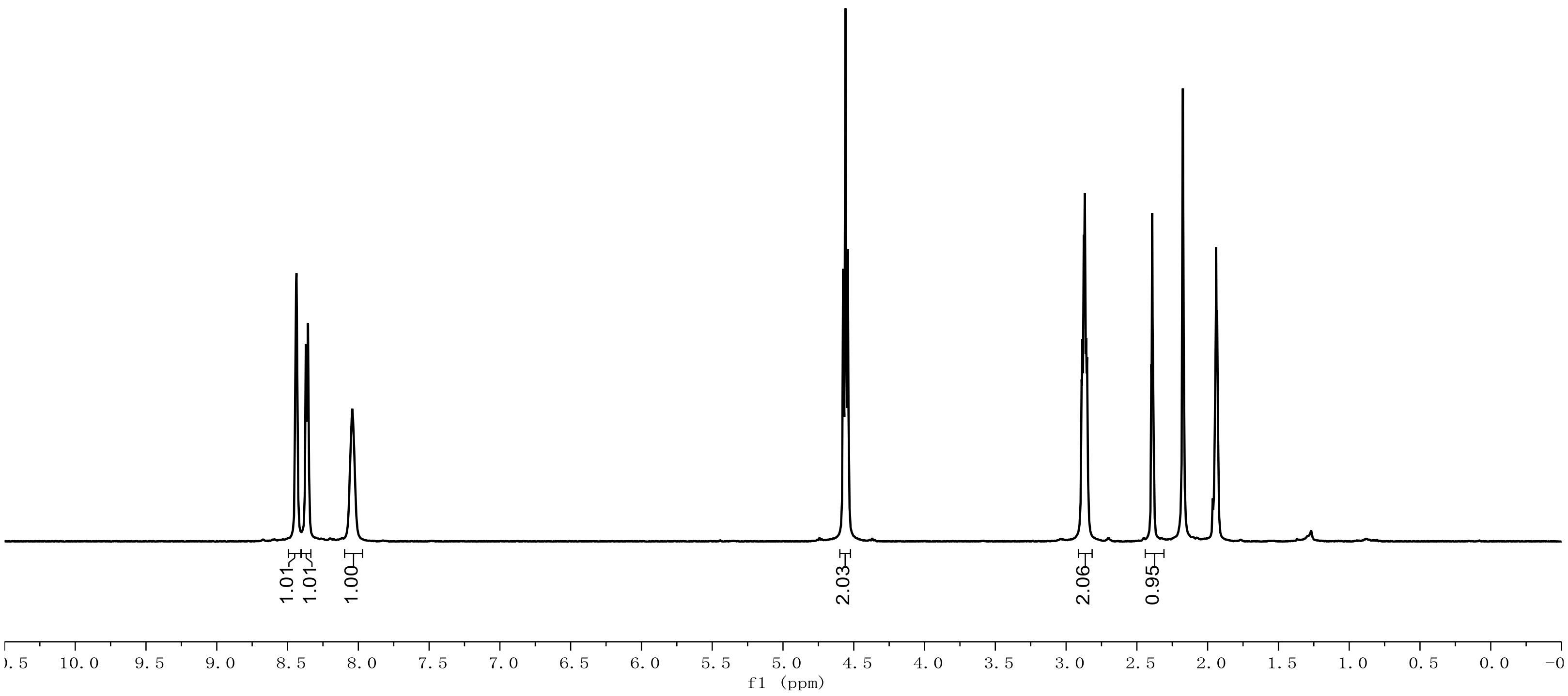

^{19}F NMR of 1g

CD_3CN, 376 MHz, 25 °C

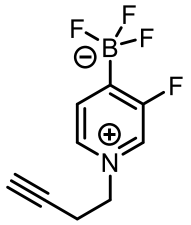

1g

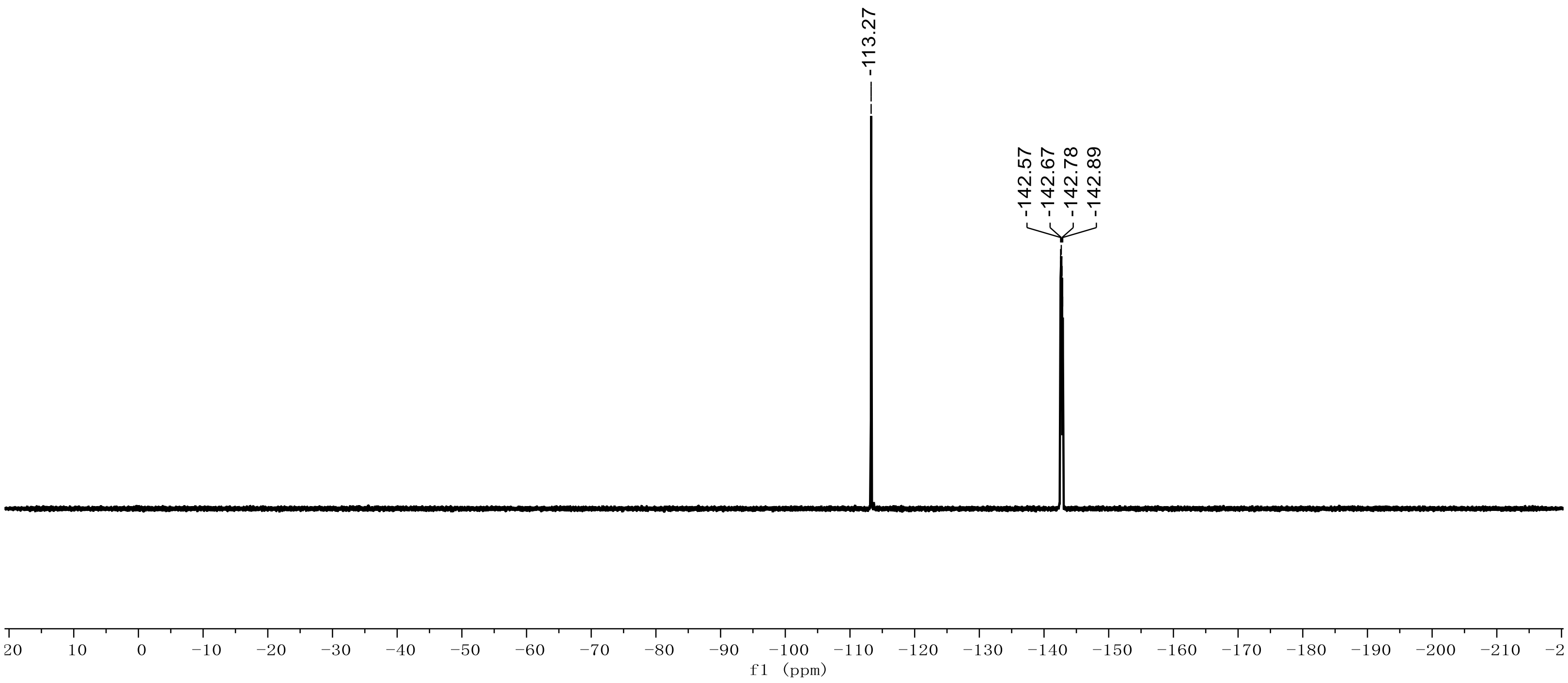

^{11}B NMR of 1g

DMSO-d_6, 128 MHz, 25 °C

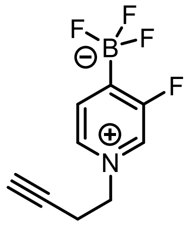

1g

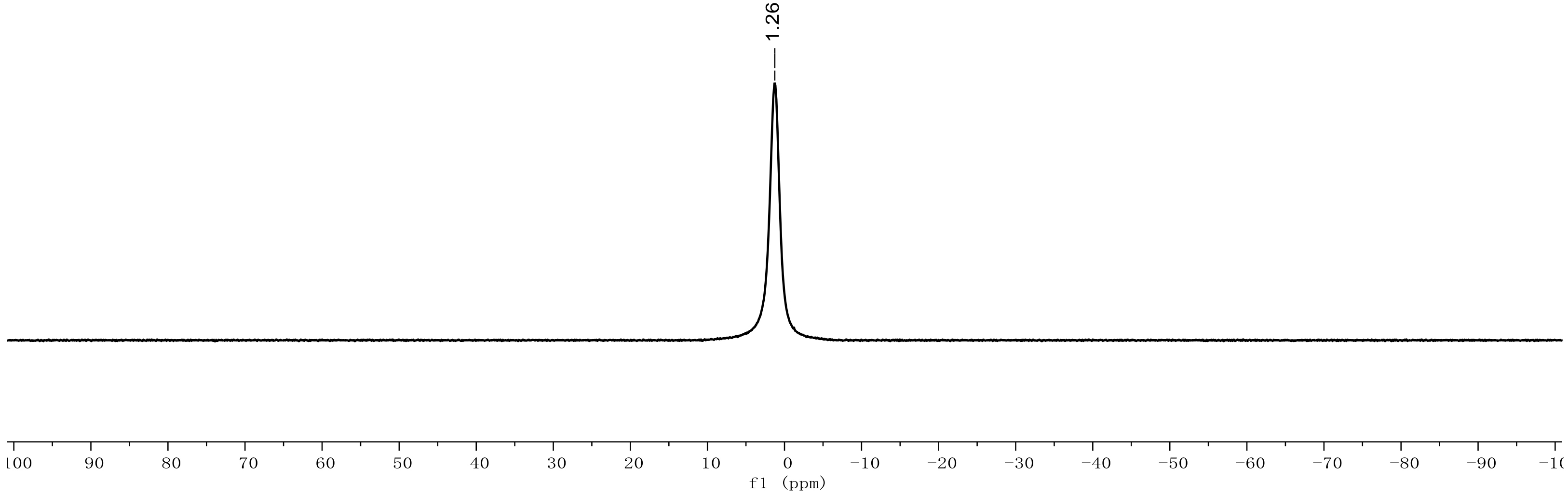

^{13}C NMR of 1g

DMSO-d_6, 101 MHz, 25 °C

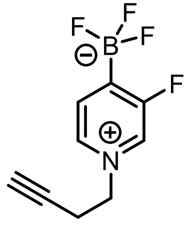

1g

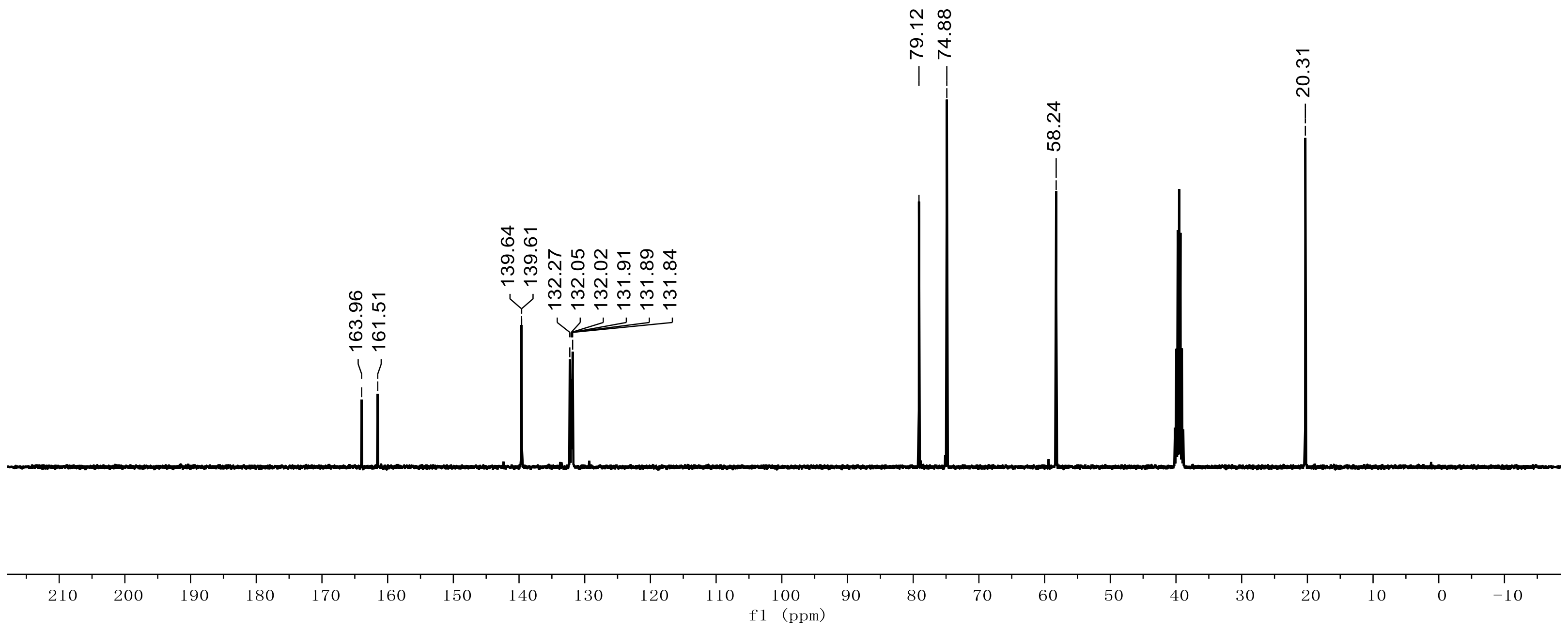

^{1}H NMR of 1h

CD_3CN, 400 MHz, 25 °C

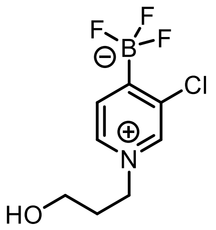

1h

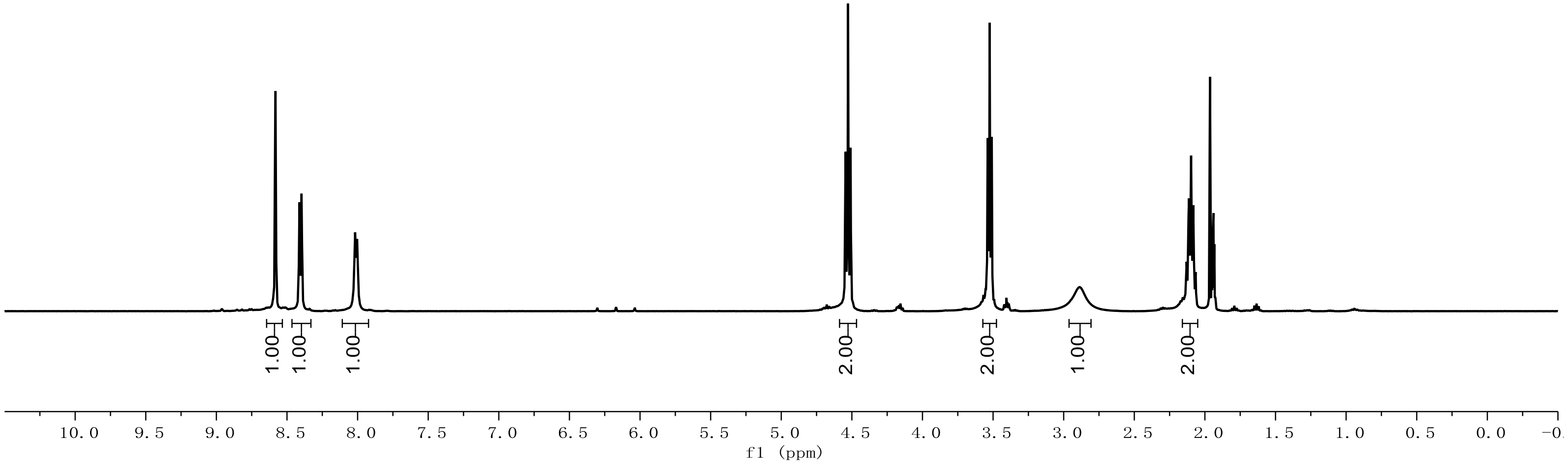

^{19}F NMR of 1h

CD_3CN, 376 MHz, 25 °C

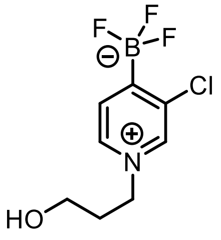

1h

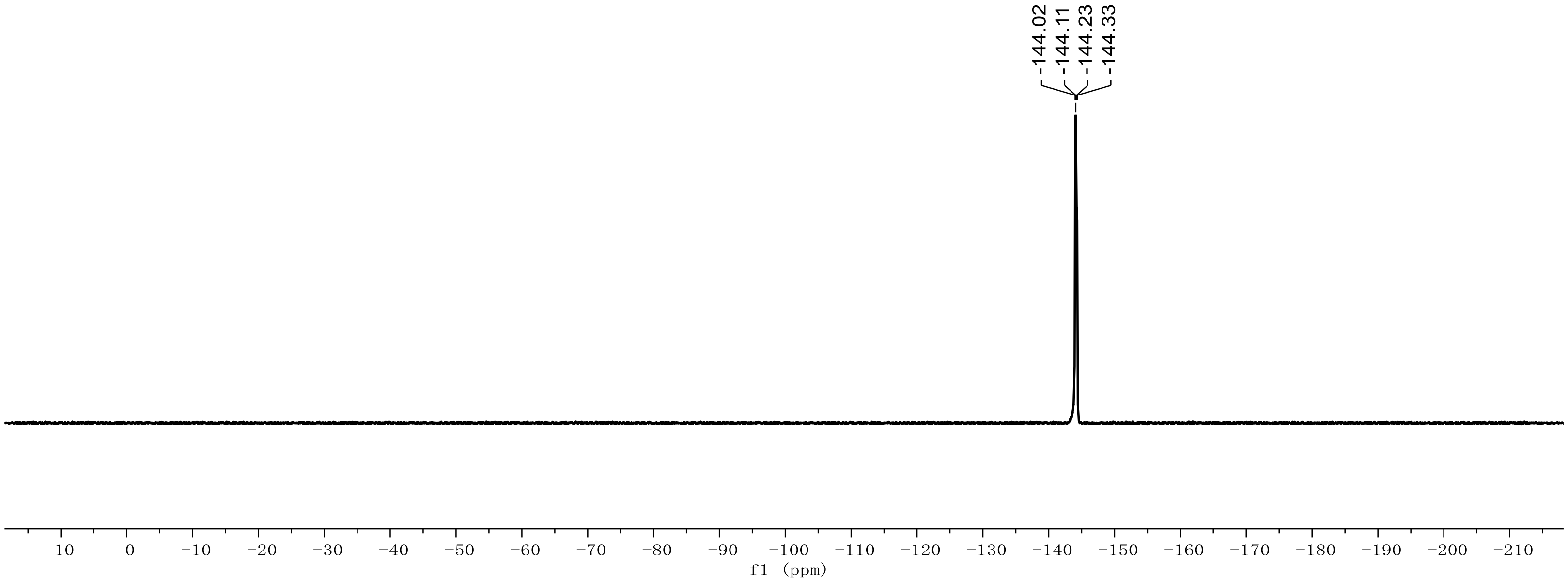

^{11}B NMR of 1h

CD_3CN, 128 MHz, 25 °C

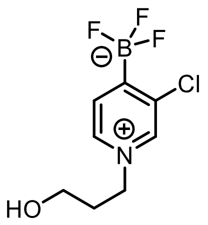

1h

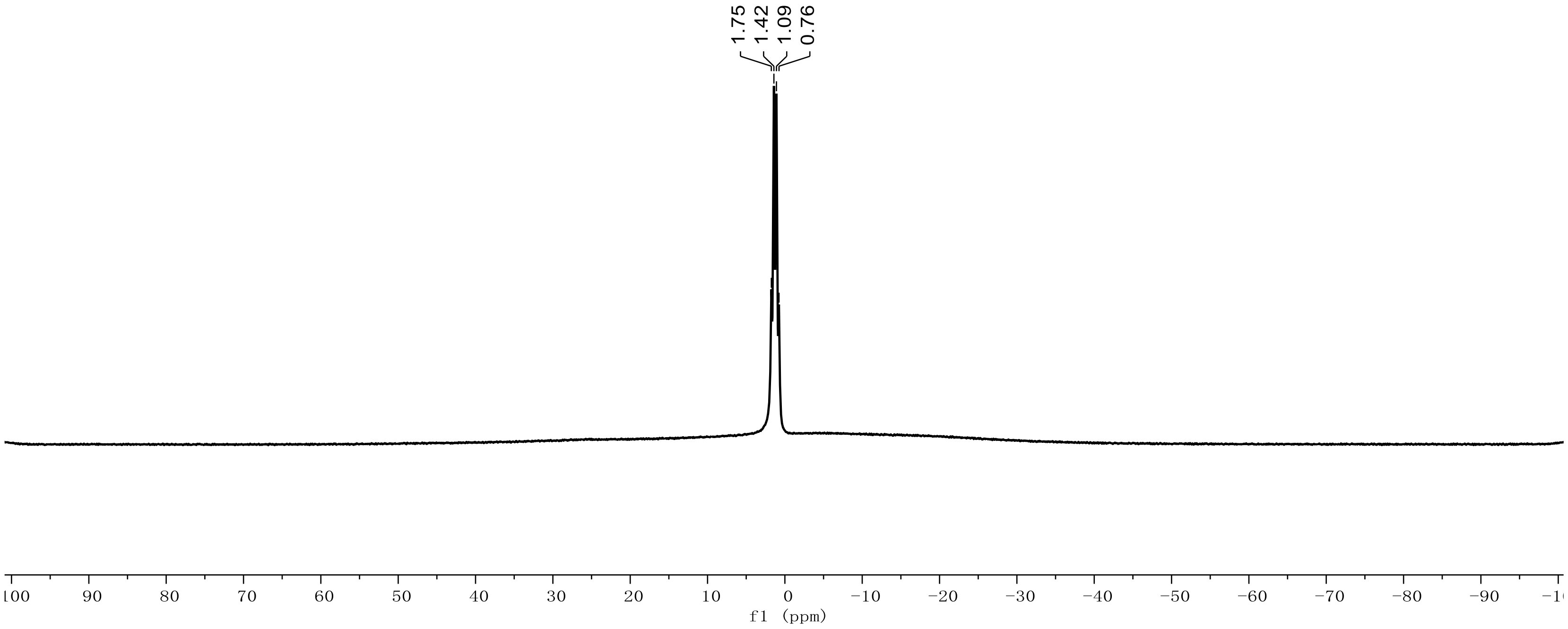

^{13}C NMR of 1h

CD_3CN, 101 MHz, 25 °C

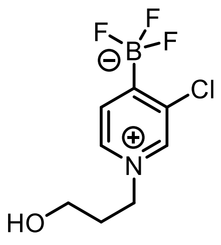

1h

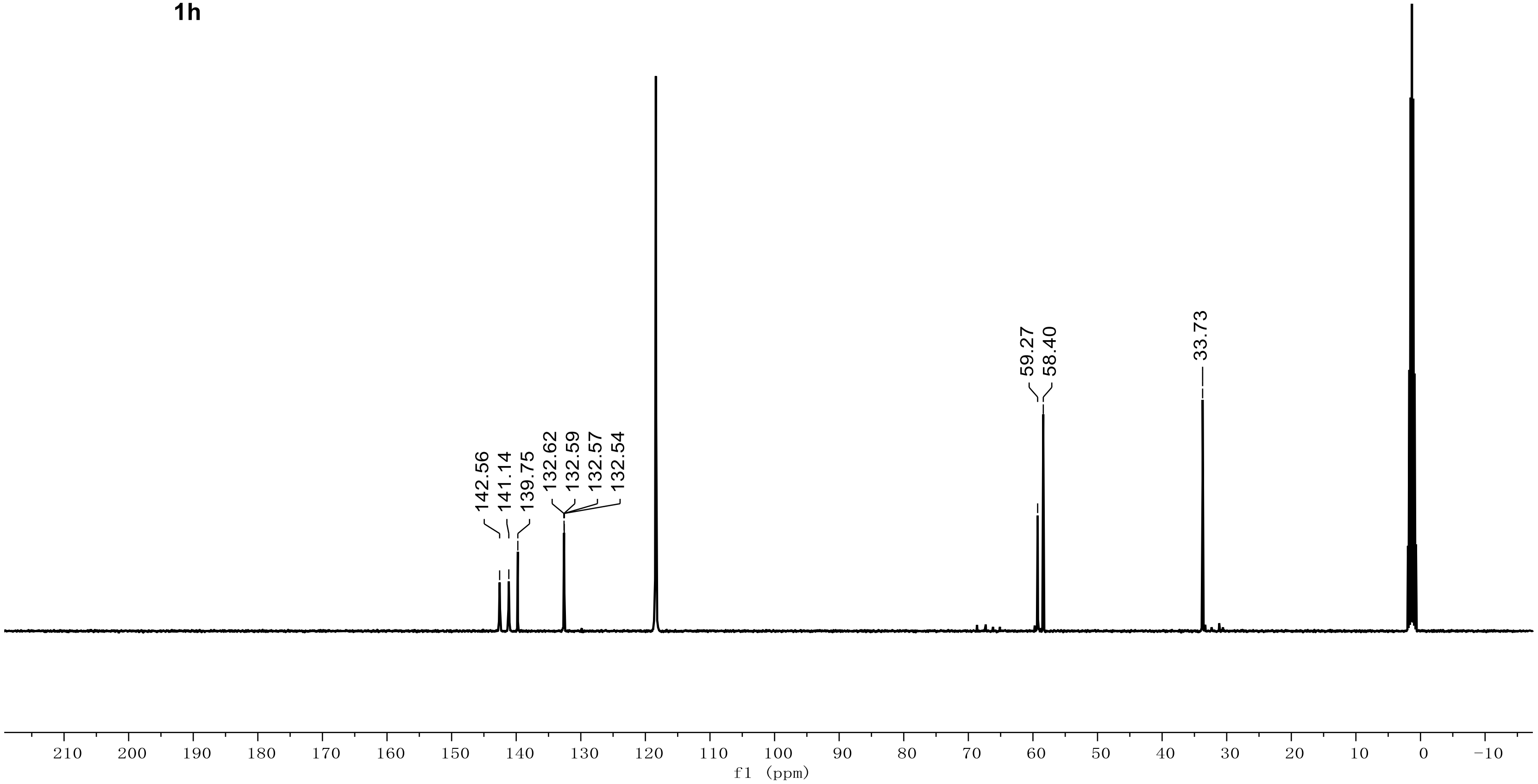

^{1}H NMR of 1i

CD$_3$CN, 400 MHz, 25 °C

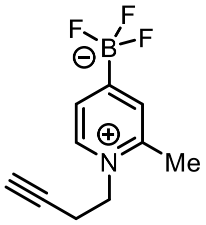

1i

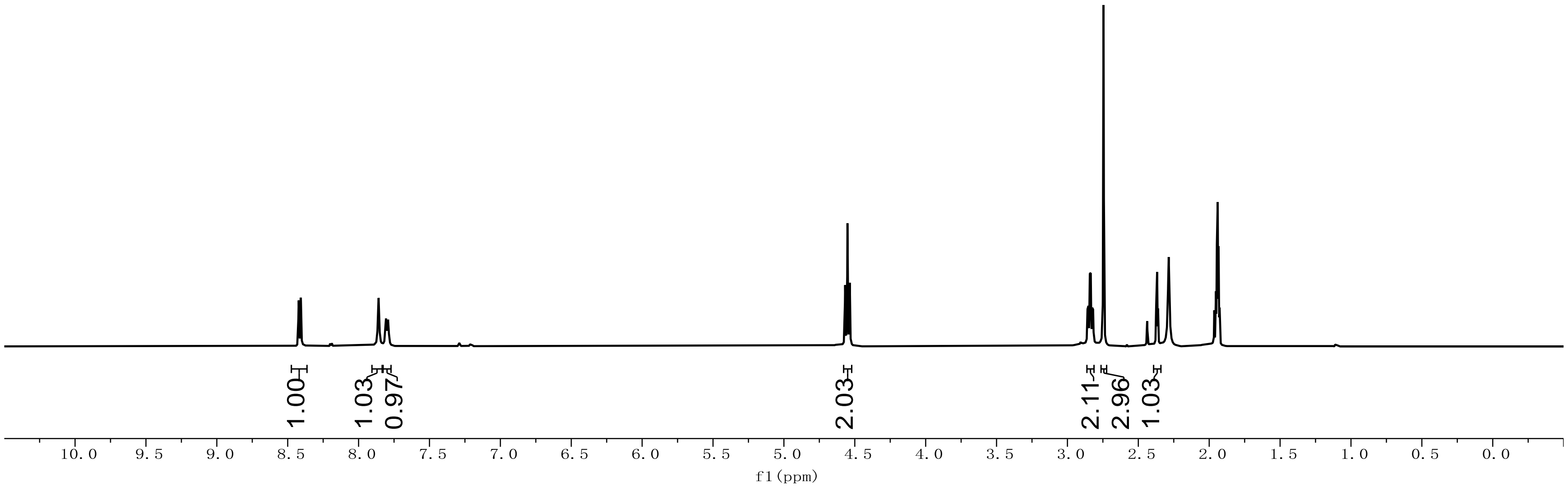

^{19}F NMR of 1i

CD_3CN, 376 MHz, 25 °C

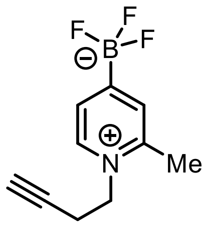

1i

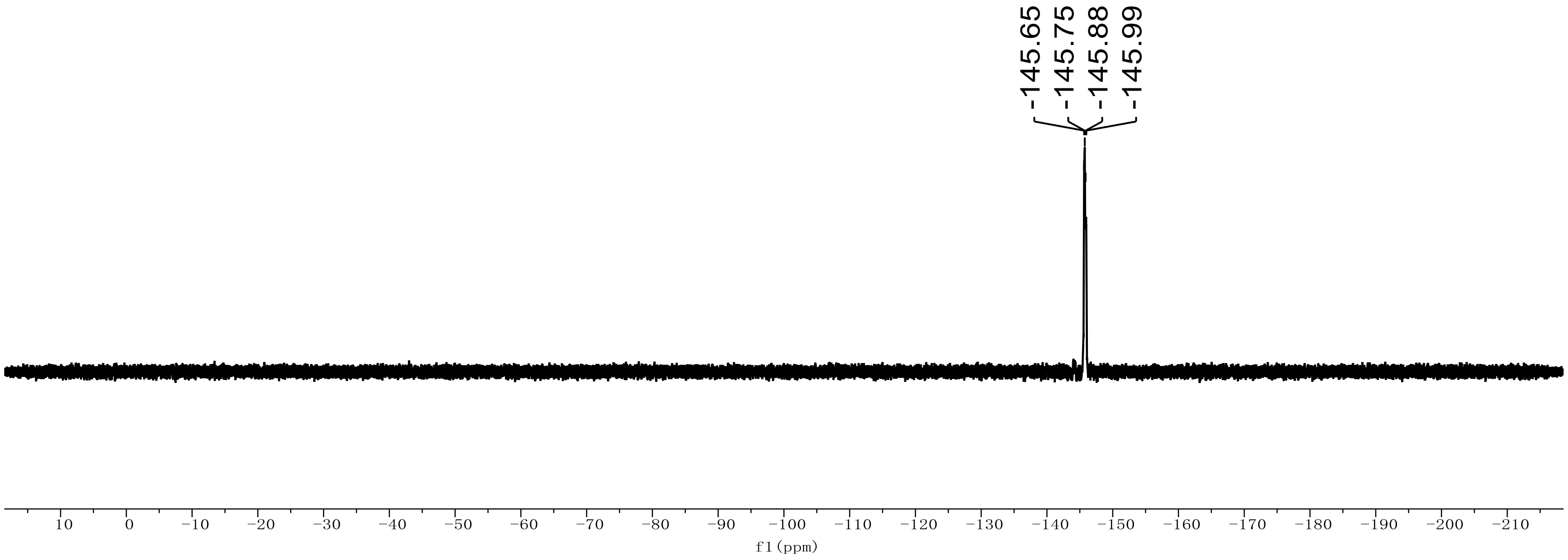

^{11}B NMR of 1i

CD_3CN, 128 MHz, 25 °C

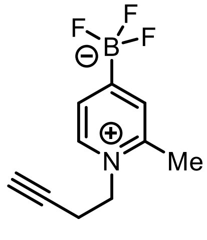

1i

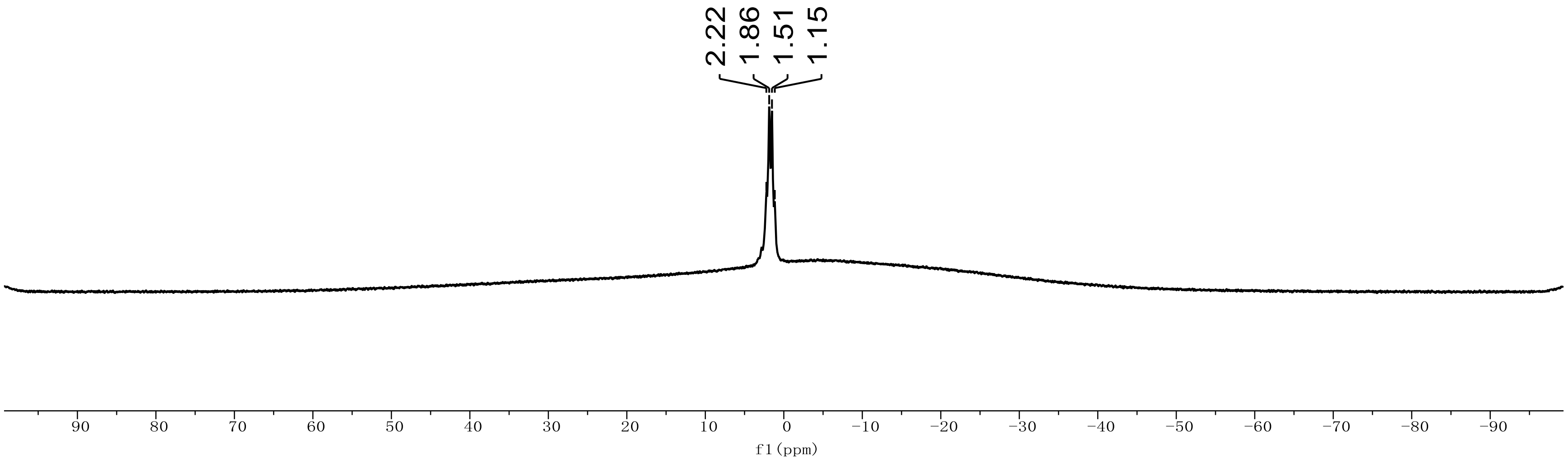

^{13}C NMR of 1i

DMSO-d_6, 101 MHz, 25 °C

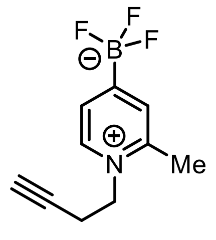

1i

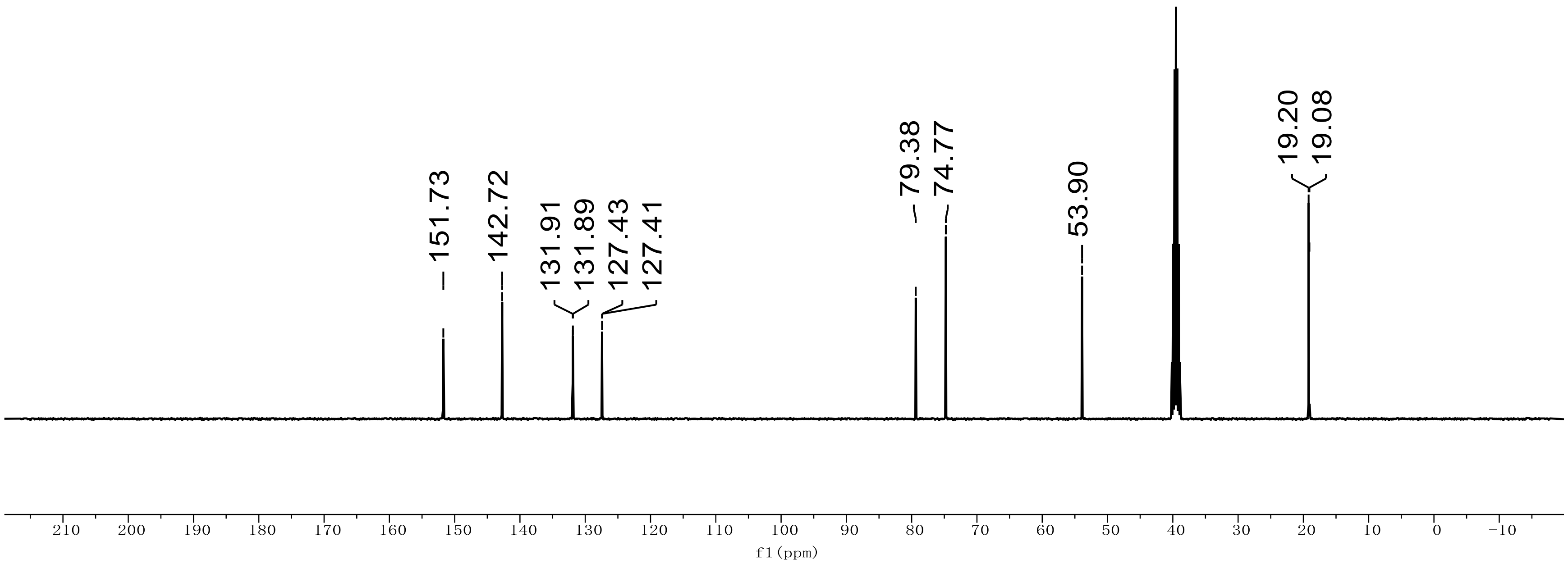

^{1}H NMR of 1j

CD_3CN, 400 MHz, 25 °C

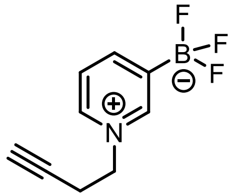

1j

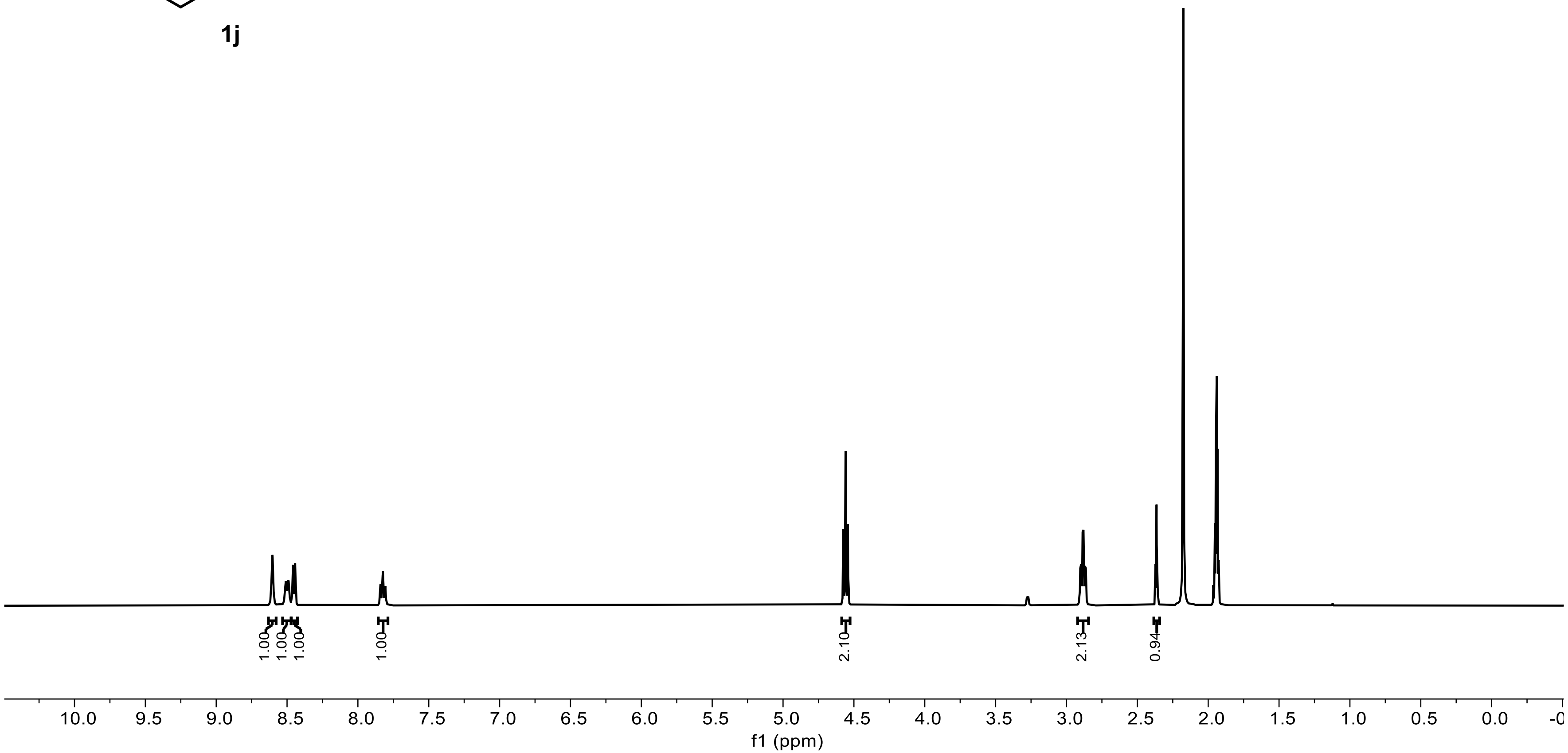

^{19}F NMR of 1j

CD_3CN, 376 MHz, 25 °C

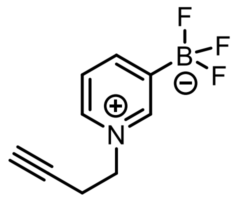

1j

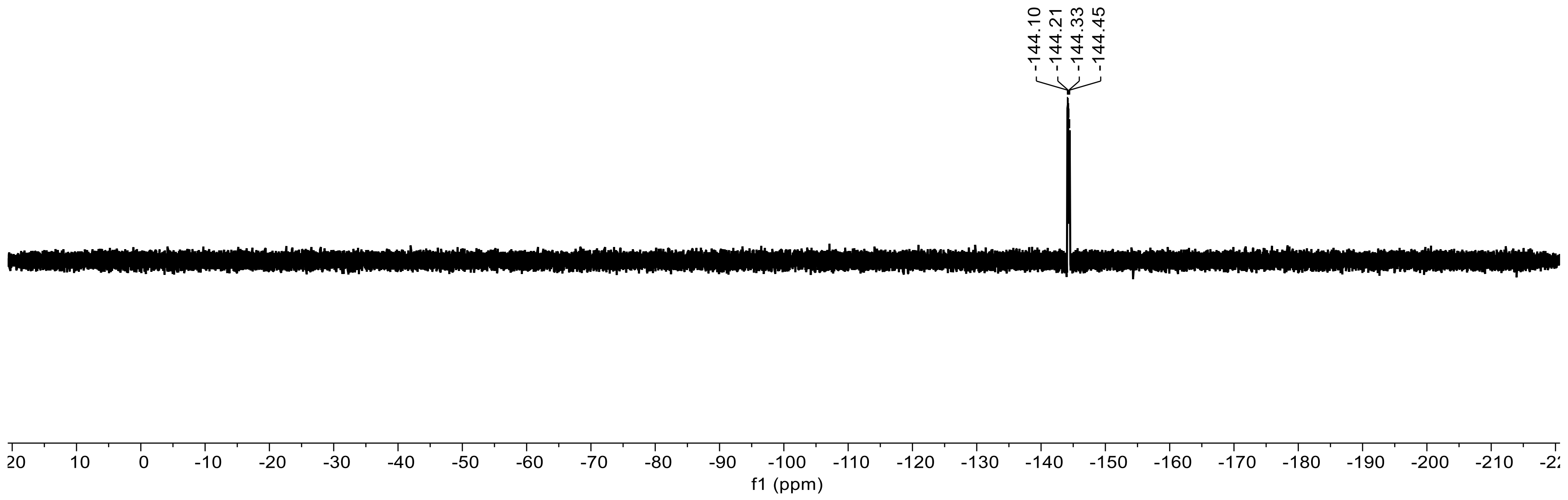

^{11}B NMR of 1j

CD_3CN, 128 MHz, 25 °C

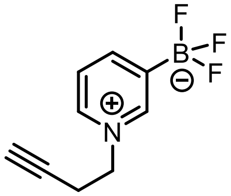

1j

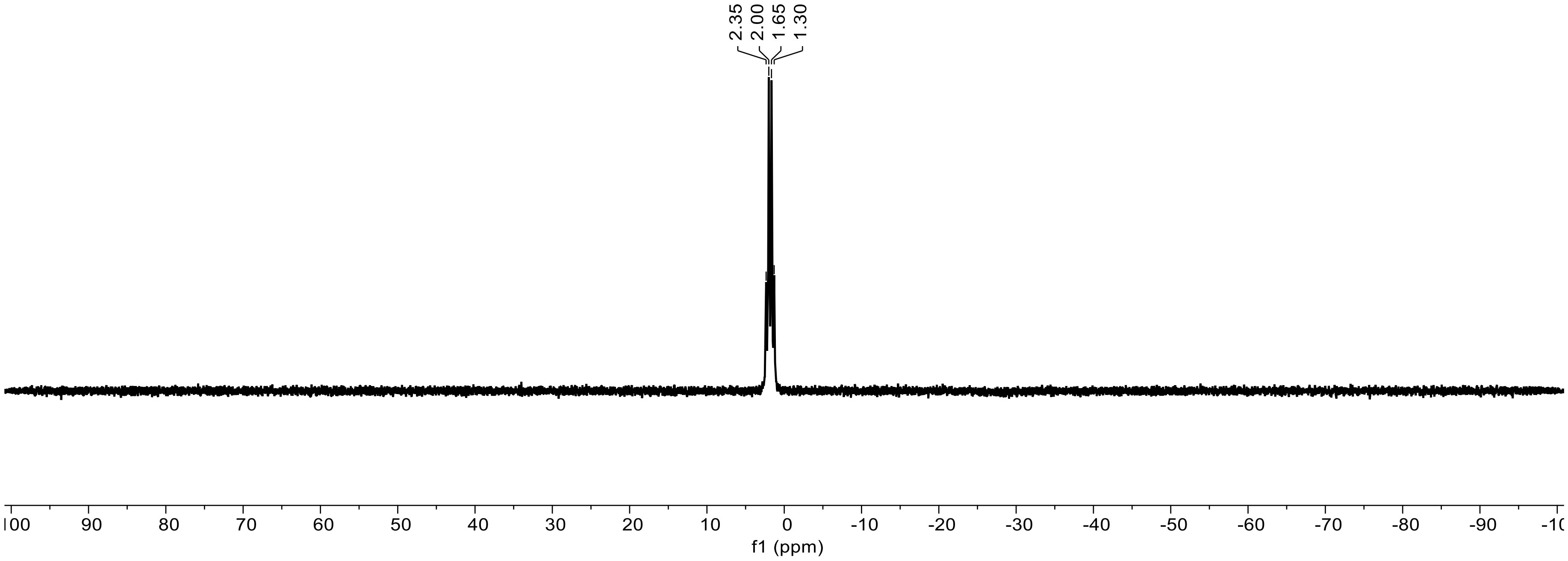

^{13}C NMR of 1j

CD_3CN, 101 MHz, 25 °C

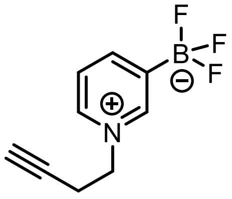

1j

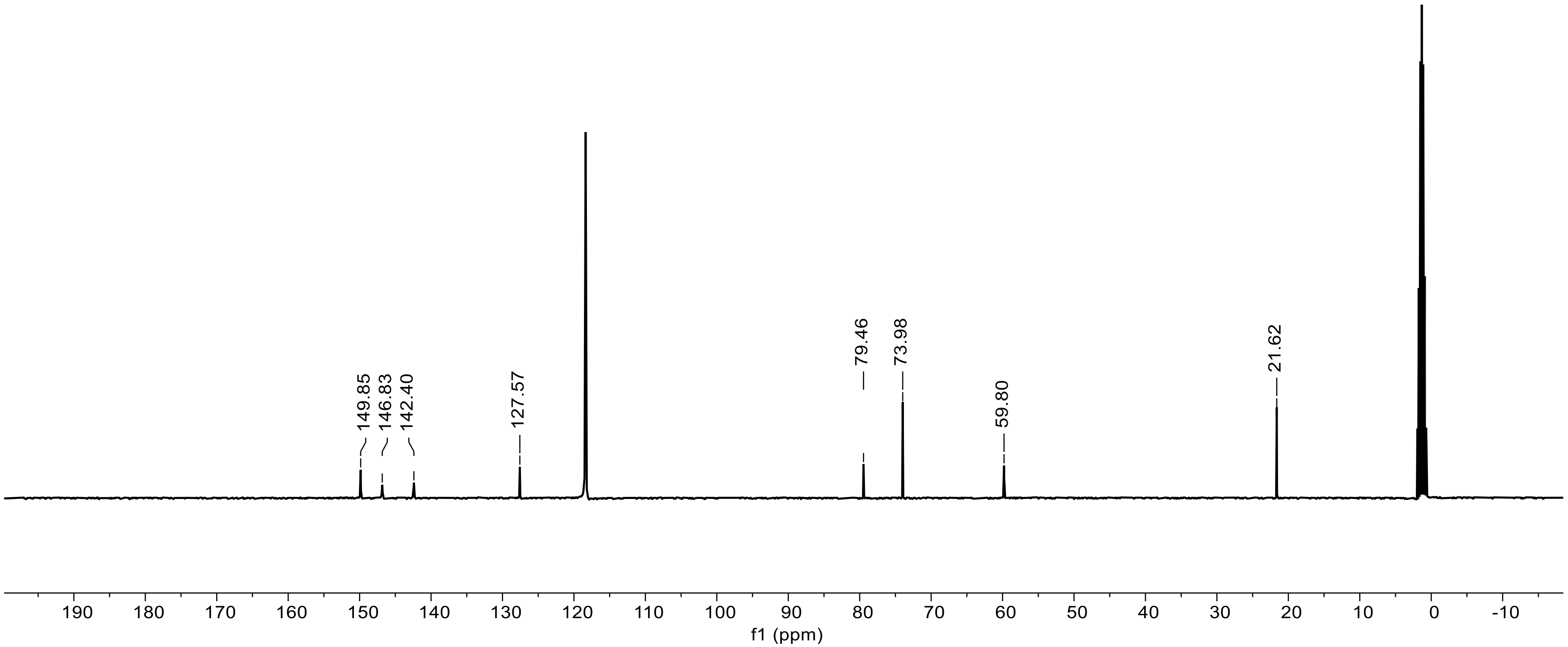

^{1}H NMR of 1k

CD_3CN, 400 MHz, 25 °C

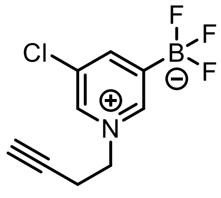

1k

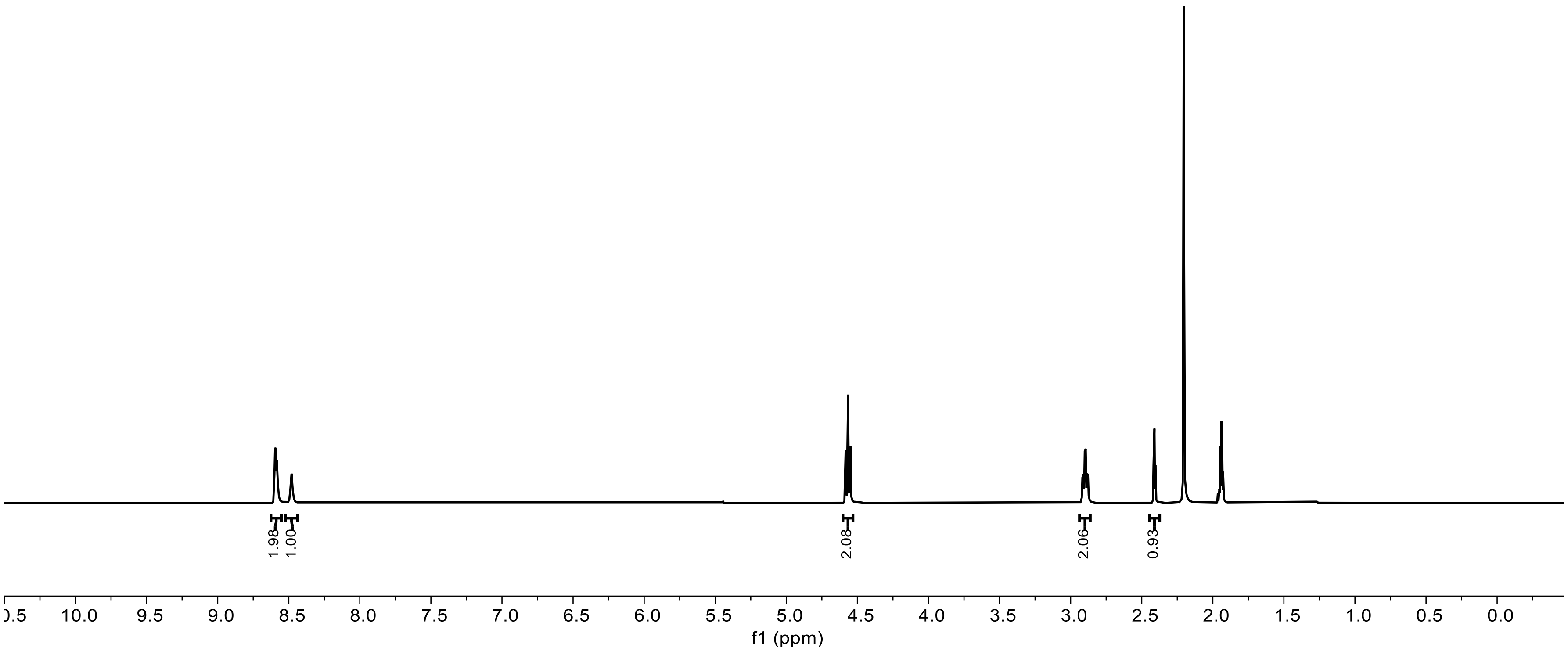

^{19}F NMR of 1k

CD_3CN, 376 MHz, 25 °C

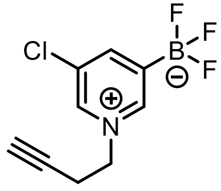

1k

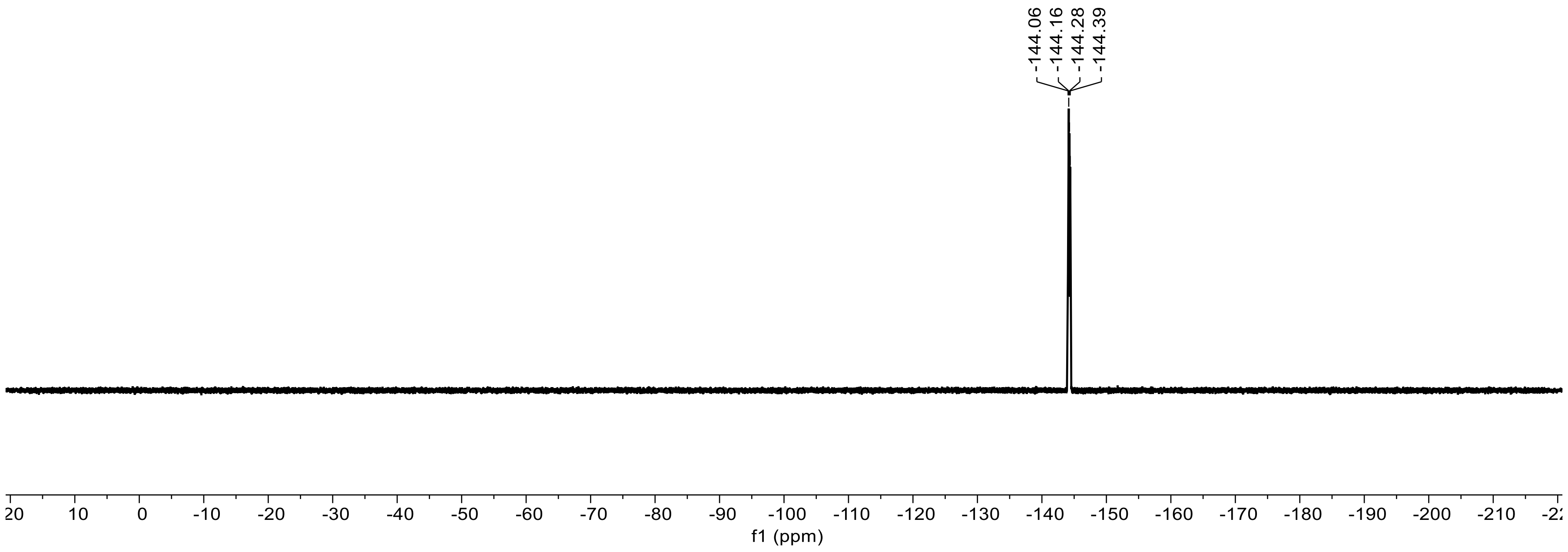

^{11}B NMR of 1k

CD_3CN, 128 MHz, 25 °C

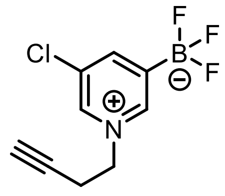

1k

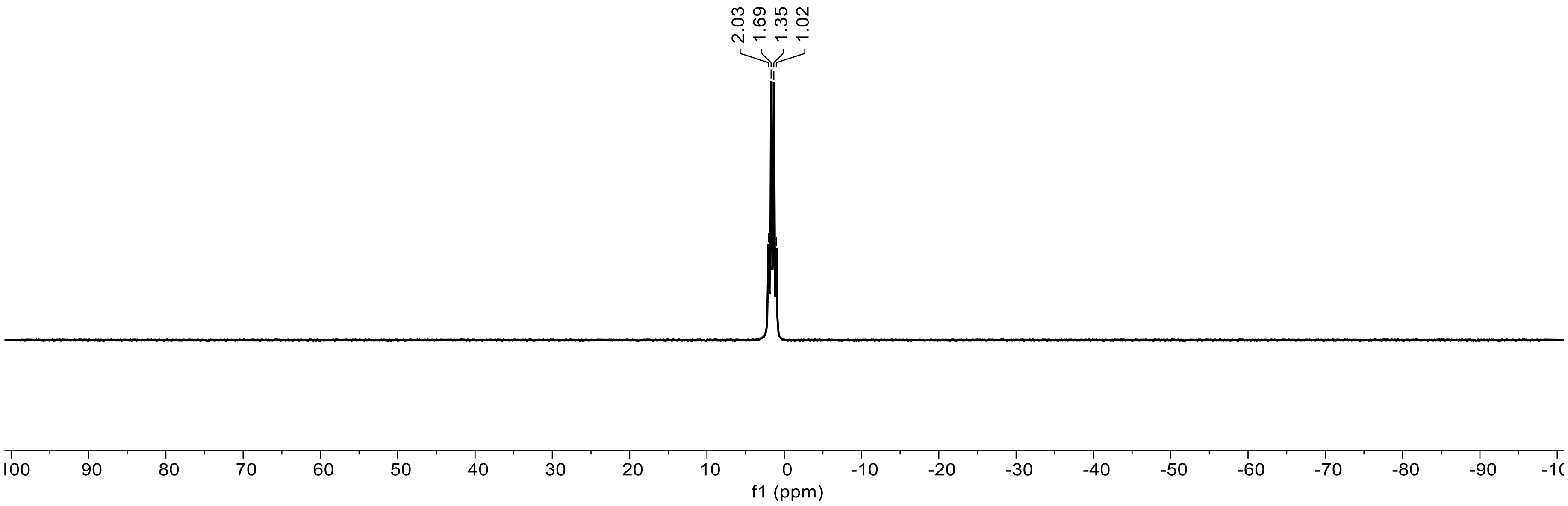

^{13}C NMR of 1k

DMSO-d_6, 101 MHz, 25 °C

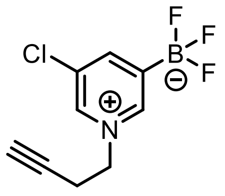

1k

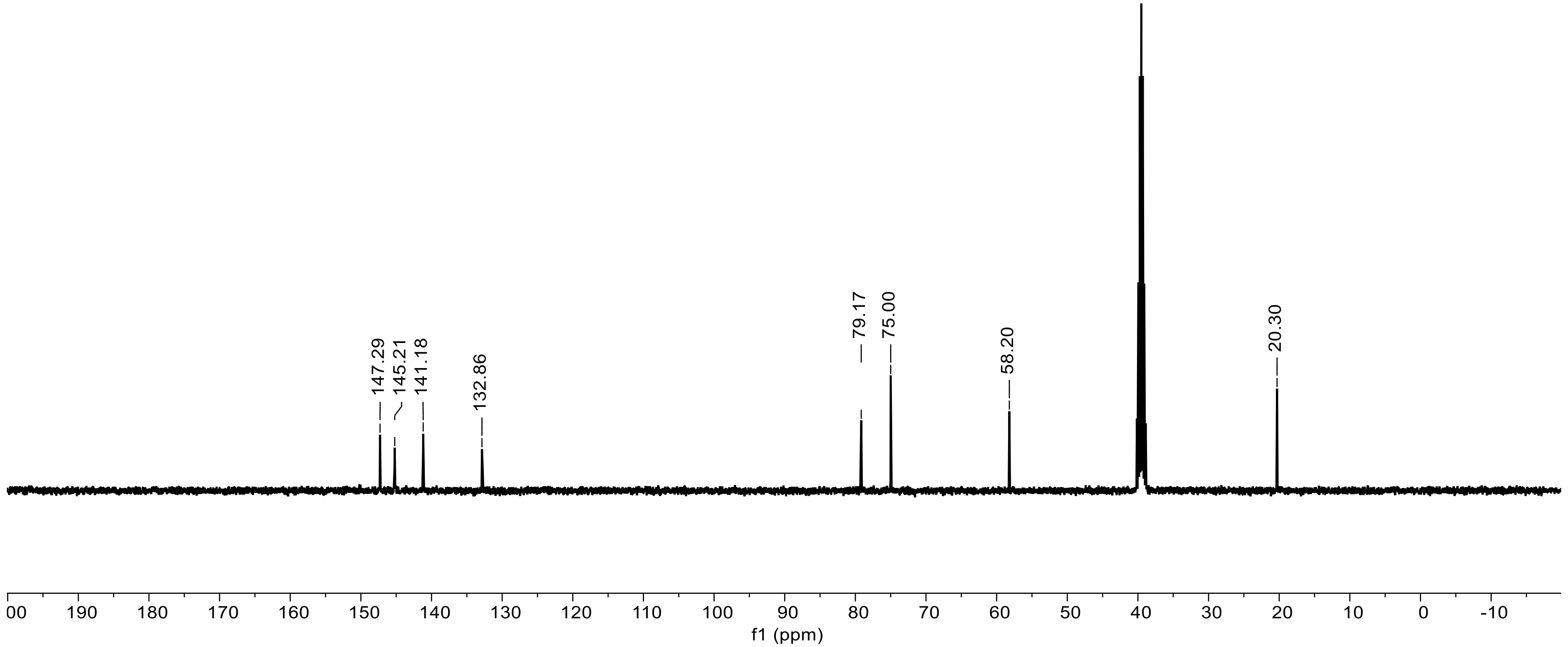

^{1}H NMR of 1l

CD_3CN, 400 MHz, 25 °C

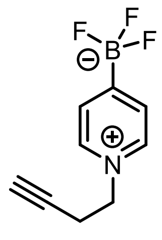

1l

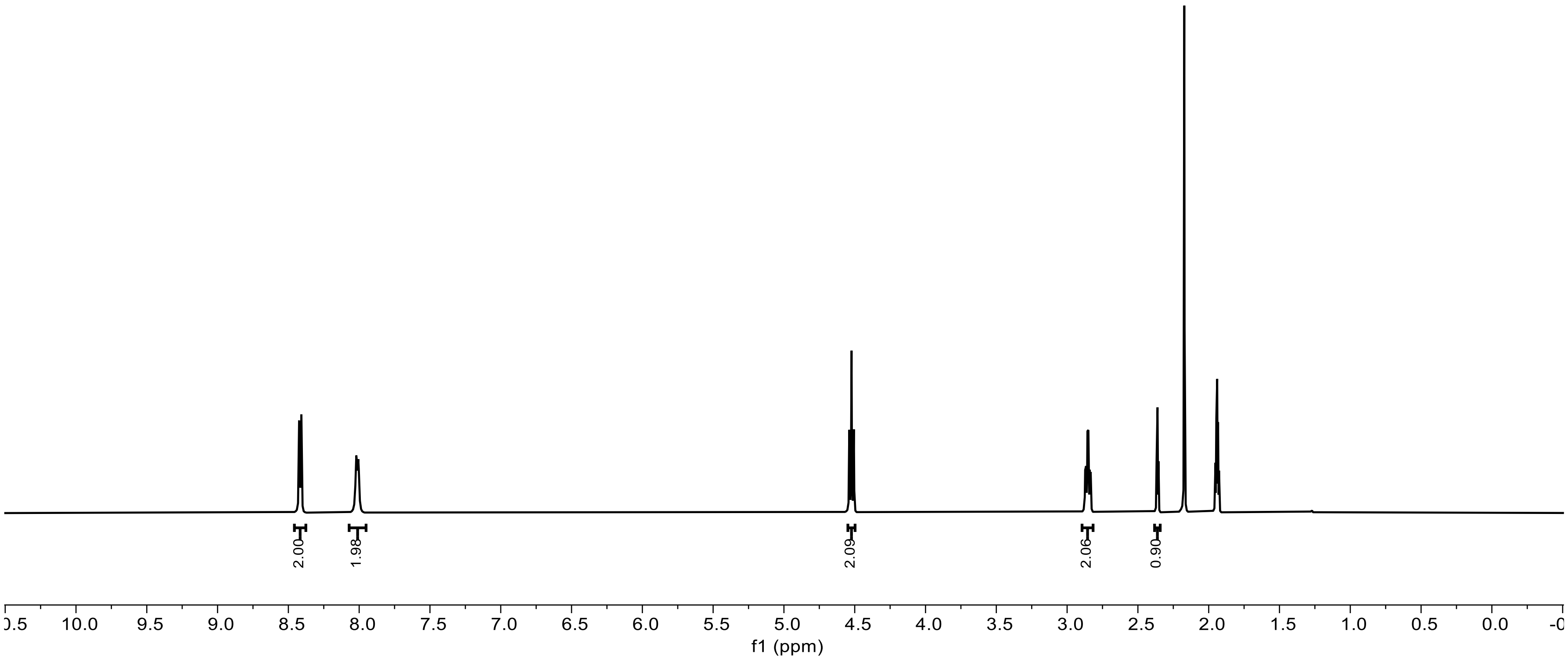

^{19}F NMR of 1l

CD_3CN, 376 MHz, 25 °C

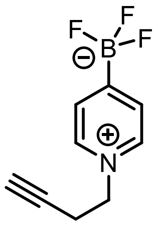

1l

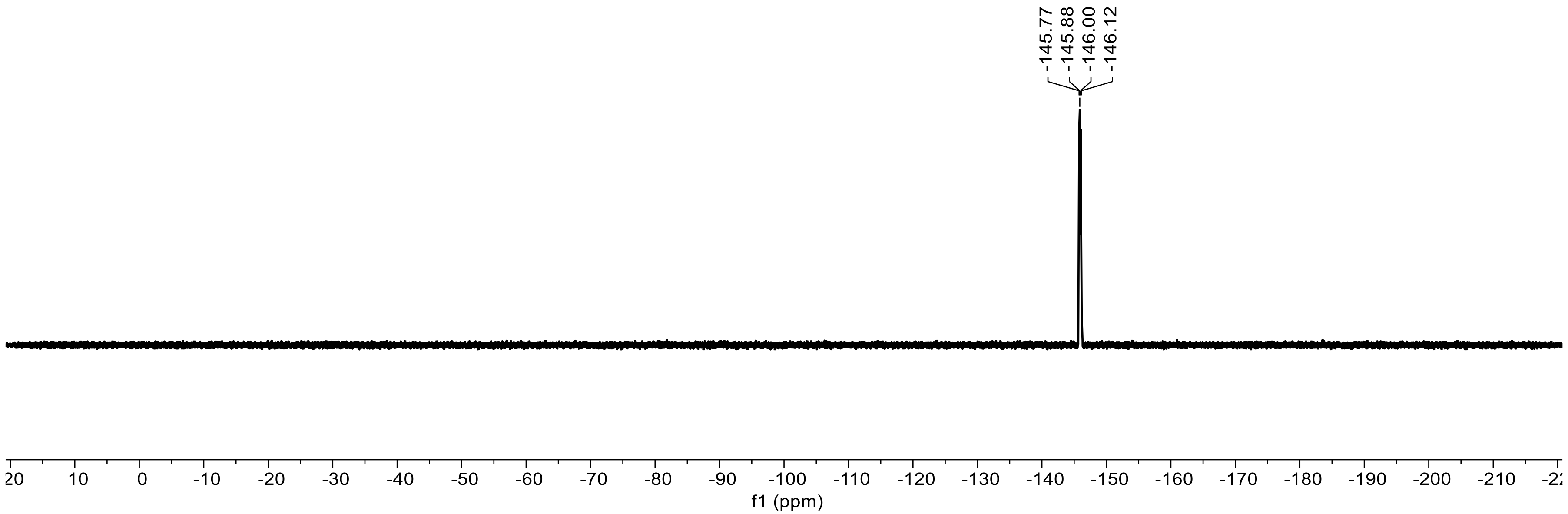

^{11}B NMR of 1l

CD_3CN, 128 MHz, 25 °C

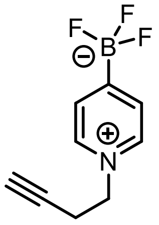

1l

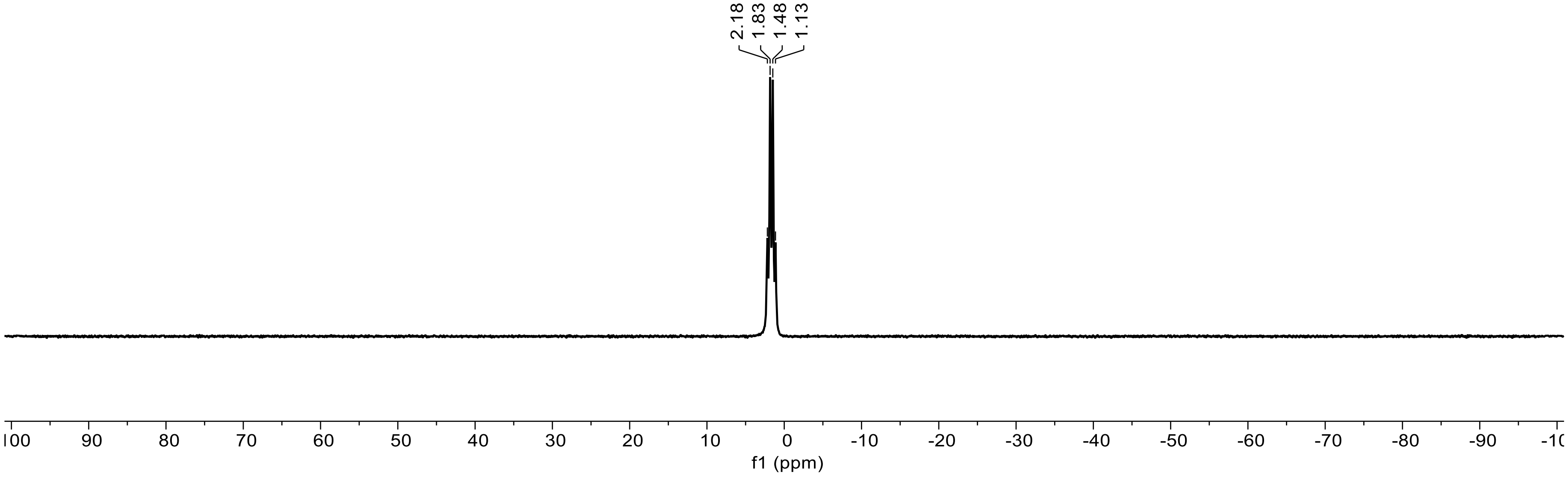

^{13}C NMR of 1l

CD_3CN, 101 MHz, 25 °C

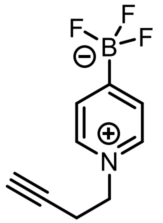

1l

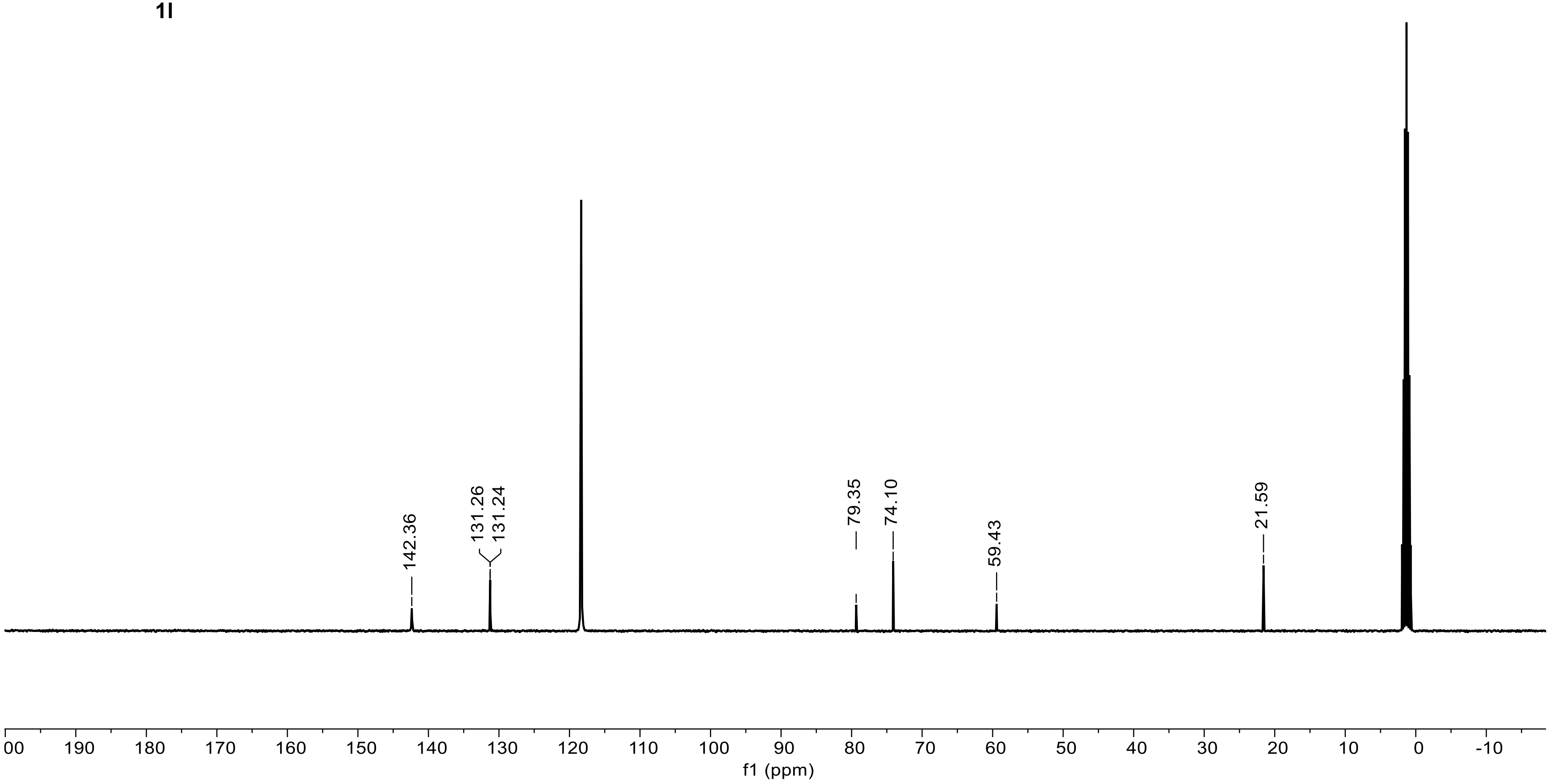

^{1}H NMR of 1m

DMSO-d_6, 400 MHz, 25 °C

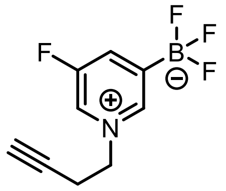

1m

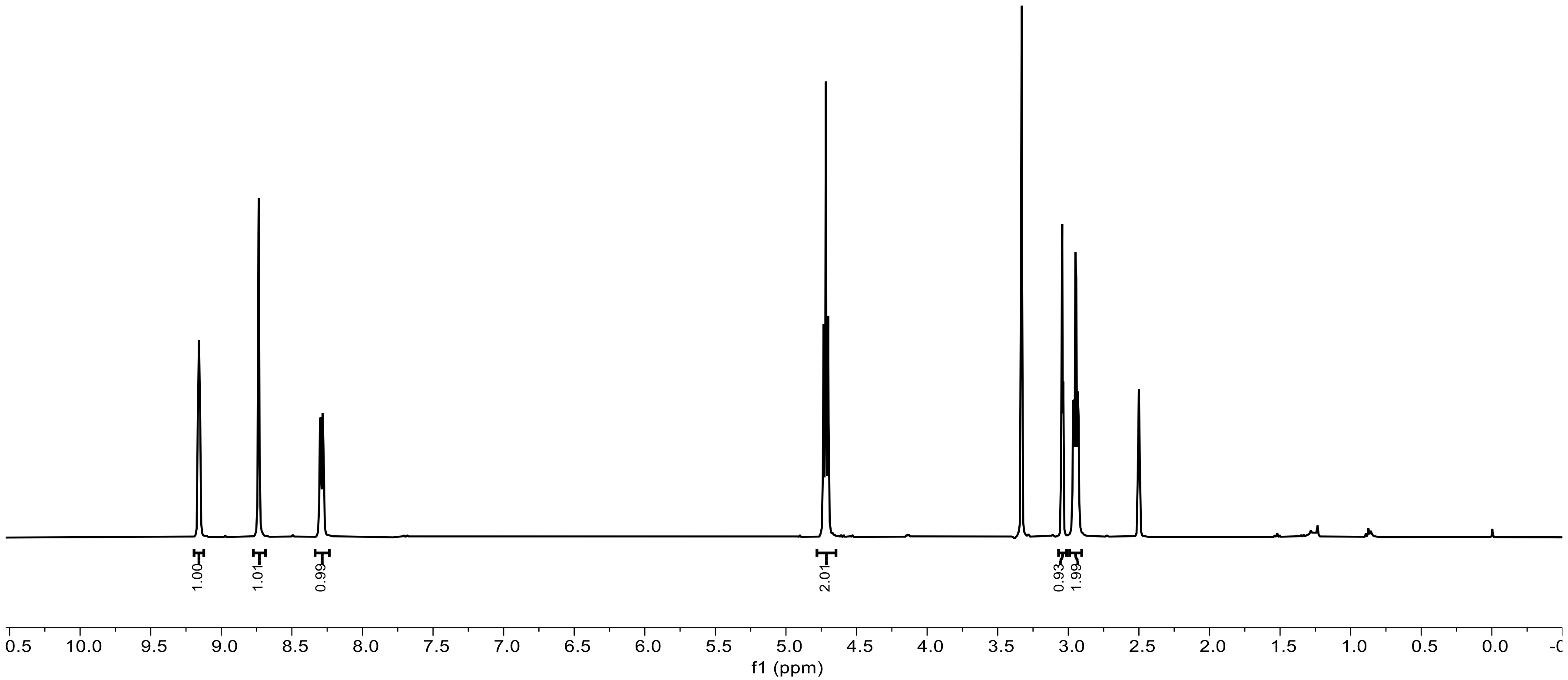

^{19}F NMR of 1m

CD_3CN, 376 MHz, 25 °C

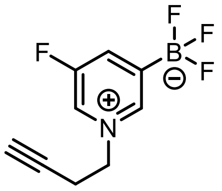

1m

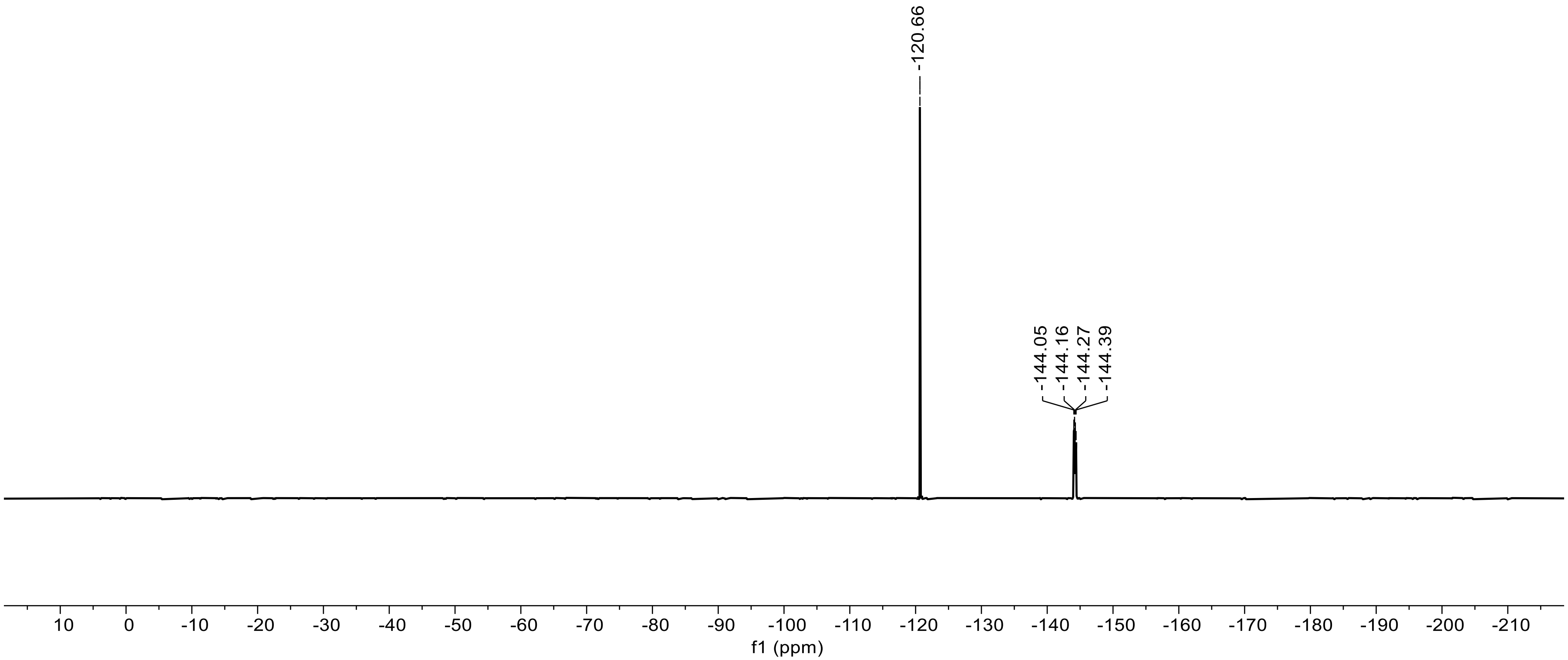

^{11}B NMR of 1m

CD_3CN, 128 MHz, 25 °C

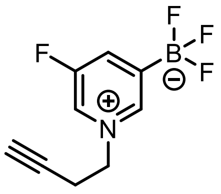

1m

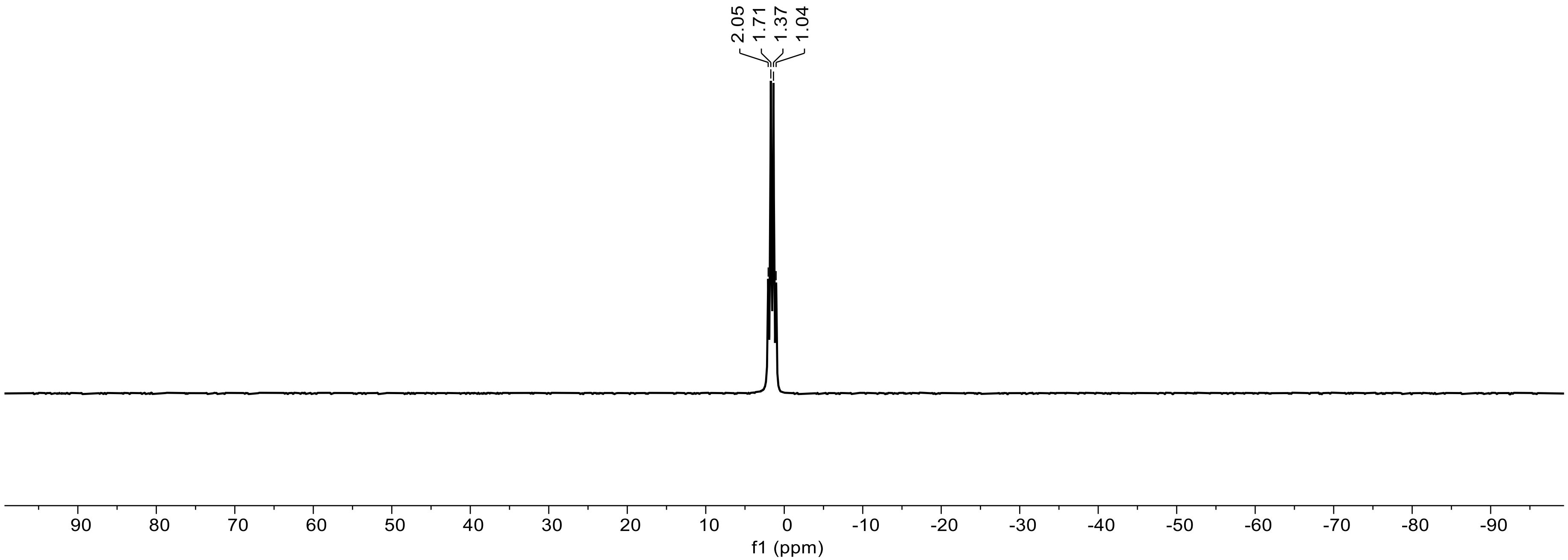

^{13}C NMR of 1m

DMSO-d_6, 101 MHz, 25 °C

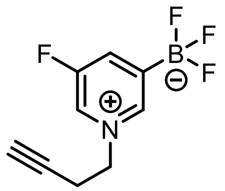

1m

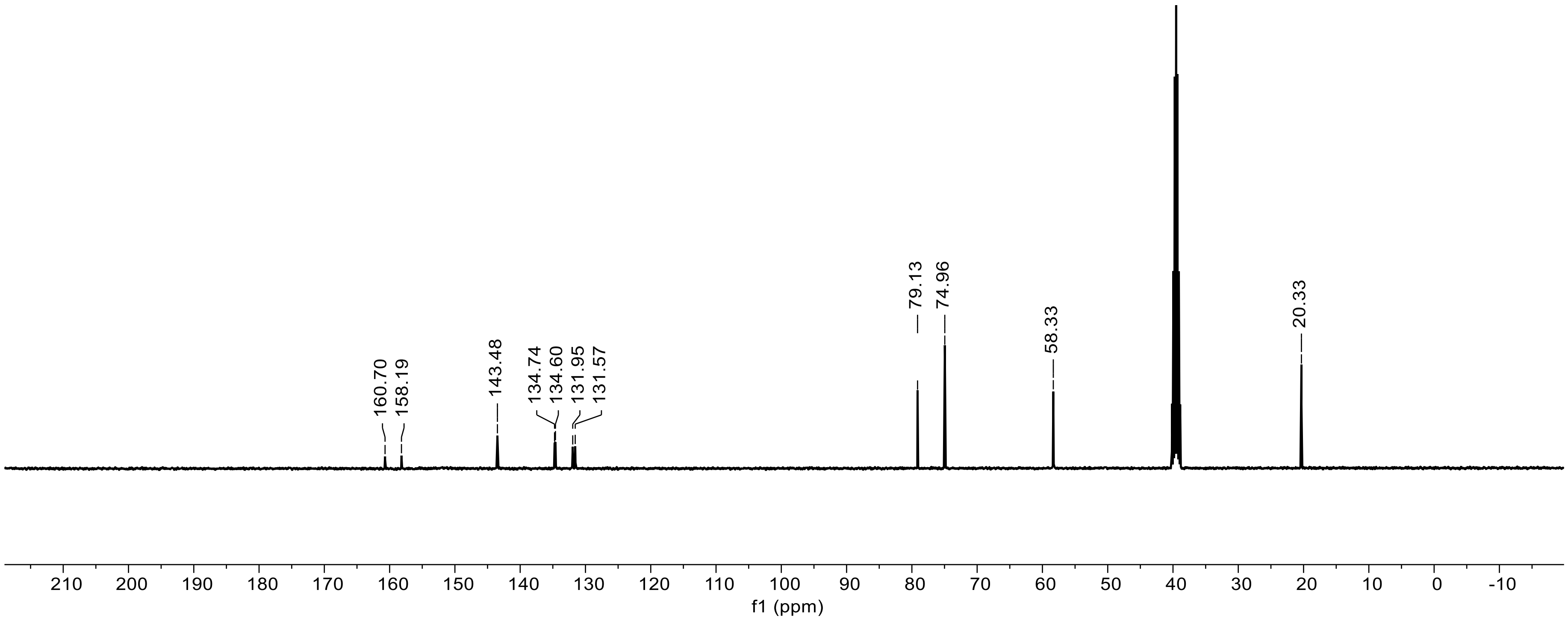

^{1}H NMR of 1n

CD_3CN, 400 MHz, 25 °C

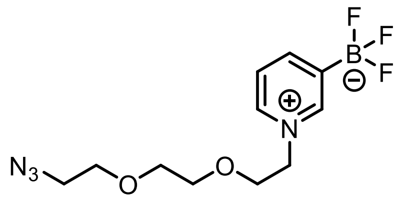

1n

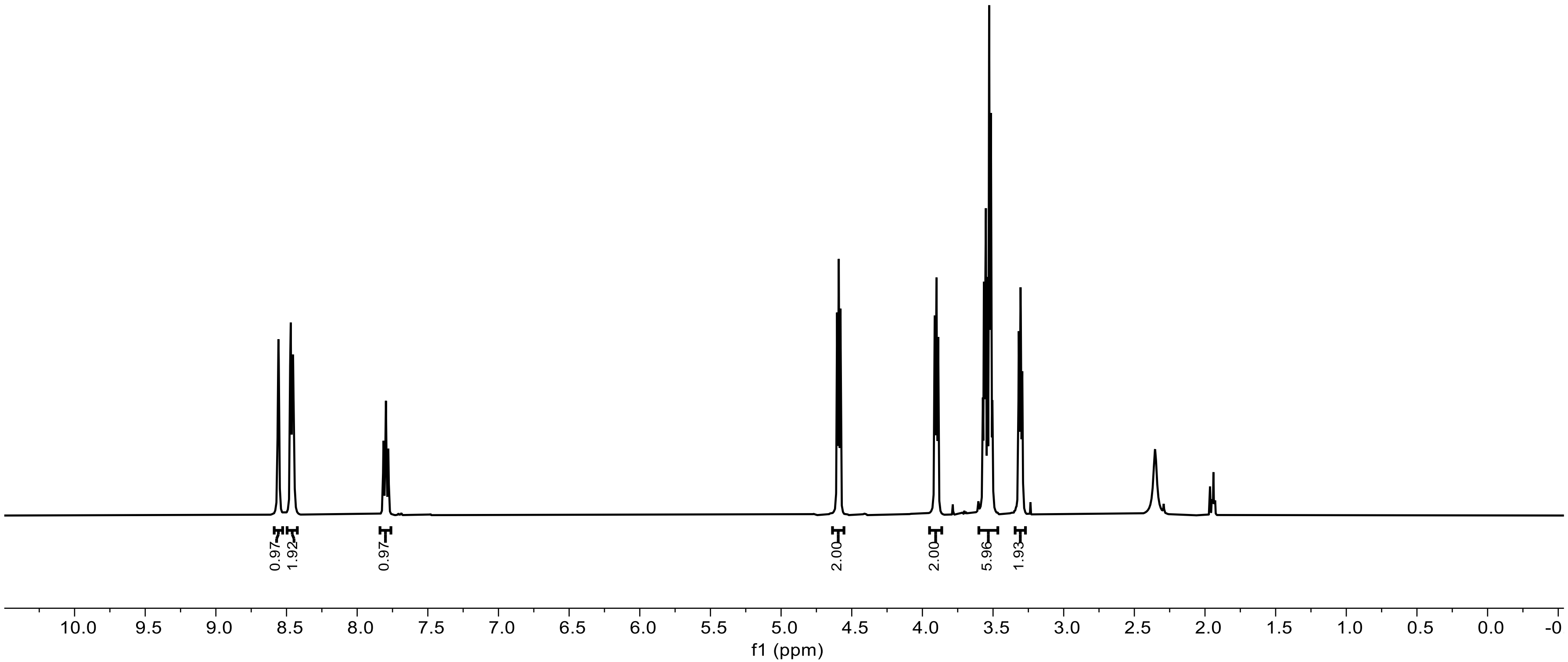

^{19}F NMR of 1n

CD_3CN, 376 MHz, 25 °C

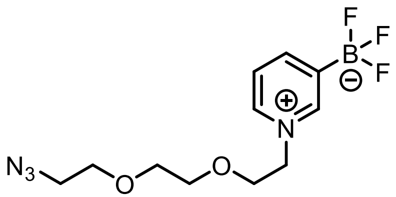

1n

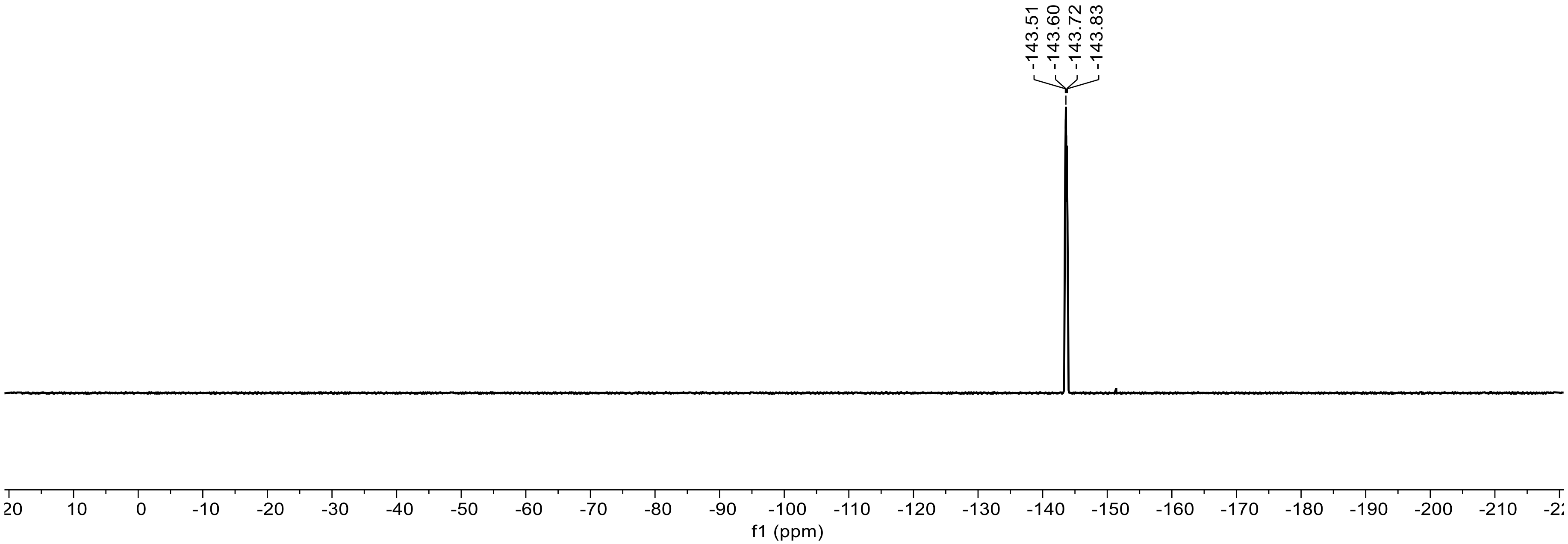

^{11}B NMR of 1n

CD_3CN, 128 MHz, 25 °C

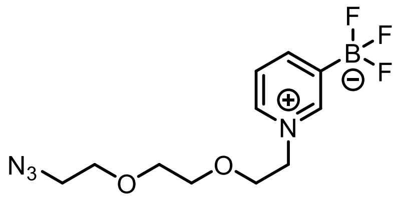

1n

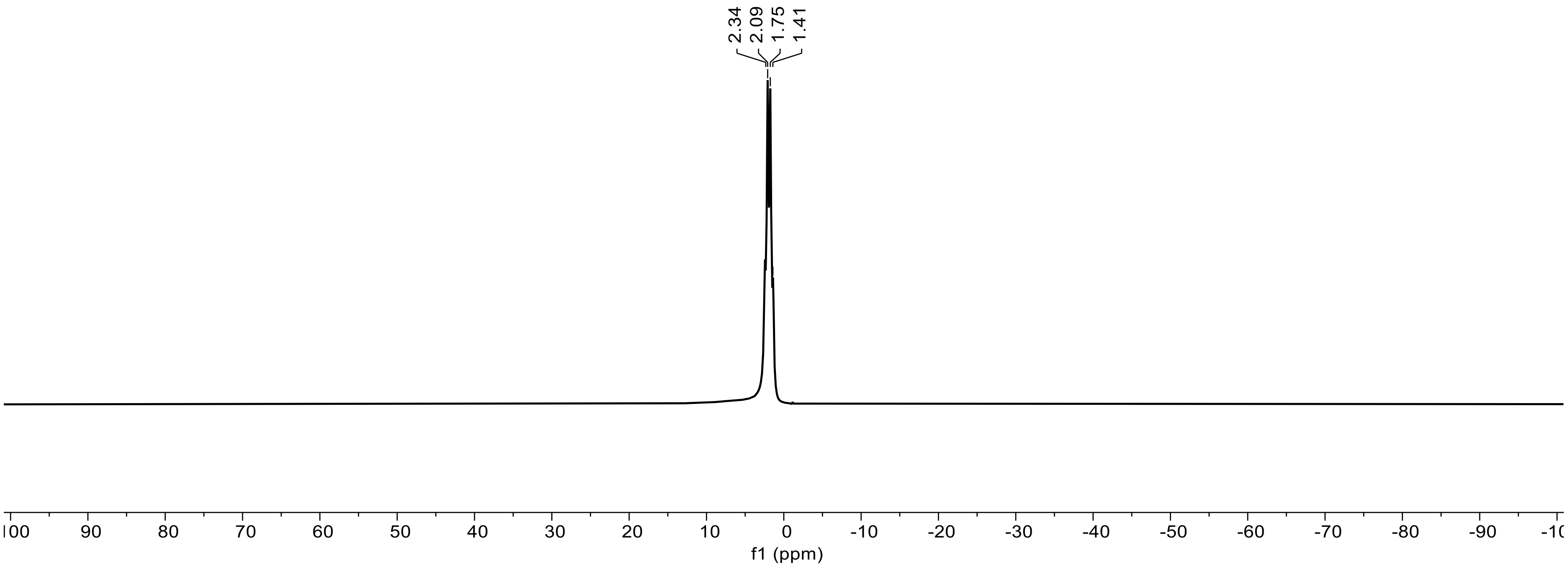

^{13}C NMR of 1n

CD_3CN, 101 MHz, 25 °C

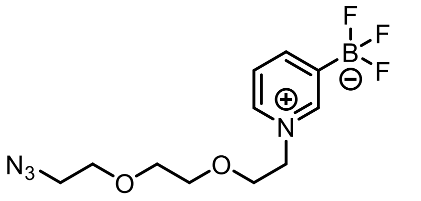

1n

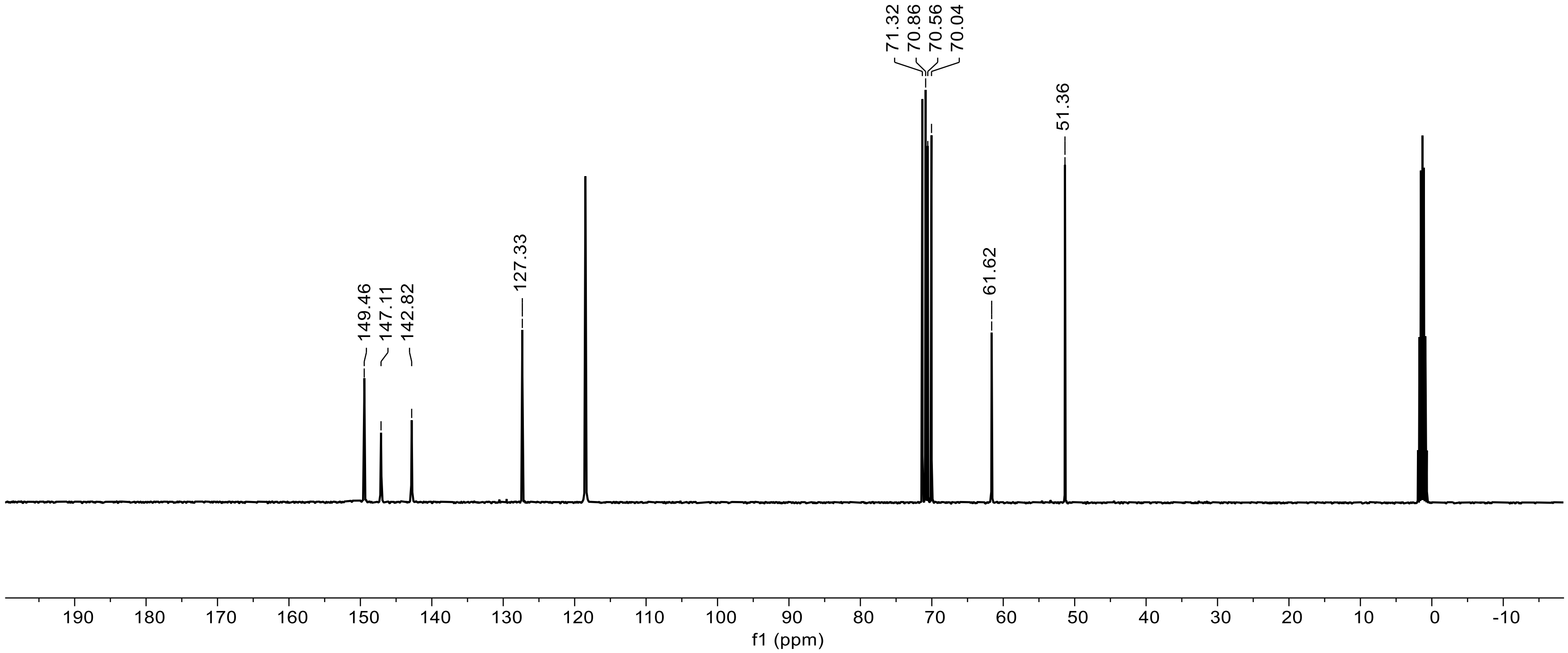

^{1}H NMR of 1o

CD_3CN, 400 MHz, 25 °C

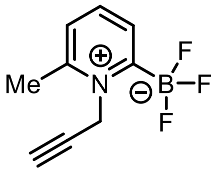

1o

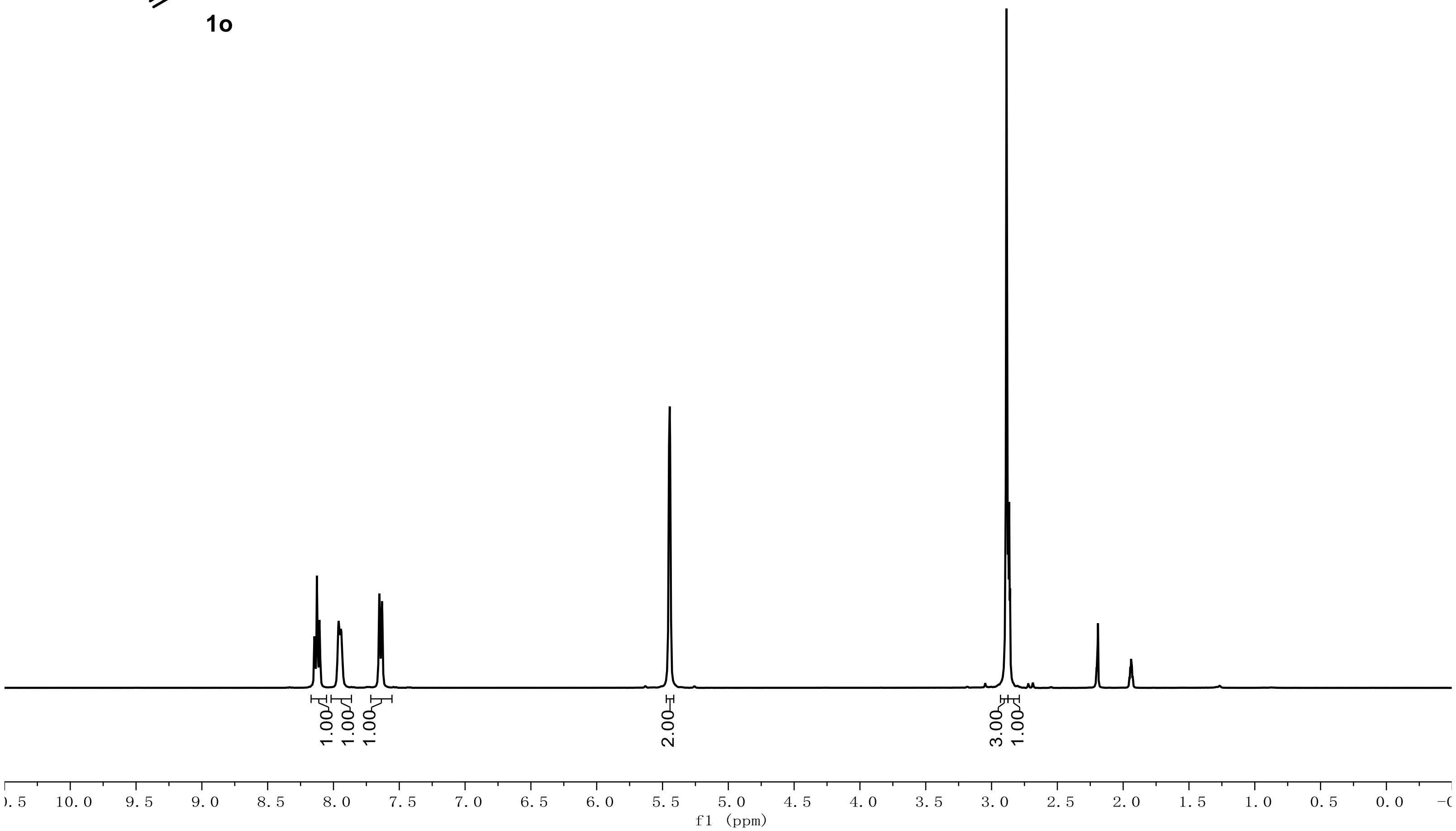

^{19}F NMR of 1o

CD_3CN, 376 MHz, 25 °C

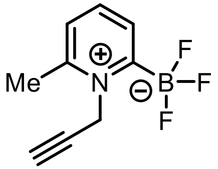

1o

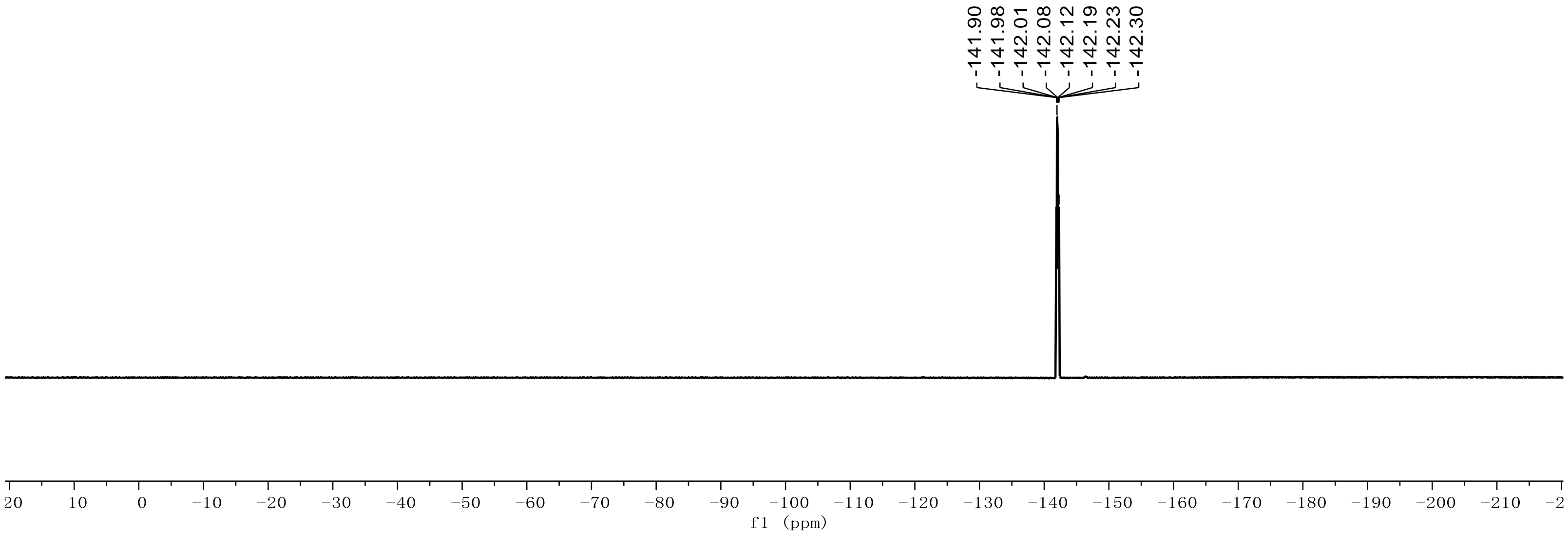

^{11}B NMR of 1o

CD_3CN, 128 MHz, 25 °C

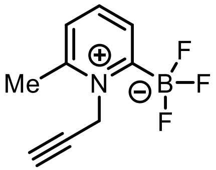

1o

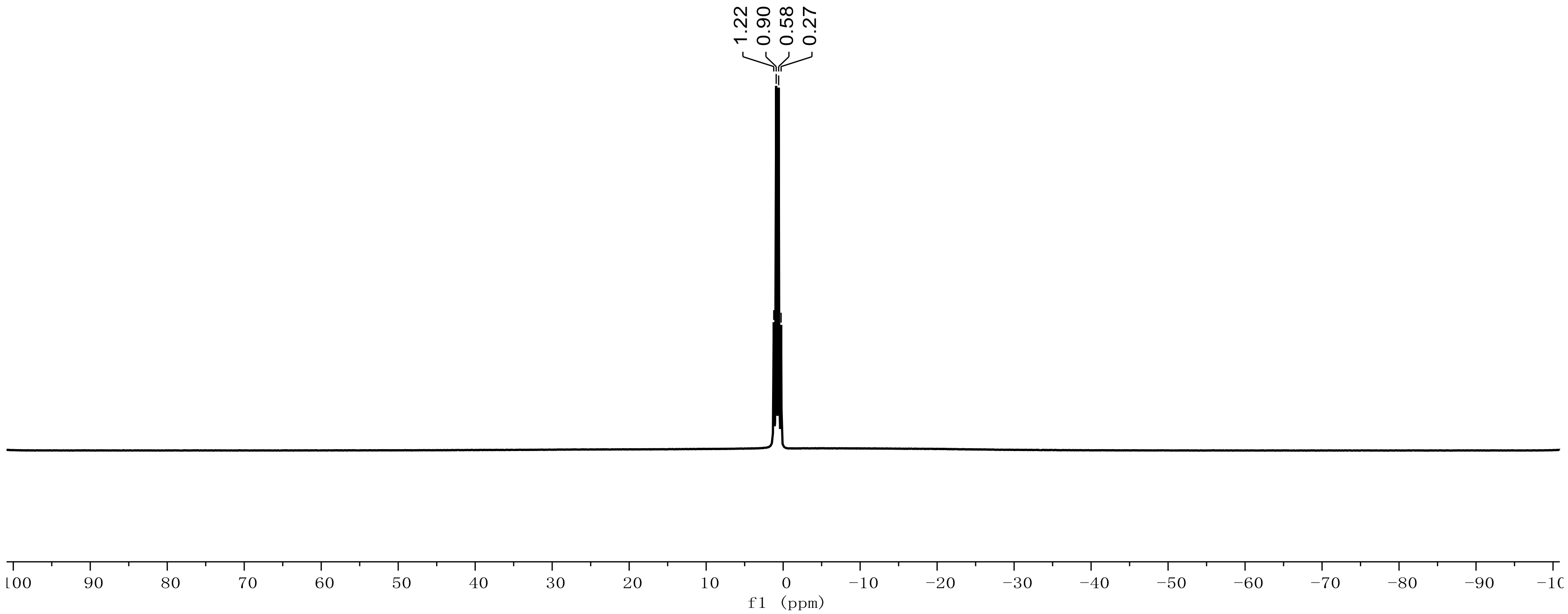

^{13}C NMR of 1o

CD_3CN, 101 MHz, 25 °C

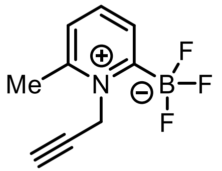

1o

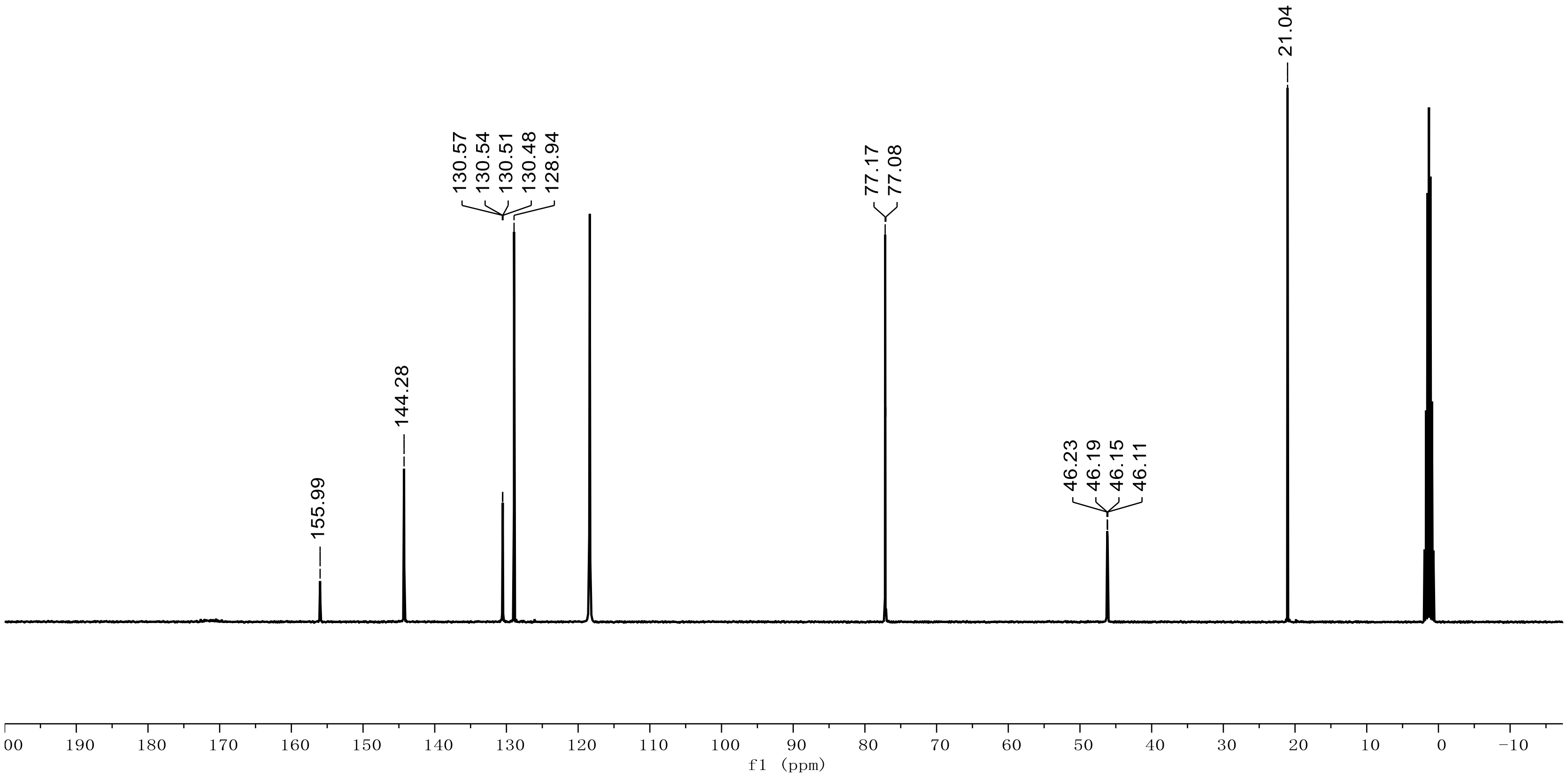

^{1}H NMR of 1p

CD_3CN, 400 MHz, 25 °C

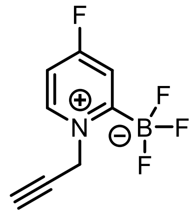

1p

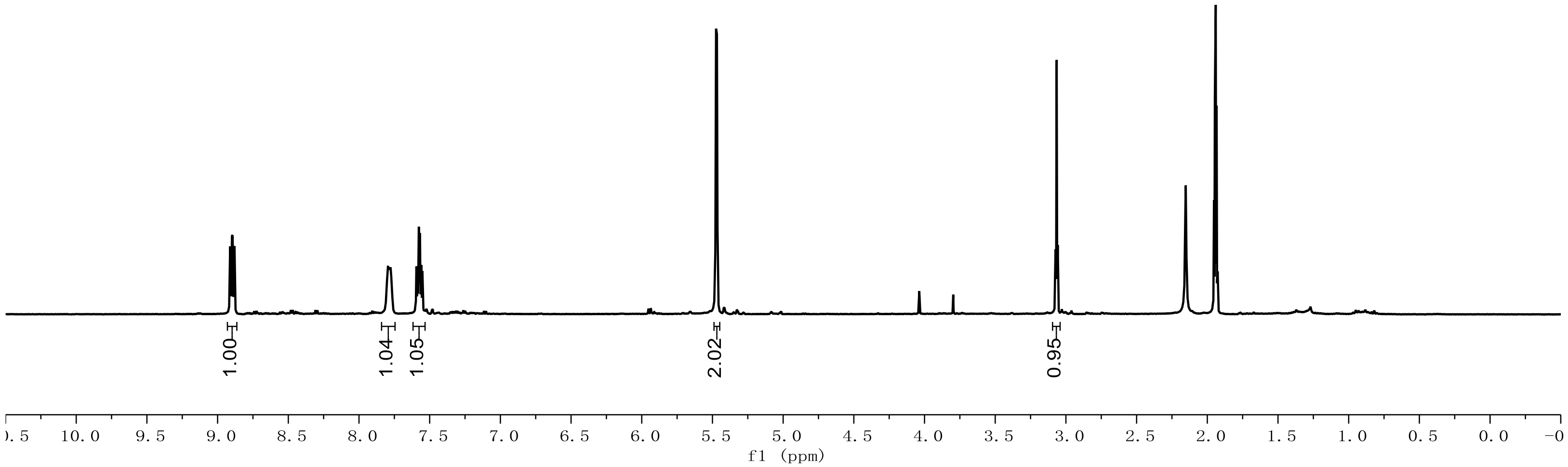

^{19}F NMR of 1p

CD_3CN, 376 MHz, 25 °C

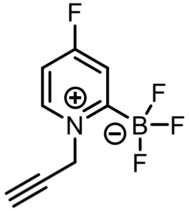

1p

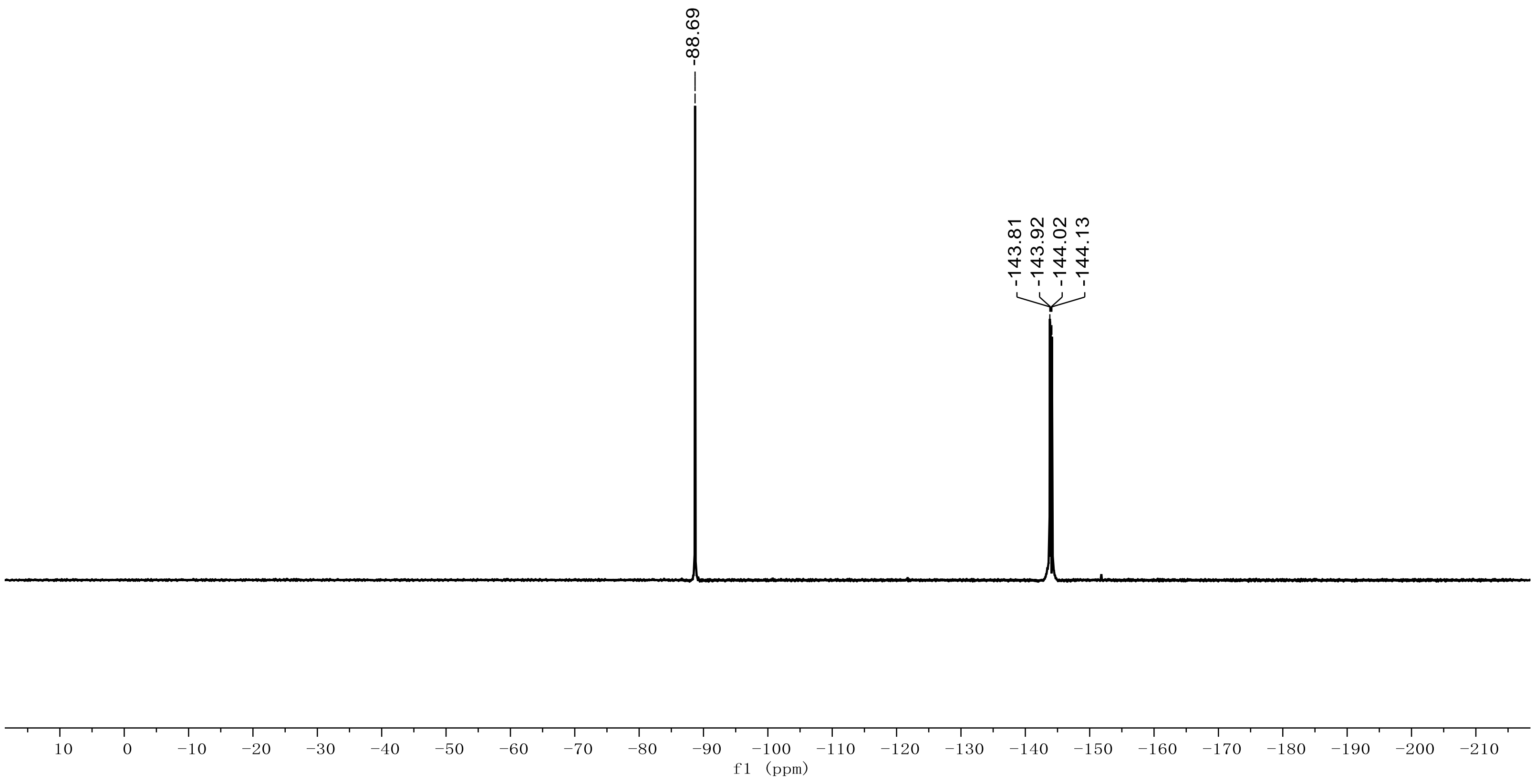

^{11}B NMR of 1p

CD_3CN, 128 MHz, 25 °C

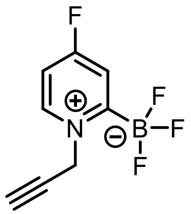

1p

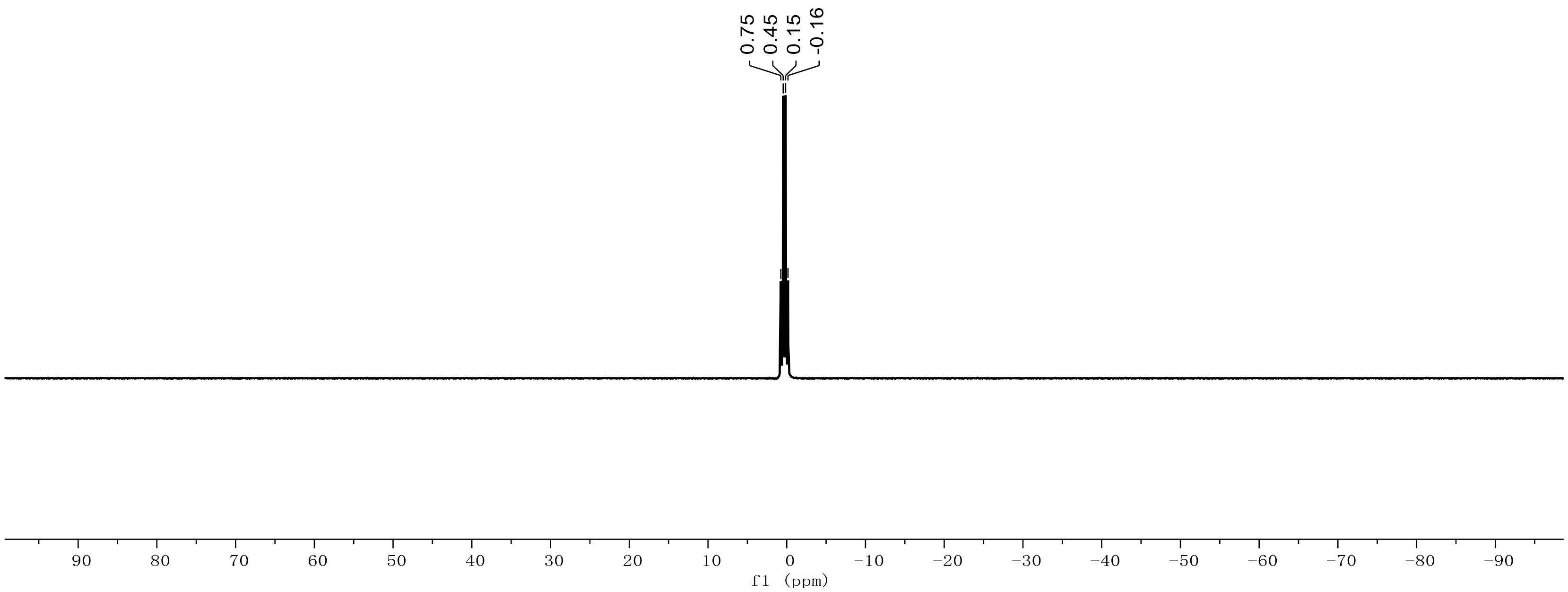

^{13}C NMR of 1p

CD_3CN, 101 MHz, 25 °C

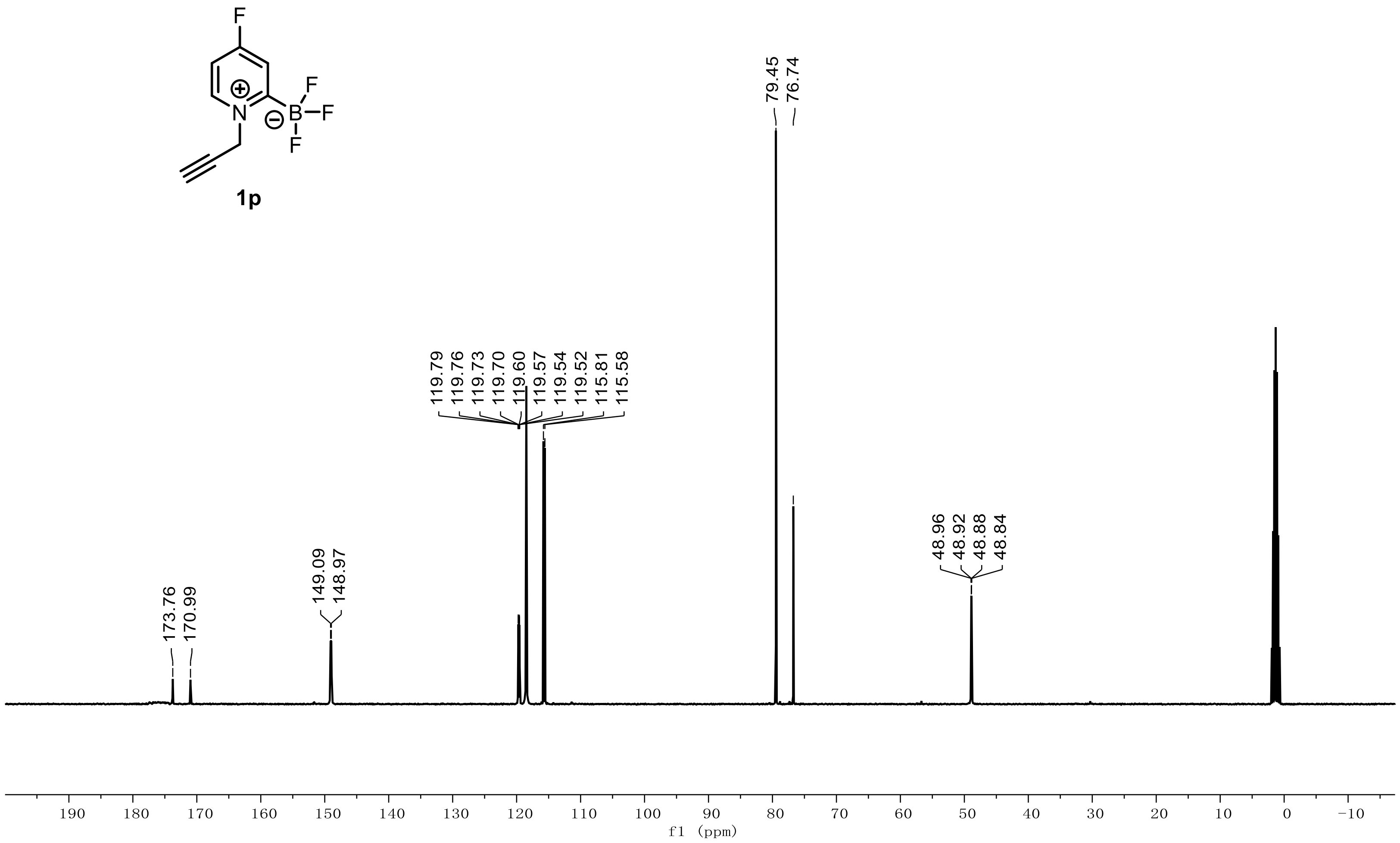

^{1}H NMR of 1q

CD_3CN, 400 MHz, 25 °C

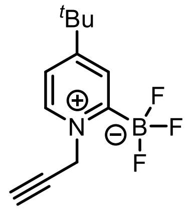

1q

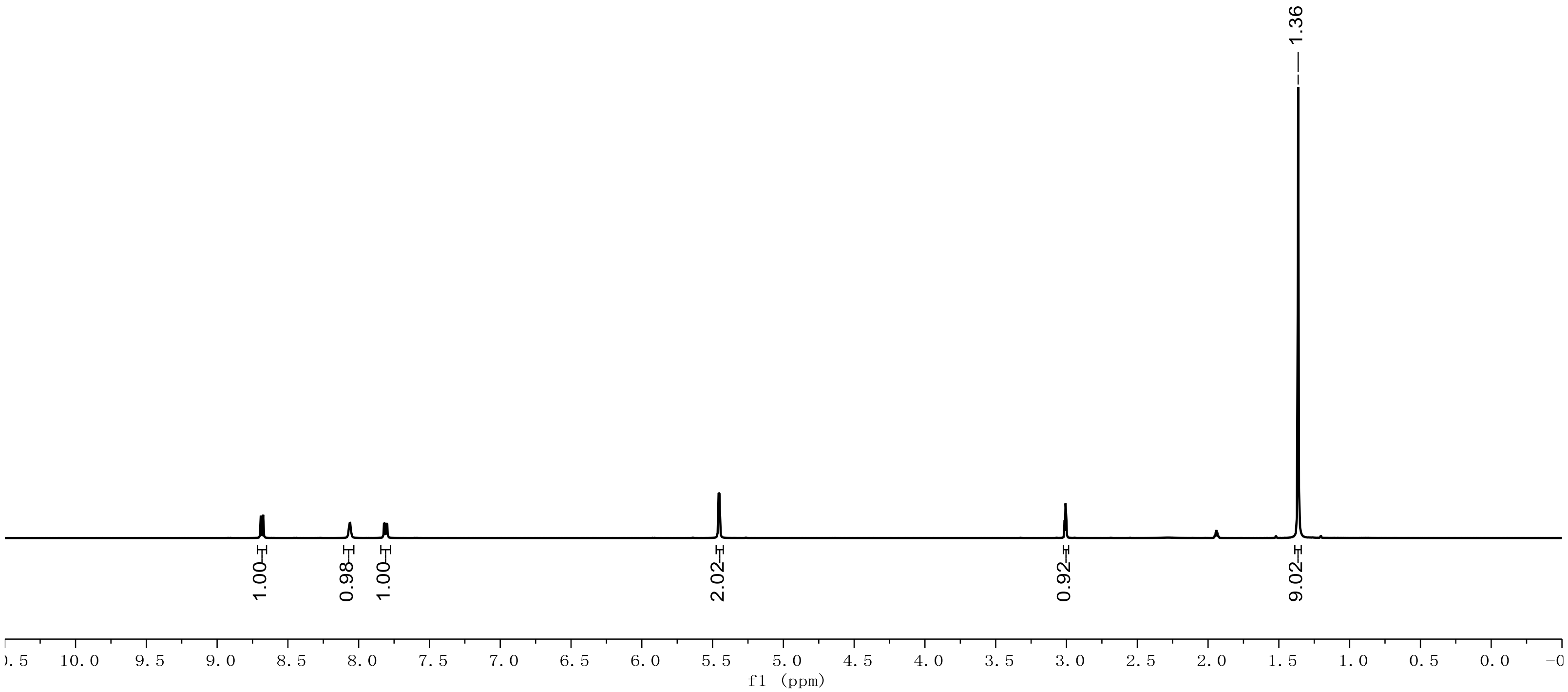

^{19}F NMR of 1q

CD_3CN, 376 MHz, 25 °C

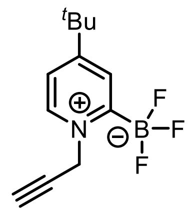

1q

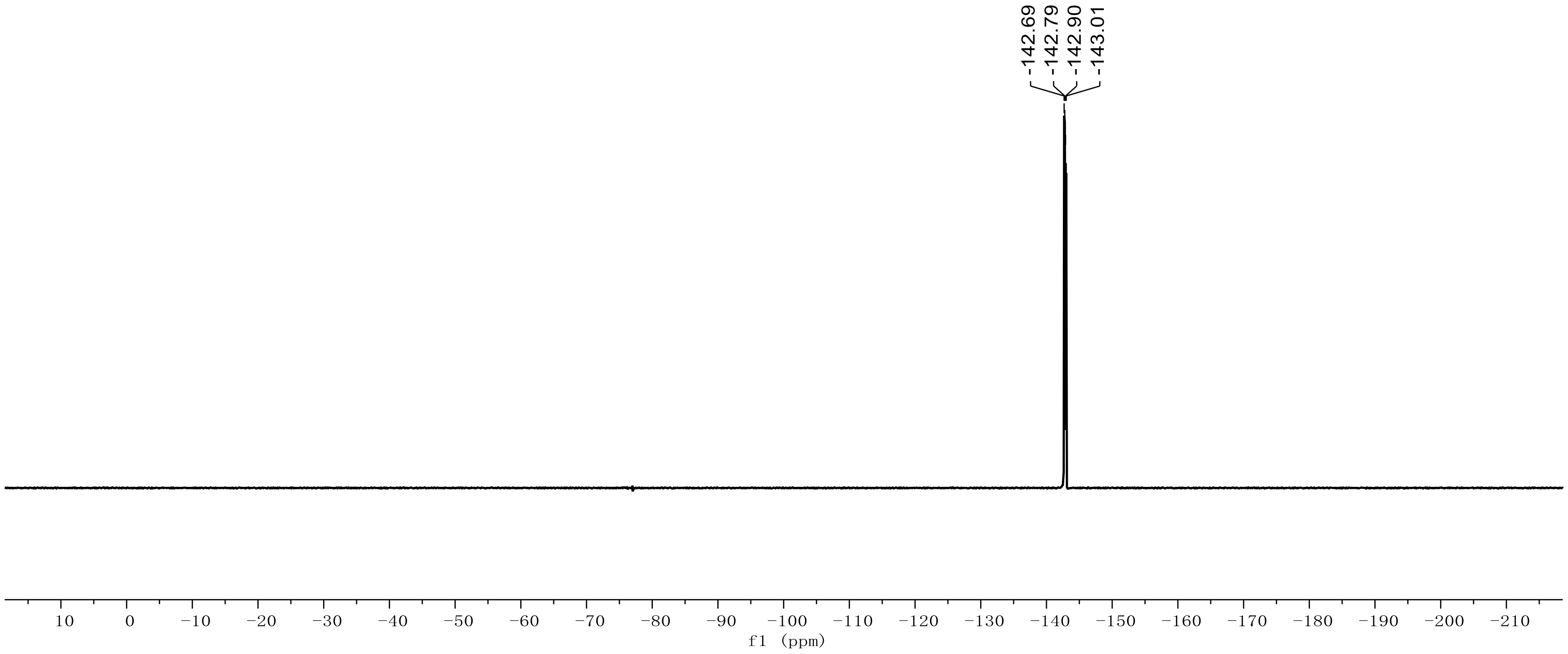

^{11}B NMR of 1q

CD_3CN, 128 MHz, 25 °C

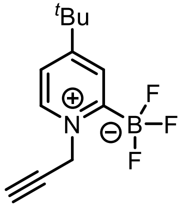

1q

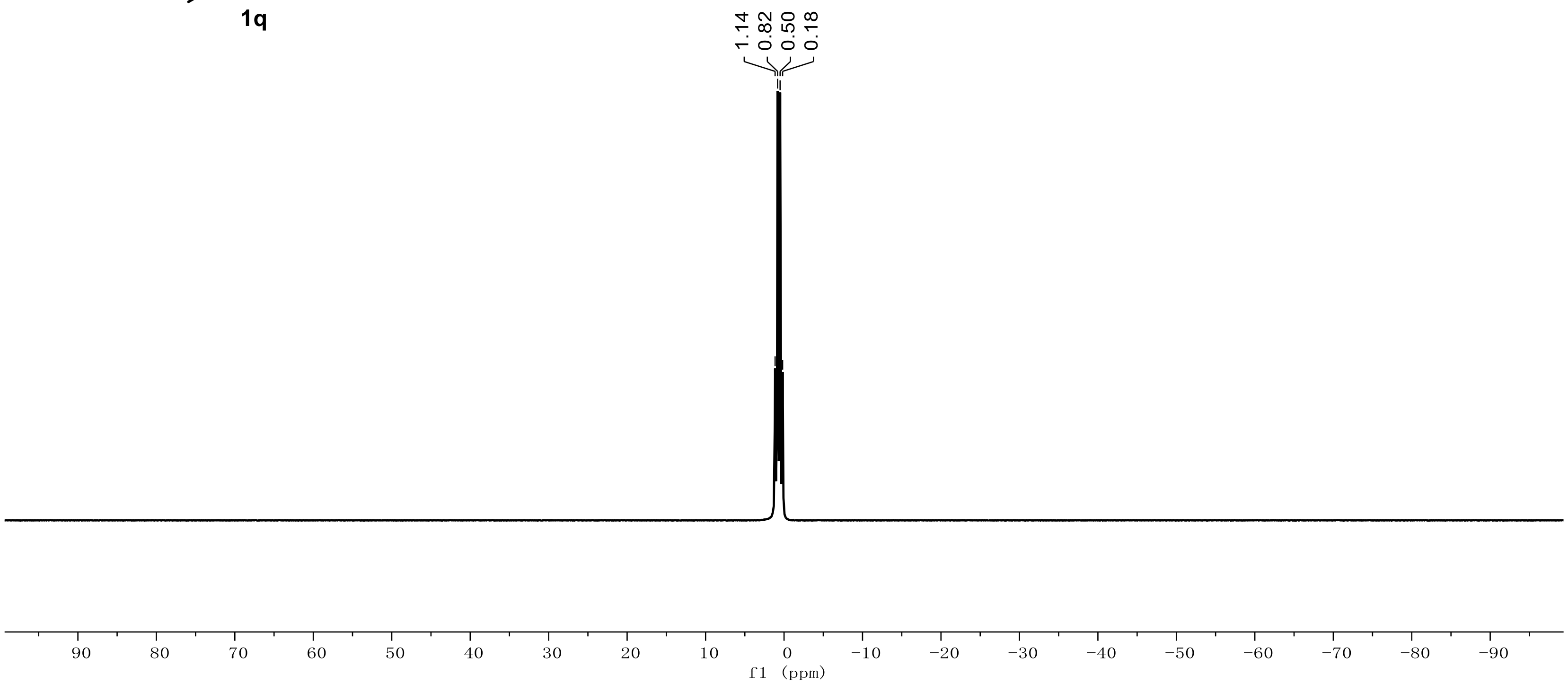

^{13}C NMR of 1q

CD_3CN, 101 MHz, 25 °C

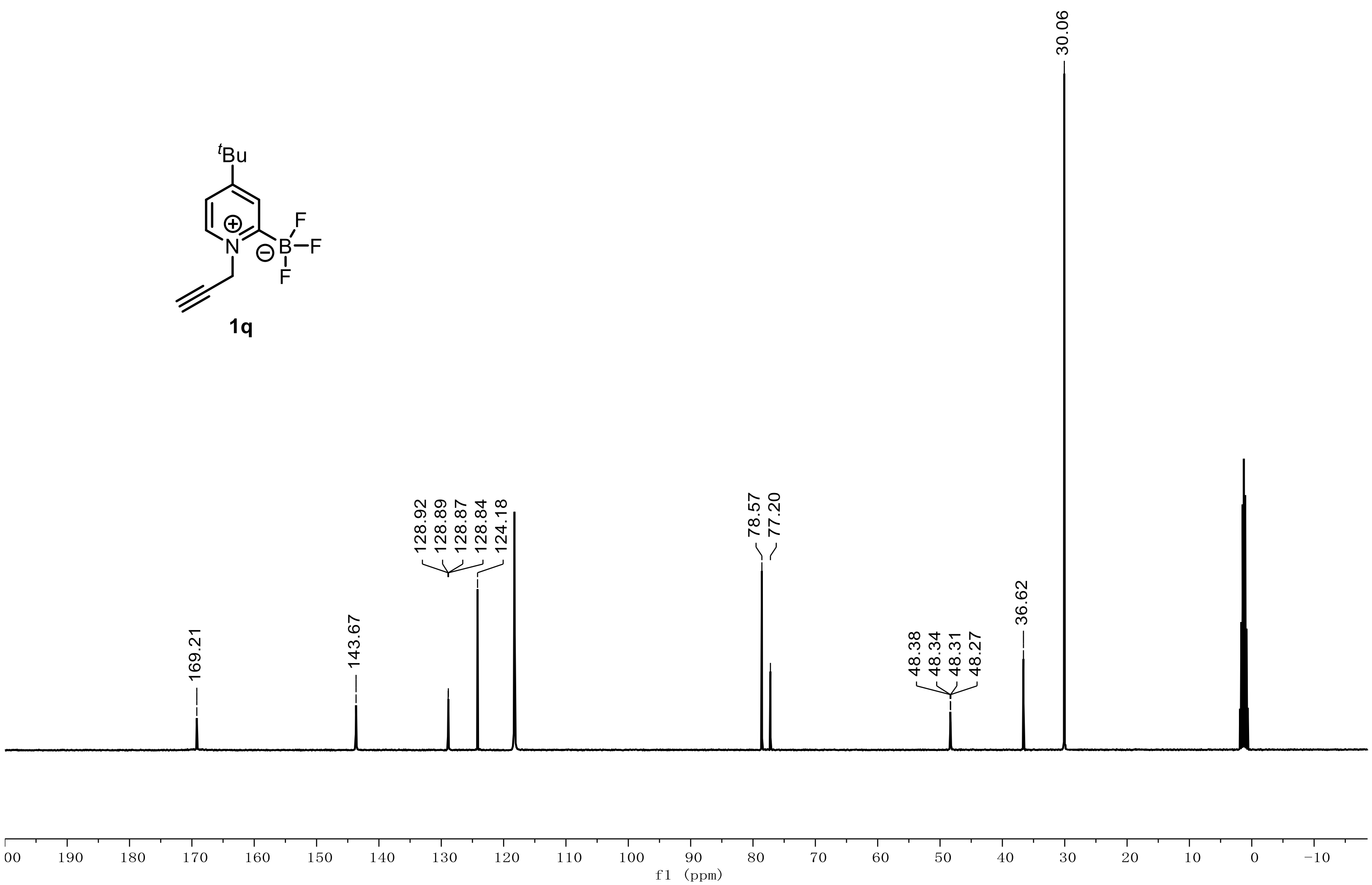

^{1}H NMR of 1r

CD_3CN, 400 MHz, 25 °C

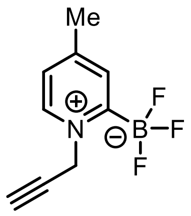

1r

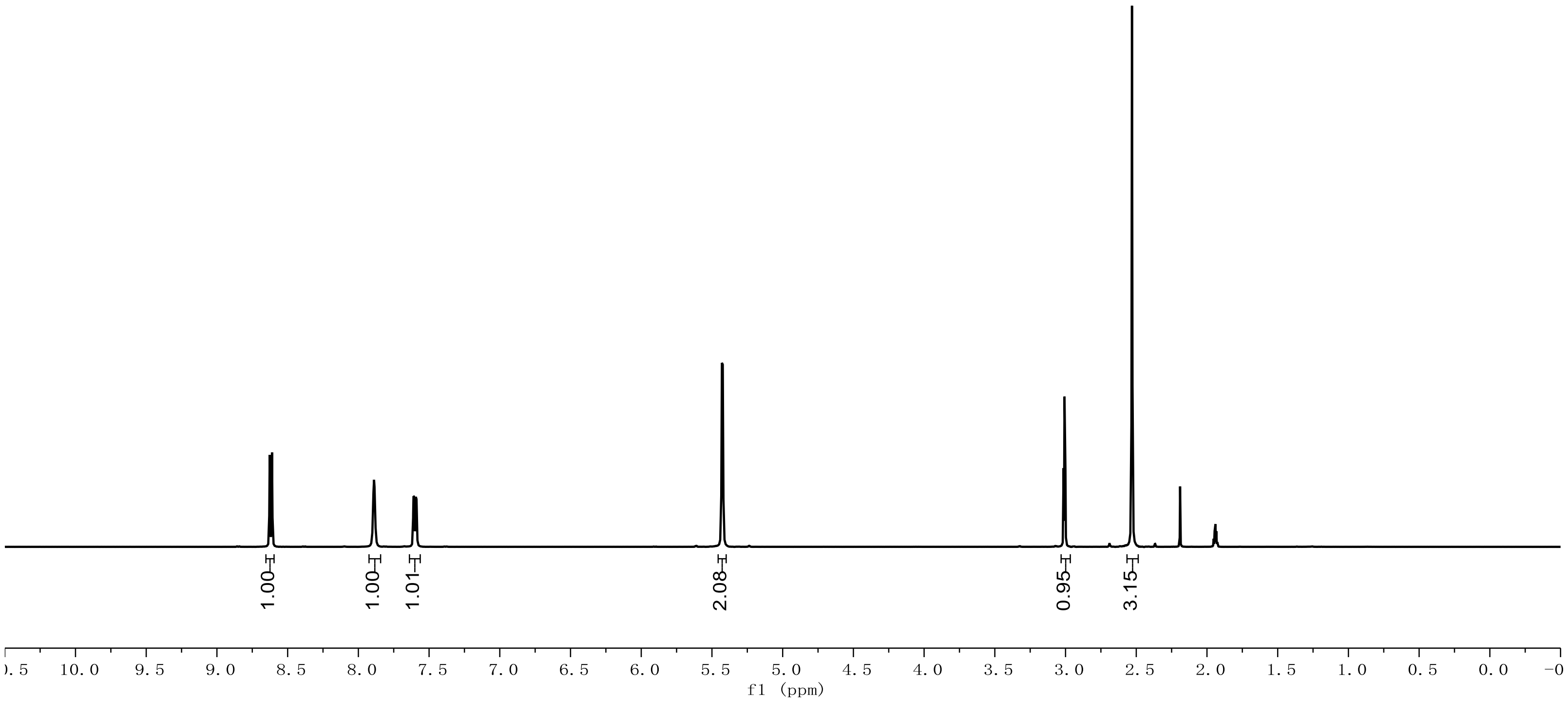

^{19}F NMR of 1r

CD_3CN, 376 MHz, 25 °C

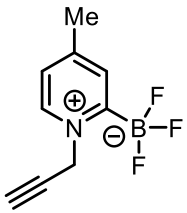

1r

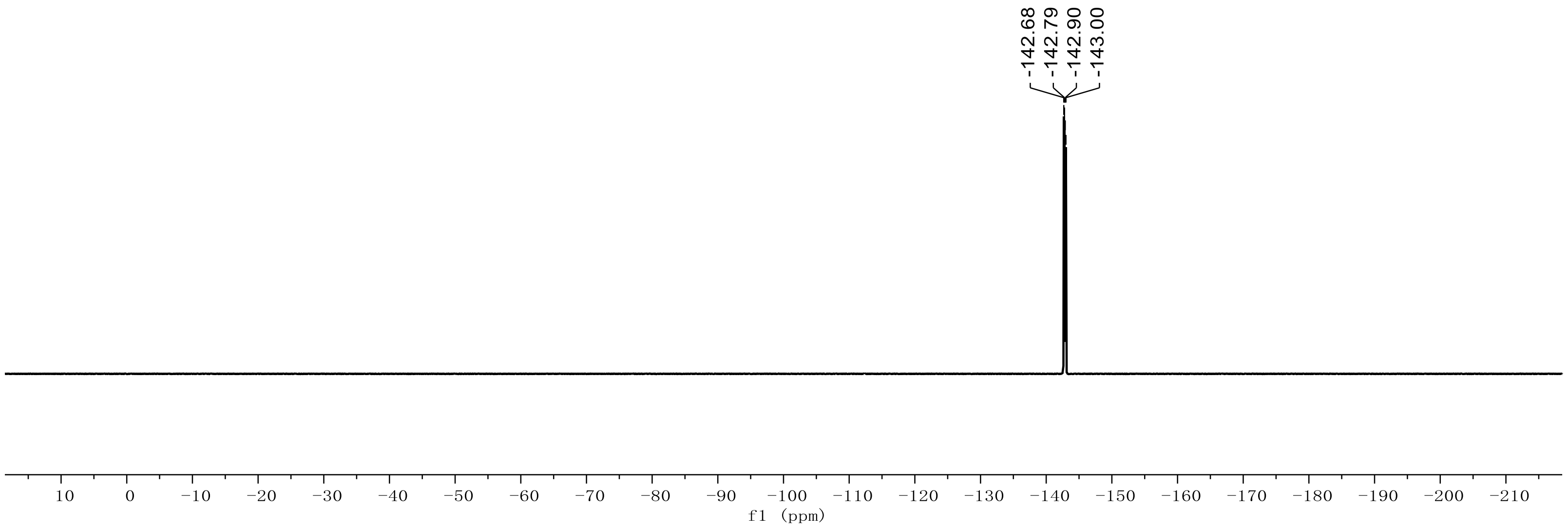

^{11}B NMR of 1r

CD_3CN, 128 MHz, 25 °C

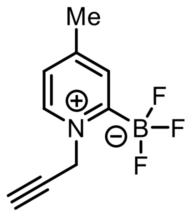

1r

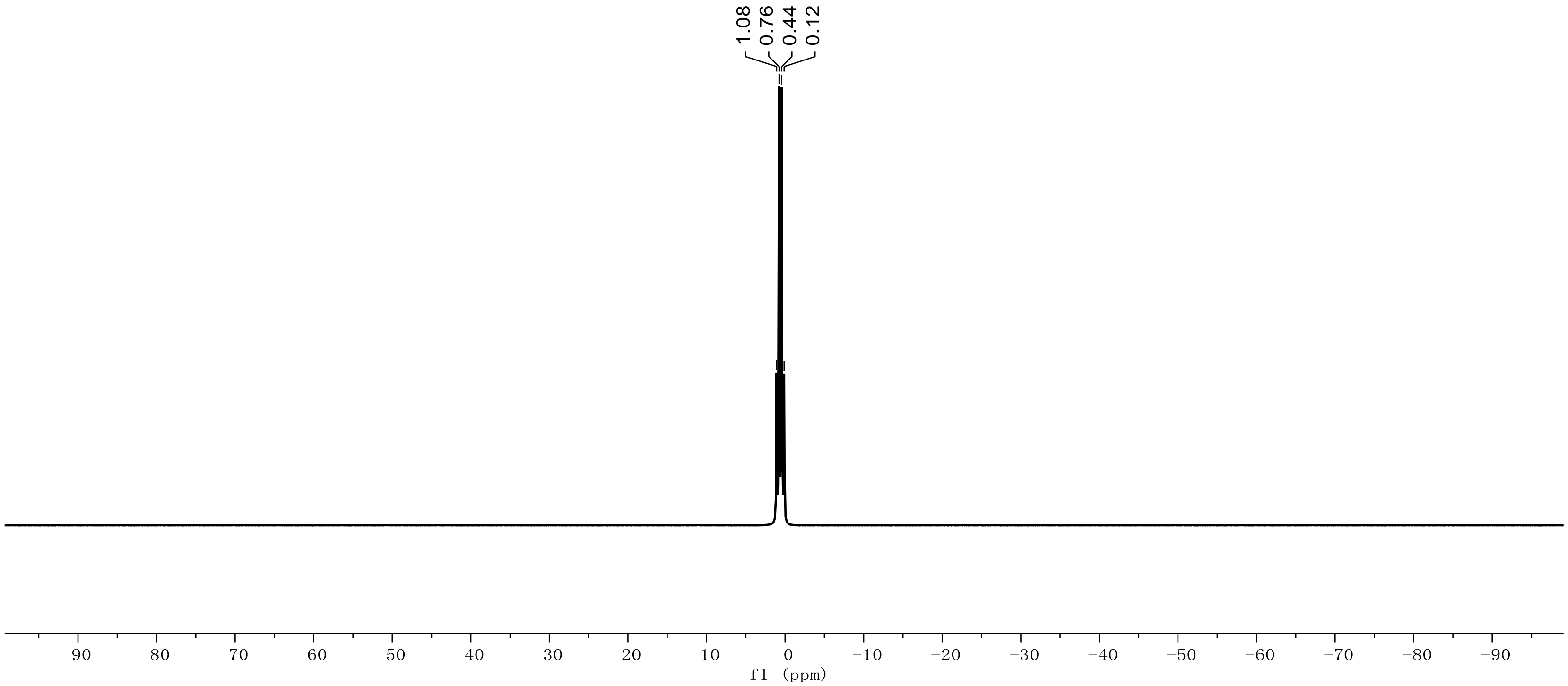

^{13}C NMR of 1r

CD_3CN, 101 MHz, 25 °C

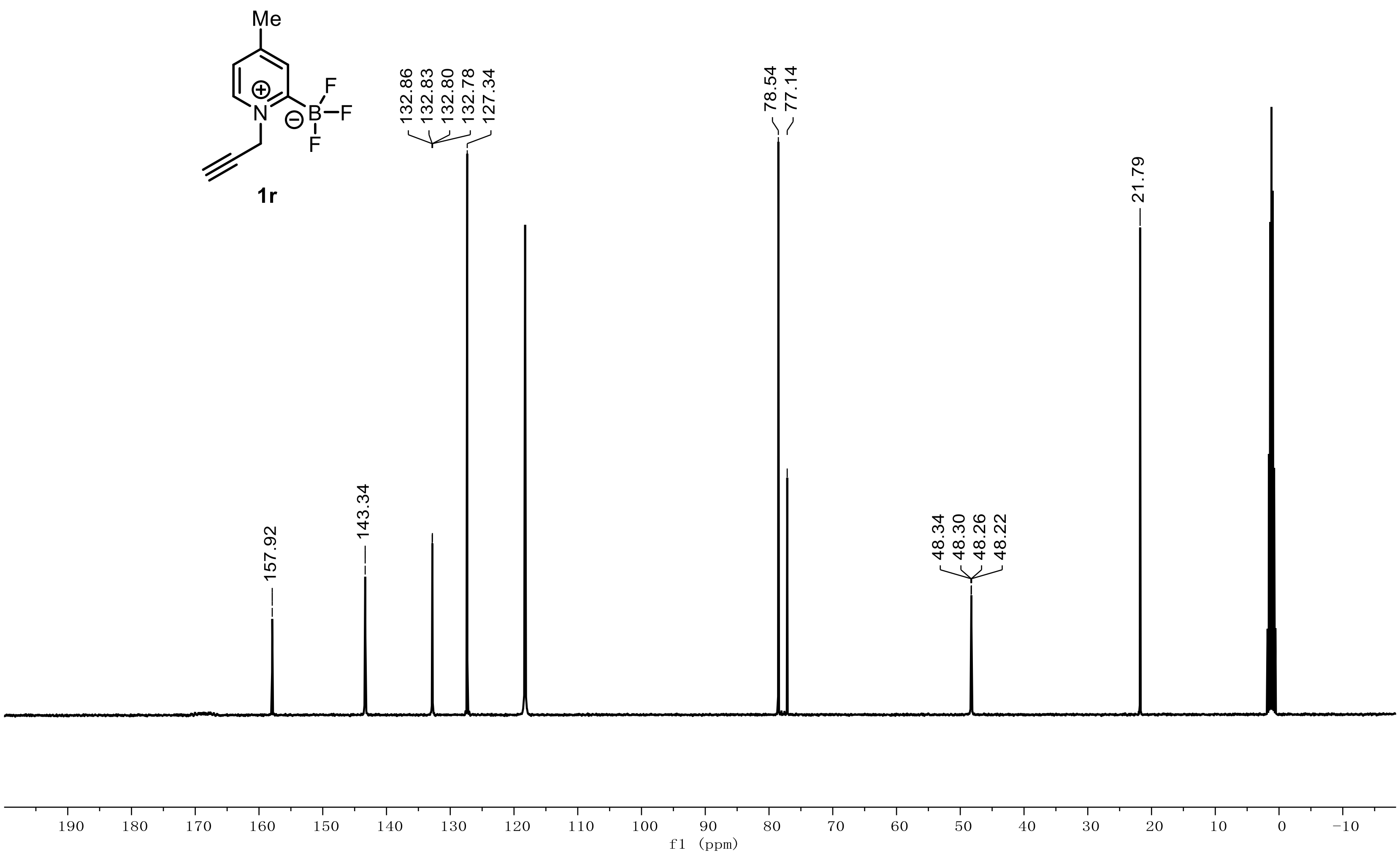

^{1}H NMR of 1s

CD_3CN, 400 MHz, 25 °C

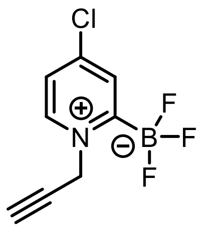

1s

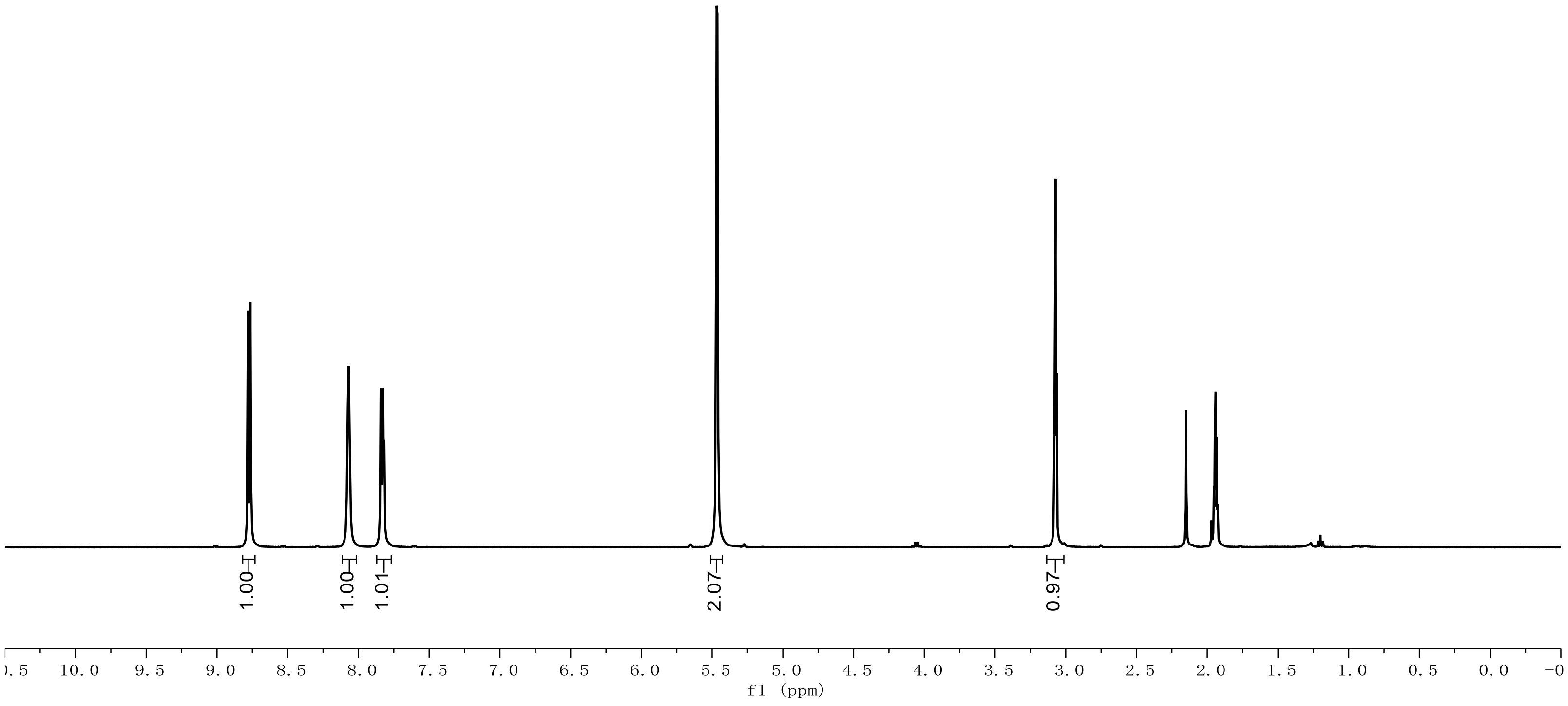

^{19}F NMR of 1s

CD_3CN, 376 MHz, 25 °C

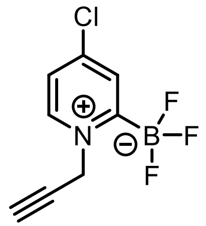

1s

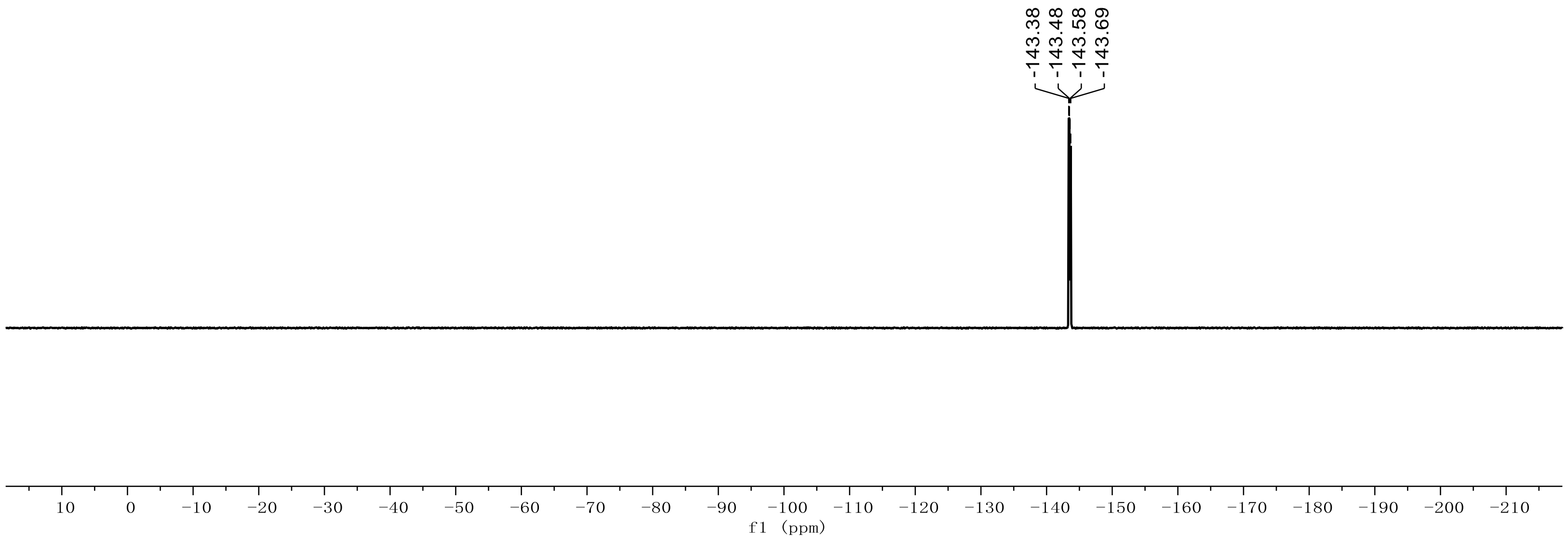

^{11}B NMR of 1s

CD_3CN, 128 MHz, 25 °C

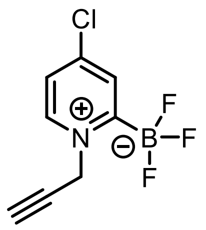

1s

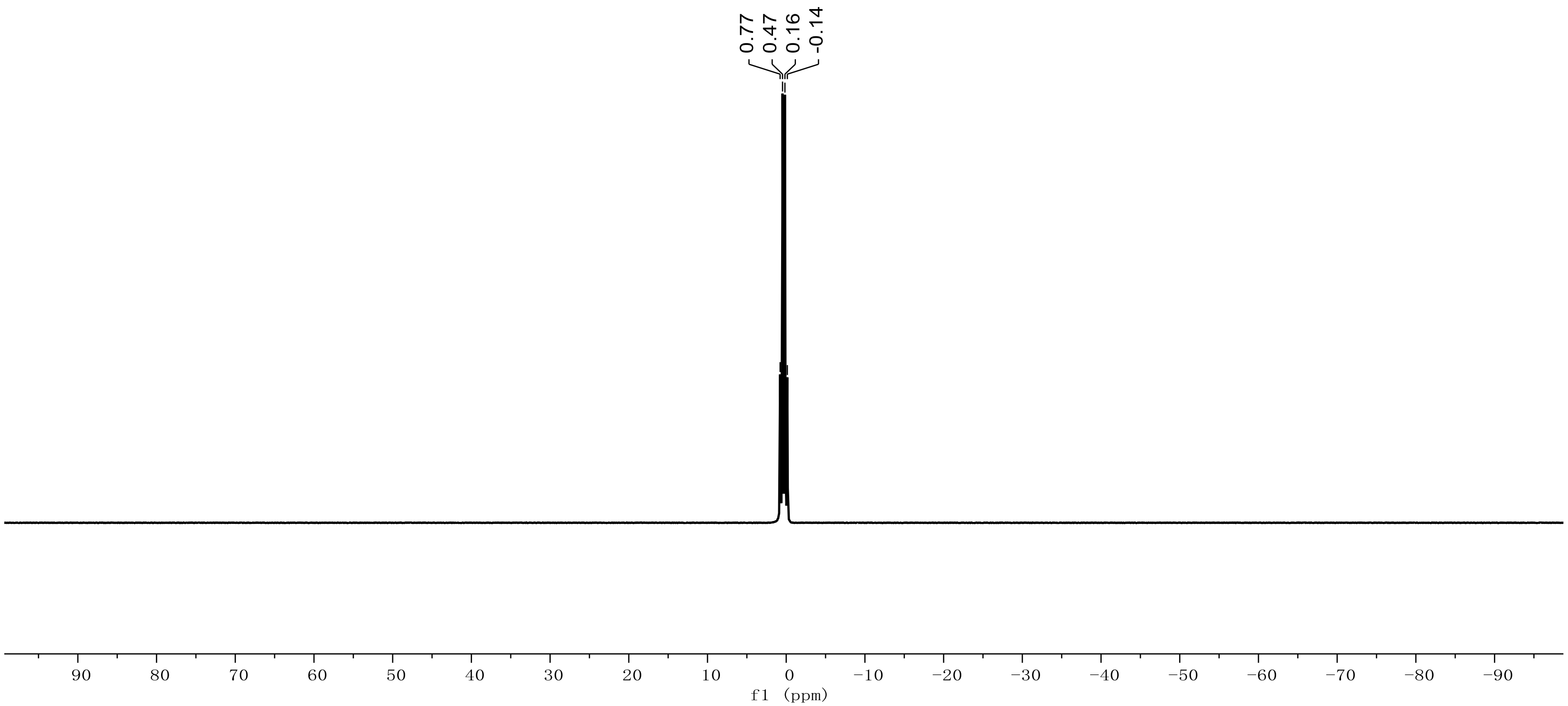

^{13}C NMR of 1s

CD_3CN, 101 MHz, 25 °C

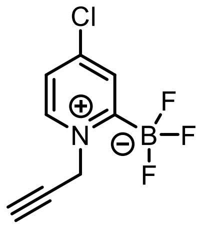

1s

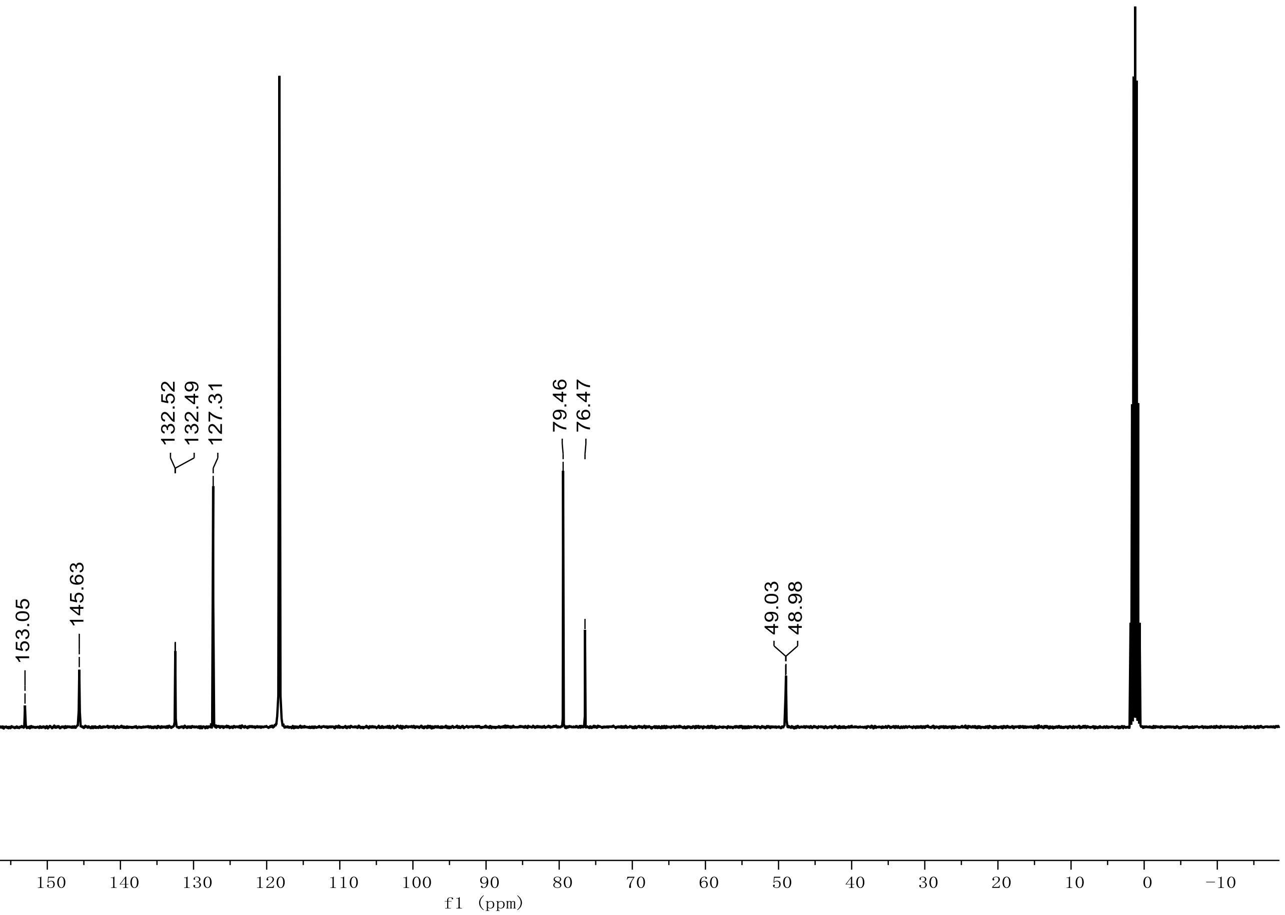

^{1}H NMR of 1t

CD_3CN, 400 MHz, 25 °C

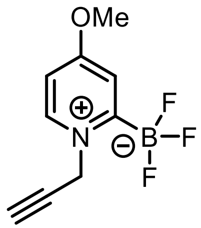

1t

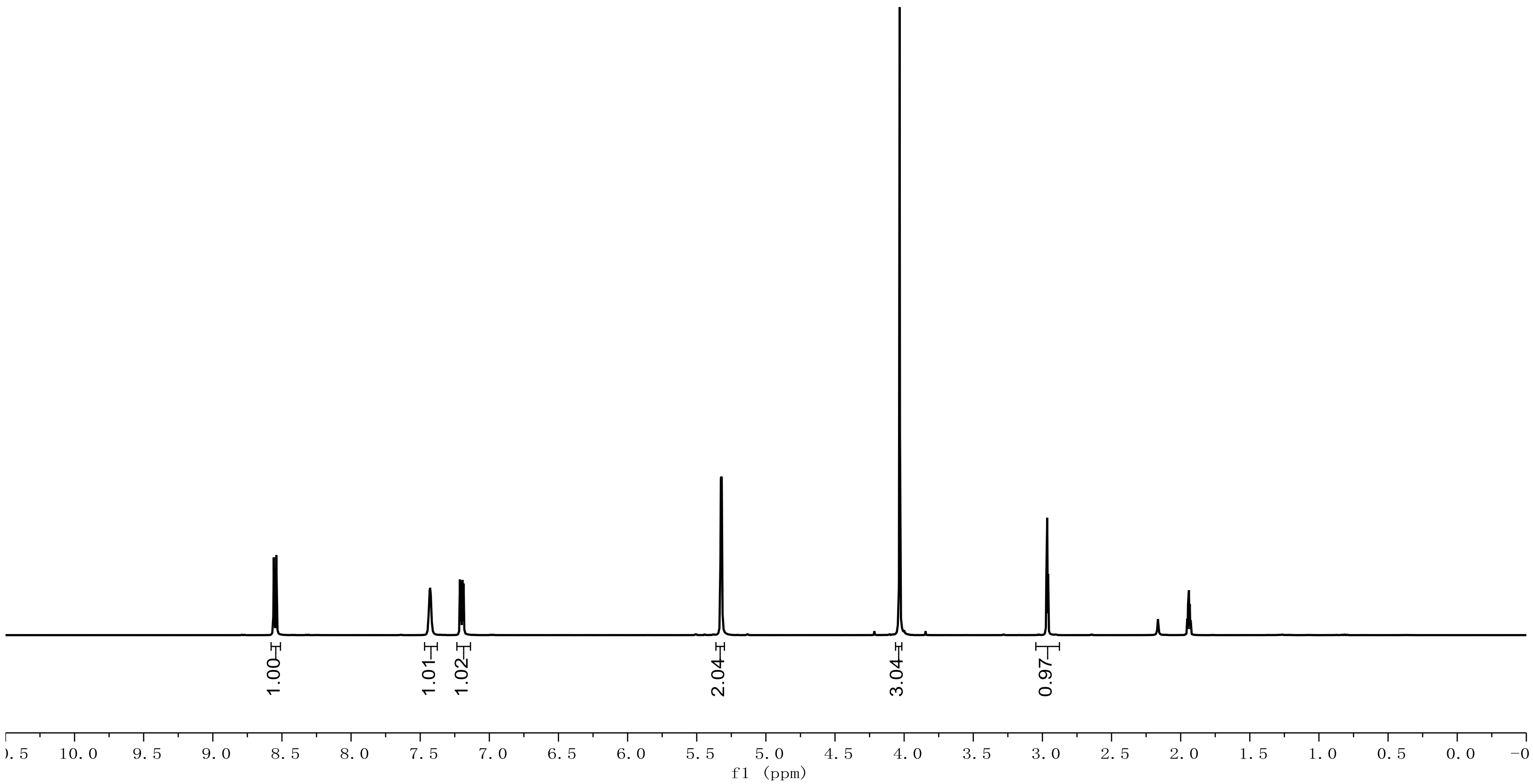

^{19}F NMR of 1t

CD_3CN, 376 MHz, 25 °C

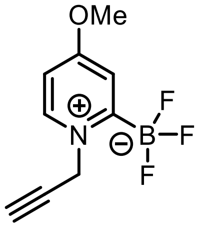

1t

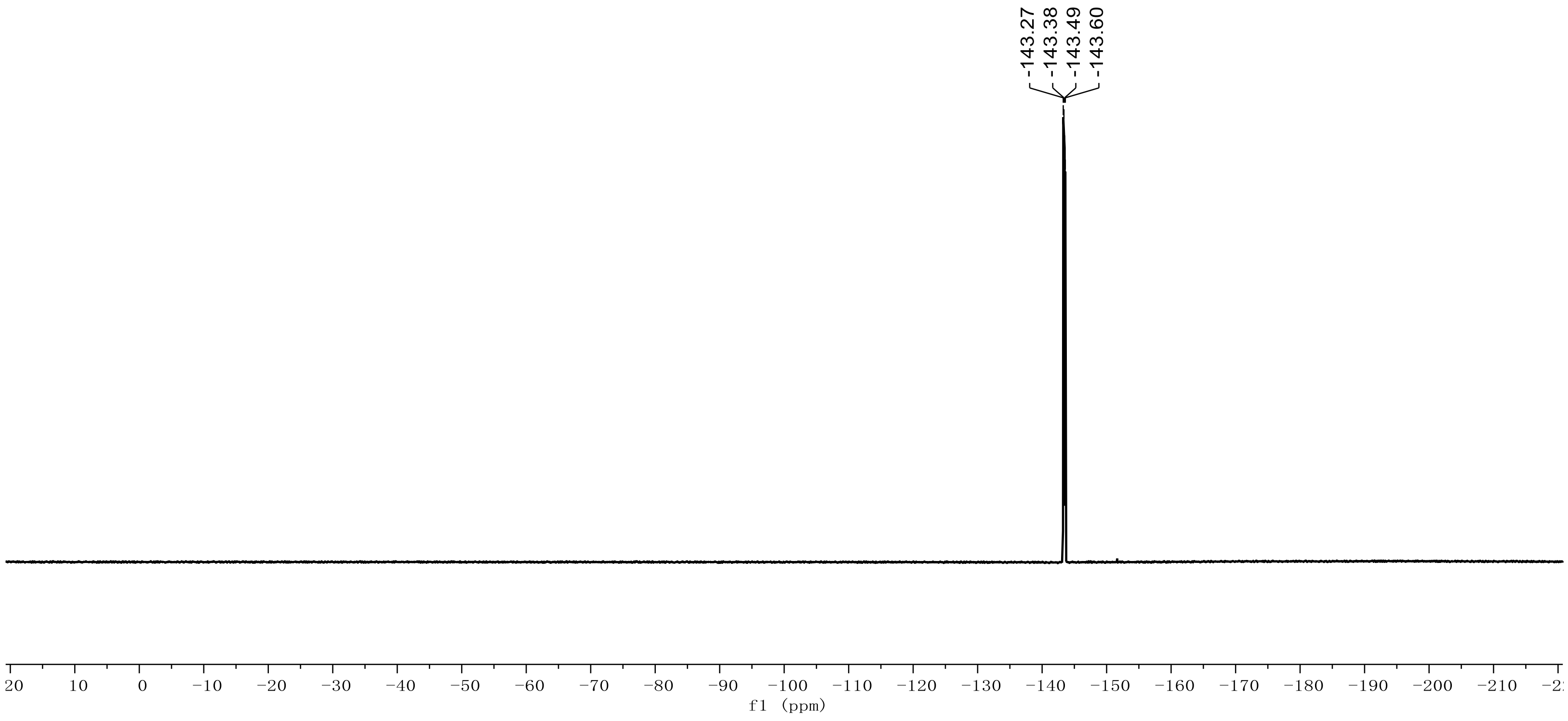

^{11}B NMR of 1t

CD_3CN, 128 MHz, 25 °C

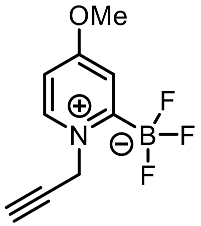

1t

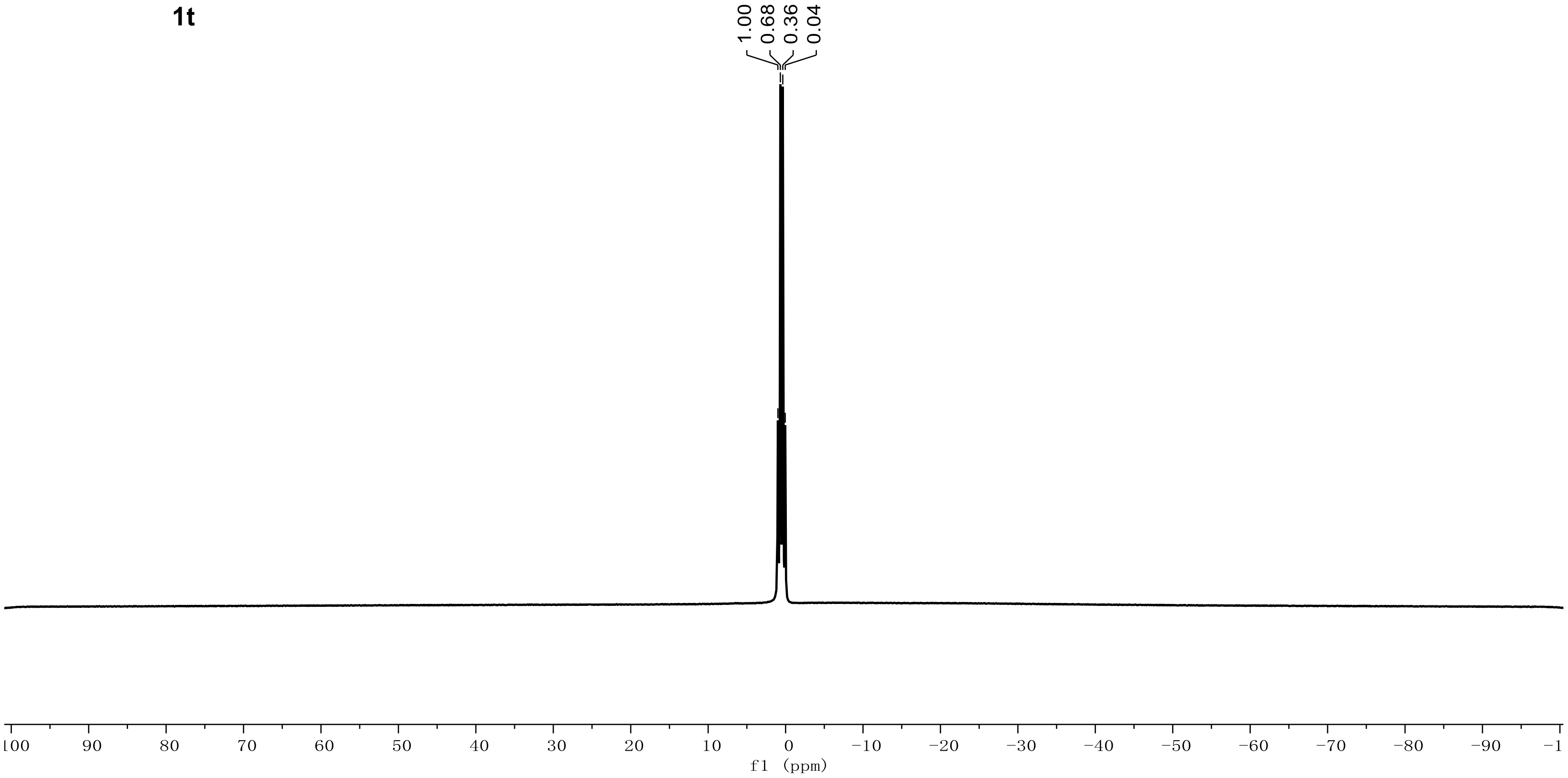

^{13}C NMR of 1t

CD_3CN, 101 MHz, 25 °C

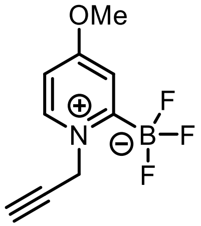

1t

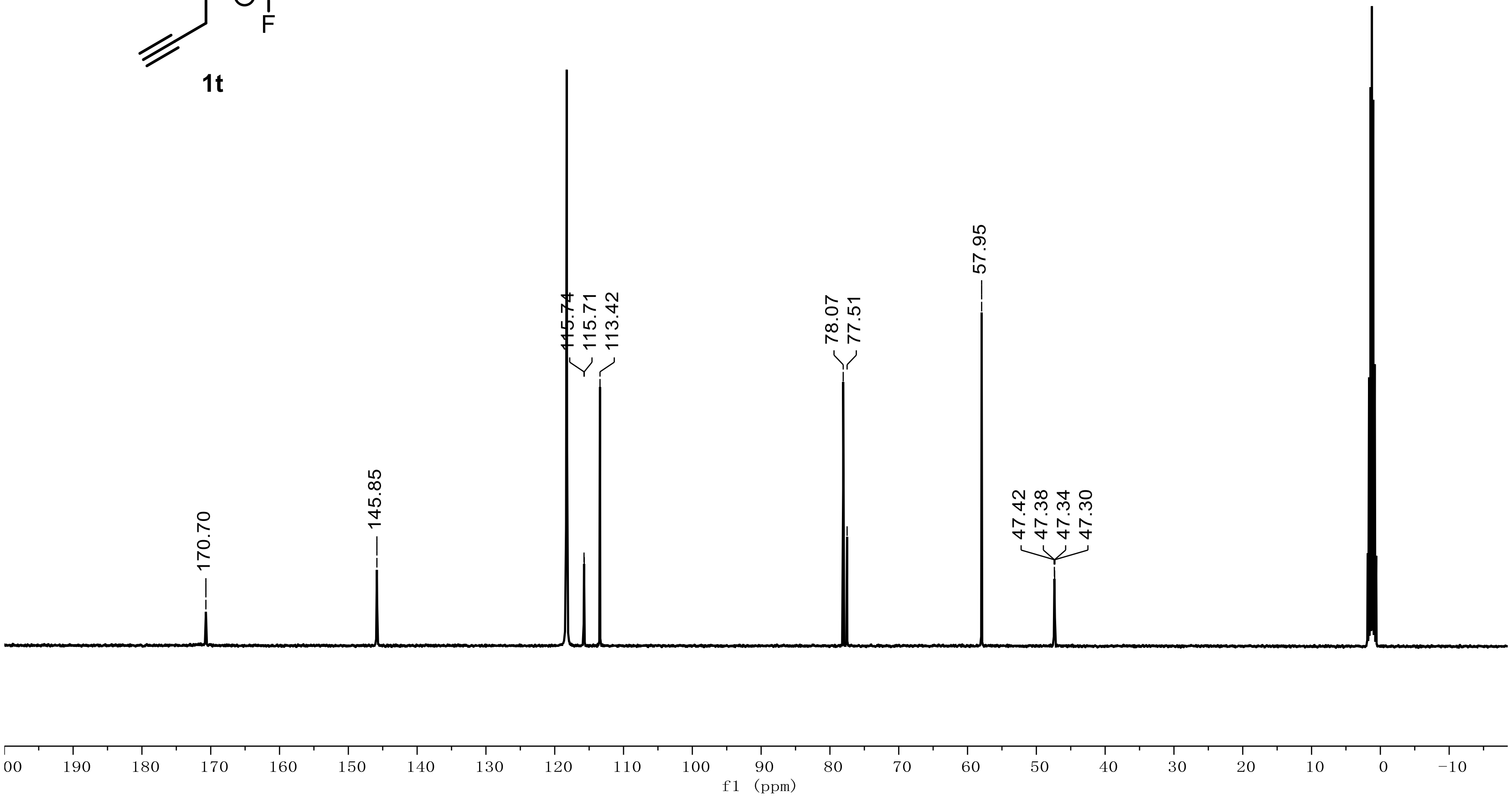

^{1}H NMR of 1b-S

CD_3CN, 400 MHz, 25 °C

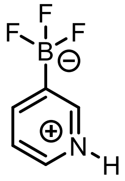

1b-S

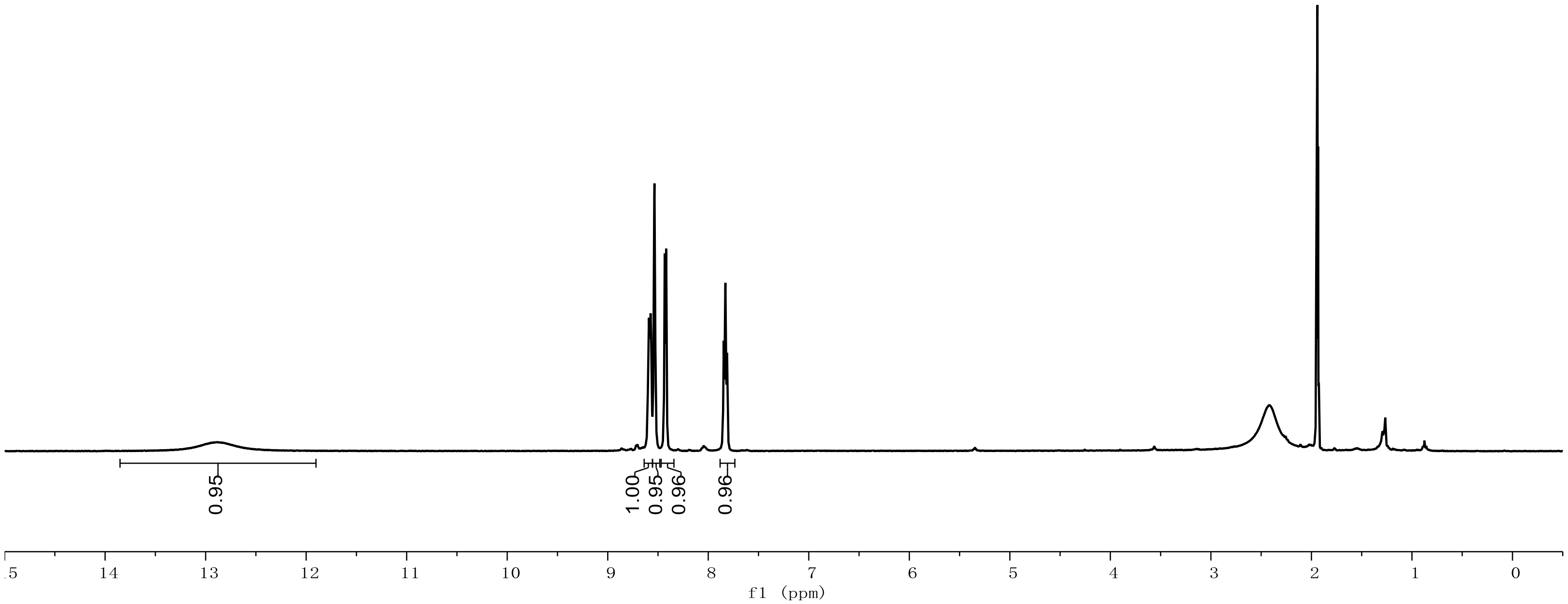

^{19}F NMR of 1b-S

CD_3CN, 376 MHz, 25 °C

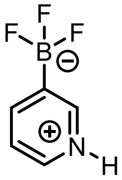

1b-S

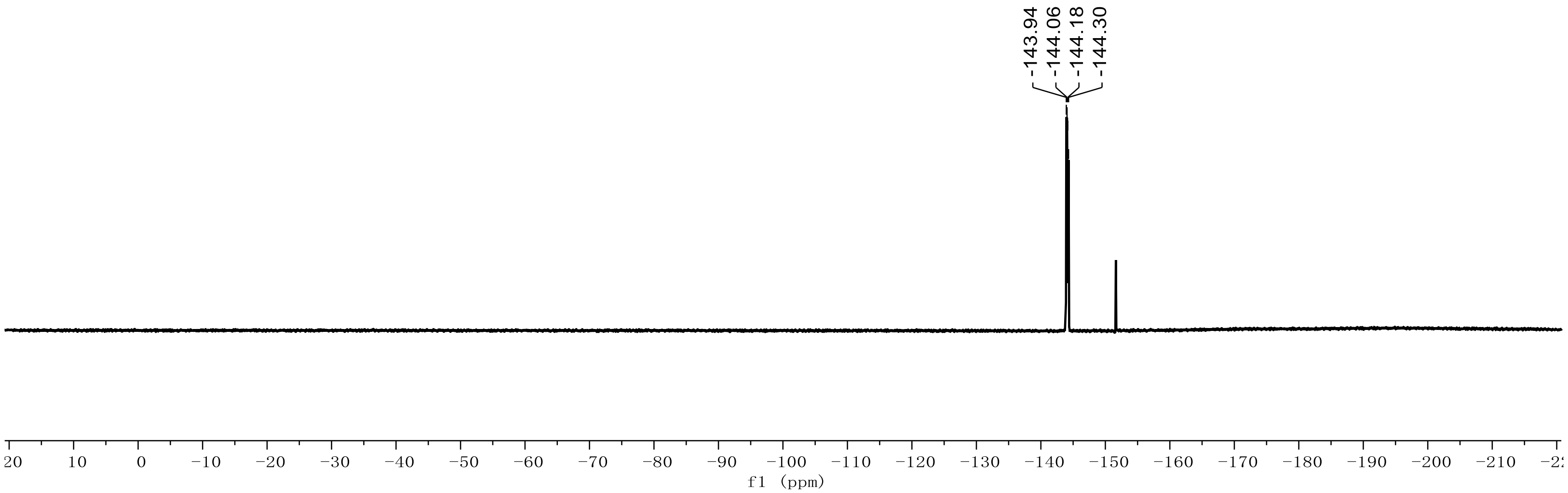

^{11}B NMR of 1b-S

CD_3CN, 128 MHz, 25 °C

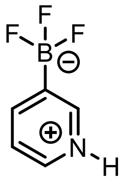

1b-S

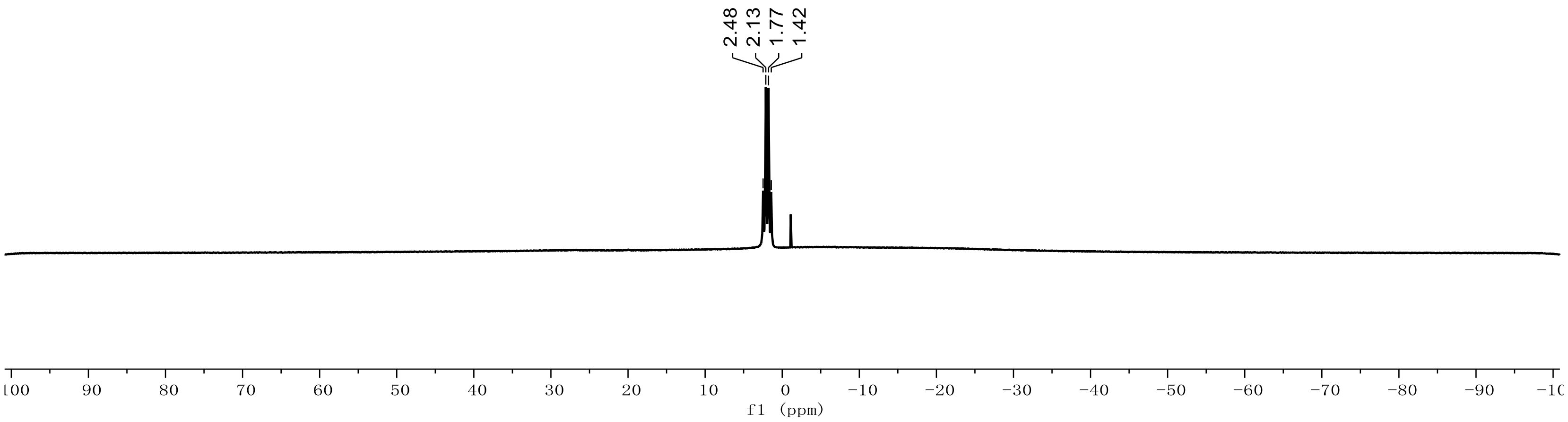

^{13}C NMR of 1b-S

CD_3CN, 101 MHz, 25 °C

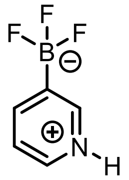

1b-S

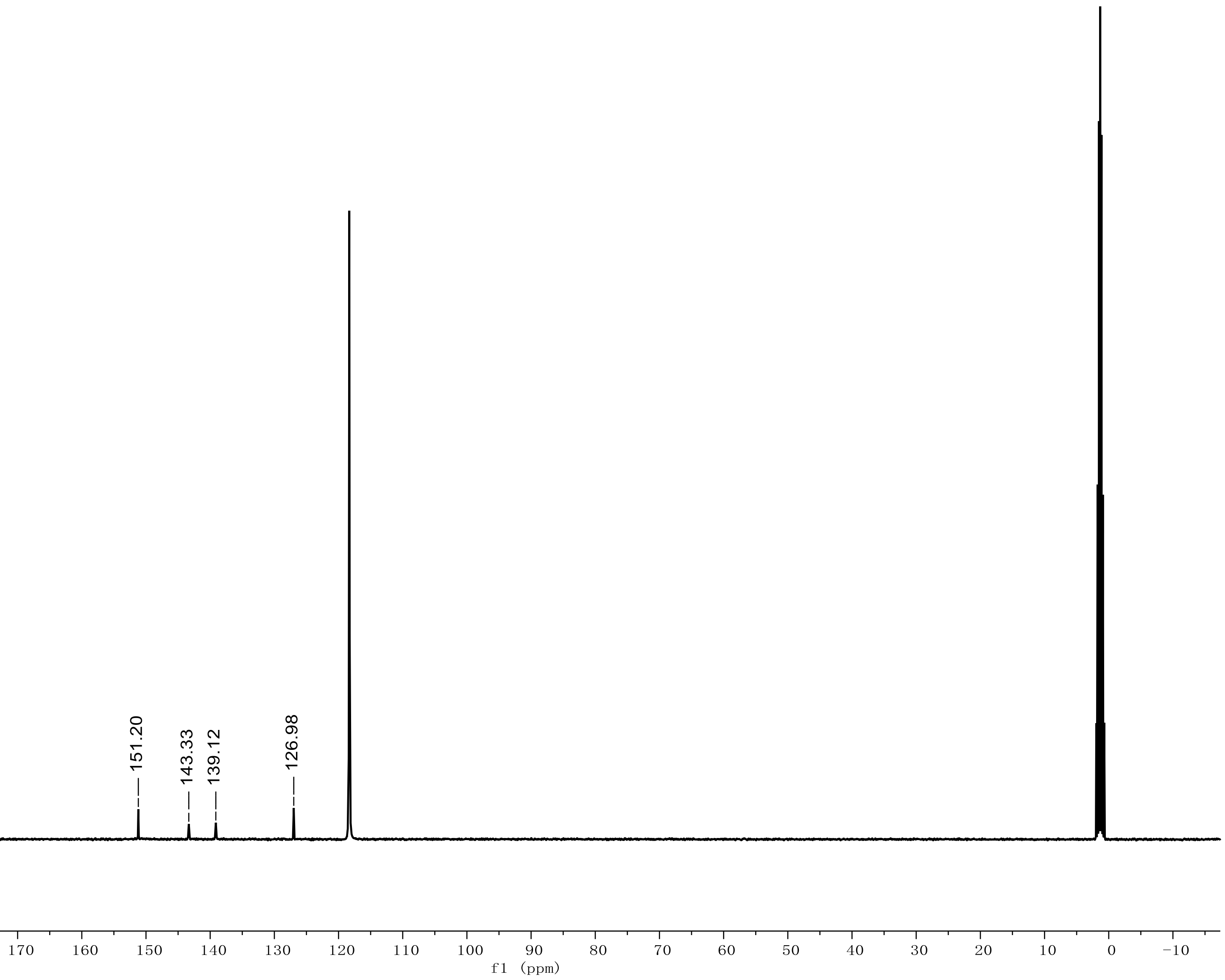

^{1}H NMR of 1c-S

CD_3CN, 400 MHz, 25 °C

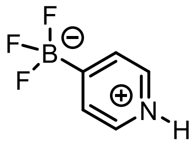

1c-S

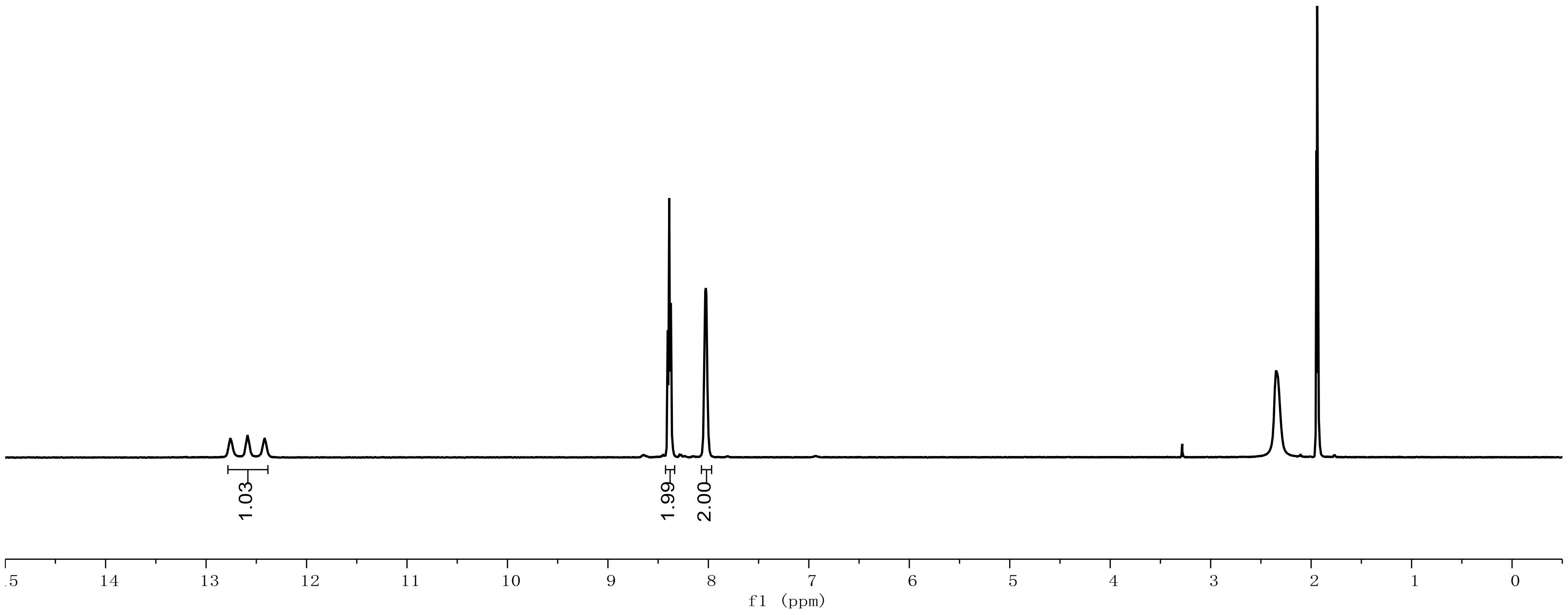

^{19}F NMR of 1c-S

CD_3CN, 376 MHz, 25 °C

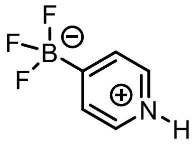

1c-S

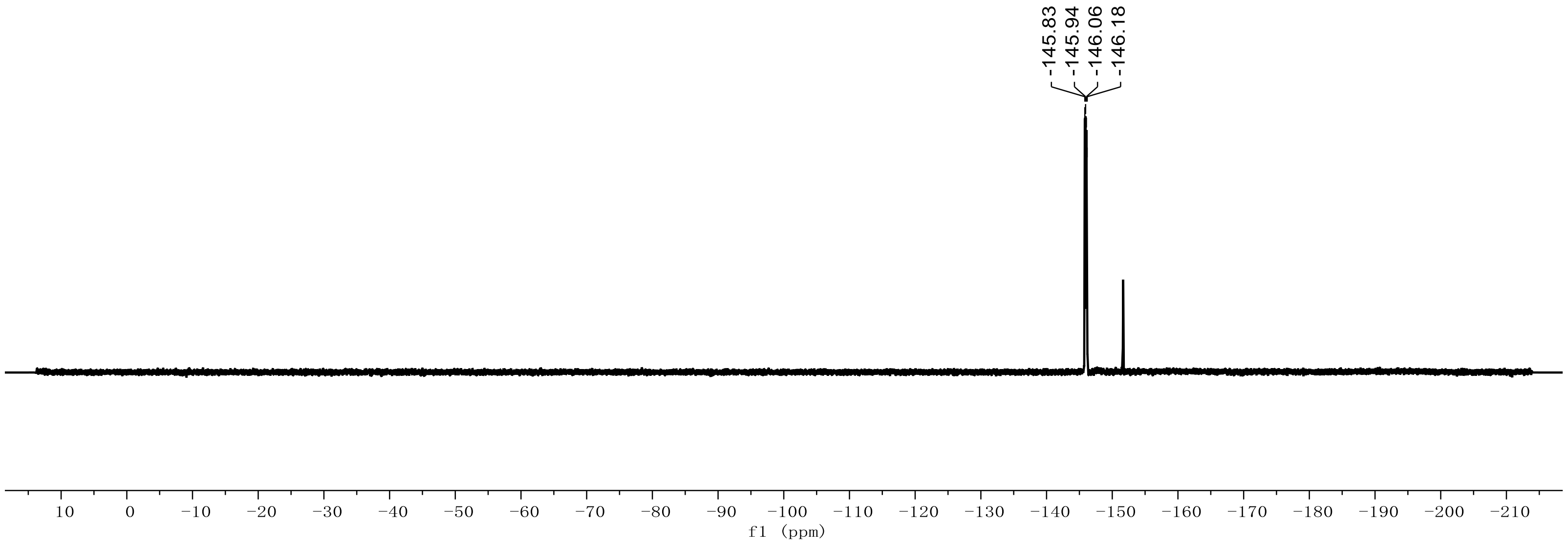

^{11}B NMR of 1c-S

CD_3CN, 128 MHz, 25 °C

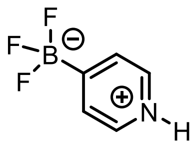

1c-S

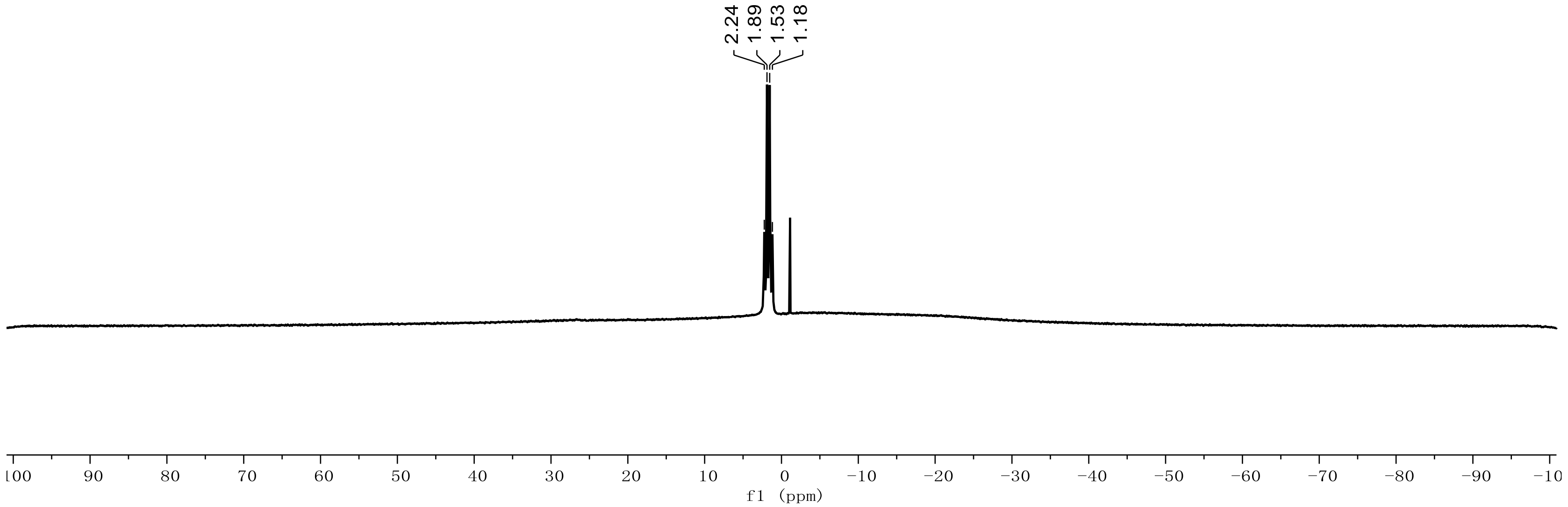

^{13}C NMR of 1c-S

CD_3CN, 101 MHz, 25 °C

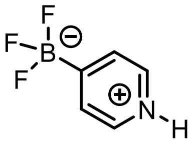

1c-S

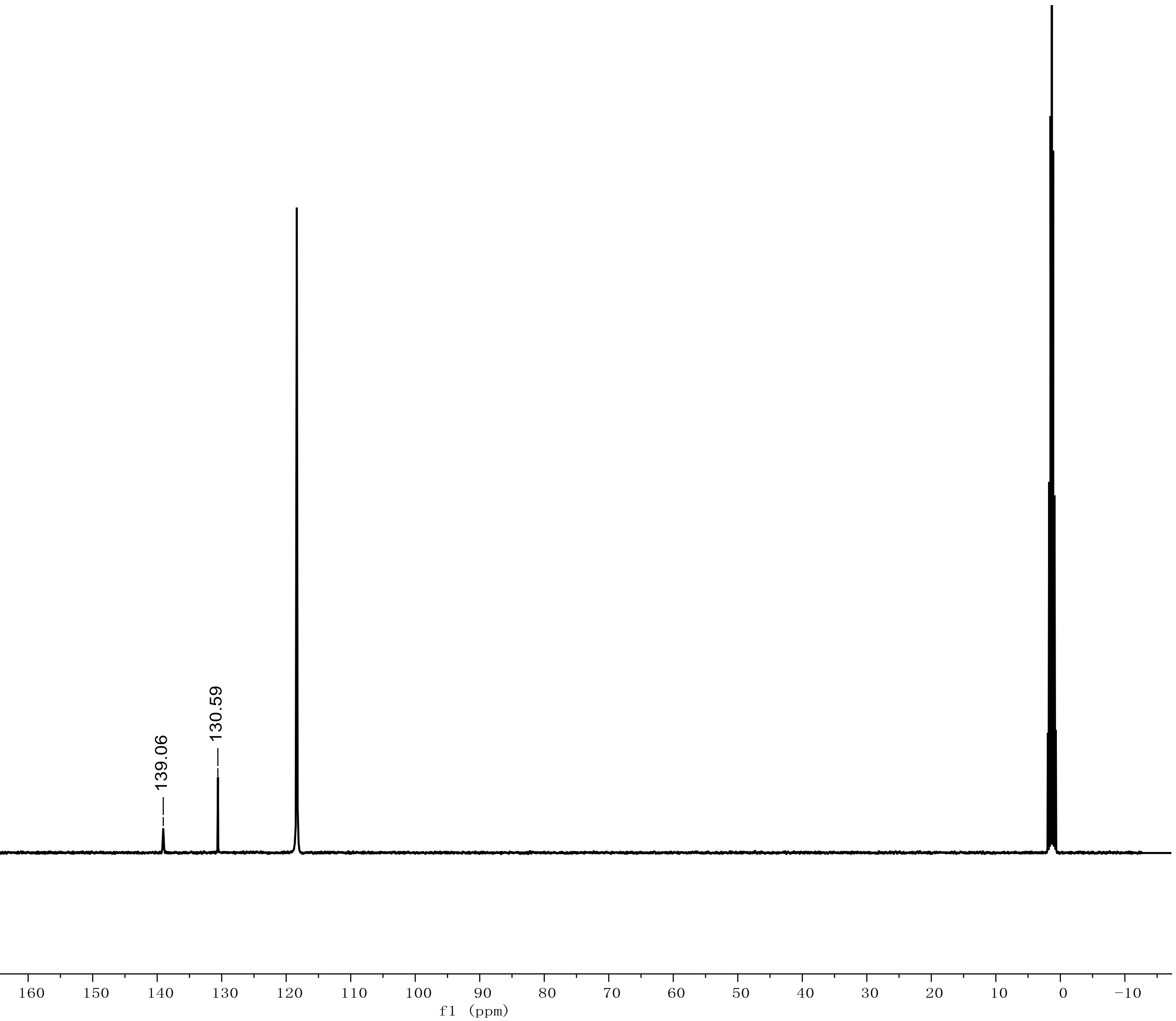

^{1}H NMR of 1d-S

CD_3CN, 400 MHz, 25 °C

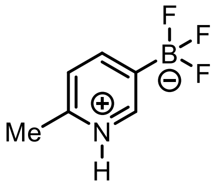

1d-S

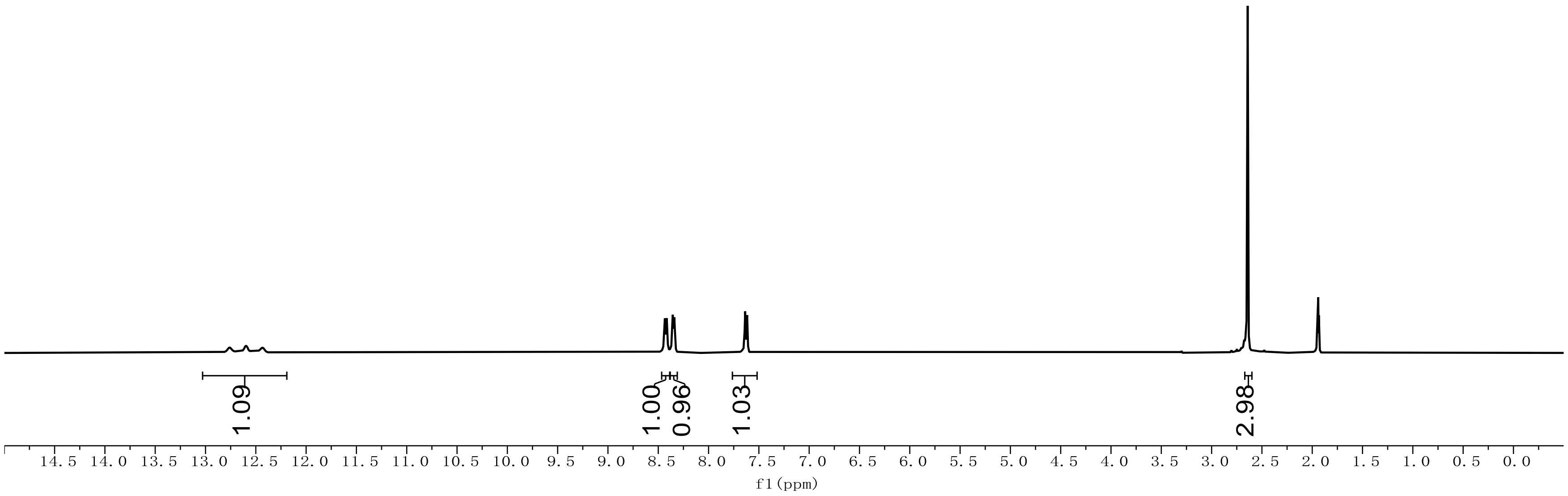

^{19}F NMR of 1d-S

CD_3CN, 376 MHz, 25 °C

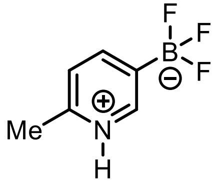

1d-S

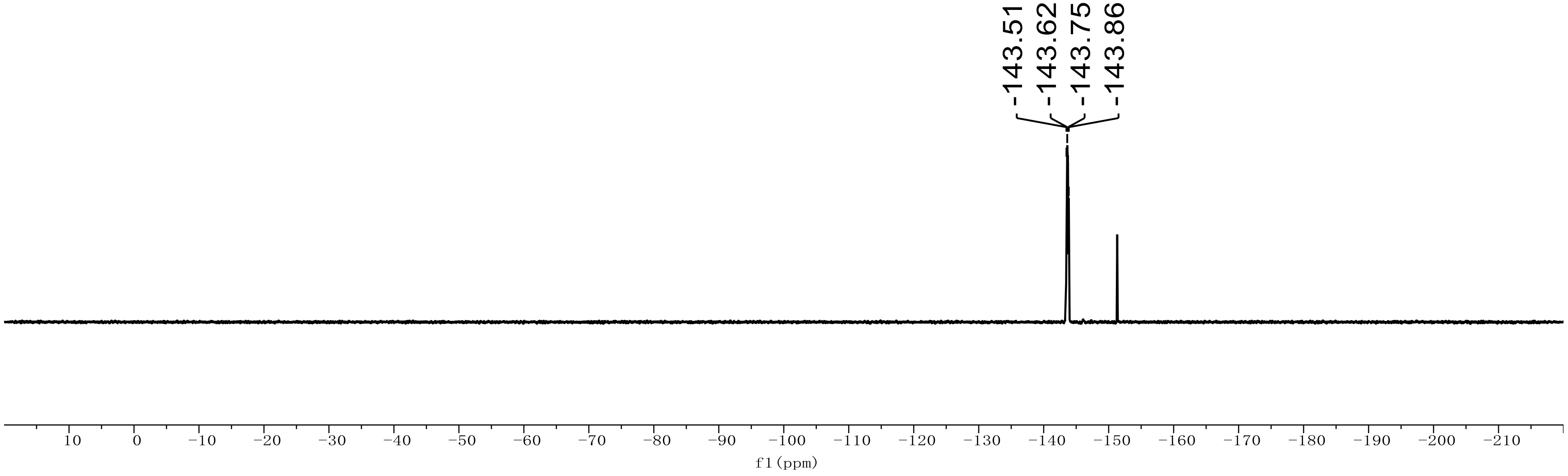

^{11}B NMR of 1d-S

CD_3CN, 128 MHz, 25 °C

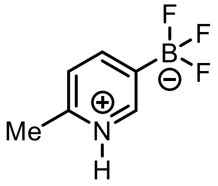

1d-S

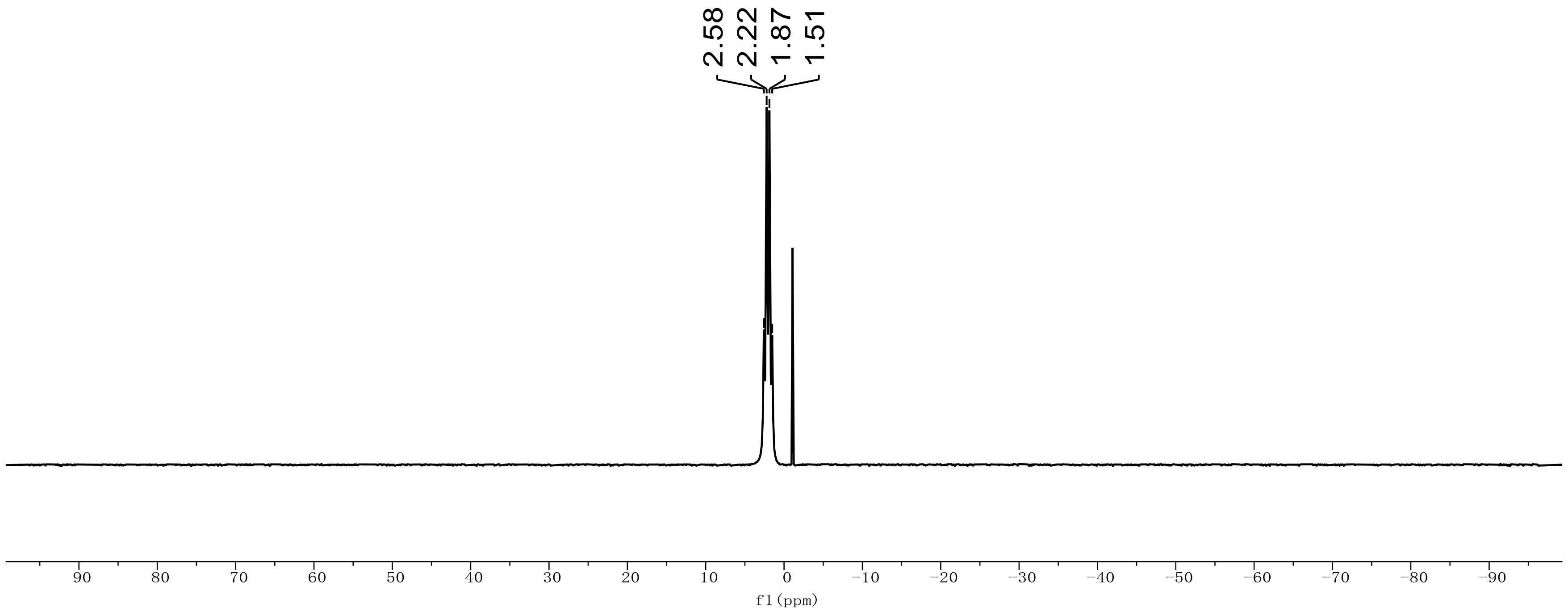

^{13}C NMR of 1d-S

CD_3CN, 101 MHz, 25 °C

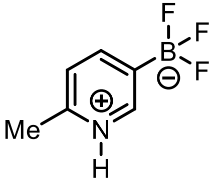

1d-S

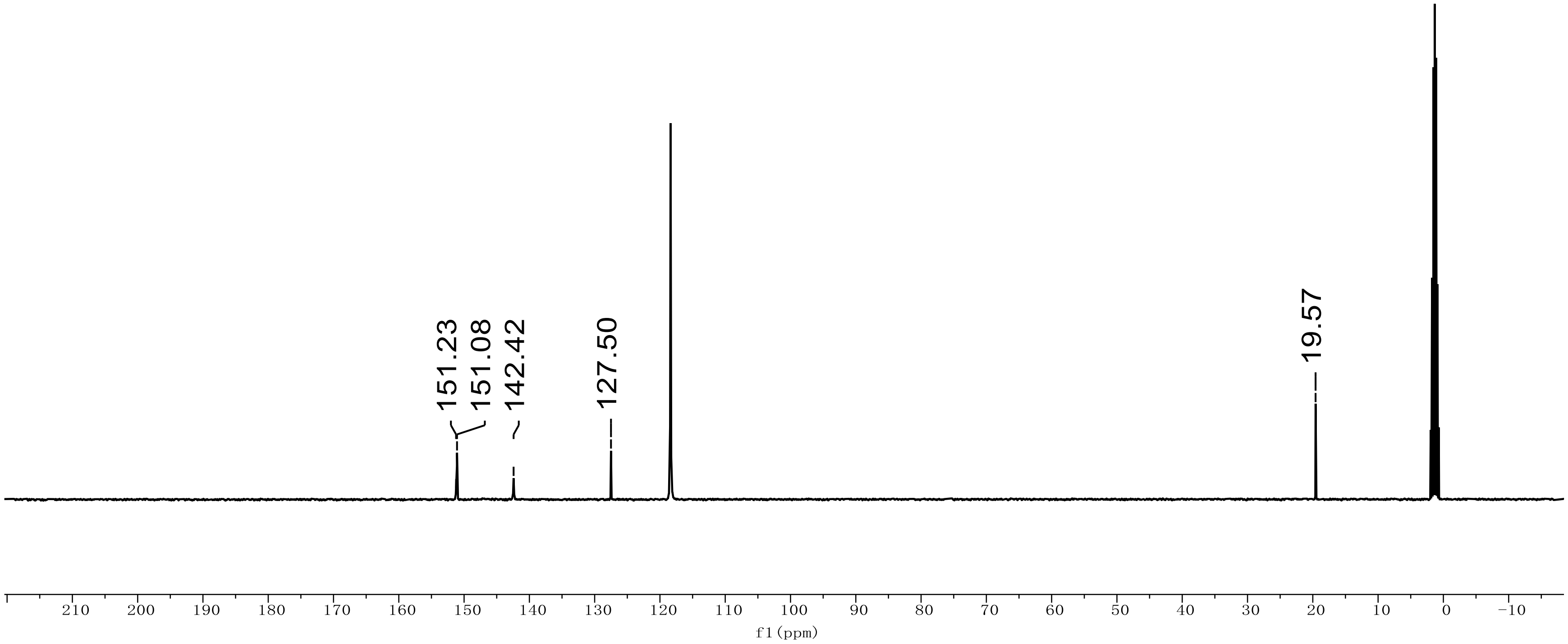

^{1}H NMR of 1e-S

CD3CN, 400 MHz, 25 °C

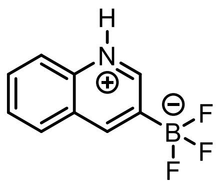

1e-S

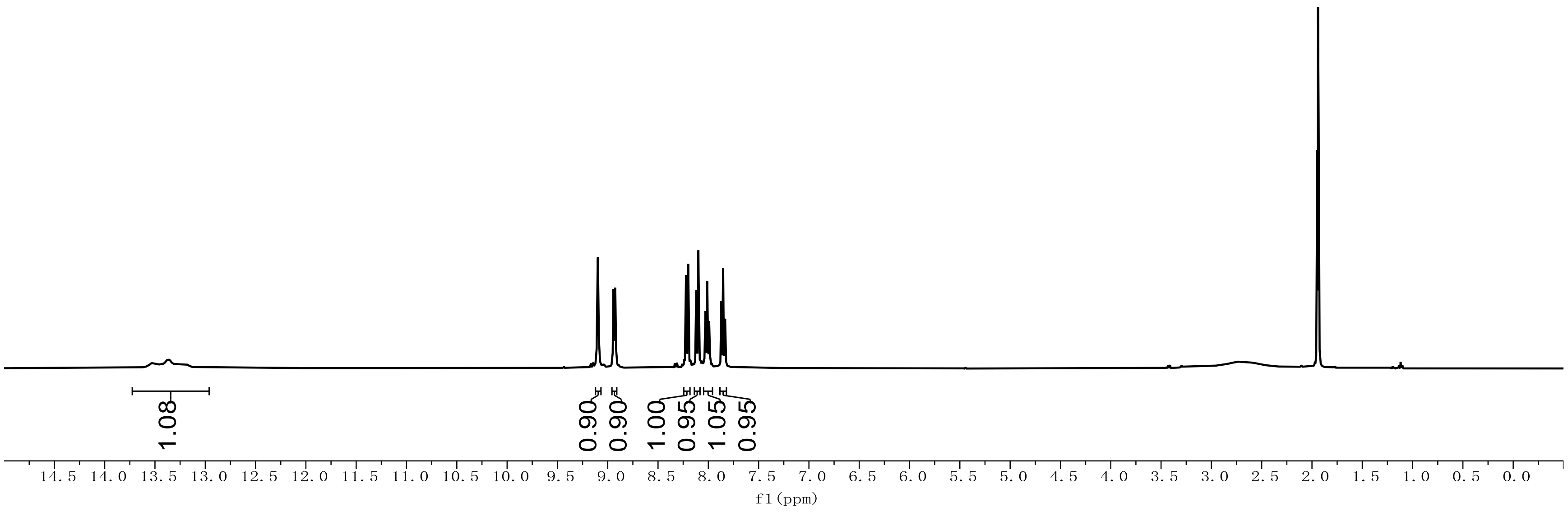

^{19}F NMR of 1e-S

DMSO-d_6, 376 MHz, 25 °C

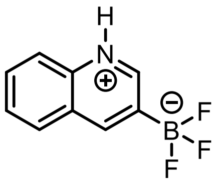

1e-S

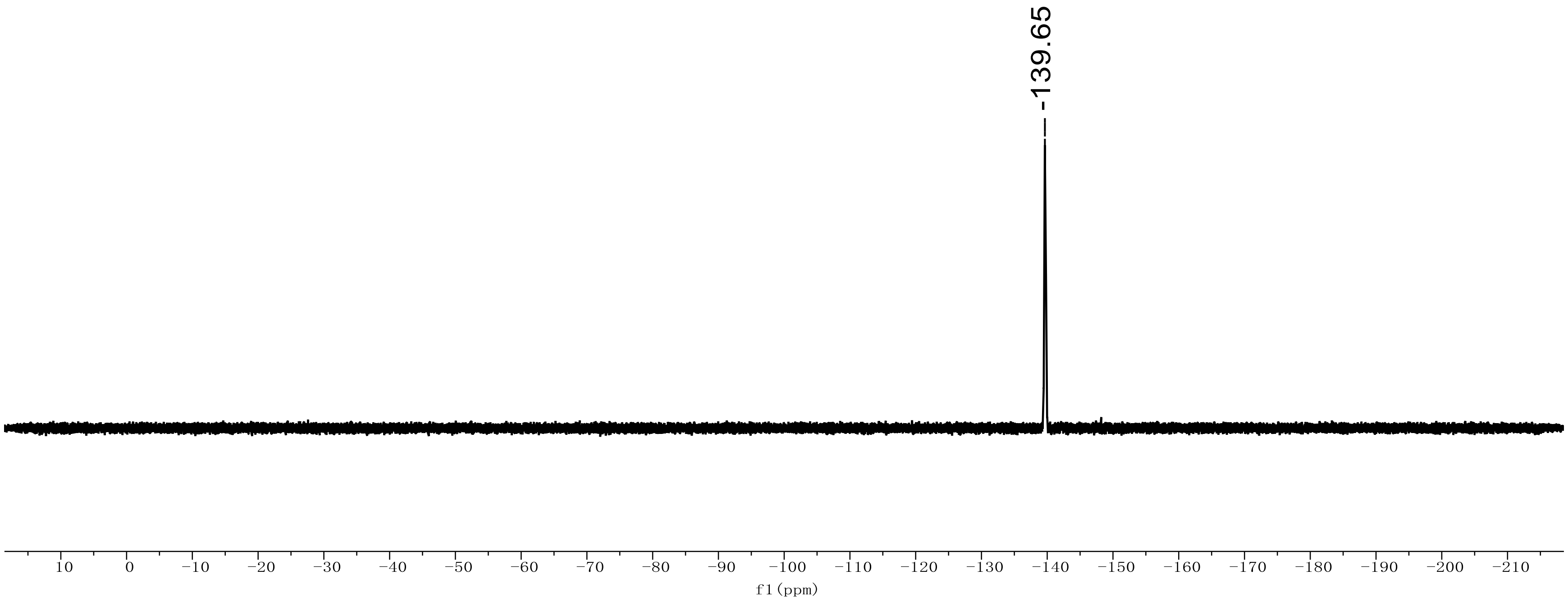

^{11}B NMR of 1e-S

DMSO-d_6, 128 MHz, 25 °C

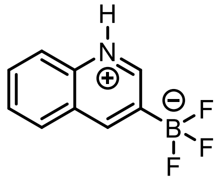

1e-S

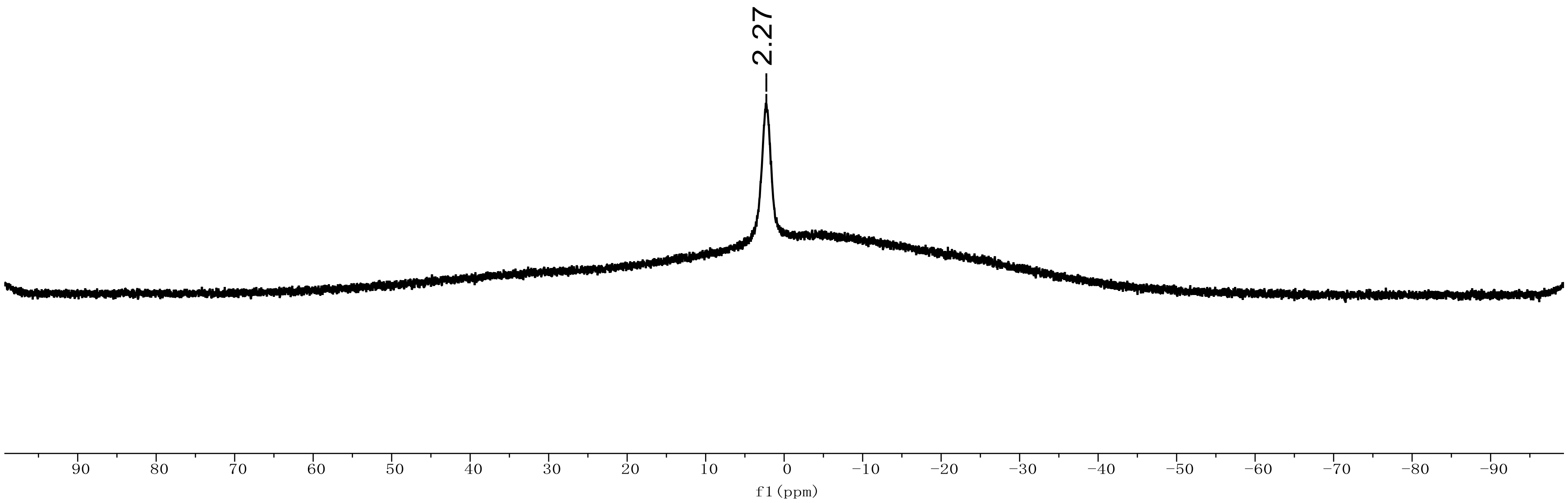

^{13}C NMR of 1e-S

DMSO-d_6, 101 MHz, 25 °C

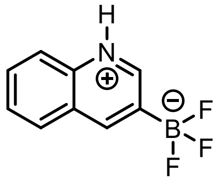

1e-S

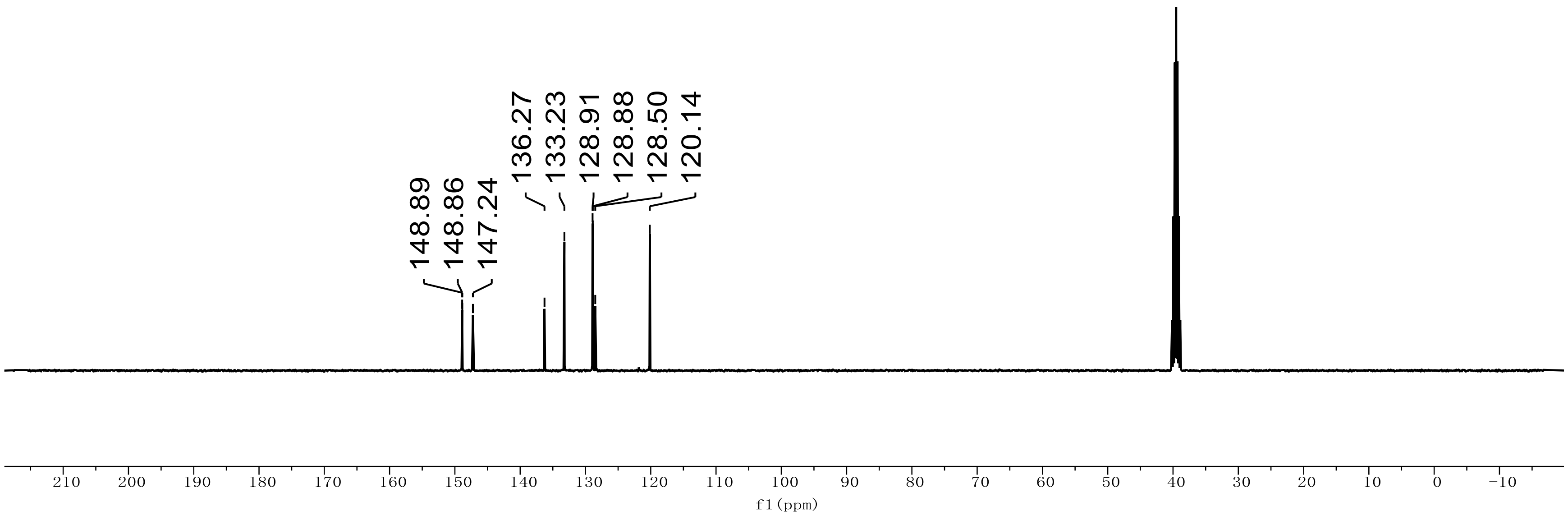

^{1}H NMR of 1f-S

CD_3CN, 400 MHz, 25 °C

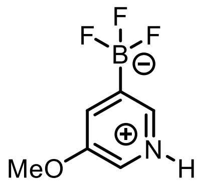

1f-S

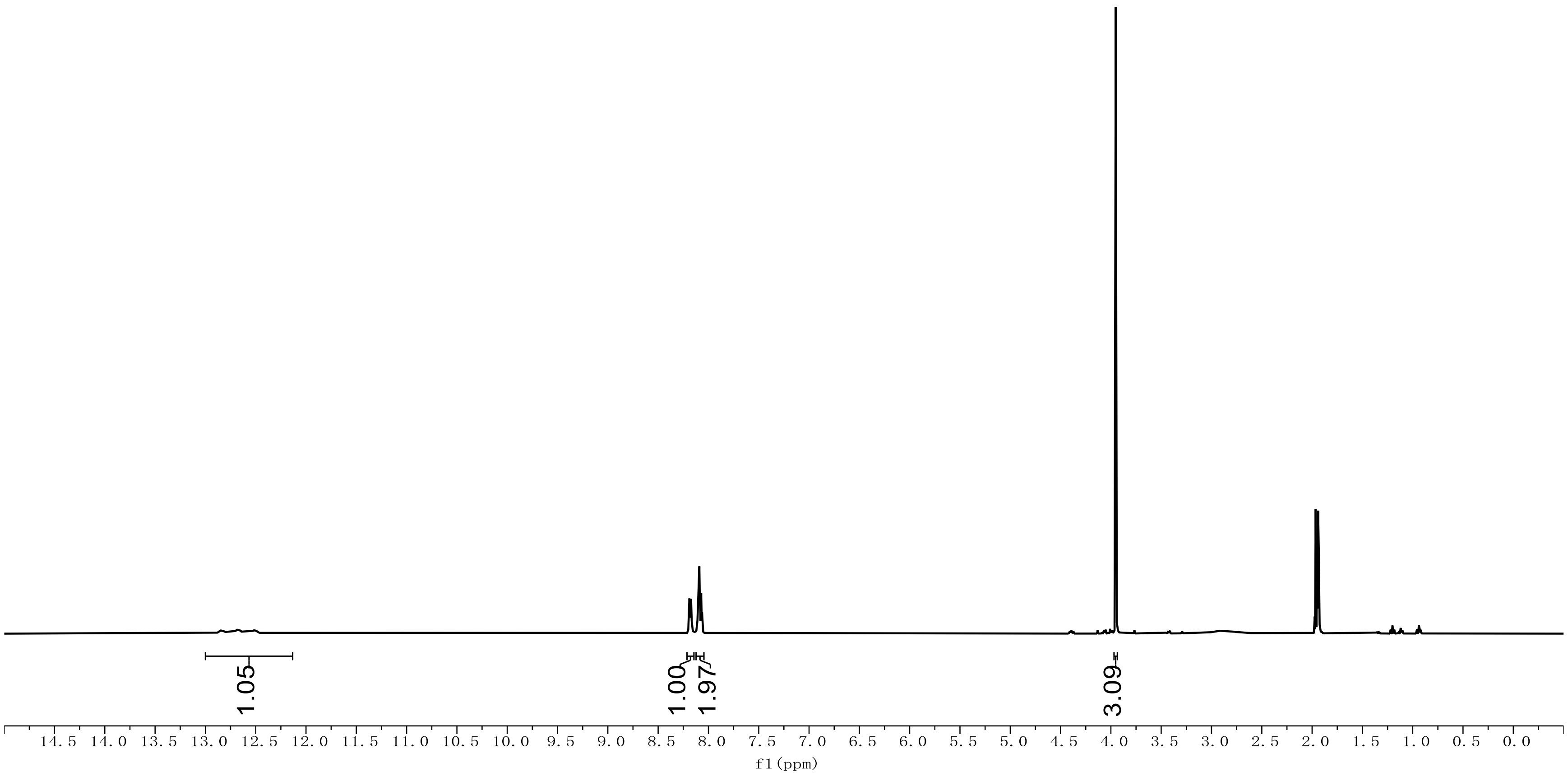

^{19}F NMR of 1f-S

DMSO-d_6, 376 MHz, 25 °C

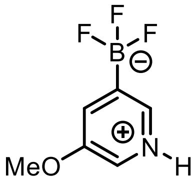

1f-S

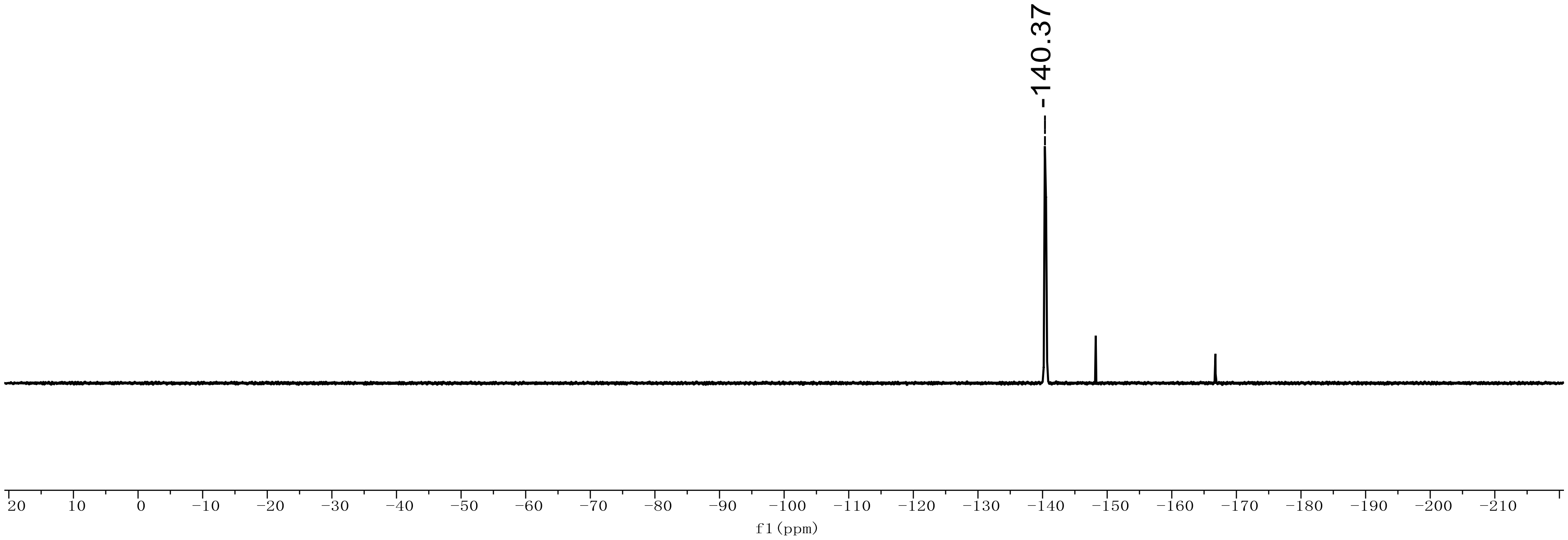

^{11}B NMR of 1f-S

DMSO-d_6, 128 MHz, 25 °C

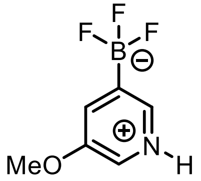

1f-S

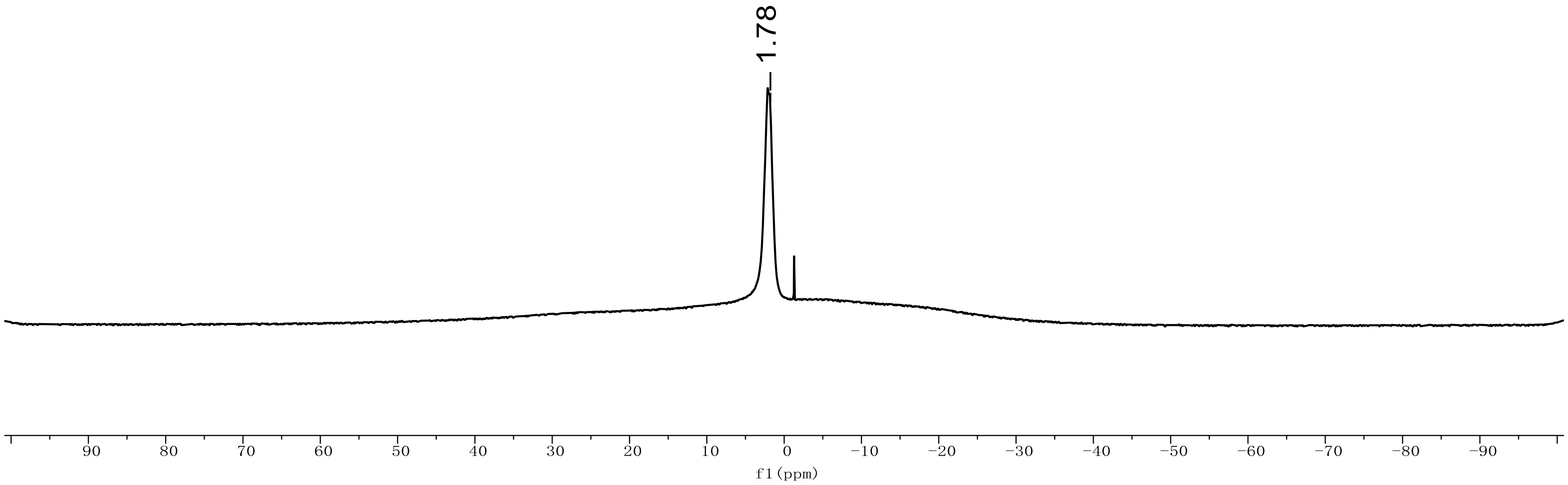

^{13}C NMR of 1f-S

DMSO-d_6, 101 MHz, 25 °C

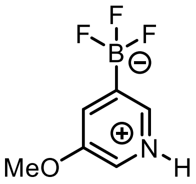

1f-S

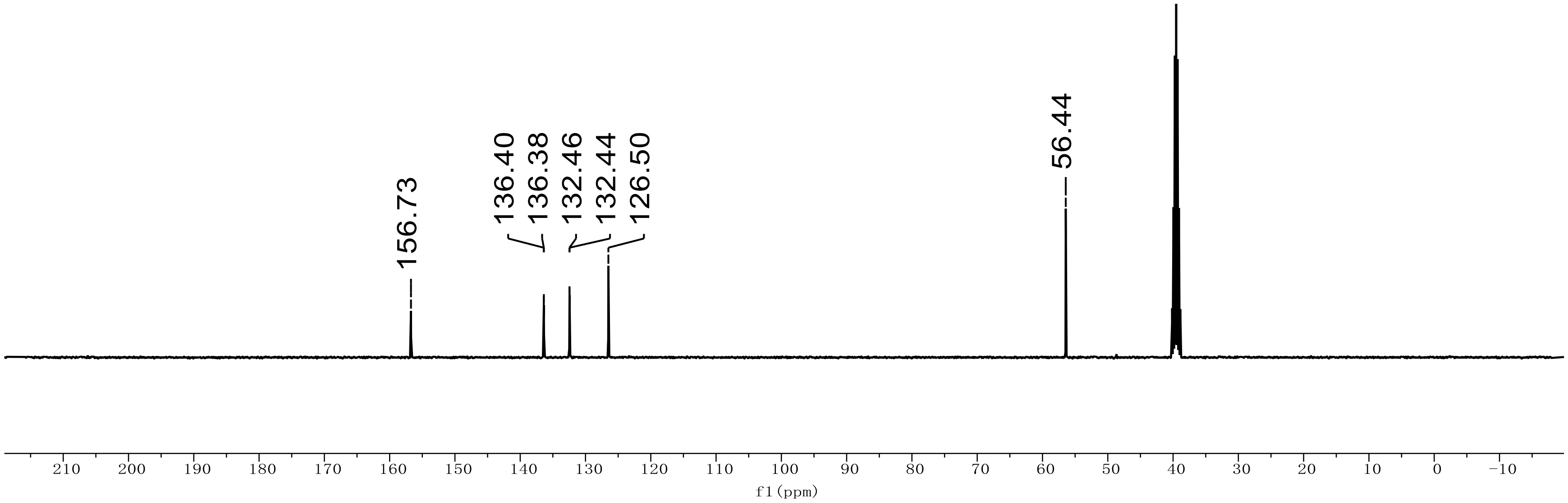

^{1}H NMR of 1g-S

CD_3CN, 400 MHz, 25 °C

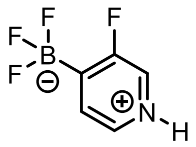

1g-S

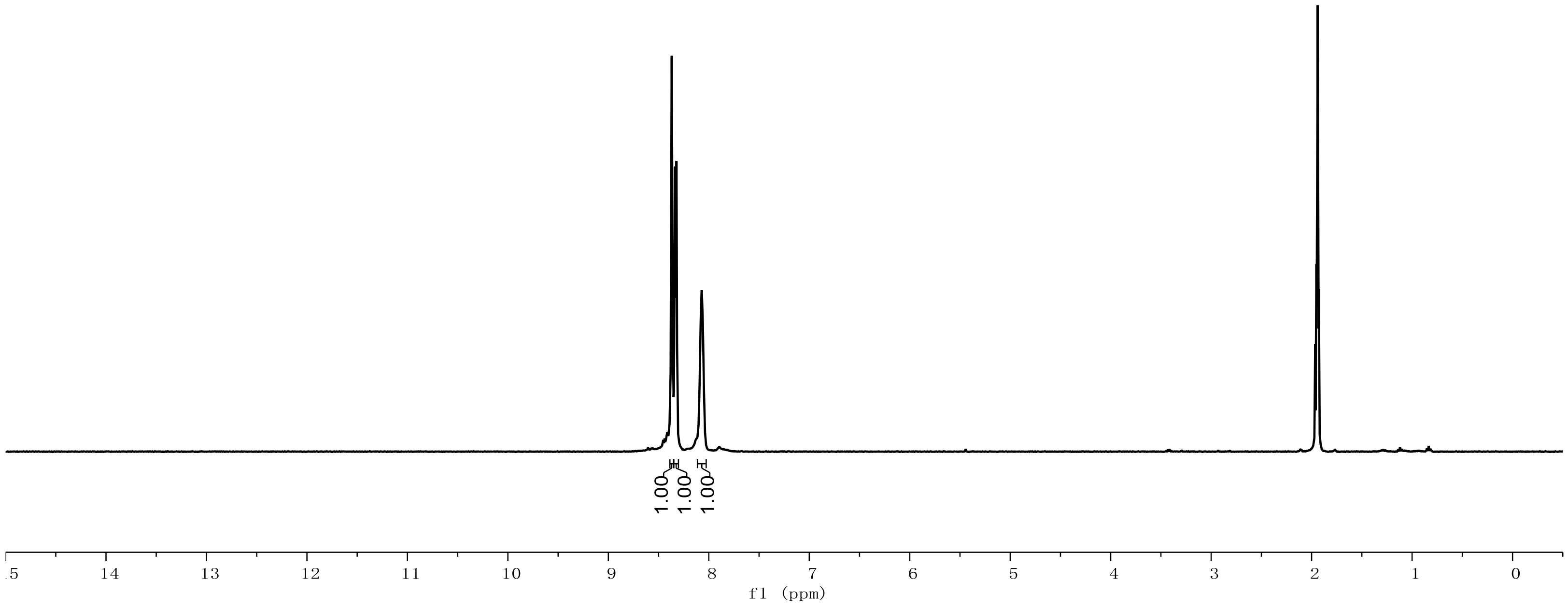

^{19}F NMR of 1g-S

CD_3CN, 376 MHz, 25 °C

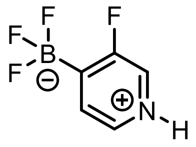

1g-S

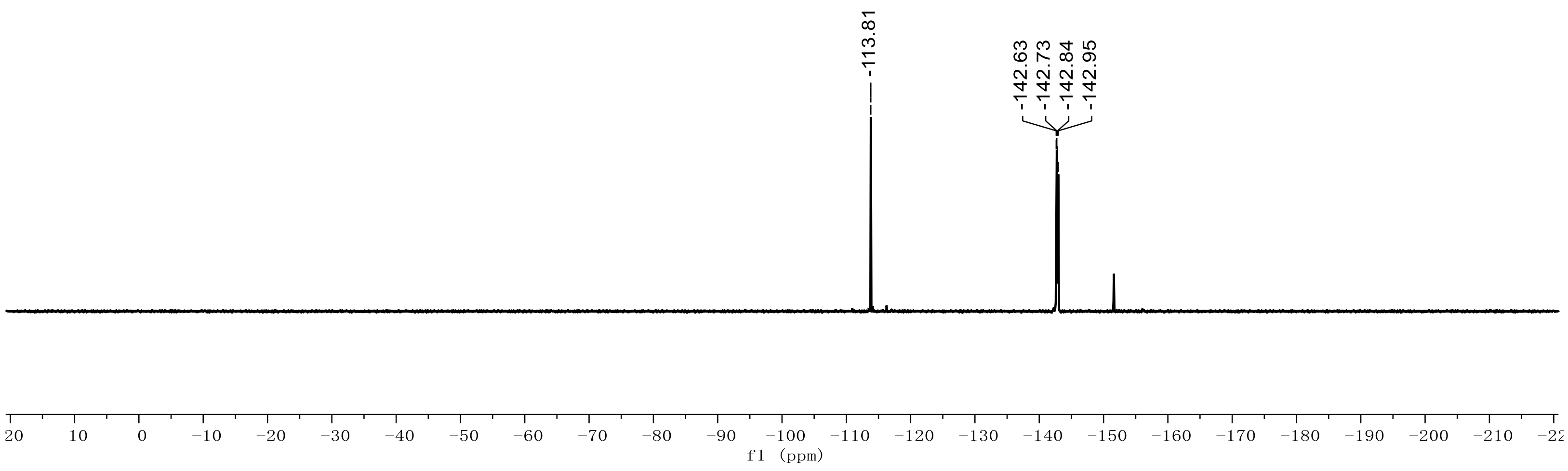

^{11}B NMR of 1g-S

DMSO-d_6, 128 MHz, 25 °C

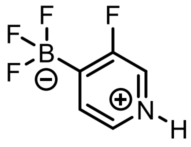

1g-S

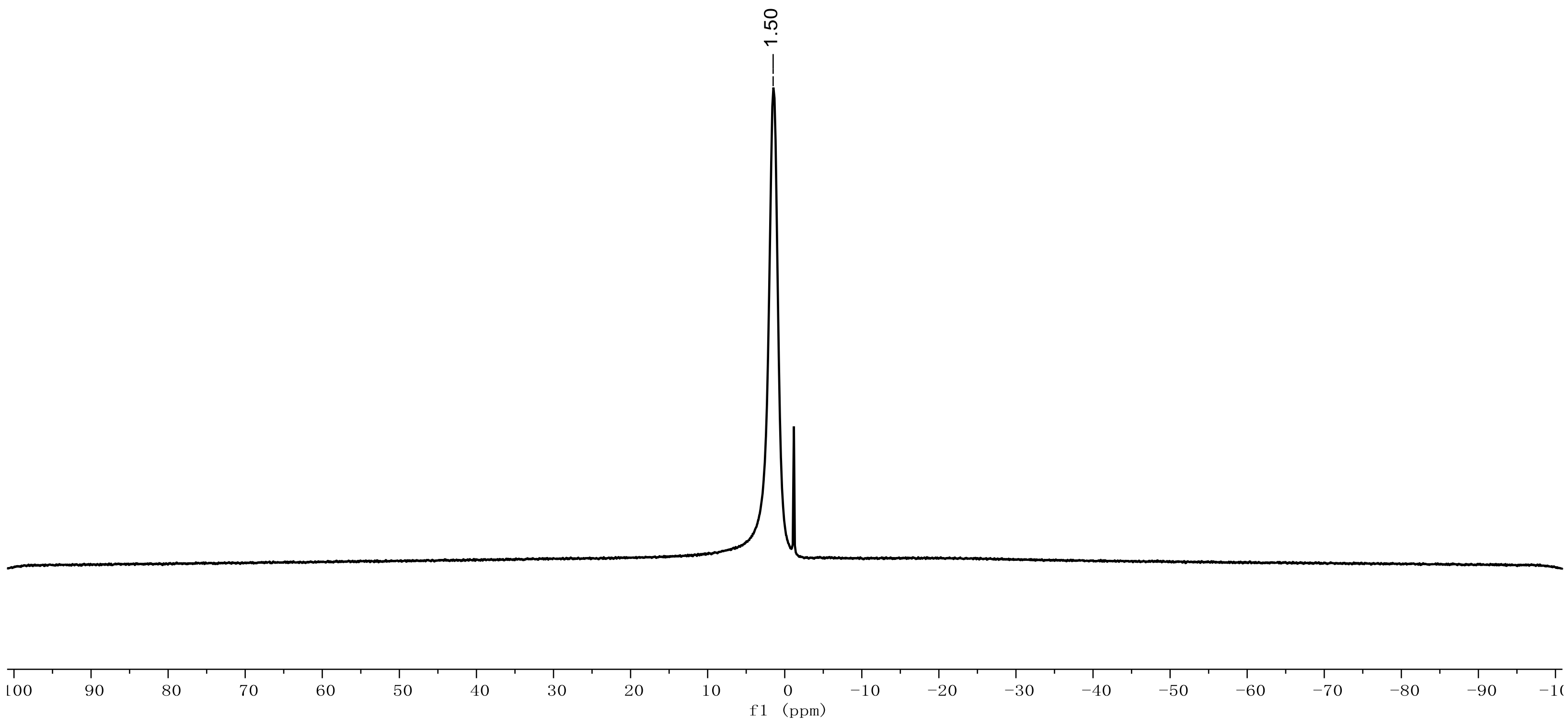

^{13}C NMR of 1g-S

DMSO-d_6, 101 MHz, 25 °C

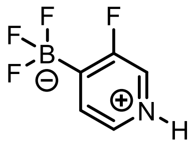

1g-S

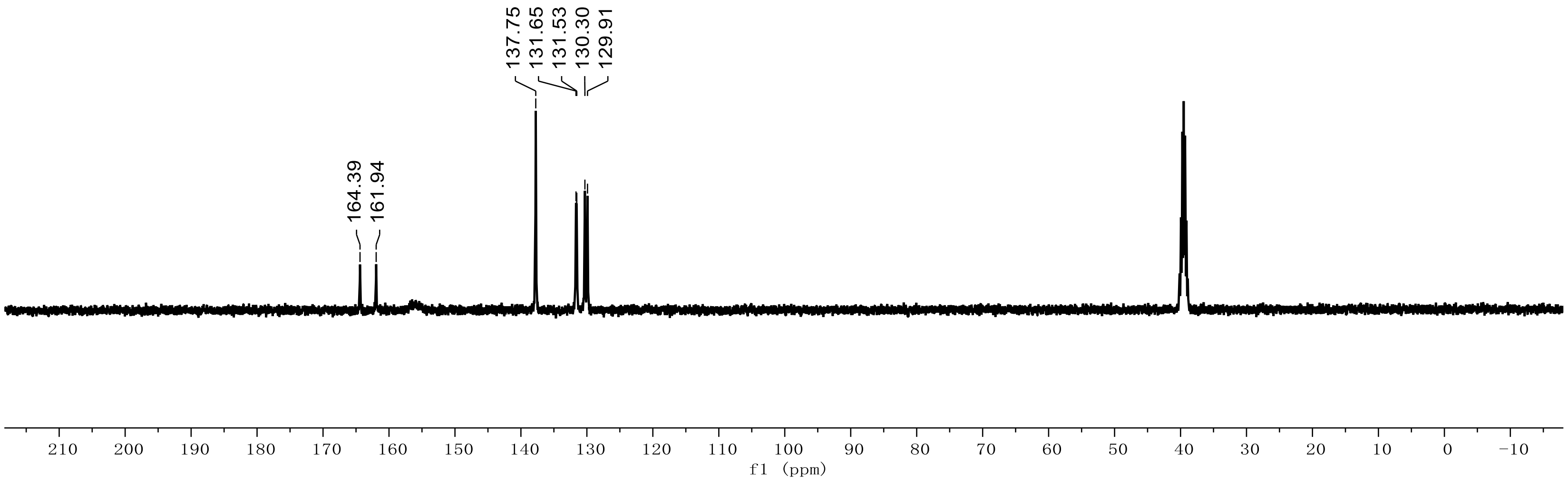

^{1}H NMR of 1h-S

CD_3CN, 400 MHz, 25 °C

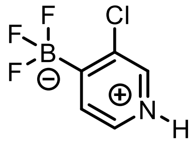

1h-S

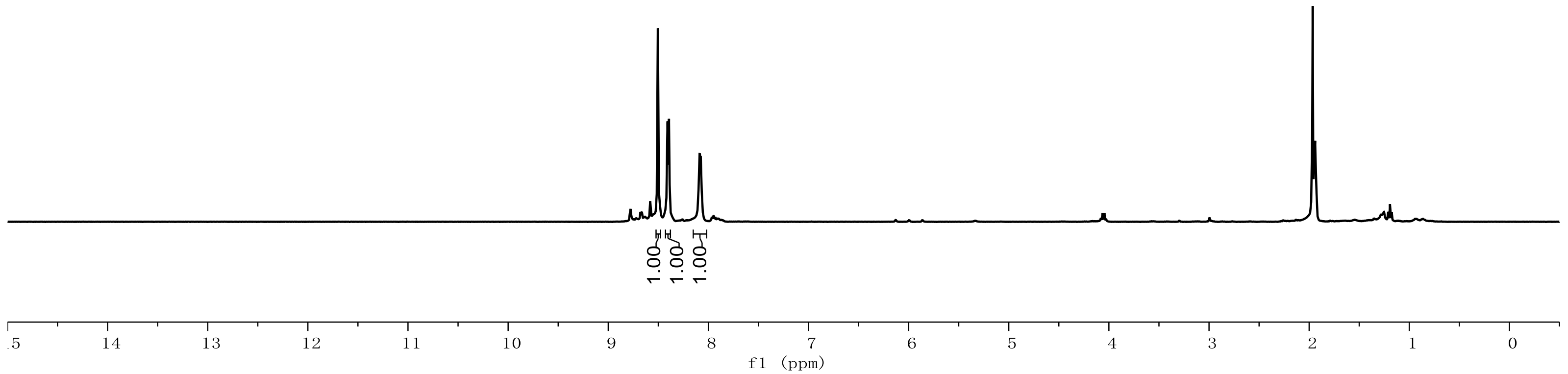

^{19}F NMR of 1h-S

DMSO-d_6, 376 MHz, 25 °C

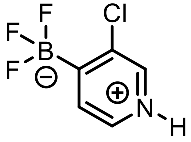

1h-S

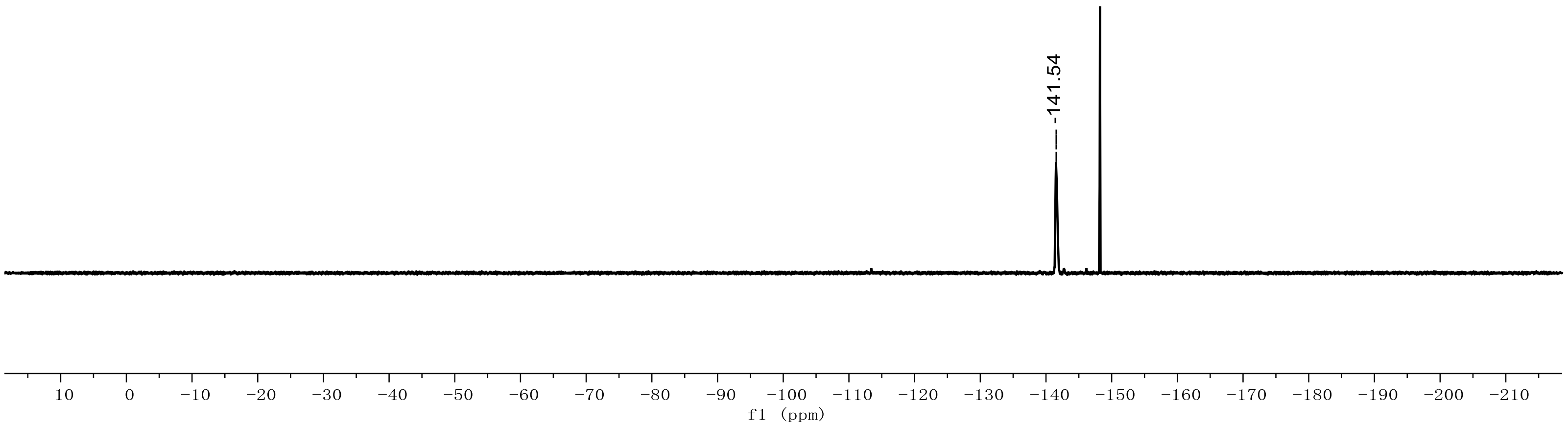

^{11}B NMR of 1h-S

DMSO-d_6, 128 MHz, 25 °C

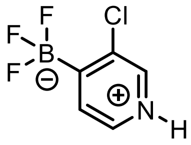

1h-S

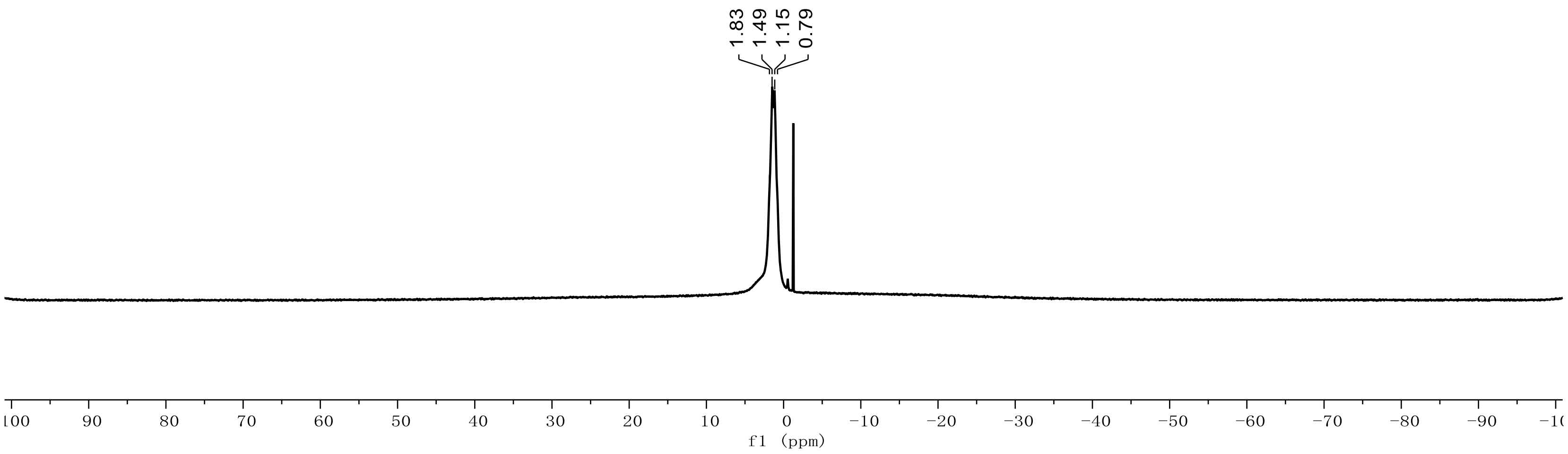

^{13}C NMR of 1h-S

DMSO-d_6, 101 MHz, 25 °C

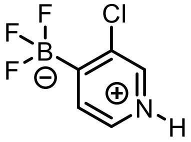

1h-S

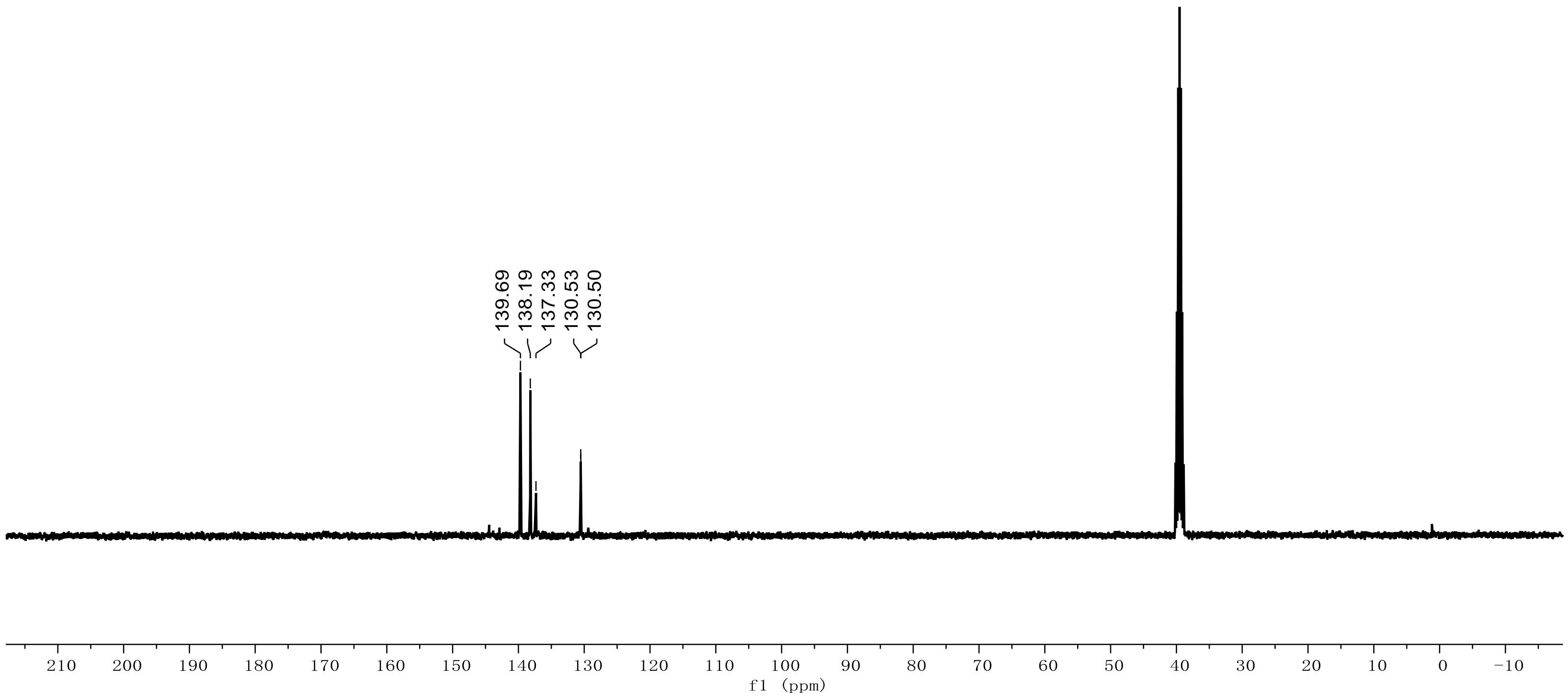

^{1}H NMR of 1i-S

CD_3CN, 400 MHz, 25 °C

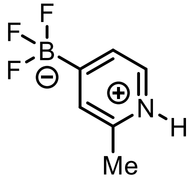

1i-S

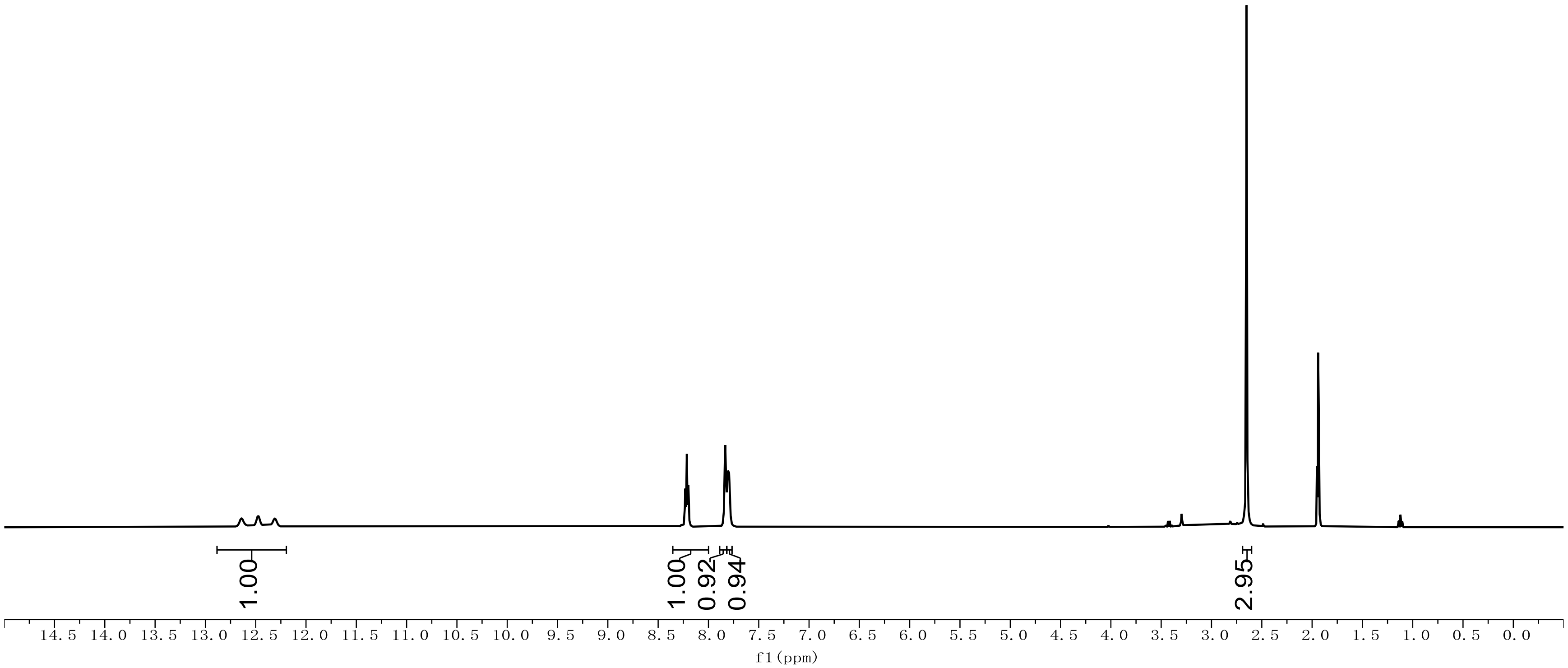

^{19}F NMR of 1i-S

CD_3CN, 376 MHz, 25 °C

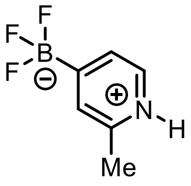

1i-S

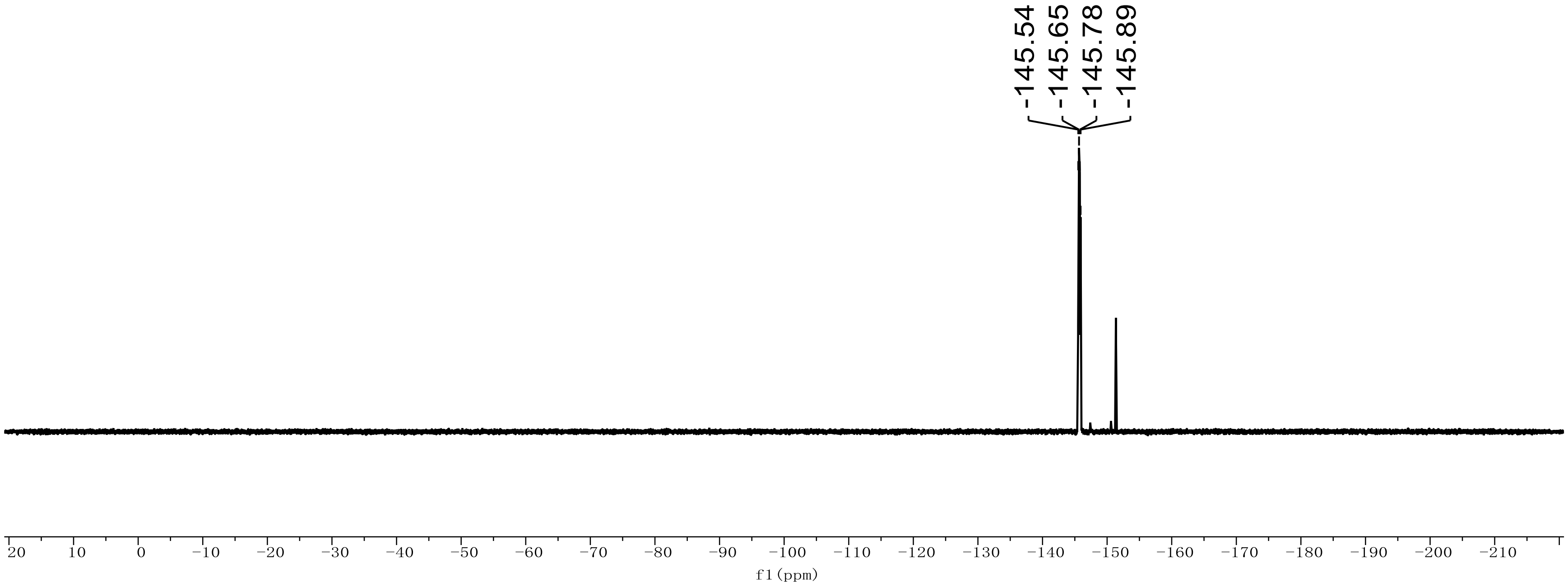

^{11}B NMR of 1i-S

CD_3CN, 128 MHz, 25 °C

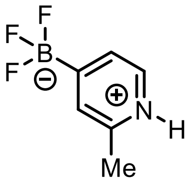

1i-S

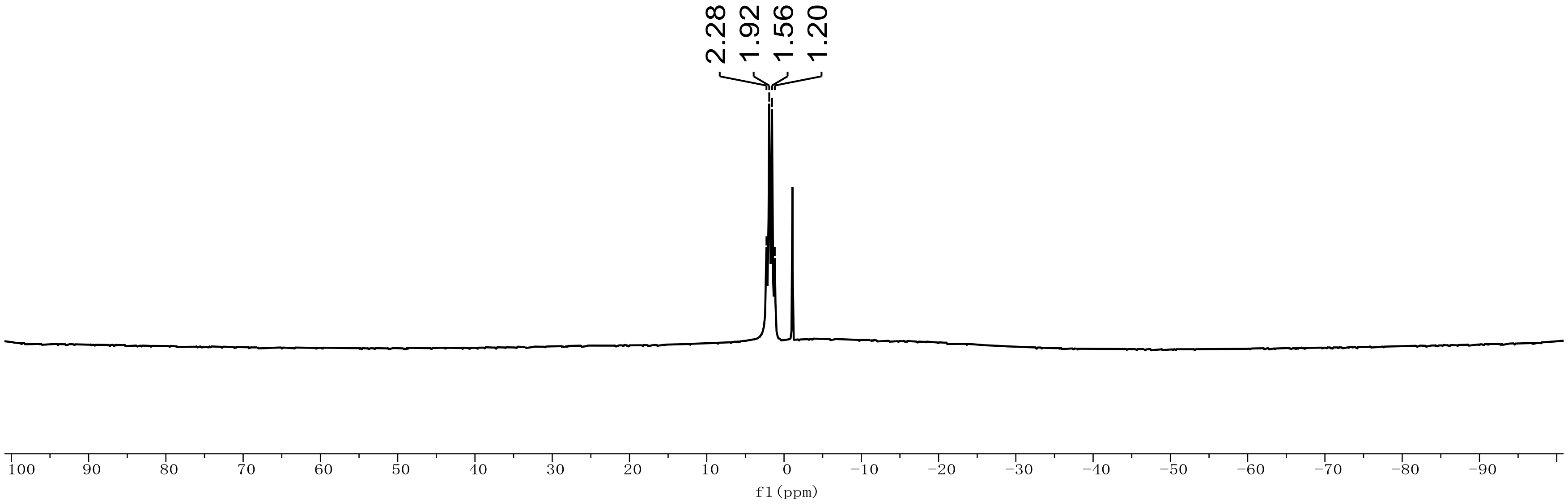

^{13}C NMR of 1i-S

DMSO-d_6, 101 MHz, 25 °C

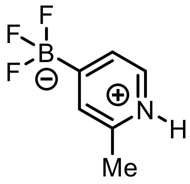

1i-S

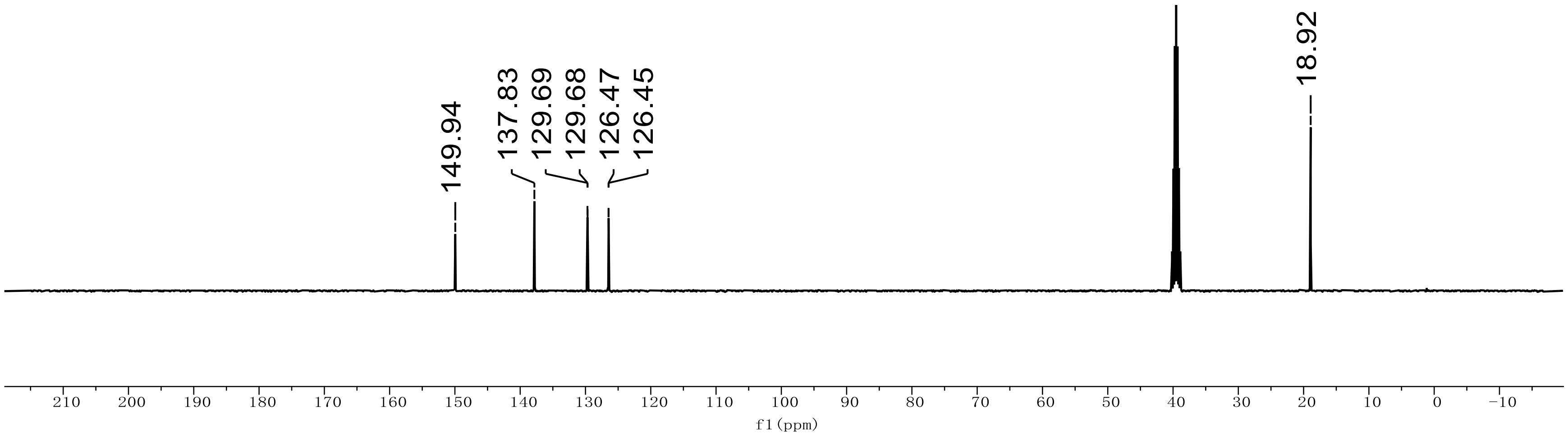

^{1}H NMR of 1k-S

DMSO-d_6, 400 MHz, 25 °C

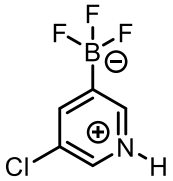

1k-S

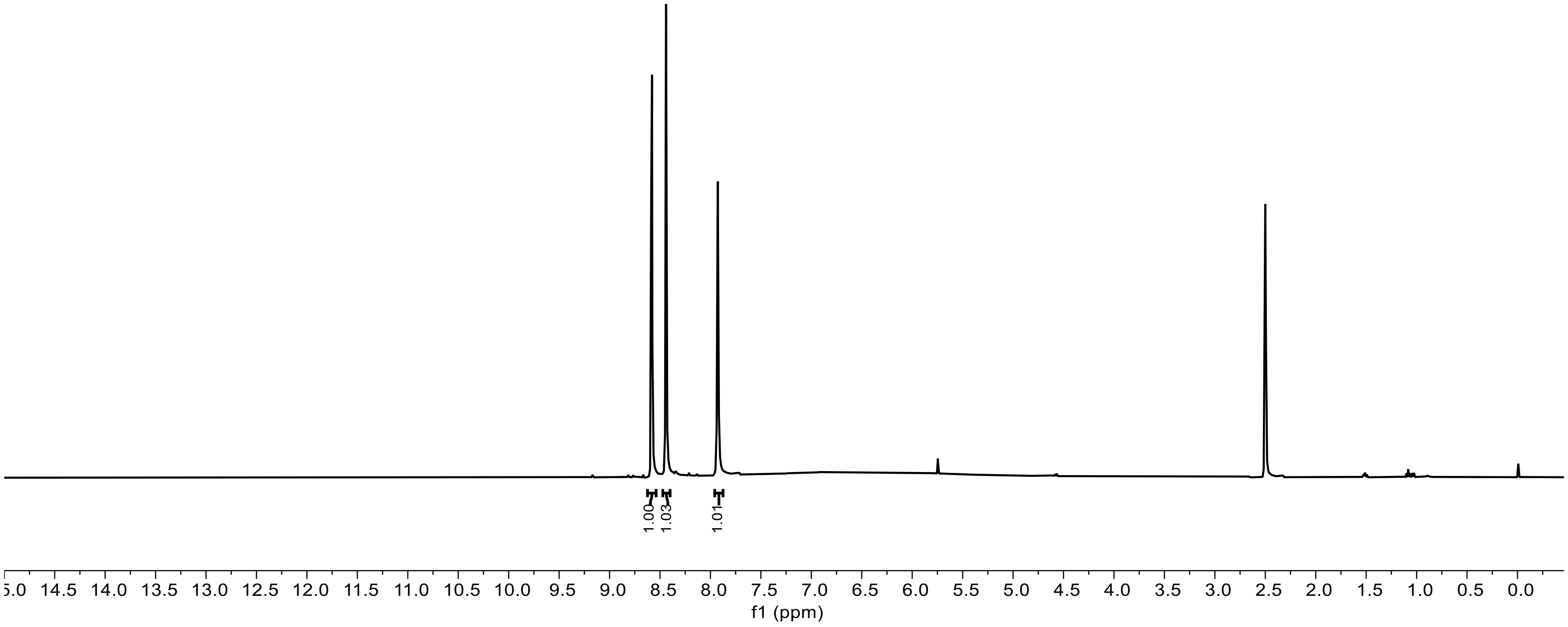

^{19}F NMR of 1k-S

CD_3CN, 376 MHz, 25 °C

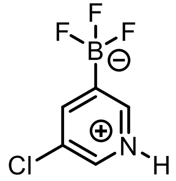

1k-S

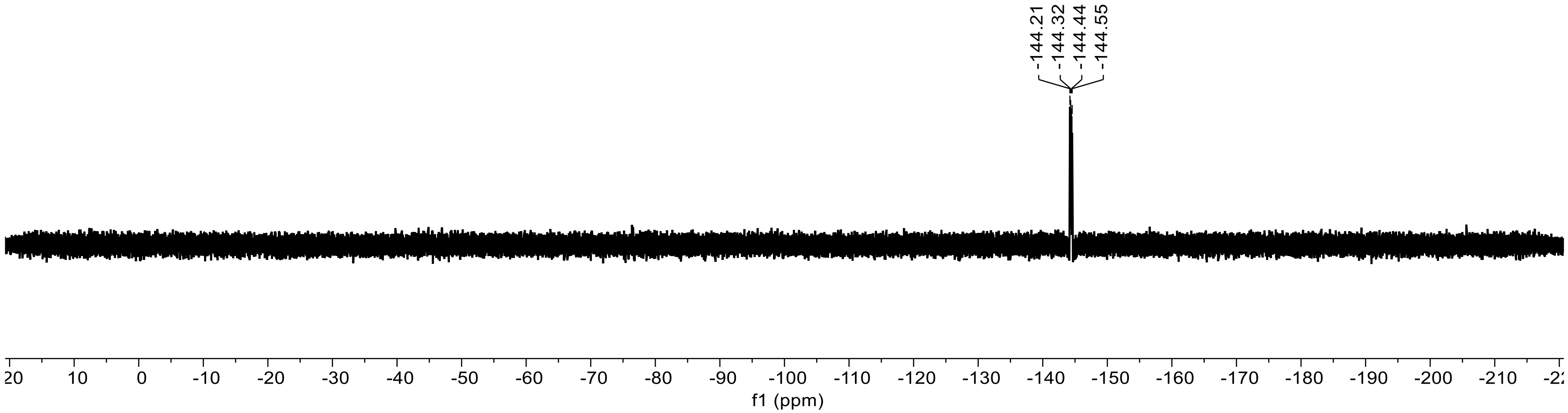

^{11}B NMR of 1k-S

CD_3CN, 128 MHz, 25 °C

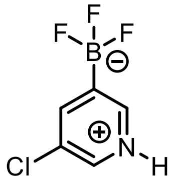

1k-S

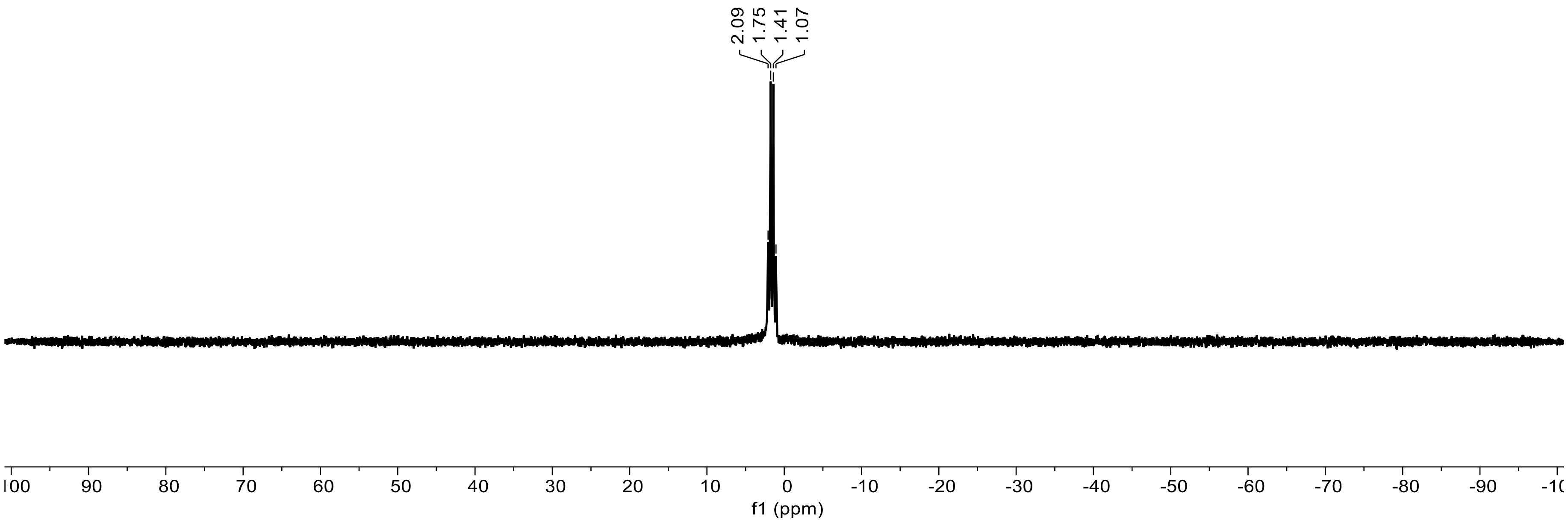

^{13}C NMR of 1k-S

CD_3CN, 101 MHz, 25 °C

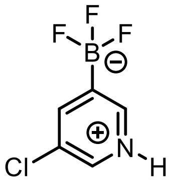

1k-S

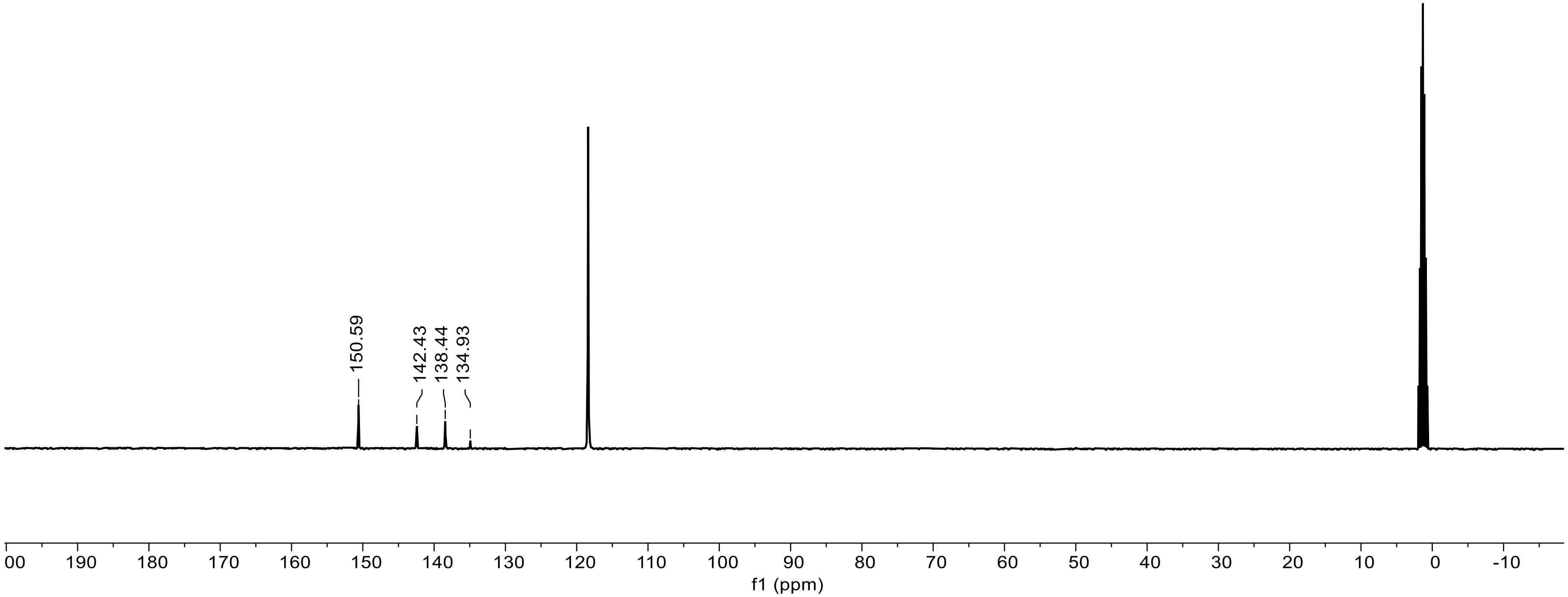

^{1}H NMR of 1m-S

CD_3CN, 400 MHz, 25 °C

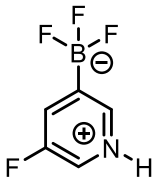

1m-S

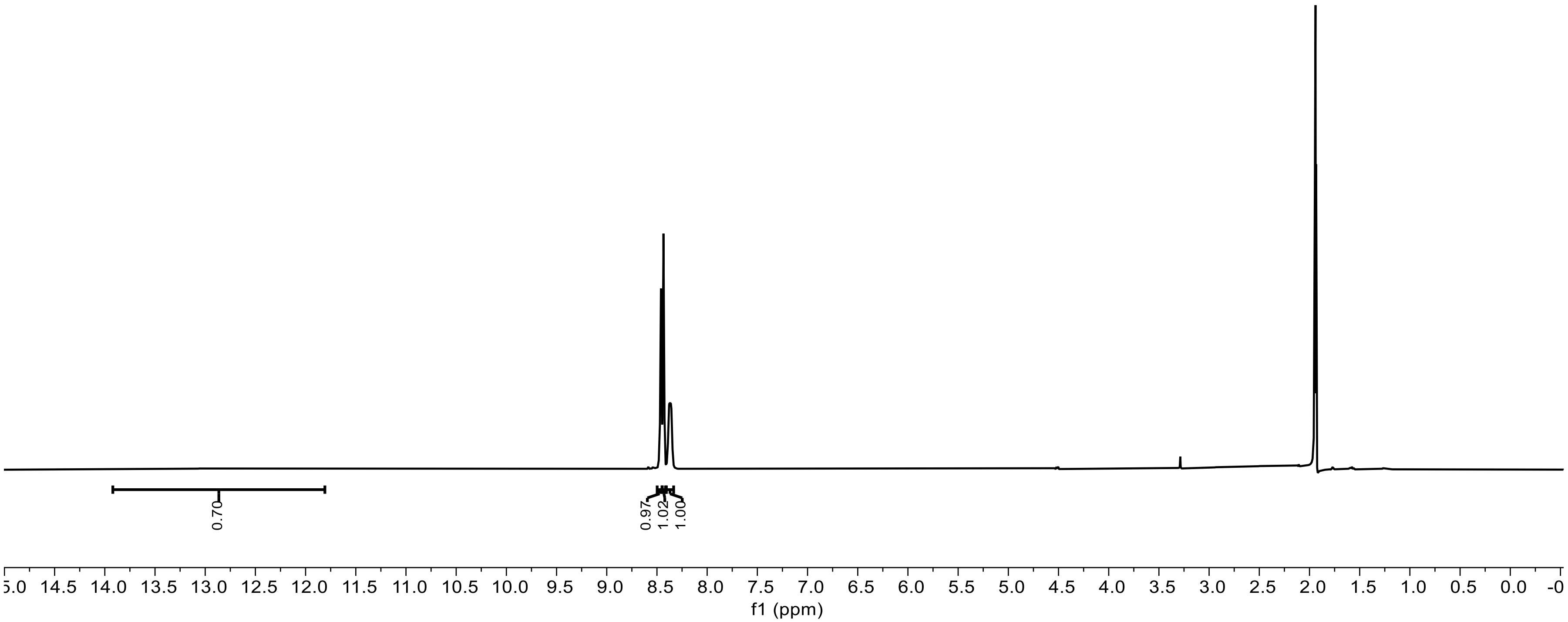

^{19}F NMR of 1m-S

DMSO-d_6, 376 MHz, 25 °C

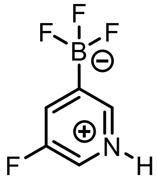

1m-S

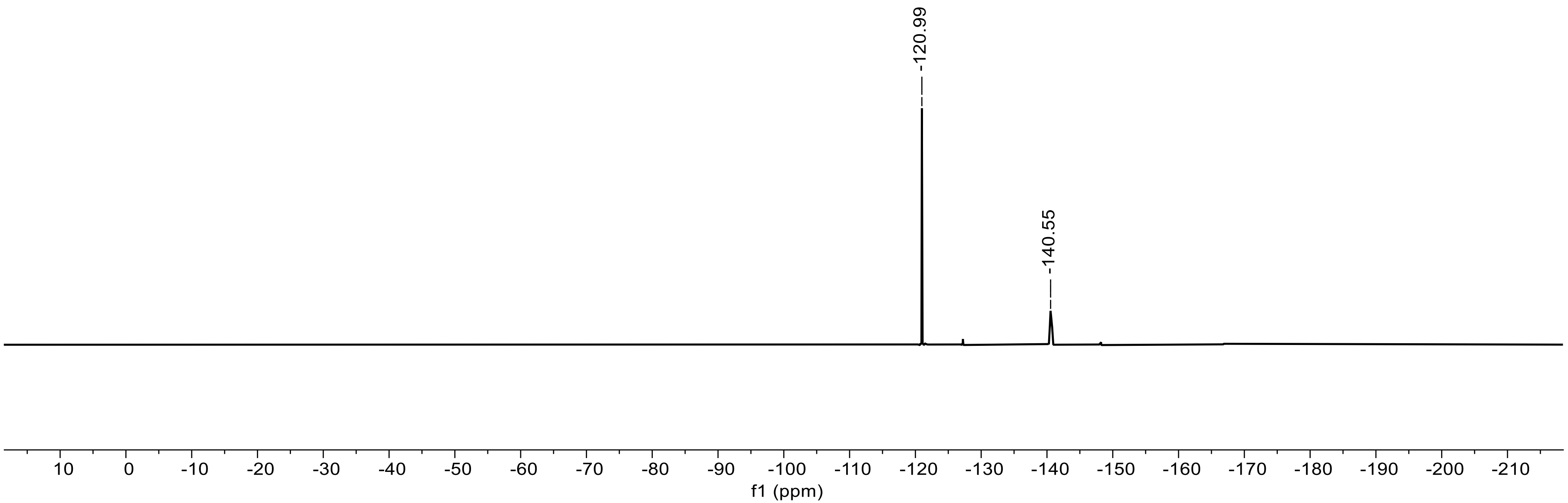

^{11}B NMR of 1m-S

DMSO-d_6, 128 MHz, 25 °C

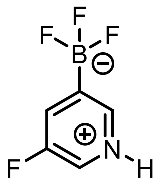

1m-S

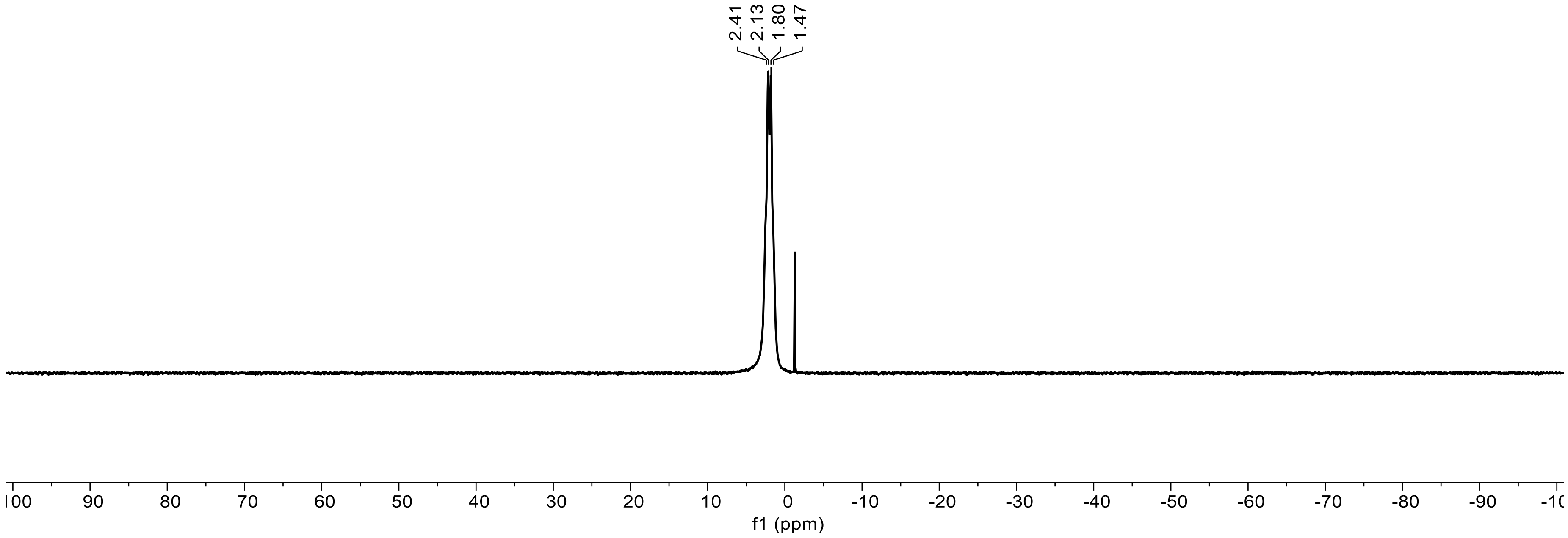

^{13}C NMR of 1m-S

DMSO-d_6, 101 MHz, 25 °C

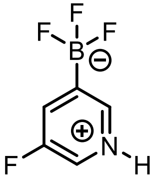

1m-S

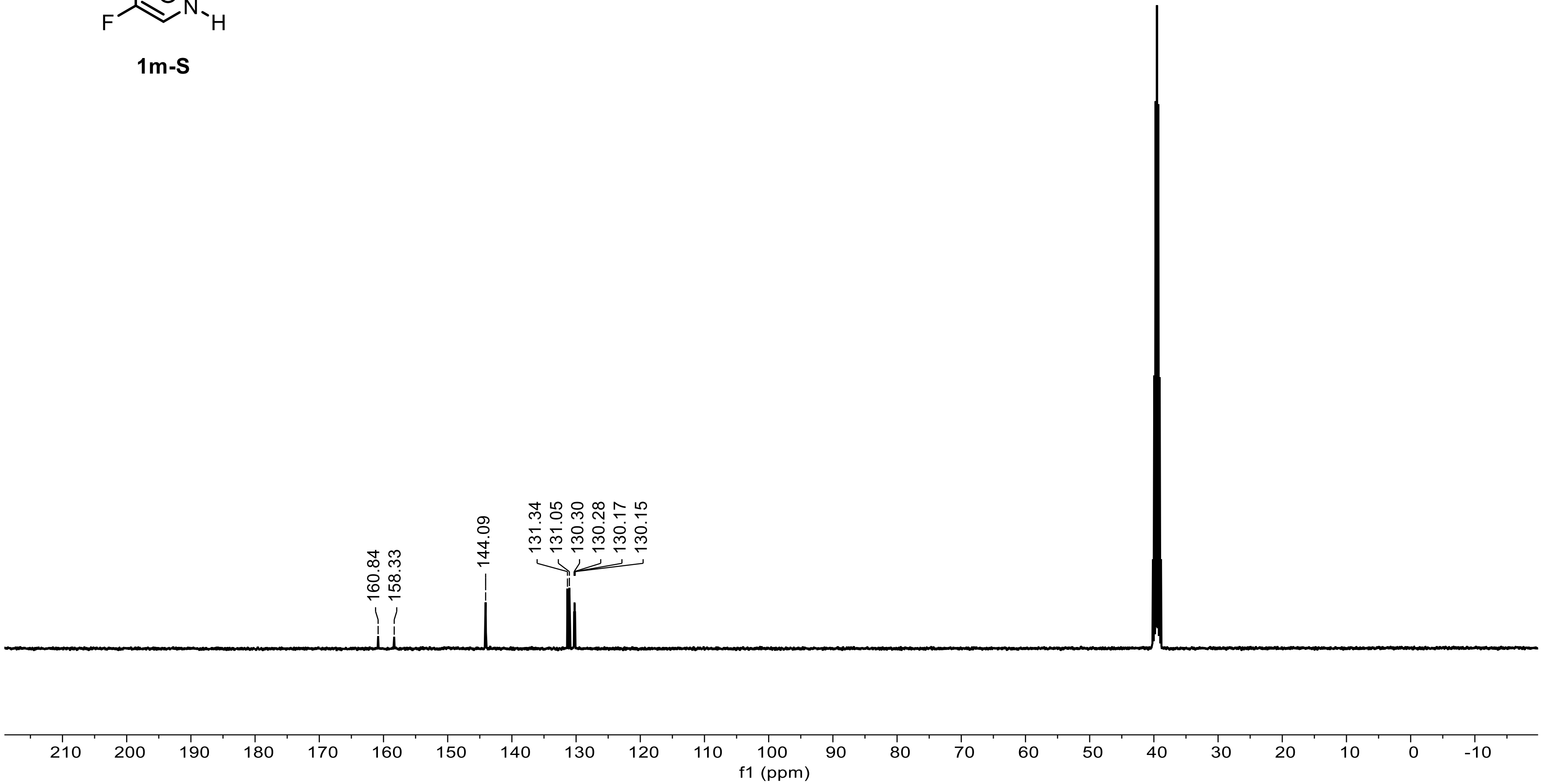

^{1}H NMR of 1p-S

CD₃CN, 400 MHz, 25 °C

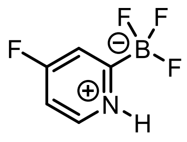

1p-S

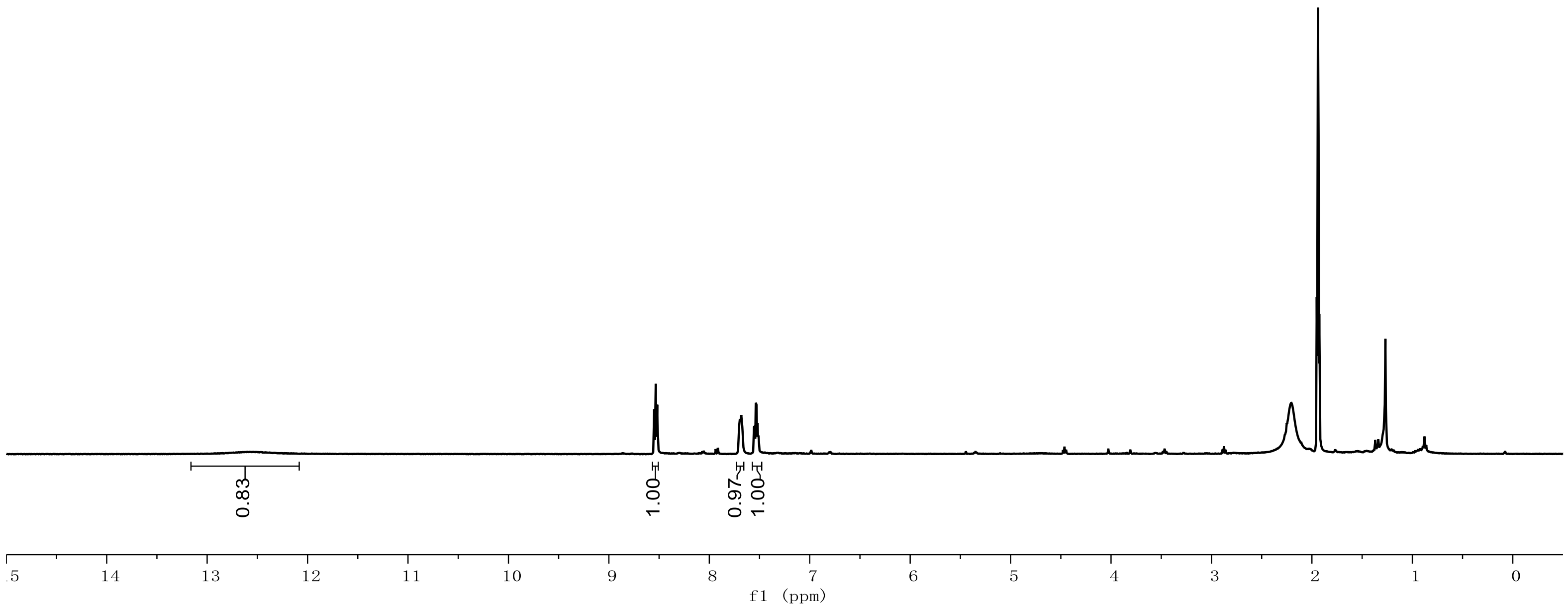

^{19}F NMR of 1p-S

CD_3CN, 376 MHz, 25 °C

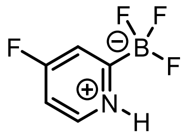

1p-S

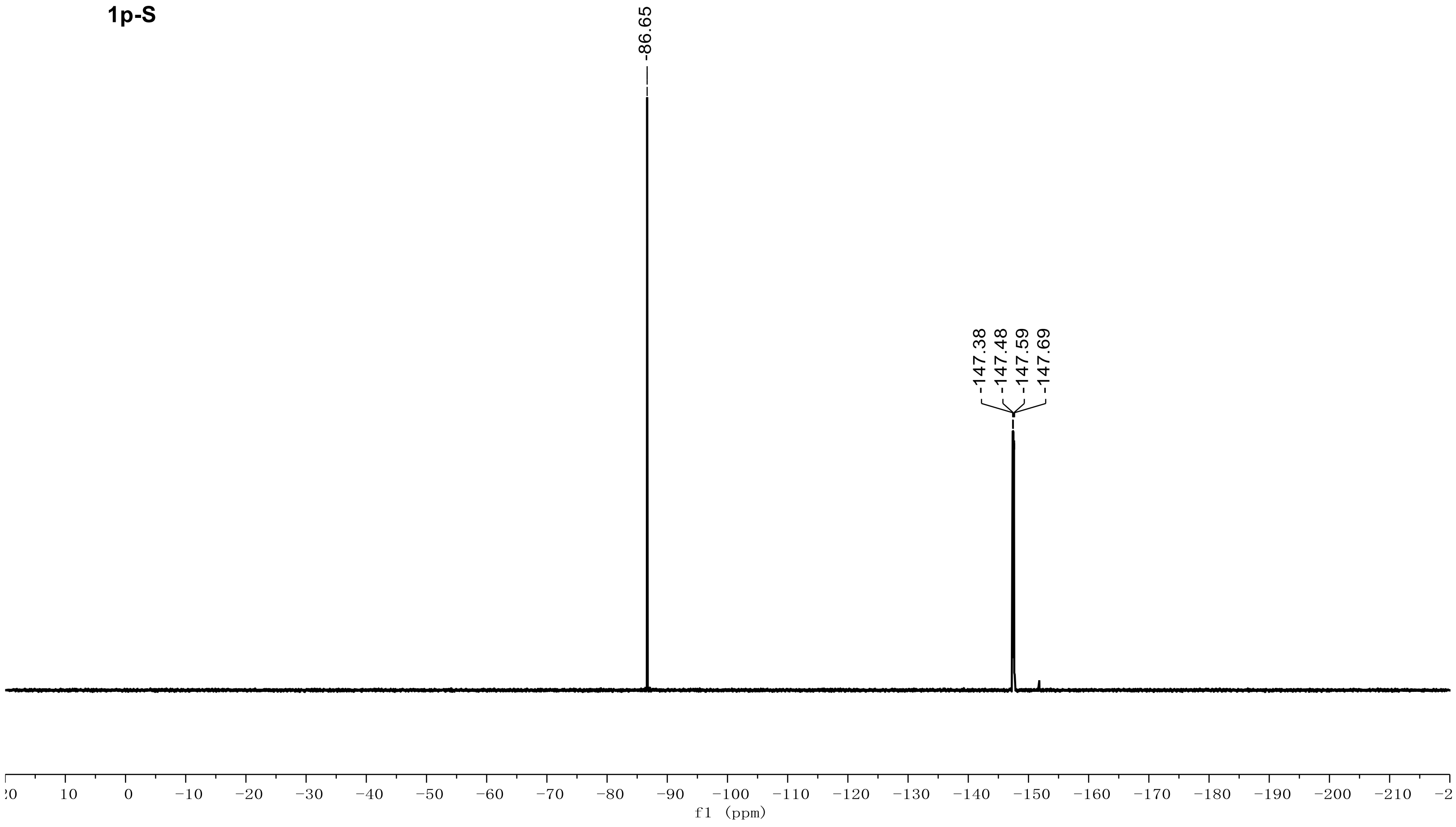

^{11}B NMR of 1p-S

CD_3CN, 128 MHz, 25 °C

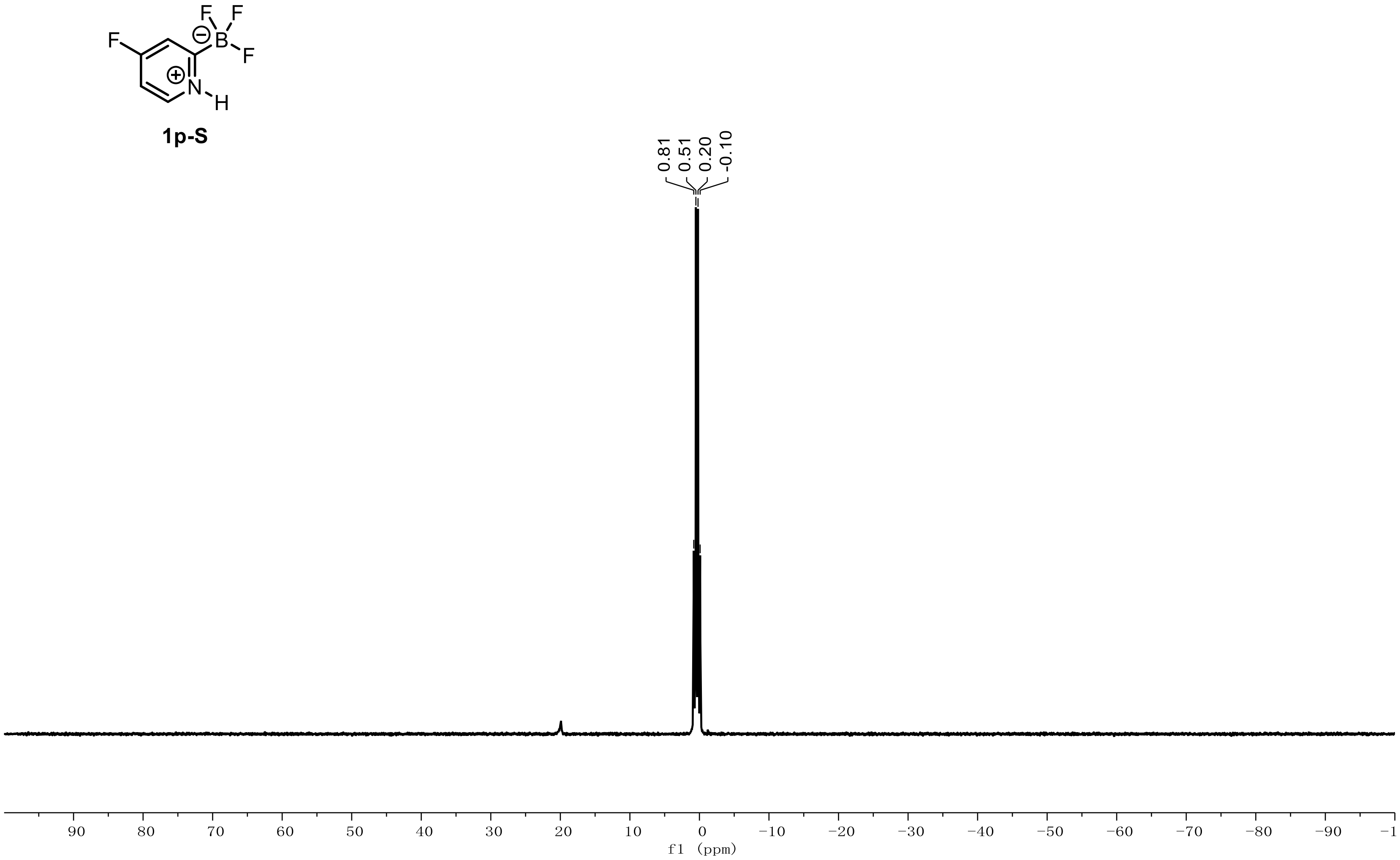

^{13}C NMR of 1p-S

CD_3CN, 101 MHz, 25 °C

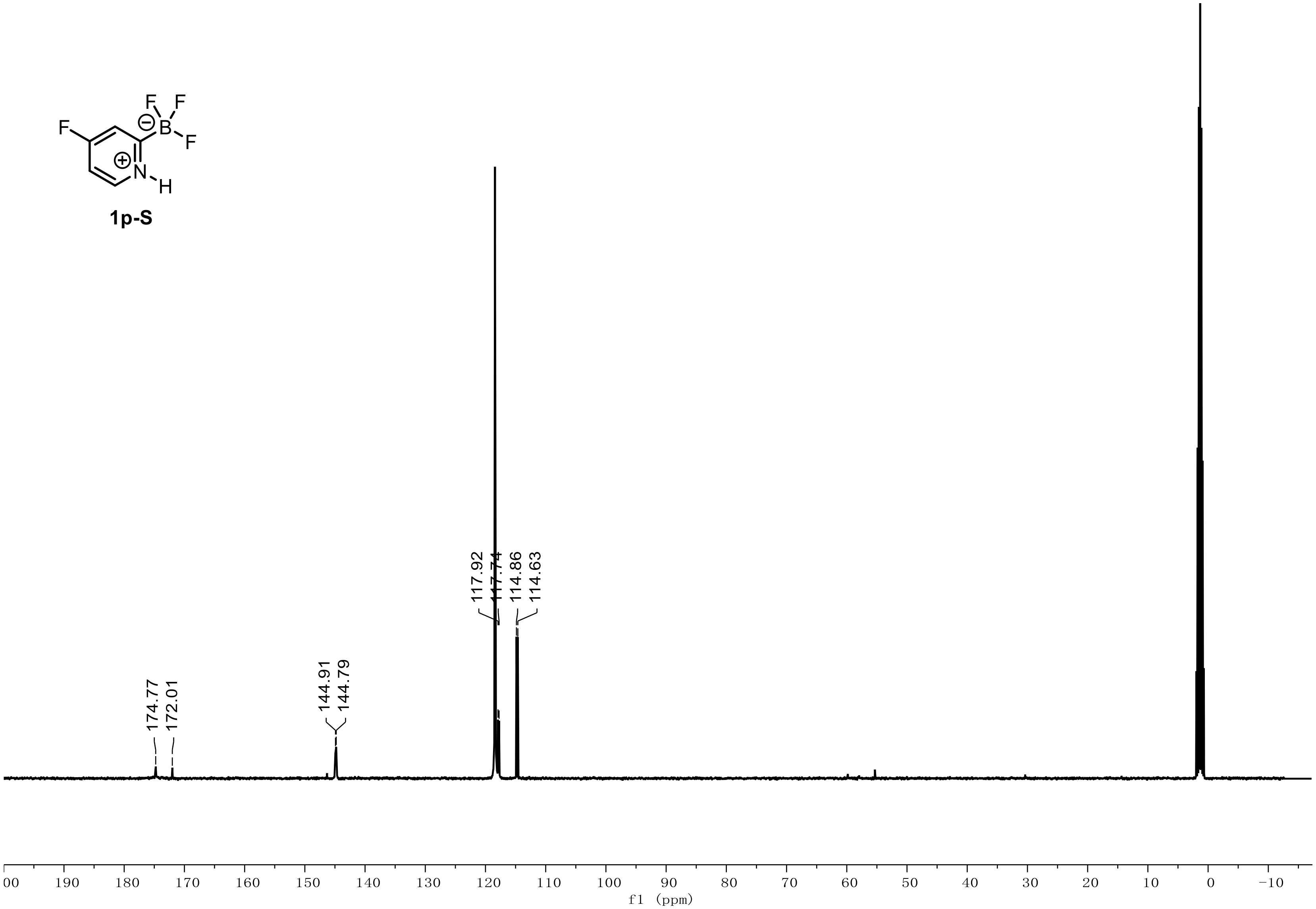

REFERENCES

1. G. R. Fulmer, A. J. M. Miller, N. H. Sherden, H. E. Gottlieb, A. Nudelman, B. M. Stoltz, J. E. Bercaw, K. I. Goldberg. NMR chemical shifts of trace Impurities: Common laboratory solvents, oganics, and gases in deuterated solvents relevant to the organometallic chemist. *Organometallics* **2010**, *29*, 2176–2179.

2. K. Barral, A. D. Moorhouse, J. E. Moses. Efficient conversion of aromatic amines into azides: a one-pot synthesis of triazole linkages. *Org. Lett.* **2007**, *9*, 1809–1811.

3. M. Kitamura, S. Kato, M. Yano, N. Tashiro, Y. Shiratake, M. Sando, T. Okauchi. A reagent for safe and efficient diazo-transfer to primary amines: 2-azido-1,3-dimethylimidazolinium hexafluorophosphate. *Org. Biomol. Chem.* **2014**, *12*, 4397.

4. M. Zheng, L. Cai, T. Ma, H. Tan, X. Lai, J. Dong, X. Xue. Computational analysis of modular diazotransfer reactions for the development of predictive reactivity models and diazotransfer reagents. *Nat. Synth*. **2024**, *3*, 1507–1517.

5. G. Meng, T. Guo, T. Ma, J. Zhang, Y. Shen, K. B. Sharpless, J. Dong. Modular click chemistry libraries for functional screens using a diazotizing reagent. *Nature* **2019**, *574*, 86–89.

6. S. Yoshida, T. Nonaka, T. Moritaa, T. Hosoya. Modular synthesis of bis- and tris-1,2,3-triazoles by permutable sequential azide–aryne and azide–alkyne cycloadditions. *Org*. *Biomol*. *Chem*. **2014**, *12*, 7489–7493.

7. M. Tanioka, F. Enomoto, F. Kitamura, R. Nakamura, A. Takano, S. Kanayama, A. Kohyama, T. Yamada, Y. Matsuya. Room-temperature ligation of iminophosphoranes with gem-difluoroalkenes. *J. Org. Chem.* **2025**, *90*, 3610–3614.

8. J. Ma, H. Xu, K. Hou, Y. Cao, D. Xie, J. Yan, W. Dong, T. Jiang, C. Chen. Design and synthesis of cyclic dinucleotide analogues containing triazolyl C-nucleosides. *J. Org. Chem*. **2024**, *89*, 11380–11393.

9. S. Graßl, J. Singer, P. Knochel. Iron-mediated electrophilic amination of organozinc halides using organic azides. *Angew. Chem. Int. Ed*. **2020**, *59*, 335–338.

10. D. Reddy, I. Novitskiy, A. Beloglazkina, A. Kutateladze. Oxidative control of photoinduced cascade electrocyclizations in aromatic azido imines to access complex fused imidazoles or pyrazoles. *Org. Lett*. **2024**, *26*, 2558–2563.

11. W. Yang, L. Xu, Z. Chen, L. Zhang, M. Miao, H. Ren. Ru-catalyzed synthesis of dihydrofuroquinolines from azido-cyclopropyl ketones. *Org. Lett*. **2013**, *15*, 1282–1285.

12. M. Belkheira, D. Abed, J. Pons, C. Bressy. Chemoselective organoclick–click sequence. *Synthesis*. **2018**, *50*, 4254–4262.

13. W. Li, Y. Chen, R. Yuan, X. Jia, Y. Yao, L. Zhang. Water-Stable 2-Pyridylboron Reagents: Pd-Catalyzed 2-Pyridylation Reaction of Aryl Halides. *Angew. Chem. Int. Ed.* **2025**, *64*, e202500315.

14. Á. Roxin, C. Zhang, S. Huh, M. L. Lepage, Z. Zhang, K. Lin, F. Bénard, D. M. Perrin. Preliminary evaluation of ^{18}F-labeled LLP2A-trifluoroborate conjugates as VLA-4 ($\alpha_4\beta_1$ integrin) specific radiotracers for PET imaging of melanoma. *Nucl Med Biol*. **2018**, *61*, 11–20.

15. X. Zhang, C. Jiang, L. Qi, C. Wu, C. Zhang. Enhancement of optical anisotropy from [BF_4] units by ligand substitution. *J. Organomet. Chem*. **2025**, *1038*, 123756.

16. Z. Liu, D. Chao, Y. Li, R. Ting, J. Oh, D. M. Perrin. From Minutes to Years: Predicting Organotrifluoroborate Solvolysis Rates. *Chem. Eur. J.* **2015**, *21*, 3924–3928.

17. E. J. L. Stéen, N. Nyberg, S. Lehel, V. L. Andersen, P. D. Pilato, G. M. Knudsen, J. L. Kristensen, M. M. Herth. Development of a simple proton nuclear magnetic resonance-based procedure to estimate the approximate distribution coefficient at physiological pH (log$D_{7.4}$): Evaluation and comparison to existing practices. *Bioorg. Med. Chem. Lett.* **2017**, *27*, 319–322.

18. M. J. Frisch, G. W. Trucks, H. B. Schlegel, G. E. Scuseria, M. A. Robb, J. R. Cheeseman, G. Scalmani, V. Barone, G. A. Petersson, H. Nakatsuji, X. Li, M. Caricato, A. V. Marenich, J. Bloino, B. G. Janesko, R. Gomperts, B. Mennucci, H. P. Hratchian, J. V. Ortiz, A. F. Izmaylov, J. L. Sonnenberg, D. Williams-Young, F. Ding, F. Lipparini, F. Egidi, J. Goings, B. Peng, A. Petrone, T. Henderson, D. Ranasinghe, V. G. Zakrzewski, J. Gao, N. Rega, G. Zheng, W. Liang, M. Hada, M. Ehara, K. Toyota, R. Fukuda, J. Hasegawa, M. Ishida, T. Nakajima, Y. Honda, O. Kitao, H. Nakai, T. Vreven, K. Throssell, J. A. Montgomery, Jr., J. E. Peralta, F. Ogliaro, M. J. Bearpark, J. J. Heyd, E. N. Brothers, K. N. Kudin, V. N. Staroverov, T. A. Keith, R. Kobayashi, J. Normand, K. Raghavachari, A. P. Rendell, J. C. Burant, S. S. Iyengar, J. Tomasi, M. Cossi, J. M. Millam, M. Klene, C. Adamo, R. Cammi, J. W. Ochterski, R. L. Martin, K. Morokuma, O. Farkas, J. B. Foresman, D. J. Fox, Gaussian, Inc., Wallingford CT, 2016.

19. C. Adamo, V. Barone. Toward reliable density functional methods without adjustable parameters: The PBE0 model. *J. Chem. Phys.* **1999**, *110*, 6158–6170.

20. S. Grimme, J. Antony, S. Ehrlich, H. Krieg. A consistent and accurate ab initio parametrization of density functional dispersion correction (DFT-D) for the 94 elements H–Pu. *J. Chem. Phys.* **2010**, *132*, 154104.

21. S. Grimme, S. Ehrlich, L. Goerigk. Effect of the damping function in dispersion corrected density functional theory. *J. Comput. Chem.* **2011**, *32*, 1456–1465.

22. F. Weigend, R. Ahlrichs. Balanced basis sets of split valence, triple zeta valence and quadruple zeta valence quality for H to Rn: Design and assessment of accuracy. *Phys. Chem. Chem. Phys.* **2005**, *7*, 3297–3305.

23. V. Barone, M. Cossi, Quantum calculation of molecular energies and energy gradients in solution by a conductor solvent model, *J. Phys. Chem. A* **1998**, *102*, 1995–2001.

24. A. Lennox, G. Lloyd-Jones. Organotrifluoroborate hydrolysis: boronic acid release mechanism and an acid-base paradox in cross-coupling. *J. Am. Chem. Soc*. **2012**, 134, 7431–7441.

25. K. Chansaenpak, M. Wang, Z. Wu, R. Zaman, Z. Li, F. Gabbaï. [^{18}F]–NHC–BF_3 adducts as water stable radio-prosthetic groups for PET imaging. *Chem. Commun*. **2015**, 51, 12439–12442.

26. J. Lozada, W. Lin, R. Cao-Shen, R. Tai, D. Perrin. Salt metathesis: Tetrafluoroborate anion rapidly fluoridates organoboronic acids to give organotrifluoroborates. *Angew. Chem. Int. Ed.* **2023**, 62, e202215371.
