# Supplementary material for: Versatile Pyridinium Trifluoroborate Platform for Facile Preparation of 18F‑Labeled PET Tracers in Water
Source: ACS Cent Sci. 2026 May 6;12(6):824–30. doi: 10.1021/acscentsci.6c00164 (PMC13306595; doi:10.1021/acscentsci.6c00164)

checkCIF/PLATON report

Structure factors have been supplied for datablock(s) g250402e_auto

THIS REPORT IS FOR GUIDANCE ONLY. IF USED AS PART OF A REVIEW PROCEDURE FOR PUBLICATION, IT SHOULD NOT REPLACE THE EXPERTISE OF AN EXPERIENCED CRYSTALLOGRAPHIC REFEREE.

No syntax errors found. CIF dictionary Interpreting this report

Datablock: g250402e_auto

Bond precision:	C-C = 0.0030 A		Wavelength=0.71073
Cell:	a=9.0164(13)	b=13.8720(16)	c=15.813(2)
	alpha=90	beta=90	gamma=90
Temperature:	223 K		

	Calculated	Reported
Volume	1977.8(4)	1977.8(5)
Space group	P b c a	P b c a
Hall group	-P 2ac 2ab	-P 2ac 2ab
Moiety formula	C9 H9 B F3 N O	C9 H9 B F3 N O
Sum formula	C9 H9 B F3 N O	C9 H9 B F3 N O
Mr	214.98	214.98
Dx,g cm-3	1.444	1.444
Z	8	8
Mu (mm-1)	0.130	0.130
F000	880.0	880.0
F000'	880.65	
h,k,lmax	12,19,22	12,18,20
Nref	2979	2607
Tmin,Tmax	0.949,0.962	0.842,1.000
Tmin'	0.949	

Correction method= # Reported T Limits: Tmin=0.842 Tmax=1.000
AbsCorr = MULTI-SCAN

Data completeness= 0.875 Theta(max)= 30.340

R(reflections)= 0.0500(1513) wR2(reflections)= 0.1415(2607)

S = 1.054 Npar= 137

The following ALERTS were generated. Each ALERT has the format
test-name_ALERT_alert-type_alert-level.
Click on the hyperlinks for more details of the test.

Alert level G

```
PLAT912_ALERT_4_G Missing # of FCF Reflections Above STh/L=  0.600        327 Note
PLAT941_ALERT_3_G Average HKL Measurement Multiplicity ..........         3.0 Low
PLAT952_ALERT_5_G Calculated (ThMax) and CIF-Reported Lmax Differ.          2 Units
PLAT958_ALERT_1_G Calculated (ThMax) and Actual (FCF) Lmax Differ.          2 Units
PLAT969_ALERT_5_G The 'Henn et al.' R-Factor-gap value ..........       2.975 Note
              Predicted wR2: Based on SigI**2  4.76 or SHELX Weight 13.42
PLAT978_ALERT_2_G Number C-C Bonds with Positive Residual Density.          1 Info
```

0 **ALERT level A** = Most likely a serious problem - resolve or explain
0 **ALERT level B** = A potentially serious problem, consider carefully
0 **ALERT level C** = Check. Ensure it is not caused by an omission or oversight
6 **ALERT level G** = General information/check it is not something unexpected

1 ALERT type 1 CIF construction/syntax error, inconsistent or missing data
1 ALERT type 2 Indicator that the structure model may be wrong or deficient
1 ALERT type 3 Indicator that the structure quality may be low
1 ALERT type 4 Improvement, methodology, query or suggestion
2 ALERT type 5 Informative message, check

It is advisable to attempt to resolve as many as possible of the alerts in all categories. Often the minor alerts point to easily fixed oversights, errors and omissions in your CIF or refinement strategy, so attention to these fine details can be worthwhile. In order to resolve some of the more serious problems it may be necessary to carry out additional measurements or structure refinements. However, the purpose of your study may justify the reported deviations and the more serious of these should normally be commented upon in the discussion or experimental section of a paper or in the "special_details" fields of the CIF. checkCIF was carefully designed to identify outliers and unusual parameters, but every test has its limitations and alerts that are not important in a particular case may appear. Conversely, the absence of alerts does not guarantee there are no aspects of the results needing attention. It is up to the individual to critically assess their own results and, if necessary, seek expert advice.

Publication of your CIF in IUCr journals

A basic structural check has been run on your CIF. These basic checks will be run on all CIFs submitted for publication in IUCr journals (*Acta Crystallographica*, *Journal of Applied Crystallography*, *Journal of Synchrotron Radiation*); however, if you intend to submit to *Acta Crystallographica Section C* or *E* or *IUCrData*, you should make sure that full publication checks are run on the final version of your CIF prior to submission.

Publication of your CIF in other journals

Please refer to the *Notes for Authors* of the relevant journal for any special instructions relating to CIF submission.

PLATON version of 02/02/2025; check.def file version of 02/02/2025

Datablock g250402e_auto - ellipsoid plot

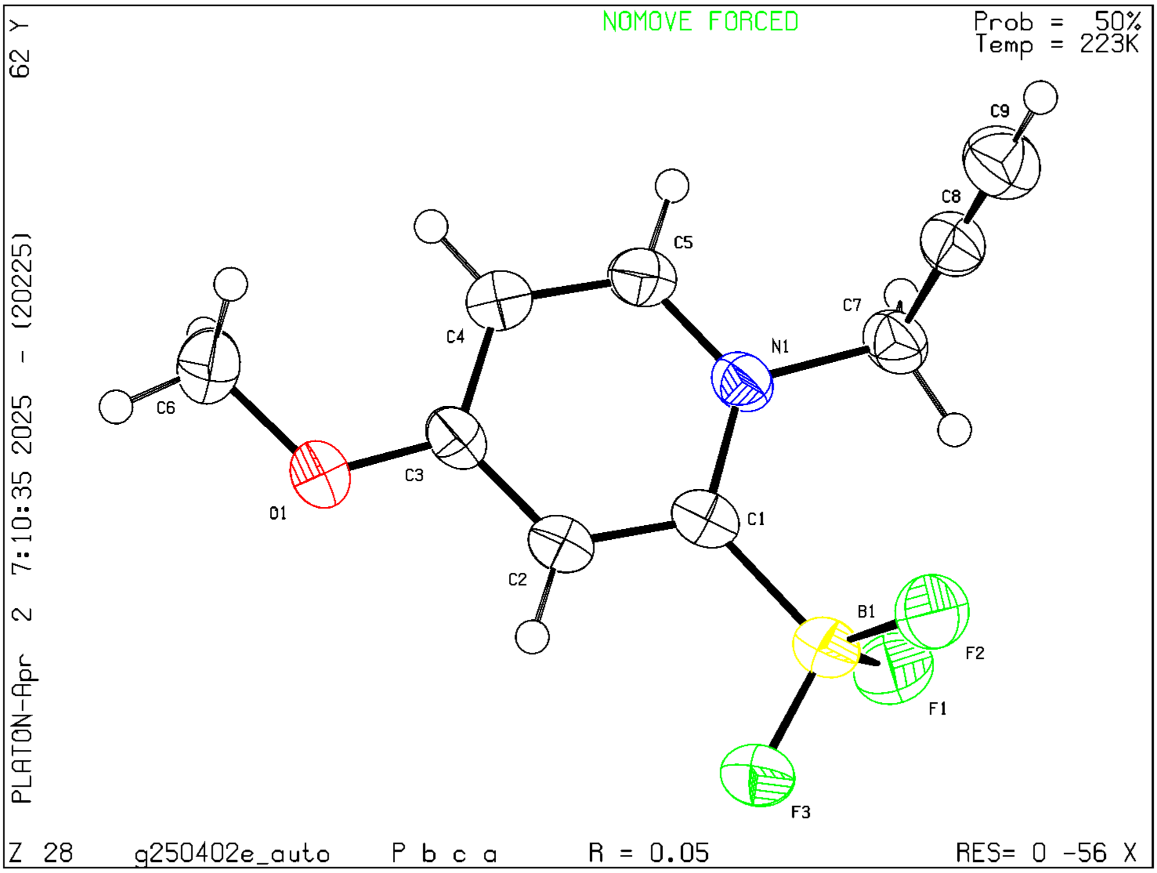

Supplement: Supplementary file 2 [file oc6c00164_si_002.zip › X-Ray Data/checkcif-2440358.pdf]
