# Supplementary material for: Versatile Pyridinium Trifluoroborate Platform for Facile Preparation of 18F‑Labeled PET Tracers in Water
Source: ACS Cent Sci. 2026 May 6;12(6):824–30. doi: 10.1021/acscentsci.6c00164 (PMC13306595; doi:10.1021/acscentsci.6c00164)

checkCIF/PLATON report

Structure factors have been supplied for datablock(s) b250604a_0m_a

THIS REPORT IS FOR GUIDANCE ONLY. IF USED AS PART OF A REVIEW PROCEDURE FOR PUBLICATION, IT SHOULD NOT REPLACE THE EXPERTISE OF AN EXPERIENCED CRYSTALLOGRAPHIC REFEREE.

No syntax errors found. CIF dictionary Interpreting this report

Datablock: b250604a_0m_a

```
Bond precision:   C-C = 0.0040 A          Wavelength=0.71073

Cell:             a=6.6841(13)      b=15.985(3)        c=7.8753(13)
                  alpha=90          beta=93.887(5)     gamma=90
Temperature:      223 K

                 Calculated                   Reported
Volume           839.5(3)                     839.5(3)
Space group      P 21/c                       P 1 21/c 1
Hall group       -P 2ybc                      -P 2ybc
Moiety formula   C8 H7 B F3 N                 C8 H7 B F3 N
Sum formula      C8 H7 B F3 N                 C8 H7 B F3 N
Mr               184.96                       184.96
Dx,g cm-3        1.463                        1.463
Z                4                            4
Mu (mm-1)        0.132                        0.132
F000             376.0                        376.0
F000'            376.28
h,k,lmax         8,20,10                      8,20,9
Nref             1927                         1877
Tmin,Tmax        0.974,0.974                  0.544,0.746
Tmin'            0.974

Correction method= # Reported T Limits: Tmin=0.544 Tmax=0.746
AbsCorr = NONE

Data completeness= 0.974          Theta(max)= 27.482

                                                   wR2(reflections)=
R(reflections)= 0.0699( 1304)                      0.1919( 1877)

S = 1.107                  Npar= 146
```

The following ALERTS were generated. Each ALERT has the format
test-name_ALERT_alert-type_alert-level.
Click on the hyperlinks for more details of the test.

Alert level C

```
PLAT213_ALERT_2_C Atom F6              has ADP max/min Ratio .....         3.4 prolat
PLAT234_ALERT_4_C Large Hirshfeld Difference F5       --B1       .        0.18 Ang.
PLAT242_ALERT_2_C Low    'MainMol' Ueq as Compared to Neighbors of          B1 Check
PLAT906_ALERT_3_C Large K Value in the Analysis of Variance ......       9.666 Check
PLAT911_ALERT_3_C Missing FCF Refl Between Thmin & STh/L=    0.600          14 Report
                1  0  0,   1  3  0,   0  4  0,   1  2  1,  -1  4  1,   1  4  1,
               -1  0  2,   0  0  2,   0  1  2,  -1  2  2,  -4  0  8,  -1  1  9,
                0  1  9,   1  1  9,
PLAT913_ALERT_3_C Missing # of Very Strong Reflections in FCF ....           7 Note
                1  3  0,   1  2  1,   1  4  1,  -1  0  2,   0  0  2,   0  1  2,
               -1  2  2,
```

Alert level G

```
PLAT002_ALERT_2_G Number of Distance or Angle Restraints on AtSite           7 Note
PLAT003_ALERT_2_G Number of Uiso or U(i,j)  Restrained non-H-Atoms           1 Report
PLAT176_ALERT_4_G The CIF-Embedded .res File Contains SADI Records           3 Report
PLAT186_ALERT_4_G The CIF-Embedded .res File Contains ISOR Records           1 Report
PLAT187_ALERT_4_G The CIF-Embedded .res File Contains RIGU Records           2 Report
PLAT190_ALERT_3_G A Non-default RIGU Restraint Value for First Par      0.0010 Report
PLAT190_ALERT_3_G A Non-default RIGU Restraint Value for SecondPar      0.0010 Report
PLAT190_ALERT_3_G A Non-default RIGU Restraint Value for First Par      0.0010 Report
PLAT190_ALERT_3_G A Non-default RIGU Restraint Value for SecondPar      0.0010 Report
PLAT301_ALERT_3_G Main Residue  Disorder .............(Resd    1)         23% Note
PLAT432_ALERT_2_G Short Inter X...Y Contact  F3       ..C1       .        2.85 Ang.
                                             x,3/2-y,-1/2+z  =      4_575 Check
PLAT432_ALERT_2_G Short Inter X...Y Contact  F4       ..C8       .        2.70 Ang.
                                           -1+x,3/2-y,1/2+z  =      4_476 Check
PLAT860_ALERT_3_G Number of Least-Squares Restraints ............           9 Note
PLAT883_ALERT_1_G Absent Datum for _atom_sites_solution_primary ..      Please Do !
PLAT912_ALERT_4_G Missing # of FCF Reflections Above STh/L=  0.600          20 Note
PLAT969_ALERT_5_G The 'Henn et al.' R-Factor-gap value ..........       3.595 Note
              Predicted wR2: Based on SigI**2  5.34 or SHELX Weight 17.33
PLAT978_ALERT_2_G Number C-C Bonds with Positive Residual Density.           0 Info
```

0 **ALERT level A** = Most likely a serious problem - resolve or explain
0 **ALERT level B** = A potentially serious problem, consider carefully
6 **ALERT level C** = Check. Ensure it is not caused by an omission or oversight
17 **ALERT level G** = General information/check it is not something unexpected

1 ALERT type 1 CIF construction/syntax error, inconsistent or missing data
7 ALERT type 2 Indicator that the structure model may be wrong or deficient
9 ALERT type 3 Indicator that the structure quality may be low
5 ALERT type 4 Improvement, methodology, query or suggestion
1 ALERT type 5 Informative message, check

Validation response form

Please find below a validation response form (VRF) that can be filled in and pasted into your CIF.

```
# start Validation Reply Form
_vrf_PLAT213_b250604a_0m_a
;
PROBLEM: Atom F6              has ADP max/min Ratio .....        3.4 prolat
RESPONSE: ...
;
_vrf_PLAT234_b250604a_0m_a
;
PROBLEM: Large Hirshfeld Difference F5       --B1       .       0.18 Ang.
RESPONSE: ...
;
_vrf_PLAT242_b250604a_0m_a
;
PROBLEM: Low    'MainMol' Ueq as Compared to Neighbors of         B1 Check
RESPONSE: ...
;
_vrf_PLAT906_b250604a_0m_a
;
PROBLEM: Large K Value in the Analysis of Variance ......      9.666 Check
RESPONSE: ...
;
_vrf_PLAT911_b250604a_0m_a
;
PROBLEM: Missing FCF Refl Between Thmin & STh/L=    0.600         14 Report
RESPONSE: ...
;
_vrf_PLAT913_b250604a_0m_a
;
PROBLEM: Missing # of Very Strong Reflections in FCF ....          7 Note
RESPONSE: ...
;
# end Validation Reply Form
```

It is advisable to attempt to resolve as many as possible of the alerts in all categories. Often the minor alerts point to easily fixed oversights, errors and omissions in your CIF or refinement strategy, so attention to these fine details can be worthwhile. In order to resolve some of the more serious problems it may be necessary to carry out additional measurements or structure refinements. However, the purpose of your study may justify the reported deviations and the more serious of these should normally be commented upon in the discussion or experimental section of a paper or in the "special_details" fields of the CIF. checkCIF was carefully designed to identify outliers and unusual parameters, but every test has its limitations and alerts that are not important in a particular case may appear. Conversely, the absence of alerts does not guarantee there are no aspects of the results needing attention. It is up to the individual to critically assess their own results and, if necessary, seek expert advice.

Publication of your CIF in IUCr journals

A basic structural check has been run on your CIF. These basic checks will be run on all CIFs submitted for publication in IUCr journals (*Acta Crystallographica*, *Journal of Applied Crystallography*, *Journal of Synchrotron Radiation*); however, if you intend to submit to *Acta Crystallographica Section C* or *E* or *IUCrData*, you should make sure that full publication checks are run on the final version of your CIF prior to submission.

Publication of your CIF in other journals

Please refer to the *Notes for Authors* of the relevant journal for any special instructions relating to CIF submission.

PLATON version of 02/02/2025; check.def file version of 02/02/2025

Datablock b250604a_0m_a - ellipsoid plot

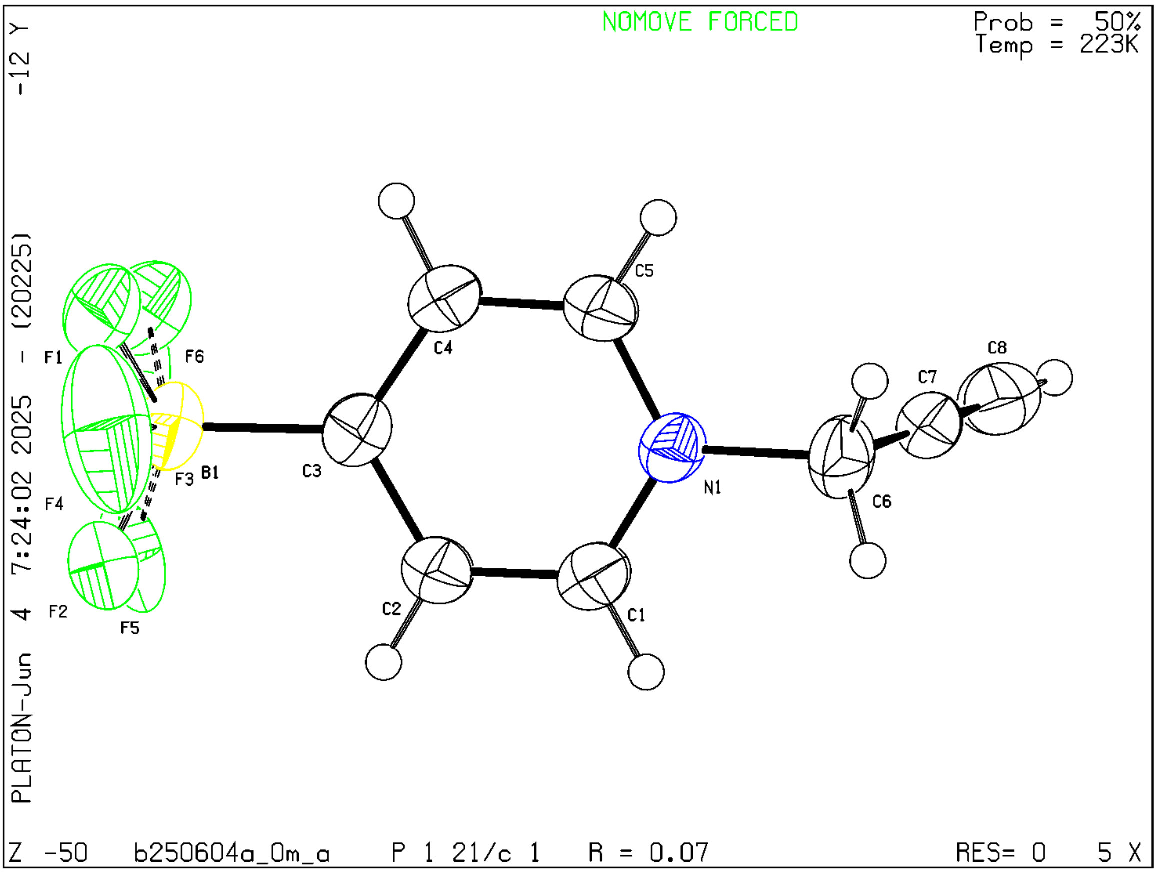

Supplement: Supplementary file 2 [file oc6c00164_si_002.zip › X-Ray Data/checkcif-2456372.pdf]
